# Nickel(0)-catalyzed divergent reactions of silacyclobutanes with internal alkynes

**DOI:** 10.1038/s41467-022-31006-y

**Published:** 2022-06-13

**Authors:** Xi-Chao Wang, Bo Li, Cheng-Wei Ju, Dongbing Zhao

**Affiliations:** State Key Laboratory and Institute of Elemento-Organic Chemistry, Haihe Laboratory of Sustainable Chemical Transformations, College of Chemistry, Nankai University, Tianjin, 300071 China

**Keywords:** Synthetic chemistry methodology, Stereochemistry

## Abstract

Transition metal-catalyzed reactions of silacyclobutanes with a variety of π units have attracted much attention and become one of the most straightforward and efficient approaches to rapidly access structurally diverse organosilicon compounds. However, the reaction of silacyclobutanes with alkynes still suffers from some limitations: (1) internal alkynes remain challenging substrates; (2) expensive Pd- or Rh-based catalysts have been employed in all existing systems; (3) controlling chemodivergence has not yet been realized. Herein we realize Ni-catalyzed chemodivergent reactions of silacyclobutanes with alkynes. In comparison with the previous Pd or Rh catalytic systems, our Ni-catalytic system features: 1) complementary substrate scope; 2) ligand-controlled chemodivergence; 3) low cost. The ligand precisely dictates the pathway selectivity, leading to the divergent formation of (benzo)silacyclohexenes and allyl vinylsilanes. Moreover, we demonstrate that employment of a chiral phosphine ligand is capable of forming silicon-stereogenic allyl vinylsilanes in high yields and enantioselectivities. In addition, DFT calculation is performed to elucidate the origin of the switchable selectivities, which is mainly attributed to different ligand steric effects.

## Abstract (web summary)

Transition metal-catalyzed reactions of silacyclobutanes to access structurally diverse organosilicon compounds have attracted attention in recent years. Here, the authors show nickel-catalysed, ligand-controlled chemodivergent reactions of these compounds with alkynes to (benzo)silacyclohexenes and allyl vinylsilane.

## Introduction

The discovery of general and efficient methodologies to approach silicon-containing organic compounds^1^, including chiral silicons^2–10^, has attracted great interest. Because these molecules are fundamentally important with a multitude of applications ranging from organic synthesis, materials science to medicinal chemistry^11–16^. Sila-substitution often leads to the improvement of the potency and ADMET properties of drug-like candidates^17,18^.

**Fig. 1:**
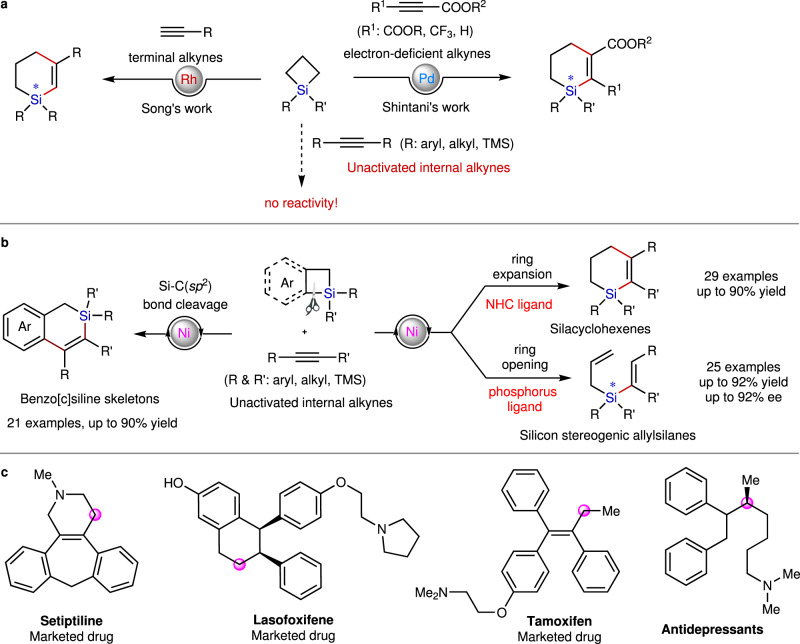
Research background and our work on the reaction of SCBs and benzosilacyclobutenes with alkynes. **a** Pd or Rh-catalyzed reaction of SCBs with alkynes in literature. **b** This work for Ni-catalyzed divergent reactions of SCBs with alkynes. **c** Medicinally relevant molecules possessing vicinal-diaryl structures. The potential “C/Si switch” site in those vicinal-diaryl structures has been highlighted by pink ball.

Silacyclobutanes (SCBs), which was first synthesized by Kipping^19–21^, has proven to possess unique reactivity and served as synthetic linchpin to rapidly access a variety of organosilicon compounds including chiral silicon arising from their high ring strain and enhanced Lewis acidity^22–26^. For example, low valent transition metals can facilely insert into the SCBs to form 5-membered silametallacycles, which is capable of undergoing the migratory insertion of a variety of π units into the M−Si bonds, cultivated in various fascinating transition metal-catalyzed reactions. Within this context, in 1975, Sakurai and Imai realized the first cycloaddition of the SCBs with alkynyl carboxylates or phenylacetylene by use of Pd-catalytic system, but giving the mixture of silacyclohexenes and allyl vinylsilanes^27,28^, which severely hampers their practical applications. In 2012, Shintani et al. made an important breakthrough in the cycloaddition of the SCBs with alkynyl carboxylates (Fig. 1a, right)^29^. They disclosed that a palladium catalyst coordinated with a sterically demanding chiral phosphoramidite ligand could facilitate the reductive elimination step to highly chemoselective and enantioselective formation of the enantioenriched silicon-stereogenic silacyclohexenes. Unfortunately, less electron-deficient alkynes such as phenylacetylenes and diphenylacetylenes are clearly incompatible. Until recently, Song and Xu independently have achieved the first highly efficient and chemoselective cycloaddition of the SCBs with alkynes to produce the silacyclohexenes by employment of Rh-catalyzed condition (Fig. 1a, left)^25,30^. Moreover, Song et al. further proved that the use of chiral phosphoramidite ligand during the reaction is capable of inducing moderate to good enantioselectivity at the stereogenic silicon center. Despite significant advances, the reaction still suffers from some limitations to date: (1) SCBs have not been shown to react with unactivated internal alkynes such as diarylacetylenes in an intermolecular manner; (2) expensive Pd- or Rh-based catalysts have been employed in all existing systems; (3) the steering of the reaction pathway exclusively toward chemoselective and enantioselective formation of allyl vinylsilanes has not yet been realized. Because Ni is smaller and more nucleophilic than Pd and Rh, nickel-based catalysts are generally more active than the corresponding complexes of palladium and rhodium at activating internal alkynes^31–35^ and cleaving the C−Si bond on SCBs^36–39^. At the same time, β-H elimination is often more facile at Ni-center in comparison to Pd or Rh-centers. We questioned whether Ni(0) catalyst is capable of facilitating the reaction of SCBs with unactivated internal alkynes and simultaneously controlling the pathway selectivity in combination of proper ligands to selective formation of either the reductive elimination or β-H elimination product (Fig. 1b), thus further broadening the utility of this process in modular assembly of diverse silicon-containing scaffolds such as an array of vicinal-diaryl substituted silacyclohexenes and allyl-tethered vinylsilanes, which are difficult to be synthesized by the other methods. It is worthy to note that vicinal-diaryl structures with/without chiral centers are a common scaffold in numerous marketed drugs as shown in Fig. 1c. Considering the importance of “C/Si switch” for the development of new materials, drugs and pesticides^11–18^, introduction of silyl moieties into 1,2-diaryl structures is of great significance and highly desired.

We herein disclose Ni-catalyzed ligand-controlled chemodivergent reactions of SCBs with alkynes. In comparison with the previous Pd or Rh catalytic systems, our Ni-catalytic system features: (1) complementary substrate scope; (2) ligand-controlled chemodivergence; (3) low cost. In our system, by use of a steric hindered NHC ligand to highly-efficient suppress β-H elimination, SCBs undergo ring expansion to exclusively provide silacycles. In contrast, phosphine ligands permit the β-H elimination step to afford allyl vinylsilanes. Moreover, we further demonstrate that employment of a chiral phosphine ligand is capable of the asymmetric induction of the ring-opening reaction of SCBs and internal alkynes, forming silicon-stereogenic allyl vinylsilanes in high yields and enantioselectivities. Additionally, we find that [4 + 2] annulation of benzosilacyclobutanes with internal alkynes also proceeds regioselectively through Si−C(*sp*^2^) bond cleavage to produce an array of dihydrobenzo[c]silines by choice of proper ligands.

## Results and discussion

### Condition screening

We started our investigations by using SCB **1a** and diphenyl acetylene **2a** as the model substrates under the Pd- or Rh-catalyzed methods developed for the cycloaddition of SCBs with terminal alkynes, but these conditions were found to be unreactive (Table 1, entries 1–2). To our delight, the reaction of **1a** and **2a** occurred smoothly in the presence of Ni(cod)_2_ (10 mol%) and PCy_3_ (20 mol%), yielding a mixture of silacyclohexene **3aa** and allyl vinylsilane **4aa** in a 6:61 ratio (Entry 3). A large panel of ligands were then screened (see Supplementary Table 1). Finally, we found that the β-H elimination product **4aa** was exclusively obtained in 83% yield by use of small phosphine PMe_3_ as the ligand (Entry 5). In contrast, the reaction performed with commercially available NHC ligand IMes·HCl (1,3-dimesity-4,5-dimethyl-1H-imidazol-3-ium chloride) resulted in exclusive formation of silacycle **3aa** under identical reaction conditions (Entry 8). The best results for producing **3aa** (84% isolated yield) were found in the presence of 20 mol% IPr·HCl (1,3-bis(2,6-diisopropylphenyl)imidazolium chloride) with incorporation of 20 mol% LiO^*t*^Bu at 120 °C (Entry 10).

**Table 1:** Condition optimization^a^.


Entry	[M] cat.	Ligand	3aa[%]^d^	4aa[%]^d^	Entry	[M] cat.	Ligand	3aa[%]^d^	4aa[%]^d^
1	Pd(OAc)_2_	PPh_3_	0	0	6	Ni(cod)_2_	Xantphos	6	74
2	Rh(PPh_3_)_3_Cl	–	0	0	7	Ni(cod)_2_	DPPB	0	0
3	Ni(cod)_2_	PCy_3_	6	61	8^b^	Ni(cod)_2_	IMes·HCl	72	0
4	Ni(cod)_2_	PPh_3_	6	94	9^b^	Ni(cod)_2_	IPr·HCl	80	0
5	Ni(cod)_2_	PMe_3_	trace	86 (83)	10^b,c^	Ni(cod)_2_	IPr·HCl	85 (84)	0

^a^[M] Catalyst (0.01 mmol), ligand (0.02 mmol), **1a** (0.3 mmol), and **2a** (0.1 mmol) in toluene for 24 h at 100 °C.

^b^20 mol% LiO^*t*^Bu as additive.

^c^120 °C for 24 h.

^d^Yields were determined by ^1^H NMR analysis using CH_2_Br_2_ as the internal standard, and values in parentheses indicate the isolated yield.

### Substrate scope for cycloaddition of SCBs with alkynes

With this optimized protocol, we first investigated the substrate scope and functional group tolerance of the Ni-catalyzed ring expansion of SCBs with internal alkynes (Table 2). It is pleased that the reaction of SCB **1a** and a wide variety of symmetric monosubstituted or multisubstituted diaryl alkynes afforded the desired products in good yields with nearly neglectable electronic and steric effects of substituents (**3ab**–**an**). Heteroaryl-substituted alkyne was also well tolerated (**3ao**). Importantly, this catalyst system is remarkably tolerant of various functional groups on the phenyl ring of diaryl alkynes, such as F, CF_3_, COOEt, and OMe groups. Additionally, various aliphatic internal alkynes are also well compatible with the established condition, enabling approach to the six-membered silacyclic products (**3bp**−**br**). In addition to symmetrical alkynes, we also applied this Ni-catalytic procedure to unsymmetrical internal alkynes to further broaden the applicability of this reaction. Fortunately, the reaction of 1-phenylpropyne **2s** with SCB **1b** proceeded smoothly under Ni-catalyzed standard conditions to produce the product **3bs** in 60% yield. However, only moderate regioisomeric ratio (rr) was observed (rr = 3:1). The further experiments proved that employment of 1-phenyl-1-pentyne (**2t**) as the alkyne partner would increase the regioisomeric ratio to 6:1 with 70% yield (**3bt**). These results indicated that increasing the steric bulk of the substituent may ensure a highly selective carbometalation step during the reaction, thus resulting in an improvement of regioselectivity. Then we examined the effect of increased steric difference between R^1^ and R^2^ with *i*-propyl or cyclopropyl-containing **2u** and **2v**. The cycloaddition reaction proved to be less efficient (35% yield for **3bu** and 45% yield for **3bv**), but the regioselectivity was significantly improved (rr > 20:1 for both **3bu** and **3bv**). Next, we examined the reactivity of TMS-protected alkynes **2w** and **2x**. Under our reaction conditions, the cycloaddition of TMS-protected arylacetylenes **2w** and **2x** with SCB **1b** led exclusively to the corresponding silacycles **3bw** and **3bx** in moderate yields (63% yield for **3bw** and 55% yield for **3bx**) and complete regioselectivity (rr > 20:1 for both **3bw** and **3bx**). However, terminal alkynes, such as phenylacetylene or 1-hexyne, which worked well with SCBs in previous Rh-catalyzed conditions, failed to participate in the reaction due to rapid self-oligomerization of the alkynes under the Ni-catalyzed condition. Then, the scope of SCBs with diverse substituents on silicon was examined. Silicon can be either mono- or diaryl-substituted, leading to **3ba**−**ga** and **3la** in high yields. The electron-donating and electron-withdrawing groups on the *meta*- or *para*-position of the phenyl ring were well tolerated (**3da**−**fa**). A methyl group at the *ortho*-position was sterically disfavored, such that ring expansion product **3ca** was generated in only 25% yield. Switching of the substituents on silicon to ethyl, n-butyl and benzyl groups did not significantly affect the performance. Good yields were observed as well (**3ha**−**ka**).

**Table 2:**
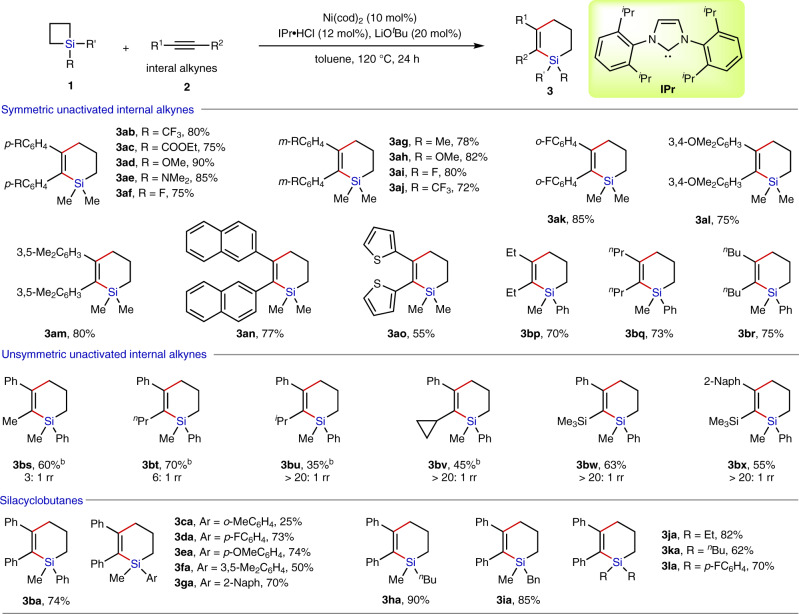
Substrate scope for cycloaddition of SCBs with alkynes^a^.

^a^Internal alkynes **2** (0.2 mmol), SCBs **1** (0.6 mmol), Ni(cod)_2_ (0.02 mmol), IPr•HCl (0.024 mmol), and LiO^*t*^Bu (0.04 mmol) in toluene (1.0 ml) at 120 °C for 24 h. Values represent isolated yields.

^b^Alkynes were slowly added to the mixture of the catalyst and SCBs at 100 °C via syringe driver over 6 h.

### Ring-opening reaction of SCBs with internal alkynes

Encouraged by the high efficiency and selectivity for the ring-opening reaction of SCBs with internal alkynes achieved that uses Ni catalysis in combination with phosphine ligand for the formation of allyl vinylsilanes **4**, we attempt to develop an asymmetric version for enantioselective construction of highly enantioenriched silicon-stereogenic allylsilanes. We performed the evaluation of a variety of different chiral P-ligands by use of SCB **1b** and diphenyl acetylene **2a** as the model substrates in the presence of Ni(cod)_2_ (10 mol%) in toluene at 100 °C for 24 h (Table 3, entries 1–20). Results showed that only TADDOL-derived phosphonite **L20** could efficiently inducing good enantioselectivity with good yield (Entry 11; 90% yield and 85:15 er). The enantioselectivity was further improved (89.5:11.5 er) without significant yield loss (88% yield) by lowering the reaction temperature to 60 °C (Table 3, entry 21). However, the yield of **4ba** decreased significantly (40% yield) while further lowering the temperature to 40 °C (Table 3, entry 22). Further investigations proved that the replacement of phenyl group on silicon of SCB **1b** by *o*-tolyl group led to a significant enhancement of the enantioselectivity (Entry 23, 95.5:4.5 er for **4ca**). In addition, further improvement in enantioselectivity and yield was seen by use of 2-MeTHF as the solvent, generating product **4ca** with a 92% yield and 96:4 er (Entry 24).

**Table 3:** Condition optimization for asymmetric ring-opening reaction of SCBs with alkyne 2a^a^.


Entry	L*	Yield (%)^b^	er^c^	Entry	L*	Yield (%)^b^	er^c^	Entry	L*	Yield (%)^b^	er^c^
1	**L1-9**	n.r.	–	9	**L18**	22	78:22	17	**L26**	39	80:20
2	**L10**	70	60:40	10	**L19**	18	60:40	18	**L27**	<5	86:14
3	**L11**	55	50:50	11	**L20**	90	85:15	19	**L28**	18	87.5:13.5
4	**L12**	37	72:28	12	**L21**	75	82:18	20	**L29**	80	88:12
5	**L13**	40	80:20	13	**L22**	80	71:19	21^d^	**L20**	88	89.5:11.5
6	**L14**	n.r.	–	14	**L23**	65	81:19	22^e^	**L20**	40	90:10
7	**L15**	10	78:22	15	**L24**	<5	93.5:6.5	23^d,f^	**L20**	90	95.5:4.5
8	**L16-17**	37	50:50	16	**L25**	n.r.	–	24^d,f,g^	**L20**	92	96:4


^a^Reactions were performed by use of Ni(cod)_2_ (10 mol%), chiral ligand (L*; 10–20 mol%), **1b** (0.2 mmol) and **2a** (0.1 mmol) in toluene (0.1 M) at 100 °C for 24 h.

^b^Isolated yield is given.

^c^The enantiomeric ratio (er) was determined by chiral HPLC on commercial columns.

^d^60 °C for 24 h.

^e^40 °C for 24 h.

^f^SCB **1c** was employed as the substrate.

^g^2-MeTHF was used as the solvent.

Under the optimized conditions for the asymmetric version to access enantioenriched silicon-stereogenic allylsilanes **4**, the substrate scope was then examined (Table 4). We first explored various diarylalkynes **2** in the ring-opening reaction with 1-methyl-1-(*o*-tolyl)siletane **1c**. The results indicate that diarylalkynes bearing electron-neutral, electron-deficient, and electron-rich substituents on different positions of the phenyl ring were compatible with the current reaction, enabling access to silicon stereogenic allyl vinylsilanes in 40 to 92% yield and 91:9 er to 96:4 er (**4ca**−**co**). Symmetrical aliphatic alkyne reacted with SCB **1q** to furnish the desired product **4cp** in 85% yield with moderate enantiomeric ratio (74:26 er for **4cp**). In addition, unsymmetrical internal alkynes such as 1-phenylpropyne were inert. Then, a number of SCBs bearing diverse substituents on silicon were tested using **2a** as the alkyne partner under the optimized condition. SCBs **1d-e** bearing different substitutents at the *ortho*-position of the phenyl group on silicon reacted smoothly with **2a** to give the products **4da** and **4ea** with good enantioselectivities (92:8 er for **4da** & 91:9 er for **4ea**). The low yield of product **4da** is attributed to the higher steric hindrance. The effect of substituents at *meta*- and *para*-positions and the π-conjugation of the phenyl ring on silicon were also illustrated by products **4fa**−**ia**. Replacement of the methyl group on silicon of SCBs by other alkyl groups such as ethyl, *i*-propyl, and cyclopropyl did not significantly affect the reaction efficiency and enantioselectivity, offering an array of chiral silicon-stereogenic allyl vinylsilanes (**4ja**–**oa**) in a 35–85% yield and 90:10 er to 92.5:7.5 er. Notably, as the products **4** are liquid, a chiroptical method was employed to determine the absolute configuration of the product **4cb**^40,41^. The computational electronic circular dichroism (ECD) spectra for **4cb** calculated by time-dependent density functional theory (DFT) is well-matched with the experimental ECD spectra (see Supplementary Figs. 1–3). Thus **4cb** was assigned to be *S*-configured.

**Table 4:**
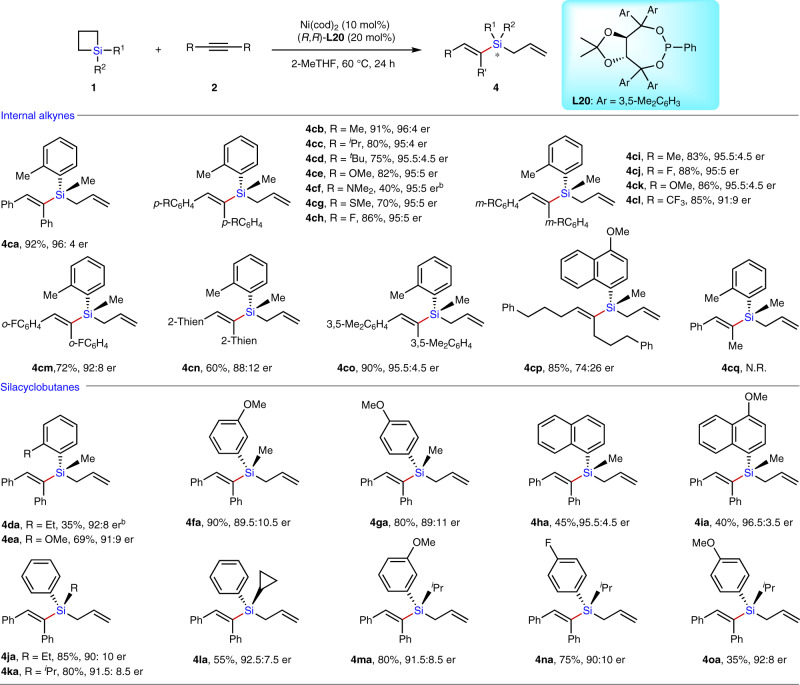
Substrate scope for enantioselective synthesis of silicon-stereogenic allyl vinylsilanes 4^a^.

^a^Internal alkynes **2** (0.2 mmol), SCBs **1** (0.4 mmol), Ni(cod)_2_ (0.02 mmol), **L20** (0.04 mmol) in 2-MeTHF at 60 °C for 24 h. Values represent isolated yields. The er value was determined by chiral HPLC.

^b^**L23** (0.04 mmol) as ligand at 80 °C for 24 h.

### Cycloaddition of benzosilacyclobutenes with internal alkynes

**Fig. 2:**
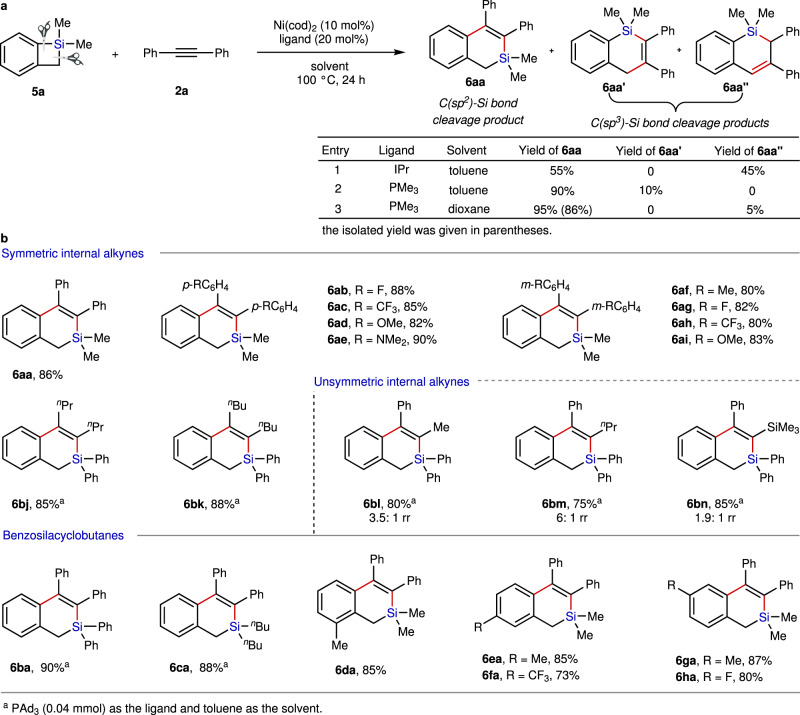
Development of Ni-catalyzed cycloaddition of benzosilacyclobutenes with internal alkynes. **a** Condition optimization for the reaction of benzosilacyclobutene **5a** with internal alkyne **2a**. **b** Substrate scope for the Ni-catalyzed cycloaddition of benzosilacyclobutenes with internal alkynes under the optimized condition. Reaction condition: alkynes **2** (0.2 mmol) and benzosilacyclobutenes **5** (0.3 mmol, 1.5 equiv.), Ni(cod)_2_ (5.8 mg, 0.02 mmol), PMe_3_ (40 μl, 1.0 M in THF, 0.04 mmol) and 1,4-dioxane (1.0 ml) at 100 °C for 24 h.

Following the success in Ni-catalyzed ligand-controlled divergent reaction of SCBs with internal alkynes, we proceeded to perform the reaction of benzosilacyclobutene **5a** with diphenylacetylene **2a** to try to further extend the practical application of this Ni-catalytic system in silacycle synthesis even the reaction of benzosilacyclobutenes with unactivated internal alkynes has also never been presented (Fig. 2a). Unfortunately, the reaction afforded a mixture of two components, **6aa** and **6aaʹʹ**, in a 55:45 ratio with full conversion under the optimized ring expansion reaction conditions due to the competitive Si−C(*sp*^3^) bond and Si−C(*sp*^2^) bond cleavage of **5a**. The formation of **6aaʹʹ** is likely attributed to the competitive migratory insertion of alkyne into the Ni–C bond, followed by an alkene isomerization and sequential reductive elimination^42,43^. To improve the regioselectivity in Si−C bond cleavage of benzosilacyclobutene **5a**, we further screened a large set of reaction parameters, such as ligands, solvents and temperatures (Supplementary Table 2). Finally, we found that employing PMe_3_ as the ligand and 1,4-dixoane as the solvent resulted in a strong preference for Si−C(*sp*^2^) bond cleavage to afford **6aa** in 86% yield. We next examined the substrate scope for the cycloaddition of benzosilacyclobutenes **5** with internal alkynes **2** to approach diverse dihydrobenzo[c]silines **6** under the modified conditions (Fig. 2, lower panel). Various diaryl alkynes bearing substituents at the *meta-* or *para*-position of the phenyl ring proceeded smoothly to afford the desired silacycles in satisfactory yields (**6ab**−**ai**). Again, our method could also be applied in dialkyl alkynes without any drop in yields (**6bj**−**bk**). Moderate regioselectivities of 1.9:1 to 6:1 were observed for the unsymmetrical internal alkynes (**6bl**−**bn**). Furthermore, we studied the benzosilacyclobutenes **5** for this reaction. To our delight, benzosilacyclobutenes containing different groups on silicon could be utilized to react with **1a** by employing PAd_3_ as the ligand, providing cyclic products **6bj**−**bm** and **6ba**−**ca** in good yields and selectivities. Finally, we proved that the high yield and selectivity was unaffected by installation of diverse substituents at different positions of the phenyl ring on benzosilacyclobutenes (**6da**−**ha**).

### Synthetic utility

**Fig. 3:**
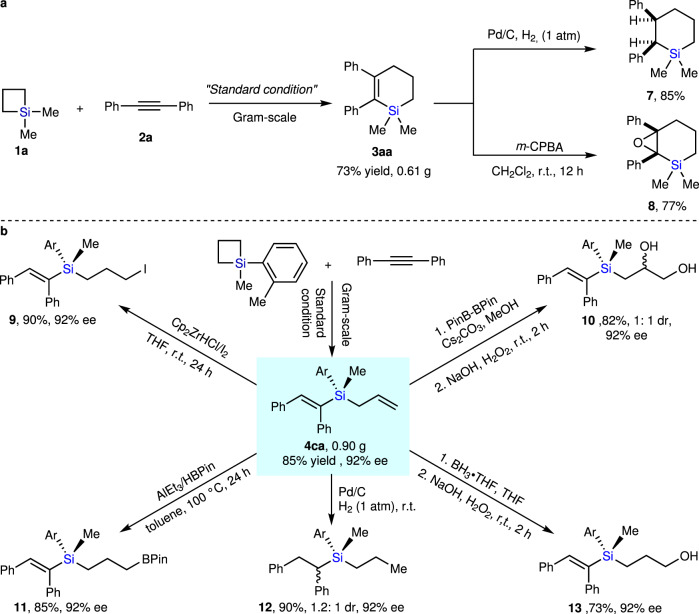
Scale up experiments and synthetic utility of our products. **a** Gram-scale synthesis and synthetic utility of **3aa**. **b** Gram-scale synthesis and synthetic utility of enantioenriched silicon-stereogenic vinylsilanes **4ca**. The details of the experiments have been provided in Supplementary Information (SI).

To demonstrate the practicality of our method, we first scaled up the Ni-catalyzed cycloaddition reaction of SCB **1a** with **2a** as well as the enantioselective ring opening reaction of SCB **1c** with **2a** to obtain 0.6 g of **3aa** in 73% yield and ~1 g of **4ca** in 85% yield with 92% ee, respectively (Fig. 3a, b). Furthermore, we demonstrated the synthetic usefulness of the 6-membered silacycle **3aa** and the enantioenriched silicon-stereogenic allylsilane **4ca** by carrying out several additional downstream transformations. The C=C bond of **3aa** can be easily reduced by hydrogen in the presence of Pd/C catalyst to deliver compound **7** in 85% yield. Moreover, **3aa** could also undergo the epoxidation reaction at room temprature in the presence of *m*-CPBA oxidant to afford epoxide **8** in 77% yield. Additionally, the product **4ca** was treated with Schwartz’s reagent, followed by molecular iodine to deliver the corresponding primary iodide **9** in 90% yield with retention of the enantioselectivity. We further demonstrated that product **4ca** can be converted to vicinal diol **10** with a 1:1 dr through a diboration/oxidation sequence. Hydroboration of the terminal C=C bond on **4ca** also proceeded smoothly to yield borylated vinylsilane **11** in 85% yield in the presence of AlEt_3_. The **4ca** could also smoothly undergo hydrogenation as well as hydroboration-oxidation to yield and product **12** in 90% yield with 92% ee and 1.2:1 dr and enantioenriched silicon-stereogenic hydroxyl-functionalized vinylsilane **13** with 73% yield and 92% ee.

### Mechanistic studies

**Fig. 4:**
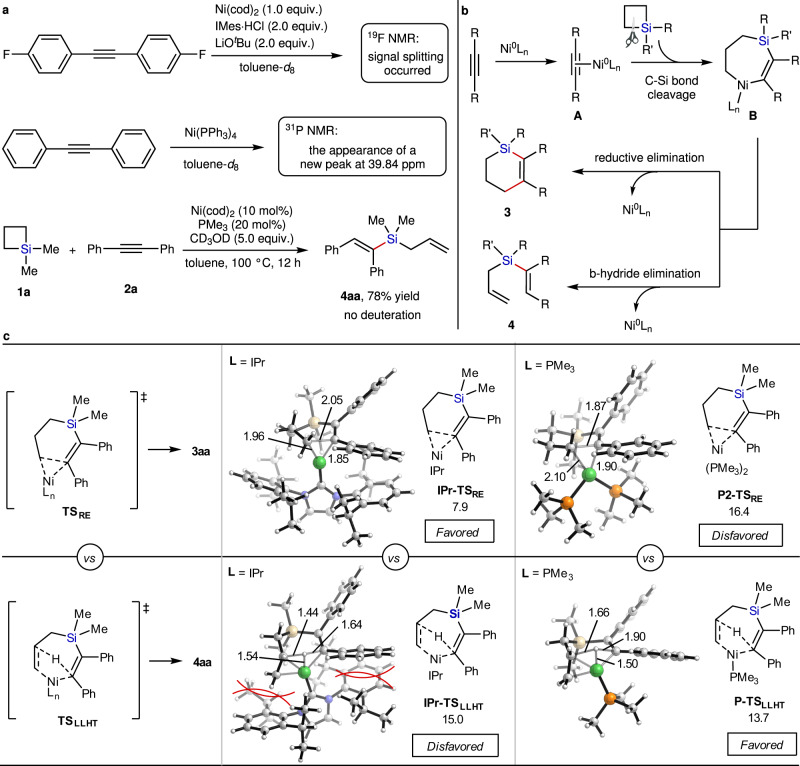
Mechanistic studies for this Ni-catalyzed divergent reactions of SCBs with alkynes. **a** Control experiments**. b** Plausible mechanism. **c** DFT investigation about mechanistic origin of the ligand-controlled structure divergence in the Ni-catalyzed reaction of SCB **1a** with **2a**. Free energies are shown in kcal/mol, distances in Å. Ni-silacycloheptene species was used as energy zero.

To probe the reaction mechanism, several mechanistically insightful experiments were carried out (Fig. 4a). When we mixed 1,2-bis(3-fluorophenyl)ethyne with 1.0 equiv. of Ni(cod)_2_ in the presence of 2.0 equiv. IMes·HCl and LiO^*t*^Bu in toluene-*d*8 for 1 h at 60 °C, signal splitting of the F atom occurred (see Supplementary Fig. 4). The addition of diphenyl acetylene to Ni(PPh_3_)_4_ resulted in the appearance of a new ^31^P NMR peak at *δ* = 39.84 ppm (see Supplementary Fig. 5). These results supported the existence of a nickel(0)-alkyne complex. In addition, there was no deuterium incorporation of **4aa** in the Ni-catalyzed ring opening reaction of **1a** and **2a** in the presence of 5.0 equiv of CD_3_OD. It strongly implys that the ring opening reaction under the optimized condition most likely involved an intramolecular hydrogen shift process. Following our experimental observation and the previous studies, we proposed a plausible mechanism to rationalize the ligand-controlled structure divergence in the Ni-catalyzed reaction of SCBs with unactivated internal alkynes (Fig. 4b). It begins with coordination of Ni^0^ to internal alkynes, thereby forming nickel(0)-alkyne complex **A**, which is followed by coordination-assisted oxidative cleavage of Si−C(*sp*^3^) on SCBs, thus producing Ni-silacycloheptene **B**. Ni-silacycloheptene **B** could smoothly undergo either reductive elimination to afford cyclic products **3** or β-hydride elimination to deliver allyl vinylsilanes **4**. To unveil the origins of the ligand-controlled structure divergence in the Ni-catalyzed reaction of SCBs with unactivated internal alkynes, computational studies for the reaction of **1a** and **2a** were carried out by DFT^44,45^. As shown in Fig. 4c and Supplementary Fig. 6, the computed profiles disclosed that the reductive elimination of the intermediate **B** leads to the ring expansion product **3aa** via a three-membered ring transition state **TS**_**RE**_ and the β-H elimination from the intermediate **B** to form the ring opening product **4aa** undergoes a ligand-to-ligand H transfer (LLHT) process via transition state **TS**_**LLHT**_. In a good agreement with experiments, the computed transition states clearly predict that IPr and PMe_3_ as the ligands favor the generation of **3aa** and **4aa** respectively with significant kinetic differentiation (7.1 kcal/mol for **IPr-TS**_**RE**_ vs. **IPr-TS**_**LLHT**_ and 2.7 kcal/mol for **P2-TS**_**RE**_ vs. **P-TS**_**LLHT**_)^46^. Importantly, it is implicated by computations that a major difference between the competing TSs lies in the steric environment around the Ni center. The extra ligations of a hydride group and a partially formed C=C unit in **TS**_**LLHT**_ render the first coordination sphere more crowded than that in **TS**_**RE**_. The latter only involves a relatively small three-membered-ring Ni–C–C array which is largely compatible with even the larger IPr ligand. These observations led us to question whether a steric control of reactivity is operative.

**Fig. 5:**
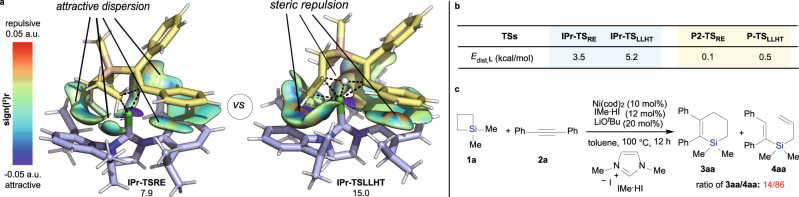
Ligand steric effects on divergent cycloaddition. **a** Visual analysis of non-covalent interactions^47^. **b** Ligand distortion energies. **c** The reaction of SCB **1a** with **2a** by use of a much less sterically hindered NHC ligand. Free energies are shown in kcal/mol, distances in Å.

To clarify our hypothesis on the steric control of reactivity, a visual analysis of non-covalent interactions for transition state **IPr-TS**_**RE**_ and **IPr-TS**_**LLHT**_ was performed. It reveals that **IPr-TS**_**LLHT**_ contains appreciable ligand-substrate steric repulsions while the competing **IPr-TS**_**RE**_ mainly shows attractive dispersions (Fig. 5a). Evaluation of ligand distortion energies also suggests higher ligand deformations in the more congested LLHT pathway if using IPr as the ligand (Fig. 5b; **E**_dist, L_ for **IPr-TS**_**LLHT**_ = 5.2 kcal/mol, **E**_dist, L_ for **IPr-TS**_**RE**_ = 3.5 kcal/mol). By contrast, the repulsions can be circumvented by use of a small PMe_3_ ligand, as reflected by the almost ignorable ligand distortion energies (≤0.5 kcal/mol in both **P2-TS**_**RE**_ and **P-TS**_**LLHT**_) and the occurrence of no repulsive atomic distances. We postulate that the LLHT pathway is plausibly intrinsically preferred against reductive elimination to form the ring opening product in this Ni-catalyzed reaction of SCBs with alkynes, which becomes a reality when small PMe_3_ ligand was used. However, the use of large sterically hindered IPr ligand in the reaction would lead to significantly destabilizing distortions of **TS**_**LLHT**_. It will steer the reaction pathway toward reductive elimination via a less crowded **TS**_**RE**_ to afford the cycloaddition product. To provide experimental support for our standpoint, the reaction of SCB **1a** with **2a** was carried out in the presence of a much less sterically hindered NHC precursor 1,3-dimethyl-3-imidazolium iodide (IMe·HI; Fig. 5c). The reaction unsurprisingly in turn favors LLHT pathway to obtain a high ratio between the ring opening product **4aa** and ring expansion product **3aa** (86:14). Notably, we have also carried out the computational studies to investigate the reaction pathway of the Ni-catalyzed cycloaddition of benzosilacyclobutenes **5** with internal alkynes **2** (see Supplementary Figs. 7 and 8). Those results are consistent with the experimental observations.

In summary, we have disclosed nickel-catalyzed reaction of (benzo)silacyclobutanes with alkynes. The choice of different ligands dictates whether the pathway involves cycloaddition or ring-opening. Moverover, by using a TADDOL-derived chiral phosphonite ligand, we achieved the highly enantioselective ring-opening reaction of SCBs with alkynes, thereby constituting an efficient and general route for asymmetric synthesis of silicon-stereogenic allyl vinylsilanes. The origins of the switchable selectivities were elucidated by DFT calculation. The results indicate that the two pathways share a Ni-silacycloheptene intermediate. IPr ligand promotes direct reductive elimination via a three-membered ring transition state from this intermediate to afford an array of (benzo)silacyclohexenes. Alternatively, small PMe_3_ ligand enables ring-opening via a LLHT process from Ni-silacycloheptene to generate diverse allyl vinylsilanes.

## Methods

### General procedure for Ni-catalyzed cycloaddition of silacyclobutanes with internal alkynes

In a glovebox, an oven-dried screw-capped 8 ml glass vial equipped with a magnetic stir bar was charged with Ni(cod)_2_ (5.8 mg, 0.02 mmol), IPr·HCl (10.2 mg, 0.024 mmol), LiO^*t*^Bu (3.2 mg, 0.04 mmol) and toluene (1.0 ml). The mixture was stirred under ambient temperature for 15 min. Then SCB **1** (0.6 mmol, 3.0 equiv.) and alkyne **2** (0.2 mmol) were added to the reaction mixture. The vial was sealed with a PTFE cap and removed from the glove box. The reaction was allowed to stir at 120 °C for 24 h. After being cooled to room temperature, the solvent was removed under vacuum and the residue was purified by flash chromatography to afford the corresponding products.

### General procedure for Ni-catalyzed asymmetric ring-opening of silacyclobutanes with internal alkynes

In a nitrogen-filled glove-box, to a 8 ml vial charged with Ni(cod)_2_ (5.8 mg, 0.02 mmol) and (*R*,*R*)-**L20** (27.0 mg, 0.04 mmol) was added 1.0 ml 2-MeTHF, and the mixture was stirred at room temperature for 1 h. Then, alkynes **2** (0.2 mmol) and SCBs **1** (0.4 mmol) were added.. The vial was then sealed with a PTFE cap, removed from the glove box. The mixture was continuously stirred at 60 °C for 24 h. After the reaction completed, the solvent was removed by rotary evaporation, and the residue was subjected to silica gel column chromatography to give the corresponding products.

### General procedure for Ni-catalyzed cycloaddition of benzosilacyclobutenes with internal alkynes

In a nitrogen-filled glove box, to an oven-dried 8 ml screw-cap vial equipped with a magnetic stir bar was added Ni(cod)_2_ (5.8 mg, 0.02 mmol), PMe_3_ (40 μl, 1.0 M in THF, 0.04 mmol) and 1,4-dioxane (1.0 ml). The mixture was stirred for 15 min at rt, at which time alkynes **2** (0.2 mmol) and benzosilacyclobutenes **5** (0.3 mmol, 1.5 equiv.) were added to the resulting mixture in this order. The vial was sealed with a PTFE cap, removed from the glove box and the reaction was stirred at 100 °C for 24 h. After being cooled to room temperature, the solvent was removed under vacuum and the residue was purified on C18(ODS) column (5 μm, 21.2 × 250 mm) with acetonitrile by preparative RP-HPLC with an Bonna-Agela CHEETAH HP series.

## Supplementary information

Supplementary Information

## Data Availability

The authors declare that the data supporting the findings of this study are available within the article and its Supplementary Information Files, and also are available from the corresponding author on request.
