# Supplementary material for: Nickel(0)-catalyzed divergent reactions of silacyclobutanes with internal alkynes
Source: Nat Commun. 2022 Jun 13;13:3392. doi: 10.1038/s41467-022-31006-y (PMC9192776; doi:10.1038/s41467-022-31006-y)
Supplement: Supplementary file 1 — Supplementary Information [file 41467_2022_31006_MOESM1_ESM.pdf]

Supplementary Information

Nickel(0)-Catalyzed Divergent Reactions of Silacyclobutanes with Internal Alkynes

Xi-Chao Wang, Bo Li, Cheng-Wei Ju, and Dongbing Zhao*

State Key Laboratory and Institute of Elemento-Organic Chemistry, Haihe Laboratory of Sustainable Chemical Transformations, College of Chemistry, Nankai University, Tianjin, 300071, China

*E-mail: dongbing.chem@nankai.edu.cn

Table of Contents

1. Supplementary Notes 2

2. Supplementary Methods 2

Preparation of TADDOL-derived phosphite ligands 2

Nickel-catalyzed cycloaddition of SCBs with internal alkynes 4

Optimization of the reaction conditions 4

General procedure 4

Characterization of products 3 5

Nickel-catalyzed asymmetric ring-opening reaction of SCBs with internal alkynes 17

General procedure 17

Characterization of silicon stereogenic allyl vinylsilanes 4 17

Nickel-catalyzed cycloaddition of benzosilacyclobutanes with alkynes 33

Optimization of the reaction conditions 33

General procedure 34

Characterization of products 6 34

Scale-up experiment and derivatization of 3aa and 4ca 42

Gram-scale experiment for 3aa 42

Derivatization of 3aa 42

Gram-scale experiment for 4ca 43

Derivatization of 4ca 44

3. Supplementary Discussion 49

Determination of absolute configuration by ECD experiments 49

Mechanistic experiments 52

4. Supplementary Figures 57

NMR spectra 57

HPLC charts of chiral products 180

Absolute energies and coordinates of optimized structures 209

5. Supplementary References 266

1. Supplementary Notes

Nuclear magnetic resonance (NMR) spectra were recorded on Bruker AV 400 spectrometer at 400MHz (**^{1}H NMR**), 100MHz (**^{13}C NMR**) and 376MHz (**^{19}F NMR**) using $CDCl_3$ as solvent. Proton and carbon chemical shifts are reported relative to the solvent used as an internal reference ($CDCl_3$: δ_H = 7.26 ppm, δ_C = 77.16 ppm). Data are presented in the following space: chemical shift, multiplicity, coupling constant in hertz (Hz), and signal area integration in natural numbers. GC-MS spectra was obtained using electron ionization (Thermo Scientific Trace 300/GC-System and ISQ/QD). High resolution mass spectra (**HRMS**) were obtained on an IonSpec FT-ICR mass spectrometer in ESI mode, or a Q Exactive GC-Orbitrap mass spectrometer in EI mode. Unless mentioned otherwise, all manipulations were performed in a nitrogen-filled glove box or using standard Schlenk techniques. Analytical thin layer chromatography (TLC) was performed on silica gel 60 F_{254} glass plates. TLC plates were visualized by exposure to short wave ultraviolet light (254 nm, 365 nm) and/or iodine. All the solvents used for reactions were distilled under nitrogen after drying over an appropriate drying agent. Commercially available chemicals were obtained from Admas, Alfa Aesar, J&K, Sigma-Aldrich, Energy Chemical and TCI and used as received unless otherwise stated. Silacyclobutanes[1], benzosilacyclobutanes[2] and alkynes[3] were synthesized according to the reported literature.

2. Supplementary Methods

Preparation of TADDOL-derived phosphite ligands

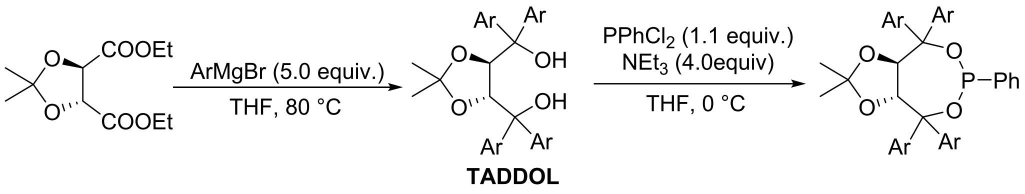

Following literature reports[4], a typical procedure for preparation of TADDOL-derived phosphite ligands is described as follow:

<u>Step 1</u>: To an oven dried 250 mL two-neck flask equipped with a stir bar and a reflux condenser was added magnesium powder (53 mmol, 5.3 equiv.), one grain of I_2 and THF (10 mL). Then a solution of arylbromide (50 mmol, 5.0 equiv.) in THF (50 mL) was added dropwise to initiate the reaction. When the color of the solution disappeared, the remaining solution was added slowly, keeping the internal temperature below the boiling point of the solvent. After complete addition, the reaction mixture was allowed to reflux for 1 h. After cooled down to room temperature, a solution of isopropylidene-protected diethyl tartrate (10 mmol, 1.0 equiv.) in THF (40 mL) was added, after complete addition, the reaction mixture was refluxed for 2 h and then cooled down to room temperature. Saturated ammonium chloride solution (40 mL), hydrochloric acid (1.0 M, 10 mL) and water (50 mL) were added carefully. The resulting biphasic mixture was extracted with ether (4 × 50 mL). The combined organic extracts were washed with brine (30 mL) and dried over Na_2SO_4. All volatiles were removed under reduced pressure. The crude mixture was purified by silica gel chromatography (petroleum ether/EtOAc) to obtain the corresponding **TADDOL**.

Step 2: To an oven dried 100 mL Schlenk tube equipped with a stir bar was added **TADDOL** (1.0 mmol), 4 Å MS (100 mg), triethylamine (0.6 mL, 4.0 mmol, 4 equiv.) and THF (10 mL) under N_2. The mixture was cooled to 0 ℃. Then dichlorophenylphosphine (1.1 mmol, 1.1 equiv.) was added *via* syringe. The reaction mixture was allowed to warm to room temperature and stirred for 2 h. The mixture was then diluted with Et_2O, filtered through celite, and concentrated under reduced pressure. Purification by flash column chromatography (petroleum ether /dichloromethane/ triethylamine = 10: 1: 0.3) afforded the phosphonite ligand.

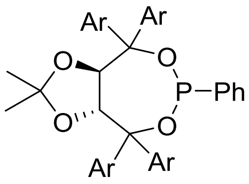

Ar = 3,5-$Me_2C_6H_3$

(*R*,*R*)-**L20**: The title compound was obtained in 55% yield as white solid. The characterization data was concordant with that previously reported in the literature[5]. **^{1}H NMR (400 MHz, CDCl$_3$)**: δ 7.62 (m, 2H), 7.28 (m, 5H), 7.04 (s, 1H), 6.99 (d, *J* = 1.5 Hz, 2H), 6.84 (d, *J* = 12.0 Hz, 4H), 6.71 (d, *J* = 9.1 Hz, 2H), 6.65 (s, 2H), 6.60 (s, 1H), 5.33 (dd, *J* = 8.5, 5.0 Hz, 1H), 4.55 (d, *J* = 8.5 Hz, 1H), 2.08 (s, 6H), 2.06 (s, 12H), 2.04 (s, 6H), 1.36 (s, 3H), -0.00 (s, 3H). **^{13}C NMR (101 MHz, CDCl$_3$)**: δ 146.9, 146.3 (d, $J_{C,P}$ = 3.5 Hz), 141.8 (d, $J_{C,P}$ = 10.0 Hz), 141.5, 137.3, 137.1, 136.9, 136.4, 130.5, 130.2, 129.9, 129.3, 129.1, 128.9, 128.5 (d, $J_{C,P}$ = 6.6 Hz), 127.4, 126.5 (d, $J_{C,P}$ = 3.2 Hz), 125.5 (d, $J_{C,P}$ = 6.4 Hz), 111.4 , 84.3 (d, $J_{C,P}$ = 4.3 Hz), 83.4 (d, $J_{C,P}$ = 7.5 Hz), 82.8 (d, $J_{C,P}$ = 25.0 Hz), 82.3 (d, $J_{C,P}$ = 4.0 Hz), 46.5, 28.1, 25.2, 21.8, 21.8, 21.7, 11.8. **^{31}P NMR (162 MHz, CDCl$_3$)**: δ 155.7.

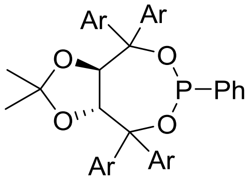

Ar = 3,5-$Ph_2C_6H_3$

(*R*,*R*)-**L23**: The title compound was obtained in 65% yield as white solid. The characterization data was concordant with that previously reported in the literature[6]. **^{1}H NMR (400 MHz, CDCl$_3$)**: δ 8.25 (d, *J* = 1.8 Hz, 2H), 8.01 (q, *J* = 4.0, 3.0 Hz, 4H), 7.90 – 7.79 (m, 5H), 7.76 – 7.66 (m, 7H), 7.64 – 7.52 (m, 15H), 7.49 – 7.35 (m, 18H), 7.31 (m, 6H), 6.26 (dd, *J* = 8.6, 4.7 Hz, 1H), 5.16 (d, *J* = 8.6 Hz, 1H), 1.60 (s, 3H), 0.35 (s, 3H). **^{13}C NMR (101 MHz, CDCl$_3$)**: δ 147.9, 146.9 (d, $J_{C,P}$ = 3.5 Hz), 143.2 (d, $J_{C,P}$ = 7.2 Hz), 142.2, 141.8 (d, $J_{C,P}$ = 5.9 Hz), 141.5, 141.4, 141.3 (d, $J_{C,P}$ = 6.2 Hz), 140.9, 131.2, 130.2, 129.9, 129.0, 128.9 (d, $J_{C,P}$ = 6.5 Hz), 128.8 (d, $J_{C,P}$ = 7.7 Hz), 127.6 (d, $J_{C,P}$ = 4.4 Hz), 127.5, 127.3, 126.7, 126.1 (d, $J_{C,P}$ = 5.9 Hz), 125.7 (d, $J_{C,P}$ = 4.4 Hz), 125.5, 112.2, 84.5 (d, $J_{C,P}$ = 2.8 Hz), 83.9 (d, $J_{C,P}$ = 6.9 Hz), 83.2 (d, $J_{C,P}$ = 5.1 Hz), 83.1 (d, $J_{C,P}$ = 4.4 Hz), 82.8 (d, $J_{C,P}$ = 4.6 Hz), 28.2, 27.1 (d, $J_{C,P}$ = 2.5 Hz), 25.1. **^{19}P NMR (162 MHz, CDCl$_3$)**: δ 156.7.

Nickel-catalyzed cycloaddition of SCBs with internal alkynes

Optimization of the reaction conditions

Supplementary Table 1. Optimization of the reaction conditions[a]

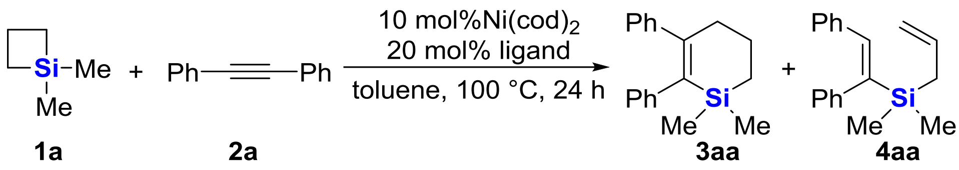

Entry	Ligand	3aa (%)[b]	4aa (%)[b]
1	PMe_3	trace	86
2	P^tBu_3	9	42
3	PCy_3	6	61
4	PPh_3	6	94
5	P(*o*-Tol)$_3$	2	14
6	P(*p*-OMe-C_6H_4)$_3$	6	88
7	P(*p*-F-C_6H_4)$_3$	4	92
8	P(2-Furyl)$_3$	4	86
9	*rac*-BINAP	0	0
10	XantPhos	6	74
11	DPPB	0	0
12	DPPF	2	0
13[c]	IMes·HCl	72	0
14[c]	IPr·HCl	80	0
15[c,d]	IPr·HCl	85	0

[a] Reaction conditions: diphenylacetylene **2a** (0.1 mmol), SCB **1a** (0.30 mmol, 3.0 equiv), Ni(cod)$_2$ (0.01 mmol), ligand (0.02 mmol) in toluene (1.0 mL) at 100 ℃ for 24 h under N_2. [b] Yields were determined by ^{1}H NMR analysis using CH_2Br_2 as the internal standard. [c] 20 mol% LiO^{t}Bu as additive. [d] 120 ℃.

General procedure

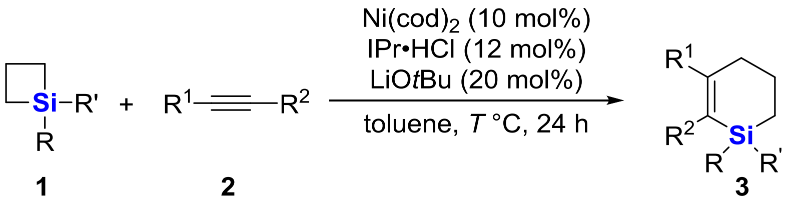

Procedure A: In a nitrogen-filled glove-box, an oven-dried 8 mL glass vial equipped with a magnetic stir bar was charged with Ni(cod)$_2$ (5.8 mg, 0.02 mmol), IPr·HCl (10.2 mg, 0.024 mmol), LiO^{t}Bu (3.2 mg, 0.04 mmol) and toluene (1.0 mL). The mixture was allowed to stir under ambient temperature for 15 min. Then silacyclobutane **1** (0.6 mmol, 3.0 equiv.) and alkyne **2** (0.2 mmol) were added sequentially. The vial was sealed with a PTFE cap, removed from the glove box and stirred at 120 ℃ for 24 h. After being cooled to

room temperature, the solvent was removed under vacuum and the residue was subjected to silica gel column chromatography to give the corresponding products.

Procedure B: In a nitrogen-filled glove-box, an oven-dried 15 mL Schlenk tube equipped with a magnetic stir bar was charged with $Ni(cod)_2$ (5.8 mg, 0.02 mmol), IPr·HCl (10.2 mg, 0.024 mmol), LiO^tBu (3.2 mg, 0.04 mmol) and toluene (1.0 mL). The mixture was allowed to stir under ambient temperature for 15 min. Then silacyclobutane **1** (0.2 mmol) was added. The tube was sealed and removed from the glove box. Alkyne **2** (0.4 mmol, 2.0 equiv.) was dissolved in 2 mL toluene under nitrogen and was added to the mixture over 6 h via syringe drive at 100 ℃. The resulting mixture was allowed to stir for 12 h at 100 ℃. Once complete, the mixture was cooled to room temperature, the solvent was removed under vacuum and the residue was purified on C18(ODS) column (5μm, 21.2x250 mm) with acetonitrile by preparative RP-HPLC with an Bonna-Agela CHEETAH HP series.

Characterization of products 3

1,1-Dimethyl-2,3-diphenyl-1-sila-2-cyclohexene (3aa):

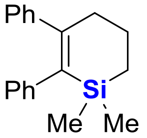

Following procedure **A**, the reaction was carried out with silacyclobutane **1a** (0.6 mmol, 3.0 equiv.), diphenylacetylene **2a** (0.2 mmol), $Ni(cod)_2$ (5.8 mg, 0.02 mmol), IPr·HCl (10.2 mg, 0.024 mmol), LiO^tBu (3.2 mg, 0.04 mmol) and toluene (1.0 mL) at 120 ℃ for 24 h. The title compound was obtained in 84% yield (46.8 mg) as colorless oil;

^{1}H NMR (400 MHz, $CDCl_3$): δ 7.10 – 7.04 (m, 4H), 7.03 – 6.94 (m, 4H), 6.83 – 6.77 (m, 2H), 2.60 – 2.53 (m, 2H), 2.05 – 1.98 (m, 2H), 0.89 – 0.83 (m, 2H), 0.08 (s, 6H).

^{13}C NMR (101 MHz, $CDCl_3$): δ 150.4, 143.0, 141.8, 140.6, 138.9, 136.3, 131.0, 130.6, 130.1, 128.0, 127.7, 127.5, 127.1, 126.2, 125.1, 124.7, 77.3, 77.2, 77.0, 76.7, 21.3, -3.7.

HRMS (EI): Calculated for $C_{19}H_{22}Si$ $[M]^+$: 278.1491. Found: 278.1486.

1,1-Dimethyl-2,3-bis(4-(trifluoromethyl)phenyl)-1-sila-2-cyclohexene (3ab):

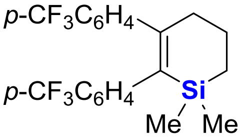

Following procedure **A**, the reaction was carried out with silacyclobutane **1a** (0.6 mmol, 3.0 equiv.), alkyne **2b** (0.2 mmol), $Ni(cod)_2$ (5.8 mg, 0.02 mmol), IPr·HCl (10.2 mg, 0.024 mmol), LiO^tBu (3.2 mg, 0.04 mmol) and toluene (1.0 mL) at 120 ℃ for 24 h. The title compound was obtained in 70% yield (58.0 mg) as colorless oil;

^{1}H NMR (400 MHz, $CDCl_3$): δ 7.37 – 7.30 (m, 4H), 7.06 – 7.00 (m, 2H), 6.91 – 6.86 (m, 2H), 2.58 – 2.50 (m, 2H), 2.07 – 1.97 (m, 2H), 0.91 – 0.85 (m, 2H), 0.07 (s, 6H).

^{13}C NMR (101 MHz, $CDCl_3$): δ 152.9, 147.8 (q, J = 1.3 Hz), 146.9 (q, J = 1.2 Hz), 138.9, 128.9, 128.5, 128.0 (d, J = 32.3 Hz), 127.2 (q, J = 32.3 Hz), 125.0 – 124.6 (m), 124.5 (q, J = 271.8 Hz), 124.2 (q, J = 271.9 Hz), 37.0, 21.7, 11.9, -2.3.

^{19}F NMR (376 MHz, $CDCl_3$): δ -62.2, -62.5.

HRMS (EI): Calculated for $C_{21}H_{20}F_6Si$ $[M]^+$: 414.1238. Found: 414.1233.

Diethyl 4,4'-(1,1-dimethyl-1,4,5,6-tetrahydrosiline-2,3-diyl)dibenzoate (3ac):

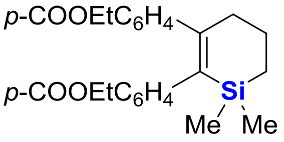

Following procedure **A**, the reaction was carried out with silacyclobutane **1a** (0.6 mmol, 3.0 equiv.), alkyne **2c** (0.2 mmol), $Ni(cod)_2$ (5.8 mg, 0.02 mmol), IPr·HCl (10.2 mg, 0.024 mmol), LiO^tBu (3.2 mg, 0.04 mmol) and toluene (1.0 mL) at 120 ℃ for 24 h. The title compound was obtained in 75% yield (63.3 mg) as colorless oil;

^{1}H NMR (400 MHz, $CDCl_3$): δ 7.80 – 7.66 (m, 4H), 7.02 – 6.96 (m, 2H), 6.87 – 6.80 (m, 2H), 4.30 (qd, *J* = 7.1, 3.3 Hz, 4H), 2.58 – 2.50 (m, 2H), 2.06 – 1.96 (m, 2H), 1.34 (td, *J* = 7.1, 3.7 Hz, 6H), 0.91 – 0.82 (m, 2H), 0.06 (s, 6H).

^{13}C NMR (101 MHz, $CDCl_3$): δ 166.8, 166.6, 153.1, 149.1, 148.4, 139.1, 129.2, 129.1, 128.7, 128.2, 128.2, 127.1, 60.9, 60.8, 36.8, 21.8, 14.5, 14.4, 12.0, -2.3.

HRMS (ESI): Calculated for [$C_{25}H_{30}O_4Si$ + Na^+]: 445.1806. Found: 445.1806.

2,3-Bis(4-methoxyphenyl)-1,1-dimethyl-1-sila-2-cyclohexene (3ad):

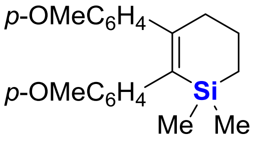

Following procedure **A**, the reaction was carried out with silacyclobutane **1a** (0.6 mmol, 3.0 equiv.), alkyne **2d** (0.2 mmol), $Ni(cod)_2$ (5.8 mg, 0.02 mmol), IPr·HCl (10.2 mg, 0.024 mmol), LiO^tBu (3.2 mg, 0.04 mmol) and toluene (1.0 mL) at 120 ℃ for 24 h. The title compound was obtained in 90% yield (60.9 mg) as colorless oil;

^{1}H NMR (400 MHz, $CDCl_3$): δ 6.92 – 6.86 (m, 2H), 6.74 – 6.68 (m, 2H), 6.68 – 6.56 (m, 4H), 3.72 (s, 3H), 3.70 (s, 3H), 2.57 – 2.49 (m, 2H), 2.03 – 1.93 (m, 2H), 0.87 – 0.78 (m, 2H), 0.06 (s, 6H).

^{13}C NMR (101 MHz, $CDCl_3$): δ 157.5, 156.8, 152.8, 137.4, 136.9, 136.0, 129.9, 129.6, 113.2, 112.9, 55.1, 37.3, 22.1, 12.5, -2.0.

HRMS (EI): Calculated for $C_{21}H_{26}O_2Si$ $[M]^+$: 338.1702. Found: 338.1696.

4,4'-(1,1-Dimethyl-1,4,5,6-tetrahydrosiline-2,3-diyl)bis(N,N-dimethylaniline) (3ae):

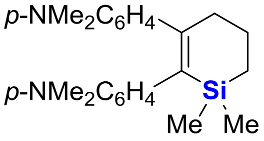

Following procedure **A**, the reaction was carried out with silacyclobutane **1a** (0.6 mmol, 3.0 equiv.), alkyne **2e** (0.2 mmol), $Ni(cod)_2$ (5.8 mg, 0.02 mmol), IPr·HCl (10.2 mg, 0.024 mmol), LiO^tBu (3.2 mg, 0.04 mmol) and toluene (1.0 mL) at 120 ℃ for 24 h. The title compound was obtained in 85% yield (62.0 mg) as colorless oil;

^{1}H NMR (400 MHz, $CDCl_3$): δ 6.92 – 6.83 (m, 2H), 6.74 – 6.66 (m, 2H), 6.58 – 6.51 (m, 2H), 6.50 – 6.42 (m, 2H), 2.86 (s, 6H), 2.85 (s, 6H), 2.54 (dd, *J* = 6.5, 5.0 Hz, 2H), 2.01 – 1.92 (m, 2H), 0.85 – 0.76 (m, 2H), 0.05 (s, 6H).

^{13}C NMR (101 MHz, $CDCl_3$): δ 152.3, 148.4, 147.6, 135.8, 133.6, 129.8, 129.5, 112.7, 111.8, 41.0, 40.7, 37.3, 22.3, 12.8, -1.7.

HRMS (ESI): Calculated for [$C_{25}H_{28}Osi$ + H^+]: 365.2408. Found: 365.2411.

2,3-Bis(4-fluorophenyl)-1,1-Dimethyl-1-sila-2-cyclohexene (3af):

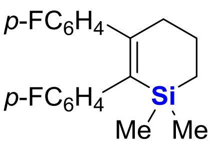

Following procedure **A**, the reaction was carried out with silacyclobutane **1a** (0.6 mmol, 3.0 equiv.), alkyne **2f** (0.2 mmol), Ni(cod)$_2$ (5.8 mg, 0.02 mmol), IPr·HCl (10.2 mg, 0.024 mmol), LiO^{t}Bu (3.2 mg, 0.04 mmol) and toluene (1.0 mL) at 120 ℃ for 24 h. The title compound was obtained in 75% yield (47.1 mg) as colorless oil;

^{1}H NMR (400 MHz, CDCl$_3$): δ 6.90 – 6.84 (m, 2H), 6.80 – 6.73 (m, 4H), 6.73 – 6.68 (m, 2H), 2.54 – 2.47 (m, 2H), 2.03 – 1.93 (m, 2H), 0.86 – 0.82 (m, 2H), 0.05 (s, 6H).

^{13}C NMR (101 MHz, CDCl$_3$): δ 161.0 (d, *J* = 244.9 Hz), 160.6 (d, *J* = 243.1 Hz), 153.0 (d, *J* = 0.8 Hz), 140.4 (d, *J* = 3.3 Hz), 139.0 (d, *J* = 3.7 Hz), 137.7, 130.2 (d, *J* = 7.5 Hz), 129.9 (d, *J* = 8.0 Hz), 114.7 (d, *J* = 17.3 Hz), 114.5 (d, *J* = 17.3 Hz), 37.2, 21.9, 12.1, -2.2.

^{19}F NMR (376 MHz, CDCl$_3$): δ -116.5, -118.8.

HRMS (EI): Calculated for $C_{19}H_{20}F_2Si$ [M]$^+$: 314.1302. Found: 314.1296.

1,1-Dimethyl-2,3-di-*m*-tolyl-1-sila-2-cyclohexene (3ag):

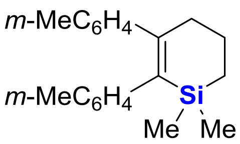

Following procedure **A**, the reaction was carried out with silacyclobutane **2a** (0.6 mmol, 3.0 equiv.), alkyne **2g** (0.2 mmol), Ni(cod)$_2$ (5.8 mg, 0.02 mmol), IPr·HCl (10.2 mg, 0.024 mmol), LiO^{t}Bu (3.2 mg, 0.04 mmol) and toluene (1.0 mL) at 120 ℃ for 24 h. The title compound was obtained in 78% yield (47.8 mg) as colorless oil;

^{1}H NMR (400 MHz, CDCl$_3$): δ 6.97 (td, *J* = 7.7, 3.6 Hz, 2H), 6.87 – 6.72 (m, 4H), 6.67 (td, *J* = 1.7, 0.9 Hz, 1H), 6.61 (dd, *J* = 7.6, 1.5 Hz, 1H), 2.59 – 2.54 (m, 2H), 2.21 (s, 3H), 2.20 (s, 3H), 2.02 (m, 2H), 0.90 – 0.83 (m, 2H), 0.10 (s, 6H).

^{13}C NMR (101 MHz, CDCl$_3$): δ 153.2, 144.8, 143.3, 137.8, 129.7, 129.0, 127.4, 127.3, 126.5, 126.1, 125.5, 125.3, 37.2, 22.0, 21.6, 21.5, 12.4, -2.1.

HRMS (EI): Calculated for $C_{21}H_{26}Si$ [M]$^+$: 306.1804. Found: 306.1798.

2,3-Bis(3-methoxyphenyl)-1,1-dimethyl-1-sila-2-cyclohexene (3ah):

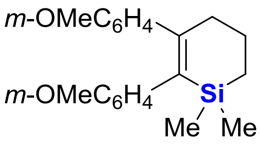

Following procedure **A**, the reaction was carried out with silacyclobutane **1a** (0.6 mmol, 3.0 equiv.), alkyne **2h** (0.2 mmol), Ni(cod)$_2$ (5.8 mg, 0.02 mmol), IPr·HCl (10.2 mg, 0.024 mmol), LiO^{t}Bu (3.2 mg, 0.04 mmol) and toluene (1.0 mL) at 120 ℃ for 24 h. The title compound was obtained in 82% yield (54.2 mg) as colorless oil.

^{1}H NMR (400 MHz, CDCl$_3$): δ 6.63 – 6.52 (m, 3H), 6.50 (dd, *J* = 2.6, 1.5 Hz, 1H), 6.45 – 6.41 (m, 1H), 6.36 (dd, *J* = 2.7, 1.5 Hz, 1H), 3.64 (s, 3H), 3.59 (s, 3H), 2.59 – 2.51 (m, 2H), 2.05 – 1.96 (m, 2H), 0.89 – 0.81 (m, 2H), 0.09 (s, 6H).

^{13}C NMR (101 MHz, CDCl$_3$): δ 159.0, 158.8, 153.1, 146.1, 144.9, 138.0, 128.5, 128.5, 121.5, 120.7, 114.4, 113.8, 112.1, 110.4, 55.2, 55.1, 37.0, 21.9, 12.3, -2.0.

HRMS (EI): Calculated for $C_{21}H_{26}O_2Si$ [M]$^+$: 338.1702. Found: 338.1696.

2,3-Bis(3-fluorophenyl)-1,1-dimethyl-1-sila-2-cyclohexene (3ai):

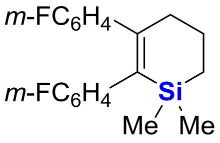

Following procedure **A**, the reaction was carried out with silacyclobutane **1a** (0.6 mmol, 3.0 equiv.), alkyne **2i** (0.2 mmol), $Ni(cod)_2$ (5.8 mg, 0.02 mmol), IPr·HCl (10.2 mg, 0.024 mmol), LiO^tBu (3.2 mg, 0.04 mmol) and toluene (1.0 mL) at 120 ℃ for 24 h. The title compound was obtained in 80% yield (50.3 mg) as colorless oil;

^{1}H NMR (400 MHz, $CDCl_3$): δ 7.08 – 6.99 (m, 2H), 6.75 – 6.63 (m, 4H), 6.59 – 6.53 (m, 1H), 6.53 – 6.47 (m, 1H), 2.55 – 2.48 (m, 2H), 2.04 – 1.94 (m, 2H), 0.89 – 0.81 (m, 2H), 0.07 (s, 6H).

^{13}C NMR (101 MHz, $CDCl_3$): δ 162.6 (d, J = 245.3 Hz), 162.4 (d, J = 245.2 Hz), 146.6 (d, J = 7.1 Hz), 145.5 (d, J = 7.5 Hz), 152.8 (d, J = 1.7 Hz), 146.6 (d, J = 7.1 Hz), 145.5 (d, J = 7.5 Hz), 138.2 (d, J = 1.4 Hz), 129.2 (d, J = 6.0 Hz), 129.1 (d, J = 5.9 Hz), 124.6 (d, J = 2.7 Hz), 124.0 (d, J = 2.8 Hz), 115.4 (d, J = 20.6 Hz), 115.1 (d, J = 21.3 Hz), 113.0 (d, J = 20.9 Hz), 111.8 (d, J = 21.2 Hz), 36.9, 21.8, 12.0, -2.3.

^{19}F NMR (376 MHz, $CDCl_3$): δ -114.2, -114.3.

HRMS (EI): Calculated for $C_{19}H_{20}F_2Si$ $[M]^+$: 314.1302. Found: 314.1296.

1,1-Dimethyl-2,3-bis(3-(trifluoromethyl)phenyl)-1-sila-2-cyclohexene (3aj):

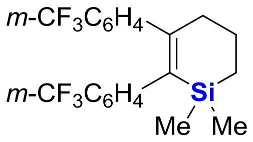

Following procedure **A**, the reaction was carried out with silacyclobutane **1a** (0.6 mmol, 3.0 equiv.), alkyne **2j** (0.2 mmol), $Ni(cod)_2$ (5.8 mg, 0.02 mmol), IPr·HCl (10.2 mg, 0.024 mmol), LiO^tBu (3.2 mg, 0.04 mmol) and toluene (1.0 mL) at 120 ℃ for 24 h. The title compound was obtained in 72% yield (59.6 mg) as colorless oil;

^{1}H NMR (400 MHz, $CDCl_3$): δ 7.20 – 7.12 (m, 2H), 7.12 – 7.03 (m, 3H), 6.99 (dt, J = 7.7, 1.5 Hz, 1H), 6.94 (d, J = 1.8 Hz, 1H), 6.83 (dt, J = 7.6, 1.5 Hz, 1H), 2.52 – 2.45 (m, 2H), 1.94 (m, 2H), 0.83 – 0.76 (m, 2H), -0.00 (s, 6H).

^{13}C NMR (101 MHz, $CDCl_3$): δ 153.4, 144.7, 143.7, 138.9, 132.0 (d, J = 1.4 Hz), 131.4 (q, J = 1.5 Hz), 130.2 (q, J = 32.0 Hz), 130.0 (q, J = 32.1 Hz), 128.3, 128.2, 125.6 (q, J = 3.8 Hz), 125.2 (q, J = 3.9 Hz), 124.2 (d, J = 272.3 Hz), 124.1 (d, J = 272.3 Hz), 123.0 (q, J = 3.9 Hz), 121.8 (q, J = 3.9 Hz), 36.8, 21.7, 11.9, -2.4.

^{19}F NMR (376 MHz, $CDCl_3$): δ -62.9.

HRMS (EI): Calculated for $C_{21}H_{20}F_6Si$ $[M]^+$: 414.1238. Found: 414.1232.

2,3-Bis(2-fluorophenyl)-1,1-dimethyl-1-sila-2-cyclohexene (3ak):

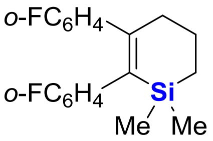

Following procedure **A**, the reaction was carried out with silacyclobutane **1a** (0.6 mmol, 3.0 equiv.), alkyne **2k** (0.2 mmol), $Ni(cod)_2$ (5.8 mg, 0.02 mmol), IPr·HCl (10.2 mg, 0.024 mmol), LiO^tBu (3.2 mg, 0.04 mmol) and toluene (1.0 mL) at 120 ℃ for 24 h. The title compound was obtained in 85% yield (51.6 mg) as colorless oil;

^{1}H NMR (400 MHz, $CDCl_3$): δ 7.04 – 6.89 (m, 3H), 6.89 – 6.75 (m, 5H), 2.50 (t, J = 5.8 Hz, 2H), 2.03 (m, 2H), 0.94 – 0.85 (m, 2H), 0.08 (d, J = 0.9 Hz, 6H).

^{13}C NMR (101 MHz, $CDCl_3$): δ 158.9 (d, J = 242.4 Hz), 158.8 (d, J = 244.3 Hz), 150.8, 134.5, 132.1 (d, J = 17.5 Hz), 130.4 (d, J = 4.1 Hz), 130.2 (d, J = 17.9 Hz), 129.8 (d, J = 4.6 Hz), 128.1 (d, J = 8.1 Hz), 127.0 (d, J

= 7.7 Hz), 123.4 (d, *J* = 3.6 Hz), 123.3 (d, *J* = 3.6 Hz), 115.1 (d, *J* = 17.8 Hz), 114.9 (d, *J* = 18.2 Hz), 36.3, 36.3, 21.7, 11.9, -2.5, -2.6.

^{19}F NMR (376 MHz, $CDCl_3$): δ -114.31, -115.83.

HRMS (EI): Calculated for $C_{19}H_{20}F_2Si$ $[M]^+$: 314.1302. Found: 314.1295.

2,3-Bis(3,4-dimethoxyphenyl)-1,1-dimethyl -1-sila-2-cyclohexene (3al):

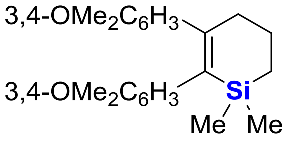

Following procedure **A**, the reaction was carried out with silacyclobutane **1a** (0.6 mmol, 3.0 equiv.), alkyne **2l** (0.2 mmol), $Ni(cod)_2$ (5.8 mg, 0.02 mmol), IPr·HCl (10.2 mg, 0.024 mmol), LiO^{t}Bu (3.2 mg, 0.04 mmol) and toluene (1.0 mL) at 120 °C for 24 h. The title compound was obtained in 75% yield (59.8 mg) as colorless oil;

^{1}H NMR (400 MHz, $CDCl_3$): δ 6.61 (t, *J* = 8.0 Hz, 3H), 6.45 (s, 1H), 6.37 (dd, *J* = 8.3, 1.9 Hz, 1H), 6.31 (s, 1H), 3.78 (s, 6H), 3.64 (s, 3H), 3.57 (s, 3H), 2.59 – 2.52 (m, 2H), 2.02 – 1.93 (m, 2H), 0.87 – 0.79 (m, 2H), 0.08 (s, 6H).

^{13}C NMR (101 MHz, $CDCl_3$): δ 152.8, 148.4, 147.8, 147.1, 146.3, 137.6, 137.1, 136.5, 120.8, 120.3, 112.8, 112.7, 110.8, 110.4, 55.8, 55.8, 55.7, 37.0, 22.0, 12.4, -1.9.

HRMS (EI): Calculated for $C_{23}H_{30}O_4Si$ $[M]^+$: 398.1913. Found: 398.1906.

2,3-Bis(3,5-dimethylphenyl)-1,1-dimethyl-1-sila-2-cyclohexene (3am):

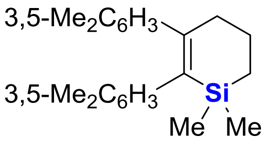

Following procedure **A**, the reaction was carried out with silacyclobutane **1a** (0.6 mmol, 3.0 equiv.), alkyne **2m** (0.2 mmol), $Ni(cod)_2$ (5.8 mg, 0.02 mmol), IPr·HCl (10.2 mg, 0.024 mmol), LiO^{t}Bu (3.2 mg, 0.04 mmol) and toluene (1.0 mL) at 120 °C for 24 h. The title compound was obtained in 80% yield (53.5 mg) as colorless oil;

^{1}H NMR (400 MHz, $CDCl_3$): δ 6.64 (s, 1H), 6.60 (s, 3H), 6.44 (s, 2H), 2.55 – 2.50 (m, 2H), 2.16 (s, 6H), 2.14 (s, 6H), 2.02 – 1.95 (m, 2H), 0.86 – 0.80 (m, 2H), 0.07 (s, 6H).

^{13}C NMR (101 MHz, $CDCl_3$): δ 153.1, 144.8, 143.1, 137.6, 136.5, 136.4, 127.3, 126.8, 126.2, 126.1, 37.4, 22.1, 21.4, 21.3, 12.4, -2.1.

HRMS (EI): Calculated for $C_{23}H_{30}Si$ $[M]^+$: 334.2117. Found: 334.2110.

1,1-Dimethyl-2,3-di(naphthalen-2-yl)-1-sila-2-cyclohexene (3an):

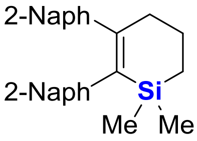

Following procedure **A,** the reaction was carried out with silacyclobutane **1a** (0.6 mmol, 3.0 equiv.), alkyne **2n** (0.2 mmol), $Ni(cod)_2$ (5.8 mg, 0.02 mmol), IPr·HCl (10.2 mg, 0.024 mmol), LiO^{t}Bu (3.2 mg, 0.04 mmol) and toluene (1.0 mL) at 120 °C for 24 h. The title compound was obtained in 77% yield (58.2 mg) as colorless oil;

^{1}H NMR (400 MHz, $CDCl_3$): δ 7.70 – 7.61 (m, 4H), 7.59 – 7.54 (m, 1H), 7.49 (dd, *J* = 16.1, 8.5 Hz, 2H), 7.41 (d, *J* = 1.6 Hz, 1H), 7.39 – 7.29 (m, 4H), 7.15 (dd, *J* = 8.5, 1.7 Hz, 1H), 7.00 (dd, *J* = 8.4, 1.7 Hz, 1H), 2.78 – 2.70 (m, 2H), 2.18 – 2.07 (m, 2H), 1.02 – 0.91 (m, 2H), 0.16 (s, 6H).

^{13}C NMR (101 MHz, $CDCl_3$): δ 153.6, 141.2, 138.6, 133.1, 131.9, 131.3, 128.5, 127.9, 127.7, 127.6, 127.5, 127.4, 127.2, 127.0, 126.7, 126.6, 125.7, 125.5, 125.4, 124.8, 37.5, 22.1, 12.4, -1.9.

HRMS (EI): Calculated for $C_{27}H_{26}Si$ $[M]^+$: 378.1804. Found: 378.1796.

1,1-Dimethyl-2,3-di(thiophen-2-yl)-1-sila-2-cyclohexene (3ao):

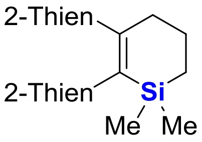

Following procedure **A**, the reaction was carried out with silacyclobutane **1a** (0.6 mmol, 3.0 equiv.), alkyne **2o** (0.2 mmol), $Ni(cod)_2$ (5.8 mg, 0.02 mmol), IPr·HCl (10.2 mg, 0.024 mmol), LiO^tBu (3.2 mg, 0.04 mmol) and toluene (1.0 mL) at 120 ℃ for 24 h. The title compound was obtained in 55% yield (32.0 mg) as colorless oil;

^{1}H NMR (400 MHz, $CDCl_3$): δ 7.09 (dd, J = 5.1, 1.2 Hz, 1H), 7.01 (dd, J = 5.0, 1.3 Hz, 1H), 6.85 (dd, J = 5.1, 3.5 Hz, 1H), 6.75 (dd, J = 3.7, 1.3 Hz, 1H), 6.72 (dd, J = 5.0, 3.7 Hz, 1H), 6.51 (dd, J = 3.4, 1.2 Hz, 1H), 2.62 – 2.58 (m, 2H), 1.92 – 1.84 (m, 2H), 0.75 – 0.69 (m, 2H), -0.00 (s, 6H).

^{13}C NMR (101 MHz, $CDCl_3$): δ 147.7, 145.1, 144.4, 131.3, 127.2, 126.4, 126.1, 126.0, 125.2, 124.9, 36.8, 21.7, 11.9, -2.5.

HRMS (EI): Calculated for $C_{15}H_{18}S_2Si$ $[M]^+$: 290.0619. Found: 290.0613.

2,3-Diethyl-1-methyl-1-phenyl-1-sila-2-cyclohexene (3bp):

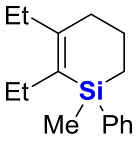

Following procedure **A**, the reaction was carried out with silacyclobutane **1b** (0.6 mmol, 3.0 equiv.), alkyne **2p** (0.2 mmol), $Ni(cod)_2$ (5.8 mg, 0.02 mmol), IPr·HCl (10.2 mg, 0.024 mmol), LiO^tBu (3.2 mg, 0.04 mmol) and toluene (1.0 mL) at 120 ℃ for 24 h. The title compound was obtained in 70% yield (34.2 mg) as colorless oil;

^{1}H NMR (400 MHz, $CDCl_3$): δ 7.56 – 7.50 (m, 2H), 7.37 – 7.31 (m, 3H), 2.24 – 2.06 (m, 5H), 2.04 – 1.93 (m, 1H), 1.87 – 1.75 (m, 2H), 1.05 (t, J = 7.6 Hz, 3H), 0.92 – 0.73 (m, 5H), 0.40 (s, 3H).

^{13}C NMR (101 MHz, $CDCl_3$): δ 156.0, 139.6, 134.5, 129.1, 128.8, 127.7, 34.4, 27.5, 23.3, 22.0, 15.5, 13.5, 12.8, -3.8.

HRMS (EI): Calculated for $C_{16}H_{24}Si$ $[M]^+$: 244.1647. Found: 244.1642.

2,3-Dipropyl-1-methyl-1-phenyl-1-sila-2-cyclohexene (3bq):

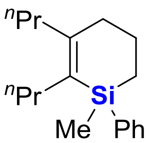

Following procedure **A**, the reaction was carried out with silacyclobutane **1b** (0.6 mmol, 3.0 equiv.), alkyne **2q** (0.2 mmol), $Ni(cod)_2$ (5.8 mg, 0.02 mmol), IPr·HCl (10.2 mg, 0.024 mmol), LiO^tBu (3.2 mg, 0.04 mmol) and toluene (1.0 mL) at 120 ℃ for 24 h. The title compound was obtained in 73% yield (39.8 mg) as colorless oil;

^{1}H NMR (400 MHz, $CDCl_3$): δ7.57 – 7.49 (m, 2H), 7.37 – 7.30 (m, 3H), 2.25 – 2.01 (m, 5H), 1.97 – 1.87 (m, 1H), 1.85 – 1.73 (m, 2H), 1.54 – 1.42 (m, 2H), 1.31 – 1.08 (m, 2H), 0.95 (t, J = 7.3 Hz, 3H), 0.90 – 0.71 (m, 5H), 0.39 (s, 3H).

^{13}C NMR (101 MHz, $CDCl_3$): δ 154.7, 139.7, 134.4, 128.8, 128.5, 127.7, 36.9, 35.0, 33.2, 24.1, 22.0, 14.8, 14.5, 12.9, -3.7.

HRMS (EI): Calculated for $C_{18}H_{28}Si$ $[M]^+$: 272.1960. Found: 272.1955.

2,3-Dibutyl-1-methyl-1-phenyl-1-sila-2-cyclohexene (3br):

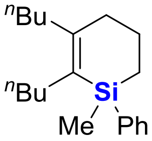

Following procedure **A**, the reaction was carried out with silacyclobutane **1b** (0.6 mmol, 3.0 equiv.), alkyne **2r** (0.2 mmol), $Ni(cod)_2$ (5.8 mg, 0.02 mmol), IPr·HCl (10.2 mg, 0.024 mmol), LiO^tBu (3.2 mg, 0.04 mmol) and toluene (1.0 mL) at 120 ℃ for 24 h. The title compound was obtained in 75% yield (45.0 mg) as colorless oil;

^{1}H NMR (400 MHz, $CDCl_3$): δ 7.56 – 7.48 (m, 2H), 7.34 (dd, J = 4.3, 2.1 Hz, 3H), 2.26 – 2.02 (m, 5H), 1.97 – 1.88 (m, 1H), 1.85 – 1.73 (m, 2H), 1.48 – 1.30 (m, 4H), 1.26 – 1.14 (m, 3H), 1.14 – 1.03 (m, 1H), 0.95 (t, J = 7.1 Hz, 3H), 0.91 – 0.71 (m, 5H), 0.39 (s, 3H).

^{13}C NMR (101 MHz, $CDCl_3$): δ 154.8, 139.7, 134.4, 128.8, 128.2, 127.7, 35.0, 34.6, 33.1, 31.1, 30.5, 23.4, 23.3, 22.0, 14.3, 14.1, 12.8, -3.7.

HRMS (EI): Calculated for $C_{20}H_{32}Si$ $[M]^+$: 300.2273. Found: 300.2268.

1,2-Dimethyl-1,3-diphenyl-1-sila-2-cyclohexene (3bs):

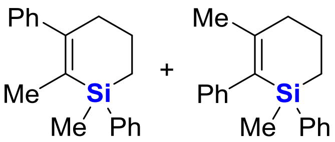

3bs' **3bs''**

Following procedure **B**, the reaction was carried out with silacyclobutane **1b** (0.2 mmol), alkyne **2s** (0.4 mmol, 2.0 equiv.), $Ni(cod)_2$ (5.8 mg, 0.02 mmol), IPr·HCl (10.2 mg, 0.024 mmol), LiO^tBu (3.2 mg, 0.04 mmol) and toluene (1.0 mL) at 100 ℃ for 24 h. The mixture was obtained as colorless oil in 60% yield (33.4 mg) and 3: 1 *rr*.

^{1}H NMR (400 MHz, $CDCl_3$): δ 7.64 – 7.57 (m, 6H), 7.47 – 7.27 (m, 20H), 7.25 – 7.20 (m, 3H), 7.19 – 7.12 (m, 8H), 7.10 – 7.05 (m, 1H), 6.81 – 6.75 (m, 2H), 2.51 – 2.41 (**3bs'**, m, 6H), 2.36 – 2.28 (**3bs''**, m, 2H), 2.05 – 1.88 (m, 8H), 1.61 (s, 3H), 1.54 (t, J = 2.0 Hz, 11H), 1.12 – 0.96 (m, 4H), 0.94 – 0.82 (m, 5H), 0.44 (s, 9H), 0.27 (s, 3H).

^{13}C NMR (101 MHz, $CDCl_3$): δ154.2, 151.5, 145.0, 138.5, 134.6, 134.4, 128.9, 128.7, 128.2, 127.9, 127.9, 127.6, 126.4, 126.3, 125.0, 37.6, 36.7, 23.0, 22.0, 21.5, 17.4, 12.1, 11.7, 0.2, -4.1, -4.8.

HRMS (EI): Calculated for $C_{19}H_{22}Si$ $[M]^+$: 278.1491. Found: 278.1485.

1-Methyl-1,3-diphenyl-2-propyl-1-sila-2-cyclohexene (3bt):

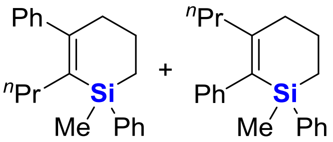

3bt' **3bt''**

Following procedure **B**, the reaction was carried out with silacyclobutane **1b** (0.2 mmol), alkyne **2s** (0.4 mmol, 2.0 equiv.), $Ni(cod)_2$ (5.8 mg, 0.02 mmol), IPr·HCl (10.2 mg, 0.024 mmol), LiO^tBu (3.2 mg, 0.04 mmol) and toluene (1.0 mL) at 100 ℃ for 24 h. The mixture was obtained as colorless oil in 70% yield (42.8 mg) and 7: 1 *rr*.

^{1}H NMR (400 MHz, $CDCl_3$): δ 7.65 – 7.57 (m, 14H), 7.45 – 7.28 (m, 41H), 7.27 – 7.18 (m, 13H), 7.16 – 7.09 (m, 16H), 7.08 – 7.02 (m, 1H), 6.77 – 6.70 (m, 2H), 2.43 (**3bt'**, t, J = 5.9 Hz, 15H), 2.33 – 2.27 (**3bt'',** m,

2H), 2.03 – 1.76 (m, 35H), 1.37 (h, J = 7.5 Hz, 3H), 1.19 – 0.81 (m, 34H), 0.75 (t, J = 7.3 Hz, 4H), 0.56 (t, J = 7.3 Hz, 22H), 0.48 (s, 22H), 0.25 (s, 3H).

^{13}C NMR (101 MHz, $CDCl_3$): δ 155.14, 145.65, 139.13, 134.53, 134.47, 131.50, 129.02, 128.87, 128.81, 128.17, 127.84, 127.72, 127.66, 127.60, 126.14, 124.85, 38.37, 38.28, 34.53, 34.06, 23.75, 21.94, 21.80, 14.51, 14.22, 12.59, 11.92, -3.53, -4.12.

HRMS (EI): Calculated for $C_{21}H_{26}Si$ $[M]^+$: 306.1804. Found: 306.1802.

2-Isopropyl-1-methyl-1,3-diphenyl-1-sila-2-cyclohexene (3bu):

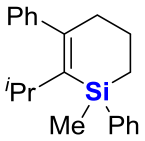

Following procedure **B**, the reaction was carried out with silacyclobutane **1b** (0.2 mmol), alkyne **2s** (0.4 mmol, 2.0 equiv.), $Ni(cod)_2$ (5.8 mg, 0.02 mmol), IPr·HCl (10.2 mg, 0.024 mmol), LiO^{t}Bu (3.2 mg, 0.04 mmol) and toluene (1.0 mL) at 100 ℃ for 24 h. The title compound was obtained in 35% yield (21.4 mg) as colorless oil;

^{1}H NMR (400 MHz, $CDCl_3$): δ 7.67 – 7.61 (m, 2H), 7.39 – 7.28 (m, 5H), 7.23 – 7.17 (m, 1H), 7.13 – 7.08 (m, 2H), 2.53 – 2.43 (m, 1H), 2.42 – 2.35 (m, 2H), 1.96 – 1.78 (m, 2H), 0.90 – 0.82 (m, 5H), 0.58 (s, 3H), 0.55 (d, J = 7.0 Hz, 3H).

^{13}C NMR (101 MHz, $CDCl_3$): δ 154.4, 146.7, 140.6, 137.4, 134.5, 128.8, 128.2, 127.7, 127.4, 126.0, 38.8, 33.3, 24.1, 23.8, 21.6, 13.9, -1.6.

HRMS (EI): Calculated for $C_{21}H_{26}Si$ $[M]^+$: 306.1804. Found: 306.1800.

2-Cyclopropyl-1-Methyl-1,3-diphenyl-1-sila-2-cyclohexene (3bv):

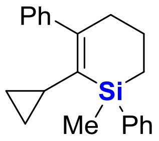

Following procedure **B**, the reaction was carried out with silacyclobutane **1b** (0.2 mmol), alkyne **2s** (0.4 mmol, 2.0 equiv.), $Ni(cod)_2$ (5.8 mg, 0.02 mmol), IPr·HCl (10.2 mg, 0.024 mmol), LiO^{t}Bu (3.2 mg, 0.04 mmol) and toluene (1.0 mL) at 100 ℃ for 24 h. The title compound was obtained in 45% yield (27.3 mg) as colorless oil;

^{1}H NMR (400 MHz, $CDCl_3$): δ 7.65 – 7.58 (m, 2H), 7.40 – 7.31 (m, 5H), 7.29 – 7.26 (m, 2H), 7.25 – 7.20 (m, 1H), 2.48 (td, J = 5.8, 1.4 Hz, 2H), 1.98 – 1.82 (m, 2H), 1.26 – 1.17 (m, 1H), 0.93 – 0.80 (m, 2H), 0.48 (s, 3H), 0.34 – 0.23 (m, 2H), 0.17 – 0.07 (m, 2H).

^{13}C NMR (101 MHz, $CDCl_3$): δ 156.6, 146.0, 139.6, 134.4, 132.0, 129.0, 128.2, 128.0, 127.8, 126.2, 38.1, 21.7, 15.4, 13.4, 7.7, 7.2, -3.0.

HRMS (EI): Calculated for $C_{21}H_{24}Si$ $[M]^+$: 304.1647. Found: 304.1647.

1-Methyl-1,3-diphenyl-2-(trimethylsilyl)-1-sila-2-cyclohexene (3bw):

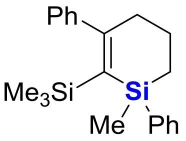

Following procedure **A**, the reaction was carried out with silacyclobutane **1b** (0.6 mmol, 3.0 equiv.), alkyne **2s** (0.2 mmol), $Ni(cod)_2$ (5.8 mg, 0.02 mmol), IPr·HCl (10.2 mg, 0.024 mmol), LiO^{t}Bu (3.2 mg, 0.04 mmol) and toluene (1.0 mL) at 120 ℃ for 24 h. The title compound was obtained in 63% yield (42.4 mg) as colorless oil;

^{1}H NMR (400 MHz, CDCl$_3$): δ 7.67 – 7.61 (m, 2H), 7.41 (dd, *J* = 4.2, 2.2 Hz, 3H), 7.38 – 7.33 (m, 2H), 7.33 – 7.29 (m, 1H), 7.22 – 7.17 (m, 2H), 2.63 – 2.47 (m, 2H), 2.00 – 1.85 (m, 2H), 0.87 – 0.77 (m, 2H), 0.58 (s, 3H), -0.38 (s, 9H).

^{13}C NMR (101 MHz, CDCl$_3$): δ 171.7, 148.87, 134.6, 134.5, 131.5, 128.9, 128.0, 127.7, 126.8, 41.4, 21.0, 13.2, 2.1, -2.4.

HRMS (EI): Calculated for $C_{21}H_{28}Si_2$ $[M]^+$: 336.1730. Found: 336.1724.

1-Methyl-3-(naphthalen-2-yl)-1-phenyl-2-(trimethylsilyl)-1-sila-2-cyclohexene (3bx):

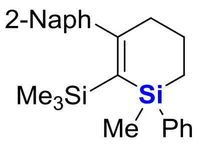

Following procedure **A**, the reaction was carried out with silacyclobutane **1b** (0.6 mmol, 3.0 equiv.), alkyne **2s** (0.2 mmol), Ni(cod)$_2$ (5.8 mg, 0.02 mmol), IPr·HCl (10.2 mg, 0.024 mmol), LiO^{t}Bu (3.2 mg, 0.04 mmol) and toluene (1.0 mL) at 120 ℃ for 24 h. The title compound was obtained in 55% yield (42.5 mg) as colorless oil;

^{1}H NMR (400 MHz, CDCl$_3$): δ 7.89 – 7.78 (m, 3H), 7.68 – 7.57 (m, 3H), 7.54 – 7.44 (m, 2H), 7.43 – 7.35 (m, 3H), 7.33 (dd, *J* = 8.4, 1.7 Hz, 1H), 2.68 – 2.49 (m, 2H), 2.03 – 1.84 (m, 2H), 0.91 – 0.73 (m, 2H), 0.57 (s, 3H), -0.43 (s, 9H).

^{13}C NMR (101 MHz, CDCl$_3$): δ 171.6, 146.2, 140.3, 134.6, 133.1, 132.4, 132.2, 128.9, 128.0, 127.9, 127.7, 127.6, 126.6, 126.3, 126.0, 125.8, 41.2, 21.1, 13.2, 2.2, -2.4.

HRMS (EI): Calculated for $C_{25}H_{30}Si_2$ $[M]^+$: 386.1886. Found: 386.1883.

1-Methyl-1,2,3-triphenyl-1-sila-2-cyclohexene (3ba):

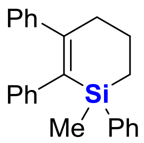

Following procedure **A**, the reaction was carried out with diphenylacetylene (0.2 mmol), silacyclobutane **1b** (0.6 mmol, 3.0 equiv.), Ni(cod)$_2$ (5.8 mg, 0.02 mmol), IPr·HCl (10.2 mg, 0.024 mmol), LiO^{t}Bu (3.2 mg, 0.04 mmol) and toluene (1.0 mL) at 120 ℃ for 24 h. The title compound was obtained in 74% yield (50.4 mg) as colorless oil;

^{1}H NMR (400 MHz, CDCl$_3$): δ 7.57 – 7.52 (m, 2H), 7.39 – 7.32 (m, 3H), 7.14 – 7.06 (m, 2H), 7.06 – 6.98 (m, 3H), 6.98 – 6.87 (m, 3H), 6.70 – 6.64 (m, 2H), 2.78 – 2.62 (m, 2H), 2.21 – 2.03 (m, 2H), 1.22 – 1.12 (m, 1H), 1.09 – 0.98 (m, 1H), 0.36 (s, 3H).

^{13}C NMR (101 MHz, CDCl$_3$): δ 155.4, 144.8, 142.9, 138.3, 135.8, 134.6, 129.4, 129.1, 128.5, 127.8, 127.6, 127.4, 126.1, 124.7, 37.3, 22.0, 12.0, -4.0.

HRMS (EI): Calculated for $C_{24}H_{24}Si$ $[M]^+$: 340.1647. Found: 340.1640.

1-Methyl-2,3-diphenyl-1-(*o*-tolyl)-1-sila-2-cyclohexene (3ca):

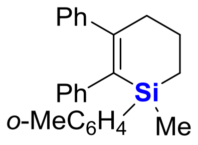

Following procedure **A**, the reaction was carried out with diphenylacetylene (0.2 mmol), silacyclobutane **1c** (0.6 mmol, 3.0 equiv.), Ni(cod)$_2$ (5.8 mg, 0.02 mmol), IPr·HCl (10.2 mg, 0.024 mmol), LiO^{t}Bu (3.2 mg, 0.04 mmol) and toluene (1.0 mL) at 120 ℃ for 24 h. The title compound was obtained in 25% yield (17.7 mg) as colorless oil;

^{1}H NMR (400 MHz, CDCl$_3$): δ 7.53 (dd, *J* = 7.4, 1.5 Hz, 1H), 7.33 – 7.27 (m, 1H), 7.21 – 7.07 (m, 4H), 7.06 – 6.97 (m, 3H), 6.92 – 6.81 (m, 3H), 6.70 – 6.58 (m, 2H), 2.80 – 2.71 (m, 1H), 2.68 – 2.58 (m, 1H), 2.56 (s, 3H), 2.21 – 2.10 (m, 1H), 2.09 – 1.95 (m, 1H), 1.25 – 1.13 (m, 1H), 1.06 – 0.97 (m, 1H), 0.38 (s, 3H).

^{13}C NMR (101 MHz, CDCl$_3$): δ 155.2, 145.0, 143.9, 143.1, 136.3, 136.2, 136.2, 130.1, 129.5, 129.2, 128.7, 127.7, 127.4, 126.2, 125.0, 124.7, 37.2, 23.0, 22.4, 12.2, -2.6.

HRMS (EI): Calculated for $C_{25}H_{26}Si$ [M]$^+$: 354.1804. Found: 354.1799.

1-(4-Fluorophenyl)-1-methyl-2,3-diphenyl-1-sila-2-cyclohexene (3da):

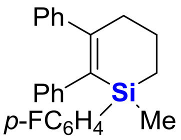

Following procedure **A**, the reaction was carried out with diphenylacetylene (0.2 mmol), silacyclobutane **1d** (0.6 mmol, 3.0 equiv.), Ni(cod)$_2$ (5.8 mg, 0.02 mmol), IPr·HCl (10.2 mg, 0.024 mmol), LiO^{t}Bu (3.2 mg, 0.04 mmol) and toluene (1.0 mL) at 120 ℃ for 24 h. The title compound was obtained in 73% yield (52.3 mg) as colorless oil;

^{1}H NMR (400 MHz, CDCl$_3$): δ 7.72 (dd, *J* = 8.2, 6.1 Hz, 2H), 7.35 – 7.12 (m, 10H), 6.92 – 6.83 (m, 2H), 3.00 – 2.84 (m, 2H), 2.42 – 2.26 (m, 2H), 1.43 – 1.32 (m, 1H), 1.32 – 1.22 (m, 1H), 0.59 (s, 3H).

^{13}C NMR (101 MHz, CDCl$_3$): δ 163.8 (d, *J* = 248.2 Hz), 155.6, 144.6, 142.8, 136.5 (d, *J* = 7.4 Hz), 135.6, 133.6 (d, *J* = 3.7 Hz), 129.3, 128.5, 127.6 (d, *J* = 11.6 Hz), 126.1, 124.8, 115.0 (d, *J* = 19.7 Hz), 37.2, 21.9, 12.0, -3.8.

^{19}F NMR (376 MHz, CDCl$_3$): δ -112.00.

HRMS (EI): Calculated for $C_{24}H_{23}Fsi$ [M]$^+$: 358.1553. Found: 358.1548.

1-(4-Methoxyphenyl)-1-methyl-2,3-diphenyl-1-sila-2-cyclohexene (3ea):

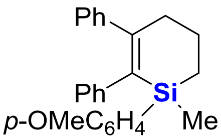

Following procedure **A**, the reaction was carried out with diphenylacetylene (0.2 mmol), silacyclobutane **1e** (0.6 mmol, 3.0 equiv.), Ni(cod)$_2$ (5.8 mg, 0.02 mmol), IPr·HCl (10.2 mg, 0.024 mmol), LiO^{t}Bu (3.2 mg, 0.04 mmol) and toluene (1.0 mL) at 120 ℃ for 24 h. The title compound was obtained in 75% yield (55.6 mg) as colorless oil;

^{1}H NMR (400 MHz, CDCl$_3$): δ 7.50 – 7.42 (m, 2H), 7.12 – 7.06 (m, 2H), 7.06 – 6.99 (m, 3H), 6.98 – 6.89 (m, 5H), 6.73 – 6.62 (m, 2H), 3.83 (s, 3H), 2.77 – 2.61 (m, 2H), 2.22 – 2.03 (m, 2H), 1.20 – 1.10 (m, 1H), 1.07 – 0.97 (m, 1H), 0.34 (s, 3H).

^{13}C NMR (101 MHz, CDCl$_3$): δ 160.5, 155.0, 144.8, 143.1, 136.2, 136.1, 129.4, 129.0, 128.5, 127.6, 127.4, 126.0, 124.6, 113.6, 55.1, 37.3, 22.0, 12.2, -3.9.

HRMS (EI): Calculated for $C_{25}H_{26}Osi$ [M]$^+$: 370.1753. Found: 370.1742.

1-(3,5-Dimethylphenyl)-1-methyl-2,3-diphenyl-1-sila-2-cyclohexene (3fa):

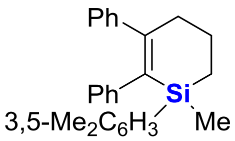

Following the general procedure, the reaction was carried out with diphenylacetylene (0.2 mmol), silacyclobutane **1f** (0.6 mmol, 3.0 equiv.), Ni(cod)$_2$ (5.8 mg, 0.02 mmol), IPr·HCl (10.2 mg, 0.024 mmol), LiO^{t}Bu (3.2 mg, 0.04 mmol) and toluene (1.0 mL) at 120 ℃ for 24 h. The title compound was obtained in 50% yield (36.9 mg) as colorless

oil;

^{1}H NMR (400 MHz, Chloroform-*d*): δ 7.14 – 7.06 (m, 4H), 7.02 (m, 4H), 6.99 – 6.87 (m, 3H), 6.73 – 6.65 (m, 2H), 2.76 – 2.61 (m, 2H), 2.31 (s, 6H), 2.19 – 2.01 (m, 2H), 1.16 (m, 1H), 1.00 (m, 1H), 0.33 (s, 3H).

^{13}C NMR (101 MHz, Chloroform-*d*): δ 155.1, 144.9, 143.1, 138.0, 137.0, 136.0, 132.4, 130.9, 129.5, 128.6, 127.6, 127.4, 126.0, 124.6, 37.3, 22.0, 21.6, 12.0, -3.8.

HRMS (EI): Calculated for $C_{26}H_{28}Si$ $[M]^+$: 368.1960. Found: 368.1949.

1-methyl-1-(naphthalen-2-yl)2,3-diphenyl-1-sila-2-cyclohexene (3ga):

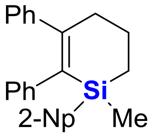

Following procedure **A**, the reaction was carried out with diphenylacetylene (0.2 mmol), silacyclobutane **1g** (0.6 mmol, 3.0 equiv.), $Ni(cod)_2$ (5.8 mg, 0.02 mmol), IPr·HCl (10.2 mg, 0.024 mmol), LiO^{t}Bu (3.2 mg, 0.04 mmol) and toluene (1.0 mL) at 120 ℃ for 24 h. The title compound was obtained in 70% yield (54.6 mg) as colorless oil;

^{1}H NMR (400 MHz, $CDCl_3$): δ 8.08 – 8.00 (m, 1H), 7.84 (d, *J* = 7.6 Hz, 4H), 7.69 – 7.61 (m, 1H), 7.57 – 7.46 (m, 2H), 7.19 – 7.02 (m, 6H), 7.00 – 6.85 (m, 3H), 6.78 – 6.65 (m, 2H), 2.92 – 2.67 (m, 2H), 2.34 – 2.08 (m, 1H), 1.33 – 1.20 (m, 1H), 1.18 – 1.05 (m, 1H), 0.47 (d, *J* = 1.7 Hz, 3H).

^{13}C NMR (101 MHz, $CDCl_3$): δ 155.6, 144.8, 142.9, 135.8, 135.8, 135.4, 133.8, 133.0, 130.9, 129.4, 128.5, 128.2, 127.8, 127.6, 127.5, 126.9, 126.4, 126.1, 125.9, 124.7, 37.4, 22.1, 12.2, -3.9.

HRMS (EI): Calculated for $C_{28}H_{26}Si$ $[M]^+$: 390.1804. Found: 390.1801.

1-Butyl-1-methyl-2,3-diphenyl-1-sila-2-cyclohexene (3ha):

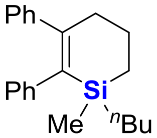

Following procedure **A**, the reaction was carried out with diphenylacetylene (0.2 mmol), silacyclobutane **1h** (0.6 mmol, 3.0 equiv.), $Ni(cod)_2$ (5.8 mg, 0.02 mmol), IPr·HCl (10.2 mg, 0.024 mmol), LiO^{t}Bu (3.2 mg, 0.04 mmol) and toluene (1.0 mL) at 120 ℃ for 24 h. The title compound was obtained in 90% yield (57.7 mg) as colorless oil;

^{1}H NMR (400 MHz, $CDCl_3$): δ 7.08 (dd, *J* = 8.1, 6.7 Hz, 4H), 7.04 – 6.94 (m, 4H), 6.83 – 6.78 (m, 2H), 2.65 – 2.51 (m, 2H), 2.10 – 1.94 (m, 2H), 1.38 – 1.19 (m, 4H), 0.99 – 0.78 (m, 5H), 0.66 – 0.48 (m, 2H), 0.10 (s, 3H).

^{13}C NMR (101 MHz, $CDCl_3$): δ 154.0, 144.9, 143.6, 137.6, 129.0, 128.4, 127.6, 127.5, 125.8, 124.6, 37.2, 26.7, 26.2, 22.2, 13.9, 10.6, -3.5.

HRMS (EI): Calculated for $C_{22}H_{28}Si$ $[M]^+$: 320.1960. Found: 320.1953.

1-Benzyl-1-methyl-2,3-diphenyl-1-sila-2-cyclohexene (3ia):

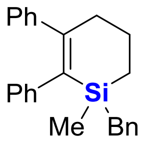

Following procedure **A**, the reaction was carried out with diphenylacetylene (0.2 mmol), silacyclobutane **1i** (0.6 mmol, 3.0 equiv.), $Ni(cod)_2$ (5.8 mg, 0.02 mmol), IPr·HCl (10.2 mg, 0.024 mmol), LiO^{t}Bu (3.2 mg, 0.04 mmol) and toluene (1.0 mL) at 120 ℃ for 24 h. The title compound was obtained in 85% yield (60.3 mg) as colorless oil;

^{1}H NMR (400 MHz, CDCl$_3$): δ 7.53 – 7.45 (m, 2H), 7.42 – 7.32 (m, 5H), 7.32 – 7.20 (m, 6H), 7.17 – 7.10 (m, 2H), 2.80 (t, *J* = 5.8 Hz, 2H), 2.52 (d, *J* = 13.6 Hz, 1H), 2.33 (d, *J* = 13.7 Hz, 1H), 2.26 – 2.09 (m, 2H), 1.23 – 1.13 (m, 1H), 0.97 (m, 1H), 0.37 (s, 3H).

^{13}C NMR (101 MHz, CDCl$_3$): δ 155.0, 144.7, 143.1, 140.0, 136.6, 129.2, 128.4, 128.3, 128.3, 127.8, 127.6, 126.0, 124.8, 124.1, 37.2, 24.1, 21.7, 10.0, -3.7.

HRMS (EI): Calculated for $C_{25}H_{26}Si$ $[M]^+$: 354.1804. Found: 354.1797.

1,1-Diethyl-2,3-diphenyl-1-sila-2-cyclohexene (3ja):

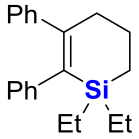

Following procedure **A**, the reaction was carried out with diphenylacetylene (0.2 mmol), silacyclobutane **1j** (0.6 mmol, 3.0 equiv.), Ni(cod)$_2$ (5.8 mg, 0.02 mmol), IPr·HCl (10.2 mg, 0.024 mmol), LiO^{t}Bu (3.2 mg, 0.04 mmol) and toluene (1.0 mL) at 120 ℃ for 24 h. The title compound was obtained in 82% yield (50.3 mg) as colorless oil;

^{1}H NMR (400 MHz, CDCl$_3$): δ 7.10 – 7.04 (m, 4H), 7.03 – 6.99 (m, 1H), 6.99 – 6.93 (m, 3H), 6.84 – 6.77 (m, 2H), 2.59 – 2.53 (m, 2H), 2.05 – 1.96 (m, 2H), 0.96 (t, *J* = 7.9 Hz, 6H), 0.91 – 0.84 (m, 2H), 0.63 – 0.53 (m, 4H).

^{13}C NMR (101 MHz, CDCl$_3$): δ 155.4, 145.0, 143.7, 136.4, 129.0, 128.4, 128.4, 127.6, 127.5, 125.9, 124.6, 37.2, 22.6, 7.8, 7.7, 4.9.

HRMS (EI): Calculated for $C_{21}H_{26}Si$ $[M]^+$: 306.1804. Found: 306.1800.

1,1-Dibutyl-2,3-diphenyl-1-sila-2-cyclohexene (3ka):

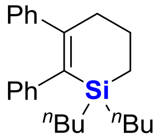

Following procedure **A**, the reaction was carried out with diphenylacetylene (0.2 mmol), silacyclobutane **1k** (0.6 mmol, 3.0 equiv.), Ni(cod)$_2$ (5.8 mg, 0.02 mmol), IPr·HCl (10.2 mg, 0.024 mmol), LiO^{t}Bu (3.2 mg, 0.04 mmol) and toluene (1.0 mL) at 120 ℃ for 24 h. The title compound was obtained in 62% yield (45.0 mg) as colorless oil;

^{1}H NMR (400 MHz, CDCl$_3$): δ 7.09 – 7.02 (m, 4H), 7.02 – 6.98 (m, 1H), 6.98 – 6.92 (m, 3H), 6.79 (dd, *J* = 7.5, 2.1 Hz, 1H), 2.58 – 2.51 (m, 2H), 2.03 – 1.94 (m, 2H), 1.39 – 1.20 (m, 9H), 0.91 – 0.82 (m, 8H), 0.62 – 0.52 (m, 4H).

^{13}C NMR (101 MHz, CDCl$_3$): δ 145.1, 143.7, 137.0, 129.0, 128.4, 127.6, 125.8, 124.6, 37.2, 26.8, 26.4, 22.5, 13.9, 13.1, 8.8.

HRMS (EI): Calculated for $C_{25}H_{34}Si$ $[M]^+$: 362.2430. Found: 362.2423.

1,1-Bis(4-fluorophenyl)-2,3-diphenyl-1-sila-2-cyclohexene (3la):

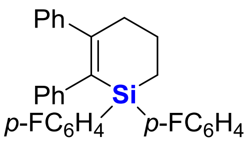

Following procedure **A**, the reaction was carried out with diphenylacetylene (0.2 mmol), silacyclobutane **1l** (0.6 mmol, 3.0 equiv.), Ni(cod)$_2$ (5.8 mg, 0.02 mmol), IPr·HCl (10.2 mg, 0.024 mmol), LiO^{t}Bu (3.2 mg, 0.04 mmol) and toluene (1.0 mL) at 120 ℃ for 24 h. The title compound was obtained in 70% yield (61.4 mg) as colorless oil;

^{1}H NMR (400 MHz, CDCl$_3$): δ 7.46 – 7.39 (m, 4H), 7.13 – 6.99 (m, 10H), 6.88 (m, 3H), 6.60 – 6.54 (m, 2H), 2.78 (t, *J* = 5.8 Hz, 2H), 2.22 – 2.12 (m, 2H), 1.40 – 1.32 (m, 2H).

^{13}C NMR (101 MHz, $CDCl_3$): δ 164.1 (d, *J* = 249.1 Hz), 157.5, 144.4, 142.6, 137.7 (d, *J* = 7.5 Hz), 133.5, 131.1 (d, *J* = 3.8 Hz), 129.9, 128.6, 127.6 (d, *J* = 19.9 Hz), 126.3, 125.0, 115.1 (d, *J* = 19.9 Hz), 37.5, 21.4, 11.2.

^{19}F NMR (376 MHz, $CDCl_3$): δ -111.2.

HRMS (EI): Calculated for $C_{29}H_{24}F_2Si$ $[M]^+$: 438.1615. Found: 438.1604.

Nickel-catalyzed asymmetric ring-opening reaction of SCBs with internal alkynes

General procedure

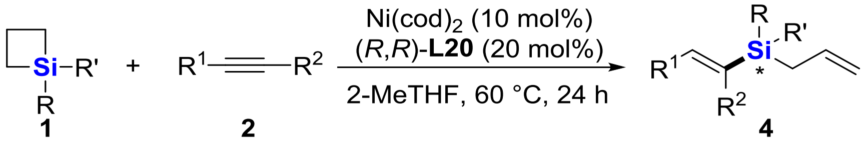

In a nitrogen-filled glove-box, an oven-dried 8 mL glass vial equipped with a magnetic stir bar was charged with $Ni(cod)_2$ (5.8 mg, 0.02 mmol), (*R*,*R*)-**L20** (27.0 mg, 0.04 mmol) and 2-MeTHF (1.0 mL). The mixture was allowed to stir under ambient temperature for 1 h. Then alkynes **2** (0.2 mmol) and silacyclobutanes **1** (0.4 mmol) were added sequentially. The vial was then sealed with a PTFE cap, removed from the glove box and stirred at 60 ℃ for 24 h. After being cooled to room temperature, the solvent was removed under vacuum and the residue was subjected to silica gel column chromatography to give the corresponding products.

Notes: The ee values of products **4cd**, **4cl**, **4da, 4fa-4ha**, **4ja-4la** were determined by HPLC after oxidation as follow.

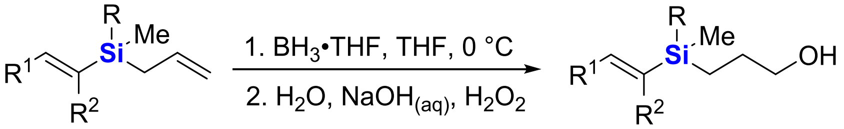

To an oven-dried 8 mL glass vial equipped with a magnetic stir bar was added the alkene substrate (0.1 mmol) and THF (0.2 mL). The solution was then cooled to 0 ℃, BH_3 (1.0 M in THF, 44 μL, 0.044 mmol, 0.22 equiv.) was added to the vial dropwise. The mixture was allowed to stir at 0 ℃ for 1 h. After this time, deionized water (15 μL), 3 M aqueous NaOH (15 μL), and H_2O_2 (15 μL) were added dropwise to the vial sequentially. The mixture was allowed to stir for 1 h at room temperature afterwards. Once complete, the reaction was quenched with water and extracted with EtOAc. The combined organic layers were dried over Na_2SO_4 and concentrated *in vacuo*. The residue was subjected to silica gel column chromatography to give the corresponding products.

Characterization of silicon stereogenic allyl vinylsilanes 4

(*S*, *E*)-allyl(1,2-diphenylvinyl)(methyl)(phenyl)silane (4ba):

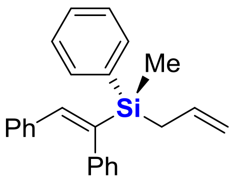

Following the general procedure, the reaction was carried out with diphenylacetylene (0.2 mmol), silacyclobutane **1b** (0.4 mmol), $Ni(cod)_2$ (5.8 mg, 0.02 mmol), (*R*,*R*)-**L20** (27.0 mg, 0.04 mmol) and 2-MeTHF (1.0 mL) at 60 ℃ for 24 h. The title compound was obtained in 88% yield (59.8 mg) as colorless oil, ee = 79%, Optical rotation: $[\alpha]_D^{25}$ 10.7 (c = 0.26, $CHCl_3$).

^{1}H NMR (400 MHz, $CDCl_3$): δ 7.48 (dd, *J* = 7.2, 2.1 Hz, 2H), 7.30 (q, *J* = 6.8, 6.4 Hz, 3H), 7.20 – 7.09 (m, 3H), 7.06 – 6.95 (m, 3H), 6.91 – 6.81 (m, 4H), 6.78 (s, 1H), 5.73 – 5.61 (m, 1H), 4.88 – 4.71 (m, 2H), 1.91 – 1.79 (m, 2H), 0.31 (s, 3H).

^{13}C NMR (101 MHz, $CDCl_3$): δ 143.5, 142.2, 140.2, 137.2, 136.3, 134.7, 134.2, 129.7, 129.4, 128.7, 128.0, 127.9, 127.8, 127.4, 126.0, 114.4, 21.4, -5.3.

HRMS (EI): Calculated for $C_{24}H_{24}Si$ $[M]^+$: 340.1647. Found: 340.1644.

HPLC: Daicel chiralpak OD-3 column, *n*-hexane, 0.3 mL/min, 254 nm UV detector, t (major) = 27.2 min, t (minor) = 28.8 min.

(*S*, *E*)-allyl(1,2-diphenylvinyl)(methyl)(*o*-tolyl)silane (4ca):

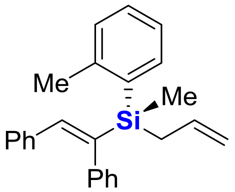

Following the general procedure, the reaction was carried out with diphenylacetylene (0.2 mmol), silacyclobutane **1c** (0.4 mmol), $Ni(cod)_2$ (5.8 mg, 0.02 mmol), (*R*,*R*)-**L20** (27.0 mg, 0.04 mmol) and 2-MeTHF (1.0 mL) at 60 ℃ for 24 h. The title compound was obtained in 92% yield (65.6 mg) as colorless oil, ee = 92%, Optical rotation: $[\alpha]_D^{25}$ 13.5 (c = 2.18, $CHCl_3$).

^{1}H NMR (400 MHz, $CDCl_3$): δ 7.57 – 7.47 (m, 1H), 7.41 – 7.32 (m, 1H), 7.31 – 7.17 (m, 5H), 7.17 – 7.08 (m, 3H), 7.03 – 6.90 (m, 4H), 6.84 (s, 1H), 5.87 – 5.67 (m, 1H), 5.00 – 4.79 (m, 2H), 2.56 (s, 3H), 2.12 – 2.01 (m, 1H), 2.00 – 1.90 (m, 1H), 0.46 (s, 3H).

^{13}C NMR (101 MHz, $CDCl_3$): δ 144.3, 143.7, 142.2, 140.0, 137.3, 135.8, 134.4, 134.4, 130.2, 129.8, 129.7, 128.7, 128.0, 127.8, 127.4, 126.0, 125.1, 114.4, 23.4, 21.9, -4.0.

HRMS (EI): Calculated for $C_{25}H_{26}Si$ $[M]^+$: 354.1804. Found: 354.1799.

HPLC: Daicel chiralpak OD-3 column, *n*-hexane, 0.3 mL/min, 254 nm UV detector, t (major) = 26.8 min, t (minor) = 29.3 min.

(*S*, *E*)-allyl(1,2-di-*p*-tolylvinyl)(methyl)(*o*-tolyl)silane (4cb):

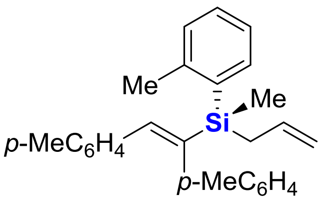

Following the general procedure, the reaction was carried out with alkyne **2t** (0.2 mmol), silacyclobutane **1c** (0.4 mmol), $Ni(cod)_2$ (5.8 mg, 0.02 mmol), (*R*,*R*)-**L20** (27.0 mg, 0.04 mmol) and 2-MeTHF (1.0 mL) at 60 ℃ for 24 h. The title compound was obtained in 91% yield (70.1 mg) as colorless oil, ee = 92%, Optical rotation: $[\alpha]_D^{25}$ 12.1 (c = 2.33, $CHCl_3$).

^{1}H NMR (400 MHz, $CDCl_3$): δ 7.50 – 7.44 (m, 1H), 7.34 – 7.28 (m, 1H), 7.21 – 7.14 (m, 2H), 7.06 – 6.99 (m, 2H), 6.90 (d, *J* = 8.1 Hz, 2H), 6.86 – 6.76 (m, 4H), 6.73 (s, 1H), 5.78 – 5.65 (m, 1H), 4.91 – 4.79 (m, 2H), 2.51 (s, 3H), 2.31 (s, 3H), 2.23 (s, 3H), 2.03 – 1.94 (m, 1H), 1.93 – 1.84 (m, 1H), 0.39 (s, 3H).

^{13}C NMR (101 MHz, CDCl$_3$): δ 144.3, 142.5, 139.8, 139.2, 137.2, 135.8, 135.3, 134.8, 134.7, 134.6, 130.1, 129.7, 129.6, 129.4, 128.7, 127.6, 125.0, 114.2, 23.4, 22.0, 21.3, 21.3, -3.9.

HRMS (EI): Calculated for $C_{27}H_{30}Si$ [M]$^+$: 382.2117. Found: 382.2107.

HPLC: Daicel chiralpak OD-3 column, n-hexane, 0.3 mL/min, 254 nm UV detector, t (major) = 22.9 min, t (minor) = 25.1 min.

(*S*, *E*)-allyl(1,2-bis(4-isopropylphenyl)vinyl)(methyl)(*o*-tolyl)silane (4cc):

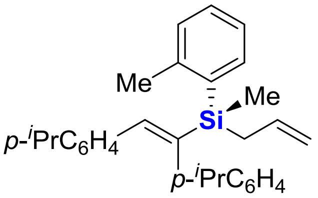

Following the general procedure, the reaction was carried out with alkyne **2u** (0.2 mmol), silacyclobutane **1c** (0.4 mmol), Ni(cod)$_2$ (5.8 mg, 0.02 mmol), (*R*,*R*)-**L20** (27.0 mg, 0.04 mmol) and 2-MeTHF (1.0 mL) at 60 ℃ for 24 h. The title compound was obtained in 80% yield (70.6 mg) as colorless oil, ee = 90%, Optical rotation: $[\alpha]_D^{25}$ 8.7 (c = 2.35, CHCl$_3$).

^{1}H NMR (400 MHz, CDCl$_3$): δ 7.51 – 7.46 (m, 1H), 7.34 – 7.28 (m, 1H), 7.18 (d, *J* = 7.6 Hz, 2H), 7.09 (d, *J* = 8.1 Hz, 2H), 6.97 – 6.91 (m, 2H), 6.89 – 6.81 (m, 4H), 6.71 (s, 1H), 5.79 – 5.61 (m, 1H), 4.93 – 4.76 (m, 2H), 2.93 – 2.83 (m, 1H), 2.82 – 2.72 (m, 1H), 2.49 (s, 3H), 1.25 (d, *J* = 6.9 Hz, 6H), 1.16 (d, *J* = 6.9 Hz, 6H), 0.40 (s, 3H).

^{13}C NMR (101 MHz, CDCl$_3$): δ 148.1, 146.5, 144.3, 142.5, 139.7, 139.6, 135.9, 135.0, 134.8, 134.6, 130.1, 129.7, 129.7, 127.5, 126.8, 126.1, 125.0, 114.2, 33.9, 33.8, 24.2, 23.9, 23.4, 22.0, -4.0.

HRMS (EI): Calculated for $C_{31}H_{38}Si$ [M]$^+$: 438.2743. Found: 438.2736.

HPLC: Phenomenex lux cellulose-3 column, CH_3CN/H_2O = 55/45, 1.0 mL/min, 230 nm UV detector, t (major) = 81.9 min, t (minor) = 89.0 min.

(*S*, *E*)-allyl(1,2-bis(4-(tert-butyl)phenyl)vinyl)(methyl)(*o*-tolyl)silane (4cd):

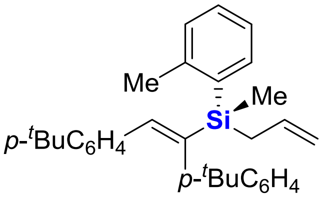

Following the general procedure, the reaction was carried out with alkyne **2v** (0.2 mmol), silacyclobutane **1c** (0.4 mmol), Ni(cod)$_2$ (5.8 mg, 0.02 mmol), (*R*,*R*)-**L20** (27.0 mg, 0.04 mmol) and 2-MeTHF (1.0 mL) at 60 ℃ for 24 h. The title compound was obtained in 75% yield (69.9 mg) as colorless oil. The ee value of **4cd** was measured by chiral HPLC on Daicel OD-3 column after oxidation (91% ee). Optical rotation: $[\alpha]_D^{25}$ 10.3 (c = 2.33, CHCl$_3$).

^{1}H NMR (400 MHz, CDCl$_3$): δ 7.49 (d, *J* = 7.3 Hz, 1H), 7.34 – 7.28 (m, 1H), 7.24 (s, 1H), 7.18 (d, *J* = 7.5 Hz, 2H), 7.09 (d, *J* = 8.3 Hz, 2H), 6.89 – 6.82 (m, 4H), 6.71 (s, 1H), 5.78 – 5.62 (m, 1H), 4.92 – 4.76 (m, 2H), 2.49 (s, 3H), 2.02 – 1.93 (m, 1H), 1.93 – 1.84 (m, 1H), 1.31 (s, 9H), 1.22 (s, 9H), 0.39 (s, 3H).

^{13}C NMR (101 MHz, CDCl$_3$): δ 150.4, 148.8, 142.6, 139.5, 139.2, 135.9, 134.8, 134.7, 134.6, 130.1, 129.7, 129.5, 127.2, 125.6, 124.99, 124.98, 114.2, 34.6, 34.6, 31.6, 31.3, 23.4, 22.0, -3.9.

HRMS (EI): Calculated for $C_{33}H_{42}Si$ [M]$^+$: 466.3056. Found: 466.3048.

(*S*, *E*)-3-((1,2-bis(4-(tert-butyl)phenyl)vinyl)(methyl)(*o*-tolyl)silyl)propan-1-ol (4cd-OH):

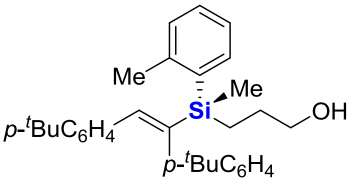

The title compound was obtained as colorless oil after oxidation of **4cd**.

^{1}H NMR (400 MHz, CDCl$_3$): δ 7.40 (dd, *J* = 7.9, 1.5 Hz, 1H), 7.23 (td, *J* = 7.5, 1.5 Hz, 1H), 7.20 – 7.15 (m, 2H), 7.10 (d, *J* = 7.3 Hz, 2H), 7.04 – 6.98 (m, 2H), 6.81 – 6.74 (m, 4H), 6.63 (s, 1H), 3.48 (t, *J* = 6.6 Hz, 2H), 2.41 (s, 3H), 1.49 – 1.39 (m, 2H), 1.24 (s, 9H), 1.15 (s, 9H), 0.95 – 0.86 (m, 1H), 0.81 (m, 1H), 0.33 (s, 3H).

^{13}C NMR (101 MHz, CDCl$_3$): δ 144.4, 143.0, 139.4, 139.3, 135.8, 135.0, 130.1, 129.6, 129.4, 127.1, 125.6, 125.04, 124.98, 65.8, 34.6, 34.6, 31.6, 31.3, 29.8, 27.3, 23.4, 9.6, -3.7.

HRMS (ESI) Calculated for [$C_{33}H_{44}OSi$ + Na]$^+$: 507.3054. Found: 507.3045.

HPLC: Daicel chiralpak OD-3 column, *n*-hexane/*i*-PrOH = 90/10, 0.5 mL/min, 254 nm UV detector, t (major) = 9.4 min, t (minor) = 11.5 min; ee = 91%.

(*S*, *E*)-allyl(1,2-bis(4-methoxyphenyl)vinyl)(methyl)(*o*-tolyl)silane (4ce):

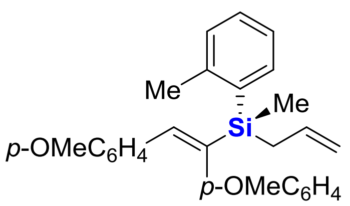

Following the general procedure, the reaction was carried out with alkyne **2d** (0.2 mmol), silacyclobutane **1c** (0.4 mmol), Ni(cod)$_2$ (5.8 mg, 0.02 mmol), (*R*,*R*)-**L20** (27.0 mg, 0.04 mmol) and 2-MeTHF (1.0 mL) at 60 ℃ for 24 h. The title compound was obtained in 80% yield (66.2 mg) as colorless oil, ee = 90%, Optical rotation: $[\alpha]_D^{25}$ 5.2 (c = 0.36, CHCl$_3$).

^{1}H NMR (400 MHz, CDCl$_3$): δ 7.39 (dd, *J* = 7.4, 1.5 Hz, 1H), 7.27 – 7.21 (m, 1H), 7.13 – 7.07 (m, 2H), 6.84 – 6.79 (m, 2H), 6.78 – 6.68 (m, 4H), 6.65 (s, 1H), 6.59 – 6.53 (m, 2H), 5.72 – 5.59 (m, 1H), 4.86 – 4.72 (m, 2H), 3.72 (s, 3H), 3.65 (s, 3H), 2.43 (s, 3H), 1.96 – 1.88 (m, 1H), 1.86 – 1.78 (m, 1H), 0.33 (s, 3H).

^{13}C NMR (101 MHz, CDCl$_3$): δ 158.6, 157.8, 144.2, 140.4, 139.4, 135.7, 134.7, 134.5, 134.4, 130.9, 130.2, 130.0, 129.5, 128.8, 124.9, 114.1, 114.1, 113.3, 55.1, 23.2, 21.9, -4.1.

HRMS (EI): Calculated for $C_{27}H_{30}O_2Si$ [M]$^+$: 414.2015. Found: 414.2008.

HPLC: Daicel chiralpak OD-3 column, *n*-hexane/*i*-PrOH = 99.5/0.5, 0.5 mL/min, 254 nm UV detector, t (major) = 12.5 min, t (minor) = 13.5 min.

(*S*, *E*)-4,4'-(1-(allyl(methyl)(*o*-tolyl)silyl)ethene-1,2-diyl)bis(N,N-dimethylaniline) (4cf):

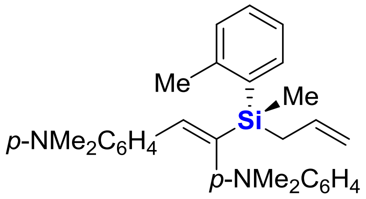

Following the general procedure, the reaction was carried out with alkyne **2e** (0.2 mmol), silacyclobutane **1c** (0.4 mmol), Ni(cod)$_2$ (5.8 mg, 0.02 mmol), (*R*,*R*)-**L20** (27.0 mg, 0.04 mmol) and 2-MeTHF (1.0 mL) at 60 ℃ for 24 h. The title compound was obtained in 40% yield (33.0 mg) as colorless oil, ee = 90%, Optical rotation: $[\alpha]_D^{25}$ 13.7 (c = 1.10, CHCl$_3$).

^{1}H NMR (400 MHz, CDCl$_3$): δ 7.48 (d, *J* = 7.3 Hz, 1H), 7.32 – 7.27 (m, 1H), 7.17 (d, *J* = 7.7 Hz, 2H), 6.88 (d, *J* = 8.4 Hz, 2H), 6.81 (d, *J* = 8.1 Hz, 2H), 6.65 (s, 3H), 6.45 (d, *J* = 8.4 Hz, 2H), 5.79 – 5.67 (m, 1H), 4.90 – 4.77 (m, 2H), 2.94 (s, 6H), 2.88 (s, 6H), 2.51 (s, 3H), 2.03 – 1.94 (m, 1H), 1.92 – 1.84 (m, 1H), 0.38 (s, 3H).

^{13}C NMR (101 MHz, CDCl$_3$): δ 149.5, 148.8, 144.4, 139.8, 138.1, 135.9, 135.6, 135.1, 131.1, 130.8, 130.0, 129.4, 128.7, 126.4, 124.9, 113.8, 113.2, 111.8, 40.9, 40.4, 23.4, 22.2, -3.8.

HRMS (ESI): Calculated for [$C_{29}H_{36}N_2Si$ + H]$^+$: 441.2721. Found: 441.2716.

HPLC: Phenomenex lux cellulose-3 column, CH_3CN/H_2O = 70/30, 1.0 mL/min, 230 nm UV detector, t (major) = 12.3 min, t (minor) = 14.6 min.

(*S*,*E*)-allyl(1,2-bis(4-(methylthio)phenyl)vinyl)(methyl)(*o*-tolyl)silane (4cg):

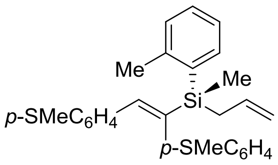

Following the general procedure, the reaction was carried out with alkyne **2w** (0.2 mmol), silacyclobutane **1c** (0.3 mmol), Ni(cod)$_2$ (5.8 mg, 0.02 mmol), (*R*,*R*)-**L20** (27.0 mg, 0.04 mmol) and 2-MeTHF (1.0 mL) at 60 ℃ for 24 h. The title compound was obtained in 70% yield (62.5 mg) as colorless oil, ee = 90%, Optical rotation: $[\alpha]_D^{25}$ 7.4 (c = 1.14, $CHCl_3$).

^{1}H NMR (400 MHz, $CDCl_3$): δ 7.45 (dd, *J* = 7.3, 1.5 Hz, 1H), 7.35 – 7.28 (m, 1H), 7.21 – 7.14 (m, 2H), 7.13 – 7.08 (m, 2H), 6.99 – 6.93 (m, 2H), 6.89 – 6.84 (m, 2H), 6.84 – 6.78 (m, 2H), 6.73 (s, 1H), 5.79 – 5.63 (m, 1H), 4.93 – 4.80 (m, 2H), 2.49 (s, 3H), 2.47 (s, 3H), 2.40 (s, 3H), 2.04 – 1.95 (m, 1H), 1.94 – 1.84 (m, 1H), 0.40 (s, 3H).

^{13}C NMR (101 MHz, $CDCl_3$): δ 144.2, 142.4, 139.5, 139.0, 137.8, 135.8, 135.7, 134.4, 134.3, 134.0, 130.2, 130.0, 129.8, 128.3, 126.8, 125.7, 125.1, 114.4, 23.4, 21.8, 15.9, 15.5, -4.0.

HRMS (ESI): Calculated for $[C_{27}H_{30}S_2Si, M+Na]^+$: 469.1450. Found: 469.1445.

HPLC: Daicel chiralpak OD-3 column, *n*-hexane/*i*-PrOH = 99/1, 0.5 mL/min, 254 nm UV detector, t (major) = 11.0 min, t (minor) = 11.6 min.

(*S*, *E*)-allyl(1,2-bis(4-fluorophenyl)vinyl)(methyl)(*o*-tolyl)silane (4ch):

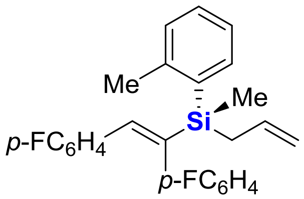

Following the general procedure, the reaction was carried out with alkyne **2f** (0.2 mmol), silacyclobutane **1c** (0.4 mmol), Ni(cod)$_2$ (5.8 mg, 0.02 mmol), (*R*,*R*)-**L20** (27.0 mg, 0.04 mmol) and 2-MeTHF (1.0 mL) at 60 ℃ for 24 h. The title compound was obtained in 86% yield (67.6 mg) as colorless oil, ee = 90%, Optical rotation: $[\alpha]_D^{25}$ 9.7 (c = 2.25, $CHCl_3$).

^{1}H NMR (400 MHz, $CDCl_3$): δ 7.46 – 7.41 (m, 1H), 7.35 – 7.29 (m, 1H), 7.21 – 7.15 (m, 2H), 6.95 – 6.85 (m, 4H), 6.84 – 6.75 (m, 5H), 5.78 – 5.65 (m, 1H), 4.95 – 4.80 (m, 2H), 2.49 (s, 3H), 2.05 – 1.96 (m, 1H), 1.95 – 1.86 (m, 1H), 0.42 (s, 3H).

^{13}C NMR (101 MHz, $CDCl_3$): δ 161.9 (d, *J* = 248.1 Hz), 161.5 (d, *J* = 244.9 Hz), 144.2, 142.4 (d, *J* = 1.8 Hz), 139.3, 135.8, 137.6 (d, *J* = 3.6 Hz), 134.1, 133.3 (d, *J* = 3.5 Hz), 131.3 (d, *J* = 8.0 Hz), 130.2, 129.3 (d, *J* = 7.6 Hz), 125.2, 115.8 (d, *J* = 21.1 Hz), 115.0 (d, *J* = 21.2 Hz), 114.6, 23.4, 21.8, -4.0.

^{19}F NMR (376 MHz, $CDCl_3$): δ -113.74, -116.75.

HRMS (EI): Calculated for $C_{25}H_{24}F_2Si$ $[M]^+$: 390.1615. Found: 390.1606.

HPLC: Daicel chiralpak OD-3 column, *n*-hexane, 0.3 mL/min, 254 nm UV detector, t (major) = 25.2 min, t (minor) = 27.0 min.

(*S*, *E*)-allyl(1,2-di-*m*-tolylvinyl)(methyl)(*o*-tolyl)silane (4ci):

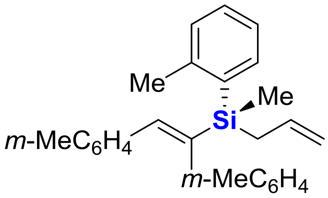

Following the general procedure, the reaction was carried out with alkyne **2g** (0.2 mmol), silacyclobutane **1c** (0.4 mmol), Ni(cod)$_2$ (5.8 mg, 0.02 mmol), (*R*,*R*)-**L20** (27.0 mg, 0.04 mmol) and 2-MeTHF (1.0 mL) at 60 ℃ for 24 h. The

title compound was obtained in 83% yield (63.5 mg) as colorless oil, ee = 91%, Optical rotation: $[\alpha]_D^{25}$ 9.3 (c = 2.12, $CHCl_3$).

^{1}H NMR (400 MHz, $CDCl_3$): δ 7.48 (dd, *J* = 7.4, 1.5 Hz, 1H), 7.36 – 7.28 (m, 1H), 7.23 – 7.15 (m, 2H), 7.15 – 7.08 (m, 1H), 7.02 – 6.88 (m, 3H), 6.78 (s, 1H), 6.72 (dd, *J* = 8.3, 2.3 Hz, 4H), 5.80 – 5.66 (m, 1H), 4.94 – 4.79 (m, 2H), 2.23 (s, 3H), 2.15 (s, 3H), 2.05 – 1.96 (m, 1H), 1.94 – 1.85 (m, 1H), 0.42 (s, 3H).

^{13}C NMR (101 MHz, $CDCl_3$): δ 144.3, 143.6, 139.8, 138.1, 137.5, 137.3, 135.9, 134.6, 134.5, 130.8, 130.1, 129.7, 128.5, 128.3, 128.1, 127.9, 126.6, 126.6, 125.0, 124.8, 114.3, 23.4, 21.9, 21.6, 21.4, -4.0.

HRMS (EI): Calculated for $C_{27}H_{30}Si$ $[M]^+$: 382.2117. Found: 382.2118.

HPLC: Daicel chiralpak OD-3 column, *n*-hexane, 0.3 mL/min, 254 nm UV detector, t (major) = 21.0 min, t (minor) = 22.5 min.

(*S*, *E*)-allyl(1,2-bis(3-fluorophenyl)vinyl)(methyl)(*o*-tolyl)silane (4cj):

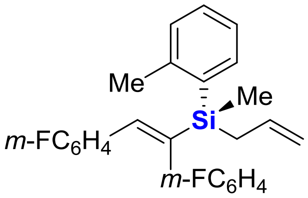

Following the general procedure, the reaction was carried out with alkyne **2i** (0.2 mmol), silacyclobutane **1c** (0.4 mmol), $Ni(cod)_2$ (5.8 mg, 0.02 mmol), (*R*,*R*)-**L20** (27.0 mg, 0.04 mmol) and 2-MeTHF (1.0 mL) at 60 ℃ for 24 h. The title compound was obtained in 88% yield (69.1 mg) as colorless oil, ee = 90%, Optical rotation: $[\alpha]_D^{25}$ 11.5 (c = 2.30, $CHCl_3$).

^{1}H NMR (400 MHz, $CDCl_3$): δ 7.45 (dd, *J* = 7.3, 1.5 Hz, 1H), 7.36 – 7.30 (m, 1H), 7.23 – 7.16 (m, 3H), 7.11 – 7.03 (m, 1H), 6.92 – 6.85 (m, 1H), 6.84 – 6.78 (m, 1H), 6.76 (s, 1H), 6.73 – 6.69 (m, 1H), 6.68 – 6.64 (m, 1H), 6.61 – 6.55 (m, 2H), 5.78 – 5.64 (m, 1H), 4.94 – 4.82 (m, 2H), 2.50 (s, 3H), 2.05 – 1.98 (m, 1H), 1.94 – 1.87 (m, 1H), 0.43 (s, 3H).

^{13}C NMR (101 MHz, $CDCl_3$): δ 163.2 (d, *J* = 246.6 Hz), 162.5 (d, *J* = 245.1 Hz), 144.3 (d, *J* = 1.6 Hz), 144.1, 144.0 (d, *J* = 7.4 Hz), 139.2 (d, *J* = 2.5 Hz), 139.1 (d, *J* = 7.7 Hz), 135.8, 133.9 (d, *J* = 1.9 Hz), 133.7, 130.4 (d, *J* = 8.6 Hz), 130.1, 129.5 (d, *J* = 8.4 Hz), 125.4 (d, *J* = 2.9 Hz), 125.2, 123.4 (d, *J* = 3.0 Hz), 116.0 (d, *J* = 22.2 Hz), 114.8 (d, *J* = 1.7 Hz), 114.6 (d, *J* = 3.7 Hz), 114.4 (d, *J* = 3.7 Hz), 113.2 (d, *J* = 21.0 Hz), 23.4, 21.8, -4.0.

^{19}F NMR (376 MHz, $CDCl_3$): δ -112.63, -113.35.

HRMS (EI): Calculated for $C_{25}H_{24}F_2Si$ $[M]^+$: 390.1615. Found: 390.1607.

HPLC: Daicel chiralpak OD-3 column, *n*-hexane, 0.5 mL/min, 254 nm UV detector, t (major) = 17.0 min, t (minor) = 20.6 min.

(*S*, *E*)-allyl(1,2-bis(3-methoxyphenyl)vinyl)(methyl)(*o*-tolyl)silane (4ck):

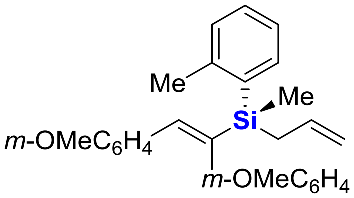

Following the general procedure, the reaction was carried out with alkyne **2h** (0.2 mmol), silacyclobutane **1c** (0.4 mmol), $Ni(cod)_2$ (5.8 mg, 0.02 mmol), (*R*,*R*)-**L20** (27.0 mg, 0.04 mmol) and 2-MeTHF (1.0 mL) at 60 ℃ for 24 h. The title compound was obtained in 86% yield (71.5 mg) as colorless oil, ee = 91%, Optical rotation: $[\alpha]_D^{25}$ 5.8 (c = 2.30, $CHCl_3$).

^{1}H NMR (400 MHz, $CDCl_3$): δ 7.45 (dd, *J* = 7.4, 1.4 Hz, 1H), 7.34 – 7.29 (m, 1H), 7.22 – 7.12 (m, 3H), 7.04 (t, *J* = 7.9 Hz, 1H), 6.76 (s, 1H), 6.72 – 6.68 (m, 1H), 6.68 – 6.64 (m, 1H), 6.61 (d, *J* = 7.6 Hz, 1H), 6.56 – 6.52 (m, 1H), 6.51 – 6.47 (m, 1H), 6.39 (dd, *J* = 2.6, 1.5 Hz, 1H), 5.82 – 5.66 (m, 1H), 4.95 – 4.82 (m, 2H), 3.59 (s, 3H), 3.46 (s, 3H), 2.51 (s, 3H), 2.06 – 1.97 (m, 1H), 1.95 – 1.85 (m, 1H), 0.43 (s, 3H).

^{13}C NMR (101 MHz, $CDCl_3$): δ 160.0, 159.1, 144.3, 143.8, 143.7, 139.6, 138.5, 135.9, 134.42, 134.35, 130.2, 129.8, 129.8, 129.0, 125.1, 122.6, 120.2, 114.5, 114.3, 113.7, 112.7, 112.1, 55.2, 54.9, 23.4, 21.8, -4.1.

HRMS (EI): Calculated for $C_{27}H_{30}O_2Si$ $[M]^+$: 414.2015. Found: 414.2010.

HPLC: Daicel chiralpak OD-3 column, *n*-hexane/*i*-PrOH = 99.5/0.5, 0.5 mL/min, 254 nm UV detector, t (major) = 11.6 min, t (minor) = 13.2 min.

(*S*,*E*)-allyl(1,2-bis(3-(trifluoromethyl)phenyl)vinyl)(methyl)(o-tolyl)silane (4cl):

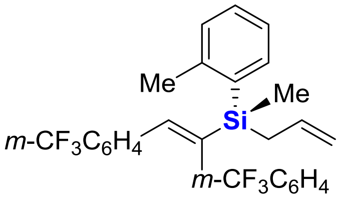

Following the general procedure, the reaction was carried out with alkyne **2j** (0.2 mmol), silacyclobutane **1c** (0.4 mmol), Ni(cod)$_2$ (5.8 mg, 0.02 mmol), (*R*,*R*)-**L20** (27.0 mg, 0.04 mmol) and 2-MeTHF (1.0 mL) at 60 ℃ for 24 h. The title compound was obtained in 72% yield (70.5 mg) as colorless oil, The ee value of **4cl** was measured by chiral HPLC on Daicel OD-3 column with the corresponding alcohol derivative (82% ee). Optical rotation: $[\alpha]_D^{25}$ -8.7 (c = 0.74, $CHCl_3$).

^{1}H NMR (400 MHz, $CDCl_3$): δ 7.47 – 7.39 (m, 2H), 7.38 – 7.30 (m, 3H), 7.24 – 7.16 (m, 3H), 7.10 (d, *J* = 13.9 Hz, 2H), 7.06 – 7.00 (m, 2H), 6.90 (s, 1H), 5.80 – 5.66 (m, 1H), 4.96 – 4.85 (m, 2H), 2.50 (s, 3H), 2.09 – 1.99 (m, 1H), 1.97 – 1.88 (m, 1H), 0.47 (s, 3H).

^{13}C NMR (101 MHz, $CDCl_3$): δ 144.8, 144.0, 142.2, 139.2, 137.2, 135.6, 133.5, 132.4 (q, *J* = 1.0 Hz), 131.1 (q, *J* = 32.1 Hz), 131.0 (q, *J* = 1.1 Hz), 130.5 (q, *J* = 32.1 Hz), 129.2, 128.5, 126.2 (q, *J* = 3.8 Hz), 124.5 (q, *J* = 3.8 Hz), 124.1 (q, *J* = 3.7 Hz), 123.9 (q, *J* = 272.6 Hz), 123.8 (q, *J* = 272.4 Hz), 123.0 (q, *J* = 3.8 Hz), 114.9, 23.3, 21.5, -4.2.

^{19}F NMR (376 MHz, $CDCl_3$): δ -62.9, -63.1.

HRMS (ESI): Calculated for $[C_{27}H_{24}F_6Si, M+Na]^+$: 513.1444. Found: 513.1440.

(*S*,*E*)-3-((1,2-bis(3-(trifluoromethyl)phenyl)vinyl)(methyl)(o-tolyl)silyl)propan-1-ol (4cl-OH):

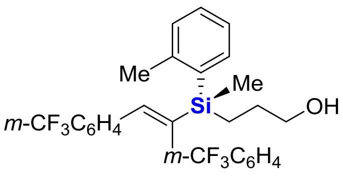

The title compound was obtained as colorless oil after oxidation of **4cl**.

^{1}H NMR (400 MHz, CDCl3): δ 7.46 – 7.39 (m, 2H), 7.38 – 7.30 (m, 3H), 7.23 – 7.16 (m, 3H), 7.12 (s, 1H), 7.07 (s, 1H), 7.06 – 6.99 (m, 2H), 6.90 (s, 1H), 3.61 (t, *J* = 6.6 Hz, 2H), 2.50 (s, 3H), 1.62 – 1.54 (m, 2H), 1.13 – 1.00 (m, 1H), 0.97 – 0.87 (m, 1H), 0.48 (s, 3H).

^{13}C NMR (101 MHz, $CDCl_3$): δ 145.4, 144.1, 142.5, 139.0, 137.4, 135.7, 133.4, 132.5, 131.2 (d, *J* = 32.0 Hz), 131.0, 130.6 (d, *J* = 31.6 Hz), 130.4, 130.2, 129.3, 128.6, 126.3 (q, *J* = 4.0 Hz), 125.4, 124.4 (q, *J* = 3.3 Hz), 124.2 (q, *J* = 3.4 Hz), 123.9 (d, *J* = 272.5 Hz), 123.8 (d, *J* = 272.4 Hz), 123.1 (q, *J* = 3.3 Hz), 65.6, 27.1, 23.3, 9.5, -3.8.

^{19}F NMR (376 MHz, $CDCl_3$): δ -62.9, -63.1.

HRMS (ESI): Calculated for $[C_{27}H_{26}F_6OSi, M+Na]^+$: 531.1549. Found: 531.1550.

HPLC: Daicel chiralpak OD-3 column, *n*-hexane/*i*-PrOH = 90/10, 0.5 mL/min, 254 nm UV detector, t (major) = 14.0 min, t (minor) = 15.9 min, ee = 82%.

(*S*,*E*)-allyl(1,2-bis(2-fluorophenyl)vinyl)(methyl)(*o*-tolyl)silane (4cm):

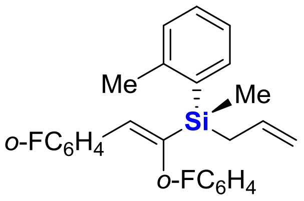

Following the general procedure, the reaction was carried out with alkyne **2k** (0.2 mmol), silacyclobutane **1c** (0.4 mmol), $Ni(cod)_2$ (5.8 mg, 0.02 mmol), (*R*,*R*)-**L20** (27.0 mg, 0.04 mmol) and 2-MeTHF (1.0 mL) at 60 ℃ for 24 h. The title compound was obtained in 72% yield (56.2 mg) as colorless oil, ee = 84%, Optical rotation: $[\alpha]_D^{25}$ 22.2 (c = 0.93, $CHCl_3$).

^{1}H NMR (400 MHz, $CDCl_3$): δ 7.51 (dd, *J* = 7.4, 1.5 Hz, 1H), 7.34 – 7.28 (m, 1H), 7.22 – 7.04 (m, 5H), 6.99 – 6.89 (m, 3H), 6.86 – 6.71 (m, 3H), 5.81 – 5.66 (m, 1H), 4.94 – 4.78 (m, 2H), 2.52 (s, 3H), 2.10 – 2.02 (m, 1H), 1.99 – 1.90 (m, 1H), 0.46 (s, 3H).

^{13}C NMR (101 MHz, $CDCl_3$): δ 160.6 (d, *J* = 248.6 Hz), 159.0 (d, *J* = 244.0 Hz), 144.5, 139.6 (d, *J* = 1.8 Hz), 135.9, 134.7 (d, *J* = 4.9 Hz), 134.1 (d, *J* = 1.9 Hz), 133.8, 130.2, 130.0 (d, *J* = 4.0 Hz), 129.9, 129.5 (d, *J* = 3.1 Hz), 129.2 (d, *J* = 8.5 Hz), 128.1 (d, *J* = 7.7 Hz), 125.4 (d, *J* = 12.7 Hz), 124.2 (d, *J* = 3.6 Hz), 123.5 (d, *J* = 3.6 Hz), 115.6 (d, *J* = 22.3 Hz), 115.3 (d, *J* = 22.1 Hz), 114.6, 23.4, 21.9, -3.9 (d, *J* = 1.1 Hz).

^{19}F NMR (376 MHz, $CDCl_3$): δ -113.6, -116.1.

HRMS (ESI): Calculated for $[C_{25}H_{24}F_2Si, M+Na]^+$: 413.1508. Found: 413.1504.

HPLC: Daicel chiralpak OD-3 column, *n*-hexane, 0.3 mL/min, 254 nm UV detector, t (major) = 24.9 min, t (minor) = 26.3.

(*S*,*E*)-allyl(1,2-di(thiophen-2-yl)vinyl)(methyl)(*o*-tolyl)silane (4cn):

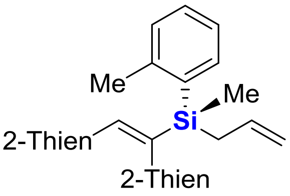

Following the general procedure, the reaction was carried out with alkyne **2o** (0.2 mmol), silacyclobutane **1c** (0.4 mmol), $Ni(cod)_2$ (5.8 mg, 0.02 mmol), (*R*,*R*)-**L20** (27.0 mg, 0.04 mmol) and 2-MeTHF (1.0 mL) at 60 ℃ for 24 h. The title compound was obtained in 60% yield (44.0 mg) as colorless oil, ee = 76%, Optical rotation: $[\alpha]_D^{25}$ 6.1 (c = 0.93, $CHCl_3$).

^{1}H NMR (400 MHz, $CDCl_3$): δ 7.51 (dd, *J* = 7.3, 1.5 Hz, 1H), 7.40 – 7.30 (m, 2H), 7.25 – 7.18 (m, 2H), 7.17 – 7.13 (m, 1H), 7.10 (s, 1H), 7.06 (dd, *J* = 5.1, 3.4 Hz, 1H), 6.95 (dd, *J* = 3.6, 1.3 Hz, 1H), 6.89 (dd, *J* = 5.1, 3.6 Hz, 1H), 6.66 (dd, *J* = 3.5, 1.2 Hz, 1H), 5.86 – 5.69 (m, 1H), 5.00 – 4.84 (m, 2H), 2.52 (s, 3H), 2.10 – 1.97 (m, 2H), 0.49 (s, 3H).

^{13}C NMR (101 MHz, $CDCl_3$): δ 144.3, 141.7, 140.5, 136.8, 135.9, 134.2, 133.8, 133.3, 130.22, 130.16, 129.9, 128.2, 127.8, 126.0, 125.8, 125.2, 125.1, 114.6, 29.8, 23.4, 21.7, -4.2.

HRMS (ESI): Calculated for $[C_{21}H_{22}S_2Si, M+Na]^+$: 389.0824. Found: 389.0828.

HPLC: Daicel chiralpak OD-3 column, *n*-hexane/*i*-PrOH = 99.8/0.2, 0.5 mL/min, 254 nm UV detector, t (major) = 26.1 min, t (minor) = 28.9 min.

(*S*, *E*)-allyl(1,2-bis(3,5-dimethylphenyl)vinyl)(methyl)(*o*-tolyl)silane (4co):

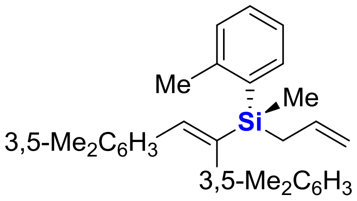

Following the general procedure, the reaction was carried out with alkyne **2m** (0.2 mmol), silacyclobutane **1c** (0.4 mmol), $Ni(cod)_2$ (5.8 mg, 0.02 mmol), (*R*,*R*)-**L20** (27.0 mg, 0.04 mmol) and 2-MeTHF (1.0 mL) at 60 ℃ for 24 h. The title compound was obtained in 90% yield (74.2 mg) as colorless oil, ee = 91%, Optical rotation: $[\alpha]_D^{25}$ 7.6 (c = 2.45, $CHCl_3$).

^{1}H NMR (400 MHz, $CDCl_3$): δ 7.47 (dd, *J* = 7.4, 1.5 Hz, 1H), 7.35 – 7.28 (m, 1H), 7.23 – 7.14 (m, 2H), 6.81 (s, 1H), 6.73 (s, 1H), 6.65 (s, 1H), 6.59 – 6.54 (m, 2H), 6.53 – 6.47 (m, 2H), 5.80 – 5.65 (m, 1H), 4.95 – 4.78 (m, 2H), 2.51 (s, 3H), 2.19 (s, 6H), 2.08 (s, 6H), 2.02 – 1.94 (m, 1H), 1.93 – 1.84 (m, 1H), 0.41 (s, 3H).

^{13}C NMR (101 MHz, $CDCl_3$): δ 144.3, 143.5, 142.2, 139.6, 137.9, 137.3, 137.2, 135.9, 134.7, 134.7, 130.1, 129.7, 129.0, 127.7, 127.4, 125.3, 125.0, 114.12 23.4, 21.9, 21.3, -4.0.

HRMS (EI): Calculated for $C_{29}H_{34}Si$ $[M]^+$: 410.2430. Found: 410.2430.

HPLC: Phenomenex lux cellulose-3 column, CH_3CN/H_2O = 55/45, 1.0 mL/min, 230 nm UV detector, t (major) = 40.1 min, t (minor) = 46.1 min.

(*R*,*E*)-allyl(1,8-diphenyloct-4-en-4-yl)(4-methoxynaphthalen-1-yl)(methyl)silane (4cp):

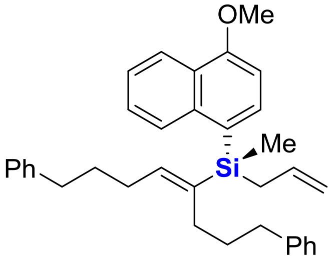

Following the general procedure, the reaction was carried out with alkyne **2n** (0.2 mmol), silacyclobutane **1q** (0.4 mmol), $Ni(cod)_2$ (5.8 mg, 0.02 mmol), (*R*,*R*)-**L20** (27.0 mg, 0.04 mmol) and 2-MeTHF (1.0 mL) at 60 ℃ for 24 h. The title compound was obtained in 85% yield (89.6 mg) as colorless oil, ee = 48%, Optical rotation: $[\alpha]_D^{25}$ 1.6 (c = 1.0, $CHCl_3$).

^{1}H NMR (400 MHz, $CDCl_3$): δ 8.29 – 8.20 (m, 1H), 8.03 – 7.92 (m, 1H), 7.51 (d, *J* = 7.6 Hz, 1H), 7.40 – 7.31 (m, 2H), 7.18 (d, *J* = 7.3 Hz, 2H), 7.14 – 7.01 (m, 6H), 6.87 – 6.80 (m, 2H), 6.73 (d, *J* = 7.7 Hz, 1H), 5.86 (t, *J* = 6.9 Hz, 1H), 5.74 – 5.54 (m, 1H), 4.82 – 4.67 (m, 2H), 3.94 (s, 3H), 2.56 – 2.43 (m, 2H), 2.32 (t, *J* = 7.7 Hz, 2H), 2.12 – 1.89 (m, 6H), 1.65 – 1.53 (m, 2H), 1.45 – 1.34 (m, 2H), 0.37 (s, 3H).

^{13}C NMR (101 MHz, $CDCl_3$): δ157.0, 143.1, 142.6, 142.5, 139.2, 138.4, 135.1, 135.1, 128.6, 128.4, 128.3, 128.2, 126.2, 125.9, 125.9, 125.8, 125.7, 124.9, 122.6, 113.8, 103.4, 55.5, 36.2, 35.7, 31.7, 31.4, 29.9, 28.4, 22.8, -3.3.

HRMS (EI): Calculated for $[C_{35}H_{40}OSi, M+Na]^+$: 527.2741. Found: 527.2740.

HPLC: Daicel chiralpak OD-3 column, *n*-hexane/*i*-PrOH = 99.2/0.8, 0.5 mL/min, 254 nm UV detector, t (major) = 19.8 min, t (minor) = 21.5 min.

(*S*, *E*)-allyl(1,2-diphenylvinyl)(2-ethylphenyl)(methyl)silane (4da):

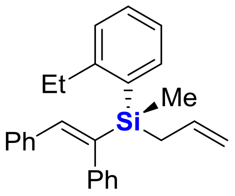

Following the general procedure, the reaction was carried out with diphenylacetylene (0.2 mmol), silacyclobutane **1m** (0.4 mmol), $Ni(cod)_2$ (5.8 mg, 0.02 mmol), (*R*,*R*)-**L23** (45.0 mg, 0.04 mmol) and 2-MeTHF (1.0 mL) at 60 ℃ for 24 h. The title compound was obtained in 35% yield (25.7 mg) as colorless oil; The ee value of **4da** was measured by chiral HPLC on Daicel OD-3 column after oxidation (84% ee). Optical rotation: $[\alpha]_D^{25}$ 12.1 (c = 0.33, $CHCl_3$).

^{1}H NMR (400 MHz, $CDCl_3$): δ 7.41 (dd, *J* = 7.5, 1.5 Hz, 1H), 7.31 (td, *J* = 7.5, 1.5 Hz, 1H), 7.24 – 7.08 (m, 5H), 7.04 – 6.96 (m, 3H), 6.84 (m, 4H), 6.72 (s, 1H), 5.74 – 5.57 (m, 1H), 4.88 – 4.71 (m, 2H), 2.78 (q, *J* = 7.5 Hz, 2H), 1.87 (m, 2H), 1.17 (t, *J* = 7.5 Hz, 3H), 0.34 (s, 3H).

^{13}C NMR (101 MHz, $CDCl_3$): δ 150.6, 144.2, 142.2, 140.0, 137.4, 135.9, 134.5, 134.0, 130.0 129.7, 128.7, 128.3, 128.0, 127.8, 127.3, 126.0, 125.2, 114.4, 29.4, 22.4, 16.1, -3.4.

HRMS (EI): Calculated for $C_{26}H_{28}Si$ $[M]^+$: 368.1960. Found: 368.1955.

(*S*, *E*)-3-((1,2-diphenylvinyl)(2-ethylphenyl)(methyl)silyl)propan-1-ol (4da-OH):

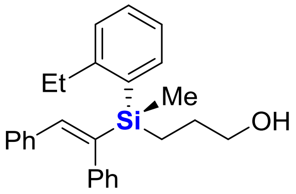

The title compound was obtained as colorless oil after oxidation of **4da.**

^{1}H NMR (400 MHz, $CDCl_3$): δ 7.39 (dd, *J* = 7.4, 1.4 Hz, 1H), 7.33 – 7.27 (m, 1H), 7.21 (d, *J* = 7.1 Hz, 1H), 7.18 – 7.07 (m, 4H), 7.02 – 6.96 (m, 3H), 6.87 – 6.81 (m, 4H), 6.71 (s, 1H), 3.48 (t, *J* = 6.7 Hz, 2H), 2.77 (q, *J* = 7.5 Hz, 2H), 1.54 – 1.42 (m, 2H), 1.16 (t, *J* = 7.5 Hz, 4H), 0.93 – 0.75 (m, 2H), 0.35 (s, 3H).

^{13}C NMR (101 MHz, $CDCl_3$): δ 150.6, 144.6, 142.4, 139.8, 137.4, 135.8, 134.2, 129.9, 129.7, 128.7, 128.3, 128.0, 127.7, 127.3, 126.0, 125.2, 65.8, 29.3, 27.4, 16.1, 10.1, -3.2.

HRMS (ESI): Calculated for $[C_{26}H_{30}OSi, M+Na]^+$: 409.1958. Found: 409.1958.

HPLC: Daicel chiralpak OD-3 column, *n*-hexane/*i*-PrOH = 90/10, 0.5 mL/min, 254 nm UV detector, t (major) = 11.3 min, t (minor) = 12.3 min. ee = 84%.

(*R*, *E*)-allyl(1,2-diphenylvinyl)(2-methoxyphenyl)(methyl)silane (4ea):

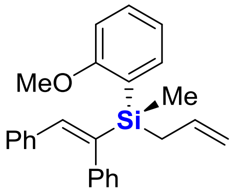

Following the general procedure, the reaction was carried out with diphenylacetylene (0.2 mmol), silacyclobutane **1n** (0.4 mmol), $Ni(cod)_2$ (5.8 mg, 0.02 mmol), (*R*,*R*)-**L20** (27.0 mg, 0.04 mmol) and 2-MeTHF (1.0 mL) at 60 ℃ for 24 h. The title compound was obtained in 69% yield (51.4 mg) as colorless oil, ee = 82%, Optical rotation: $[\alpha]_D^{25}$ 14.8 (c = 1.70, $CHCl_3$).

^{1}H NMR (400 MHz, $CDCl_3$): δ 7.44 (dd, *J* = 7.2, 1.8 Hz, 1H), 7.42 – 7.35 (m, 1H), 7.25 – 7.12 (m, 3H), 7.07 (q, *J* = 3.6 Hz, 3H), 6.99 – 6.90 (m, 5H), 6.84 (t, *J* = 4.1 Hz, 2H), 5.81 – 5.67 (m, 1H), 4.93 – 4.75 (m, 2H), 3.75 (s, 3H), 2.10 – 2.01 (m, 1H), 1.96 – 1.83 (m, 1H), 0.36 (s, 3H).

^{13}C NMR (101 MHz, $CDCl_3$): δ 164.5, 143.9, 142.7, 139.4, 137.6, 136.4, 135.1, 131.4, 129.7, 128.5, 128.0, 127.8, 127.1, 125.7, 124.3, 120.6, 113.7, 109.7, 55.1, 21.8, -4.7.

HRMS (EI): Calculated for $C_{25}H_{26}OSi$ $[M]^+$: 370.1753. Found: 370.1746.

HPLC: Phenomenex lux cellulose-3 column, CH_3CN/H_2O = 55/45, 1.0 mL/min, 230 nm UV detector, t (major) = 21.4 min, t (minor) = 24.7 min.

(*S*, *E*)-allyl(1,2-diphenylvinyl)(3-methoxyphenyl)(methyl)silane (4fa):

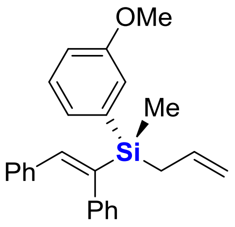

Following the general procedure, the reaction was carried out with diphenylacetylene (0.2 mmol), silacyclobutane **1o** (0.4 mmol), Ni(cod)$_2$ (5.8 mg, 0.02 mmol), **L6** (26.0 mg, 0.04 mmol) and 2-MeTHF (1.0 mL) at 60 ℃ for 48 h. The title compound was obtained in 90% yield (75.2 mg) as colorless oil, e.e. = 79%; Optical rotation: $[\alpha]_D^{25}$ 4.8 (c = 2.50, $CHCl_3$).

^{1}H NMR (400 MHz, Chloroform-*d*): δ 7.34 (t, *J* = 7.7 Hz, 1H), 7.31 – 7.25 (m, 2H), 7.24 – 7.15 (m, 2H), 7.15 – 7.09 (m, 4H), 6.98 (m, 5H), 6.91 (s, 1H), 5.79 (m, 1H), 4.97 – 4.85 (m, 2H), 3.83 (s, 3H), 2.00 – 1.90 (m, 2H), 0.43 (s, 3H).

^{13}C NMR (101 MHz, Chloroform-*d*): δ 159.0, 143.3, 142.2, 140.3, 138.0, 137.2, 134.2, 129.7, 129.1, 128.7, 128.0, 127.9, 127.4, 127.0, 126.0, 120.1, 114.7, 114.4, 55.2, 21.4, -5.2.

HRMS (EI): Calculated for $C_{25}H_{26}OSi$ $[M]^+$: 370.1753. Found: 370.1745.

HPLC: Daicel chiralpak OD-3 column, *n*-hexane/*i*-PrOH = 99/1, 0.5 mL/min, 254 nm UV detector, t (major) = 9.6 min, t (minor) = 10.6 min.

(*S*, *E*)-allyl(1,2-diphenylvinyl)(3-methoxyphenyl)(methyl)silane (4ga):

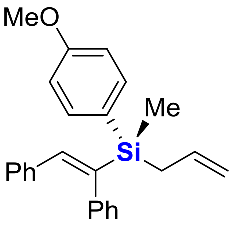

Following the general procedure, the reaction was carried out with diphenylacetylene **1a** (0.2 mmol), silacyclobutane **1e** (0.4 mmol), Ni(cod)$_2$ (5.8 mg, 0.02 mmol), **L6** (52 mg, 0.04 mmol) and 2-MeTHF (1.5 mL) at 60 ℃ for 24 h. The title compound was obtained in 80% yield (59.5 mg) as colorless oil, e.e. = 78%; Optical rotation: $[\alpha]_D^{25}$ 5.2 (c = 2.0, $CHCl_3$).

^{1}H NMR (400 MHz, Chloroform-*d*): δ 7.66 – 7.60 (m, 2H), 7.43 – 7.37 (m, 2H), 7.36 – 7.30 (m, 1H), 7.27 – 7.20 (m, 3H), 7.09 (m, 6H), 7.00 (s, 1H), 5.99 – 5.83 (m, 1H), 5.08 – 4.96 (m, 2H), 3.99 (s, 3H), 2.06 (m, 2H), 0.52 (s, 3H).

^{13}C NMR (101 MHz, Chloroform-*d*): δ 160.7, 143.9, 140.0, 137.3, 136.2, 134.4, 129.7, 129.7, 128.7, 128.0, 127.8, 127.4, 127.0, 125.9, 114.2, 113.7, 55.2, 21.6, -5.1.

HRMS (EI): Calculated for $C_{25}H_{26}OSi$ $[M]^+$: 370.1753. Found: 370.1746.

HPLC: Daicel chiralpak OD-3 column, *n*-hexane/*i*-PrOH = 99.9/0.1, 0.5 mL/min, 254 nm UV detector, t (minor) = 24.8 min, t (major) = 26.5 min.

(*R*,*E*)-allyl(1,2-diphenylvinyl)(methyl)(naphthalen-1-yl)silane (4ha):

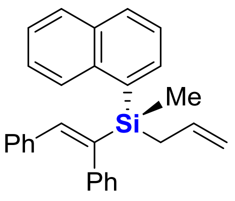

Following the general procedure, the reaction was carried out with diphenylacetylene (0.2 mmol), silacyclobutane **1p** (0.4 mmol), Ni(cod)$_2$ (5.8 mg, 0.02 mmol), (*R*,*R*)-**L20** (27.0 mg, 0.04 mmol) and 2-MeTHF (1.0 mL) at 60 ℃ for 24 h. The title compound

was obtained in 45% yield (35.2 mg) as colorless oil, ee = 91%, Optical rotation: $[\alpha]_D^{25}$ 14.4 (c = 0.60, $CHCl_3$).

^{1}H NMR (400 MHz, $CDCl_3$): δ 8.26 – 8.16 (m, 1H), 7.86 – 7.76 (m, 2H), 7.63 (dd, *J* = 6.9, 1.3 Hz, 1H), 7.45 – 7.33 (m, 3H), 7.14 – 7.05 (m, 3H), 7.02 – 6.92 (m, 3H), 6.88 – 6.77 (m, 5H), 5.70 – 5.57 (m, 1H), 4.83 – 4.70 (m, 2H), 2.12 – 2.03 (m, 1H), 2.02 – 1.91 (m, 1H), 0.46 (s, 3H).

^{13}C NMR (101 MHz, $CDCl_3$): δ 144.0, 142.1, 140.5, 137.3, 137.2, 135.2, 134.3, 134.0, 133.6, 130.5, 129.7, 129.2, 128.7, 128.6, 128.0, 127.8, 127.4, 126.03, 125.95, 125.6, 125.2, 114.5, 22.1, -3.7.

HRMS (ESI): Calculated for $[C_{28}H_{26}Si, M+Na]^+$: 413.1696. Found: 413.1696.

HPLC: Phenomenex lux cellulose-3 column, MeCN/H_2O = 70/30, 1.0 mL/min, 230 nm UV detector, t (major) = 18.5 min, t (minor) = 20.8 min.

(*R*,*E*)-allyl(1,2-diphenylvinyl)(4-methoxynaphthalen-1-yl)(methyl)silane (4ia):

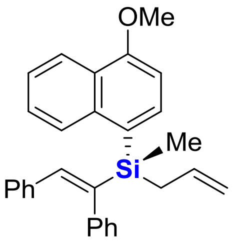

Following the general procedure, the reaction was carried out with diphenylacetylene (0.2 mmol), silacyclobutane **1q** (0.4 mmol), Ni(cod)$_2$ (5.8 mg, 0.02 mmol), (*R*,*R*)-**L20** (27.0 mg, 0.04 mmol) and 2-MeTHF (1.0 mL) at 60 ℃ for 24 h. The title compound was obtained in 40% yield (33.6 mg) as colorless oil, ee = 93%, Optical rotation: $[\alpha]_D^{25}$ 15.5 (c = 1.0, $CHCl_3$).

^{1}H NMR (400 MHz, $CDCl_3$): δ 8.39 – 8.34 (m, 1H), 8.28 – 8.22 (m, 1H), 7.64 (d, *J* = 7.7 Hz, 1H), 7.53 – 7.46 (m, 2H), 7.23 – 7.13 (m, 3H), 7.10 – 7.04 (m, 3H), 6.96 – 6.88 (m, 5H), 6.83 (d, *J* = 7.7 Hz, 1H), 5.79 – 5.66 (m, 1H), 4.94 – 4.76 (m, 2H), 4.03 (s, 3H), 2.20 – 2.09 (m, 1H), 2.07 – 1.98 (m, 1H), 0.52 (s, 3H).

^{13}C NMR (101 MHz, $CDCl_3$): δ 157.3, 144.5, 142.3, 140.3, 138.3, 137.4, 135.8, 134.6, 129.7, 128.7, 128.3, 128.0, 127.8, 127.3, 126.5, 126.0, 125.9, 125.0, 124.6, 122.7, 114.3, 103.5, 55.5, 22.2, -3.7.

HRMS (ESI): Calculated for $[C_{29}H_{28}OSi, M+Na]^+$: 443.1802. Found: 443.1800.

HPLC: Phenomenex lux cellulose-3 column, MeCN/H_2O = 55/45, 1.0 mL/min, 230 nm UV detector, t (major) = 41.5 min, t (minor) = 43.9 min.

(*S*,*E*)-allyl(1,2-diphenylvinyl)(ethyl)(phenyl)silane (4ja):

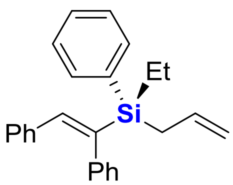

Following the general procedure, the reaction was carried out with diphenylacetylene (0.2 mmol), silacyclobutane **1r** (0.4 mmol), Ni(cod)$_2$ (5.8 mg, 0.02 mmol), (*R*,*R*)-**L20** (27.0 mg, 0.04 mmol) and 2-MeTHF (1.0 mL) at 60 ℃ for 24 h. The title compound was obtained in 85% yield (60.3 mg) as colorless oil. The ee value of **4ja** was measured by chiral HPLC on Daicel OD-3 column with the corresponding alcohol derivative (80% ee). Optical rotation: $[\alpha]_D^{25}$ 1.0 (c = 0.75, $CHCl_3$).

^{1}H NMR (400 MHz, $CDCl_3$): δ 7.91 – 7.82 (m, 2H), 7.72 – 7.61 (m, 3H), 7.56 – 7.49 (m, 2H), 7.48 – 7.42 (m, 1H), 7.41 – 7.33 (m, 3H), 7.28 – 7.20 (m, 4H), 7.17 (d, *J* = 1.9 Hz, 1H), 6.13 – 5.97 (m, 1H), 5.25 – 5.10 (m, 2H), 2.31 – 2.18 (m, 2H), 1.33 – 1.20 (m, 5H).

^{13}C NMR (101 MHz, CDCl$_3$): δ 142.5, 142.4 140.9, 137.2, 135.6, 135.0, 134.2, 129.8, 129.3, 128.7, 128.0, 127.9, 127.8, 127.4, 126.0, 19.5, 7.3, 3.3.

HRMS (ESI): Calculated for [$C_{25}H_{26}Si$, M+Na]$^+$: 377.1696. Found: 377.1697.

(*S*,*E*)-3-((1,2-diphenylvinyl)(ethyl)(phenyl)silyl)propan-1-ol (4ja-OH):

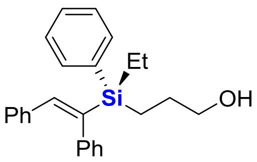

The title compound was obtained as colorless oil after oxidation of **4ja**.

^{1}H NMR (400 MHz, CDCl$_3$): δ 7.48 (dd, *J* = 7.5, 2.0 Hz, 2H), 7.34 – 7.25 (m, 3H), 7.19 – 7.08 (m, 3H), 7.03 – 6.97 (m, 3H), 6.91 – 6.82 (m, 4H), 6.79 (s, 1H), 3.48 (t, *J* = 6.7 Hz, 2H), 1.52 – 1.45 (m, 2H), 0.95 – 0.80 (m, 7H).

^{13}C NMR (101 MHz, CDCl$_3$): δ 142.8, 142.5, 140.6, 137.2, 135.9, 134.9, 129.8, 129.3, 128.7, 128.0, 128.0, 127.7, 127.4, 125.9, 65.8, 27.0, 7.4, 7.1, 3.6.

HRMS (ESI): Calculated for [$C_{25}H_{28}OSi$, M+Na]$^+$: 395.1801. Found: 395.1806.

HPLC: Daicel chiralpak OD-3 column, *n*-hexane/*i*-PrOH = 90/10, 0.5 mL/min, 254 nm UV detector, t (minor) = 12.0 min, t (major) = 13.0 min, ee = 80%.

(*R*,*E*)-allyl(1,2-diphenylvinyl)(isopropyl)(phenyl)silane (4ka):

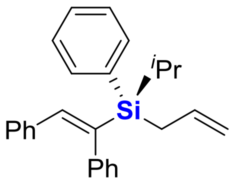

Following the general procedure, the reaction was carried out with diphenylacetylene (0.2 mmol), silacyclobutane **1s** (0.4 mmol), Ni(cod)$_2$ (5.8 mg, 0.02 mmol), (*R*,*R*)-**L20** (27.0 mg, 0.04 mmol) and 2-MeTHF (1.0 mL) at 60 ℃ for 24 h. The title compound was obtained in 80% yield (59.0 mg) as colorless oil. The ee value of **4ka** was measured by chiral HPLC on Daicel OD-3 column with the corresponding alcohol derivative (83% ee). Optical rotation: $[\alpha]_D^{25}$ -29.3 (c = 0.34, CHCl$_3$).

1H NMR (400 MHz, CDCl3): δ 7.50 – 7.44 (m, 2H), 7.43 – 7.37 (m, 1H), 7.29 – 7.18 (m, 5H), 7.13 – 7.07 (m, 2H), 7.06 – 7.00 (m, 1H), 6.99 – 6.90 (m, 3H), 6.86 (dd, J = 7.2, 2.0 Hz, 2H), 6.83 – 6.75 (m, 3H), 5.71 – 5.56 (m, 1H), 4.84 – 4.63 (m, 2H), 1.90 – 1.75 (m, 2H), 1.24 – 1.15 (m, 1H), 0.92 – 0.84 (m, 6H).

^{13}C NMR (101 MHz, CDCl$_3$): δ 142.6, 141.8, 141.7, 137.3, 135.6, 134.9, 134.6, 131.8, 129.8, 129.3, 128.7, 128.5, 128.4, 128.0, 127.8, 127.4, 126.0, 114.5, 29.9, 19.2, 18.0, 17.9, 11.6.

HRMS (ESI): Calculated for [$C_{26}H_{28}Si$, M+Na]$^+$: 391.1852. Found: 391.1852.

(*R*,*E*)-3-((1,2-diphenylvinyl)(isopropyl)(phenyl)silyl)propan-1-ol (4ka-OH):

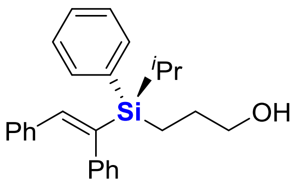

The title compound was obtained as colorless oil after oxidation of **4ka**.

^{1}H NMR (400 MHz, CDCl$_3$): δ 7.56 – 7.49 (m, 2H), 7.34 – 7.26 (m, 3H), 7.19 – 7.13 (m, 2H), 7.12 – 7.06 (m, 1H), 7.04 – 6.97 (m, 3H), 6.95 – 6.90 (m, 2H), 6.89 – 6.85 (m, 2H), 6.83 (d, *J* = 1.5 Hz, 1H), 3.45 (t, *J* = 6.6 Hz, 2H), 1.54 – 1.40 (m, 2H), 1.28 – 1.15 (m, 2H), 0.97 – 0.90 (m, 6H), 0.89 – 0.82 (m, 2H).

^{13}C NMR (101 MHz, CDCl$_3$): δ 142.8, 141.9, 141.6, 137.3, 135.5, 135.0, 129.8, 129.2, 128.7, 128.0, 127.9, 127.9, 127.4, 126.0, 65.8, 27.2, 18.2, 18.1, 11.7, 6.7.

HRMS (ESI): Calculated for [$C_{26}H_{30}OSi$, M+Na]$^+$: 409.1958. Found: 409.1953.

HPLC: Daicel chiralpak OD-3 column, *n*-hexane/*i*-PrOH = 96/4, 0.5 mL/min, 254 nm UV detector, t (minor) = 19.9 min, t (major) = 21.5 min, ee = 83%.

(*R*,*E*)-allyl(cyclopropyl)(1,2-diphenylvinyl)(phenyl)silane (4la):

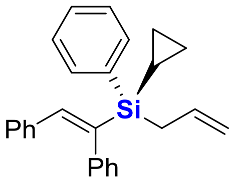

Following the general procedure, the reaction was carried out with diphenylacetylene (0.2 mmol), silacyclobutane **1t** (0.4 mmol), Ni(cod)$_2$ (5.8 mg, 0.02 mmol), (*R*,*R*)-**L20** (27.0 mg, 0.04 mmol) and 2-MeTHF (1.0 mL) at 60 ℃ for 24 h. The title compound was obtained in 55% yield (40.5 mg) as colorless oil. The ee value of **4la** was measured by chiral HPLC on Daicel OD-3 column with the corresponding alcohol derivative (85% ee). Optical rotation: $[\alpha]_D^{25}$ -31.9 (c = 0.67, $CHCl_3$).

^{1}H NMR (400 MHz, $CDCl_3$): δ 7.74 – 7.66 (m, 2H), 7.49 – 7.38 (m, 3H), 7.33 – 7.20 (m, 3H), 7.20 – 7.15 (m, 3H), 7.14 (s, 1H), 7.10 – 7.03 (m, 4H), 5.91 – 5.78 (m, 1H), 5.00 – 4.87 (m, 2H), 2.03 – 1.95 (m, 1H), 1.93 – 1.83 (m, 1H), 0.81 – 0.68 (m, 2H), 0.45 – 0.36 (m, 1H), 0.34 – 0.24 (m, 1H), 0.06 – -0.06 (m, 1H).

^{13}C NMR (101 MHz, $CDCl_3$): δ 142.4, 141.6, 137.3, 135.4, 134.7, 134.3, 129.8, 129.5, 128.6, 128.0, 127.8, 127.4, 126.0, 114.4, 19.7, 2.0, 1.6, -7.2.

HRMS (ESI): Calculated for [$C_{26}H_{26}F_2Si$, M+Na]$^+$: 389.1696. Found: 389.1696.

(*R*,*E*)-3-(cyclopropyl(1,2-diphenylvinyl)(phenyl)silyl)propan-1-ol (4la-OH):

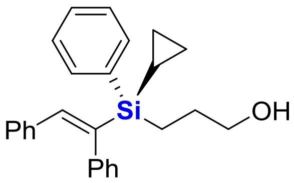

The title compound was obtained as colorless oil after oxidation of **4la**.

^{1}H NMR (400 MHz, $CDCl_3$): δ 7.65 – 7.59 (m, 2H), 7.41 – 7.33 (m, 3H), 7.22 (dd, *J* = 8.1, 6.3 Hz, 2H), 7.19 – 7.15 (m, 1H), 7.13 – 7.08 (m, 3H), 7.07 (s, 1H), 7.03 – 6.96 (m, 4H), 3.53 (t, *J* = 6.6 Hz, 2H), 1.65 – 1.56 (m, 2H), 0.94 – 0.84 (m, 1H), 0.82 – 0.60 (m, 3H), 0.35 – 0.27 (m, 1H), 0.22 – 0.15 (m, 1H), -0.03 – -0.14 (m, 1H).

^{13}C NMR (101 MHz, $CDCl_3$): δ 142.6, 142.6, 141.4, 137.3, 135.4, 134.9, 129.8, 129.4, 128.6, 128.0, 127.9, 127.8, 127.4, 126.0, 65.8, 27.2, 7.3, 2.1, 1.5, -7.2.

HRMS (ESI): Calculated for [$C_{26}H_{28}OSi$, M+Na]$^+$: 407.1801. Found: 407.1802.

HPLC: Daicel chiralpak OD-3 column, *n*-hexane/*i*-PrOH = 96/4, 0.5 mL/min, 254 nm UV detector, t (minor) = 23.4 min, t (major) = 25.5 min, ee = 85%

(*R*,*E*)-allyl(1,2-diphenylvinyl)(isopropyl)(3-methoxyphenyl)silane (4ma):

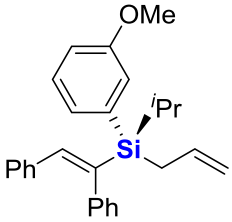

Following the general procedure, the reaction was carried out with diphenylacetylene (0.2 mmol), silacyclobutane **1n** (0.4 mmol), Ni(cod)$_2$ (5.8 mg, 0.02 mmol), (*R*,*R*)-**L20** (27.0 mg, 0.04 mmol) and 2-MeTHF (1.0 mL) at 60 ℃ for 24 h. The title compound was obtained in 80% yield (63.8 mg) as colorless oil. The ee value of **4fa** was measured by chiral HPLC on Daicel OD-3 column with the corresponding alcohol derivative **(**83% ee). Optical rotation: $[\alpha]_D^{25}$ -33.9 (c = 1.00, $CHCl_3$).

^{1}H NMR (400 MHz, CDCl$_3$): δ 7.26 – 7.20 (m, 1H), 7.18 – 7.09 (m, 4H), 7.06 – 7.03 (m, 1H), 7.03 – 6.98 (m, 3H), 6.95 – 6.90 (m, 2H), 6.89 – 6.82 (m, 4H), 5.78 – 5.62 (m, 1H), 4.89 – 4.74 (m, 2H), 1.93 – 1.86 (m, 2H), 1.30 – 1.20 (m, 1H), 0.99 – 0.90 (m, 6H).

^{13}C NMR (101 MHz, CDCl$_3$): δ 158.9, 142.6, 141.8, 141.6, 137.3, 136.6, 134.6, 129.8, 129.0, 128.7, 128.1, 128.0, 127.9, 127.4, 126.0, 121.0, 114.6, 114.5, 55.2, 29.8, 19.2, 18.05, 17.96, 11.6.

HRMS (EI): Calculated for [$C_{27}H_{30}OSi$, M+Na]$^+$: 421.1958. Found: 421.1957.

(*R*,*E*)-3-((1,2-diphenylvinyl)(isopropyl)(3-methoxyphenyl)silyl)propan-1-ol (4ma-OH):

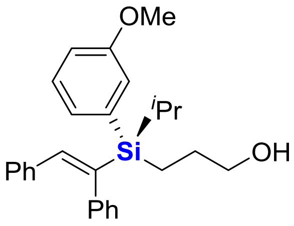

The title compound was obtained as colorless oil after oxidation of **4ma**.

^{1}H NMR (400 MHz, CDCl$_3$): δ 7.37 – 7.32 (m, 1H), 7.29 – 7.24 (m, 2H), 7.23 – 7.19 (m, 2H), 7.16 – 7.14 (m, 1H), 7.13 – 7.10 (m, 3H), 7.06 – 7.02 (m, 2H), 6.99 – 6.94 (m, 4H), 3.82 (s, 3H), 3.56 (t, *J* = 6.6 Hz, 2H), 1.62 – 1.55 (m, 2H), 1.37 – 1.31 (m, 1H), 1.07 – 1.02 (m, 6H), 0.99 – 0.94 (m, 2H).

^{13}C NMR (101 MHz, CDCl$_3$): δ 159.0, 142.8, 141.8, 141.7, 137.3, 136.7, 129.8, 129.0, 128.7, 128.03, 127.95, 127.8, 127.4, 126.0, 121.1, 114.4, 65.9, 55.2, 27.3, 18.2, 18.1, 11.8, 6.8.

HRMS (EI): Calculated for [$C_{27}H_{32}O_2Si$, M+Na]$^+$: 439.2064. Found: 439.2062.

HPLC: Daicel chiralpak OD-3 column, *n*-hexane/*i*-PrOH = 90/10, 0.5 mL/min, 254 nm UV detector, t (minor) = 12.4 min, t (major) = 15.0 min, ee = 83%.

(*R*,*E*)-allyl(1,2-diphenylvinyl)(4-fluorophenyl)(isopropyl)silane (4na):

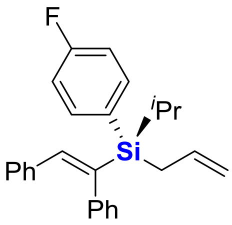

Following the general procedure, the reaction was carried out with diphenylacetylene (0.2 mmol), silacyclobutane **1o** (0.4 mmol), Ni(cod)$_2$ (5.8 mg, 0.02 mmol), (*R*,*R*)-**L20** (27.0 mg, 0.04 mmol) and 2-MeTHF (1.0 mL) at 60 ℃ for 24 h. The title compound was obtained in 75% yield (58.0 mg) as colorless oil. The ee value of **4ga** was measured by chiral HPLC on Daicel OD-3 column with the corresponding alcohol derivative (80% ee). Optical rotation: $[\alpha]_D^{25}$ -26.6 (c = 0.89, CHCl$_3$).

^{1}H NMR (400 MHz, CDCl$_3$): δ 7.75 – 7.69 (m, 2H), 7.42 – 7.36 (m, 2H), 7.36 – 7.30 (m, 1H), 7.27 – 7.18 (m, 5H), 7.15 – 7.08 (m, 4H), 7.05 (s, 1H), 5.98 – 5.84 (m, 1H), 5.12 – 4.97 (m, 2H), 2.15 – 2.08 (m, 2H), 1.51 – 1.44 (m, 1H), 1.20 – 1.14 (m, 6H).

^{13}C NMR (101 MHz, CDCl$_3$): δ 163.9 (d, *J* = 248.6 Hz), 142.4, 141.9, 141.5, 137.4 (d, *J* = 7.5 Hz), 137.2, 134.3, 130.3 (d, *J* = 4.1 Hz), 129.8, 128.8, 128.0 (d, *J* = 10.0 Hz), 127.5, 126.1, 115.0 (d, *J* = 19.6 Hz), 114.7, 29.9, 19.2, 18.0, 17.9, 11.6.

^{19}F NMR (376 MHz, CDCl$_3$): δ -111.6.

HRMS (EI): Calculated for [$C_{26}H_{27}FSi$, M+Na]$^+$: 409.1758. Found: 409.1753.

(*R*,*E*)-3-((1,2-diphenylvinyl)(4-fluorophenyl)(isopropyl)silyl)propan-1-ol (4na-OH):

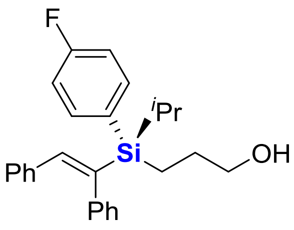

The title compound was obtained as colorless oil after oxidation of **4na**.

^{1}H NMR (400 MHz, CDCl$_3$): δ 7.62 – 7.55 (m, 2H), 7.30 – 7.24 (m, 2H), 7.23 – 7.18 (m, 1H), 7.14 – 7.06 (m, 5H), 7.02 – 6.94 (m, 4H), 6.91 (s, 1H), 3.57 (t, *J* = 6.6 Hz, 2H), 1.63 – 1.56 (m, 2H), 1.35 – 1.30 (m, 1H), 1.03 (t, *J* = 7.5 Hz, 6H), 0.99 – 0.94 (m, 2H).

^{13}C NMR (101 MHz, CDCl$_3$): δ 163.9 (d, *J* = 248.4 Hz), 142.6, 141.8, 141.7, 137.4 (d, *J* = 7.4 Hz), 137.2, 130.4 (d, *J* = 4.3 Hz), 129.8, 128.8, 128.1, 127.9, 127.5, 126.1, 115.1 (d, *J* = 19.3 Hz), 65.8, 27.2, 18.1, 18.0, 11.7, 6.8.

^{19}F NMR (376 MHz, CDCl$_3$): δ -111.7.

HRMS (EI): Calculated for [$C_{26}H_{29}FOSi$, M+Na]$^+$: 427.1864. Found: 427.1863.

HPLC: Daicel chiralpak OD-3 column, *n*-hexane/*i*-PrOH = 97/3, 0.5 mL/min, 254 nm UV detector, t (minor) = 21.4 min, t (major) = 22.7 min, ee = 80%.

(*R*,*E*)-allyl(1,2-diphenylvinyl)(isopropyl)(4-methoxyphenyl)silane (4oa):

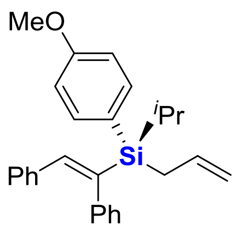

Following the general procedure, the reaction was carried out with diphenylacetylene (0.2 mmol), silacyclobutane **1p** (0.4 mmol), Ni(cod)$_2$ (5.8 mg, 0.02 mmol), (*R*,*R*)-**L20** (27.0 mg, 0.04 mmol) and 2-MeTHF (1.0 mL) at 60 ℃ for 24 h. The title compound was obtained in 35% yield (28.0 mg) as colorless oil. The ee value of **4ha** was measured by chiral HPLC on Daicel OD-3 column with the corresponding alcohol derivative (84% ee). Optical rotation: $[\alpha]_D^{25}$ -43.5 (c = 0.23, CHCl$_3$).

^{1}H NMR (400 MHz, CDCl$_3$): δ 7.71 – 7.65 (m, 2H), 7.42 – 7.36 (m, 2H), 7.35 – 7.31 (m, 1H), 7.27 – 7.21 (m, 3H), 7.17 – 7.12 (m, 2H), 7.11 – 7.06 (m, 4H), 7.05 (s, 1H), 5.99 – 5.86 (m, 1H), 5.10 – 4.95 (m, 2H), 3.99 (s, 3H), 2.14 – 2.06 (m, 2H), 1.48 – 1.43 (m, 1H), 1.21 – 1.12 (m, 6H).

^{13}C NMR (101 MHz, CDCl$_3$): δ 160.5, 142.6, 142.0, 141.4, 137.2, 136.9, 134.6, 129.6, 128.6, 127.9, 127.2, 125.8, 125.3, 114.2, 113.5, 55.0, 29.7, 19.2, 17.9, 17.8, 11.5.

HRMS (EI): Calculated for [$C_{27}H_{30}OSi$, M+Na]$^+$: 421.1958. Found: 421.1958.

(*R*,*E*)-3-((1,2-diphenylvinyl)(isopropyl)(4-methoxyphenyl)silyl)propan-1-ol (4oa-OH):

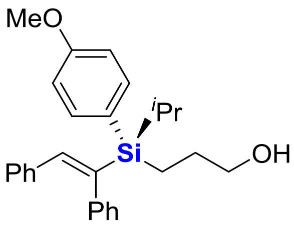

The title compound was obtained as colorless oil after oxidation of **4oa**.

^{1}H NMR (400 MHz, CDCl$_3$): δ 7.65 – 7.59 (m, 2H), 7.37 – 7.32 (m, 2H), 7.31 – 7.27 (m, 1H), 7.21 – 7.16 (m, 3H), 7.13 – 7.08 (m, 2H), 7.07 – 7.01 (m, 4H), 7.00 (s, 1H), 3.94 (s, 3H), 3.64 (t, *J* = 6.6 Hz, 2H), 1.72 – 1.64 (m, 2H), 1.42 – 1.38 (m, 1H), 1.14 – 1.08 (m, 6H), 1.04 – 0.99 (m, 2H).

^{13}C NMR (101 MHz, CDCl$_3$): δ 160.6, 142.9, 142.3, 141.4, 137.4, 137.0, 129. 8, 128.7, 128.0, 127.9, 127.3, 125.9, 125.6, 113.7, 65.9, 55.1, 29.8, 27.3, 18.2, 18.1, 11.8, 6.9.

HRMS (EI): Calculated for [$C_{27}H_{32}O_2Si$, M+Na]$^+$: 439.2064. Found:.

HPLC: Daicel chiralpak OD-3 column, *n*-hexane/*i*-PrOH = 92/8, 0.5 mL/min, 254 nm UV detector, t (major) = 12.7 min, t (minor) = 13.5 min, ee = 84%.

Nickel-catalyzed cycloaddition of benzosilacyclobutanes with alkynes

Optimization of the reaction conditions

Supplementary Table 2. Optimization of the reaction conditions [a]

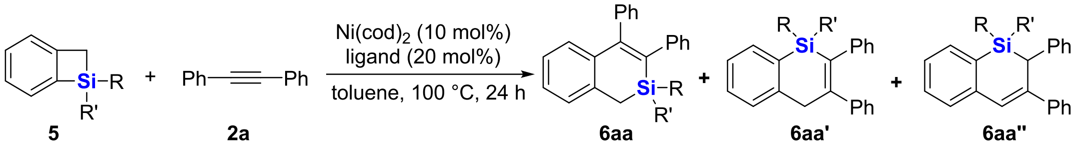

Entry	R	Ligand	Solvent	Conv. of 1a (%)[b]	6aa (%)[b]	6aa′ (%)[b]	6aa″ (%)[b]
1	Me	PPh_3	toluene	100	74	18	8
2	Me	P(*o*-Me-C_6H_4)$_3$	toluene	100	81	13	6
3	Me	P(*p*-Me-C_6H_4)$_3$	toluene	100	76	15	9
4	Me	P(*o*-OMe-C_6H_4)$_3$	toluene	100	68	26	6
5	Me	P(*p*-OMe-C_6H_4)$_3$	toluene	100	79	13	8
6	Me	P(*p*-F-C_6H_4)$_3$	toluene	100	66	25	9
7	Me	P(*p*-CF_3-C_6H_4)$_3$	toluene	100	52	36	12
8	Me	PMe_3	toluene	100	90	10	0
9	Me	PEt_3	toluene	100	87	0	13
10	Me	P^nPr_3	toluene	100	86	0	14
11	Me	P^nBu_3	toluene	100	86	0	14
12	Me	P^tBu_3	toluene	100	86	2	12
13	Me	PCy_3	toluene	100	63	0	37
14	Me	$PAd_2(^nBu)$	toluene	100	54	0	46
15[c]	Me	IMes·HCl	toluene	100	56	0	44
16[c]	Me	IPr·HCl	toluene	100	55	0	45
17	Me	PMe_3	THF	100	90	7	3
18	Me	PMe_3	2-MeTHF	32	27	5	0
**19**	**Me**	**PMe_3**	**1,4-dioxane**	**100**	**95 (86)**	**0**	**5**
20	Me	PMe_3	$PhCF_3$	100	92	8	0
21	Me	PMe_3	*o*-xylene	100	89	11	0
22	Me	PMe_3	*p*-xylene	100	93	7	-
23	Me	PMe_3	Mesitylene	100	91	9	0
24	Me	PMe_3	DME	94	82	12	0
25	Me	PMe_3	DMF	0	-	-	-
26	Me	PMe_3	*n*-heptane	86	78	8	0
27	Ph	PMe_3	toluene	100	93	5	2
**28**	**Ph**	**PAd_3**	**toluene**	**100**	**97 (90)**	**<1**	**3**

[a] Unless otherwise noted, all reactions were carried out with Ni(cod)$_2$ (0.01 mmol, 10 mol%), ligand (0.02 mmol, 20 mol%), diphenylacetylene **2a** (0.1 mmol) and **5** (0.15 mmol, 1.5 equiv.) in 1.0 mL solvent at 100 ℃ for 24 h. [b] the ratios were determined by GC-MS, the values in parentheses indicated the isolated yield. [c] 20 mol% LiO^{t}Bu as additive.

General procedure

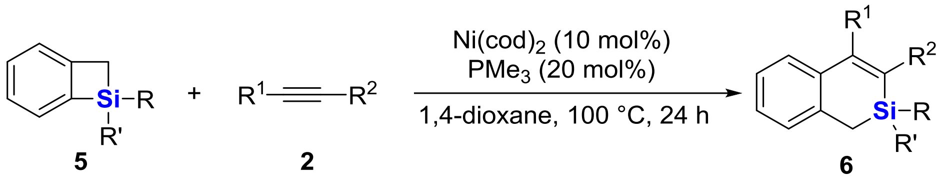

Procedure A: In a nitrogen-filled glove-box, an oven-dried 8 mL glass vial equipped with a magnetic stir bar was charged with $Ni(cod)_2$ (5.8 mg, 0.02 mmol), PMe_3 (40μL, 1.0 M in THF, 0.04 mmol) and 1,4-dioxane (1.0 mL). The mixture was allowed to stir under ambient temperature for 10 min. Then alkynes **2** (0.2 mmol) and benzosilacyclobutanes **5** (0.3 mmol, 1.5 equiv.) were added sequentially. The vial was sealed with a PTFE cap, removed from the glove box and stirred at 100 ℃ for 24 h. After being cooled to room temperature, the solvent was removed under vacuum and the residue was purified on C18(ODS) column (5μm, 21.2x250 mm) with acetonitrile by preparative RP-HPLC with an Bonna-Agela CHEETAH HP series.

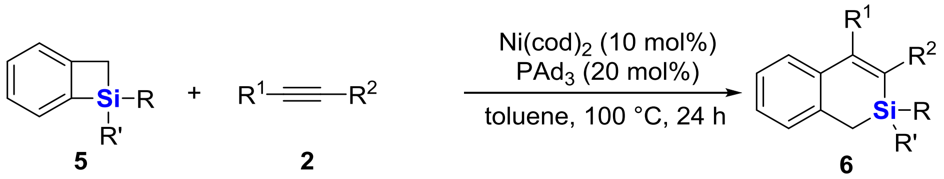

Procedure B: In a nitrogen-filled glove-box, an oven-dried 8 mL glass vial equipped with a magnetic stir bar was charged with $Ni(cod)_2$ (5.8 mg, 0.02 mmol), PAd_3 (17.6 mg, 0.04 mmol) and toluene (1.0 mL). The mixture was allowed to stir under ambient temperature for 10 min. Then alkynes **2** (0.2 mmol) and benzosilacyclobutanes **5** (0.3 mmol, 1.5 equiv.) were added sequentially. The vial was sealed with a PTFE cap, removed from the glove box and stirred at 100 ℃ for 24 h. After being cooled to room temperature, the solvent was removed under vacuum and the residue was purified on C18(ODS) column (5μm, 21.2x250 mm) with acetonitrile by preparative RP-HPLC with an Bonna-Agela CHEETAH HP series.

Characterization of products 6

2,2-Dimethyl-3,4-diphenyl-1,2-dihydrobenzo[*c*]saline (6aa):

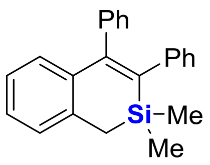

Following procedure A, the reaction was carried out with diphenylacetylene (0.2 mmol), benzosilacyclobutene **5a** (0.3 mmol), $Ni(cod)_2$ (5.8 mg, 0.02 mmol), PMe_3 (40μL, 1.0 M in THF, 0.04 mmol) and 1,4-dioxane (1.0 mL) at 100 ℃ for 24 h. The title compound was obtained in 86% yield (56.0 mg) as colorless oil.

^{1}H NMR (400 MHz, $CDCl_3$): δ 7.21 (dd, J = 7.5, 1.4 Hz, 1H), 7.17 – 7.05 (m, 6H), 6.94 - 7.02 (m, 4H), 6.78 (m, 3H), 2.31 (s, 2H), 0.07 (s, 6H).

^{13}C NMR (101 MHz, $CDCl_3$): δ 150.4, 143.0, 141.8, 140.6, 138.9, 136.3, 131.0, 130.6, 130.1, 128.0, 127.7, 127.5, 127.1, 126.2, 125.1, 124.7, 77.3, 77.2, 77.0, 76.7, 21.3, -3.7.

HRMS (EI): Calculated for $C_{23}H_{22}Si$ $[M]^+$: 326.1491. Found: 326.1483.

1,1-Dimethyl-2,3-diphenyl-1,2-dihydrobenzo[b]saline (6aa″):

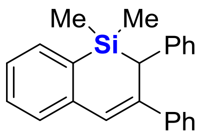

The title compound was obtained as colorless oil.

^{1}H NMR (400 MHz, CDCl$_3$): δ 7.44 – 7.39 (m, 2H), 7.38 – 7.29 (m, 2H), 7.28 – 7.14 (m, 5H), 7.13 – 7.07 (m, 2H), 7.04 (s, 1H), 7.02 – 6.96 (m, 3H), 3.33 (s, 1H), 0.24 (s, 3H), 0.00 (s, 3H).

^{13}C NMR (101 MHz, CDCl$_3$): δ 144.8, 142.4, 140.7, 140.0, 133.6, 130.7, 130.6, 130.2, 128.9, 128.6, 128.4, 127.4, 127.3, 127.0, 126.2, 124.8, 37.8, -1.7, -5.9.

HRMS (EI): Calculated for $C_{23}H_{22}Si$ $[M]^+$: 326.1491. Found: 326.1483.

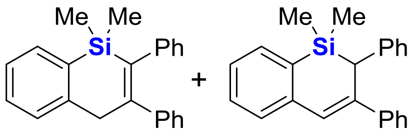

6aa' + **6aa"**

The mixture of **6aa′** and **6aa″** (2:1) were obtained as colorless oil.

^{1}H NMR (400 MHz, CDCl$_3$): δ 7.58 (dd, *J* = 7.1, 1.6 Hz, 1H), 7.45 – 7.41 (m, 2H), 7.41 – 7.16 (m, 11H), 7.15 – 6.95 (m, 14H), 6.88 – 6.82 (m, 2H), 4.04 (**6aa′,** –CH$_2$–, s, 2H), 3.35 (**6aa″,** –CH$_2$–, s, 1H), 0.28 (s, 6H), 0.26 (s, 3H), 0.02 (s, 3H).

^{13}C NMR (101 MHz, CDCl$_3$): δ 150.7, 144.9, 144.8, 144.0, 142.7, 142.4, 140.7, 140.1, 137.9, 134.6, 133.6, 133.3, 130.7, 130.6, 130.2, 129.1, 128.9, 128.7, 128.6, 128.4, 128.2, 127.8, 127.8, 127.4, 127.3, 127.0 126.4, 126.2, 125.9, 125.0, 124.8, 42.8, 37.8, -1.7, -1.9, -5.9.

HRMS (EI) of **6aa′**: Calculated for $C_{23}H_{22}Si$ $[M]^+$: 326.1491. Found: 326.1486.

3,4-Bis(4-fluorophenyl)-2,2-dimethyl-1,2-dihydrobenzo[c]siline (6ab):

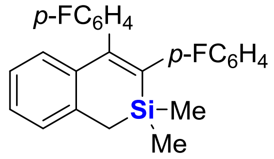

Following procedure A, the reaction was carried out with alkyne **2f** (0.2 mmol), benzosilacyclobutene **5a** (0.3 mmol), Ni(cod)$_2$ (5.8 mg, 0.02 mmol), PMe$_3$ (40μL, 1.0 M in THF, 0.04 mmol) and 1,4-dioxane (1.0 mL) at 100 ℃ for 24 h. The title compound was obtained in 88% yield (63.7 mg) as colorless oil.

^{1}H NMR (400 MHz, CDCl$_3$): δ 7.21 (dd, *J* = 7.5, 1.3 Hz, 1H), 7.11 (td, *J* = 7.3, 1.4 Hz, 1H), 7.02 – 6.96 (m, 1H), 6.94 – 6.89 (m, 2H), 6.89 – 6.78 (m, 4H), 6.77 – 6.68 (m, 3H), 2.30 (s, 2H), 0.07 (s, 6H).

^{13}C NMR (101 MHz, CDCl$_3$): δ 161.5 (d, *J* = 245.3 Hz), 160.6 (d, *J* = 243.9 Hz), 150.0, 140.2, 138.8 (d, *J* = 3.3 Hz), 138.6 , 137.6 (d, *J* = 3.3 Hz), 136.4, 132.2 (d, *J* = 7.7 Hz), 131.2, 130.1, 129.5 (d, *J* = 7.6 Hz), 127.5, 125.4, 115.0 (d, *J* = 14.3 Hz), 114.8 (d, *J* = 15.2 Hz), 21.3, -3.6.

^{19}F NMR (376 MHz, CDCl$_3$): δ -115.9, -118.1.

HRMS (EI): Calculated for $C_{23}H_{20}F_2Si$ $[M]^+$: 362.1302. Found: 362.1296.

2,2-Dimethyl-3,4-bis(4-(trifluoromethyl)phenyl)-1,2-dihydrobenzo[c]siline (6ac):

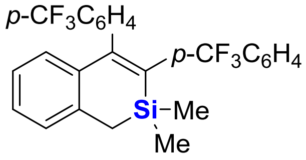

Following procedure A, the reaction was carried out with alkyne **2b** (0.2 mmol), benzosilacyclobutene **5a** (0.3 mmol), Ni(cod)$_2$ (5.8 mg, 0.02 mmol), PMe$_3$ (40μL, 1.0 M in THF, 0.04 mmol) and 1,4-dioxane (1.0 mL) at 100 ℃ for 24 h. The title compound was obtained in 85% yield (78.5 mg) as colorless oil.

^{1}H NMR (400 MHz, CDCl$_3$): δ 7.42 (d, *J* = 8.0 Hz, 2H), 7.36 (d, *J* = 8.1 Hz, 2H), 7.25 – 7.21 (m, 1H), 7.14 (td, *J* = 7.4, 1.4 Hz, 1H), 7.08 (d, *J* = 8.0 Hz, 2H), 7.00 (td, *J* = 7.7, 1.5 Hz, 1H), 6.88 (d, *J* = 8.0 Hz, 2H), 6.66 – 6.61 (m, 1H), 2.33 (s, 2H), 0.08 (s, 6H).

^{13}C NMR (101 MHz, CDCl$_3$): δ 150.0, 146.7 (q, *J* = 0.9 Hz), 145.3 (q, *J* = 1.3 Hz), 140.9, 137.8, 136.4, 131.4, 130.9, 130.2, 128.9 (q, *J* = 32.5 Hz), 128.2, 128.0, 127.4 (q, *J* = 32.4 Hz), 125.6, 125.1 (q, *J* = 3.8 Hz), 124.9 (q, *J* = 3.8 Hz), 124.4 (q, *J* = 271.7 Hz), 124.3 (d, *J* = 271.8 Hz), 21.2, -3.7.

^{19}F NMR (376 MHz, CDCl$_3$): δ -62.3, -62.4.

HRMS (EI): Calculated for $C_{25}H_{20}F_6Si$ [M]$^+$: 462.1238. Found: 462.1232.

3,4-Bis(4-methoxyphenyl)-2,2-dimethyl-1,2-dihydrobenzo[c]siline (6ad):

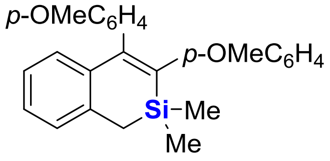

Following procedure A, the reaction was carried out with alkyne **2d** (0.2 mmol), benzosilacyclobutene **5a** (0.3 mmol), Ni(cod)$_2$ (5.8 mg, 0.02 mmol), PMe$_3$ (40µL, 1.0 M in THF, 0.04 mmol) and 1,4-dioxane (1.0 mL) at 100 ℃ for 24 h. The title compound was obtained in 82% yield (63.4 mg) as colorless oil.

^{1}H NMR (400 MHz, CDCl$_3$): δ 7.19 (dd, *J* = 7.4, 1.4 Hz, 1H), 7.08 (td, *J* = 7.4, 1.4 Hz, 1H), 6.97 (td, *J* = 7.6, 1.5 Hz, 1H), 6.92 – 6.86 (m, 2H), 6.79 (dd, *J* = 7.9, 1.3 Hz, 1H), 6.75 – 6.62 (m, 6H), 3.77 (s, 3H), 3.74 (s, 3H), 2.27 (s, 2H), 0.06 (s, 6H).

^{13}C NMR (101 MHz, CDCl$_3$): δ 158.0, 156.9, 149.9, 139.7, 139.5, 136.5, 135.6, 134.5, 132.0, 131.0, 130.1, 129.3, 127.0, 125.2, 113.3, 113.2, 55.2, 55.2, 21.5, -3.4.

HRMS (EI): Calculated for $C_{25}H_{26}O_2Si$ [M]$^+$: 386.1702. Found: 386.1697.

4,4'-(2,2-Dimethyl-1,2-dihydrobenzo[c]siline-3,4-diyl)bis(N,N-dimethylaniline) (6ae):

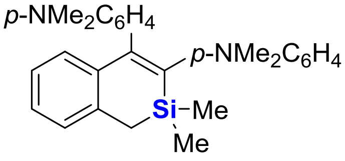

Following procedure A, the reaction was carried out with alkyne **2e** (0.2 mmol), benzosilacyclobutene **5a** (0.3 mmol), Ni(cod)$_2$ (5.8 mg, 0.02 mmol), PMe$_3$ (40µL, 1.0 M in THF, 0.04 mmol) and toluene (1.0 mL) at 100 ℃ for 24 h. The title compound was obtained in 90% yield (74.3 mg) as yellow solid.

^{1}H NMR (400 MHz, CDCl$_3$): δ 7.15 (d, J = 7.4 Hz, 1H), 7.04 (td, J = 7.4, 1.4 Hz, 1H), 6.95 (td, J = 7.6, 1.5 Hz, 1H), 6.88 – 6.79 (m, 3H), 6.71 (d, J = 8.2 Hz, 2H), 6.56 (d, J = 8.5 Hz, 4H), 2.91 (s, 6H), 2.88 (s, 6H), 2.24 (s, 2H), 0.05 (s, 6H).

^{13}C NMR (101 MHz, CDCl$_3$): δ 149.4, 148.7, 147.6, 140.2, 138.7, 136.7, 131.8, 130.8, 130.7, 130.1, 129.2, 126.5, 125.0, 112.4, 111.9, 40.9, 40.7, 21.6, -3.2.

HRMS (ESI): Calculated for [$C_{27}H_{32}N_2Si$ + H]$^+$: 413.2408. Found: 413.2404.

2,2-Dimethyl-3,4-di-*m*-tolyl-1,2-dihydrobenzo[*c*]saline (6af):

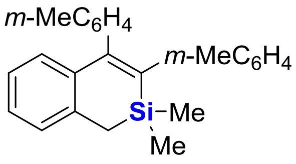

Following procedure A, the reaction was carried out with alkyne **2g** (0.2 mmol), benzosilacyclobutene **5a** (0.3 mmol), Ni(cod)$_2$ (5.8 mg, 0.02 mmol), PMe$_3$ (40µL, 1.0 M in THF, 0.04 mmol) and 1,4-dioxane (1.0 mL) at 100 ℃ for 24 h. The title

compound was obtained in 80% yield (56.6 mg) as colorless oil.

^{1}H NMR (400 MHz, CDCl$_3$): δ 7.21 (dd, *J* = 7.5, 1.4 Hz, 1H), 7.11 (td, *J* = 7.4, 1.4 Hz, 1H), 7.06 – 6.91 (m, 4H), 6.85 – 6.74 (m, 4H), 6.66 – 6.56 (m, 2H), 2.30 (s, 2H), 2.21 (s, 3H), 2.19 (s, 3H), 0.09 (s, 6H).

^{13}C NMR (101 MHz, CDCl$_3$): δ 150.4, 143.0, 141.9, 140.5, 139.2, 136.99, 136.96, 136.5, 131.3, 131.0, 130.3, 128.8, 127.9, 127.5, 127.4, 127.0, 126.9, 125.5, 125.2, 125.2, 21.6, 21.5, 21.5, -3.5.

HRMS (EI): Calculated for $C_{25}H_{26}Si$ $[M]^+$: 354.1804. Found: 354.1796.

3,4-Bis(3-fluorophenyl)-2,2-dimethyl-1,2-dihydrobenzo[*c*]saline (6ag):

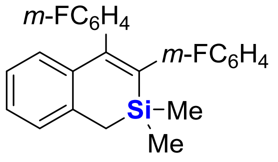

Following procedure A, the reaction was carried out with alkyne **2i** (0.2 mmol), benzosilacyclobutene **5a** (0.3 mmol), Ni(cod)$_2$ (5.8 mg, 0.02 mmol), PMe$_3$ (40μL, 1.0 M in THF, 0.04 mmol) and 1,4-dioxane (1.0 mL) at 100 ℃ for 24 h. The title compound was obtained in 82% yield (59.5 mg) as colorless oil.

^{1}H NMR (400 MHz, CDCl$_3$): δ 7.22 (dd, J = 7.5, 1.4 Hz, 1H), 7.16 – 7.04 (m, 3H), 7.00 (td, J = 7.6, 1.5 Hz, 1H), 6.85 (tdd, J = 8.5, 2.6, 1.0 Hz, 1H), 6.78 – 6.66 (m, 4H), 6.58 (dt, J = 7.7, 1.3 Hz, 1H), 6.51 (ddd, J = 10.1, 2.7, 1.6 Hz, 1H), 2.31 (s, 2H), 0.08 (s, 6H).

^{13}C NMR (101 MHz, CDCl$_3$): δ 162.6 (d, *J* = 245.4 Hz), 162.4 (d, *J* = 245.8 Hz), 149.7, 145.1 (d, *J* = 7.5 Hz), 143.7 (d, *J* = 7.4 Hz), 140.3 (d, *J* = 1.6 Hz), 137.9, 136.2, 131.2, 130.1, 129.4 (d, *J* = 8.7 Hz), 129.2 (d, *J* = 8.6 Hz), 127.6, 126.2 (d, *J* = 2.9 Hz), 125.4, 123.6 (d, *J* = 3.0 Hz), 117.3 (d, *J* = 21.2 Hz), 114.5 (d, *J* = 20.8 Hz), 113.5 (d, *J* = 20.5 Hz), 111.9 (d, *J* = 21.1 Hz), 21.1, -3.83.

^{19}F NMR (376 MHz, CDCl$_3$): δ -113.9, -114.0.

HRMS (EI): Calculated for $C_{23}H_{20}F_2Si$ $[M]^+$: 362.1302. Found: 362.1295.

2,2-Dimethyl-3,4-bis(3-(trifluoromethyl)phenyl)-1,2-dihydrobenzo[c]siline (6ah):

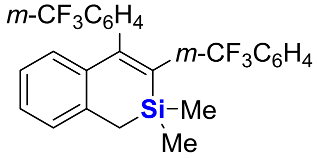

Following procedure A, the reaction was carried out with alkyne **2j** (0.2 mmol), benzosilacyclobutene **5a** (0.3 mmol), Ni(cod)$_2$ (5.8 mg, 0.02 mmol), PMe$_3$ (40μL, 1.0 M in THF, 0.04 mmol) and 1,4-dioxane (1.0 mL) at 100 ℃ for 24 h. The title compound was obtained in 80% yield (73.9 mg) as colorless oil.

^{1}H NMR (400 MHz, CDCl$_3$): δ 7.28 (d, *J* = 7.8 Hz, 1H), 7.20 – 7.08 (m, 5H), 7.07 – 6.99 (m, 2H), 6.91 (td, *J* = 7.6, 1.4 Hz, 1H), 6.86 (dt, *J* = 3.9, 1.9 Hz, 2H), 6.62 (dd, *J* = 7.8, 1.3 Hz, 1H), 2.24 (s, 2H), 0.00 (s, 6H).

^{13}C NMR (101 MHz, CDCl$_3$): δ 150.3, 143.4, 142.0, 140.8, 137.5, 136.2, 133.6, 131.4, 131.0, 130.4 (q, *J* = 32.1 Hz), 130.2 (q, *J* = 32.1 Hz), 128.4, 128.2, 127.8, 127.4 (q, 3.9 Hz), 125.5, 124.8 (q, *J* = 3.3 Hz), 124.0 (q, *J* = 272.6 Hz), 123.9 (d, *J* = 272.2 Hz), 123.4 (q, *J* = 3.4 Hz), 121.8 (q, *J* = 3.5 Hz), 21.0, -3.8.

^{19}F NMR (376 MHz, CDCl$_3$): δ -62.9, -63.0.

HRMS (EI): Calculated for $C_{25}H_{20}F_6Si$ $[M]^+$: 462.1238. Found: 462.1233.

3,4-Bis(3-methoxyphenyl)-2,2-dimethyl-1,2-dihydrobenzo[c]siline (6ai):

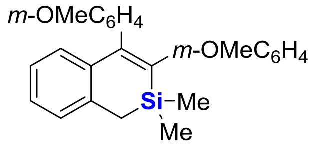

Following procedure A, the reaction was carried out with alkyne **2h** (0.2 mmol), benzosilacyclobutene **5a** (0.3 mmol), Ni(cod)$_2$ (5.8 mg, 0.02 mmol), PMe$_3$ (40μL,

1.0 M in THF, 0.04 mmol) and 1,4-dioxane (1.0 mL) at 100 ℃ for 24 h. The title compound was obtained in 83% yield (64.0 mg) as colorless oil.

^{1}H NMR (400 MHz, CDCl$_3$): δ 7.21 (dd, *J* = 7.6, 1.4 Hz, 1H), 7.13 – 6.96 (m, 4H), 6.82 (dd, *J* = 7.9, 1.4 Hz, 1H), 6.69 (ddd, *J* = 8.3, 2.6, 1.0 Hz, 1H), 6.61 (dt, *J* = 7.5, 1.2 Hz, 1H), 6.58 – 6.50 (m, 2H), 6.45 (dt, *J* = 7.6, 1.2 Hz, 1H), 6.34 (dd, *J* = 2.6, 1.5 Hz, 1H), 3.61 (s, 3H), 3.60 (s, 3H), 2.30 (s, 2H), 0.09 (s, 6H).

^{13}C NMR (101 MHz, CDCl$_3$): δ 159.2, 159.2, 150.4, 144.6, 143.4, 140.5, 138.8, 136.4, 131.1, 130.3, 128.8, 128.6, 127.2, 125.3, 123.2, 120.5, 115.9, 113.5, 112.8, 110.9, 55.3, 55.2, 21.5, -3.5.

HRMS (EI): Calculated for $C_{25}H_{26}O_2Si$ $[M]^+$: 386.1702. Found: 386.1694.

2,2-Diphenyl-3,4-dipropyl-1,2-dihydrobenzo[c]siline (6bj):

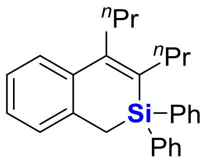

Following procedure B, the reaction was carried out with alkyne **2q** (0.2 mmol), benzosilacyclobutene **5c** (0.3 mmol), Ni(cod)$_2$ (5.8 mg, 0.02 mmol), PAd$_3$ (17.6 mg, 0.04 mmol) and toluene (1.0 mL) at 100 ℃ for 24 h. The title compound was obtained in 85% yield (65.0 mg) as colorless oil.

^{1}H NMR (400 MHz, CDCl$_3$): δ 7.51 – 7.44 (m, 4H), 7.41 (dd, *J* = 7.9, 1.3 Hz, 1H), 7.39 – 7.33 (m, 2H), 7.30 (tt, *J* = 6.5, 1.2 Hz, 4H), 7.10 (td, *J* = 7.6, 1.6 Hz, 1H), 7.03 (dd, *J* = 7.5, 1.6 Hz, 1H), 6.96 (td, *J* = 7.3, 1.3 Hz, 1H), 2.78 – 2.68 (m, 2H), 2.54 (s, 2H), 2.29 – 2.19 (m, 2H), 1.62 – 1.47 (m, 2H), 1.27 – 1.14 (m, 2H), 0.97 (t, *J* = 7.4 Hz, 3H), 0.73 (t, *J* = 7.3 Hz, 3H).

^{13}C NMR (101 MHz, CDCl$_3$): δ 150.6, 138.4, 135.5, 134.1, 133.9, 131.4, 129.4, 127.7, 126.3, 126.1, 125.5, 34.2, 32.2, 23.6, 22.8, 19.9, 14.4, 14.2.

HRMS (EI): Calculated for $C_{27}H_{30}Si$ $[M]^+$: 382.2117. Found: 382.2107.

3,4-Dibutyl-2,2-diphenyl-1,2-dihydrobenzo[*c*]siline (6bk):

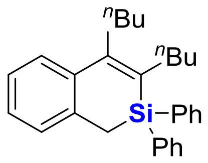

Following procedure B, the reaction was carried out with alkyne **2r** (0.2 mmol), benzosilacyclobutene **5c** (0.3 mmol), Ni(cod)$_2$ (5.8 mg, 0.02 mmol), PAd$_3$ (17.6 mg, 0.04 mmol) and toluene (1.0 mL) at 100 ℃ for 24 h. The title compound was obtained in 88% yield (72.0 mg) as colorless oil.

^{1}H NMR (400 MHz, CDCl$_3$): δ 7.51 – 7.45 (m, 4H), 7.41 (dd, *J* = 8.0, 1.3 Hz, 1H), 7.39 – 7.33 (m, 2H), 7.30 (tt, *J* = 6.6, 1.2 Hz, 4H), 7.10 (td, *J* = 7.6, 1.6 Hz, 1H), 7.03 (dd, *J* = 7.5, 1.6 Hz, 1H), 6.96 (td, *J* = 7.3, 1.3 Hz, 1H), 2.78 – 2.70 (m, 2H), 2.55 (s, 2H), 2.29 – 2.22 (m, 2H), 1.45 – 1.34 (m, 2H), 1.19 – 1.07 (m, 4H), 0.94 (t, *J* = 7.2 Hz, 3H), 0.74 – 0.63 (m, 3H).

^{13}C NMR (101 MHz, CDCl$_3$): δ 150.8, 138.6, 135.7, 135.3, 134.2, 133.7, 131.5, 129.6, 127.8, 126.4, 126.2, 125.6, 32.4, 32.1, 31.8, 30.2, 23.2, 23.1, 20.0, 14.2, 13.8.

HRMS (EI): Calculated for $C_{29}H_{34}Si$ $[M]^+$: 410.2430. Found: 410.2426.

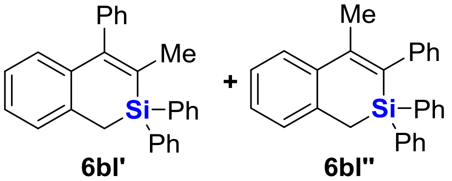

6bl' + **6bl"**

6bl: Following procedure B, the reaction was carried out with alkyne **2s** (0.2 mmol), benzosilacyclobutene **5c** (0.3 mmol), $Ni(cod)_2$ (5.8 mg, 0.02 mmol), PAd_3 (17.6 mg, 0.04 mmol) and toluene (1.0 mL) at 100 ℃ for 24 h. The product was obtained as a mixture in 80% yield (62.0 mg). rr = 3.5: 1.

^{1}H NMR (400 MHz, $CDCl_3$): δ 7.57 – 7.44 (m, 17H), 7.39 – 7.19 (m, 44H), 7.16 – 6.99 (m, 23H), 6.95 – 6.88 (m, 4H), 6.88 – 6.81 (m, 4H), 6.78 – 6.72 (m, 2H), 6.63 – 6.57 (m, 4H), 2.71 (s, 2H), 2.69 (s, 7H), 2.06 (s, 3H), 1.62 (s, 11H).

^{13}C NMR (101 MHz, $CDCl_3$): δ 153.2, 143.2, 142.0, 135.6, 135.6, 135.0, 133.9, 133.6, 131.8, 131.6, 130.01, 129.96, 129.8, 129.63, 129.59, 128.5, 128.1, 127.8, 127.4, 126.9, 126.7, 125.9, 125.5, 125.4, 19.8, 19.6, 19.6, 19.2.

HRMS (EI): Calculated for $C_{28}H_{24}Si$ $[M]^+$: 388.1647. Found: 388.1642.

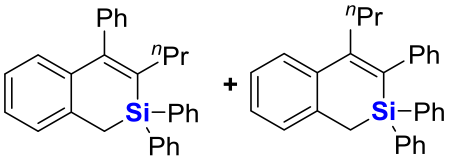

6bm' + **6bm"**

6bm: Following procedure B, the reaction was carried out with alkyne **2t** (0.2 mmol), benzosilacyclobutene **5c** (0.3 mmol), $Ni(cod)_2$ (5.8 mg, 0.02 mmol), PAd_3 (17.6 mg, 0.04 mmol) and toluene (1.0 mL) at 100 ℃ for 24 h. The product was obtained as a mixture in 75% yield (62.4 mg). rr = 6: 1.

^{1}H NMR (400 MHz, $CDCl_3$): δ 7.67 – 7.59 (m, 1H), 7.59 – 7.46 (m, 18H), 7.42 – 7.26 (m, 39H), 7.25 – 7.11 (m, 18H), 7.06 (m, 8H), 6.89 (m, 9H), 6.81 – 6.77 (m, 1H), 6.64 (m, 4H), 2.71 (s, 1H), 2.69 (s, 7H), 2.59 – 2.52 (m, 1H), 1.98 – 1.86 (m, 9H), 1.43 (m, 1H), 1.18 – 1.06 (m, 8H), 0.76 (t, J = 7.4 Hz, 2H), 0.48 (t, J = 7.2 Hz, 12H).

^{13}C NMR (101 MHz, $CDCl_3$): δ 152.37, 151.50, 143.08, 142.09, 138.99, 135.61, 135.57, 135.47, 134.72, 133.71, 133.43, 131.41, 129.97, 129.68, 129.55, 129.48, 129.46, 128.40, 128.22, 127.96, 127.90, 127.62, 127.18, 126.79, 126.63, 126.59, 125.79, 125.35, 125.21, 35.66, 33.42, 23.73, 22.44, 19.85, 19.54, 14.43, 14.05.

HRMS (EI): Calculated for $C_{30}H_{28}Si$ $[M]^+$: 416.1960. Found: 416.1951.

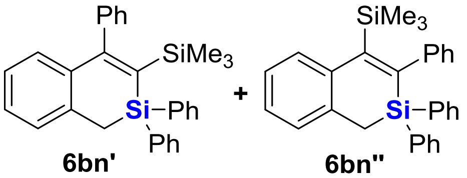

6bn' + **6bn"**

6bn: Following procedure B, the reaction was carried out with alkyne **2s** (0.2 mmol), benzosilacyclobutene **5c** (0.3 mmol), $Ni(cod)_2$ (5.8 mg, 0.02 mmol), PAd_3 (17.6 mg, 0.04 mmol) and toluene (1.0 mL) at 100 ℃ for 24 h. The product was obtained as a mixture in 85% yield (75.8 mg). rr = 1.9: 1.

^{1}H NMR (400 MHz, $CDCl_3$): δ 7.66 (m, 1H), 7.54 – 7.43 (m, 13H), 7.38 – 7.06 (m, 41H), 7.04 – 6.89 (m, 6H), 6.84 (m, 2H), 6.80 – 6.70 (m, 4H), 2.83 (s, 2H), 2.54 (s, 4H), 0.09 (s, 9H), -0.56 (s, 17H).

^{13}C NMR (101 MHz, $CDCl_3$): δ 166.8, 145.0, 143.8, 138.4, 138.2, 137.0, 135.93, 135.90, 135.33, 134.9, 134.6, 134.1, 133.9, 132.3, 131.5, 130.4, 130.3, 130.1, 130.0, 129.9, 129.6, 129.4, 129.3, 129.1, 128.9, 128.8, 128.4, 128.4, 128.1, 128.0, 127.9, 127.8, 127.2, 127.0, 126.7, 126.6, 126.2, 125.4, 124.4, 24.7, 21.1, 2.3, 0.8.

HRMS (EI): Calculated for $C_{30}H_{30}Si_2$ $[M]^+$: 446.1886. Found: 446.1880.

2,2-Dibutyl-3,4-diphenyl-1,2-dihydrobenzo[c]siline (6ba):

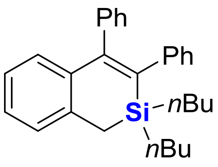

Following procedure B, the reaction was carried out with diphenylacetylene (0.2 mmol), benzosilacyclobutene **5b** (0.3 mmol), Ni(cod)$_2$ (5.8 mg, 0.02 mmol), PAd$_3$ (17.6 mg, 0.04 mmol) and toluene (1.0 mL) at 100 ℃ for 24 h. The title compound was obtained in 88% yield (72.2 mg) as colorless oil.

^{1}H NMR (400 MHz, $CDCl_3$): δ 7.21 (d, J = 7.5 Hz, 1H), 7.10 (dt, J = 20.4, 7.1 Hz, 6H), 6.98 (dd, J = 7.5, 2.6 Hz, 4H), 6.79 (d, J = 7.5 Hz, 2H), 6.74 (d, J = 7.8 Hz, 1H), 2.32 (s, 2H), 1.25 – 1.12 (m, 8H), 0.80 – 0.72 (m, 6H), 0.58 (m, 4H).

^{13}C NMR (101 MHz, $CDCl_3$): δ 151.3, 143.3, 142.1, 140.0, 139.1, 136.8, 131.0, 130.8, 130.2, 128.3, 127.7, 127.6, 127.2, 126.2, 125.2, 124.8, 26.5, 26.1, 18.5, 13.8, 12.1.

HRMS (EI): Calculated for $C_{29}H_{34}Si$ $[M]^+$: 410.2430. Found: 410.2424.

2,2,3,4-Tetraphenyl-1,2-dihydrobenzo[c]siline (6ca):

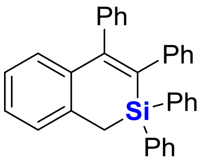

Following procedure B, the reaction was carried out with diphenylacetylene (0.2 mmol), benzosilacyclobutene **5c** (0.3 mmol), Ni(cod)$_2$ (5.8 mg, 0.02 mmol), PAd$_3$ (17.6 mg, 0.04 mmol) and toluene (1.0 mL) at 100 ℃ for 24 h. The title compound was obtained in 90% yield (81 mg) as colorless oil.

^{1}H NMR (400 MHz, $CDCl_3$): δ 7.38 – 7.30 (m, 4H), 7.25 (m, 2H), 7.21 – 7.13 (m, 5H), 7.10 – 7.02 (m, 3H), 7.02 – 6.96 (m, 3H), 6.92 (m, 1H), 6.83 (m, 2H), 6.77 (dd, J = 11.1, 7.3 Hz, 2H), 6.69 – 6.62 (m, 2H), 2.78 (s, 2H).

^{13}C NMR (101 MHz, $CDCl_3$): δ 153.6, 142.7, 141.8, 138.9, 137.4, 135.6, 135.5, 133.6, 131.5, 130.7, 130.6, 129.7, 128.7, 127.9, 127.7, 127.7, 127.6, 126.5, 125.6, 125.0, 20.4.

HRMS (EI): Calculated for $C_{33}H_{26}Si$ $[M]^+$: 450.1804. Found: 450.1796.

2,2,8-Trimethyl-3,4-diphenyl-1,2-dihydrobenzo[*c*]saline (6da):

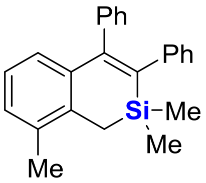

Following procedure A, the reaction was carried out with diphenylacetylene (0.2 mmol), benzosilacyclobutene **5d** (0.3 mmol), Ni(cod)$_2$ (5.8 mg, 0.02 mmol), PMe$_3$ (40μL, 1.0 M in THF, 0.04 mmol) and 1,4-dioxane (1.0 mL) at 100 ℃ for 24 h. The title compound was obtained in 86% yield (57.8 mg) as colorless oil.

^{1}H NMR (400 MHz, $CDCl_3$): δ 7.17 – 7.02 (m, 6H), 7.02 – 6.93 (m, 3H), 6.88 (t, J = 7.7 Hz, 1H), 6.83 – 6.77 (m, 2H), 6.65 (dd, J = 8.0, 1.4 Hz, 1H), 2.40 (s, 3H), 2.21 (s, 2H), 0.08 (s, 6H).

^{13}C NMR (101 MHz, CDCl$_3$): δ 151.0, 143.2, 142.5, 140.5, 139.1, 136.7, 135.0, 130.7, 129.4, 128.7, 128.1, 127.8, 127.6, 126.2, 124.8, 124.4, 21.6, 16.1, -3.5.

HRMS (EI): Calculated for $C_{24}H_{24}Si$ $[M]^+$: 340.1647. Found: 340.1639. `

2,2,7-Trimethyl-3,4-diphenyl-1,2-dihydrobenzo[c]saline (6ea):

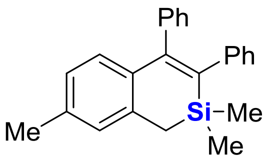

Following procedure A, the reaction was carried out with diphenylacetylene (0.2 mmol), benzosilacyclobutene **5e** (0.3 mmol), Ni(cod)$_2$ (5.8 mg, 0.02 mmol), PMe$_3$ (40μL, 1.0 M in THF, 0.04 mmol) and 1,4-dioxane (1.0 mL) at 100 ℃ for 24 h. The title compound was obtained in 85% yield (57.8 mg) as colorless oil.

^{1}H NMR (400 MHz, CDCl$_3$): δ 7.18 – 7.02 (m, 6H), 7.02 – 6.94 (m, 3H), 6.85 – 6.75 (m, 3H), 6.66 (d, *J* = 7.9 Hz, 1H), 2.31 (s, 3H), 2.28 (s, 2H), 0.09 (s, 6H).

^{13}C NMR (101 MHz, CDCl$_3$): δ 150.4, 143.3, 142.1, 139.6, 137.0, 136.3, 132.0, 130.7, 130.3, 128.2, 127.8, 127.6, 126.2, 125.9, 124.7, 21.4, 21.2, -3.4.

HRMS (EI): Calculated for $C_{24}H_{24}Si$ $[M]^+$: 340.1647. Found: 340.1639.

2,2-Dimethyl-3,4-diphenyl-7-(trifluoromethyl)-1,2-dihydrobenzo[c]siline (6fa):

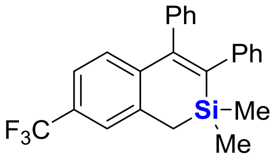

Following procedure A, the reaction was carried out with diphenylacetylene (0.2 mmol), benzosilacyclobutene **5f** (0.3 mmol), Ni(cod)$_2$ (5.8 mg, 0.02 mmol), PMe$_3$ (40μL, 1.0 M in THF, 0.04 mmol) and 1,4-dioxane (1.0 mL) at 100 ℃ for 24 h. The title compound was obtained in 69% yield (54.4mg) as colorless oil.

^{1}H NMR (400 MHz, CDCl$_3$): δ 7.44 (s, 1H), 7.22 – 7.18 (m, 1H), 7.16 – 7.07 (m, 5H), 7.03 – 6.98 (m, 1H), 6.96 – 6.92 (m, 2H), 6.85 (d, *J* = 8.2 Hz, 1H), 6.80 – 6.76 (m, 2H), 2.35 (s, 2H), 0.09 (s, 6H).

^{13}C NMR (101 MHz, CDCl$_3$): δ 149.4, 143.2, 142.6, 142.3 (q, *J* = 1.2 Hz), 137.4, 130.6, 130.3, 128.7 (q, *J* = 32.2 Hz), 128.0, 127.90, 127.87, 127.4 (q, *J* = 3.8 Hz), 126.7, 125.2, 124.3 (q, *J* = 272.0 Hz), 122.1 (q, *J* = 4.0 Hz), 21.5, -3.7.

^{19}F NMR (376 MHz, CDCl$_3$): δ -62.5.

HRMS (EI) calcd. For $C_{24}H_{21}F_3Si$ $[M]^+$: 394.1365. Found: 394.1357.

2,2,6-Trimethyl-3,4-diphenyl-1,2-dihydrobenzo[*c*]saline (6ga):

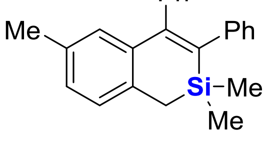

Following procedure A, the reaction was carried out with diphenylacetylene (0.2 mmol), benzosilacyclobutene **5g** (0.3 mmol), Ni(cod)$_2$ (5.8 mg, 0.02 mmol), PMe$_3$ (40μL, 1.0 M in THF, 0.04 mmol) and 1,4-dioxane (1.0 mL) at 100 ℃ for 24 h. The title compound was obtained in 87% yield (59.2 mg) as colorless oil.

^{1}H NMR (400 MHz, CDCl$_3$): δ 7.16 – 7.05 (m, 6H), 7.00 – 6.89 (m, 4H), 6.78 (dt, *J* = 6.3, 1.4 Hz, 2H), 6.58 (d, *J* = 1.9 Hz, 1H), 2.25 (s, 2H), 2.12 (s, 3H), 0.06 (s, 6H).

^{13}C NMR (101 MHz, CDCl$_3$): δ 150.6, 143.3, 142.0, 140.8, 138.8, 134.6, 133.22, 131.04, 130.97, 130.8, 128.1, 127.9, 127.8, 127.6, 126.2, 124.8, 21.2, 20.8, -3.5.

HRMS (EI): Calculated for $C_{24}H_{24}Si$ $[M]^+$: 340.1647. Found: 340.1639.

6-Fluoro-2,2-dimethyl-3,4-diphenyl-1,2-dihydrobenzo[c]siline (6ha):

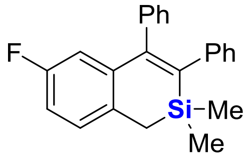

Following procedure A, the reaction was carried out with diphenylacetylene (0.2 mmol), benzosilacyclobutene **5h** (0.3 mmol), Ni(cod)$_2$ (5.8 mg, 0.02 mmol), PMe$_3$ (40μL, 1.0 M in THF, 0.04 mmol) and 1,4-dioxane (1.0 mL) at 100 ℃ for 24 h. The title compound was obtained in 80% yield (62.0 mg) as colorless oil.

^{1}H NMR (400 MHz, CDCl$_3$): δ 7.18 – 7.05 (m, 5H), 7.02 – 6.89 (m, 4H), 6.82 – 6.76 (m, 2H), 6.73 (dd, *J* = 8.8, 6.2 Hz, 1H), 6.65 (td, *J* = 8.5, 2.8 Hz, 1H), 2.30 (s, 2H), 0.09 (s, 6H).

^{13}C NMR (101 MHz, CDCl$_3$): δ 161.3 (d, *J* = 247.8 Hz), 149.5, 139.8 (d, *J* = 1.4 Hz), 139.3 (d, *J* = 7.6 Hz), 135.1 (d, *J* = 3.2 Hz), 131.8 (d, *J* = 8.2 Hz), 127.7 (d, *J* = 9.7 Hz), 126.3, 124.8, 117.5 (d, *J* = 20.7 Hz), 111.6 (d, *J* = 20.5 Hz), 21.7, -3.7. HRMS (EI): Calculated for C19H18F6Si [M]+: 388.1082. Found: 388.1078.

Scale-up experiment and derivatization of 3aa and 4ca

Gram-scale experiment for 3aa

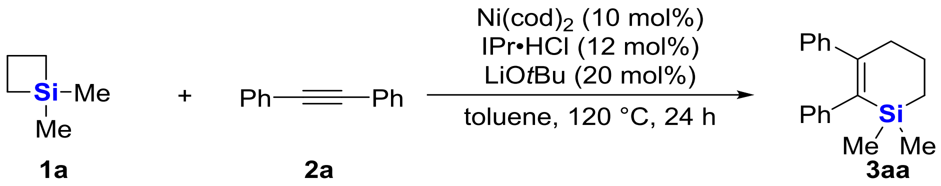

In a nitrogen-filled glove-box, an oven-dried 25 mL glass vial equipped with a magnetic stir bar was charged with Ni(cod)$_2$ (87.0 mg, 0.3 mmol), IPr·HCl (155.0 mg, 0.36 mmol), LiO^{t}Bu (48.0 mg, 0.6 mmol) and toluene (5.0 mL). The mixture was allowed to stir under ambient temperature for 30 min. Then silacyclobutane **1a** (0.6 g, 6.0 mmol, 2 equiv) and alkyne **2a** (0.53 g, 3.0 mmol, 1 equiv) were added sequentially. The vial was sealed with a PTFE cap, removed from the glove box and stirred at 120 ℃ for 24 h. After being cooled to room temperature, the solvent was removed under vacuum and the residue was subjected to silica gel column chromatography (petroleum ether). **3aa** was obtained in 73% yield (0.61 g) as colorless oil. ^{1}H NMR data matched that for **3aa** synthesized on small scale.

Derivatization of 3aa

<u>Preparation of 7</u>

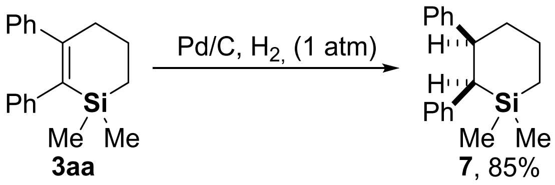

A mixture of Pd(OH)$_2$ on carbon (10.6 mg, 10 wt% Pd, 10 μmol) and **3aa** (55.0 mg, 0.20 mmol) in 1.5 mL of an ethanol/EtOAc mixture (3: 2) was stirred for 12 h at room temperature under H$_2$ (1 atm.). The catalyst was

filtered off through celite with EtOAc, and the solvent was removed under vacuum. The residue was purified *via* silica gel chromatography (petroleum ether). **7** was obtained in 85% yield (48.0 mg) as colorless oil.

^{1}H NMR (400 MHz, CDCl$_3$): δ 7.29 – 7.18 (m, 6H), 7.04 – 6.95 (m, 2H), 6.93 – 6.84 (m, 2H), 3.43 – 3.34 (m, 1H), 2.57 – 2.42 (m, 2H), 2.32 – 2.17 (m, 1H), 1.97 – 1.81 (m, 2H), 1.19 – 1.06 (m, 2H), 0.50 (s, 3H), 0.02 (s, 3H).

^{13}C NMR (101 MHz, CDCl$_3$): δ 146.9, 141.2, 131.1, 127.6, 127.5, 127.4, 125.4, 124.8, 47.3, 41.3, 29.1, 24.5, 12.7, -2.8.

HRMS (EI): Calculated for $C_{19}H_{24}Si$ $[M]^+$: 280.1647. Found: 280.1642.

<u>**Preparation of 8**</u>

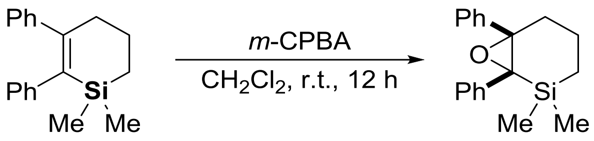

3aa **8**, 77%

To a solution of **3aa** (55.0 mg, 0.2 mmol) in CH_2Cl_2 (2.0 mL) at 0 ℃ was added *m*-CPBA (~70%, 80.0 mg, 0.3 mmol, 1.5 equiv) and the mixture was allowed to stir for 12 h at room temperature afterwards. Once complete, the reaction was quenched with sat. $NaHCO_3$ (aq.) and extracted with CH_2Cl_2, the combined organic layers were dried over Na_2SO_4 and concentrated *in vacuo*. The residue was purified *via* silica gel chromatography (petroleum ether), and **8** was obtained in 77% yield (45.3 mg) as colorless oil.

^{1}H NMR (400 MHz, CDCl$_3$): δ 7.16 – 7.10 (m, 2H), 7.08 – 6.89 (m, 8H), 2.49 – 2.39 (m, 1H), 2.25 – 2.17 (m, 1H), 1.95 – 1.75 (m, 2H), 0.91 – 0.81 (m, 1H), 0.67 – 0.54 (m, 1H), 0.18 (s, 3H), 0.01 (s, 3H).

^{13}C NMR (101 MHz, CDCl$_3$): δ 141.2, 139.2, 127.5, 127.3, 126.9, 126.5, 126.2, 125.6, 69.3, 66.2, 34.3, 17.3, 11.7, -2.0, -5.5.

HRMS (EI): Calculated for $C_{19}H_{22}OSi$ $[M]^+$: 294.1440. Found: 294.1435.

Gram-scale experiment for 4ca

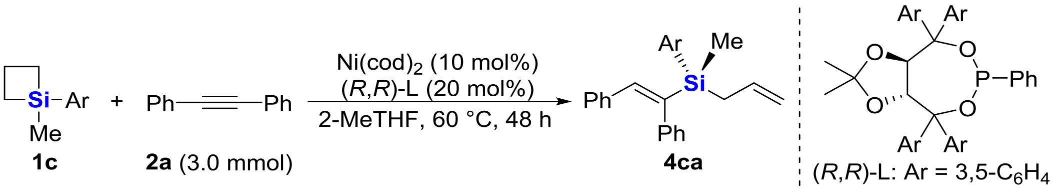

1c **2a** (3.0 mmol) **4ca** (*R*,*R*)-L: Ar = 3,5-C$_6$H$_4$

In a nitrogen-filled glove-box, an oven-dried 25 mL Schlenk tube equipped with a stir bar was charged with Ni(cod)$_2$ (82.5 mg, 0.3 mmol), (*R*,*R*)**-L** (410 mg, 0.6 mmol) and toluene (5.0 mL). The mixture was allowed to stir under ambient temperature for 1 h. Then diphenylacetylene (535.0 mg, 3.0 mmol) and silacyclobutane **1c** (1.05 g, 6.0 mmol, 2.0 equiv.) were added to the mixture sequentially. The tube was then sealed and removed from the glove box, stirred at 60 ℃ for 48 h. After being cooled to room temperature, the solvent was removed under vacuum and the residue was purified *via* silica gel chromatography (petroleum ether/DCM = 20: 1). **4ca** was obtained in 85% yield (0.90 g), ee = 92%. **HPLC**: Daicel chiralpak OD-3 column, *n*-hexane, 0.5 mL/min, 254 nm UV detector, t (major) = 16.7 min, t (minor) = 18.2 min.

^{1}H NMR (400 MHz, CDCl$_3$): δ 7.43 – 7.37 (m, 1H), 7.32 – 7.20 (m, 2H), 7.19 – 7.07 (m, 5H), 7.05 – 6.95 (m, 3H), 6.95 – 6.81 (m, 4H), 6.72 (s, 1H), 5.73 – 5.55 (m, 1H), 4.91 – 4.65 (m, 2H), 2.44 (s, 3H), 2.03 – 1.89 (m, 1H), 1.89 – 1.75 (m, 1H), 0.34 (s, 2H).

^{1}H NMR data matched that for **4ca** synthesized on small scale.

<rac-4ca>

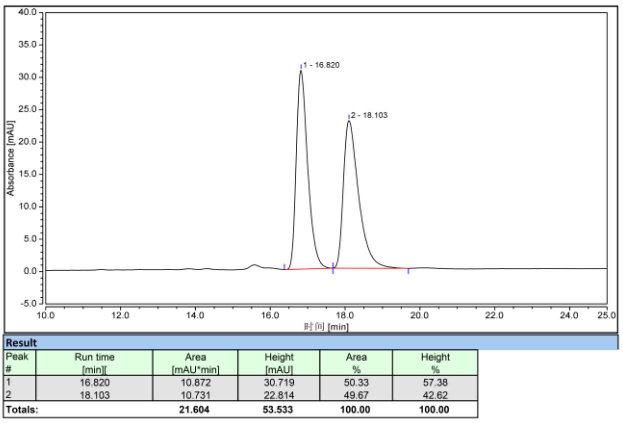

Result

Peak #	Run time [min][	Area [mAU*min]	Height [mAU]	Area %	Height %
1	16.820	10.872	30.719	50.33	57.38
2	18.103	10.731	22.814	49.67	42.62
**Totals:**		**21.604**	**53.533**	**100.00**	**100.00**

<chiral-4ca>

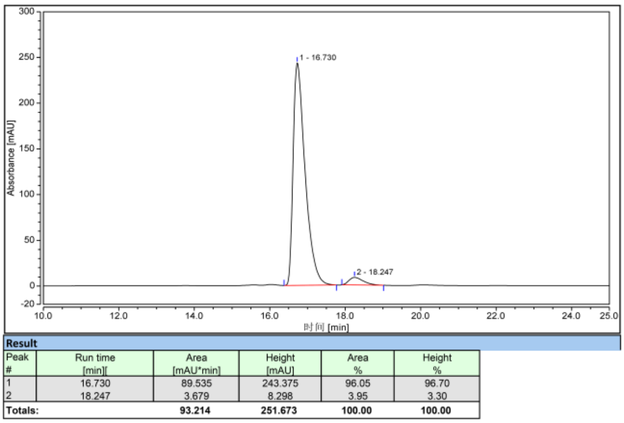

Result

Peak #	Run time [min][	Area [mAU*min]	Height [mAU]	Area %	Height %
1	16.730	89.535	243.375	96.05	96.70
2	18.247	3.679	8.298	3.95	3.30
**Totals:**		**93.214**	**251.673**	**100.00**	**100.00**

Derivatization of 4ca

<u>Preparation of 9</u>

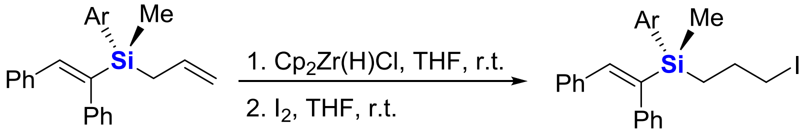

4ca **9**

To a suspension of $Cp_2Zr(H)Cl$ (162.4 mg, 0.62 mmol, 3.0 equiv.) in THF (0.5 mL) was added a solution of **4ca** (70.9 mg, 0.20 mmol; 92% ee) in THF (0.5 mL). After being stirred at room temperature for 2 h, iodine (152.3 mg, 0.6 mmol, 3.0 equiv.) in THF (0.5 mL) was added to the reaction mixture at 0 ℃. The mixture was allowed to stir for 1 h at room temperature afterwards. Once complete, the reaction was quenched with sat. $Na_2S_2O_3$ (aq.) and extracted with $CHCl_3$, the combined organic layers were dried over Na_2SO_4 and concentrated *in vacuo*. The residue was purified *via* silica gel chromatography (petroleum ether/dichloromethane = 20: 1), and **9** was obtained in 90% yield (86.8 mg) as colorless oil, ee = 92%, Optical rotation: $[\alpha]_D^{25}$ 9.1 (c = 0.57, CHCl$_3$).

^{1}H NMR (400 MHz, CDCl$_3$): δ 7.49 (d, J = 7.3 Hz, 1H), 7.39 – 7.34 (m, 1H), 7.30 – 7.20 (m, 5H), 7.15 – 7.08 (m, 3H), 7.00 – 6.90 (m, 4H), 6.82 (s, 1H), 3.20 (t, J = 7.0 Hz, 2H), 2.55 (s, 3H), 1.89 – 1.76 (m, 2H), 1.18 – 1.09 (m, 1H), 1.06 – 0.97 (m, 1H), 0.46 (s, 3H).

^{13}C NMR (101 MHz, CDCl$_3$): δ 144.3, 143.7, 142.2, 139.9, 137.2, 135.7, 134.3, 130.2, 129.9, 129.7, 128.8, 128.0, 127.6, 127.4, 126.0, 125.2, 28.7, 23.4, 15.9, 11.4, -3.6.

HRMS (ESI): Calculated for $[C_{25}H_{27}ISi, M+Na]^+$: 505.0819. Found: 505.0819.

HPLC: Daicel chiralpak OD-3 column, *n*-hexane, 0.5 mL/min, 254 nm UV detector, t (minor) = 46.1 min, t (major) = 49.0 min.

<rac-**9**>

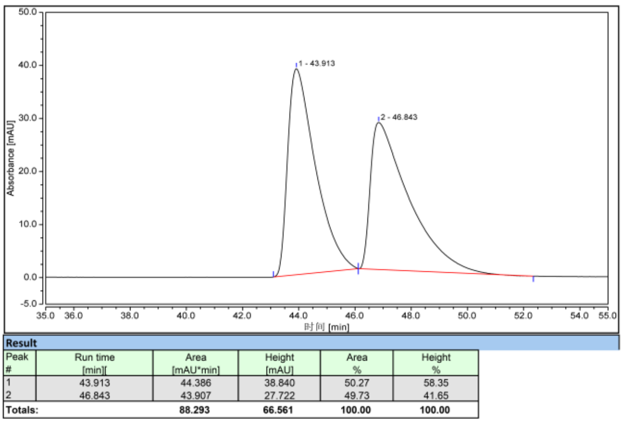

Result

Peak #	Run time [min][	Area [mAU*min]	Height [mAU]	Area %	Height %
1	43.913	44.386	38.840	50.27	58.35
2	46.843	43.907	27.722	49.73	41.65
**Totals:**		**88.293**	**66.561**	**100.00**	**100.00**

<chiral-**9**>

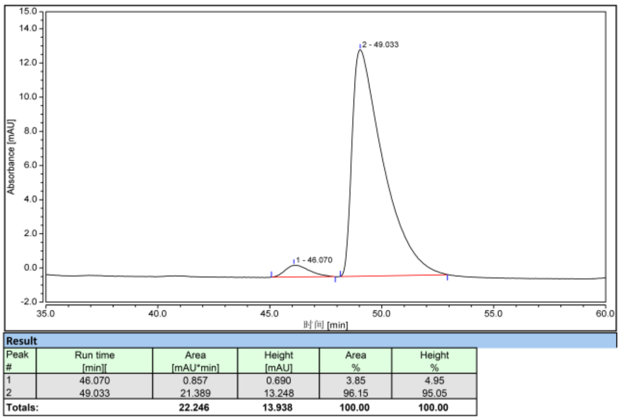

Result

Peak #	Run time [min][	Area [mAU*min]	Height [mAU]	Area %	Height %
1	46.070	0.857	0.690	3.85	4.95
2	49.033	21.389	13.248	96.15	95.05
**Totals:**		**22.246**	**13.938**	**100.00**	**100.00**

Preparation of 10

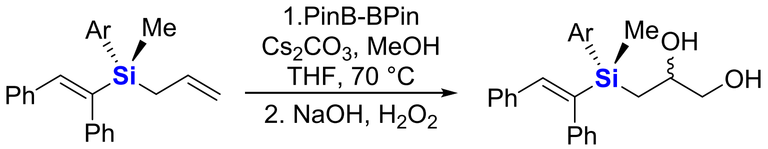

4ca **10**

In a nitrogen-filled glove-box, an oven-dried 8 mL glass vial equipped with a stir bar was charged with **4ca** (70.9 mg, 0.20 mmol; 92% ee), bis(pinacolato)diboron (102.0 mg, 0.4 mmol, 2.0 equiv.), Cs_2CO_3 (19.5 mg, 0.06 mmol, 0.3 equiv.), MeOH (40 μL, 1.0 mmol, 5.0 equiv.) and THF (1.0 mL). The vial was sealed with a PTFE cap, removed from the glove box and stirred at 70 ℃ for 12 h. The mixture was then cooled to 0 ℃ in an ice-water bath, followed by the dropwise addition of H_2O_2 (120 μL, 30% aqueous solution, 1.0 mmol, 5.0 equiv.) and NaOH (0.4 mL, 2.5 M aqueous solution, 1.0 mmol, 5.0 equiv.). The resulting mixture was allowed to stir for 1 h at room temperature afterwards. Once complete, the reaction was quenched with water and extracted with EtOAc. The combined organic layers were dried over Na_2SO_4 and concentrated *in vacuo*. The residue was purified *via* silica gel chromatography (petroleum ether/EtOAc = 2: 1), and **10** was obtained in 82% yield (63.7 mg) as colorless oil, dr = 1: 1, ee = 92%, Optical rotation: $[\alpha]_D^{25}$ 20.7 (c = 0.72, $CHCl_3$).

^{1}H NMR (400 MHz, $CDCl_3$): δ 7.54 – 7.49 (m, 2H), 7.36 – 7.31 (m, 2H), 7.25 – 7.15 (m, 10H), 7.11 – 7.06 (m, 6H), 6.96 – 6.89 (m, 8H), 6.81 (s, 1H), 6.80 (s, 1H), 3.87 – 3.74 (m, 2H), 3.54 – 3.42 (m, 2H), 3.36 – 3.23 (m, 2H), 2.54 (s, 3H), 2.51 (s, 3H), 1.38 – 1.29 (m, 2H), 1.24 – 1.15 (m, 2H), 0.55 (s, 3H), 0.53 (s, 3H).

^{13}C NMR (101 MHz, $CDCl_3$): δ 144.3, 144.2, 144.0, 143.7, 142.2, 142.1, 140.1, 139.9, 137.2, 137.2, 135.9, 135.8, 134.5, 134.3, 130.4, 130.1, 120.0, 129.7, 128.8, 128.0, 127.7, 127.45, 127.41, 126.1, 125.4, 125.3, 70.1, 70.0, 69.0, 29.8, 25.0, 23.5, 23.4, 18.9, -2.2, -2.6.

HRMS (ESI): Calculated for $C_{29}H_{34}Si$ [$C_{25}H_{28}O_2Si$, M+Na]$^+$: 411.1751. Found: 411.1750.

HPLC: Daicel chiralpak OD-3 column, *n*-hexane/*i*-PrOH = 91/9, 0.5 mL/min, 254 nm UV detector, t (major) = 19.0 min, t (minor) = 21.4 min.

<rac-**10**>

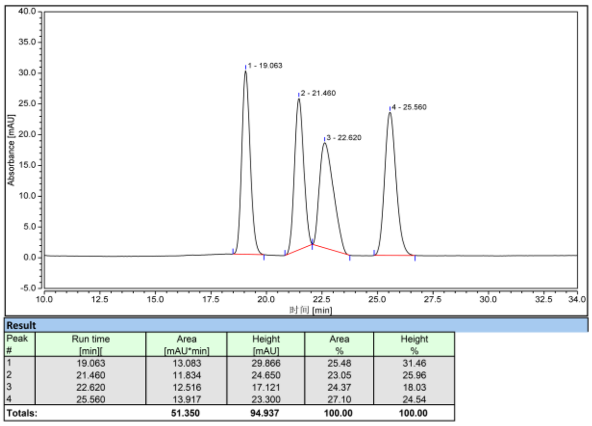

Result

Peak #	Run time [min][	Area [mAU*min]	Height [mAU]	Area %	Height %
1	19.063	13.083	29.866	25.48	31.46
2	21.460	11.834	24.650	23.05	25.96
3	22.620	12.516	17.121	24.37	18.03
4	25.560	13.917	23.300	27.10	24.54
**Totals:**		**51.350**	**94.937**	**100.00**	**100.00**

<chiral-**10**>

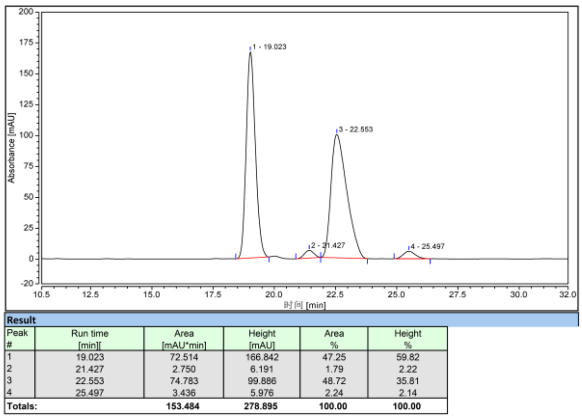

Result

Peak #	Run time [min][	Area [mAU*min]	Height [mAU]	Area %	Height %
1	19.023	72.514	166.842	47.25	59.82
2	21.427	2.750	6.191	1.79	2.22
3	22.553	74.783	99.886	48.72	35.81
4	25.497	3.436	5.976	2.24	2.14
**Totals:**		**153.484**	**278.895**	**100.00**	**100.00**

Preparation of 11

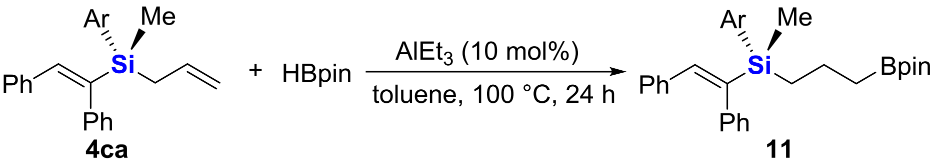

In a nitrogen-filled glove-box, an oven-dried 8 mL glass vial equipped with a stir bar was charged with **4ca** (70.9 mg, 0.20 mmol; 92% ee), Pinacolatoborane (45 μL, 0.3 mmol, 1.5 equiv.), $AlEt_3$ (1 M in toluene, 20 μL, 0.02 mmol, 10 mol%) and toluene (1.0 mL). The vial was sealed with a PTFE cap, removed from the glove box and stirred at 100 ℃ for 24 h. After being cooled to room temperature, HCl in dioxane (4 M, 0.5 mL) was added to quench the reaction. The resulting mixture was then filtered through a short pad of celite, eluting with EtOAc, the filtrate was dried over Na_2SO_4 and concentrated *in vacuo*. The crude product was purified *via* silica gel chromatography (petroleum ether/EtOAc = 15: 1), and **11** was obtained in 85% yield (82.0 mg) as colorless oil, the ee value of **11** was measured by HPLC analysis of the corresponding alcohol after oxidation of the boryl group (92% ee). Optical rotation: $[\alpha]_D^{25}$ -158.6 (c = 0.06, $CHCl_3$).

^{1}H NMR (400 MHz, $CDCl_3$): δ 7.45 – 7.38 (m, 1H), 7.26 – 7.19 (m, 1H), 7.16 – 7.04 (m, 5H), 7.03 – 6.96 (m, 3H), 6.88 – 6.78 (m, 4H), 6.69 (s, 1H), 2.42 (s, 3H), 1.46 – 1.34 (m, 2H), 1.15 (s, 12H), 1.03 – 0.92 (m, 1H), 0.91 – 0.84 (m, 1H), 0.78 (t, J = 7.3 Hz, 2H), 0.31 (s, 3H).

^{13}C NMR (101 MHz, $CDCl_3$): δ 144.7, 144.4, 142.6, 139.2, 137.5, 135.9, 135.1, 130.0, 129.6, 129.5, 128.6, 128.0, 127.6, 127.1, 125.8, 125.0, 83.0, 25.00, 24.97, 23.3, 18.6, 17.1, -3.8.

HRMS (ESI): Calculated for $[C_{31}H_{39}BO_2Si, M+Na]^+$: 505.2705. Found: 505.2702.

HPLC: Daicel chiralpak OD-3 column, *n*-hexane/*i*-PrOH = 90/10, 0.5 mL/min, 254 nm UV detector, t (major) = 13.6 min, t (minor) = 15.5 min.

<rac-**11-OH**>

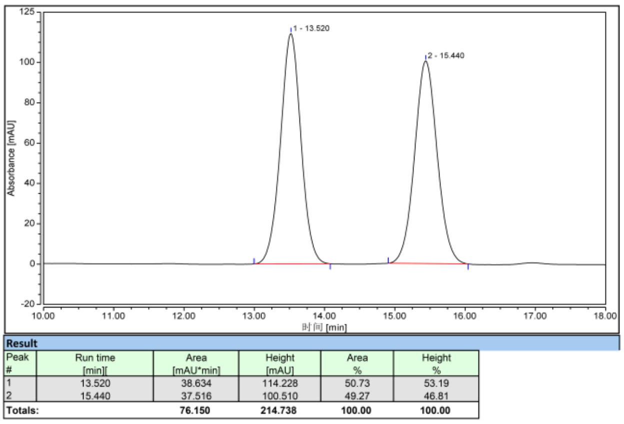

Result

Peak #	Run time [min]	Area [mAU*min]	Height [mAU]	Area %	Height %
1	13.520	38.634	114.228	50.73	53.19
2	15.440	37.516	100.510	49.27	46.81
Totals:		76.150	214.738	100.00	100.00

<chiral-**11-OH**>

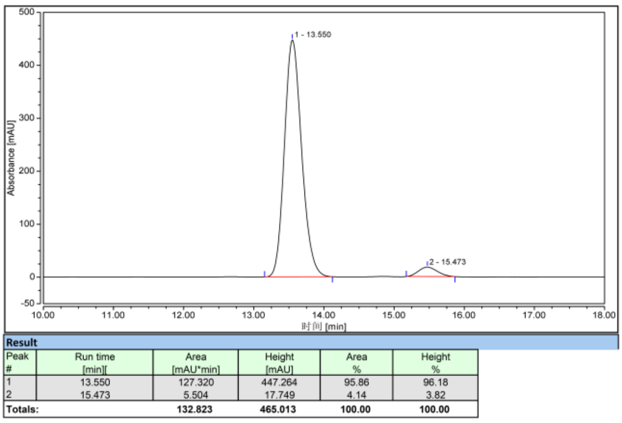

Result

Peak #	Run time [min]	Area [mAU*min]	Height [mAU]	Area %	Height %
1	13.550	127.320	447.264	95.86	96.18
2	15.473	5.504	17.749	4.14	3.82
Totals:		132.823	465.013	100.00	100.00

Preparation of 12

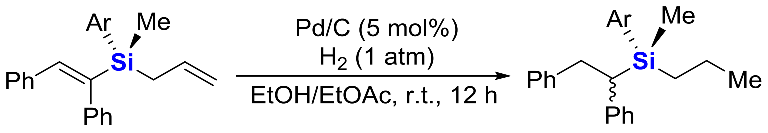

4ca **12**

A mixture of $Pd(OH)_2$ on carbon (10.6 mg, 10 wt% Pd, 10 μmol) and **4ca** (70.9 mg, 0.20 mmol; 92% e.e.) in 1.5 mL of an ethanol/EtOAc mixture (3: 2) was stirred for 12 h at room temperature under H_2 (1 atm.). The catalyst was filtered off through celite with EtOAc, and the solvent was removed under vacuum. The residue was purified *via* silica gel chromatography (petroleum ether) to give **12** as mixture of two non-isolable diastereoisomers in 90% yield (64.5 mg), 1.2: 1 dr, 92% ee. The ee value was measured by HPLC analysis of the corresponding alcohol after boration-oxidation of the allyl group.

^{1}H NMR (400 MHz, Chloroform-*d*): δ 7.37 (dd, *J* = 7.4, 1.5 Hz, 1H), 7.27 (dd, 1H), 7.23 – 7.14 (m, 2H), 7.12 – 6.94 (m, 11H), 6.94 – 6.88 (m, 5H), 6.87 – 6.83 (m, 2H), 6.83 – 6.76 (m, 2H), 3.05 – 2.93 (m, 2H), 2.90 – 2.82 (m, 1H), 2.78 – 2.58 (m, 2H), 2.28 (s, 3H), 2.21 (s, 2H), 1.29 – 1.20 (m, 1H), 1.13 – 0.94 (m, 1H), 0.89 (t, *J* = 7.2 Hz, 2H), 0.83 – 0.68 (m, 5H), 0.61 – 0.47 (m, 1H), 0.29 (s, 2H), 0.23 (s, 2H).

^{13}C NMR (101 MHz, Chloroform-*d*): δ144.1, 144.0, 142.4, 142.30, 142.26, 135.8, 135.7, 135.1, 134.8, 130.2, 130.1, 129.43, 129.35, 128.53, 128.47, 128.3, 128.2, 128.10, 128.08, 128.0, 127.9, 125.63, 125.57, 125.0, 124.8, 124.7, 124.6, 37.9, 37.6, 35.7, 35.6, 23.50, 23.49, 18.7, 18.4, 17.7, 17.5, 16.7, 16.2, -4.3, -5.5.

HRMS (ESI): Calculated for $[C_{25}H_{30}Si, M+Na]^+$: 381.2009. Found: 381.2012.

HPLC: Daicel chiralpak OD-3 column, *n*-hexane/*i*-PrOH = 90/10, 0.5 mL/min, 254 nm UV detector, t (minor) = 16.5 min, t (major) = 23.6 min.

<rac-12-OH>

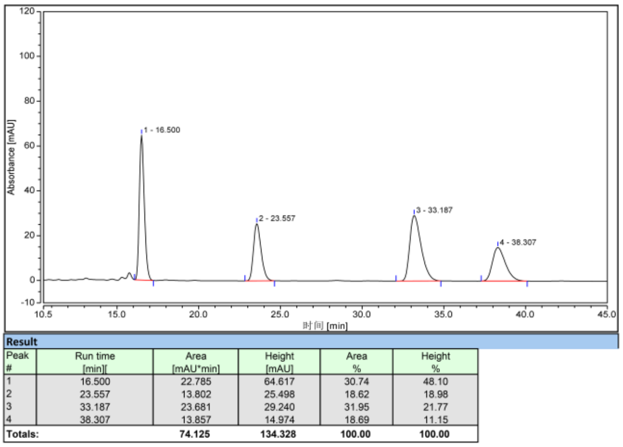

Result

Peak #	Run time [min][	Area [mAU*min]	Height [mAU]	Area %	Height %
1	16.500	22.785	64.617	30.74	48.10
2	23.557	13.802	25.498	18.62	18.98
3	33.187	23.681	29.240	31.95	21.77
4	38.307	13.857	14.974	18.69	11.15
**Totals:**		**74.125**	**134.328**	**100.00**	**100.00**

<chiral-12-OH>

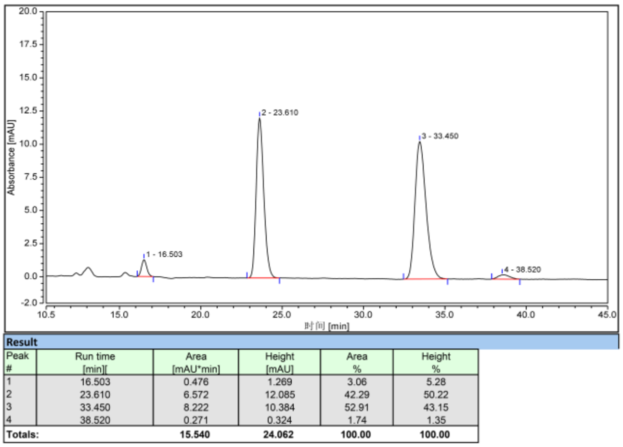

Result

Peak #	Run time [min][	Area [mAU*min]	Height [mAU]	Area %	Height %
1	16.503	0.476	1.269	3.06	5.28
2	23.610	6.572	12.085	42.29	50.22
3	33.450	8.222	10.384	52.91	43.15
4	38.520	0.271	0.324	1.74	1.35
**Totals:**		**15.540**	**24.062**	**100.00**	**100.00**

Preparation of 13

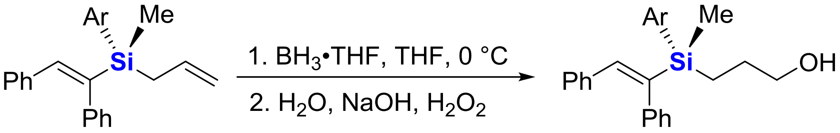

4ca **13**

An oven-dried 8 mL glass vial equipped with a stir bar was charged with **4ca** (70.9 mg, 0.20 mmol; 92% ee) and THF (0.5 mL). The solution was then cooled to 0 ℃. BH_3 (1.0 M in THF, 88 μL, 0.088 mmol, 0.44 equiv.) was added to the vial dropwise. The mixture was allowed to stir at 0 ℃ for 1 h. Then deionized water (30 μL), NaOH (0.2 mL, 2.5 M aqueous solution, 0.5 mmol, 2.5 equiv.), and H_2O_2 (60 μL, 30% aqueous solution, 0.5 mmol, 2.5 equiv.) were added dropwise to the vial sequentially. The mixture was allowed to stir for 1 h at room temperature afterwards. Once complete, the reaction was quenched with water and extracted with EtOAc. The combined organic layers were dried over Na_2SO_4 and concentrated *in vacuo*. The residue was purified *via* silica gel chromatography (petroleum ether/EtOAc = 5: 1), and **13** was obtained in 73% yield (54.4 mg) as colorless oil, ee = 92%, Optical rotation: $[\alpha]_D^{25}$ -4.7 (c = 1.0, CH_2Cl_2).

^{1}H NMR (400 MHz, $CDCl_3$): δ 7.46 (d, *J* = 7.3 Hz, 1H), 7.32 (m, 1H), 7.25 – 7.13 (m, 5H), 7.11 – 7.04 (m, 3H), 6.96 – 6.87 (m, 4H), 6.79 (s, 1H), 3.57 (t, *J* = 6.6 Hz, 2H), 2.51 (s, 3H), 1.55 (m, 2H), 1.00 (m, 1H), 0.93 – 0.86 (m, 1H), 0.43 (s, 3H).

^{13}C NMR (101 MHz, $CDCl_3$): δ 144.3, 144.1, 142.4, 139.8, 137.4, 135.8, 134.7, 130.2, 129.8, 129.7, 128.7, 128.0, 127.7, 127.3, 126.0, 125.1, 65.8, 27.3, 23.4, 9.7, -3.7.

HRMS (ESI): Calculated for $C_{29}H_{34}Si$ [$C_{25}H_{28}OSi$, M+ Na] $^+$: 395.1802. Found: 395.1805.

HPLC: Daicel chiralpak OD-3 column, *n*-hexane/*i*-PrOH = 90: 10, 0.5 mL/min, 254 nm UV detector, t(major) = 13.1 min, t(minor) =15.0 min.

<rac-**13**>

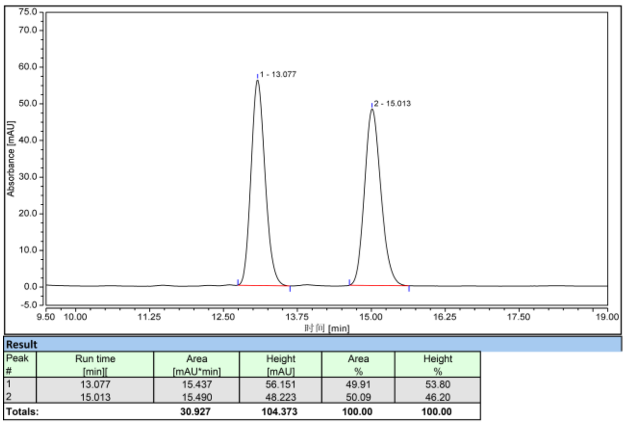

Result

Peak #	Run time [min]	Area [mAU*min]	Height [mAU]	Area %	Height %
1	13.077	15.437	56.151	49.91	53.80
2	15.013	15.490	48.223	50.09	46.20
Totals:		**30.927**	**104.373**	**100.00**	**100.00**

<chiral-**13**>

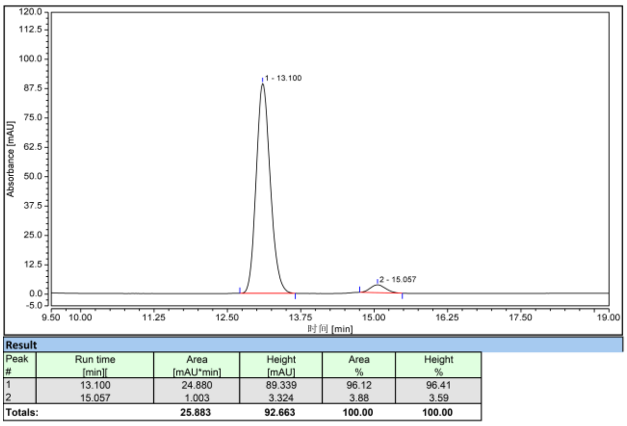

Result

Peak #	Run time [min]	Area [mAU*min]	Height [mAU]	Area %	Height %
1	13.100	24.880	89.339	96.12	96.41
2	15.057	1.003	3.324	3.88	3.59
Totals:		**25.883**	**92.663**	**100.00**	**100.00**

3. Supplementary Discussion

Determination of absolute configuration by ECD experiments

ECD experiments: ECD and UV spectra of **4cb**, at a concentration of 4×10^{-5} mol/L in acetonitrile, were recorded in a 1 mm pathlength quartz cuvette, using a Bio-Logic MOS-450 spectrometer. The experimental conditions were as follows: bandwidth, 1 nm; wavelength range, 210-360 nm; wavelength step size, 1 nm; time-per-point, 1.0 s; temperature, 25 ℃. Acetonitrile was measured under the same conditions to obtain baseline.

Computational studies: (Time-dependent) density functional theory ((TD-)DFT) calculations were performed in the Gaussian 16 software package[7] utilizing initial conformers of **4cb** generated by iterative meta-dynamics genetic z-matrix crossing (iMTD-GC) conformation search[8] in the CREST code.[9-10] All conformers in 0.0-1.0 kcal/mol were optimized and verified by vibrational frequency calculations with B3LYP/6-31G(d).[11] Rotatory strengths in velocity and length representations (R_{vel} and R_{len}), oscillator strengths and excitation energies of the 100 lowest electronic transitions were calculated by TD-M06-2X/6-311G(d).[12] ECD and UV spectra were obtained by broadening with a half width at half height of 0.3 eV, and Boltzmann-weighted spectra were generated in Multiwfn.[13] Molecular structures were visualized in CYLview.[14]

Supplementary Figure 1 shows the low-energy conformers considered in TD-DFT calculations. Supplementary Figure 2 and 3 display the observed and calculated ECD and UV spectra of **4cb** over the range of 200-320 nm. With the theory-experiment agreement, it is reasonable to determine the charity with TD-DFT calculations. The experimental ECD spectrum has a positive Cotton effect at 214 nm, which can be assigned to the calculated band of (*S*)-**4cb** at 202 nm. The observed negative Cotton effect at 229 nm is assigned to the calculated band of (*S*)-**4cb** at 218 nm. The experimental ECD spectrum has a positive Cotton effect at 285 nm,

which can be ascribed to the calculated band of (*S*)-**4cb** at 282 nm. Based on these results, **4cb** was inferred to have an *S* configuration.

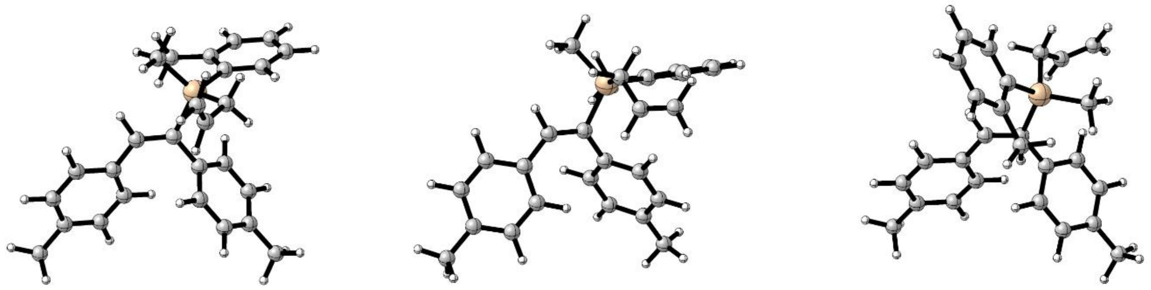

4cb-1 (8.6%) **4cb-2** (32.4%) **4cb-3** (59.0%)

Supplementary Figure 1. Low-energy conformers of **4cb** obtained through iMTD-GC search and DFT validations.

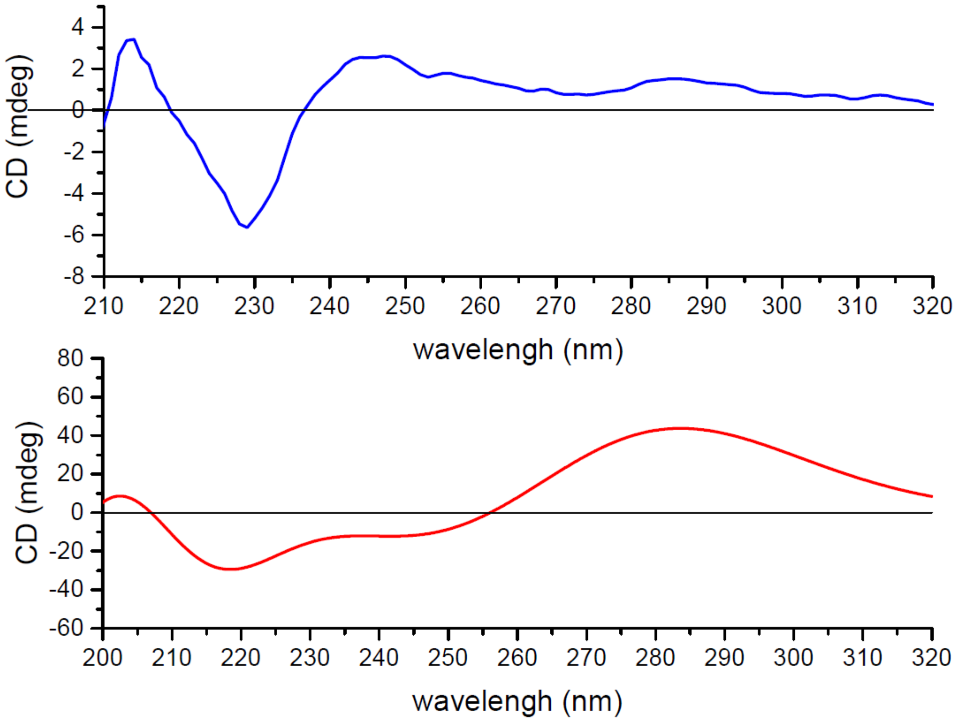

Supplementary Figure 2. Comparison of the experimental (top) and calculated (bottom) ECD spectra of **4cb**.

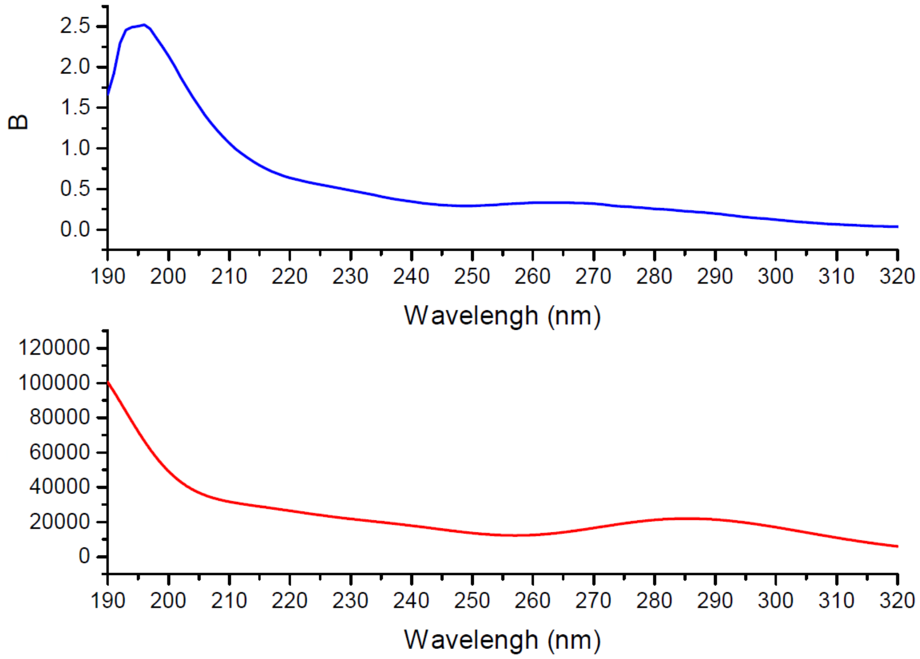

Supplementary Figure 3. Comparison of the experimental (top) and calculated (bottom) UV spectra of **4cb**.

Mechanistic experiments

Investigation of Ni(0)-alkyne complex as the Intermediates for the cycloaddition reaction

Experimental procedure: In a nitrogen-filled glove-box, Ni(cod)$_2$ (5.8 mg, 0.02 mmol), IPr•HCl (10.2 mg, 0.024 mmol), LiO^{t}Bu (3.2 mg, 0.04 mmol) and toluene-d_8 (0.5 mL) were added to an oven-dried 8 mL glass vial, The mixture was allowed to stir under ambient temperature for 15 min. Then **2f** (0.2 mmol) was added. The resulting mixture was used directly for **^{19}F-NMR** experiment (Supplementary Figure S4).

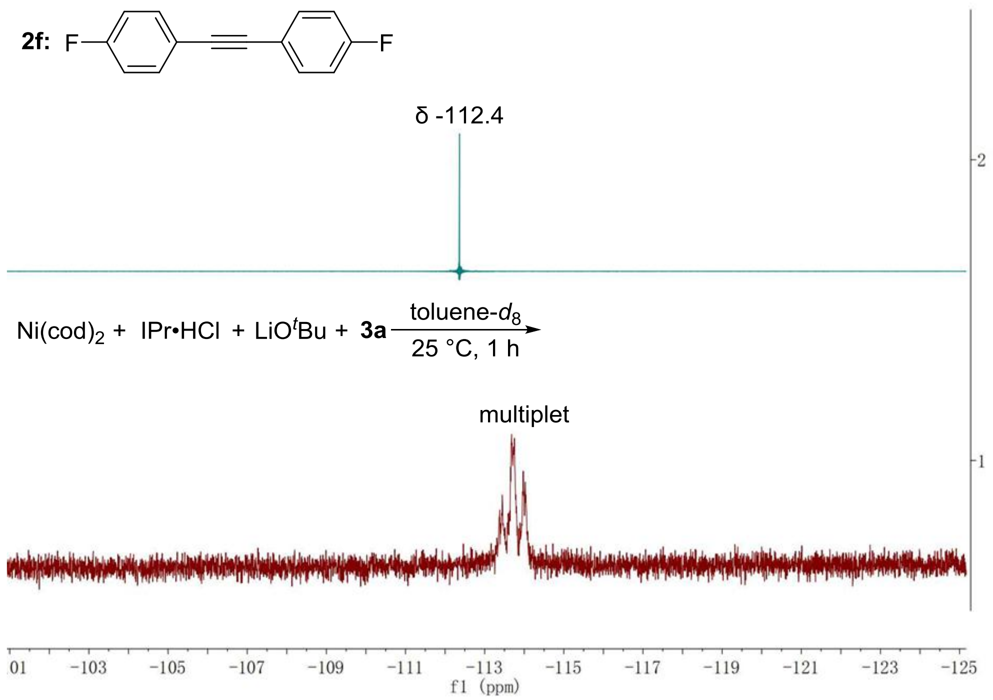

Supplementary Figure 4: ^{19}F-NMR experiment of the reaction mixture under Nickel-NHC catalysis

Investigation of Ni(0)-alkyne complex as the Intermediates for the ring-openning reaction

Experimental procedure: In a nitrogen-filled glove-box, Ni(PPh$_3$)$_4$ (46.3 mg, 0.05 mmol), diphenylacetylene (46.3 mg, 0.05 mmol) and toluene-d_8 (0.5 mL) were added to an oven-dried 8 mL glass vial, the mixture was allowed to stir for 30 min under ambient temperature. The resulting mixture was used directly for **^{31}P-NMR** experiment (Supplementary Figure S5).

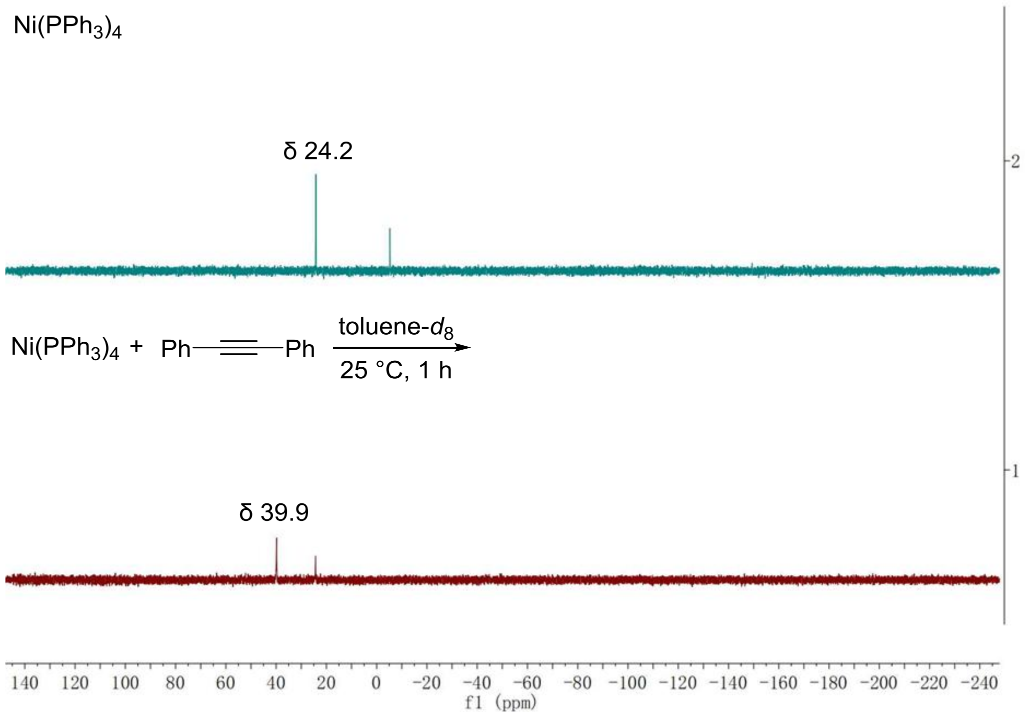

Supplementary Figure 5: ^{31}P-NMR experiment of the reaction mixture under Nickel-Phosphine catalysis

Deuterium-labeling experiment

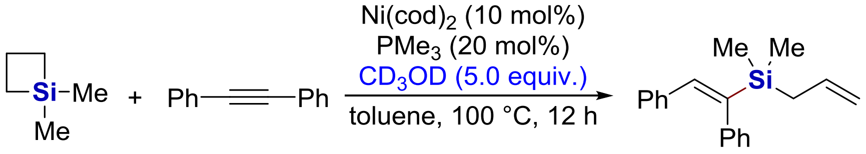

1a **2a** **4aa**, 78% yield

Experimental procedure: In a nitrogen-filled glove-box, an oven-dried 8 mL glass vial equipped with a stir bar was charged with Ni(cod)$_2$ (2.8 mg, 0.01 mmol), PMe$_3$ (20 μL, 1.0 M in THF, 0.02 mmol) and toluene (1.0 mL). The mixture was allowed to stir under ambient temperature for 15 min. Then silacyclobutane **1a** (30 mg, 0.3 mmol, 3.0 equiv.), diphenylacetylene (17.8 mg, 0.1 mmol) and CD_3OD (9.0 μL, 0.5 mmol, 5.0 equiv.) were added sequentially. The vial was then sealed with a PTFE cap, removed from the glove box and stirred at 100 ℃ for 12 h. After being cooled to room temperature, the solvent was removed under vacuum and the residue was subjected to silica gel chromatography (petroleum ether) to give the corresponding product in 78% yield. ^{1}H NMR analysis showed that no deuterated **4aa** was observed.

^{1}H NMR (400 MHz, CDCl$_3$): δ 7.50 – 7.44 (m, 2H), 7.41 – 7.34 (m, 1H), 7.27 – 7.23 (m, 3H), 7.18 – 7.14 (m, 2H), 7.11 (dd, J = 6.8, 3.0 Hz, 2H), 6.97 (s, 1H), 6.00 – 5.87 (m, 1H), 5.08 – 4.96 (m, 2H), 1.84 – 1.74 (m, 2H), 0.31 (s, 6H).

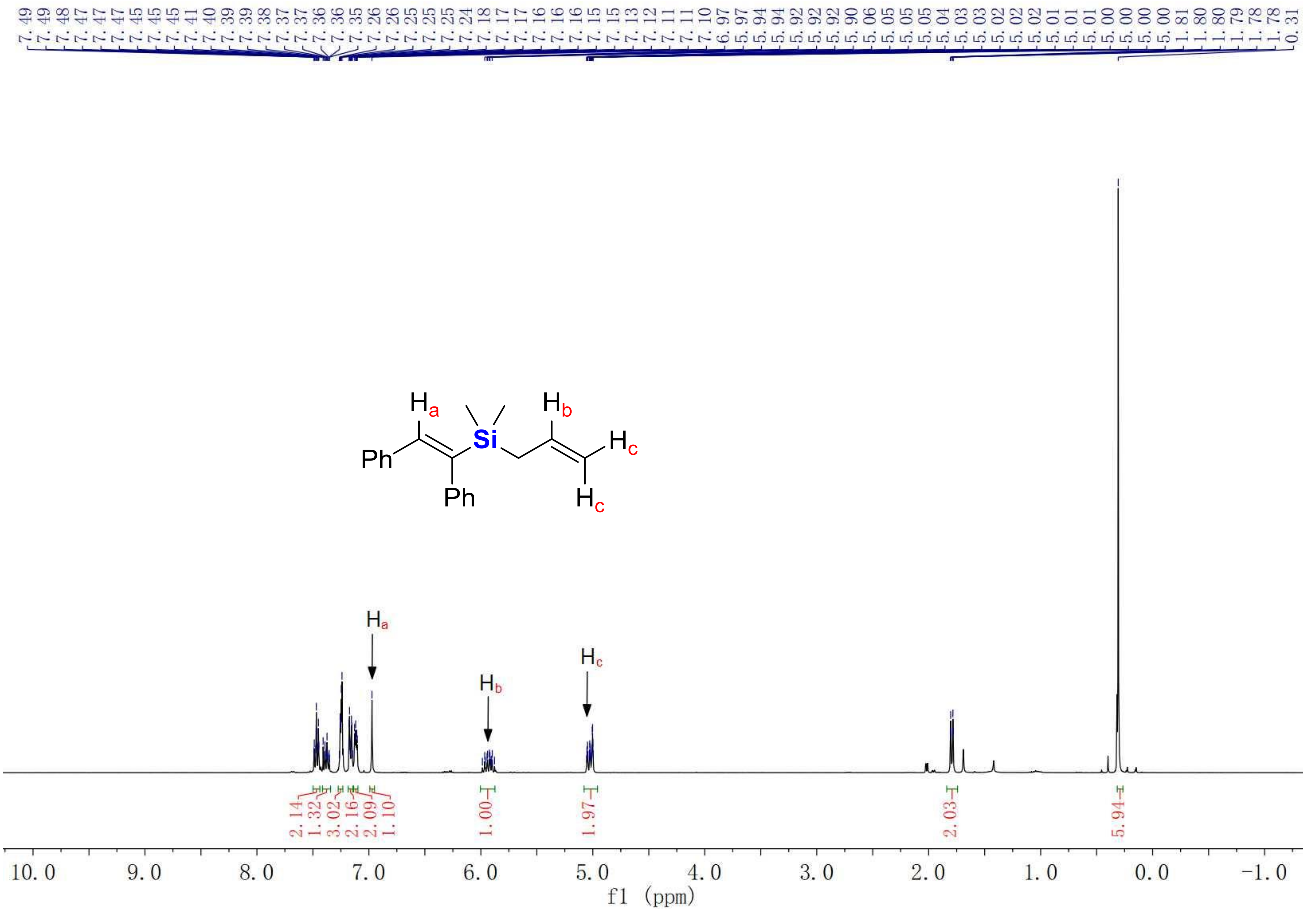

Computational studies

Computational procedure: Geometry optimizations and frequency calculations were carried out in the Gaussian 16 code[7] using the M06-L functional[14] and the def2-SV(P) basis set.[15] Single-point energies were computed with the PBE0-D3(BJ) functional,[16] the def2-TZVP basis set,[15] and the SMD model.[17] Thermochemical quantities (at 1 mol/L unless otherwise specified) were evaluated in GoodVibes[18] with quasi-harmonic approximations.[19] CYLview and PyMOL[20] were employed for visualizations. Ligand-substrate non-covalent interactions were analyzed by the independent gradient model (IGM)[21] implemented in Multiwfn.[13]

Ligand-controlled divergent reactions between 1a and 2a: Supplementary Figure 6 provides further details for the results in **Figure 5** of the manuscript. In addition to the main text discussion, we also evaluated a RE transition state **P-TS$_{\mathbf{RE}}$** with monocoordination of PMe_3. These computations use Ni-silacycloheptene species **B** as the energy zero. Since the barrier via **P-TS$_{\mathbf{RE}}$** is 21.3 kcal/mol, which is appreciably higher than **P2-TS$_{\mathbf{RE}}$** (16.4 kcal/mol), the latter is expected to be the main RE pathway and is therefore discussed. It should be noted that the reported 13.7 and 21.3 kcal/mol for **P-TS$_{\mathbf{LLHT}}$** and **P-TS$_{\mathbf{RE}}$** involve a concentration correction of PMe_3 to 0.01 M, which represents the remaining PMe_3 after monocoordination to Ni species. Because a correction

to the maximum PMe_3 concentration (0.02 M) yields 14.2 and 21.9 kcal/mol respectively for **P-TS$_{LLHT}$** and **P-TS$_{RE}$**, the conclusions are consistent using both corrections.

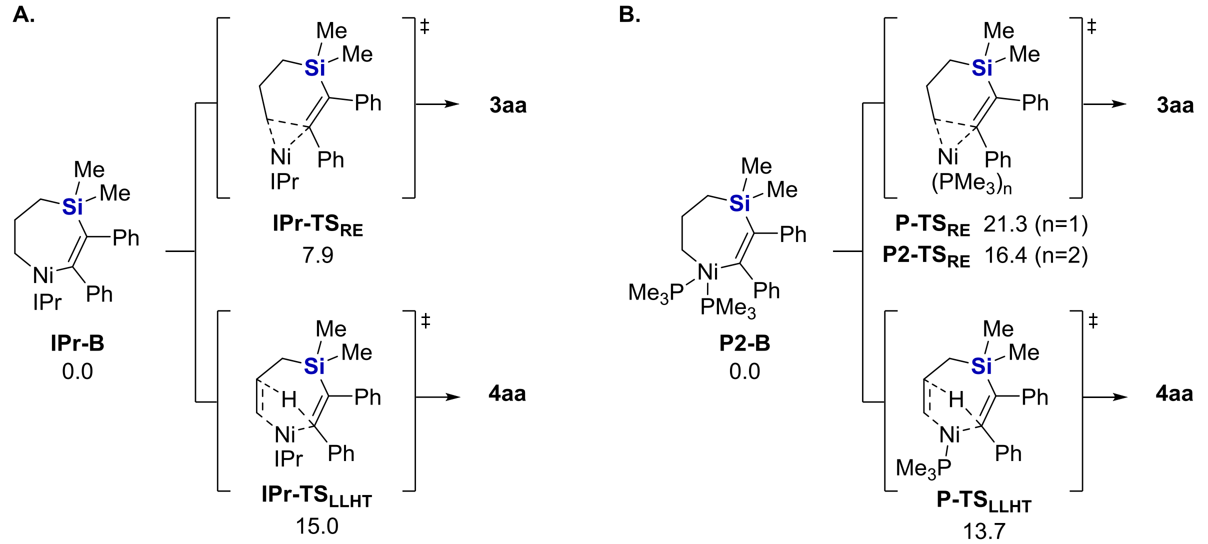

Supplementary Figure 6. Supplementary details for the ligand-controlled reactivity of **1a** with **2a**. A concentration correction to 0.01 M is applied to PMe_3. All free energies are in kcal/mol.

DFT studies for the reaction between 5a and 2a: We also studied the reaction of benzosilacyclobutane **5a** with alkyne **2a**. An evaluation of possible catalyst resting states is presented in Supplementary Figure 7.[22] The lowest free energy is observed on **IM1**, a Ni(0) complex involving ligations of diphenylacetylene **2a** and two PMe_3. The coordination by COD and benzosilacyclobutane **5a** is energetically unfavorable. Therefore, **IM1** is used as the energy zero in subsequent calculations.

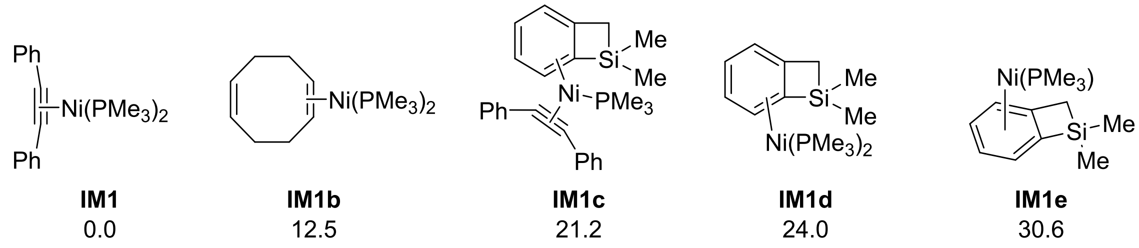

Supplementary Figure 7. Relative free energies of possible Ni(0) resting states (unit: kcal/mol).

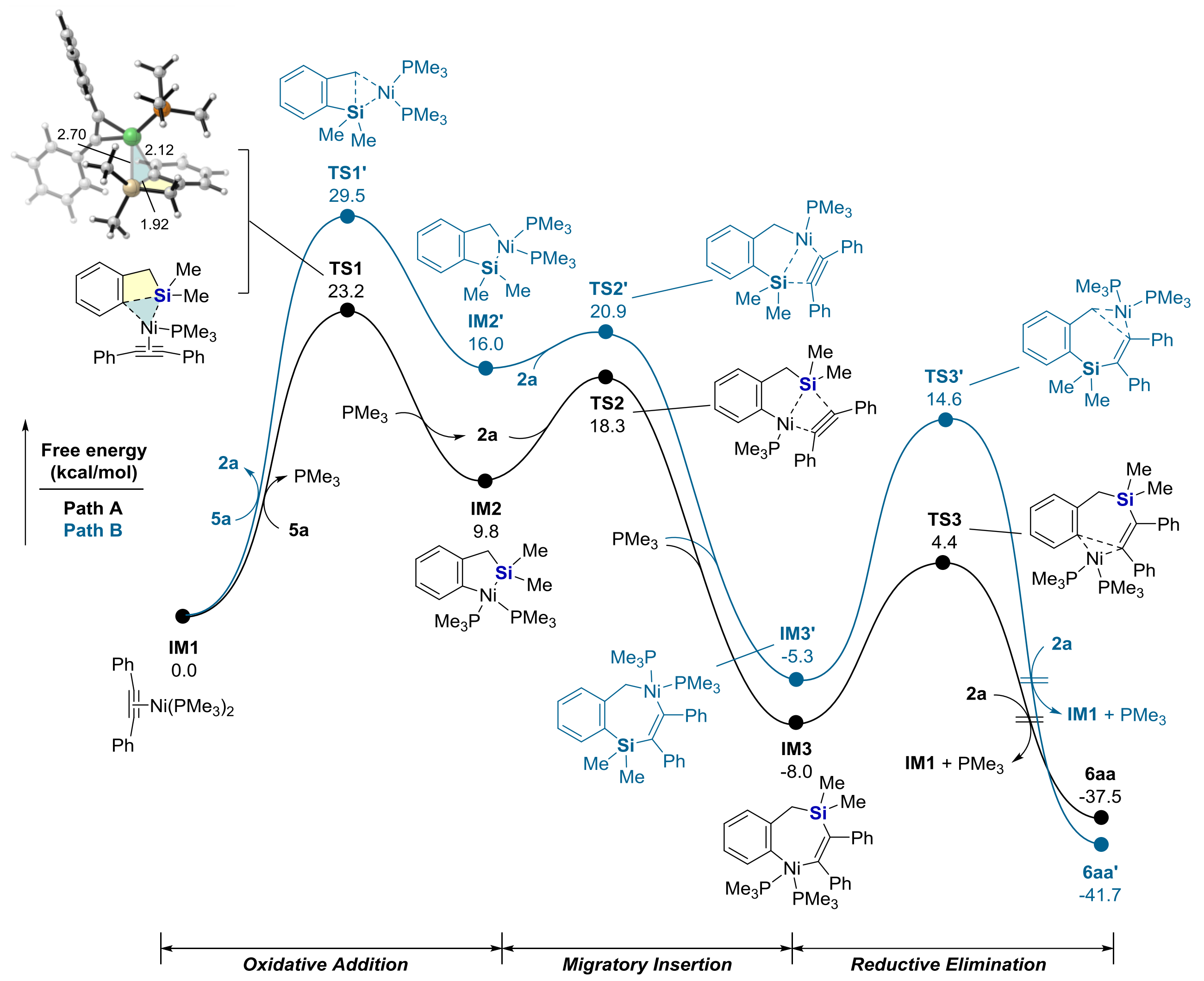

Supplementary Figure 8. Free-energy profile (in kcal/mol) for the cycloaddition of benzosilacyclobutane **5a** with **2a**.

The free-energy profile on display in Supplementary Figure 8 supports a plausible mechanism involving oxidative addition of Ni(0) species with benzosilacyclobutane, migratory insertion of alkyne into Ni–Si bond, and reductive elimination that affords the cycloaddition product. The profile suggests that oxidative addition is the rate-limiting and selectivity-determining step. The DFT computations imply a kinetic preference of 6.3 kcal/mol towards Si–C(sp^2) bond cleavage (i.e., path A) against Si–C(sp^3) cleavage (i.e., path B). Possible factors favoring path A include the smaller steric hindrance over the C(sp^2) center and the Ni…π interactions with the fused benzene ring.

4. Supplementary Figures

NMR spectra

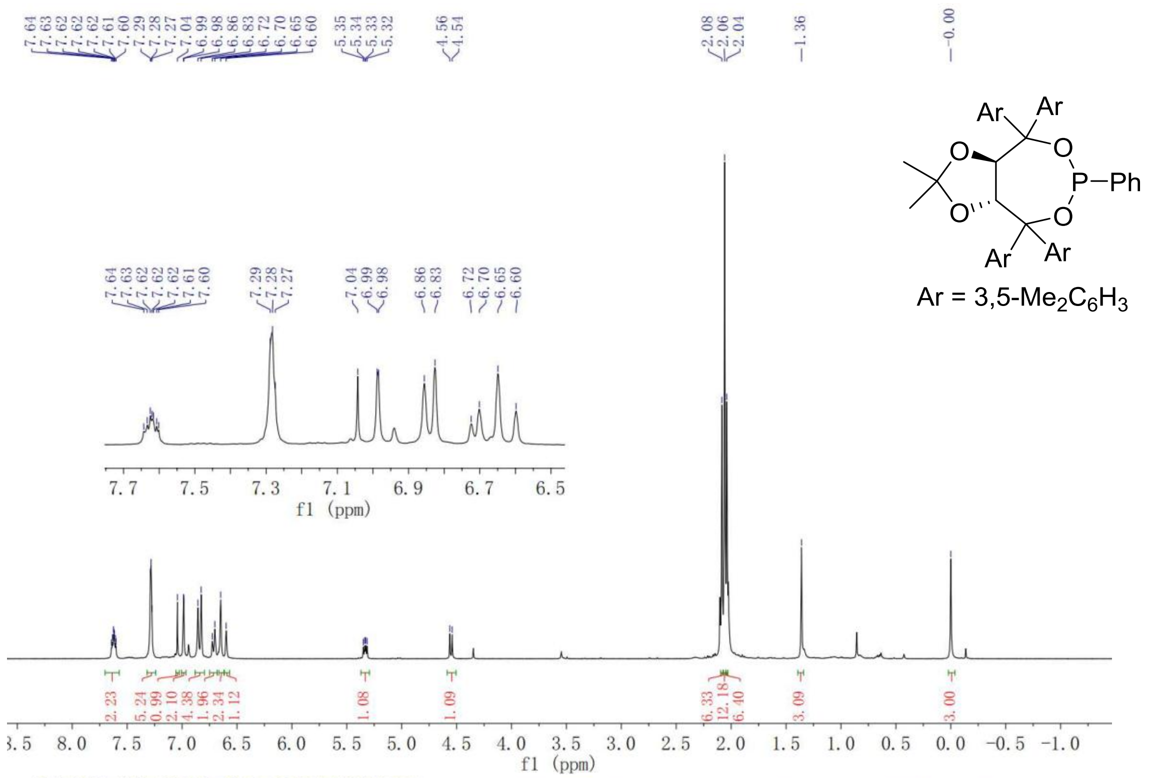

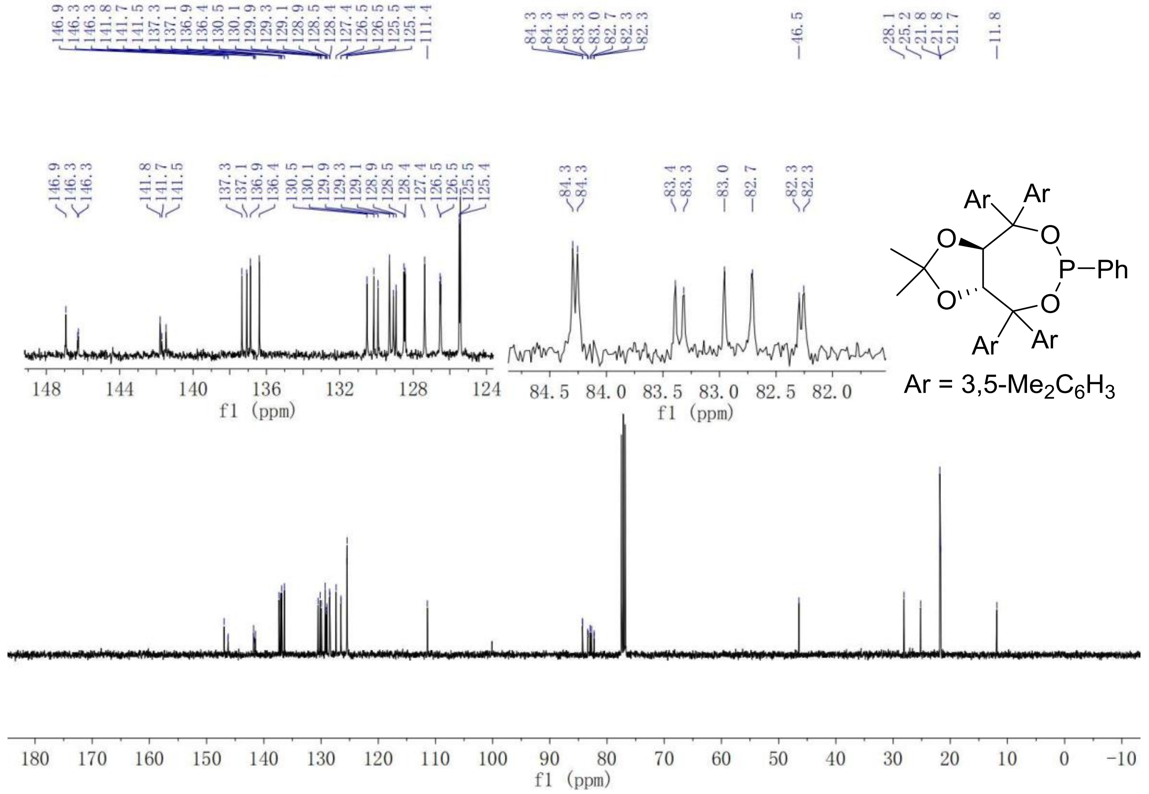

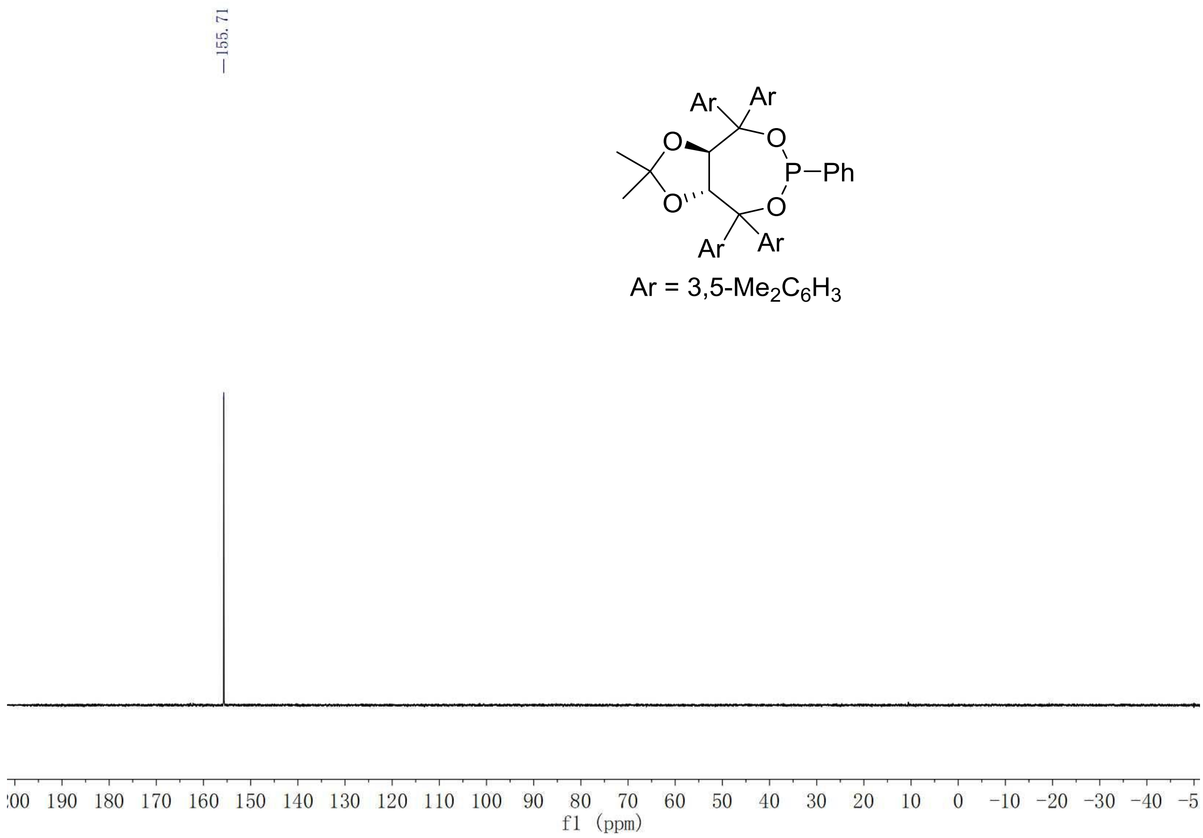

Supplementary Figure 9 ^{1}H, ^{13}C and ^{31}P NMR Spectra for (*R*,*R*)-L20

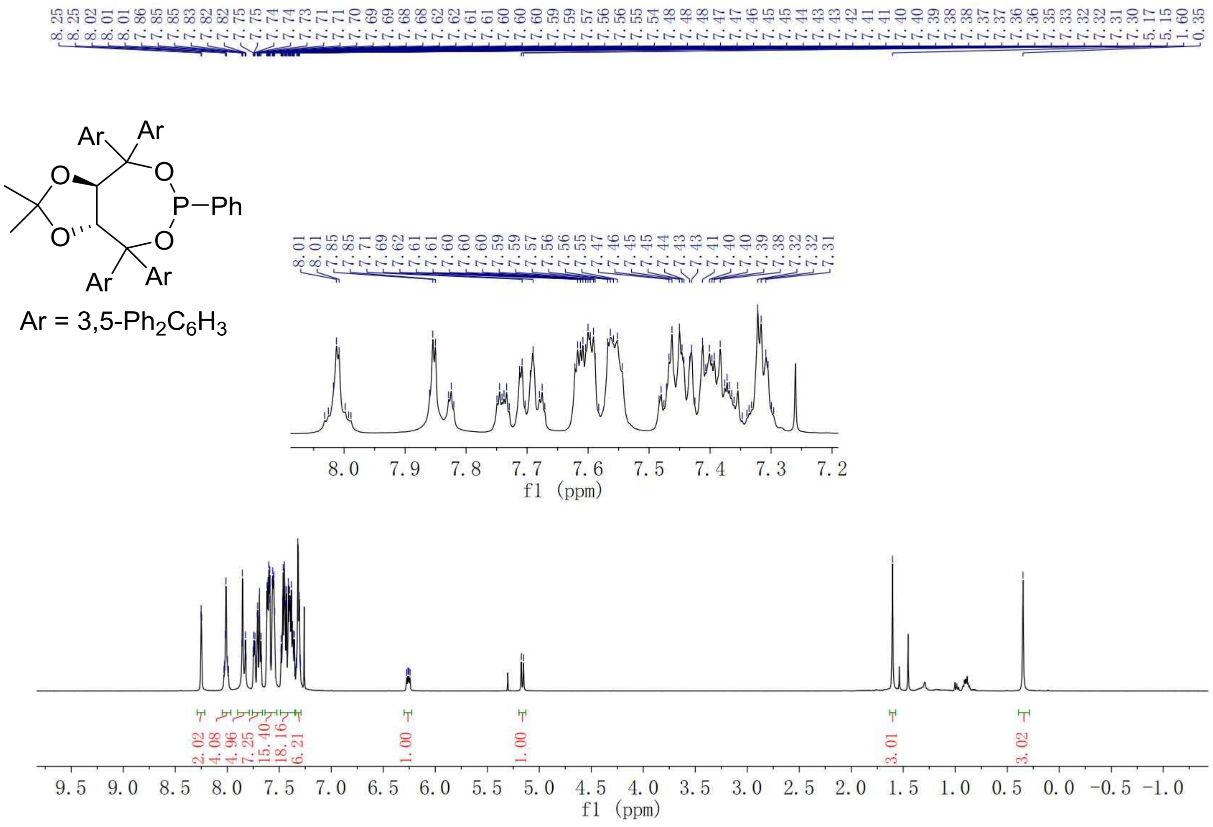

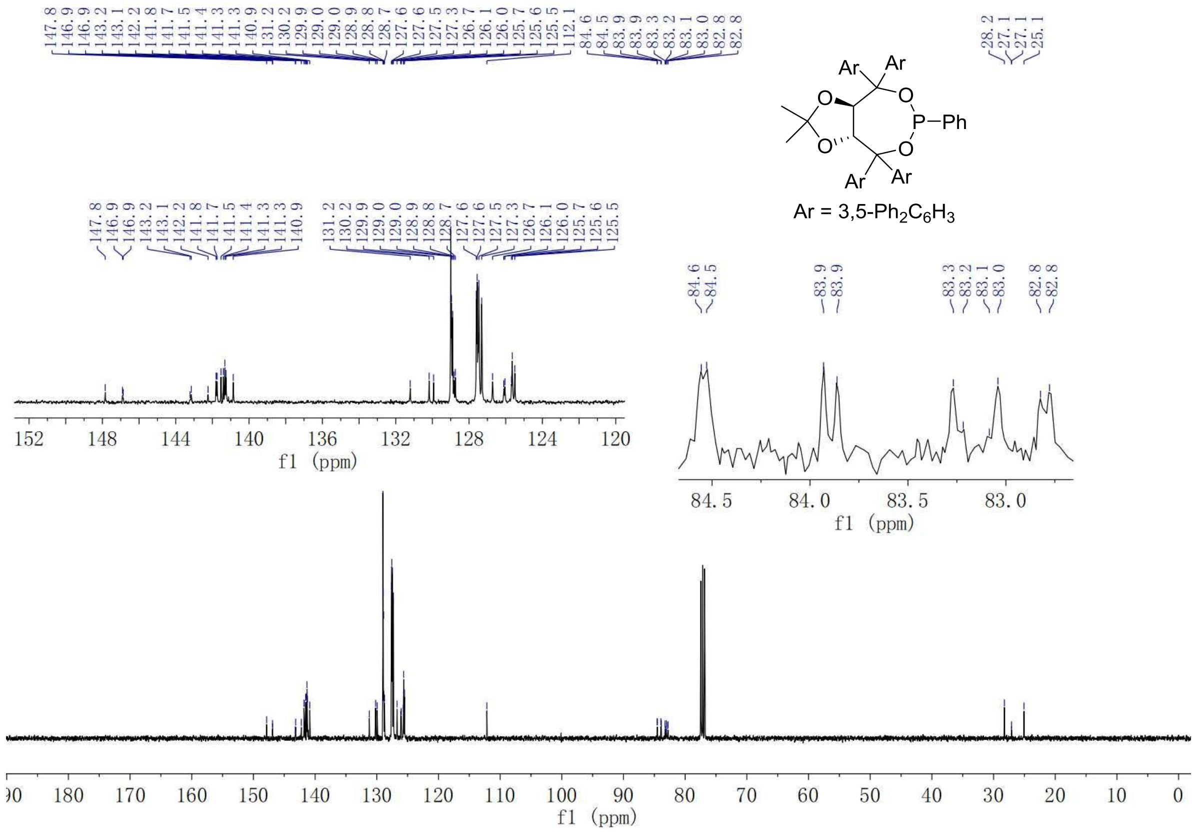

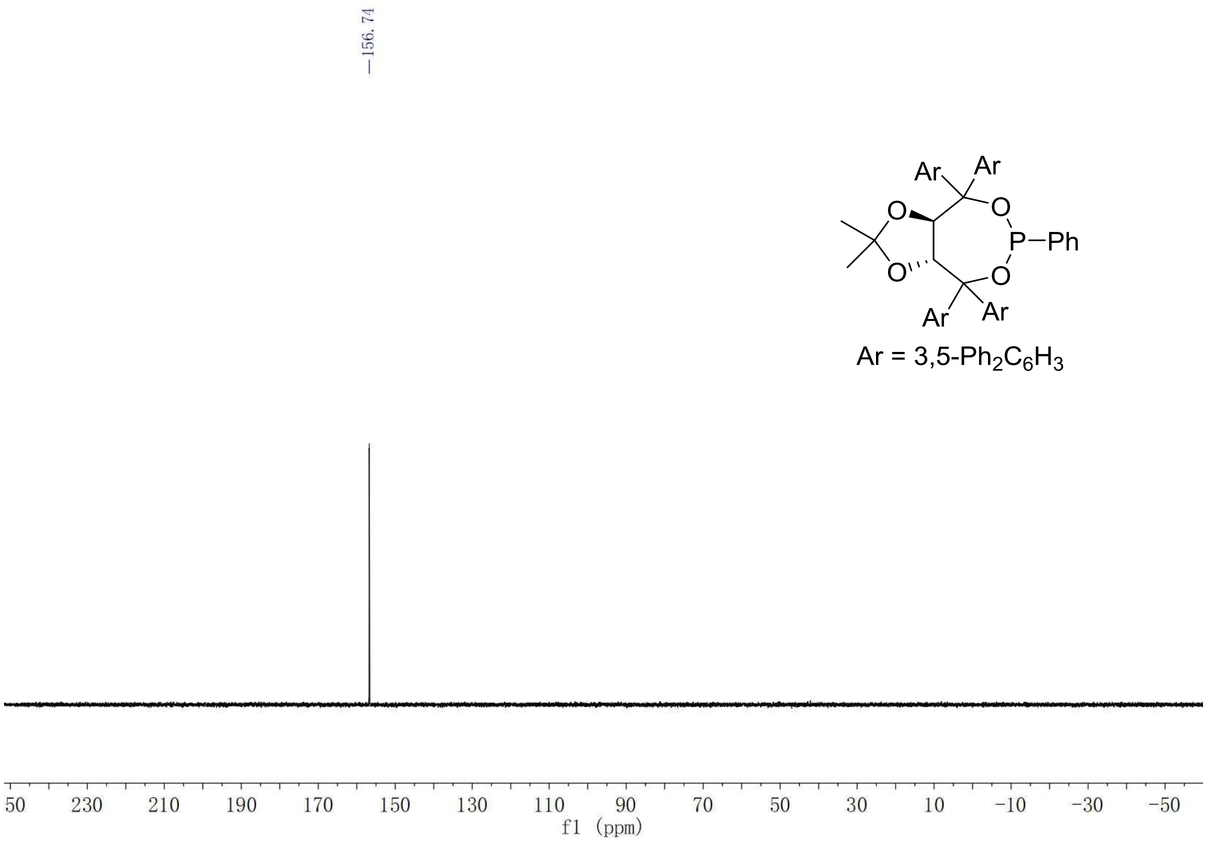

Supplementary Figure 10 ^{1}H, ^{13}C and ^{31}P NMR Spectra for (*R*,*R*)-L23

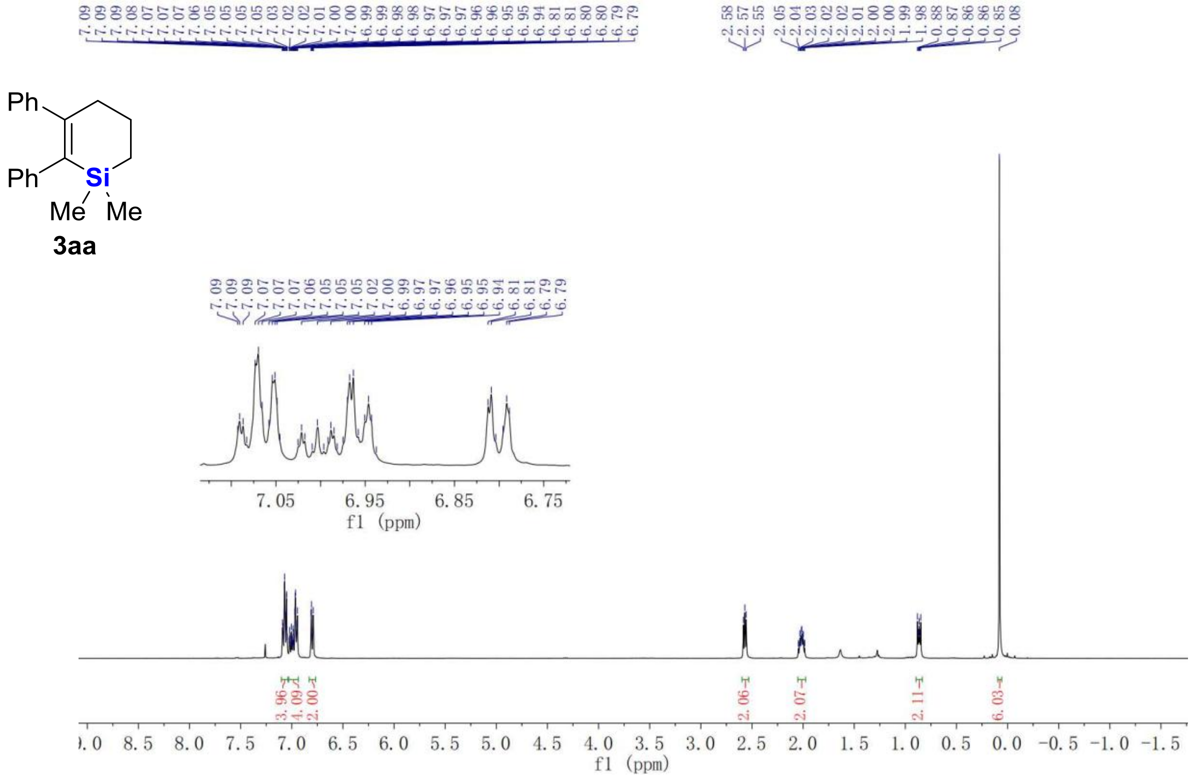

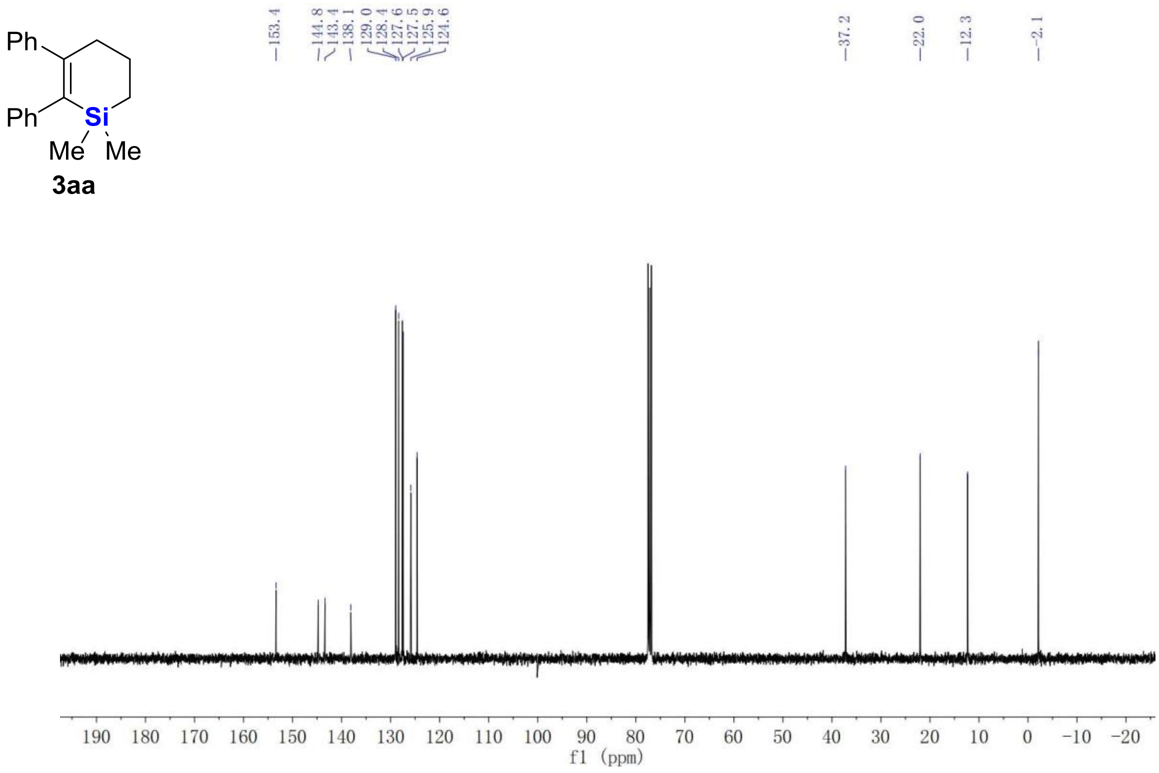

Supplementary Figure 11 ^{1}H and ^{13}C NMR Spectra for compound 3aa

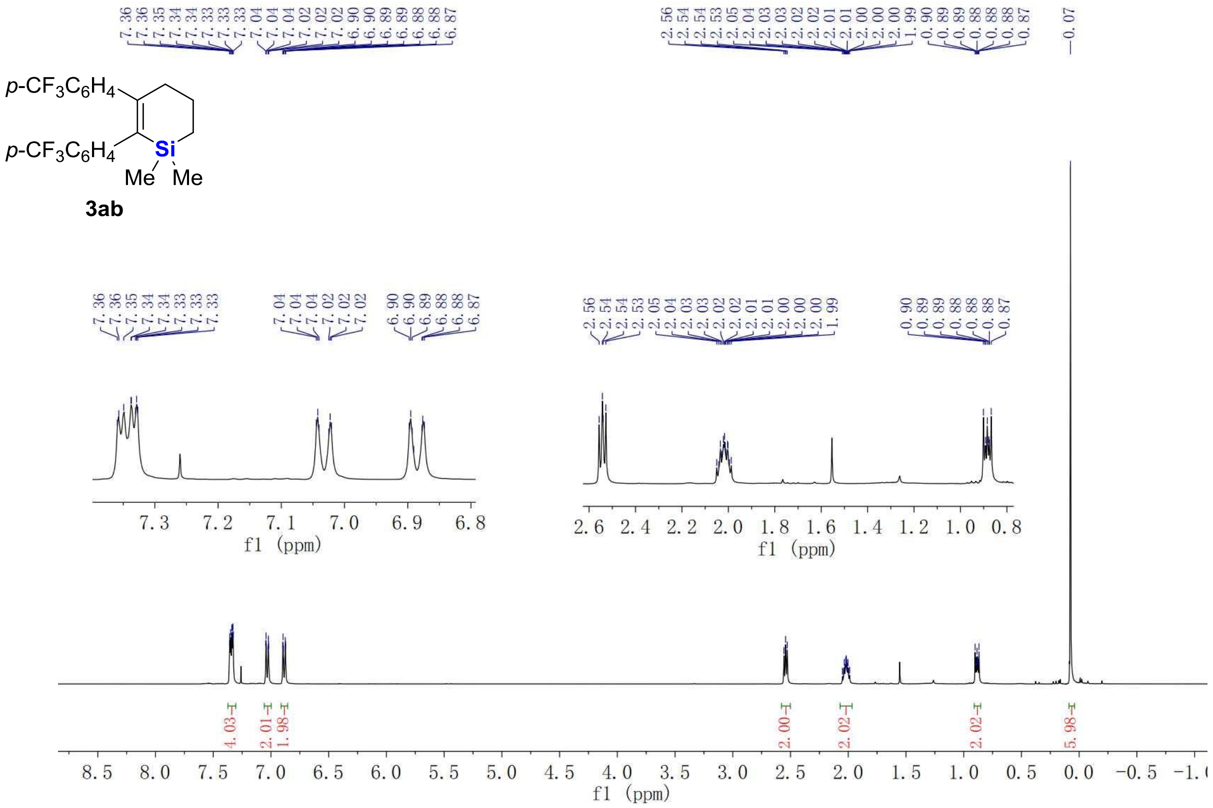
p-CF3C6H4
p-CF3C6H4
Si
Me Me
3ab
f1 (ppm)

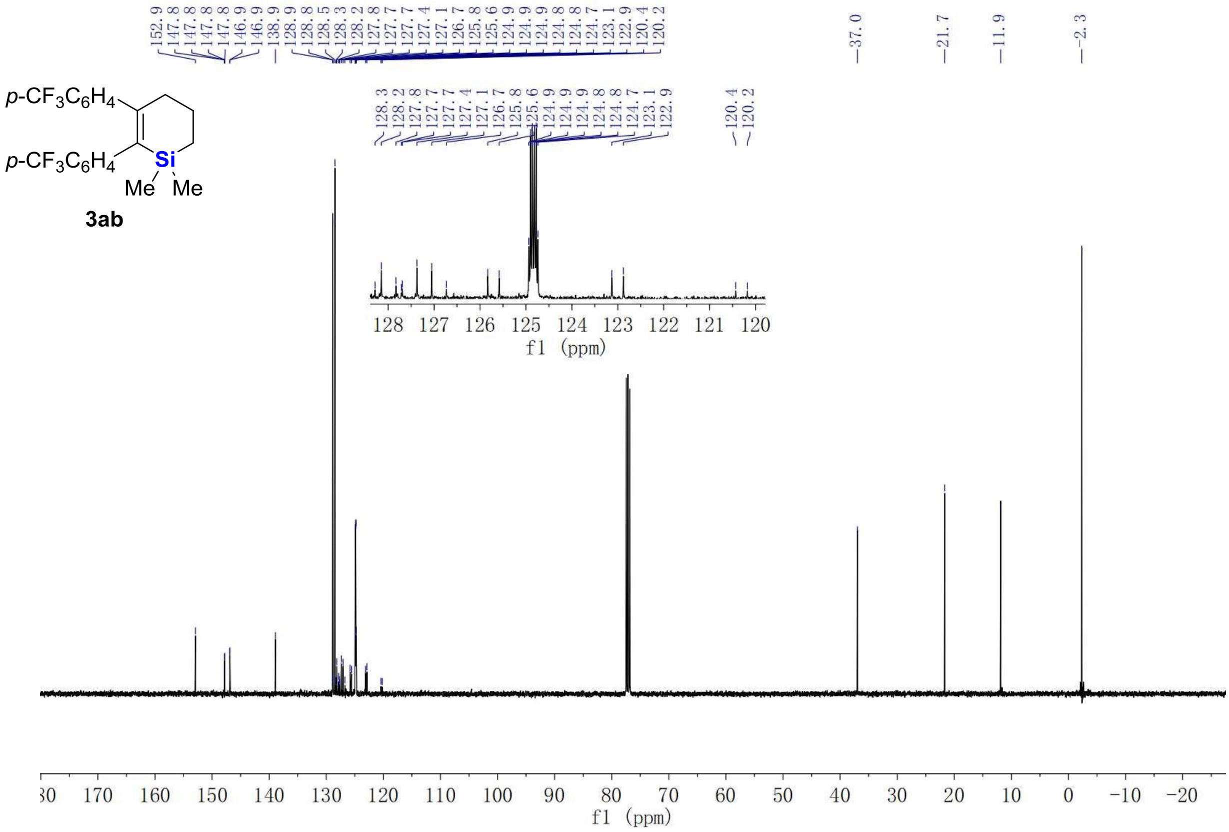
p-CF3C6H4
p-CF3C6H4
Si
Me Me
3ab
f1 (ppm)

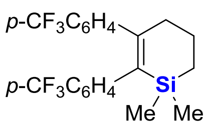

3ab

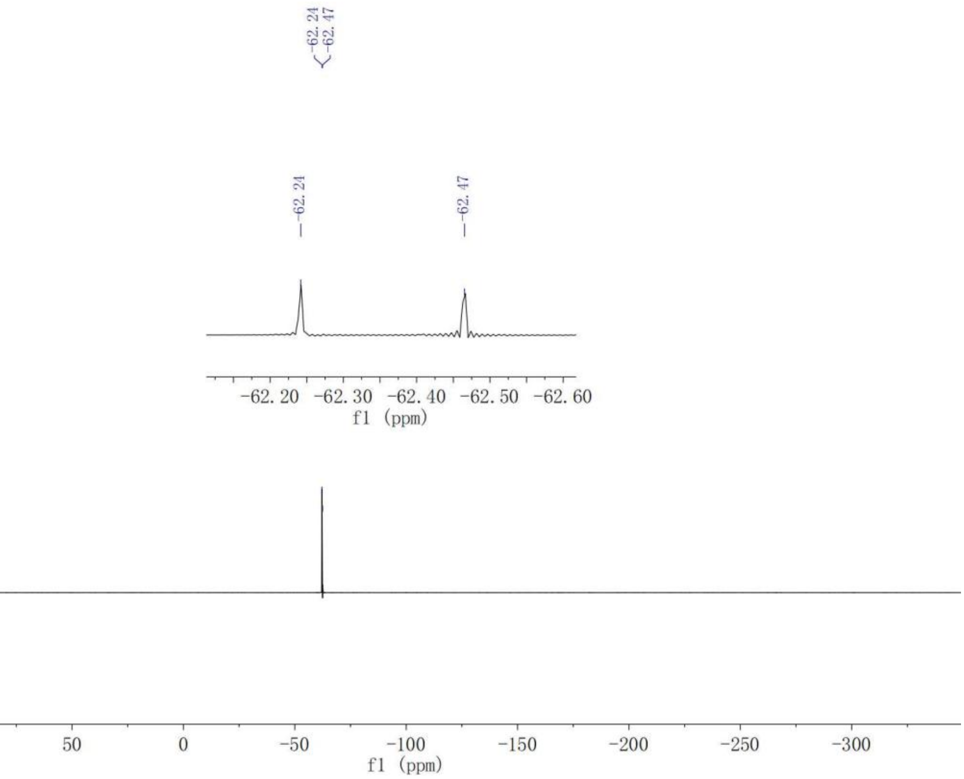

Supplementary Figure 12 ^{1}H, ^{13}C and ^{19}F NMR Spectra for compound 3ab

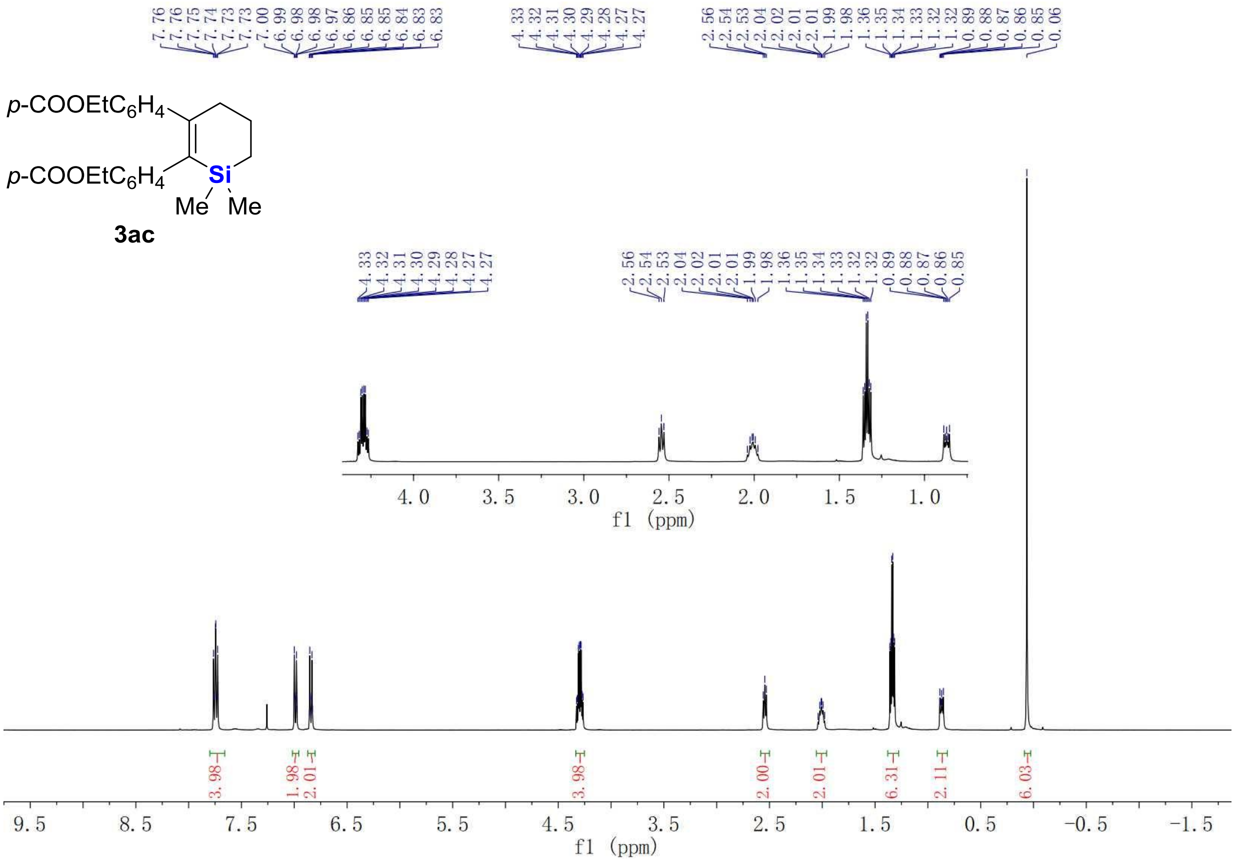

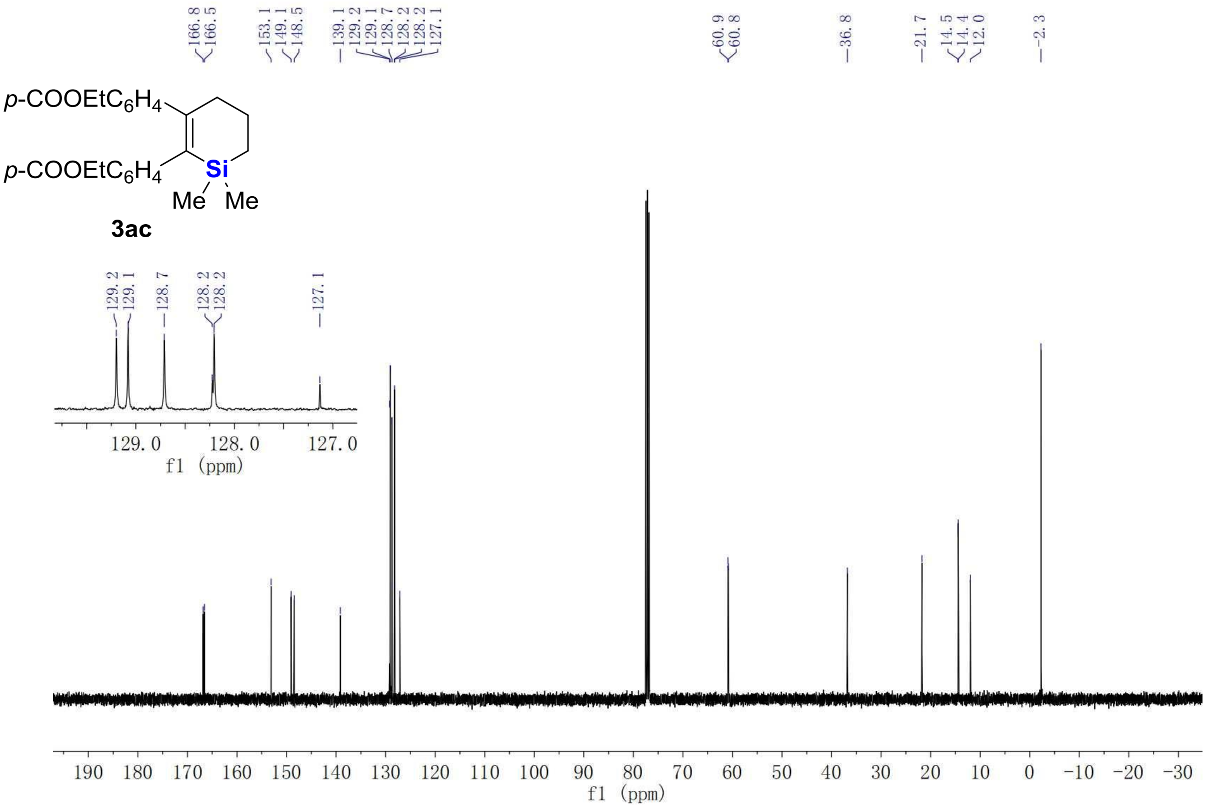

Supplementary Figure 13 ^{1}H and ^{13}C NMR Spectra for compound 3ac

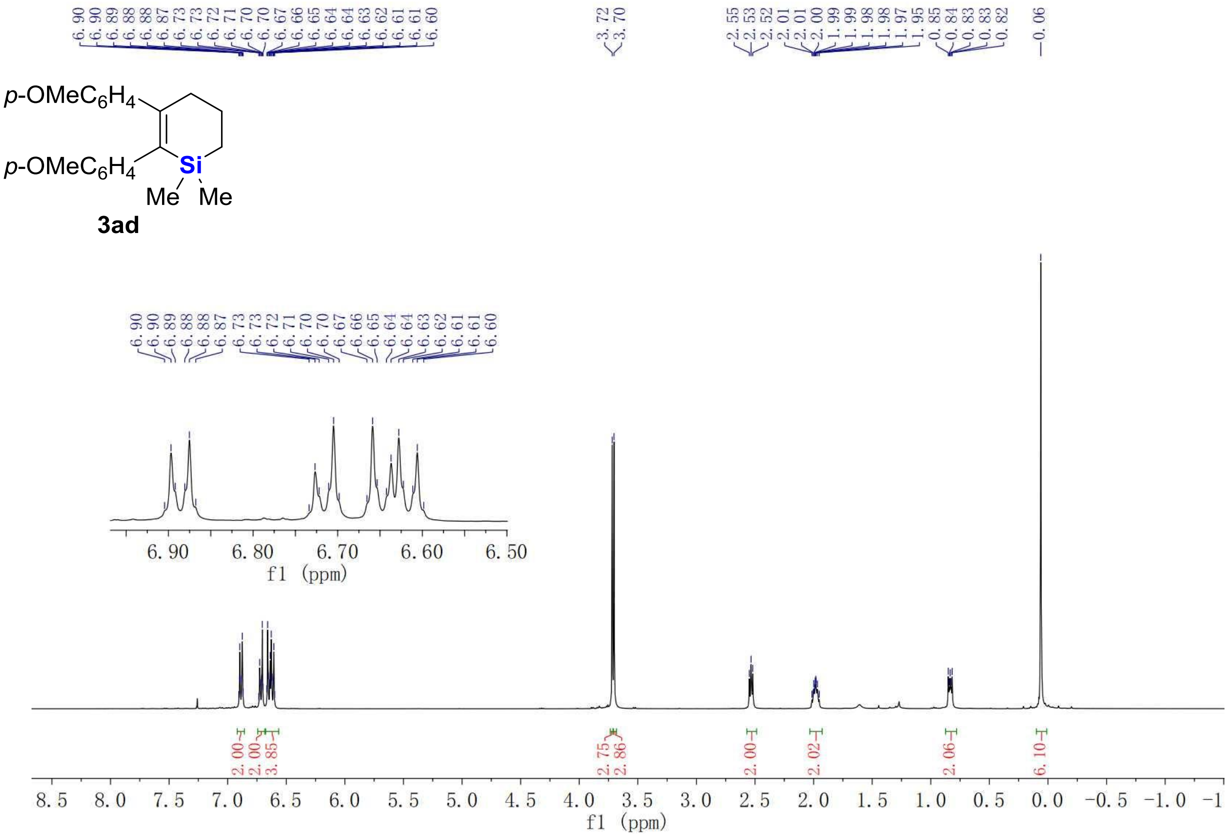

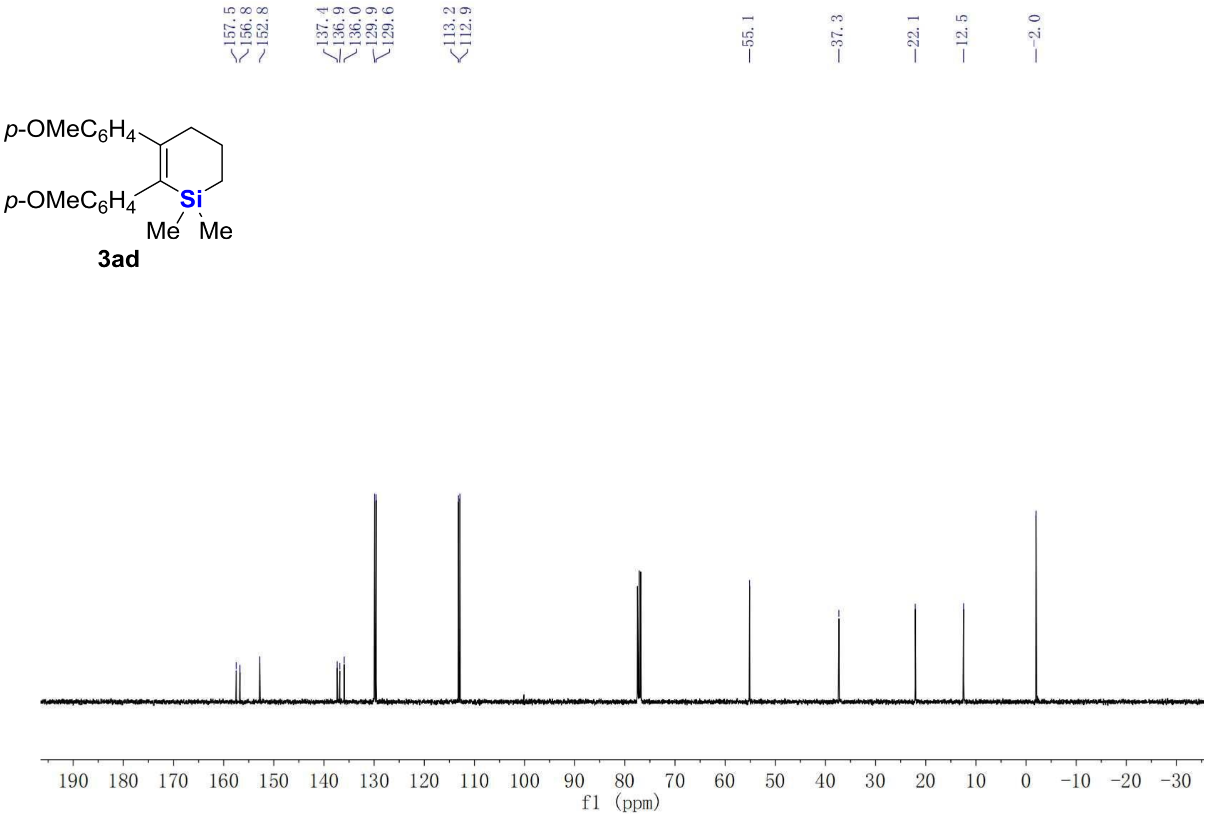

Supplementary Figure 14 ^{1}H and ^{13}C NMR Spectra for compound 3ad

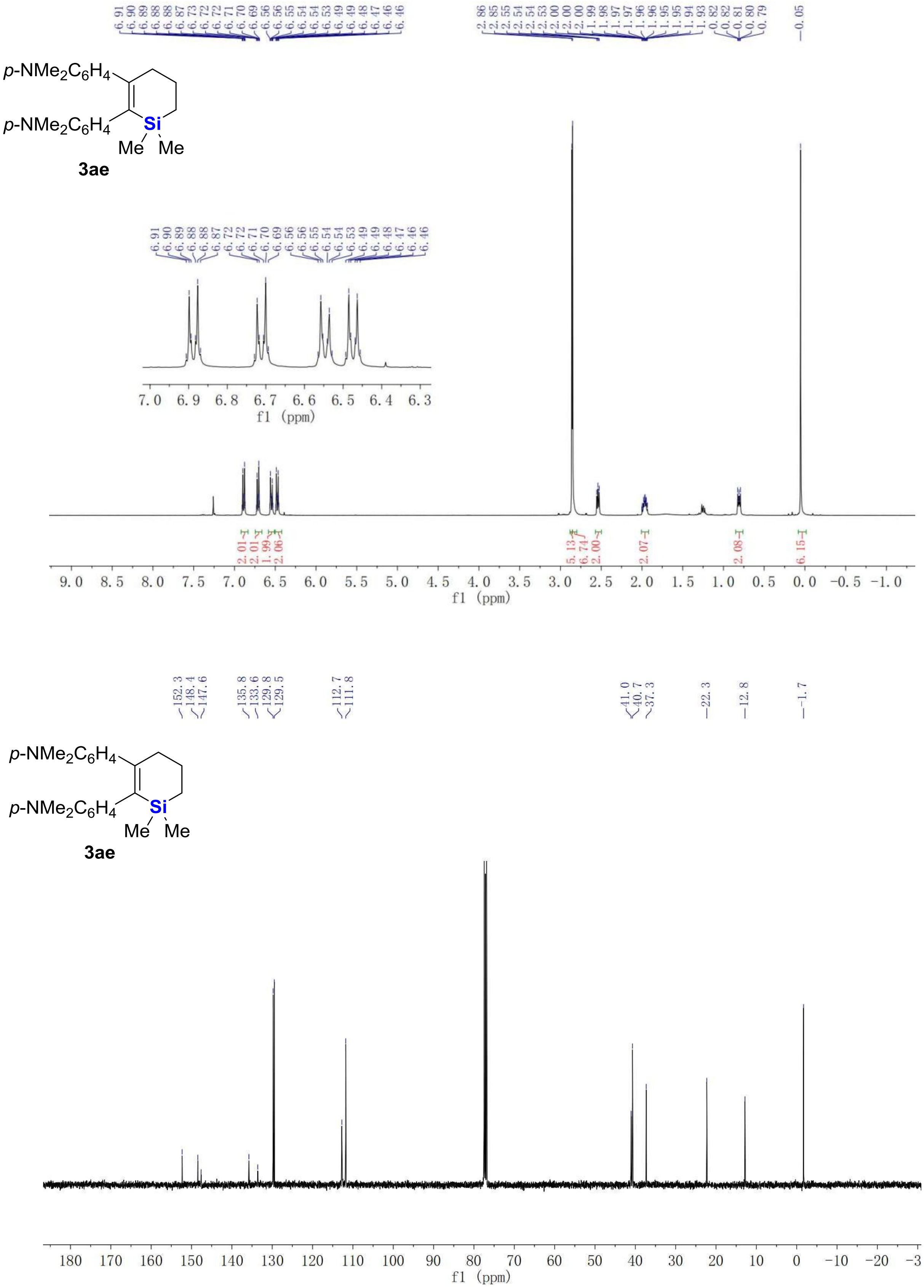

Supplementary Figure 15 ^{1}H and ^{13}C NMR Spectra for compound 3ae

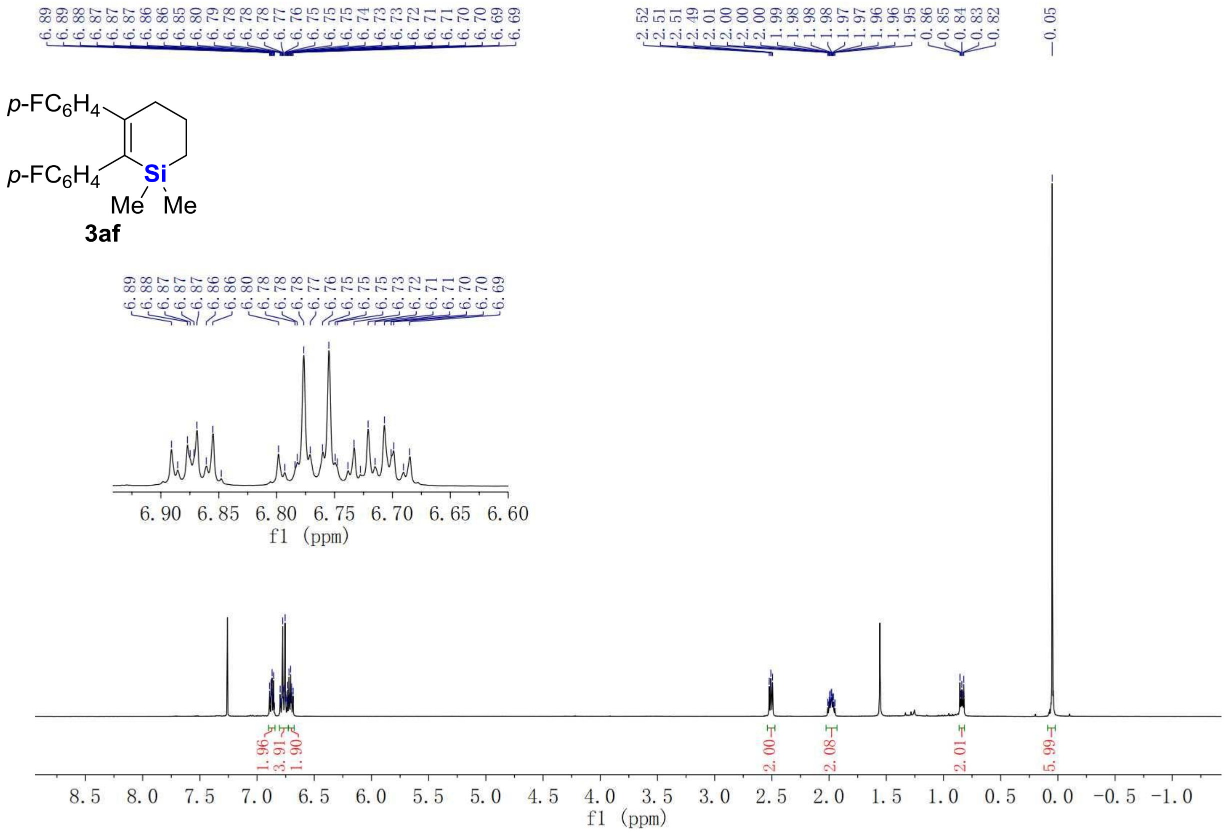

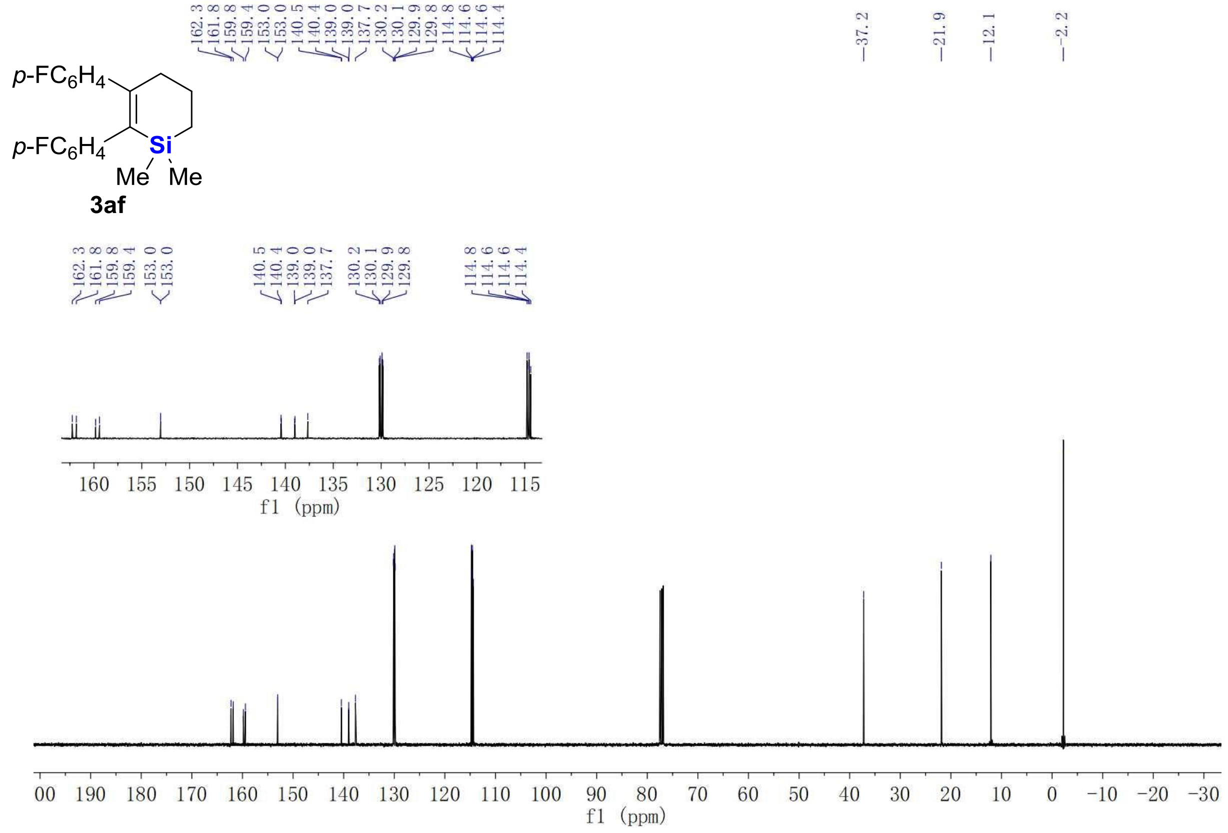

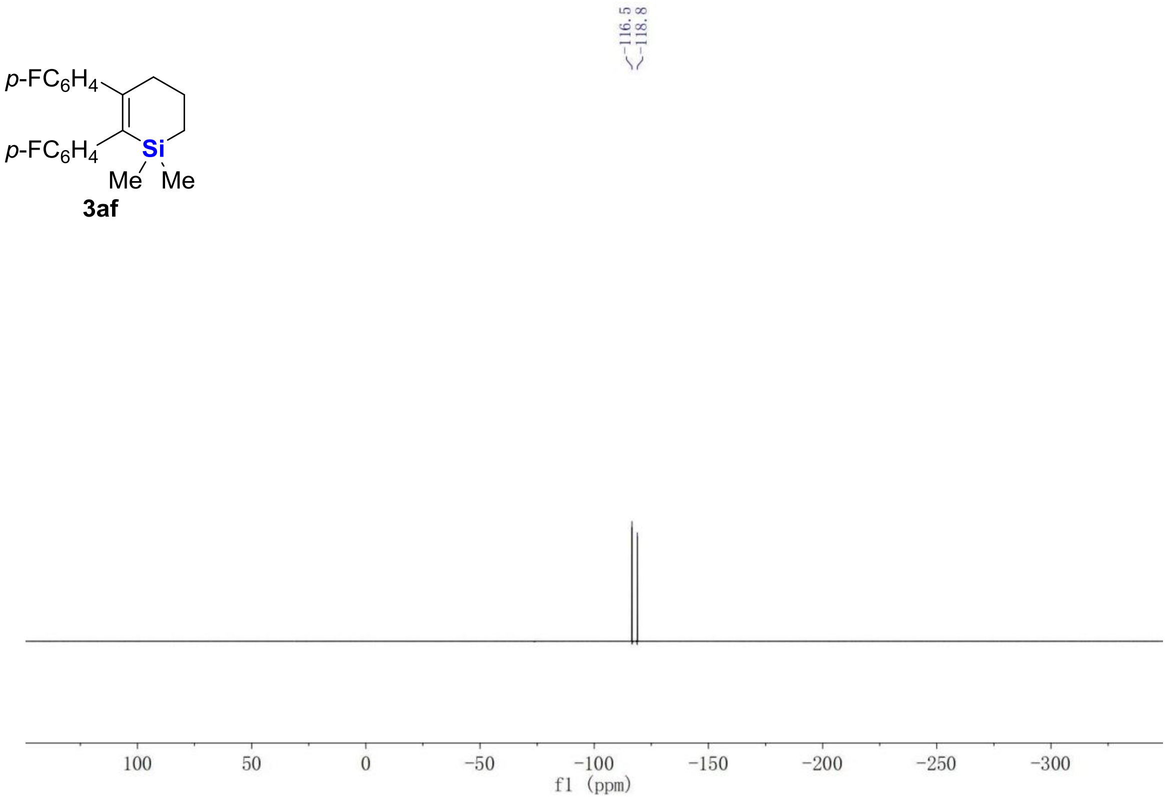

Supplementary Figure 16 ^{1}H, ^{13}C and ^{19}F NMR Spectra for compound 3af

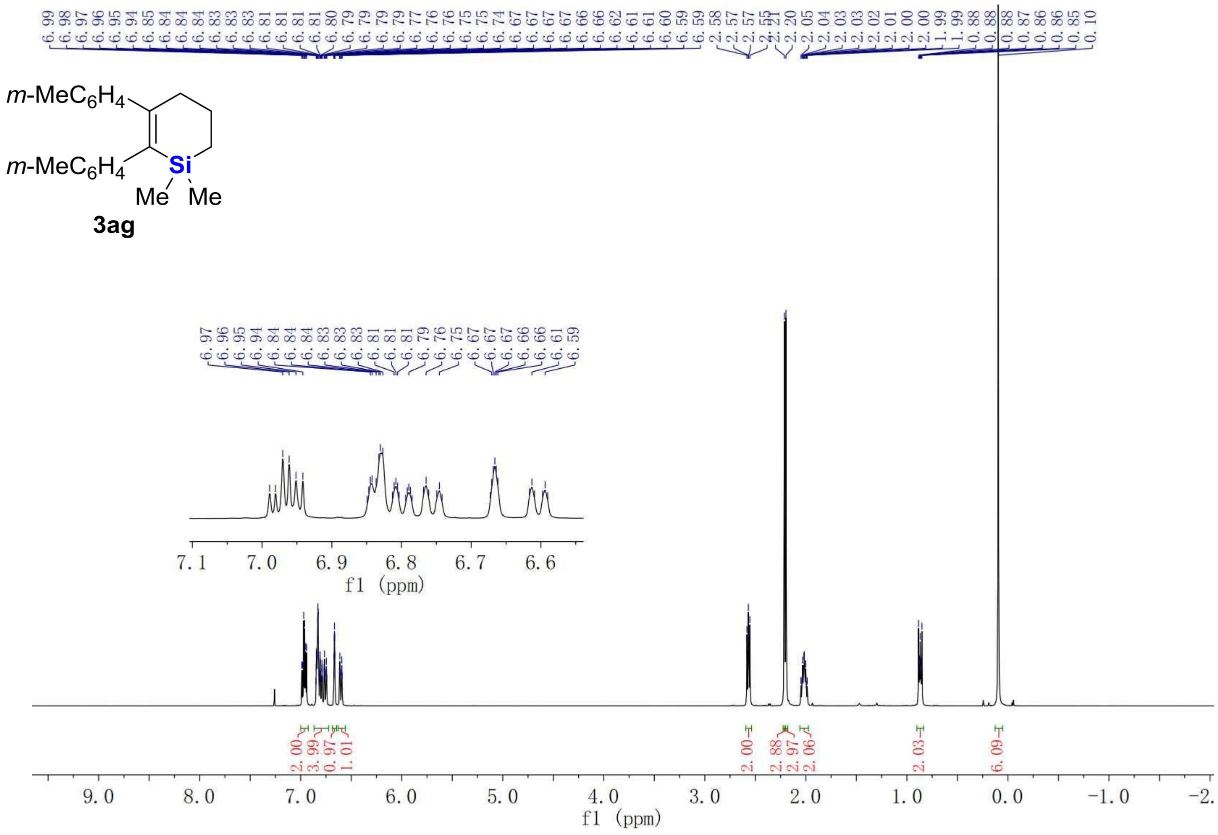

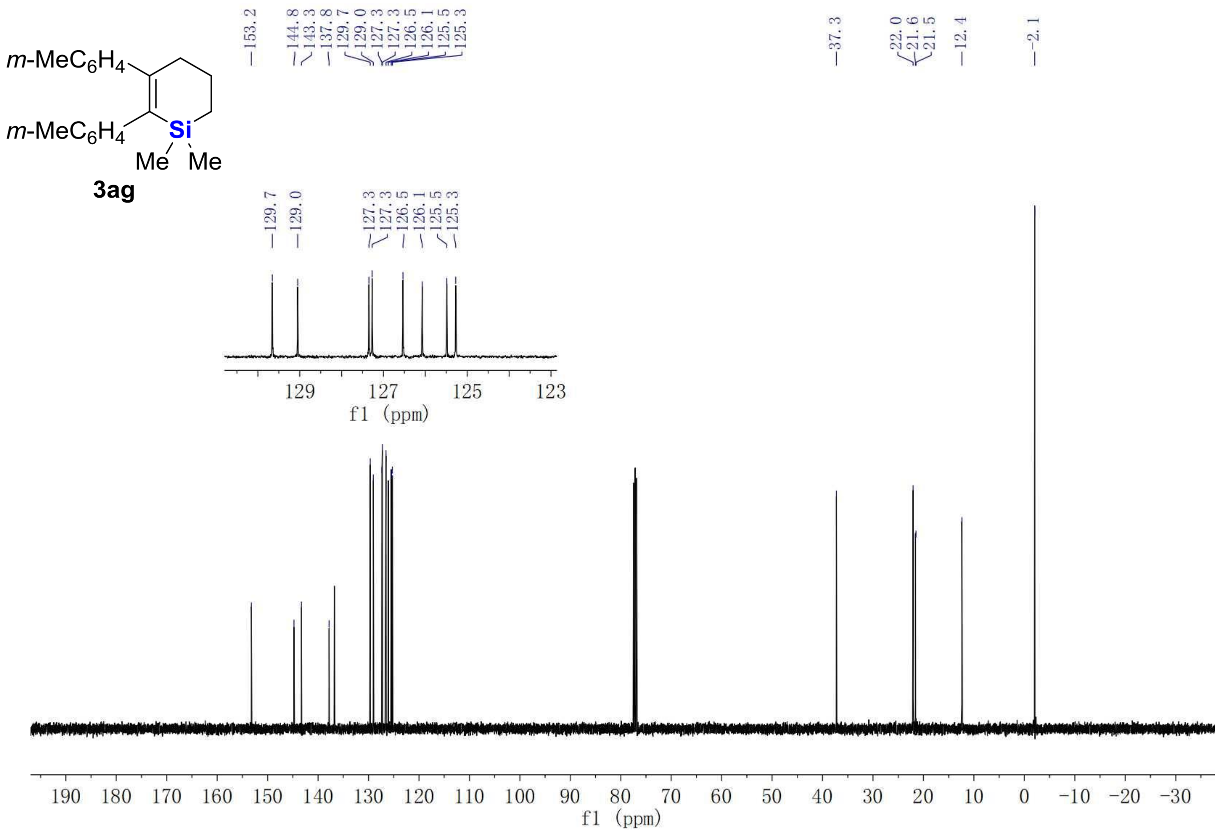

Supplementary Figure 17 ^{1}H and ^{13}C NMR Spectra for compound 3ag

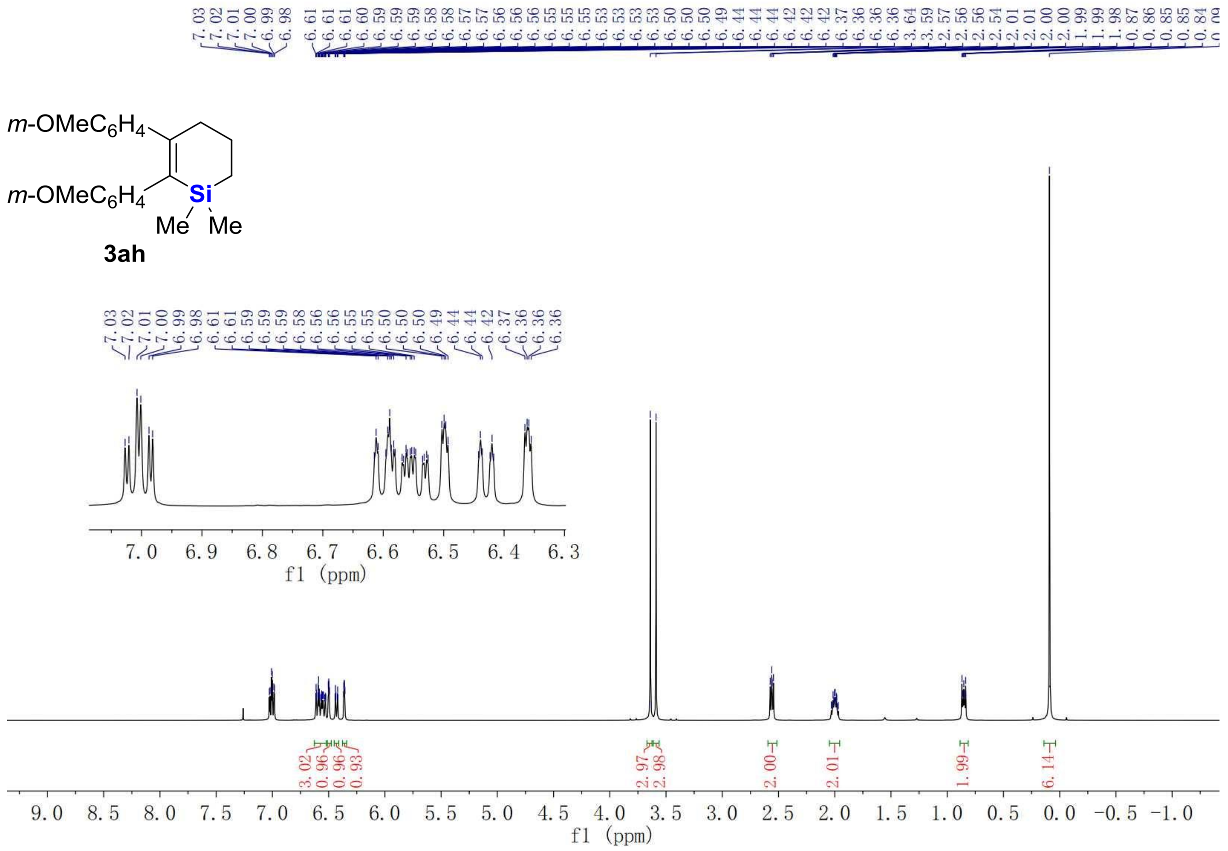

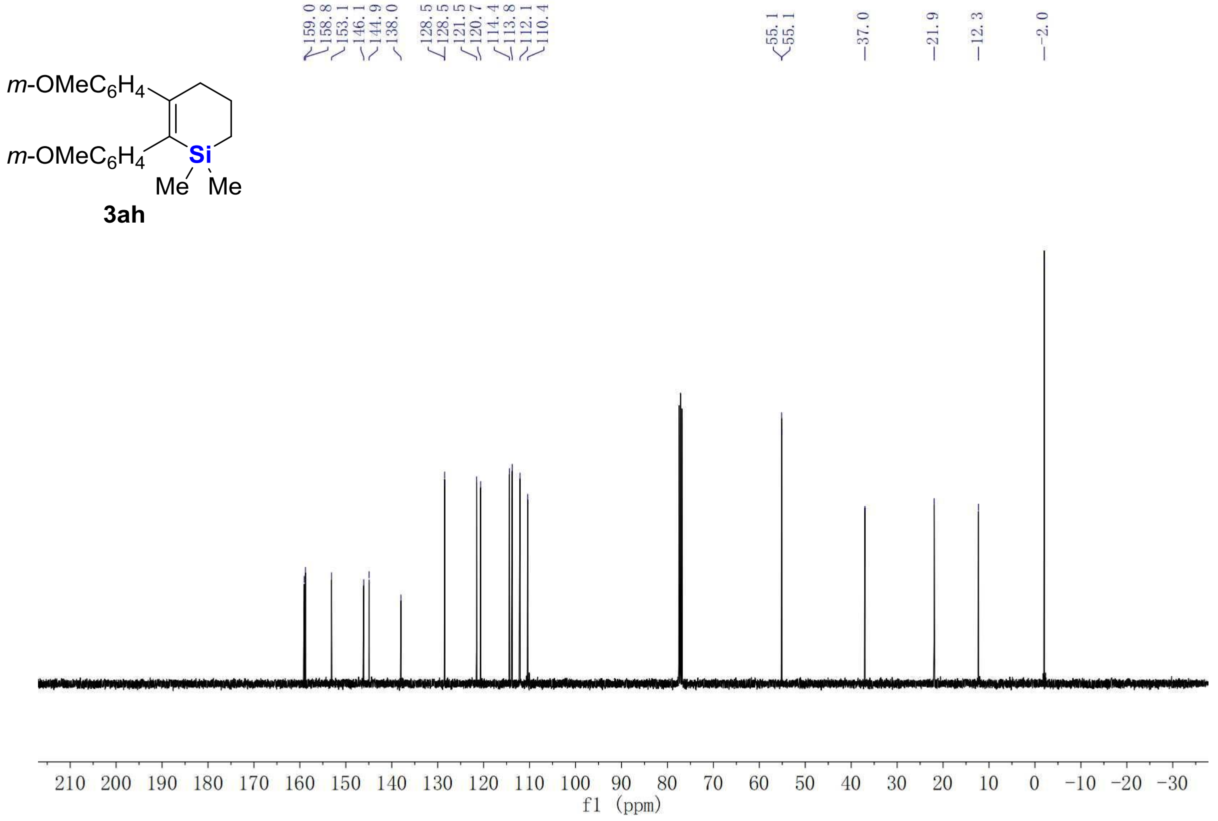

Supplementary Figure 18 ^{1}H and ^{13}C NMR Spectra for compound 3ah

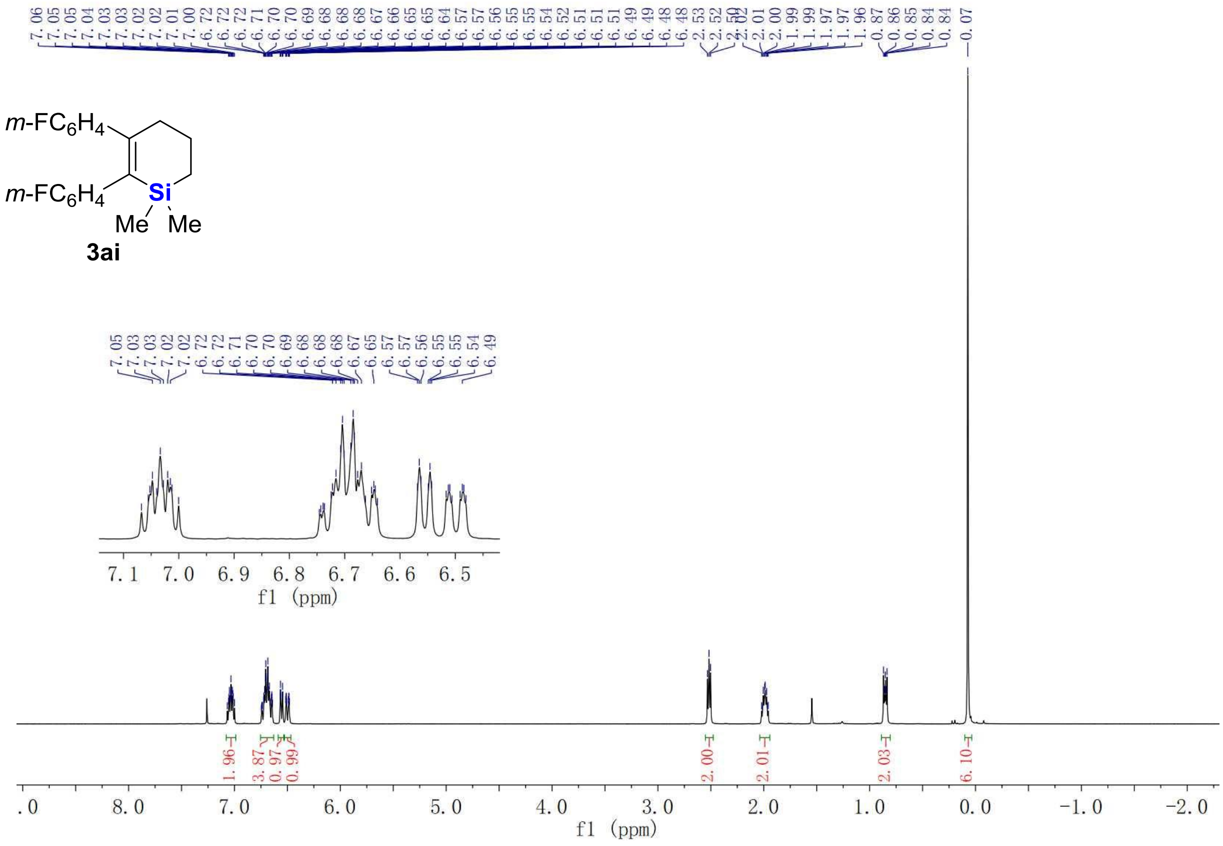
m-FC6H4
m-FC6H4
Si
Me
Me
3ai
7.1 7.0 6.9 6.8 6.7 6.6 6.5
f1 (ppm)
1.96
3.87
0.97
0.99
2.00
2.01
2.03
6.10
f1 (ppm)

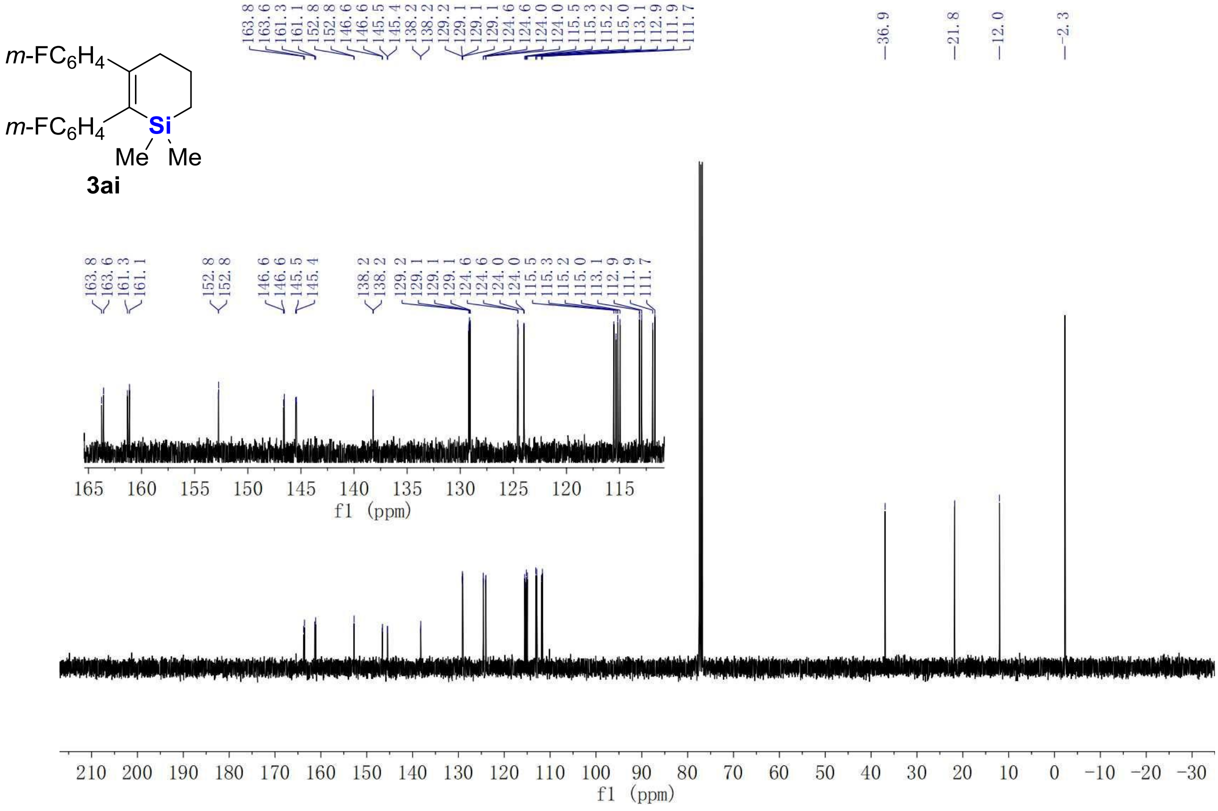
m-FC6H4
m-FC6H4
Si
Me
Me
3ai
36.9
21.8
12.0
-2.3
f1 (ppm)
f1 (ppm)

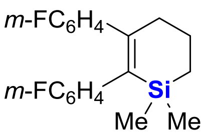

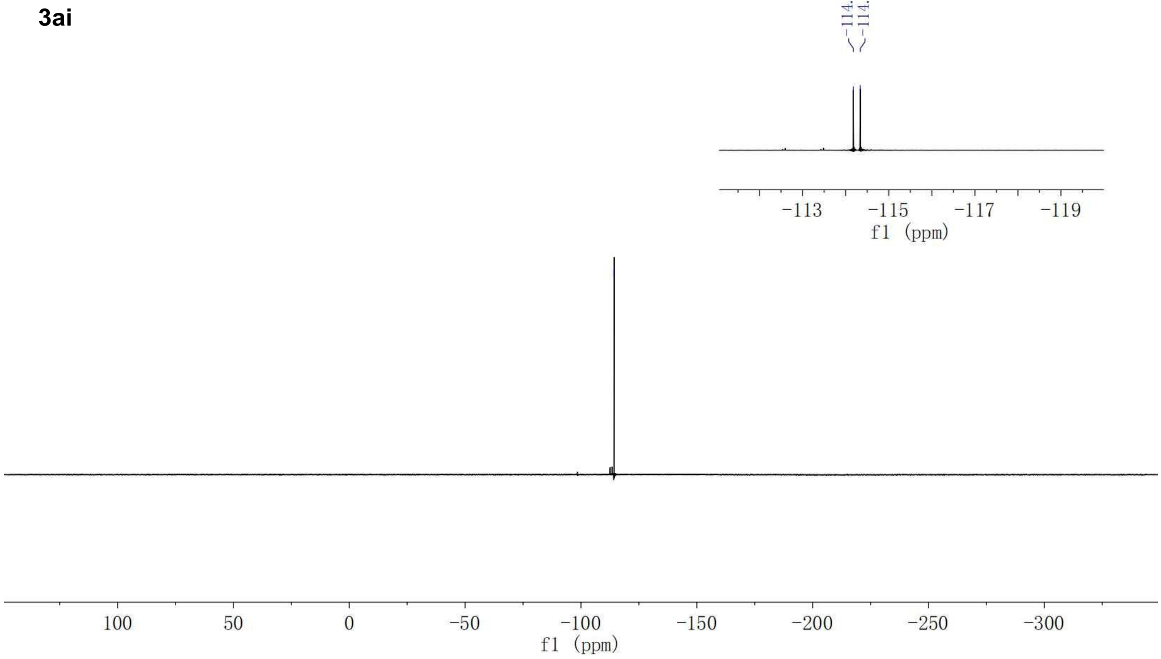

Supplementary Figure 19 ^{1}H, ^{13}C and ^{19}F NMR Spectra for compound 3ai

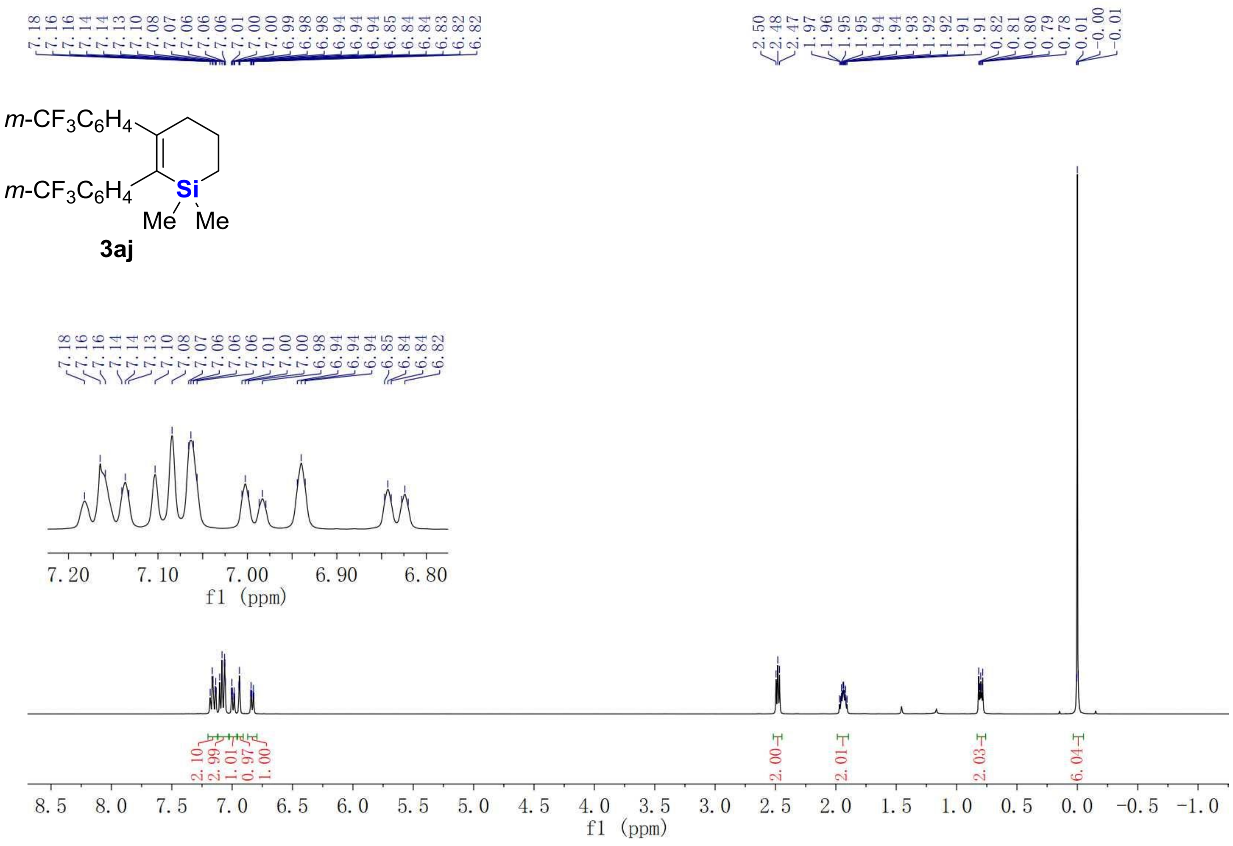
m-CF3C6H4
m-CF3C6H4
Si
Me Me
3aj
f1 (ppm)
f1 (ppm)

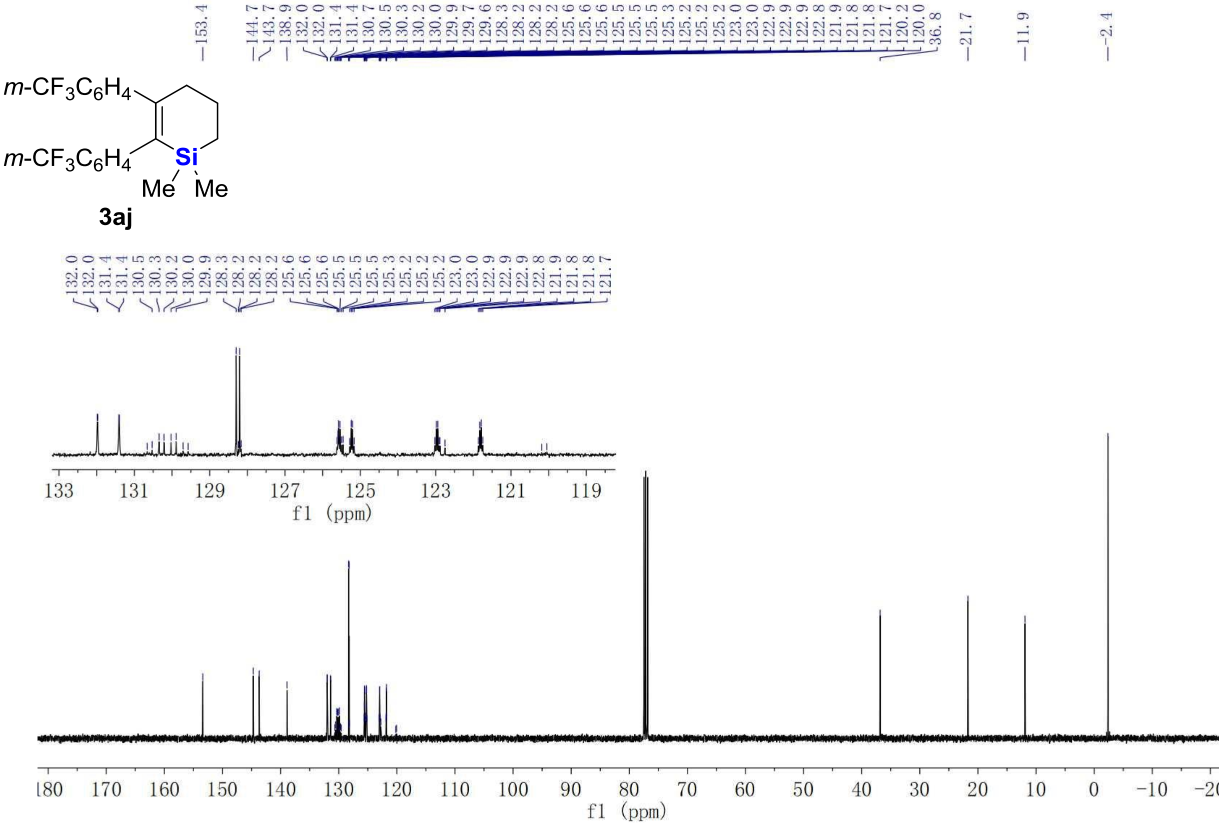
m-CF3C6H4
m-CF3C6H4
Si
Me Me
3aj
f1 (ppm)
f1 (ppm)

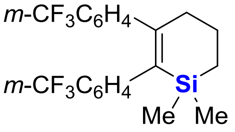

3aj

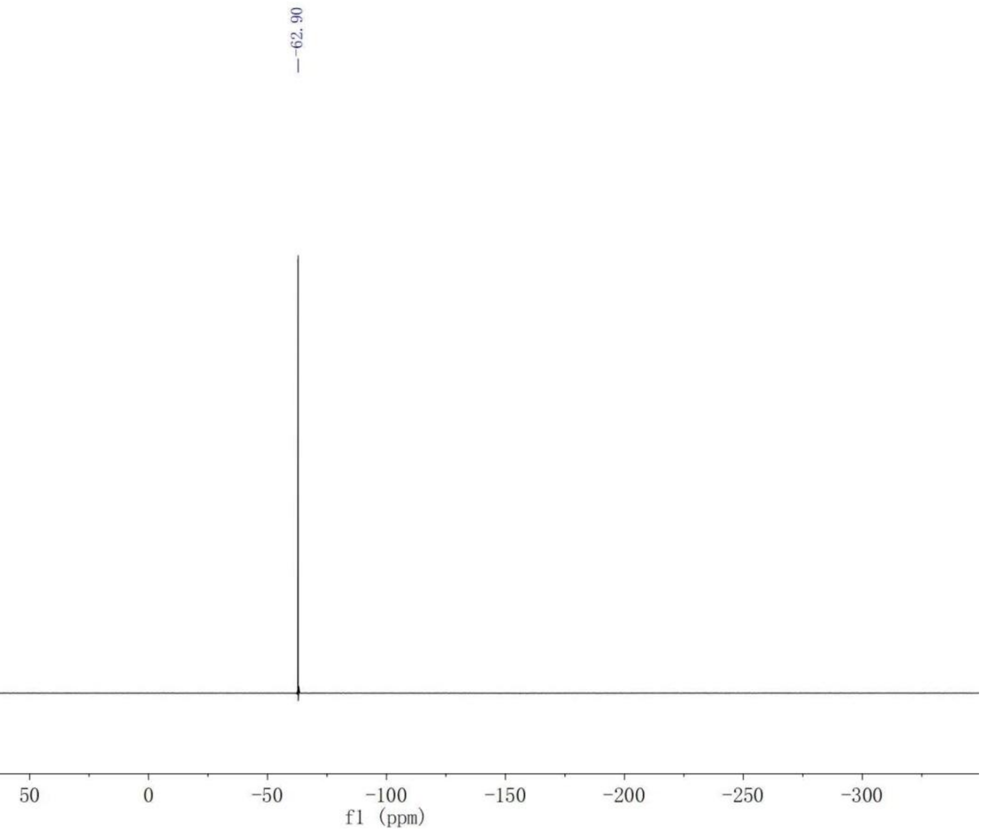

Supplementary Figure 20 ^{1}H, ^{13}C and ^{19}F NMR Spectra for compound 3aj

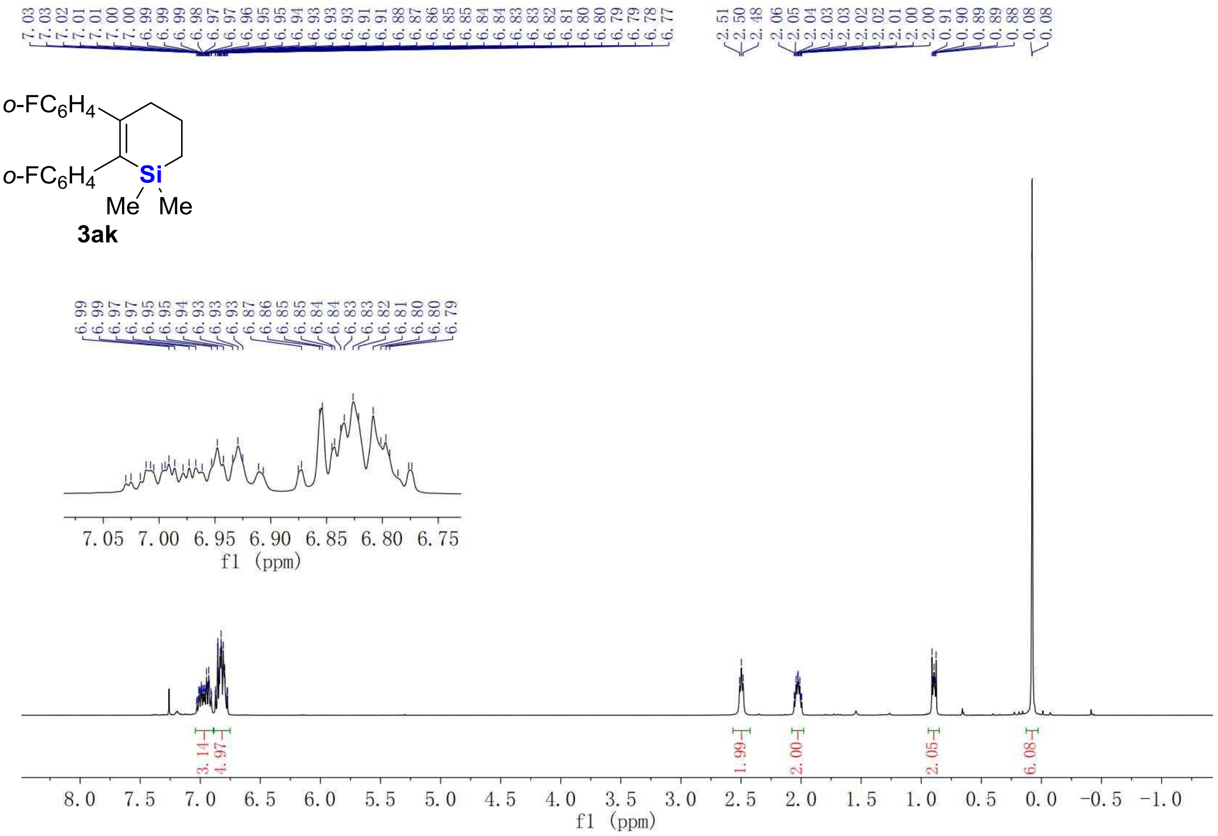
7.03 7.03 7.02 7.01 7.01 7.00 7.00 6.99 6.99 6.98 6.97 6.97 6.96 6.95 6.95 6.94 6.93 6.93 6.93 6.91 6.88 6.87 6.86 6.85 6.85 6.84 6.84 6.83 6.83 6.82 6.81 6.80 6.80 6.79 6.78 6.77
2.51 2.50 2.48 2.06 2.05 2.04 2.03 2.03 2.02 2.02 2.01 2.00 0.91 0.90 0.89 0.88 0.08 0.08
o-FC6H4
o-FC6H4
Si
Me Me
3ak
6.99 6.99 6.97 6.95 6.95 6.94 6.93 6.93 6.93 6.87 6.86 6.85 6.85 6.84 6.84 6.83 6.83 6.82 6.81 6.80 6.80 6.79
7.05 7.00 6.95 6.90 6.85 6.80 6.75
f1 (ppm)
3.14 4.97 1.99 2.00 2.05 6.08
8.0 7.5 7.0 6.5 6.0 5.5 5.0 4.5 4.0 3.5 3.0 2.5 2.0 1.5 1.0 0.5 0.0 -0.5 -1.0
f1 (ppm)

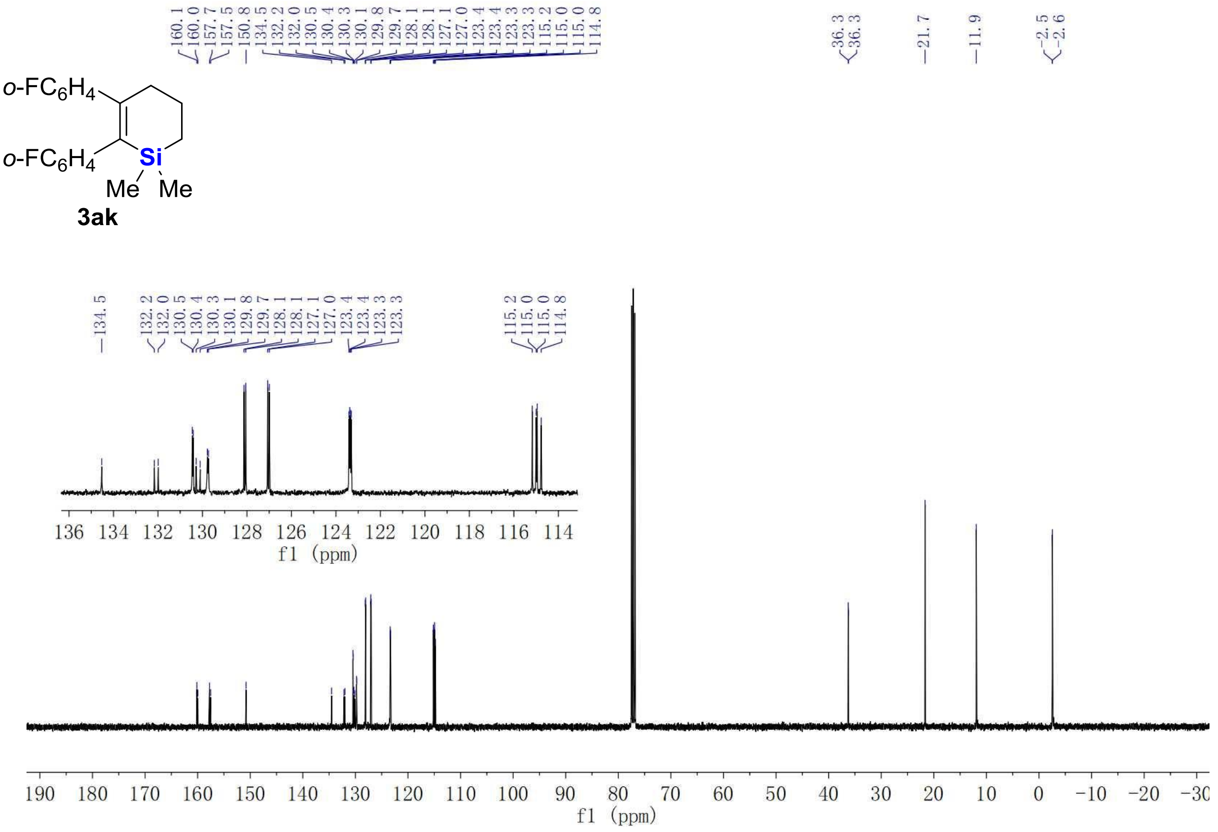
160.1 160.0 157.7 157.5 150.8 134.5 132.2 132.0 130.5 130.4 130.3 130.1 129.8 129.7 128.1 128.1 127.1 127.0 123.4 123.4 123.3 123.3 115.2 115.0 115.0 114.8
36.3 36.3 21.7 11.9 -2.5 -2.6
o-FC6H4
o-FC6H4
Si
Me Me
3ak
134.5 132.2 132.0 130.5 130.4 130.3 130.1 129.8 129.7 128.1 128.1 127.1 127.0 123.4 123.4 123.3 123.3 115.2 115.0 115.0 114.8
136 134 132 130 128 126 124 122 120 118 116 114
f1 (ppm)
190 180 170 160 150 140 130 120 110 100 90 80 70 60 50 40 30 20 10 0 -10 -20 -30
f1 (ppm)

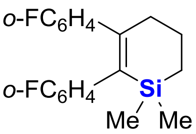

3ak

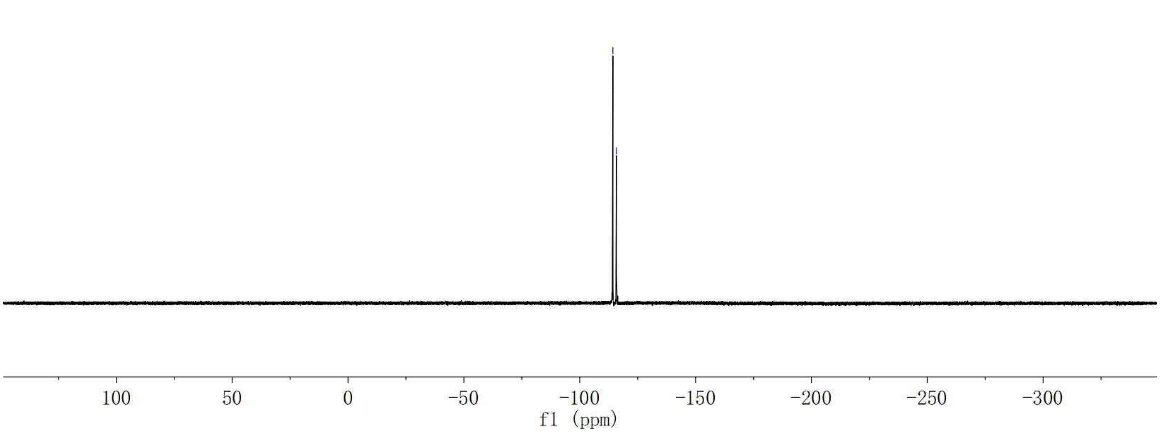

Supplementary Figure 21 ^{1}H, ^{13}C and ^{19}F NMR Spectra for compound 3ak

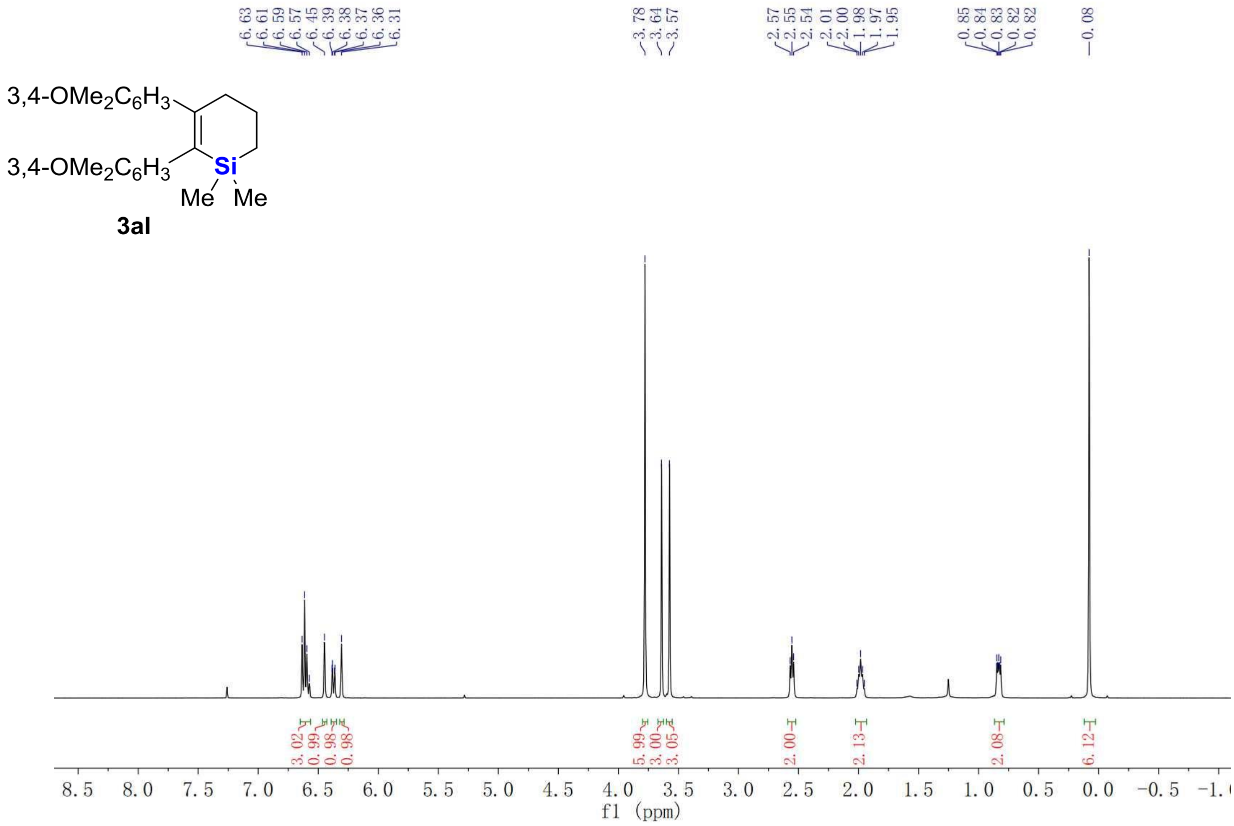

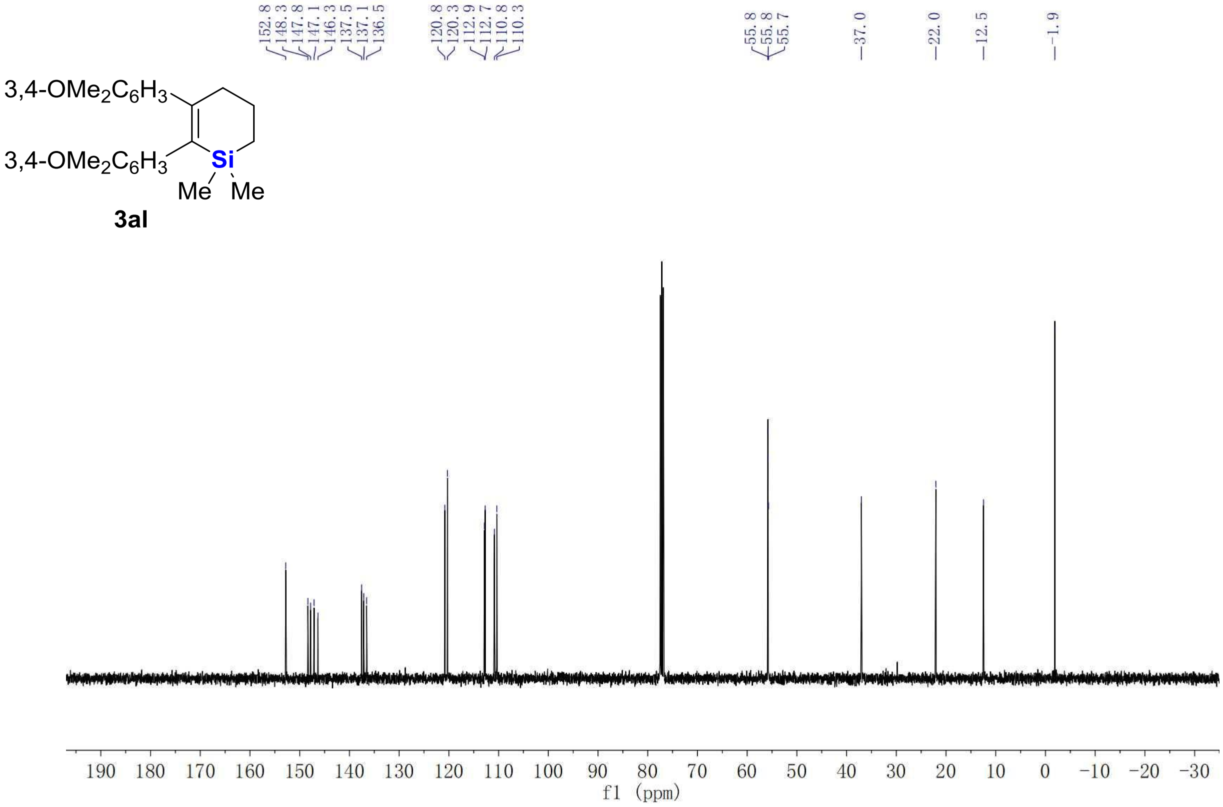

Supplementary Figure 22 ^{1}H and ^{13}C NMR Spectra for compound 3al

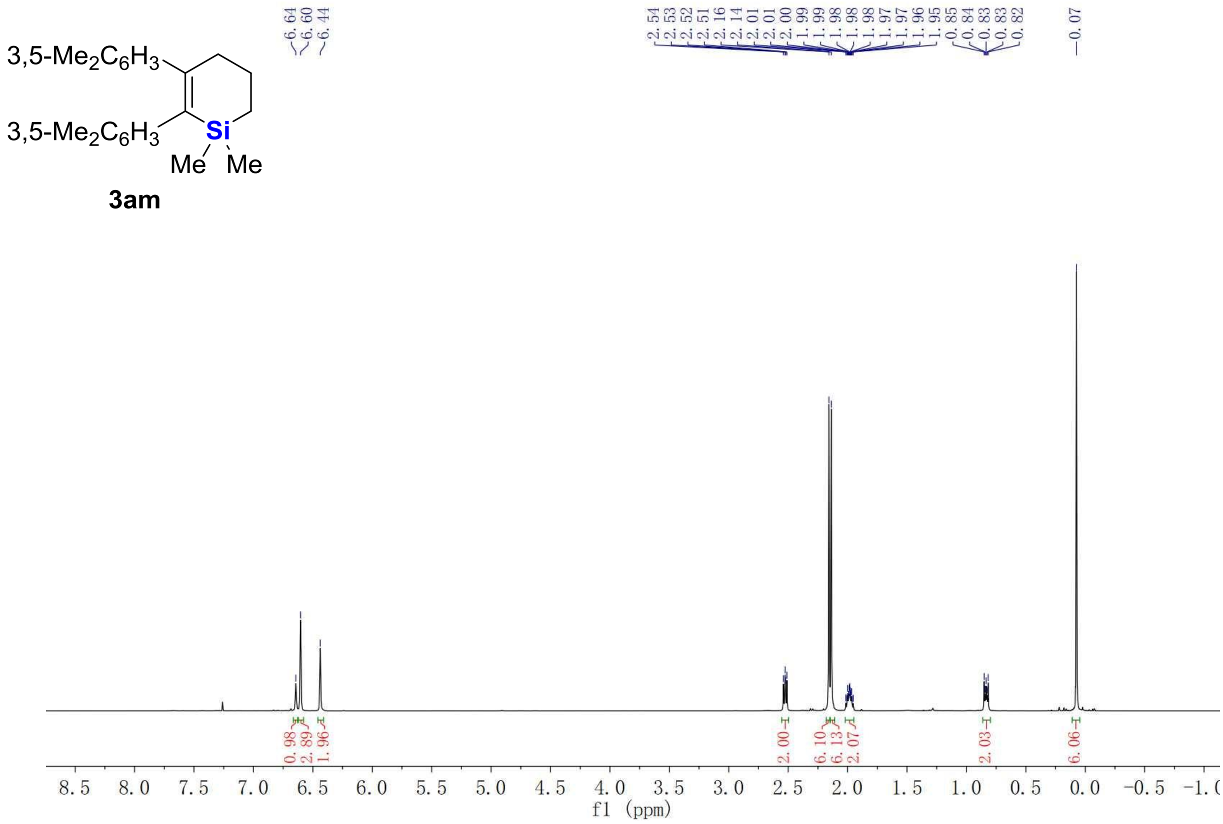

3am

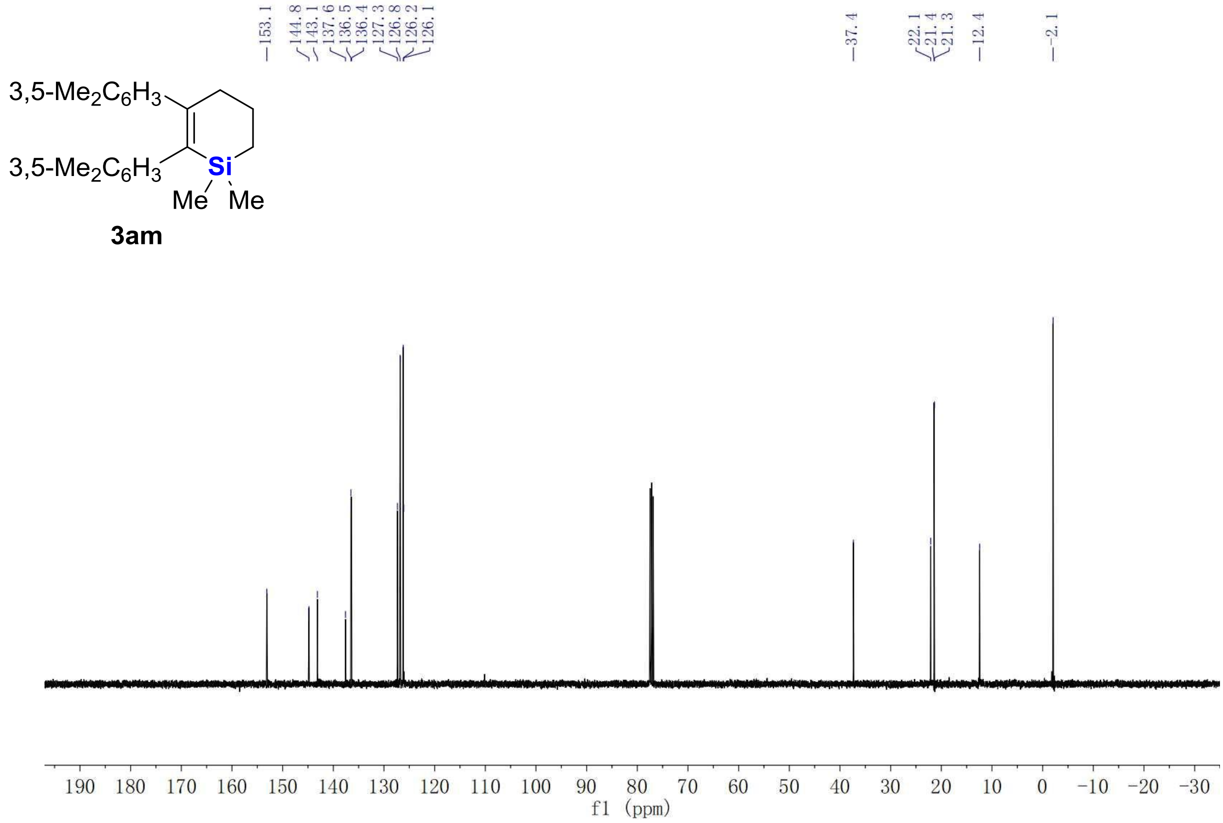

3am

Supplementary Figure 23 ^{1}H and ^{13}C NMR Spectra for compound 3am

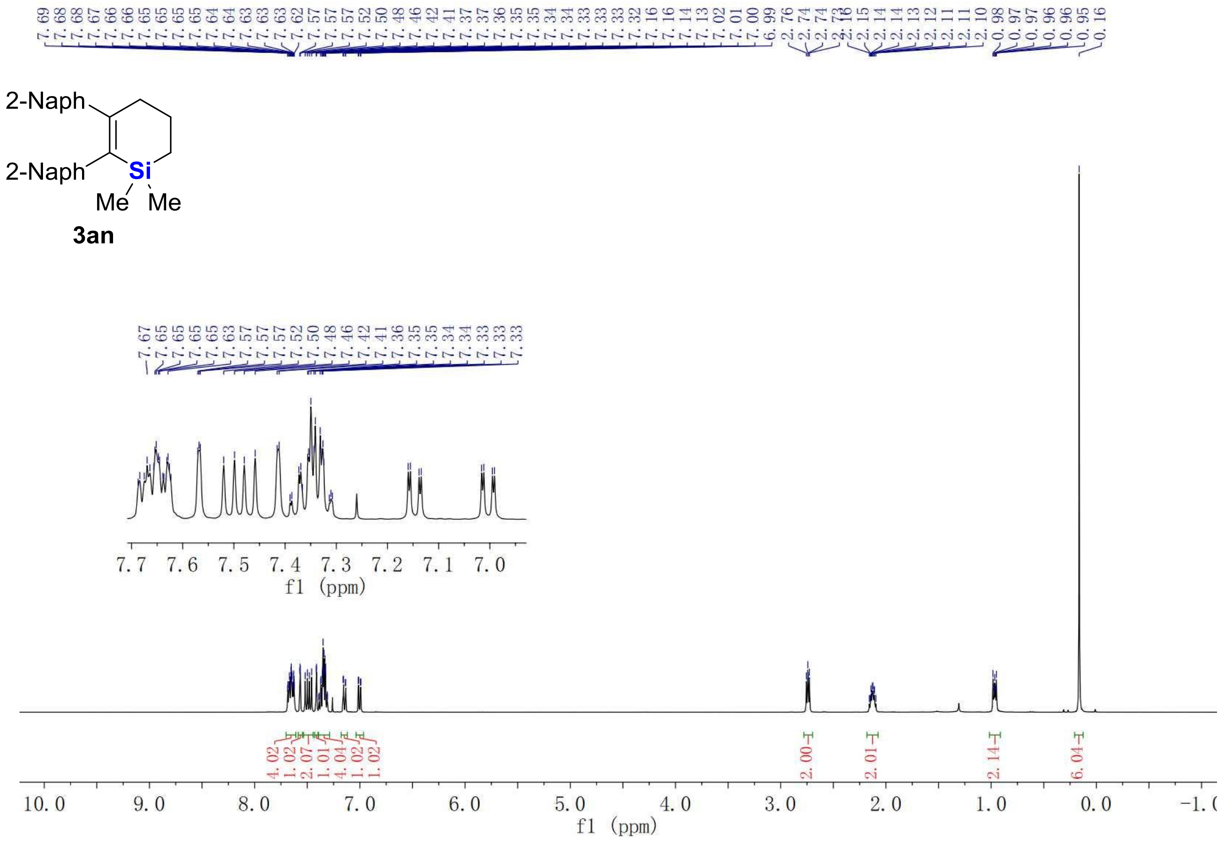

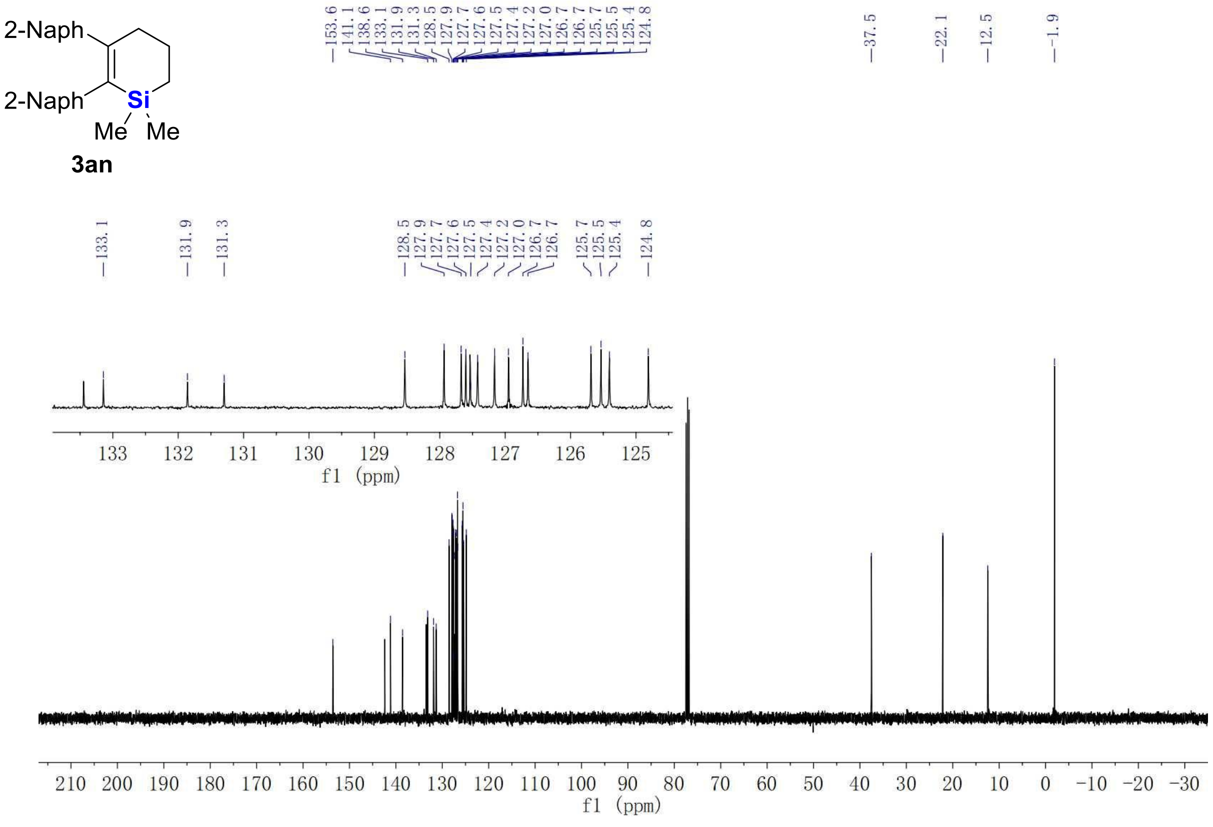

Supplementary Figure 24 ^{1}H and ^{13}C NMR Spectra for compound 3an

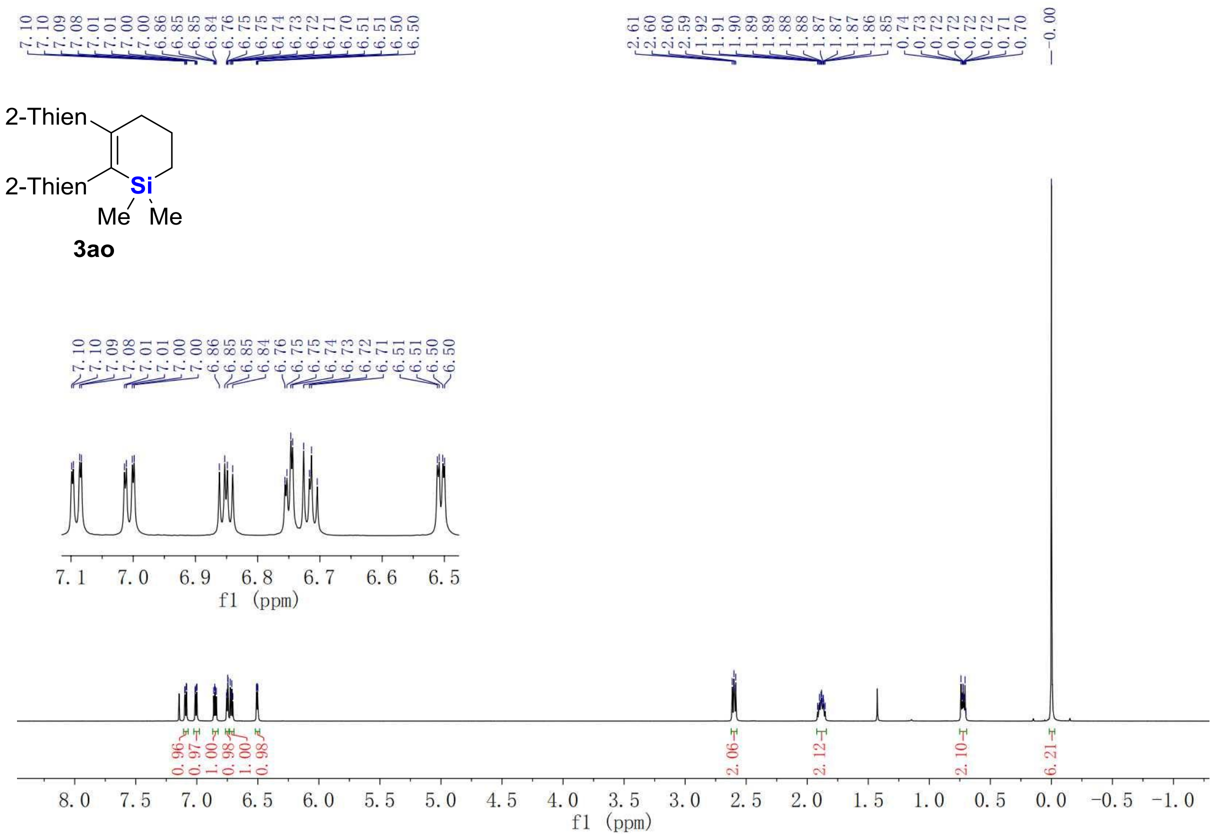

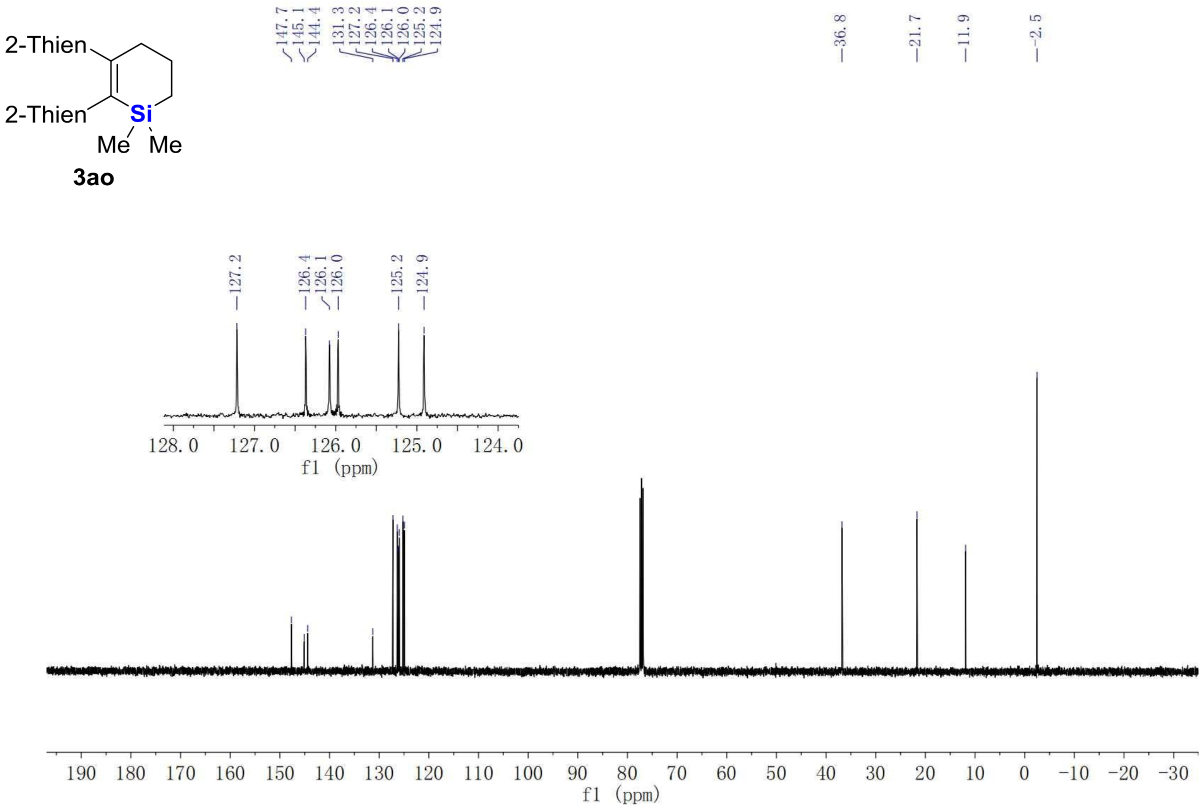

Supplementary Figure 25 ^{1}H and ^{13}C NMR Spectra for compound 3ao

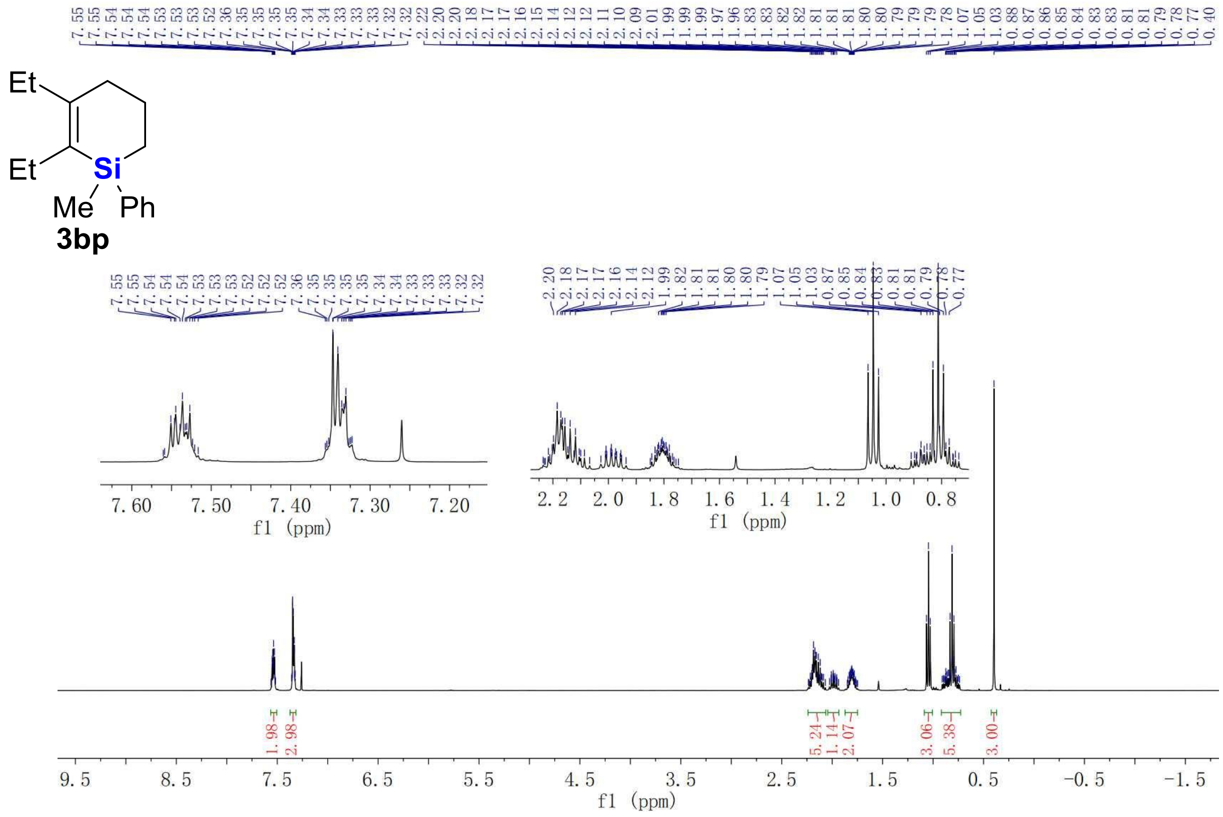

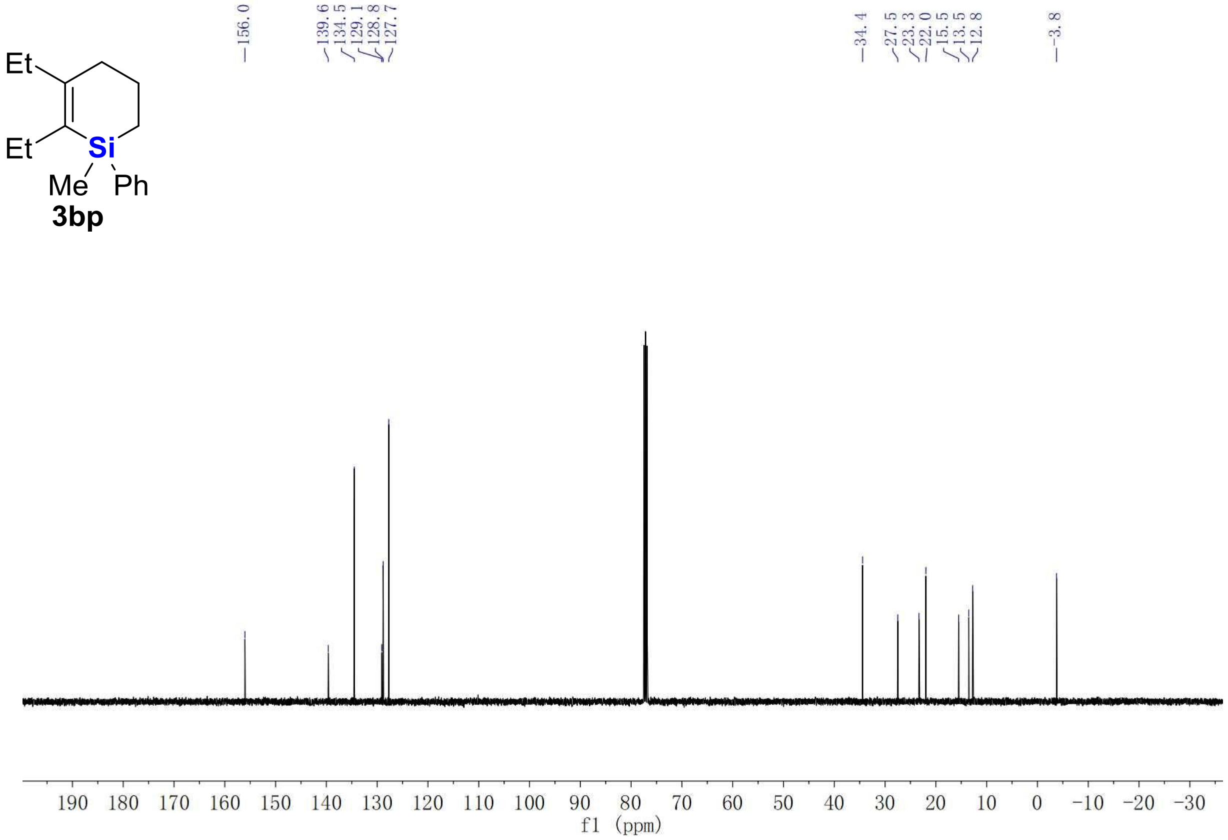

Supplementary Figure 26 ^{1}H and ^{13}C NMR Spectra for compound 3bp

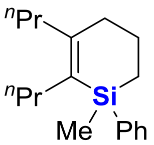

3bq

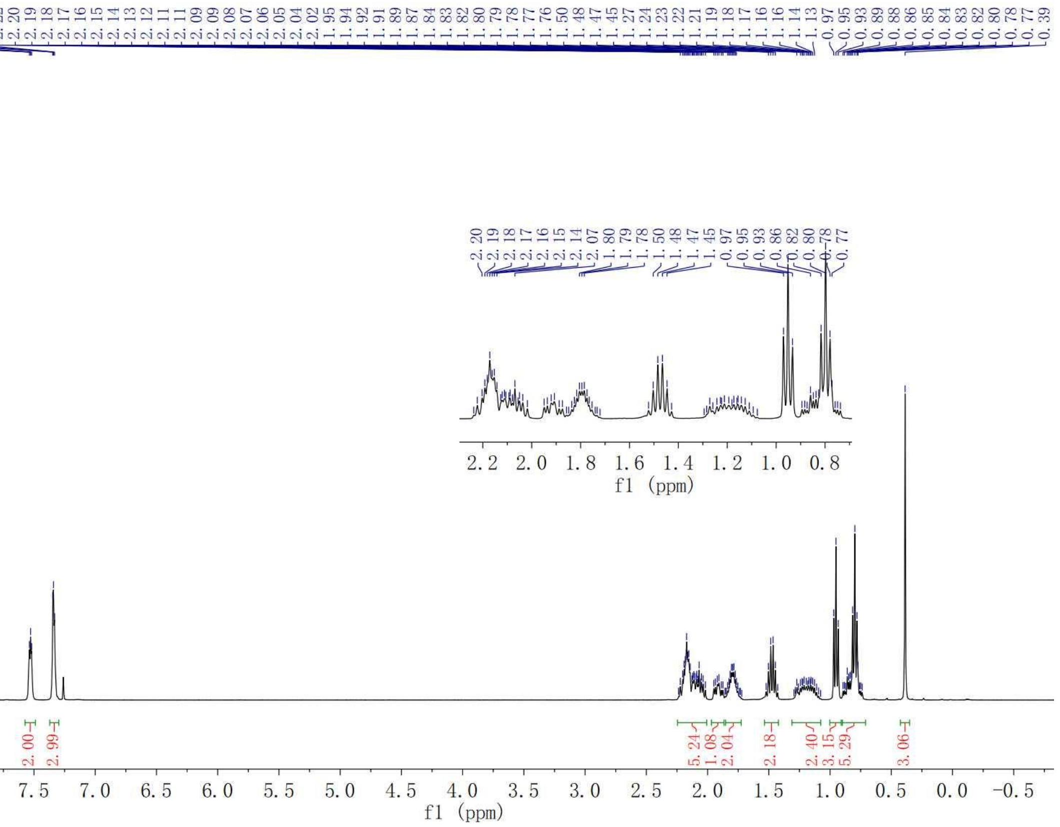

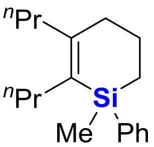

3bq

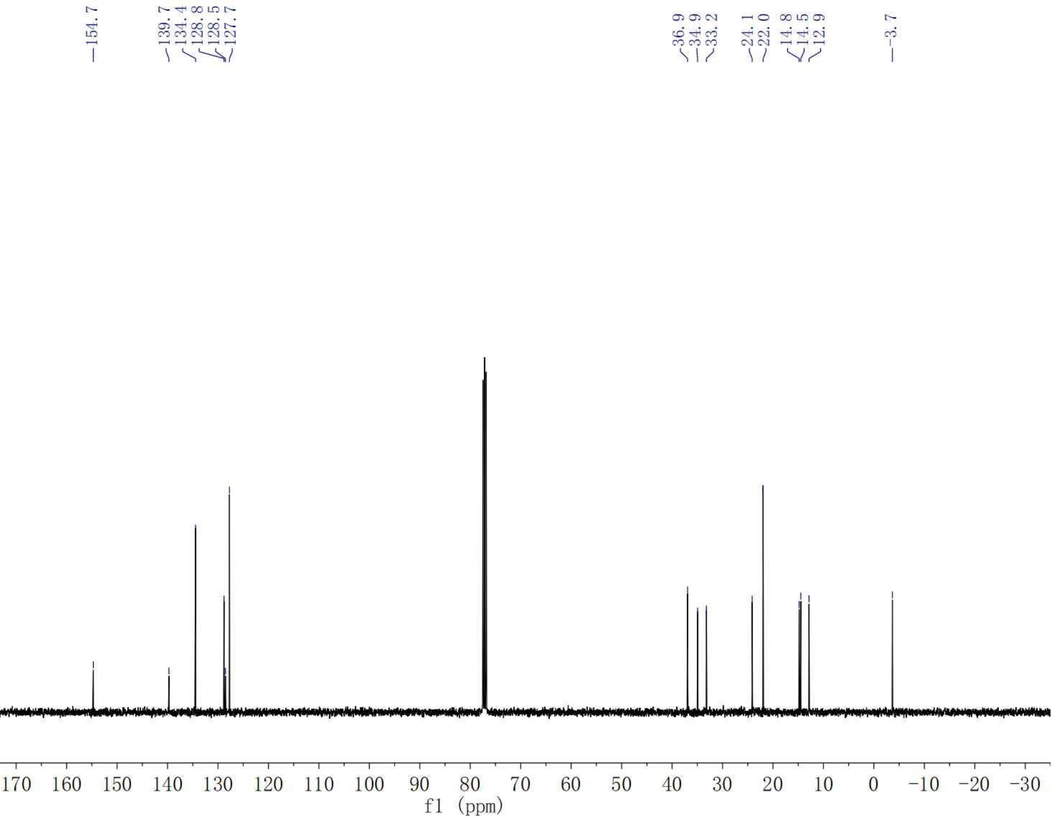

Supplementary Figure 27 ^{1}H and ^{13}C NMR Spectra for compound 3bq

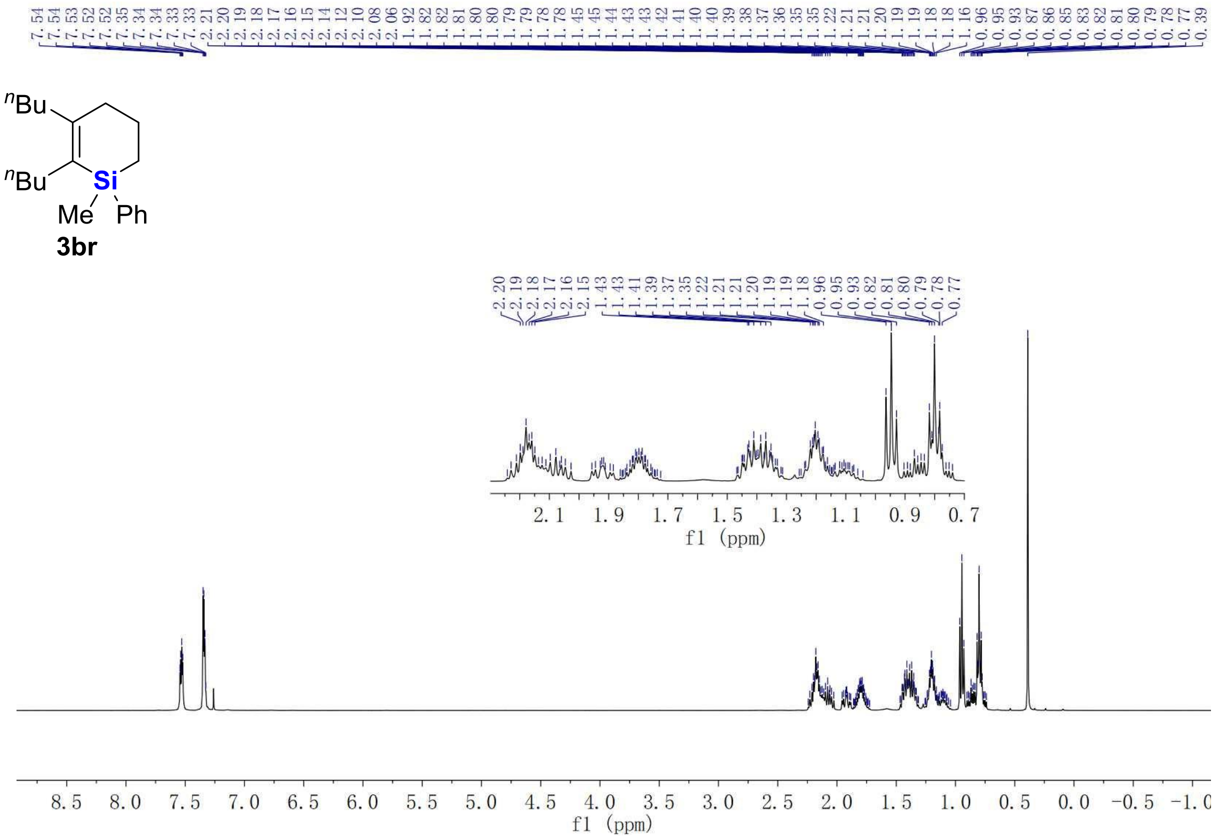

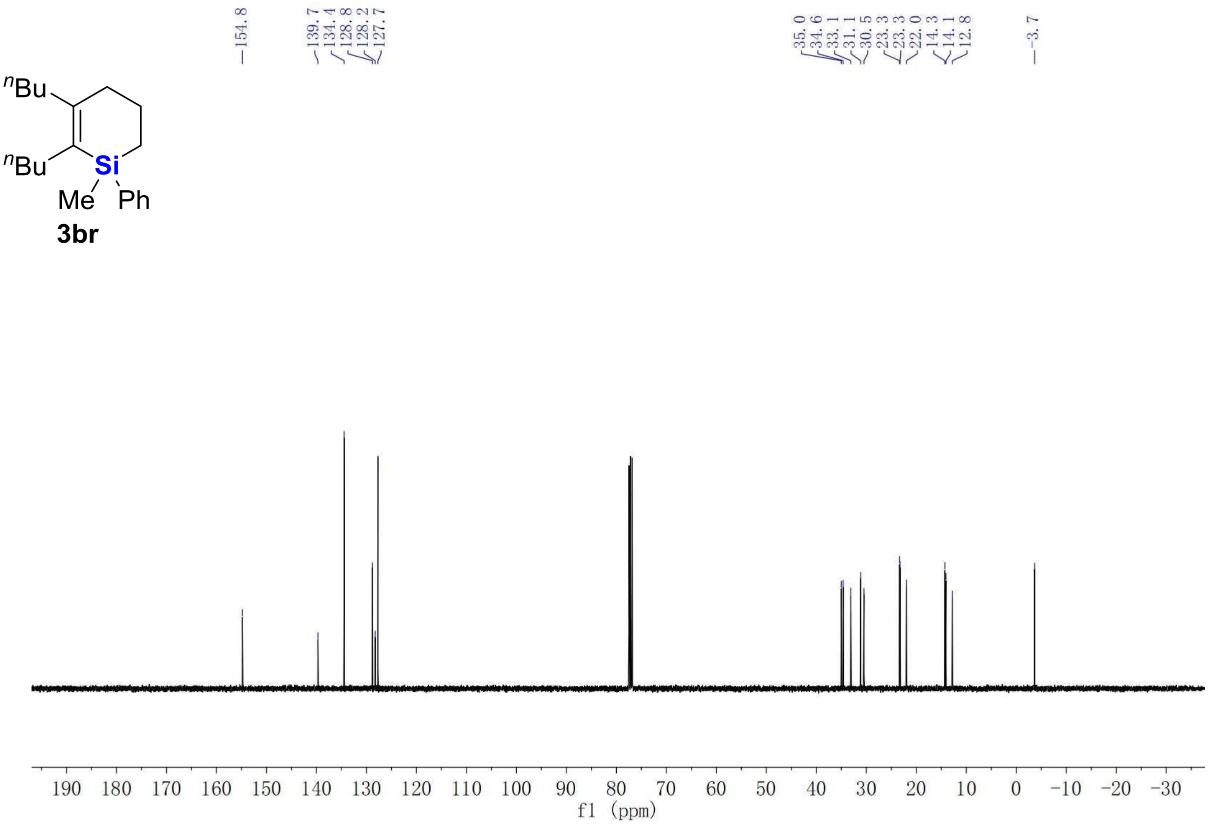

Supplementary Figure 28 ^{1}H and ^{13}C NMR Spectra for compound 3br

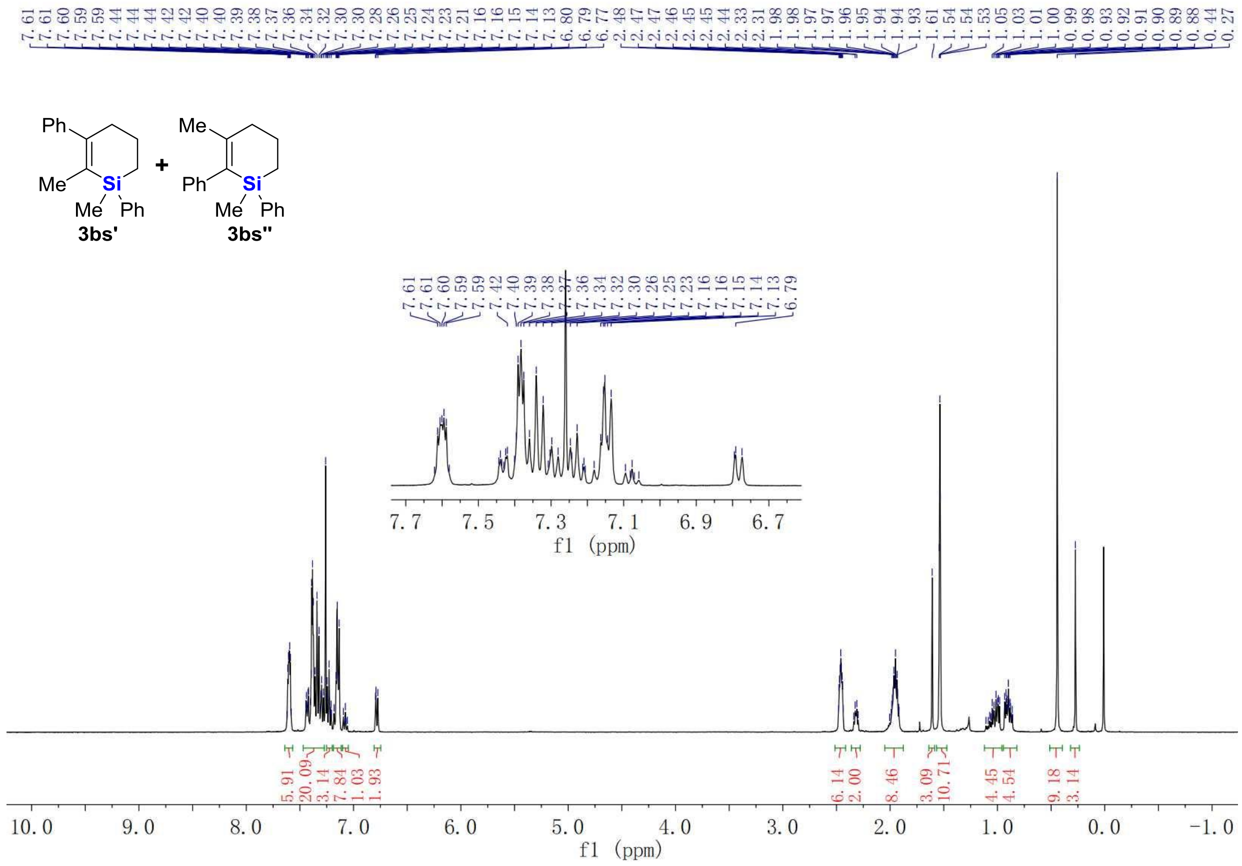

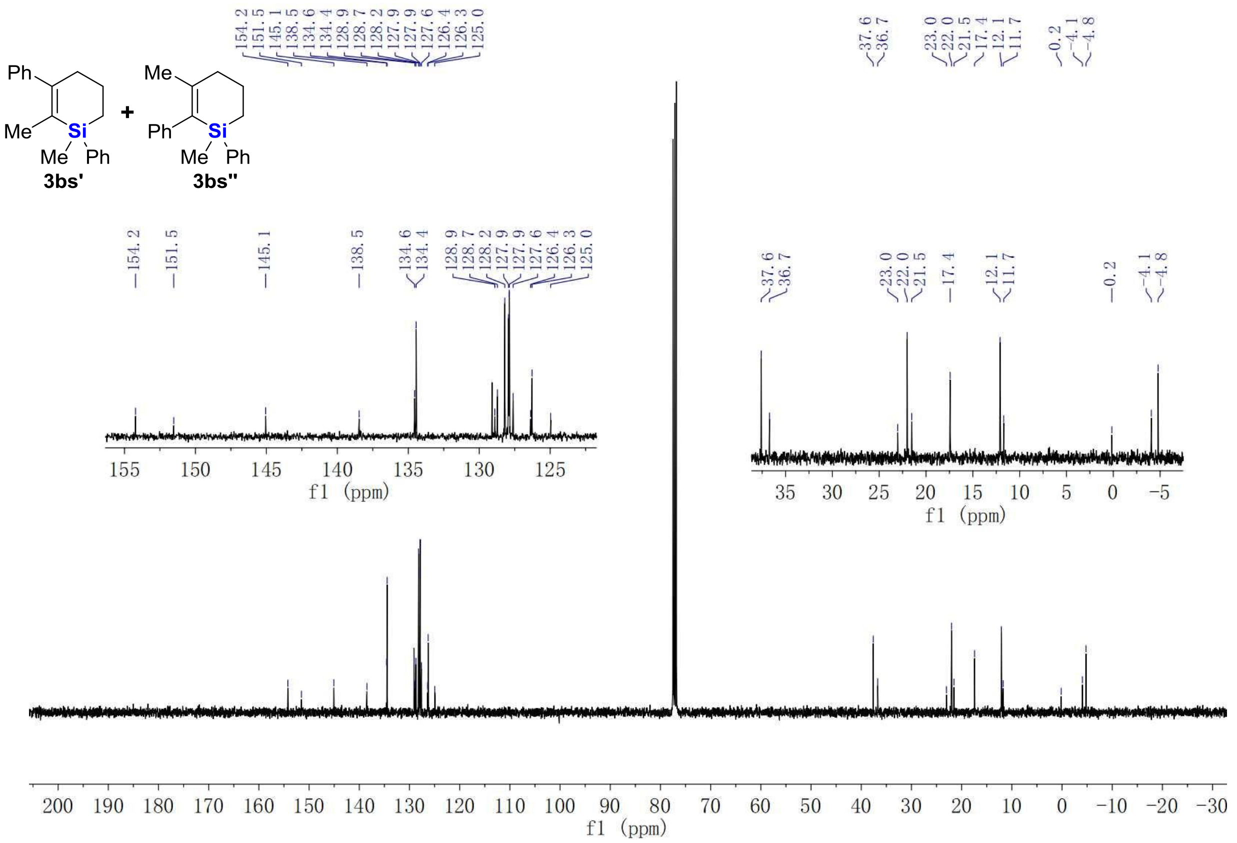

Supplementary Figure 29 ^{1}H and ^{13}C NMR Spectra for compound 3bs

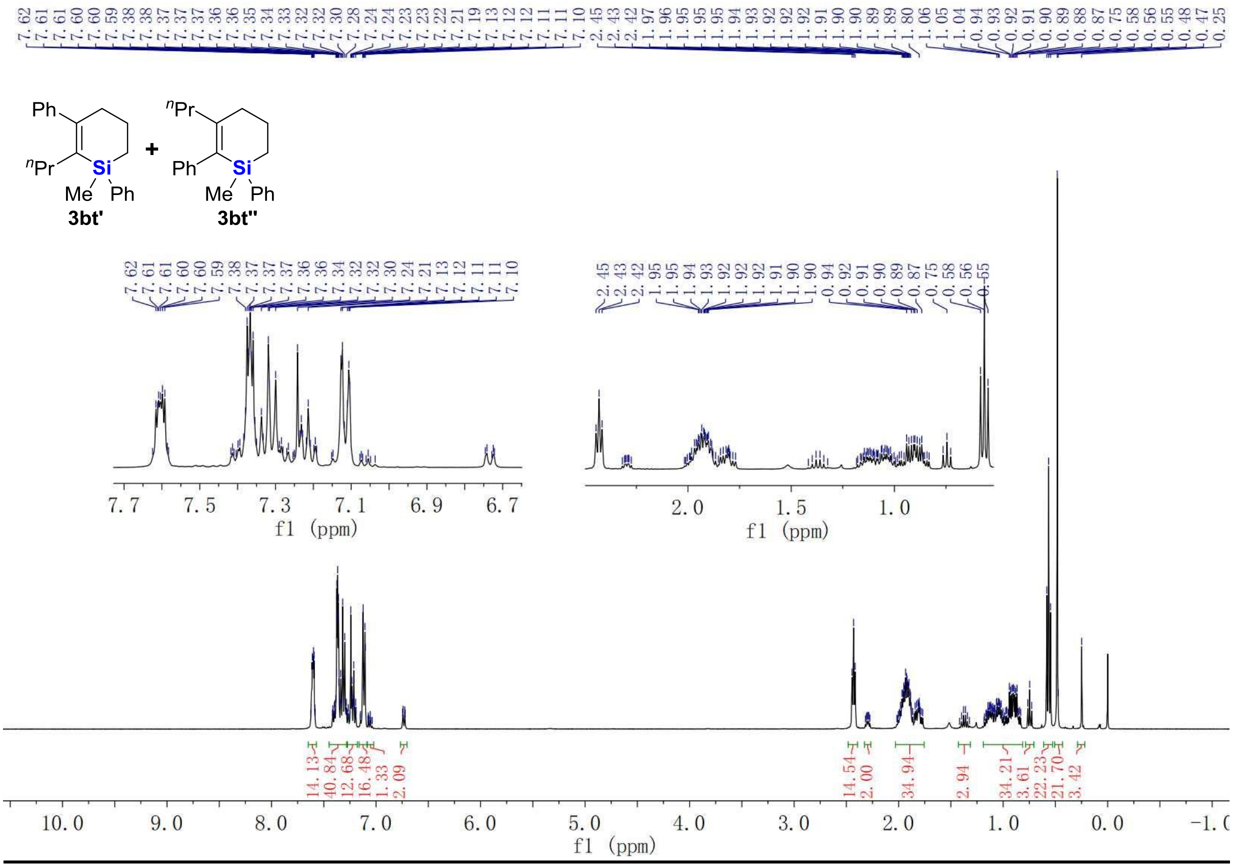

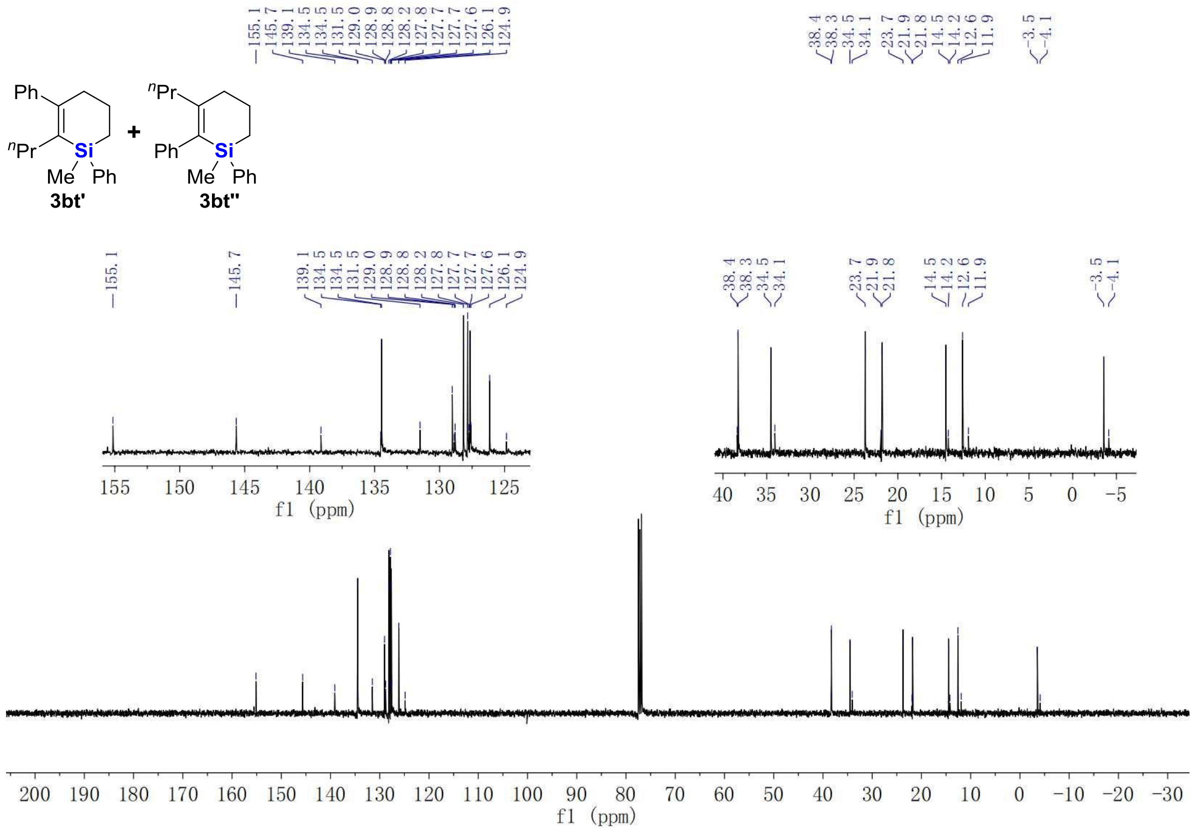

Supplementary Figure 30 ^{1}H and ^{13}C NMR Spectra for compound 3bt

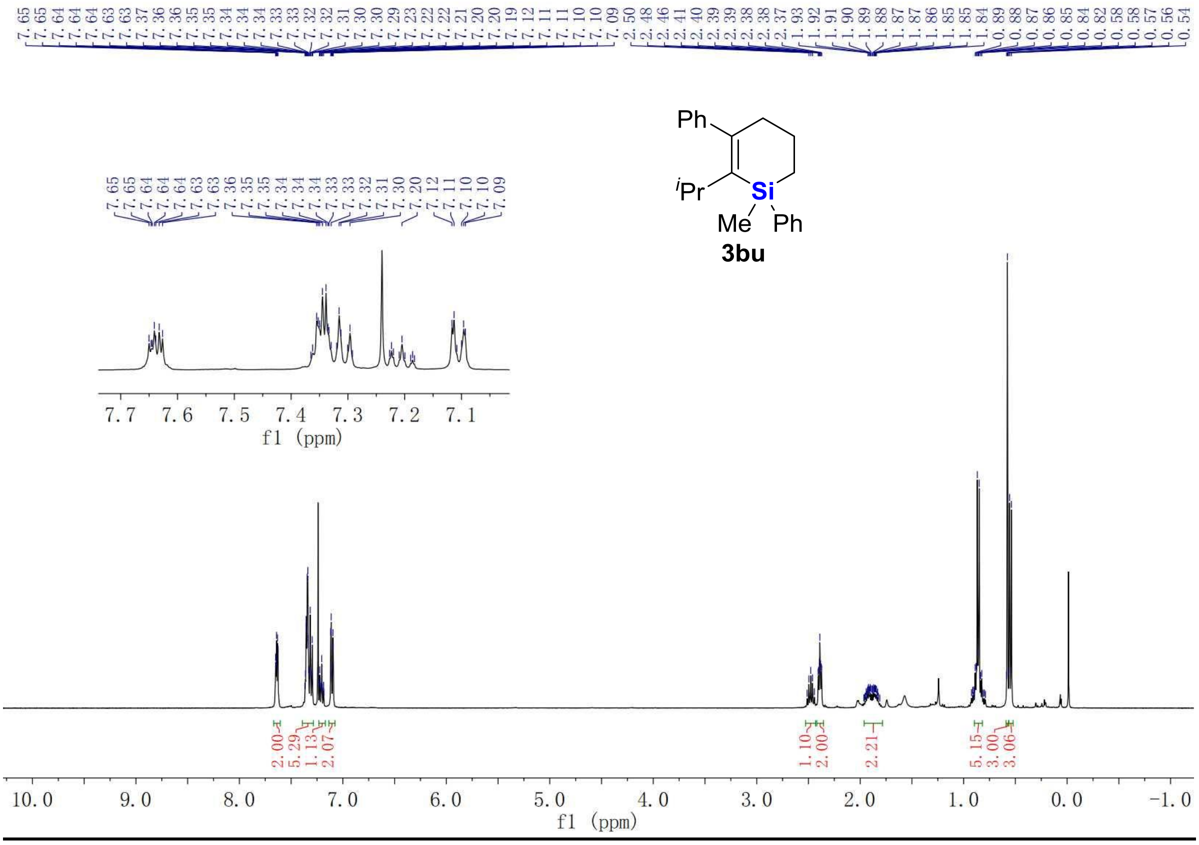

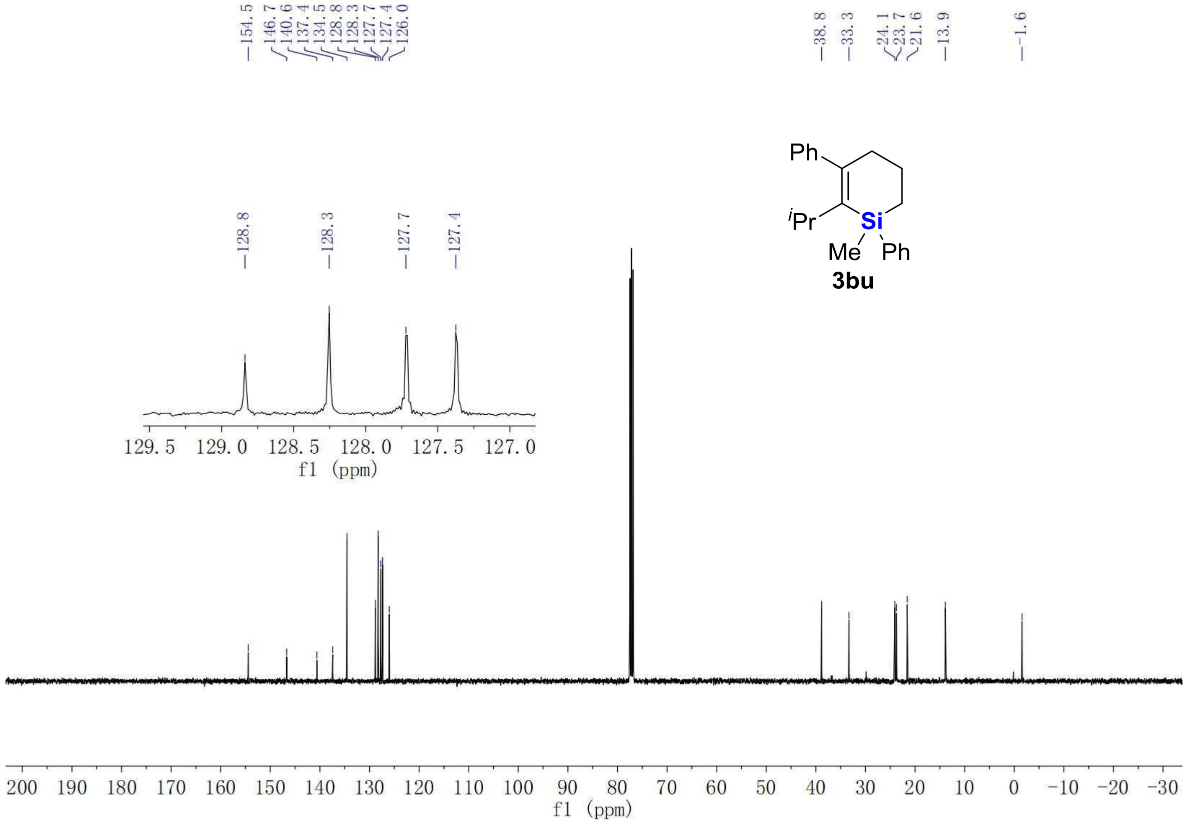

Supplementary Figure 31 ^{1}H and ^{13}C NMR Spectra for compound 3bu

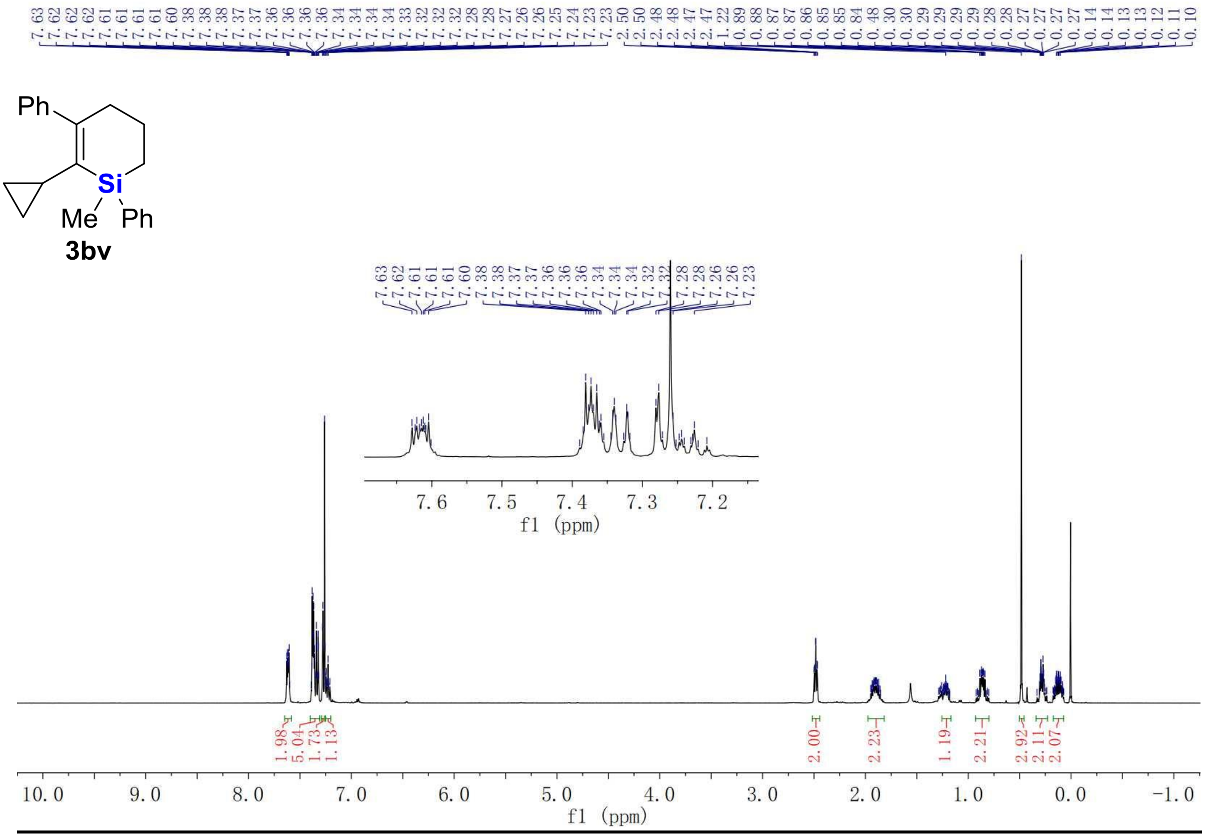

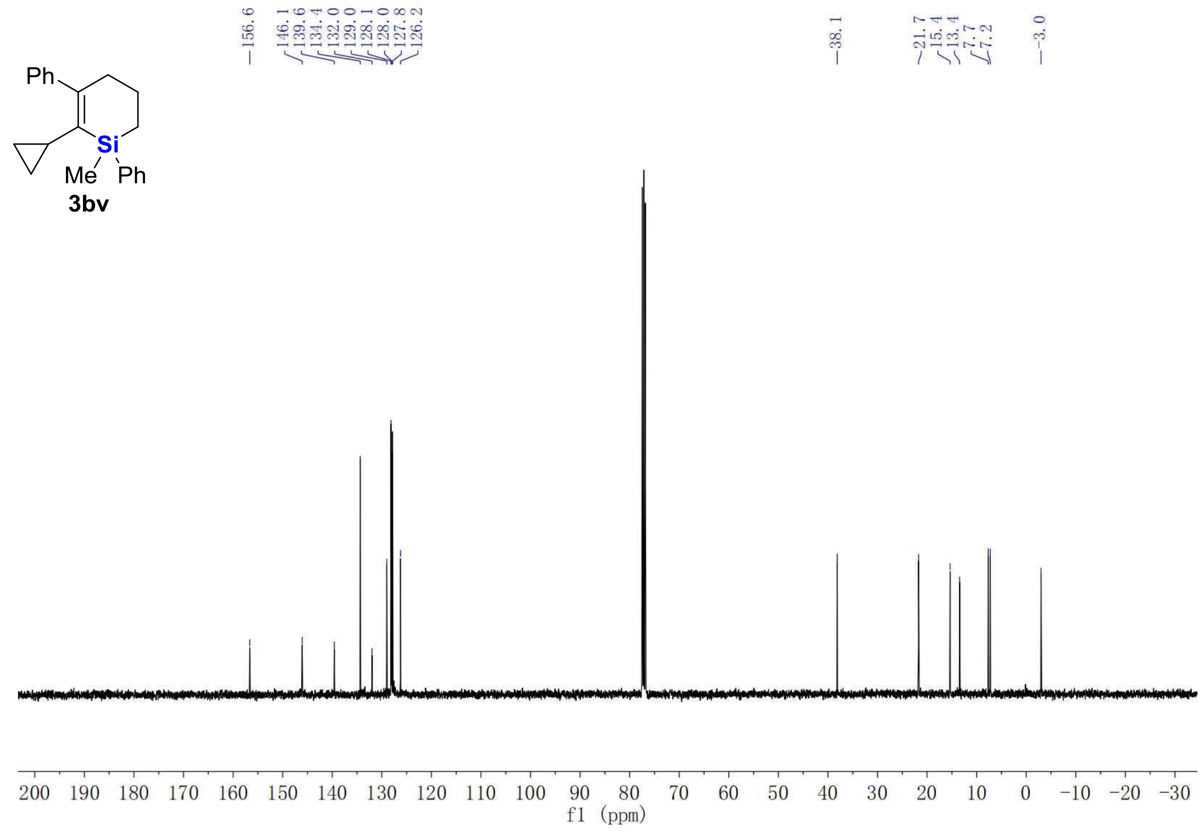

Supplementary Figure 32 ^{1}H and ^{13}C NMR Spectra for compound 3bv

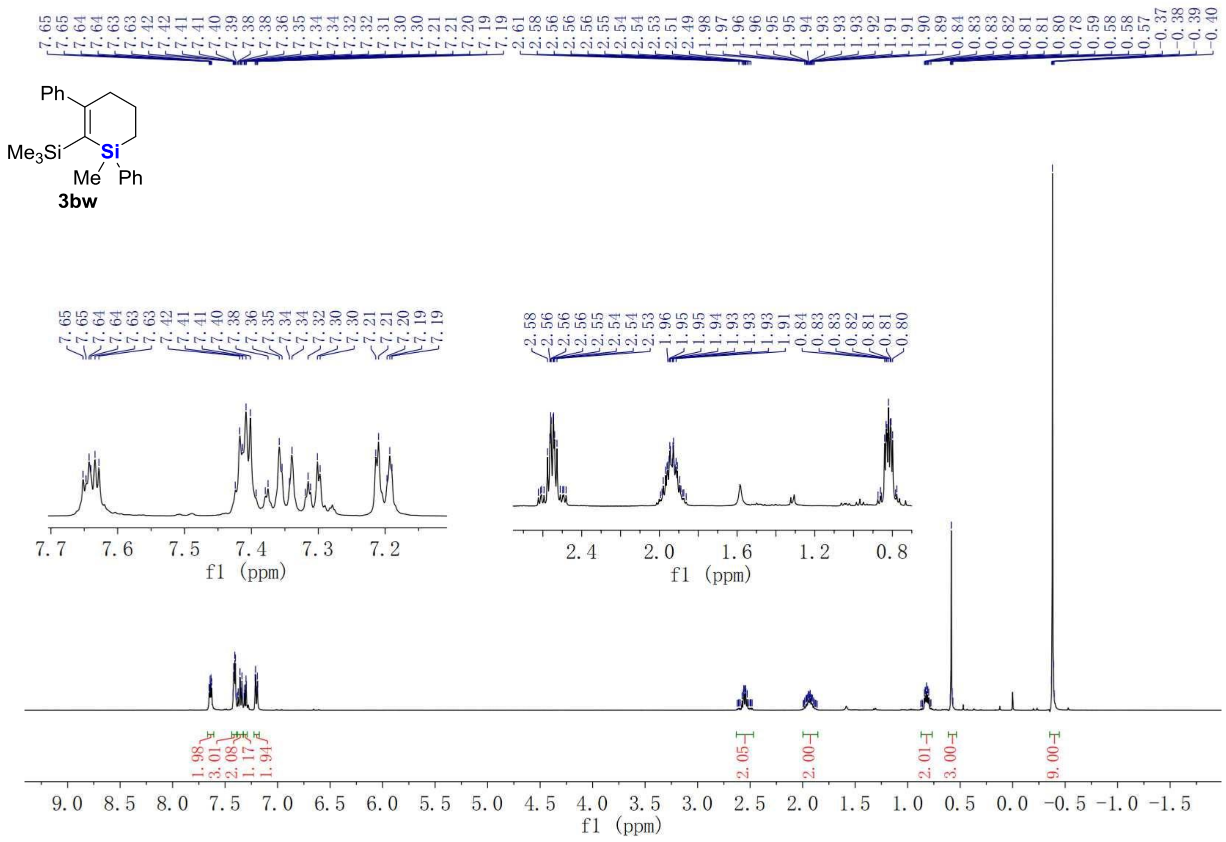

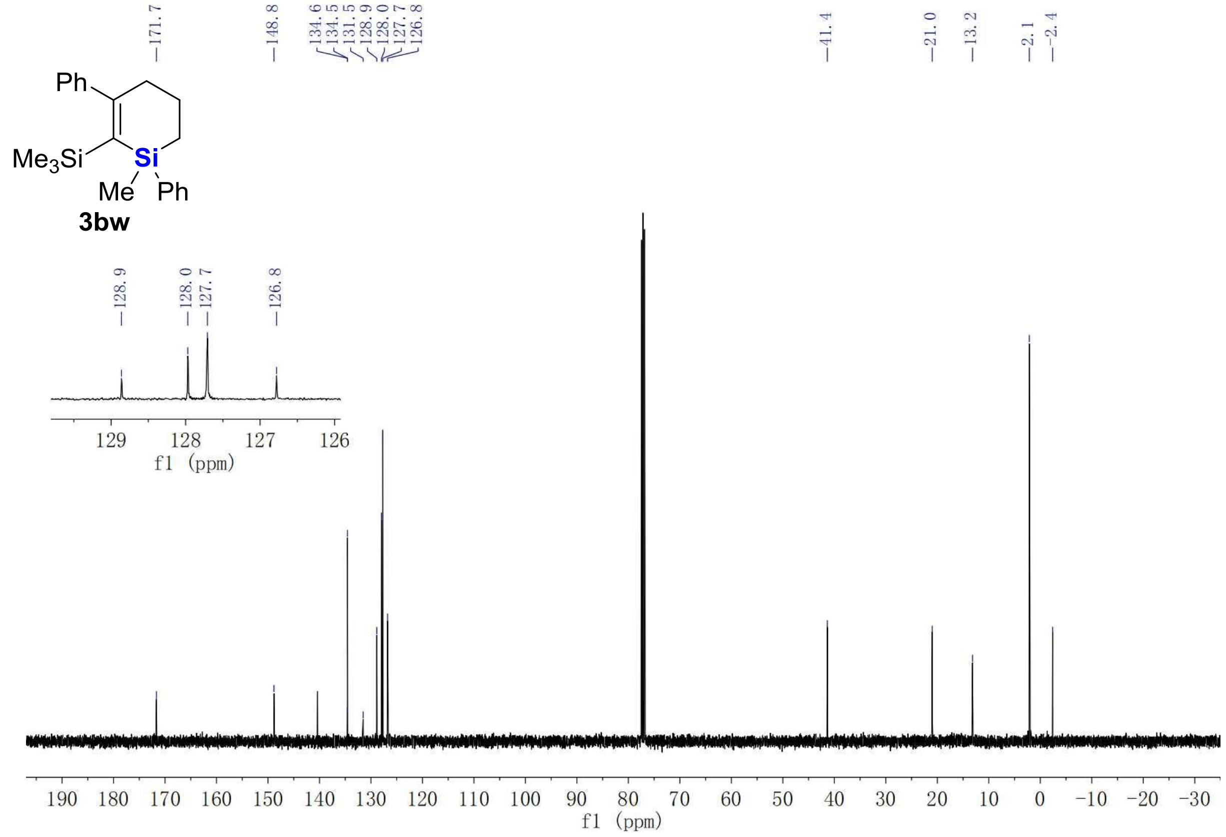

Supplementary Figure 33 ^{1}H and ^{13}C NMR Spectra for compound 3bw

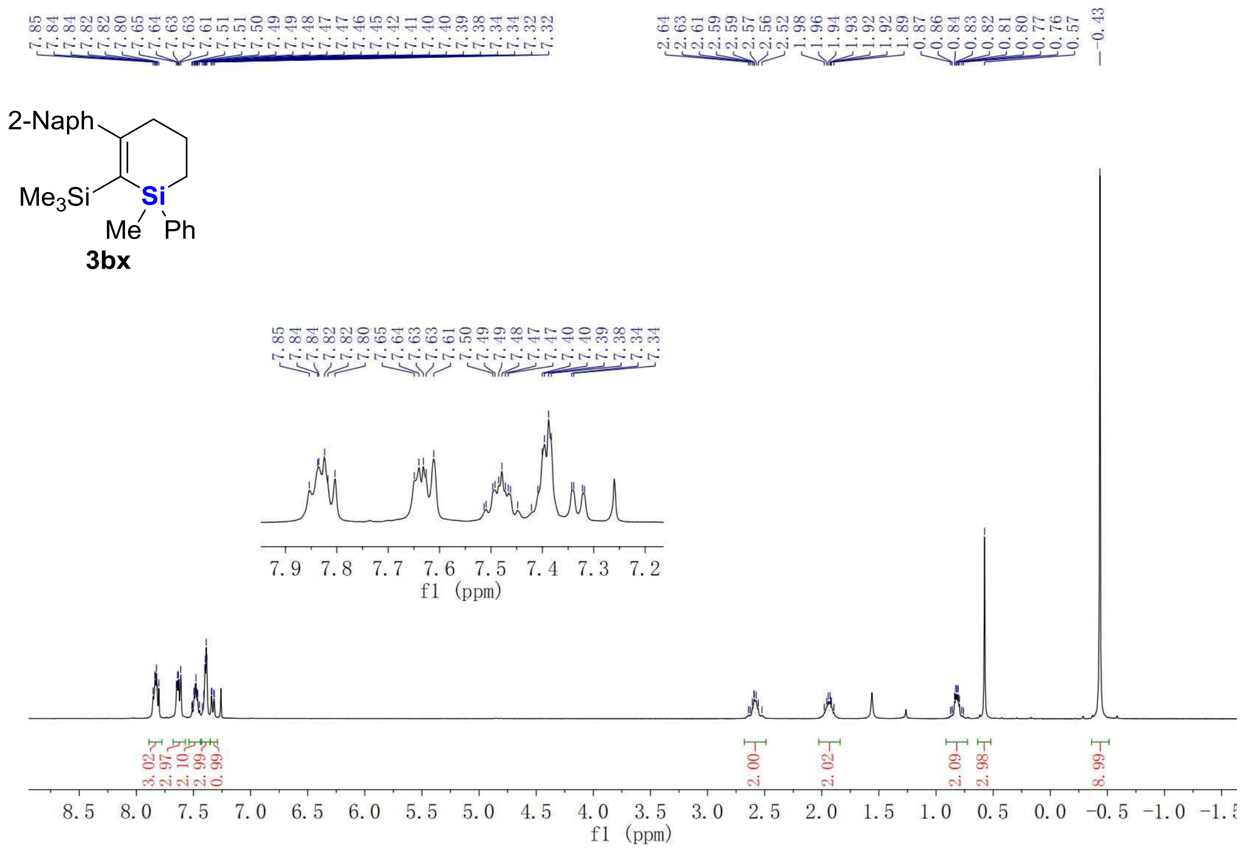

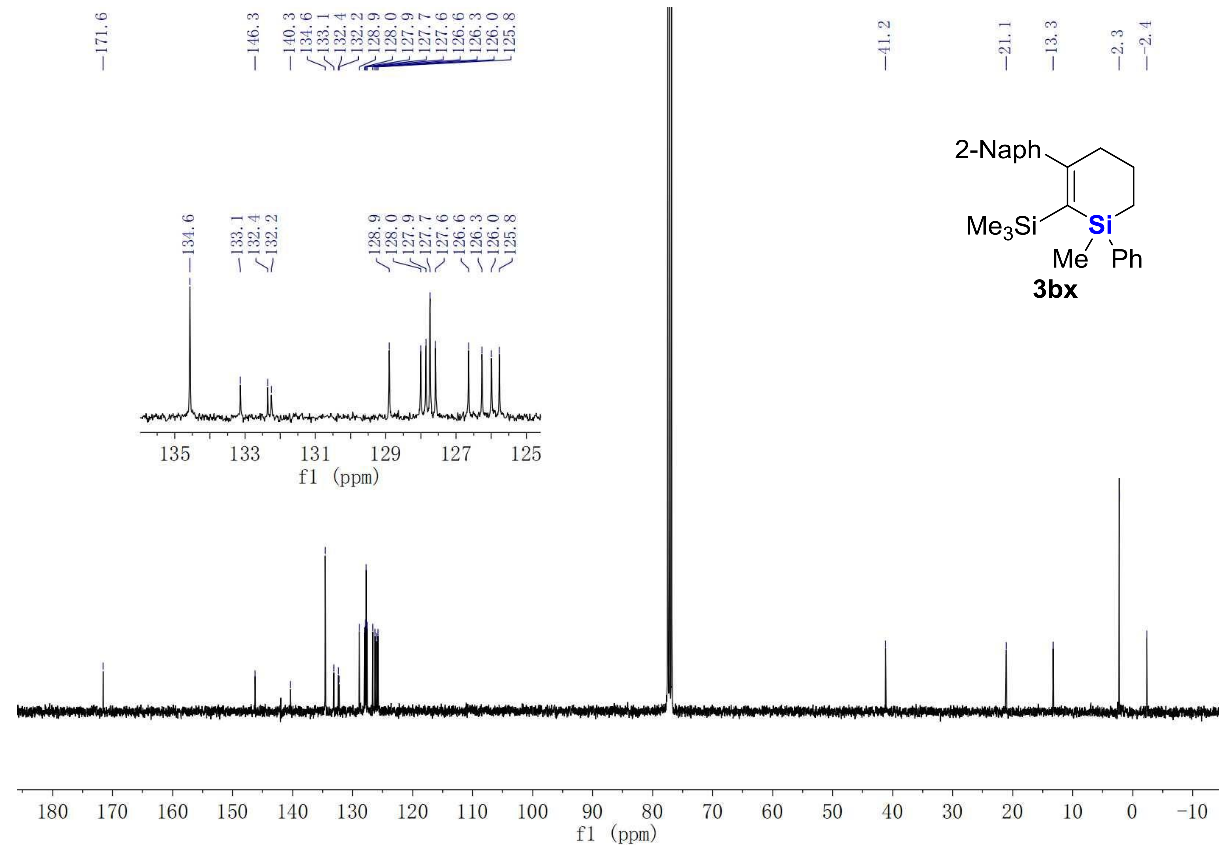

Supplementary Figure 34 ^{1}H and ^{13}C NMR Spectra for compound 3bx

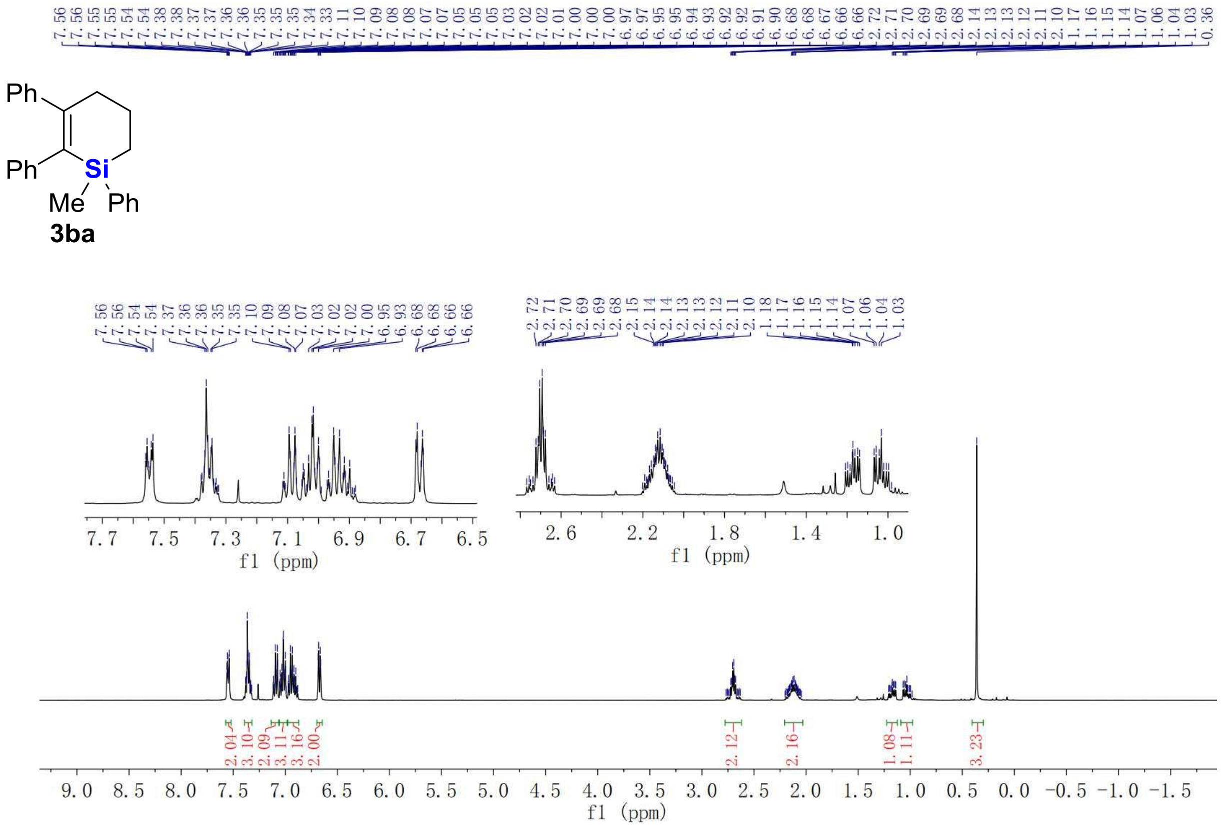

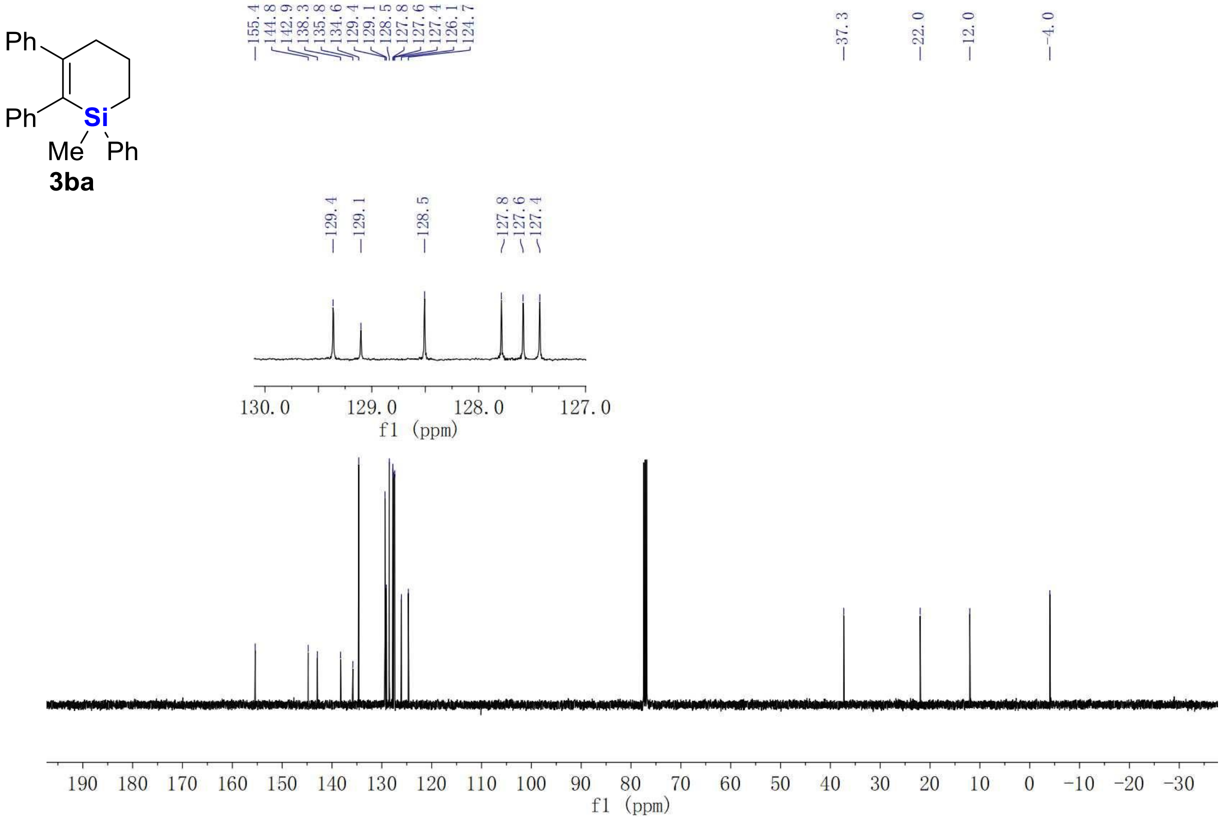

Supplementary Figure 35 ^{1}H and ^{13}C NMR Spectra for compound 3ba

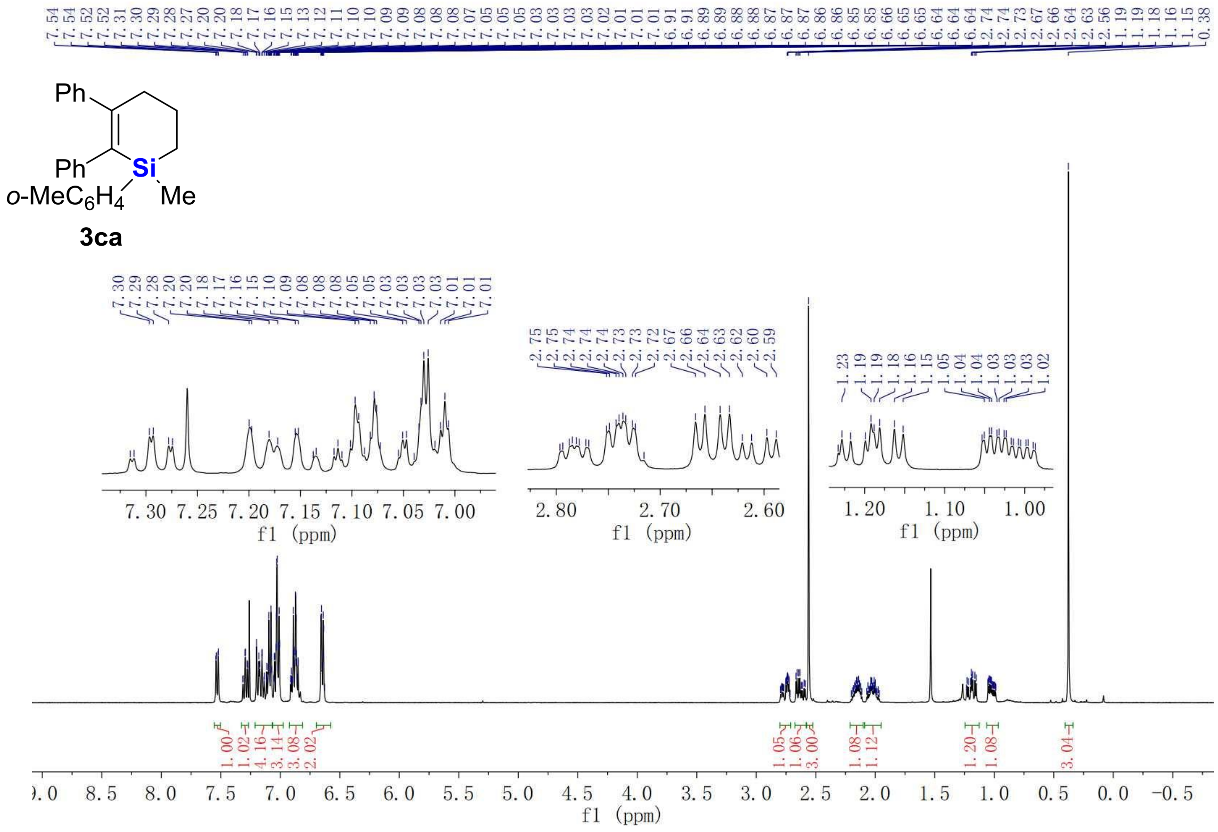

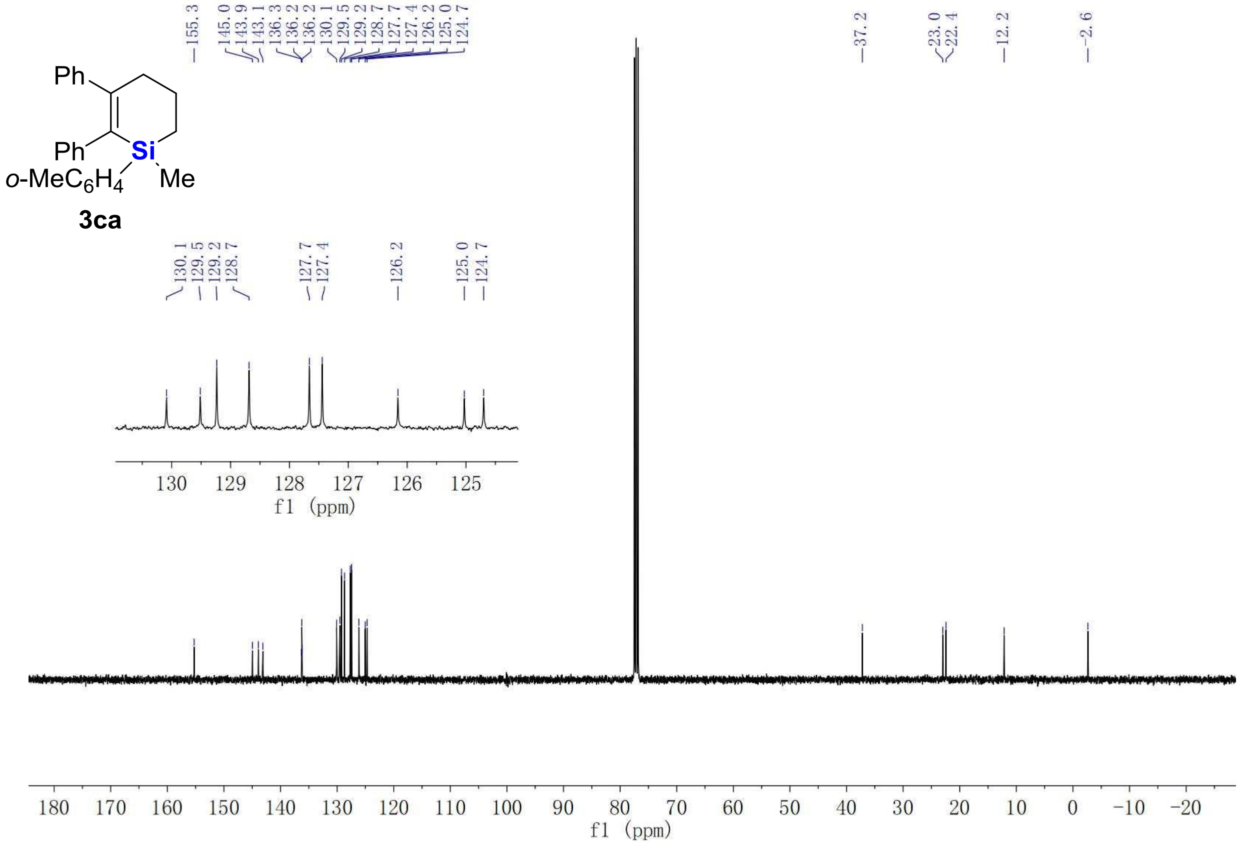

Supplementary Figure 36 ^{1}H and ^{13}C NMR Spectra for compound 3ca

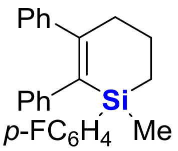

3da

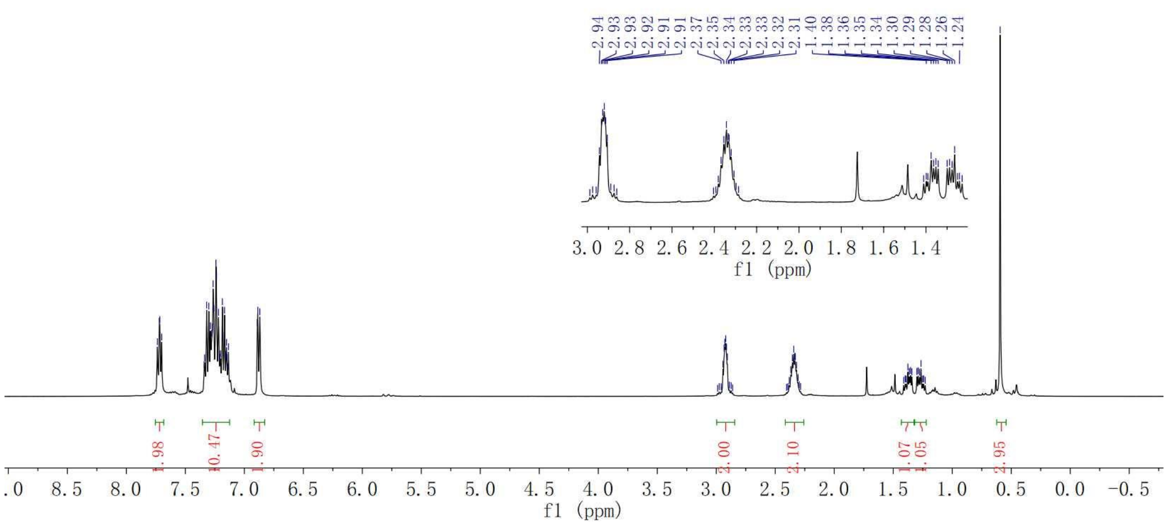

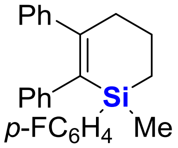

3da

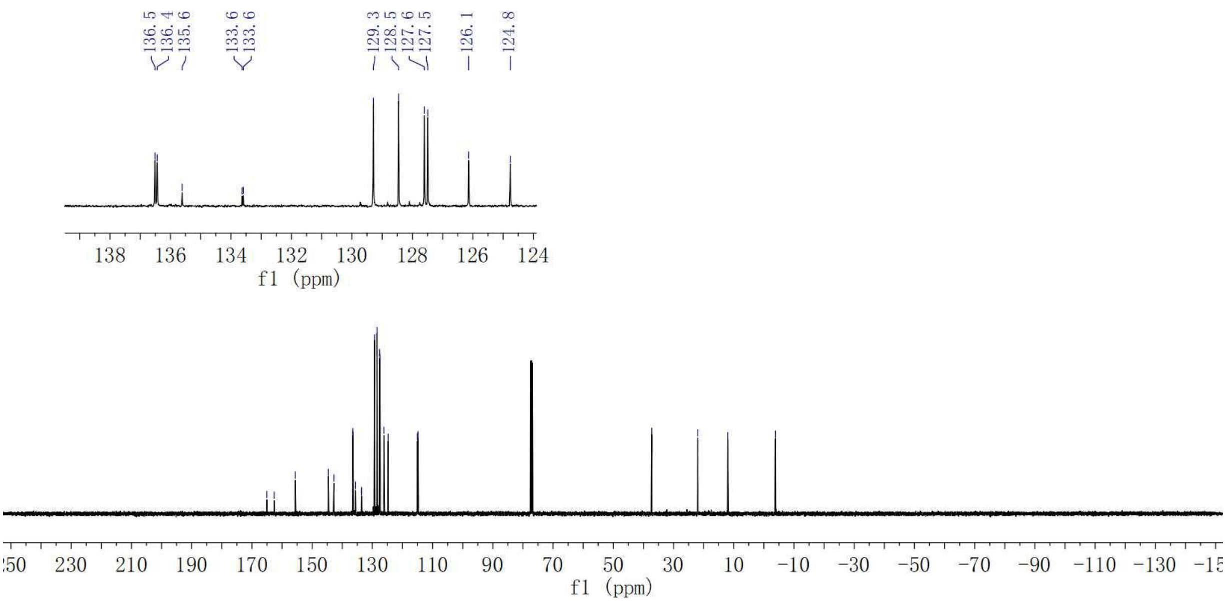

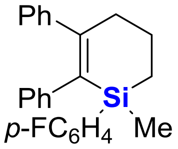

3da

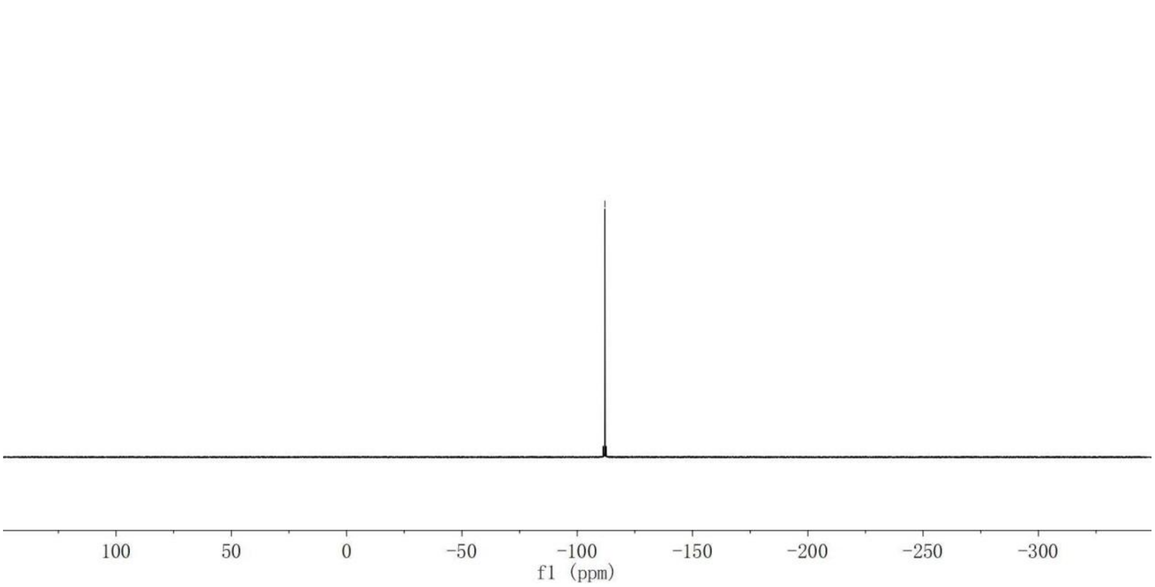

Supplementary Figure 37 ^{1}H, ^{13}C and ^{19}F NMR Spectra for compound 3da

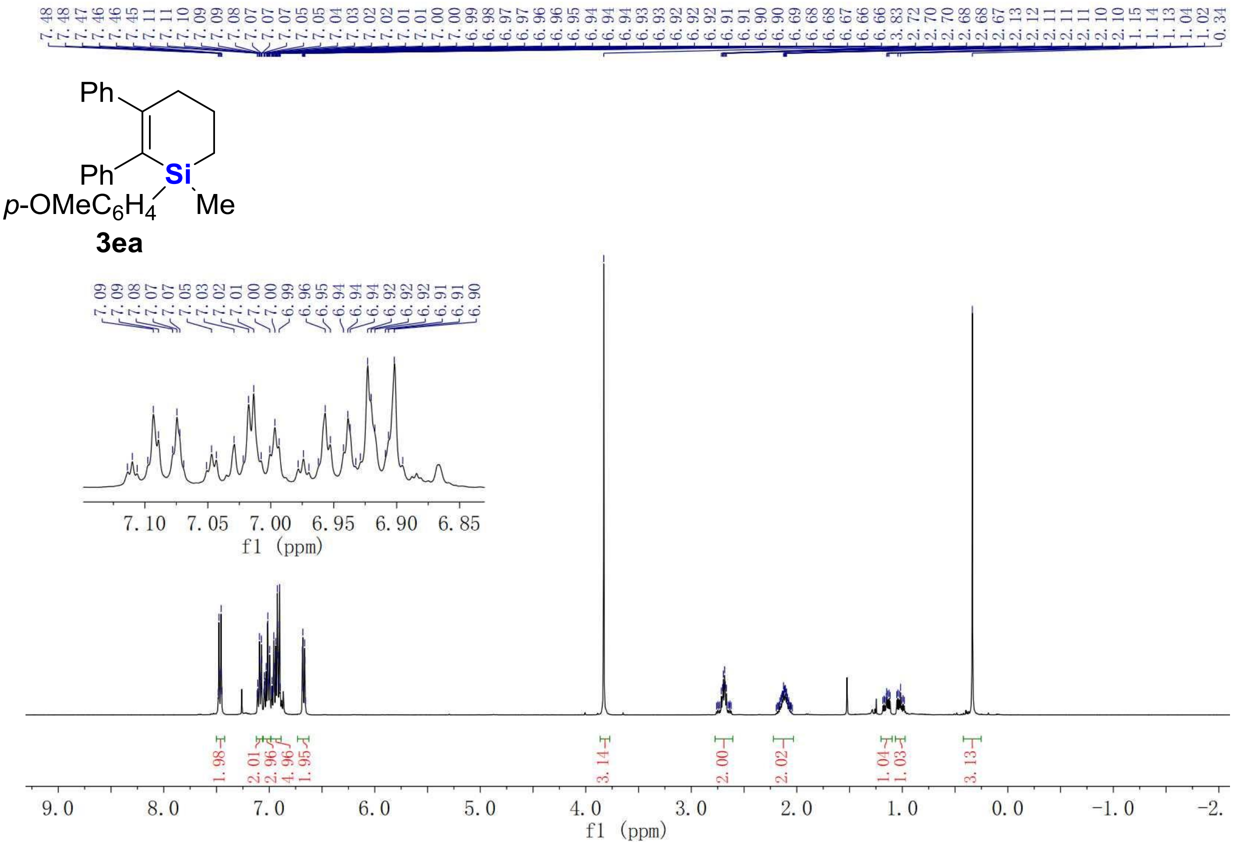

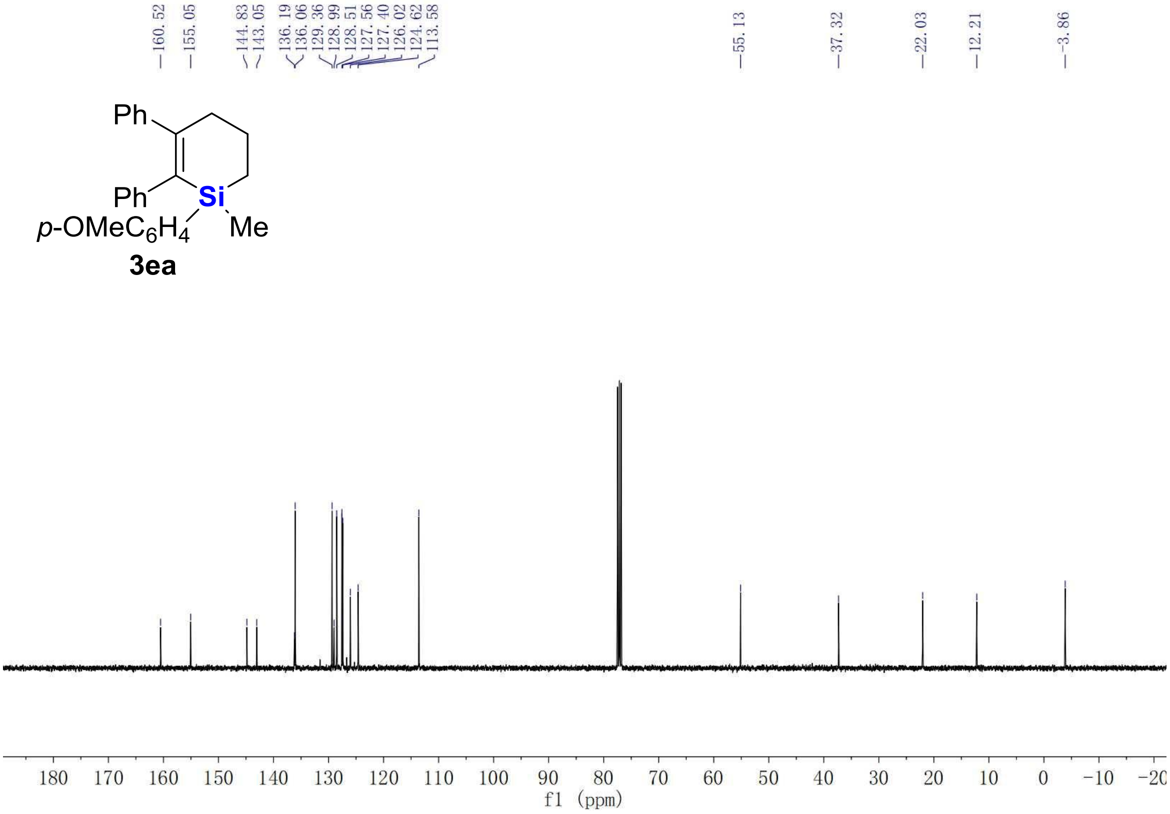

Supplementary Figure 38 ^{1}H and ^{13}C NMR Spectra for compound 3ea

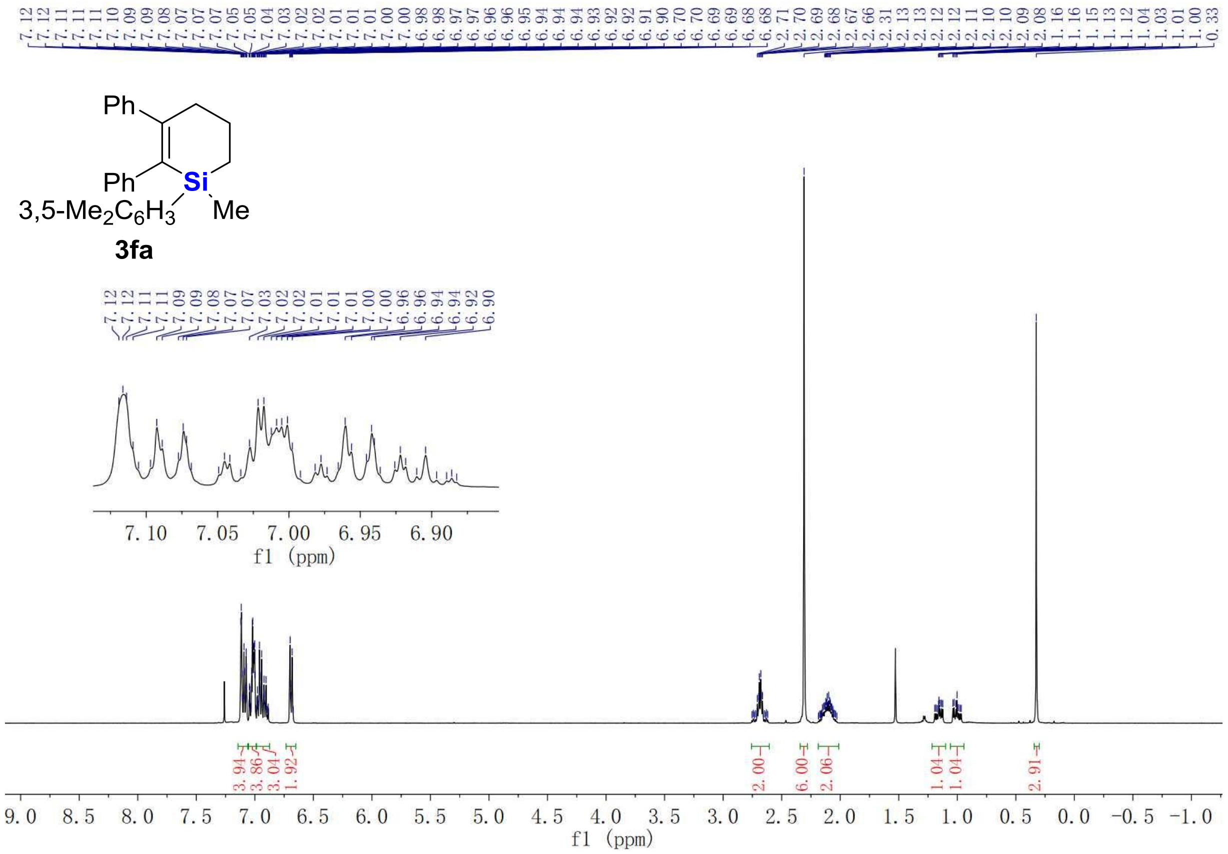

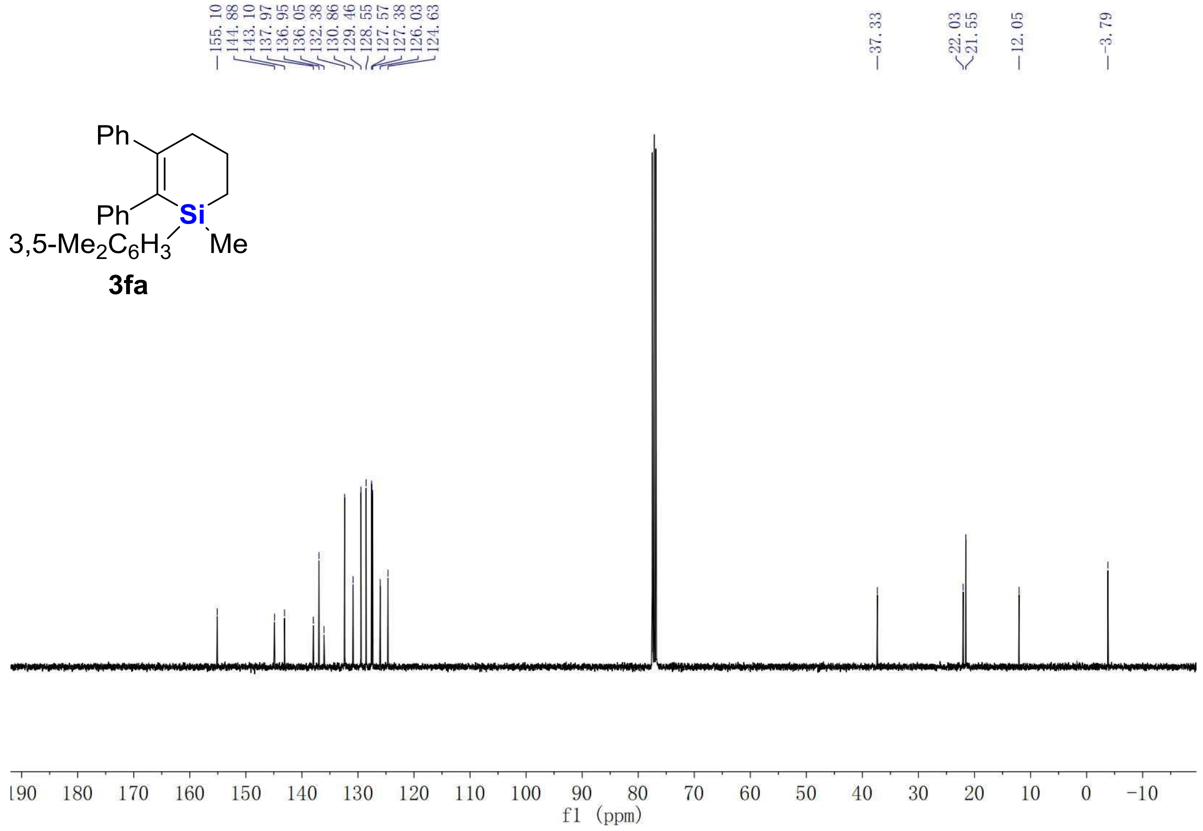

Supplementary Figure 39 ^{1}H and ^{13}C NMR Spectra for compound 3fa

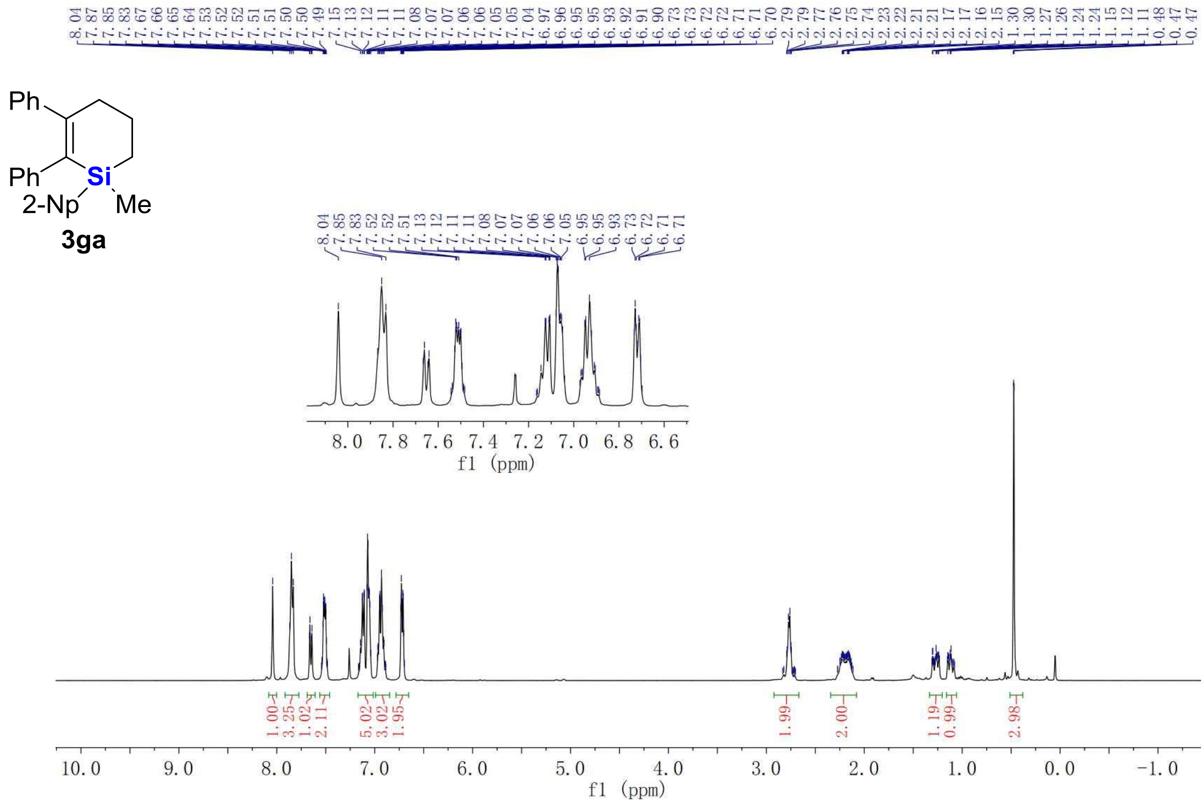

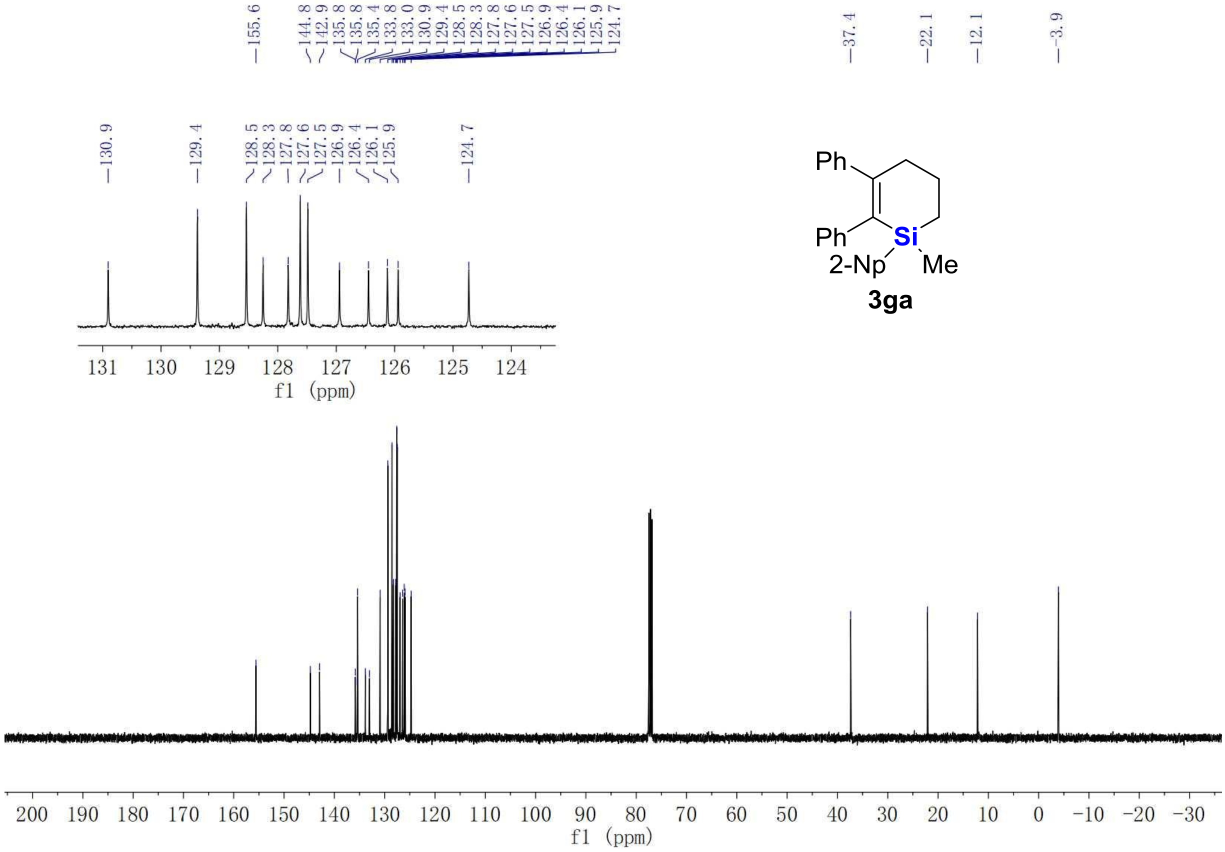

Supplementary Figure 40 ^{1}H and ^{13}C NMR Spectra for compound 3ga

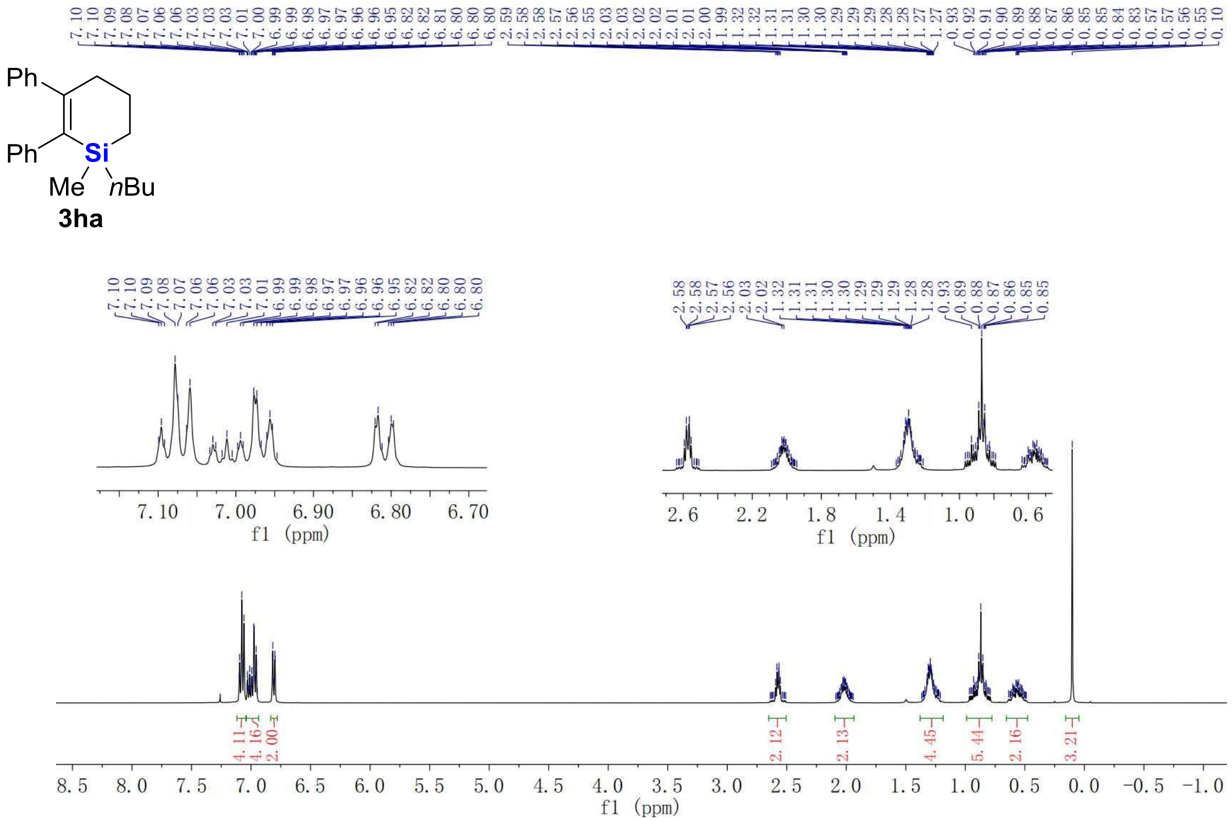

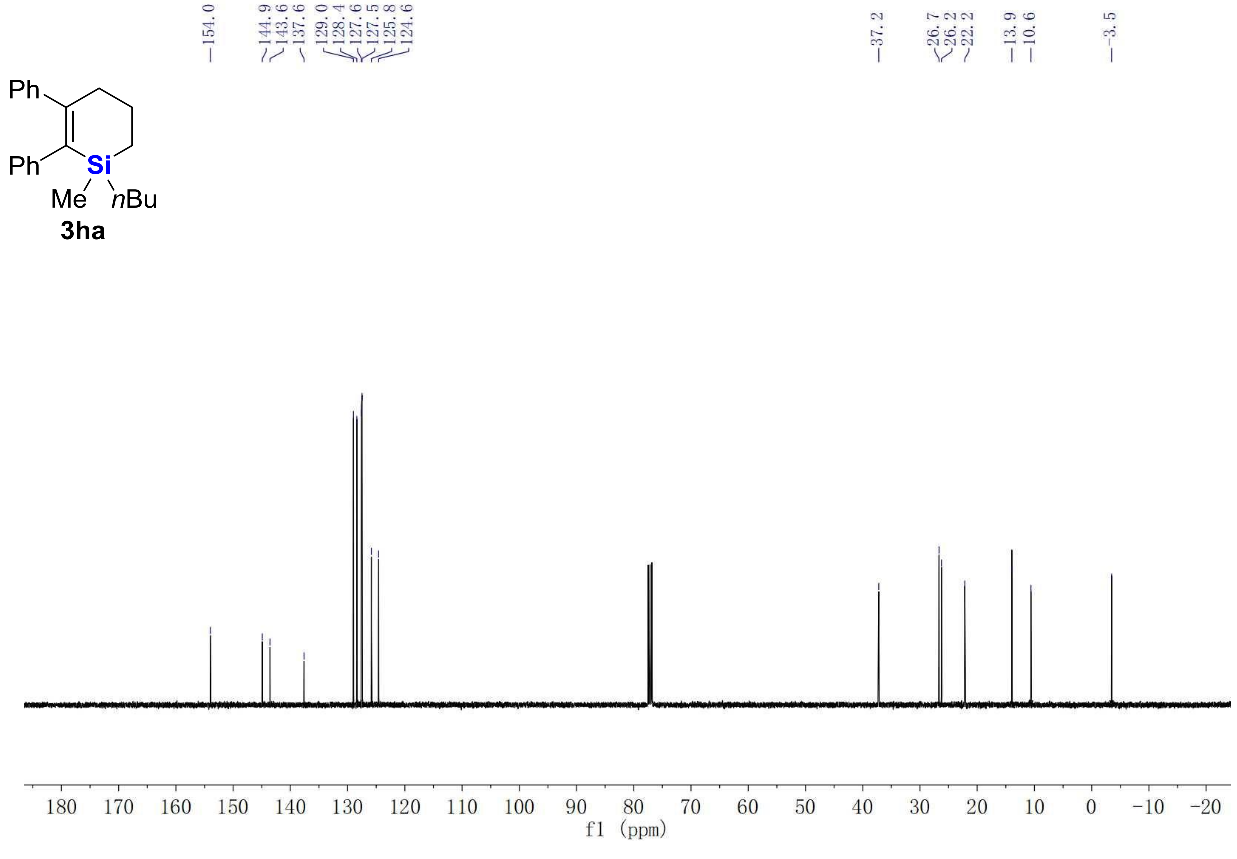

Supplementary Figure 41 ^{1}H and ^{13}C NMR Spectra for compound 3ha

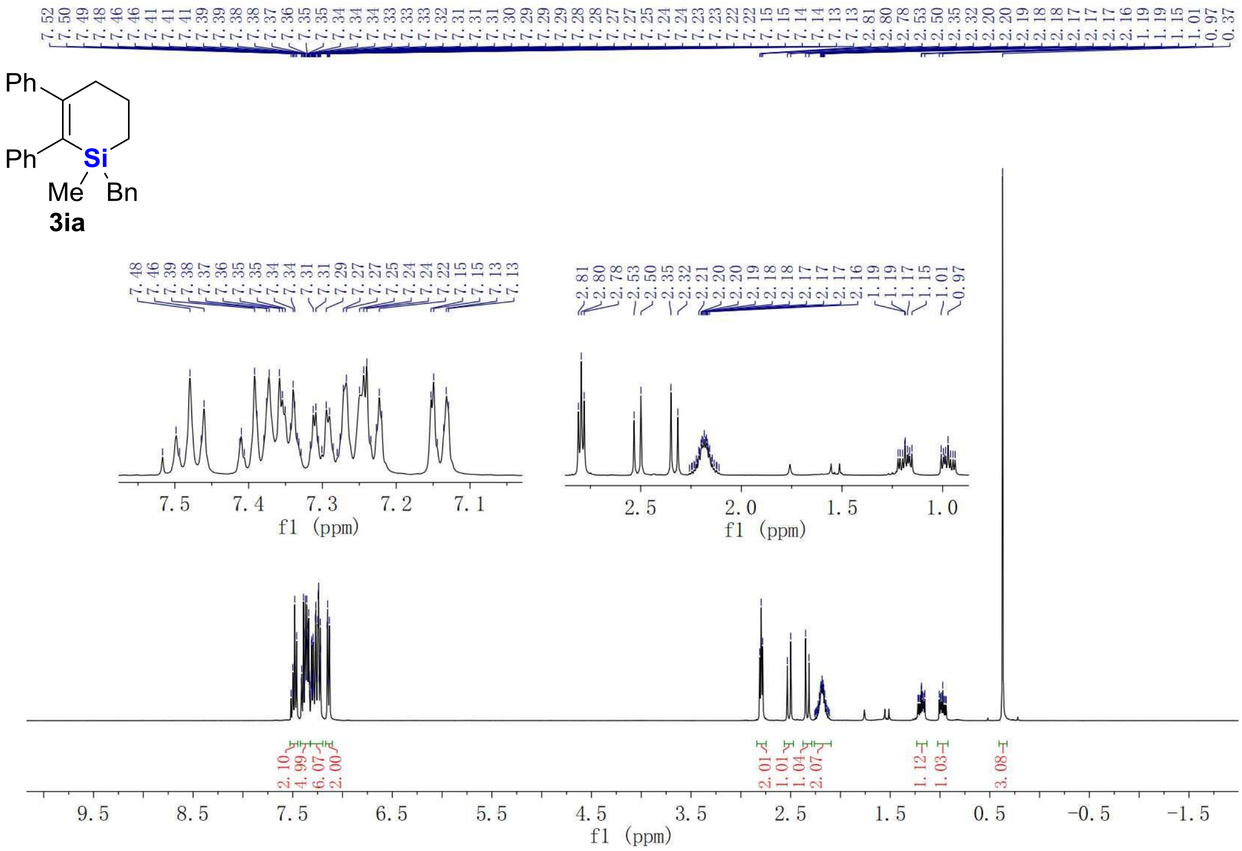

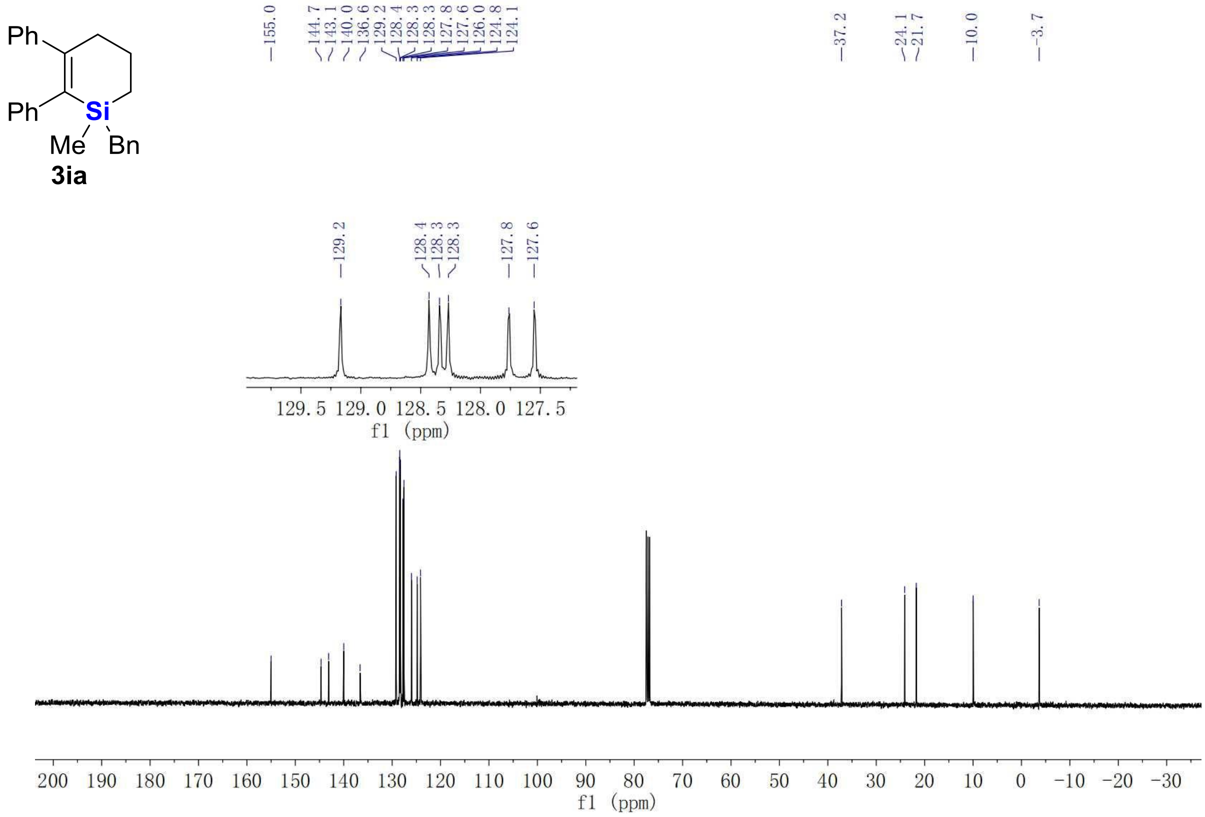

Supplementary Figure 42 ^{1}H and ^{13}C NMR Spectra for compound 3ia

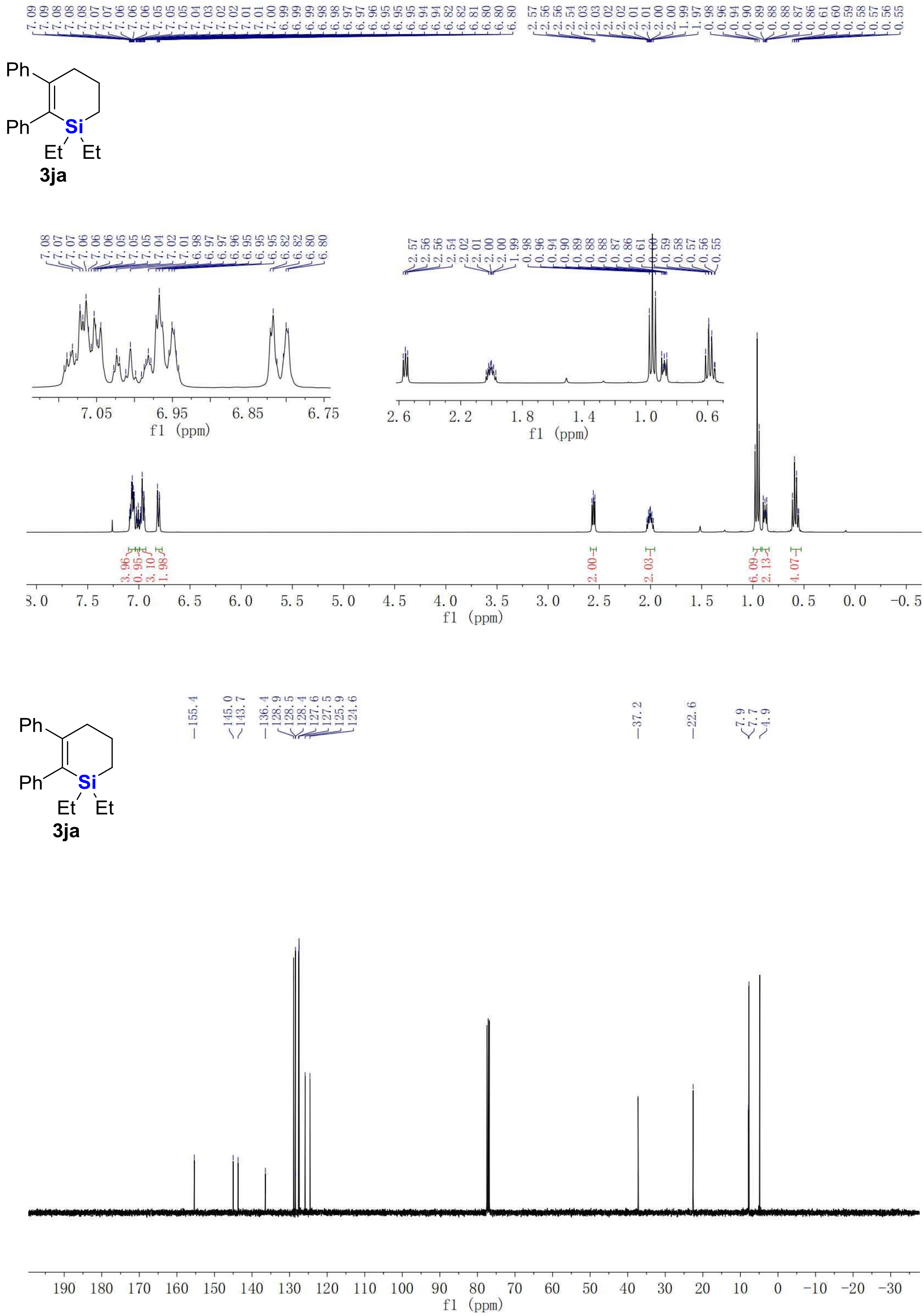

Supplementary Figure 43 ^{1}H and ^{13}C NMR Spectra for compound 3ja

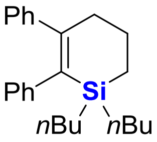

3ka

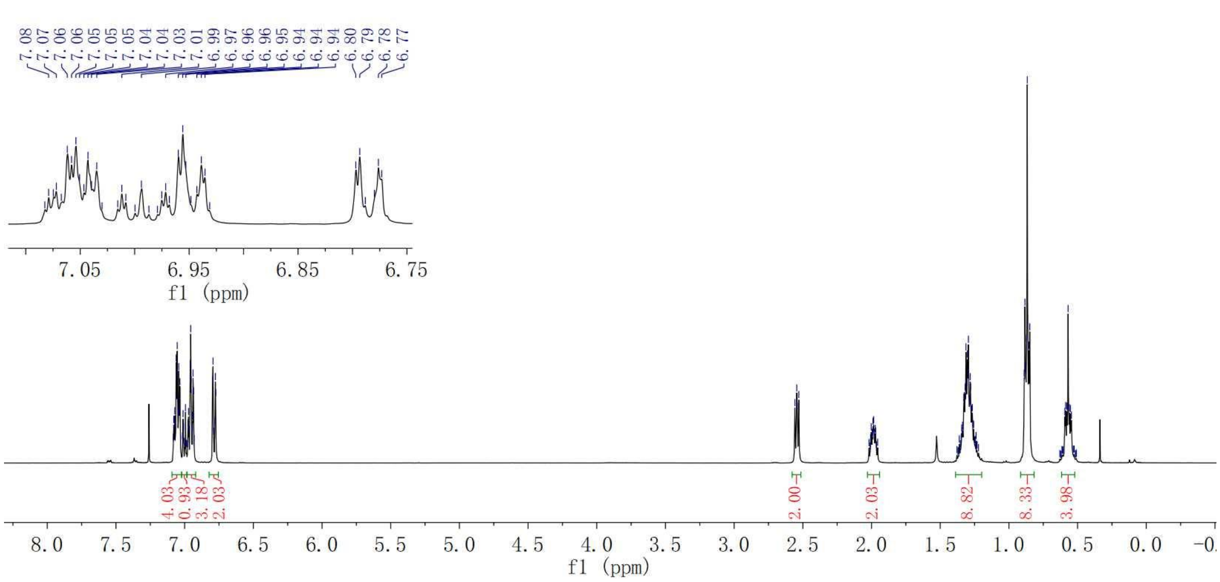

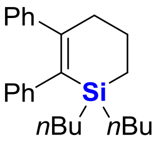

3ka

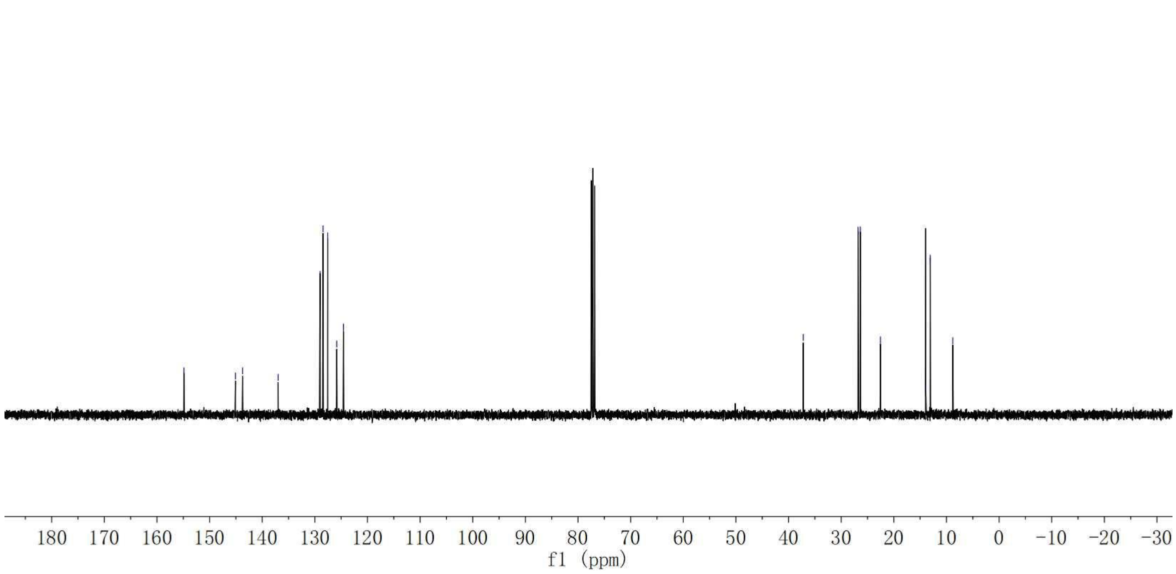

Supplementary Figure 44 ^{1}H and ^{13}C NMR Spectra for compound 3ka

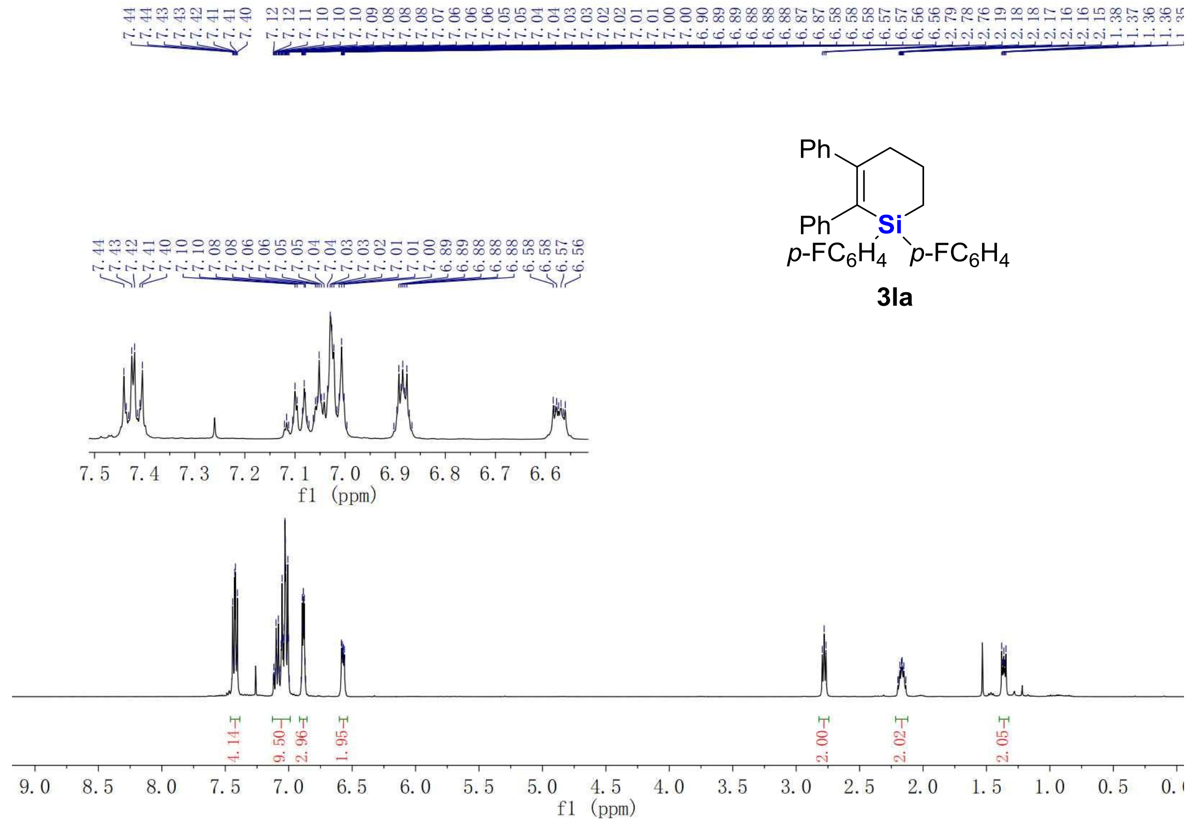
7.44 7.43 7.42 7.41 7.40 7.10 7.08 7.08 7.06 7.06 7.05 7.05 7.04 7.04 7.03 7.02 7.01 7.00 6.89 6.89 6.88 6.88 6.88 6.58 6.58 6.57 6.56
7.5 7.4 7.3 7.2 7.1 7.0 6.9 6.8 6.7 6.6
f1 (ppm)
4.14 9.50 2.96 1.95 2.00 2.02 2.05
9.0 8.5 8.0 7.5 7.0 6.5 6.0 5.5 5.0 4.5 4.0 3.5 3.0 2.5 2.0 1.5 1.0 0.5 0.0
f1 (ppm)
Ph
Ph
Si
p-FC6H4
p-FC6H4

3la

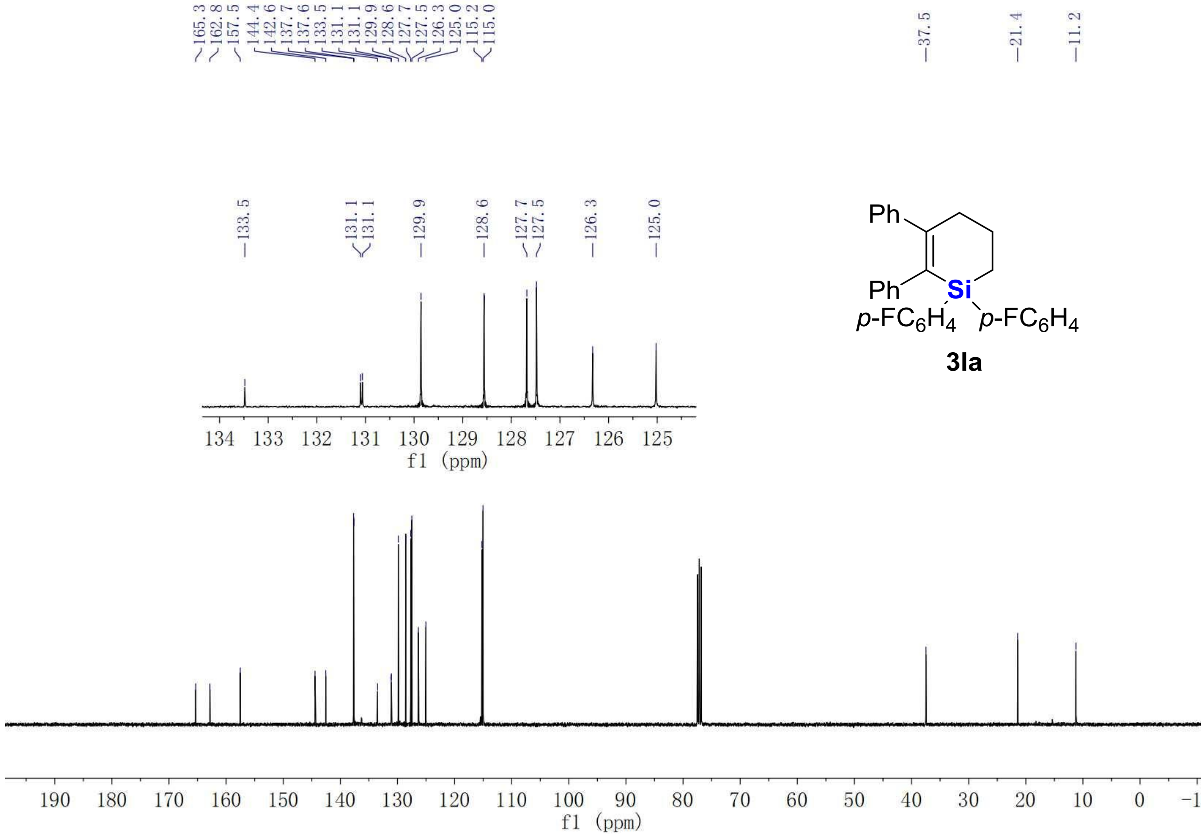
165.3 162.8 157.5 144.4 142.6 137.7 137.6 133.5 131.1 131.1 129.9 128.6 127.7 127.5 126.3 125.0 115.2 115.0
37.5
21.4
11.2
133.5 131.1 131.1 129.9 128.6 127.7 127.5 126.3 125.0
134 133 132 131 130 129 128 127 126 125
f1 (ppm)
190 180 170 160 150 140 130 120 110 100 90 80 70 60 50 40 30 20 10 0 -1
f1 (ppm)
Ph
Ph
Si
p-FC6H4
p-FC6H4

3la

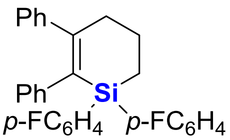

3la

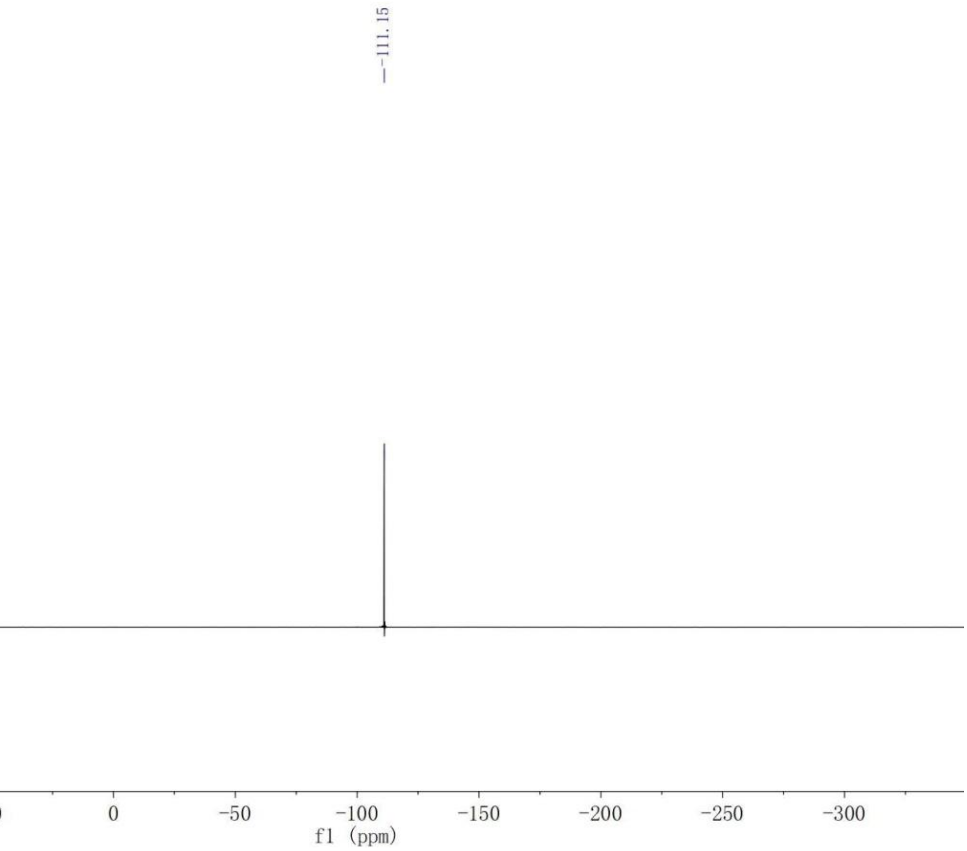

Supplementary Figure 45 ^{1}H, ^{13}C and ^{19}F NMR Spectra for compound 3la

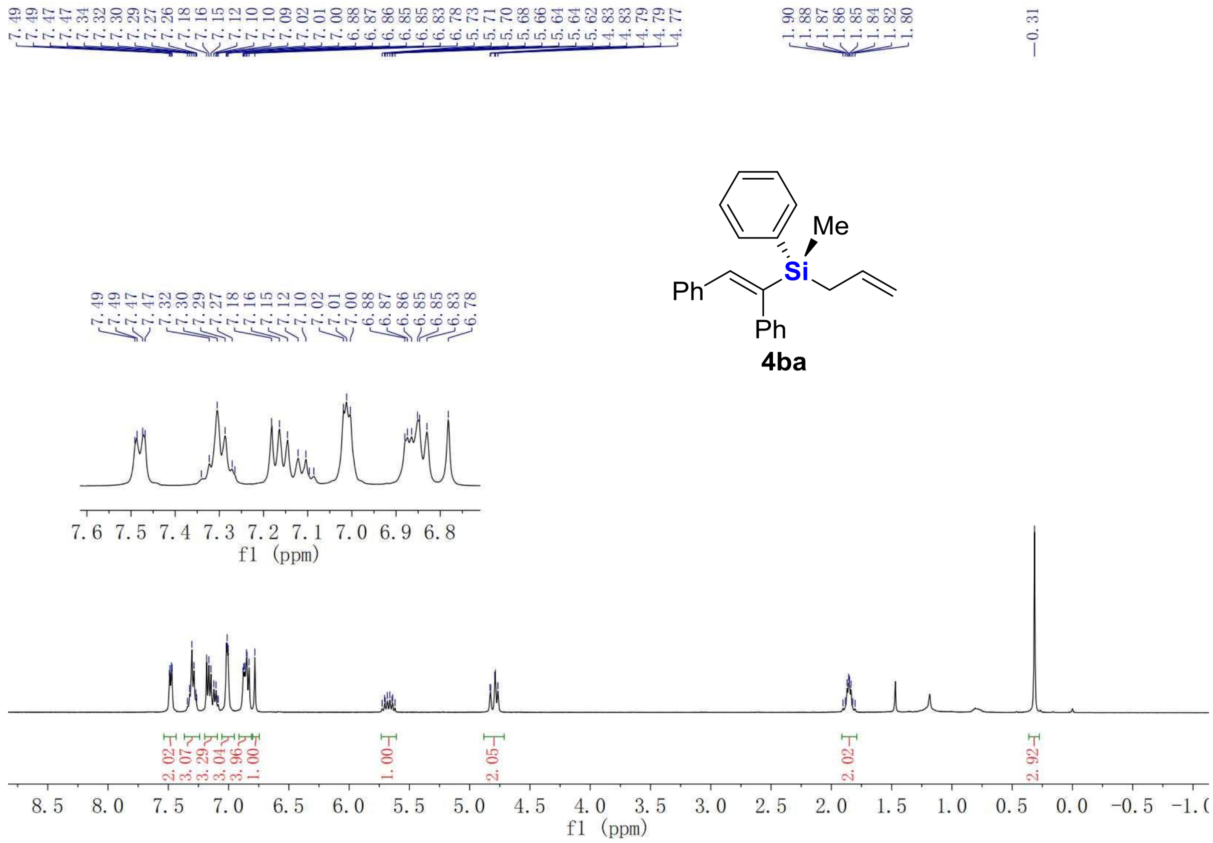

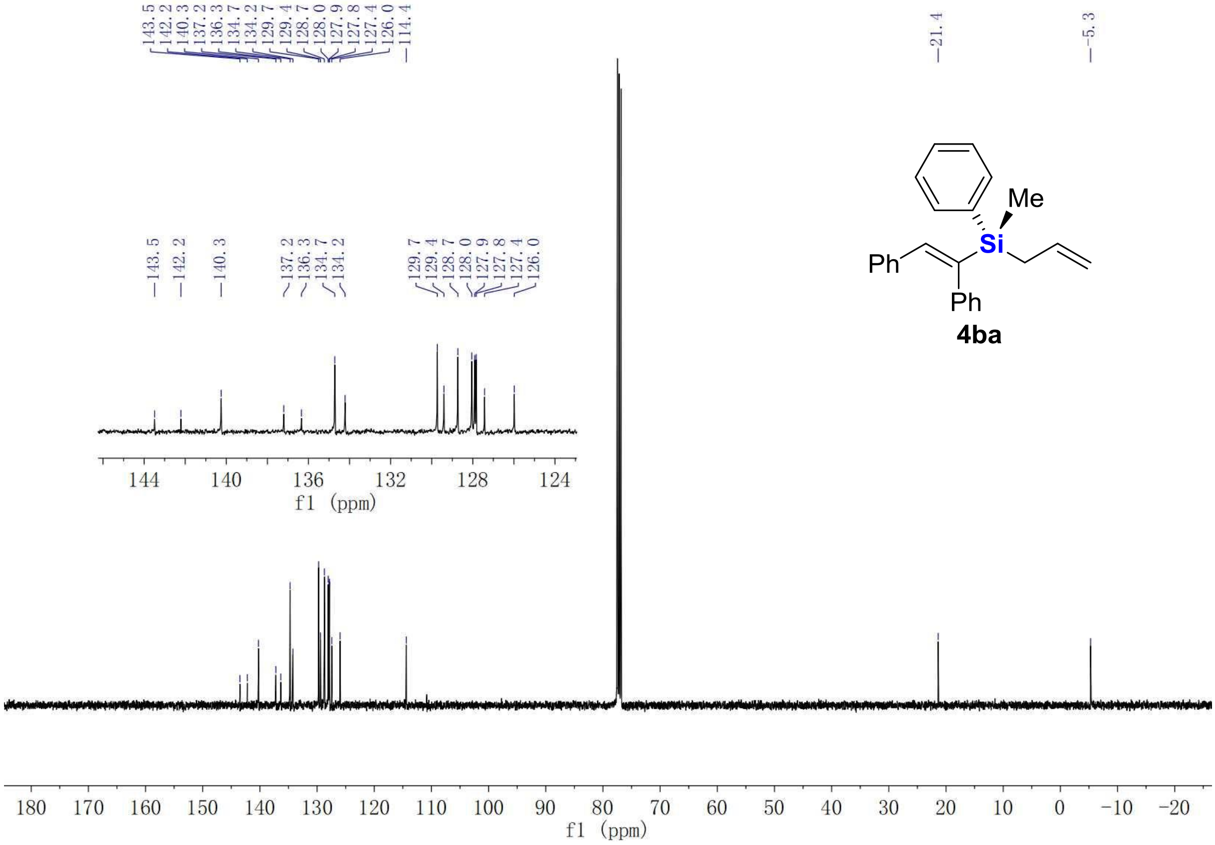

Supplementary Figure 46 ^{1}H and ^{13}C NMR Spectra for compound 4ba

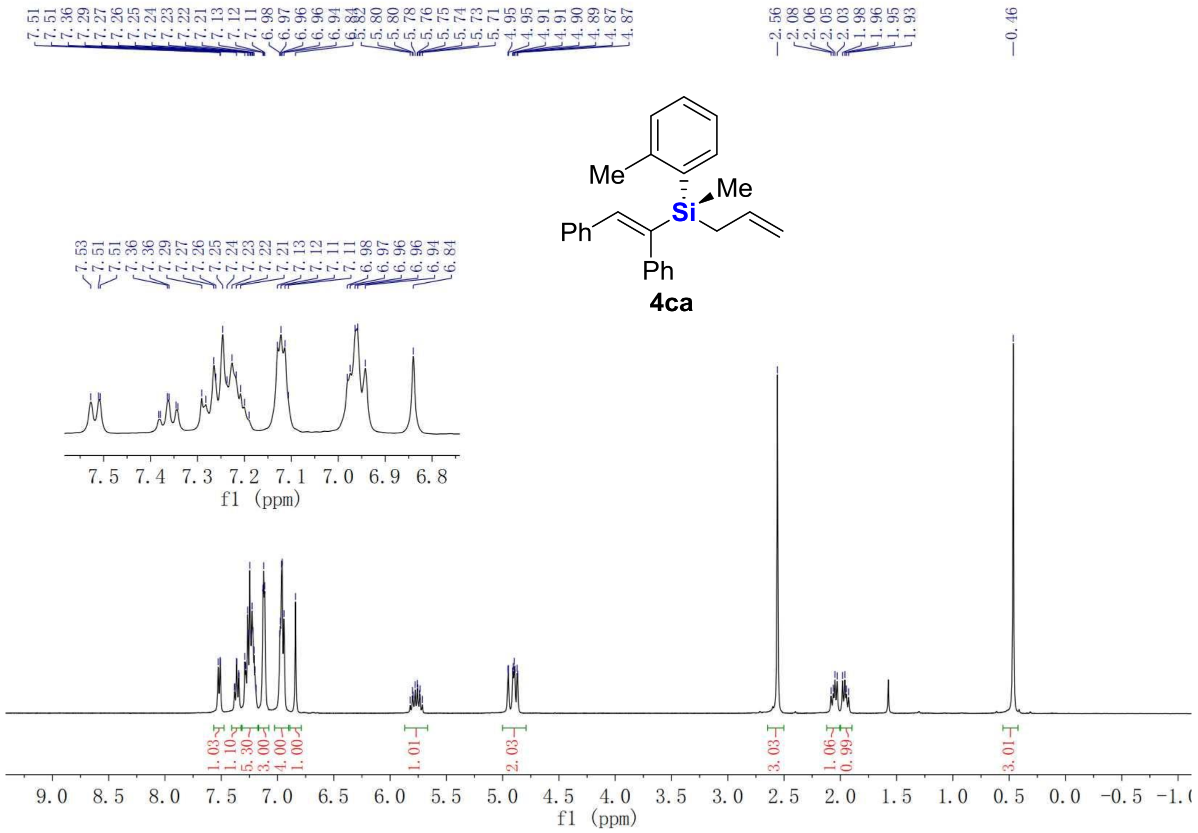

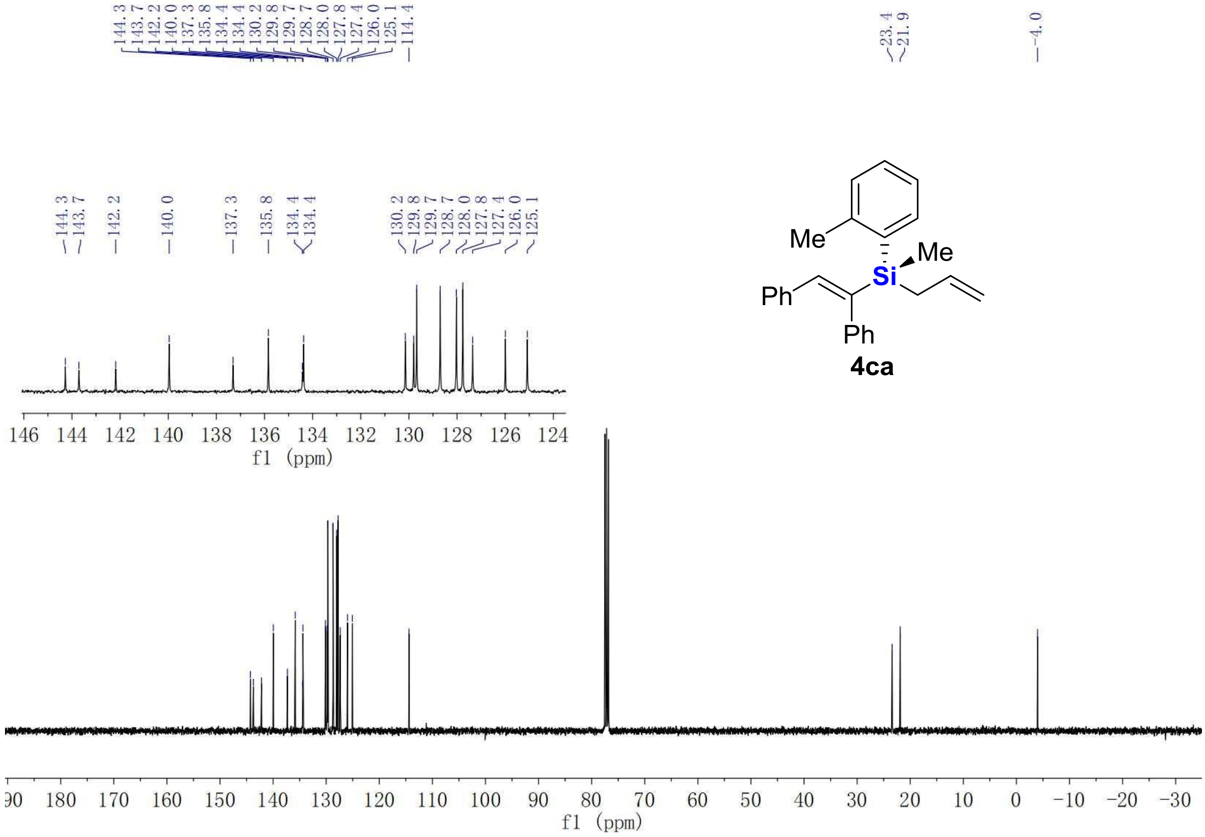

Supplementary Figure 47 ^{1}H and ^{13}C NMR Spectra for compound 4ca

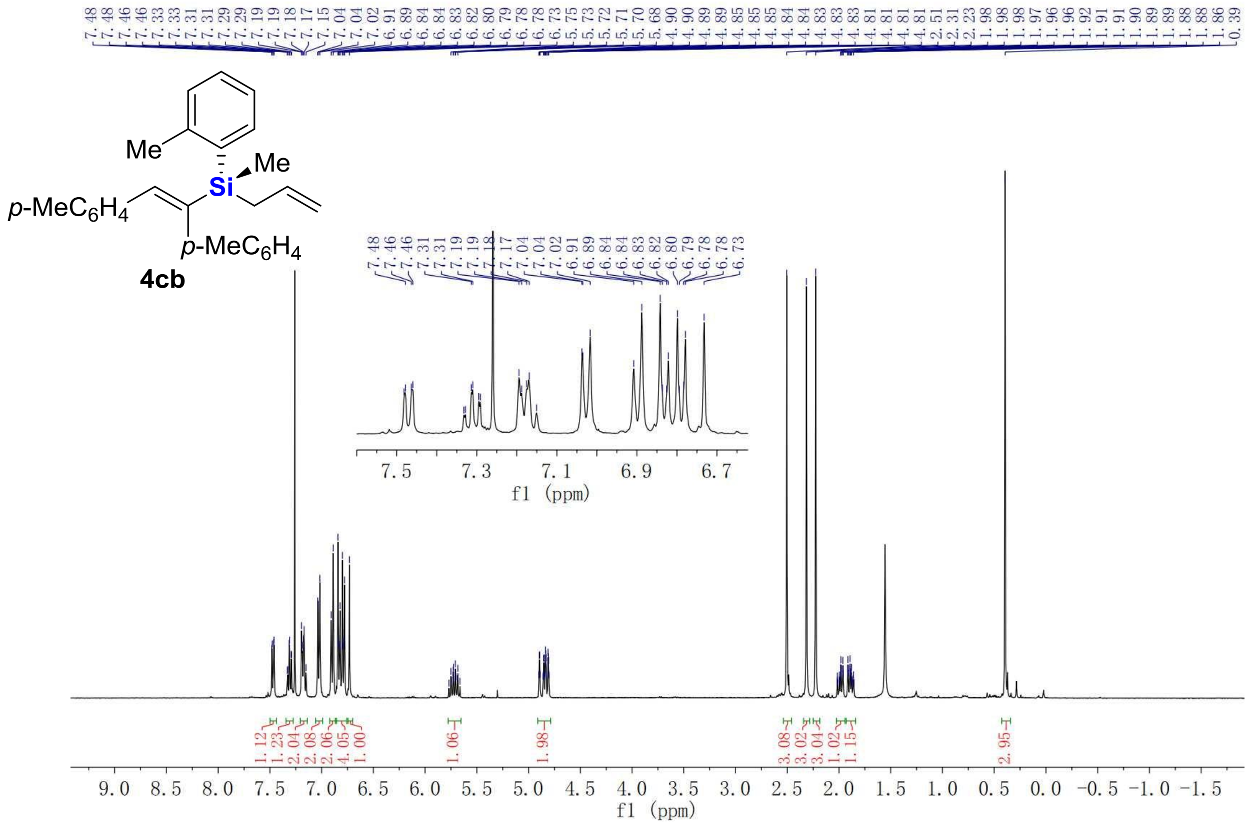

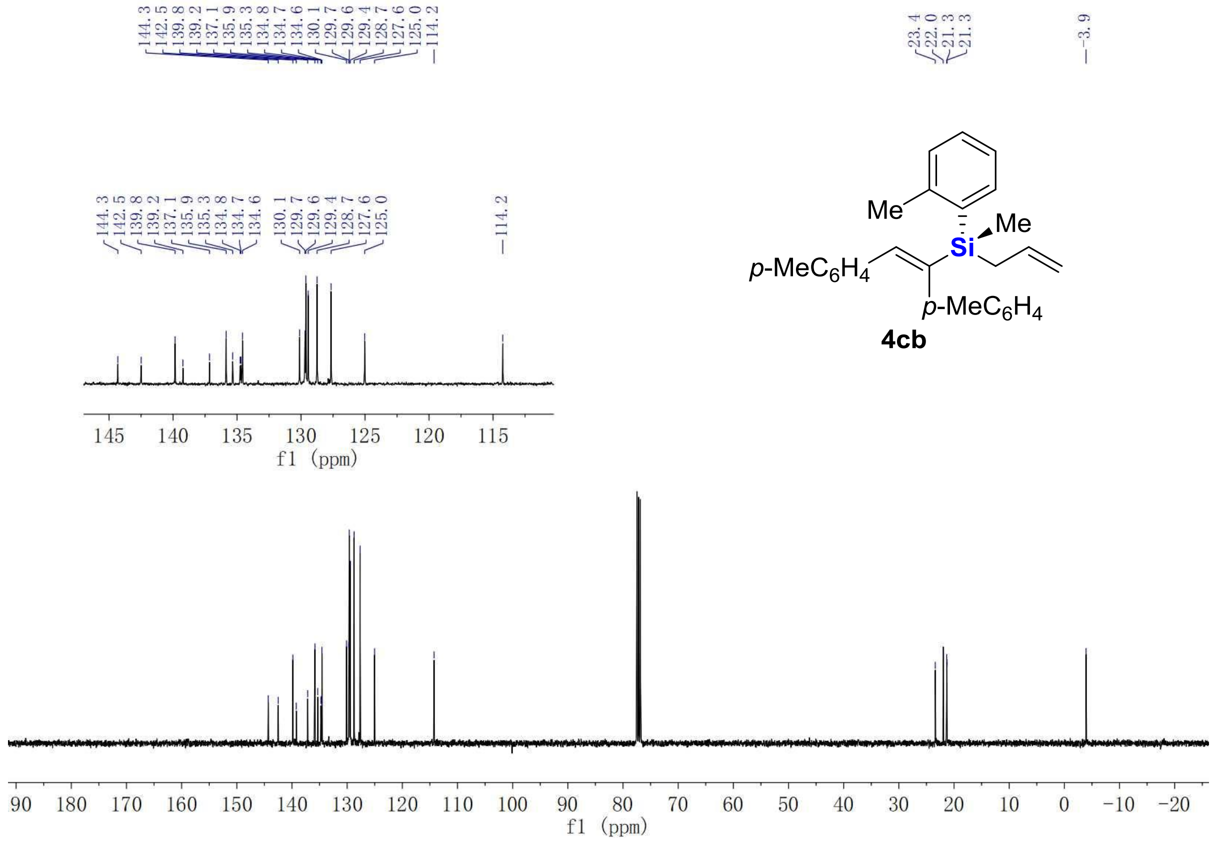

Supplementary Figure 48 ^{1}H and ^{13}C NMR Spectra for compound 4cb

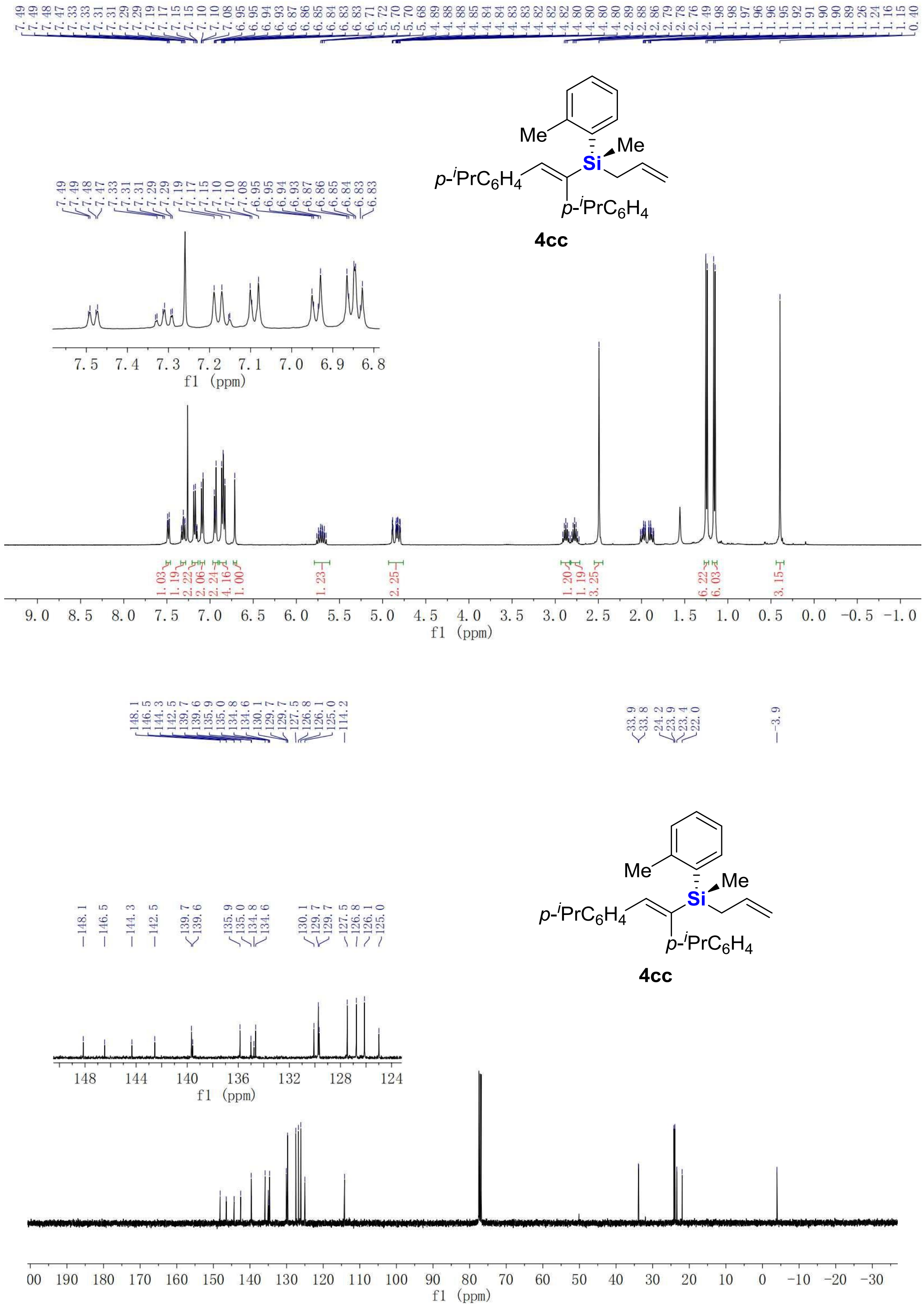

Supplementary Figure 49 ^{1}H and ^{13}C NMR Spectra for compound 4cc

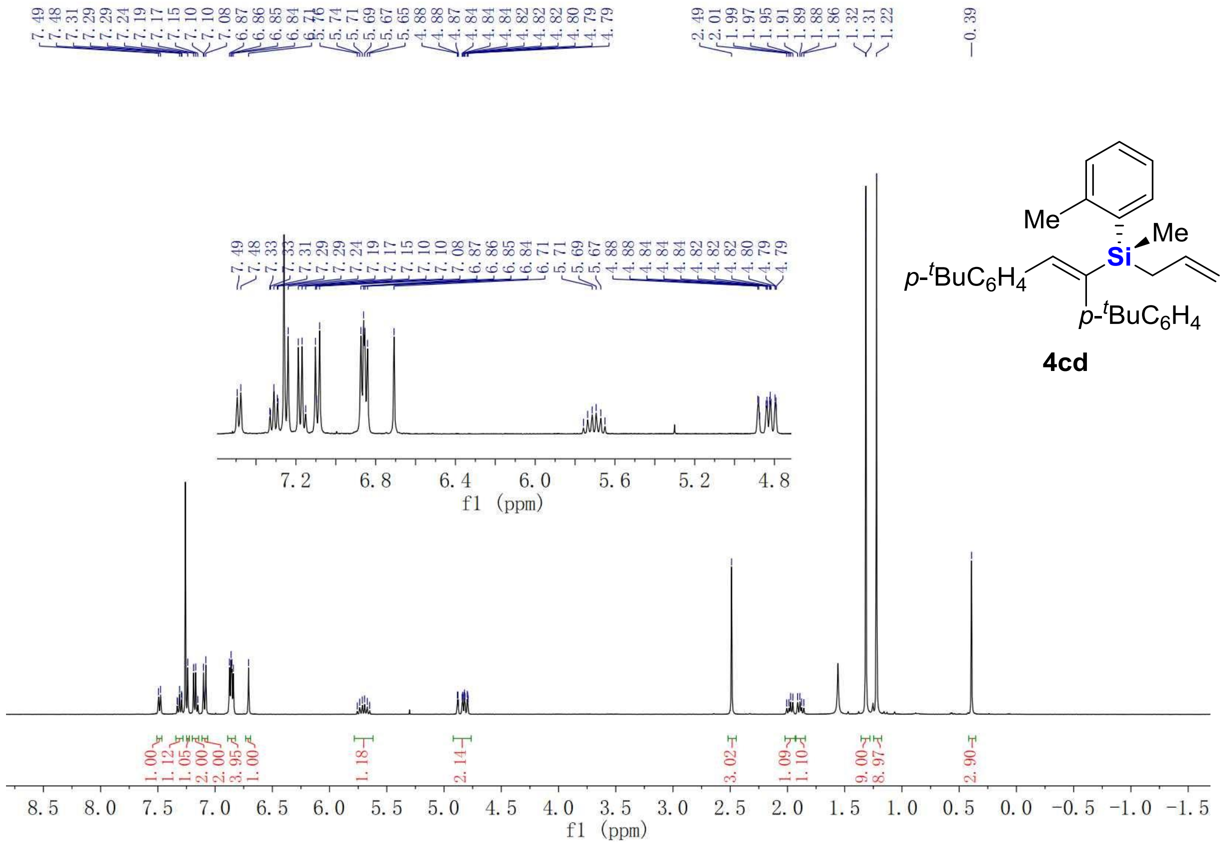

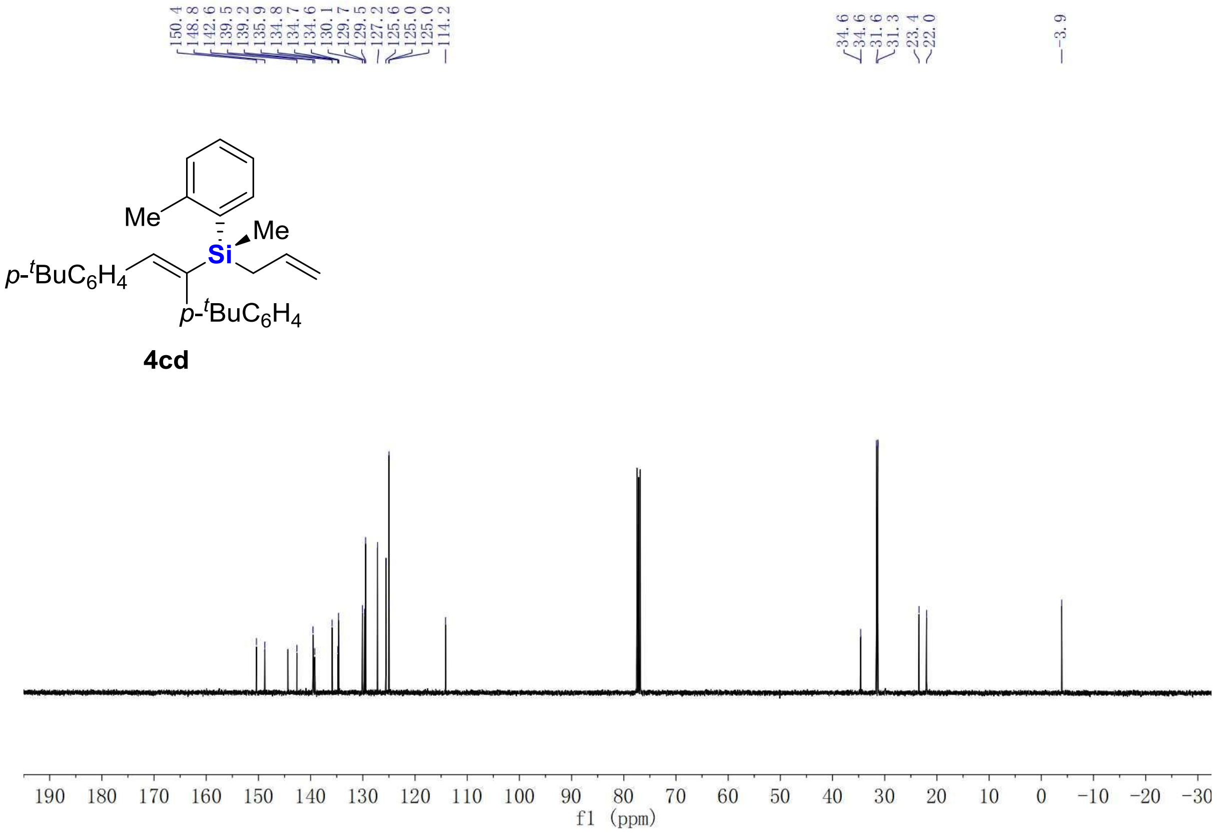

Supplementary Figure 50 ^{1}H and ^{13}C NMR Spectra for compound 4cd

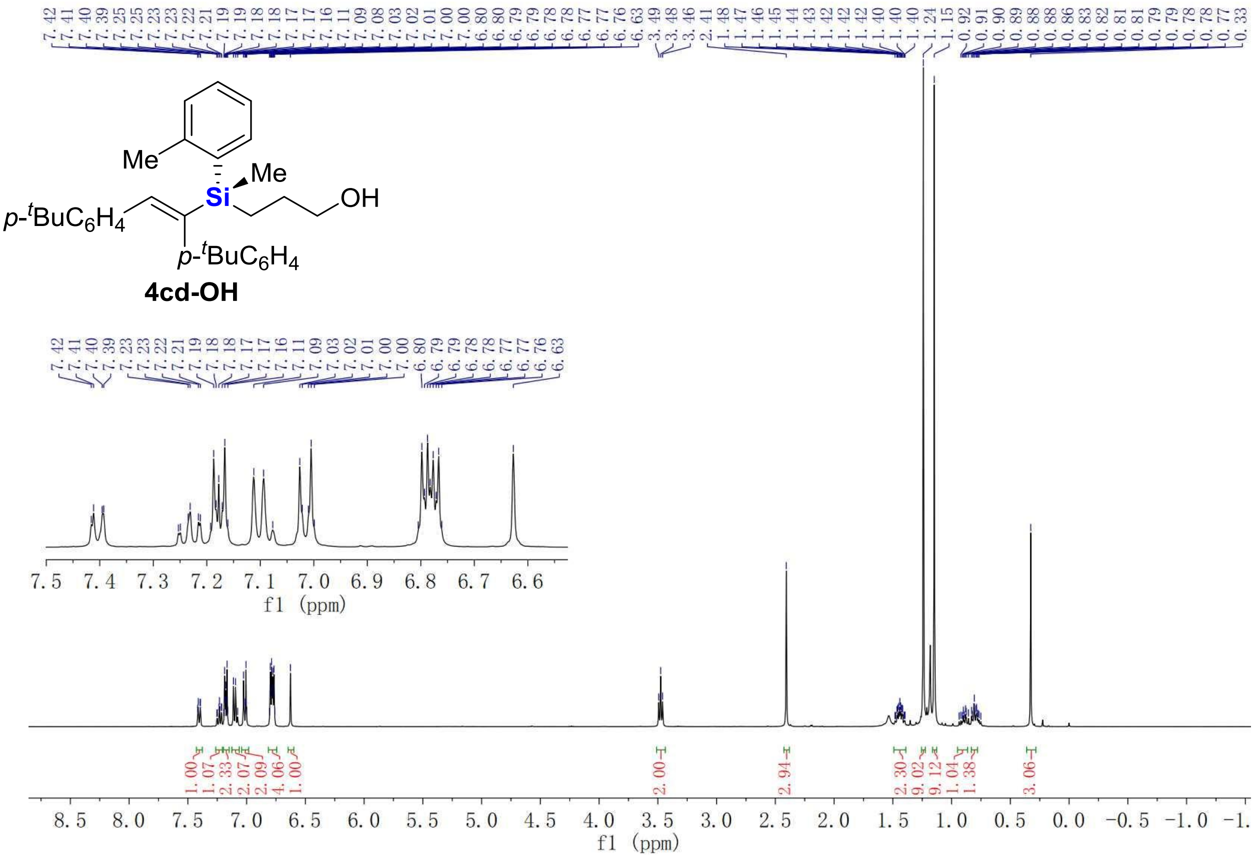

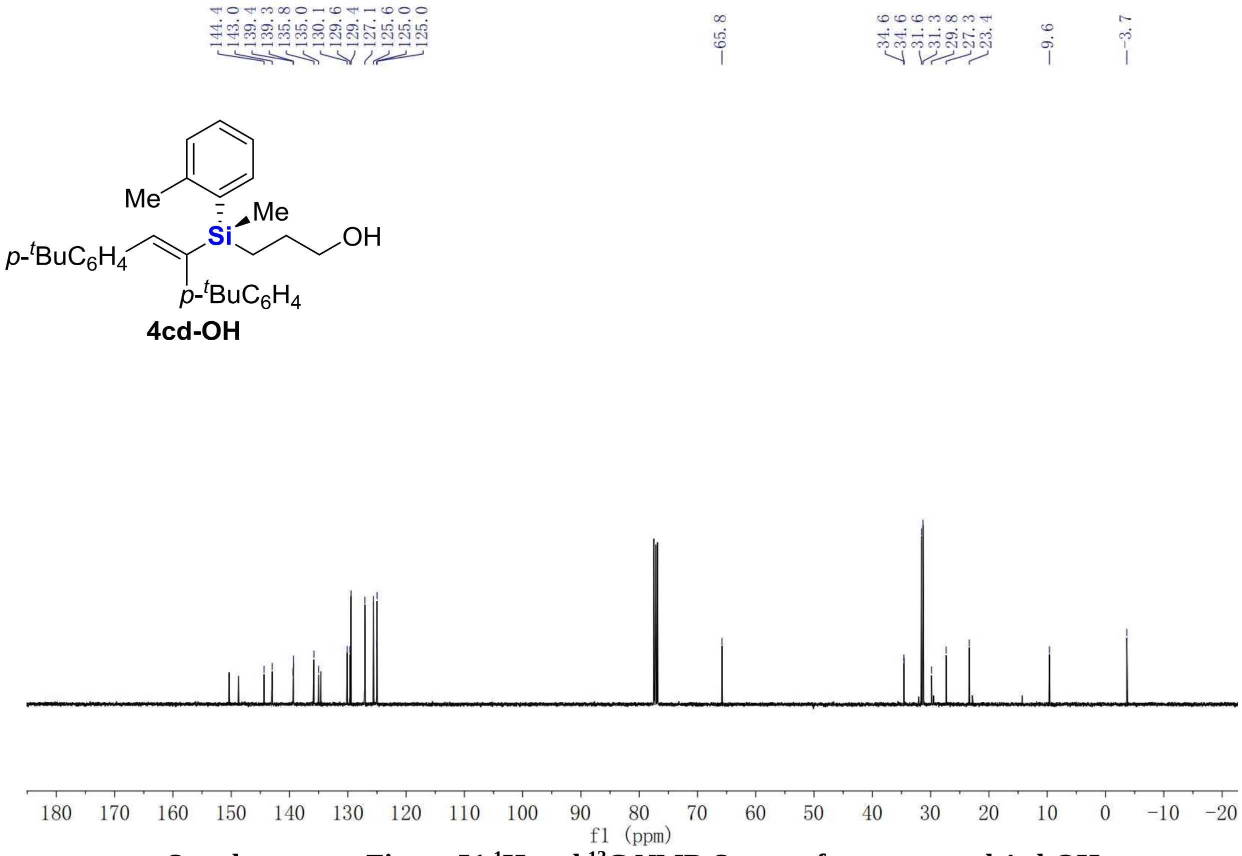

Supplementary Figure 51 ^{1}H and ^{13}C NMR Spectra for compound 4cd-OH

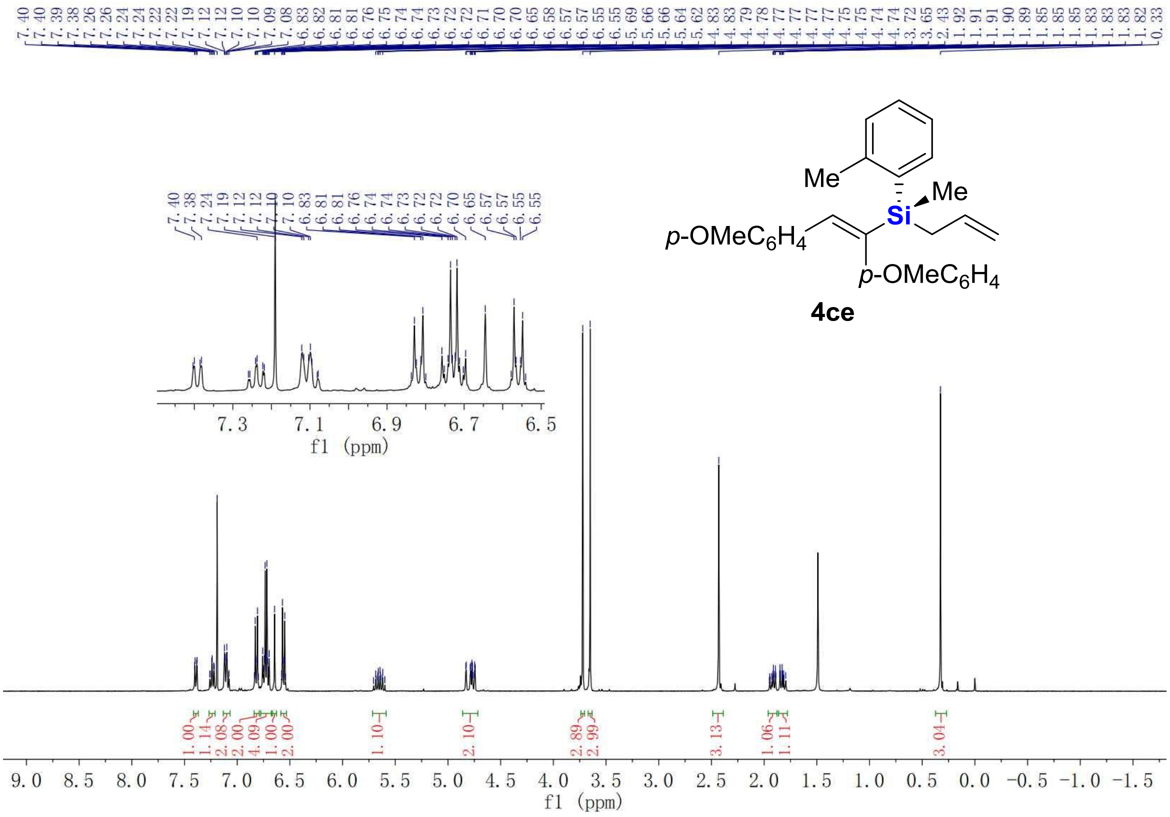

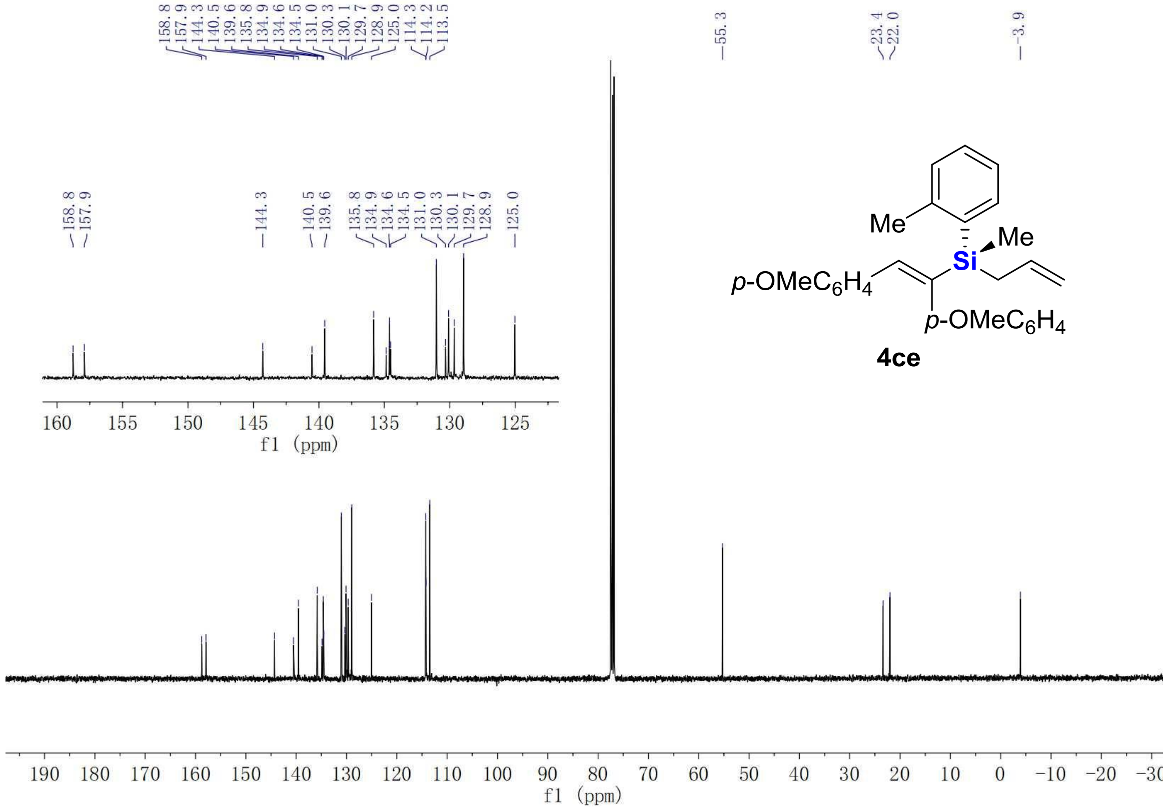

Supplementary Figure 52 ^{1}H and ^{13}C NMR Spectra for compound 4ce

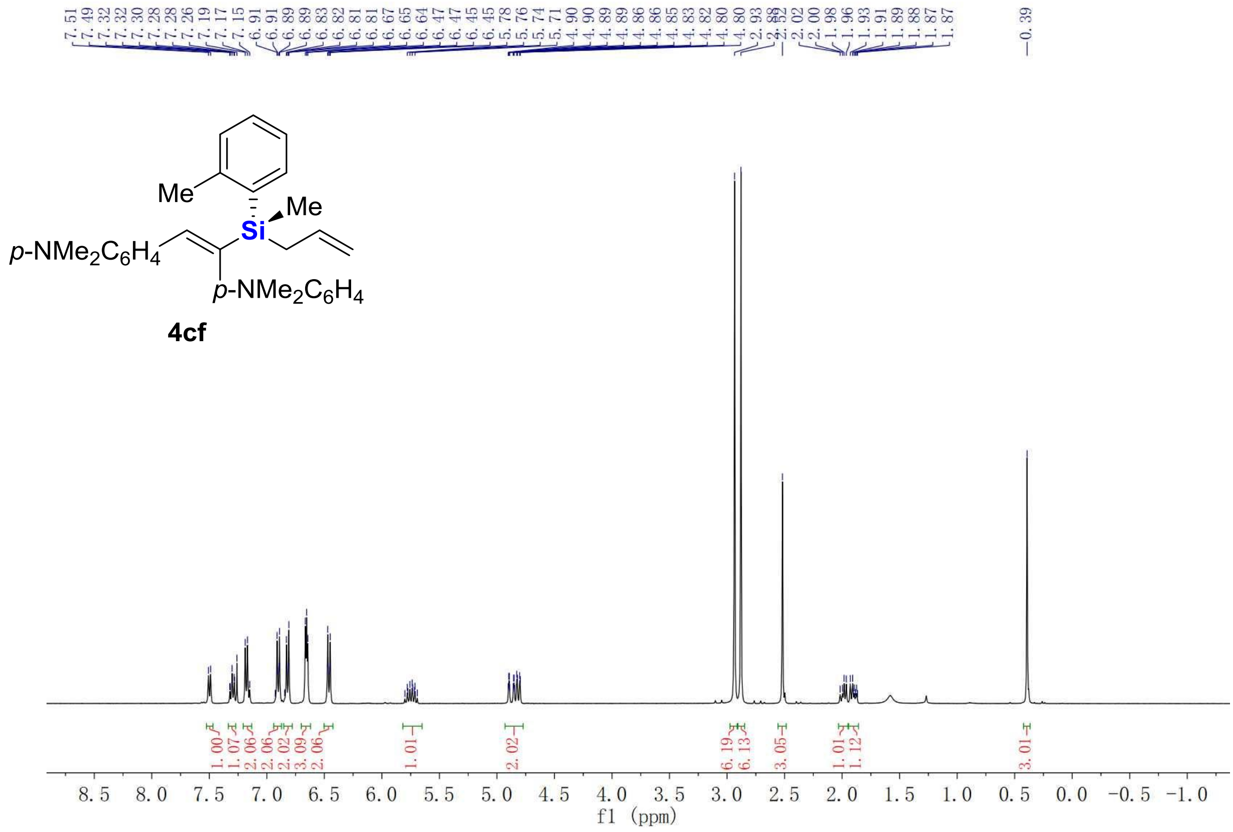

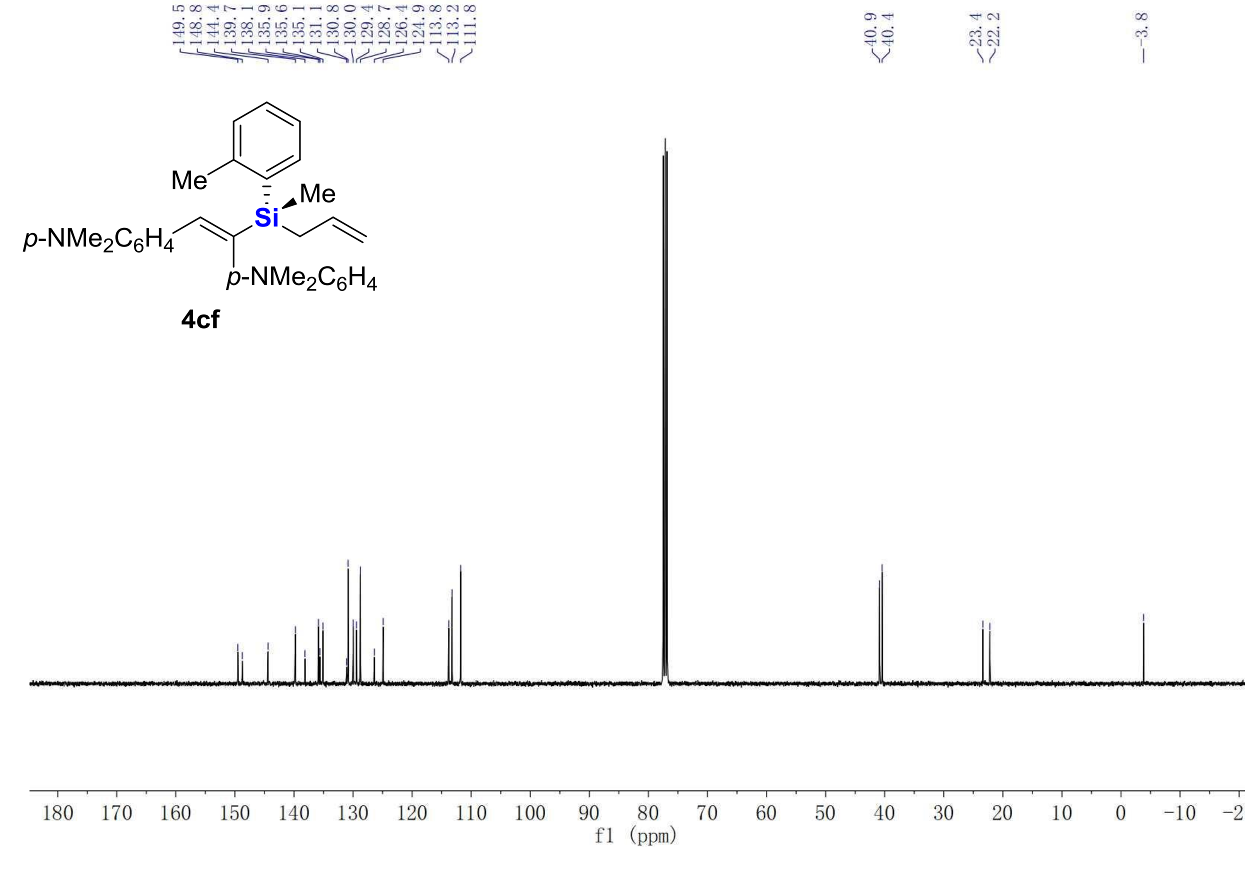

Supplementary Figure 53 ^{1}H and ^{13}C NMR Spectra for compound 4cf

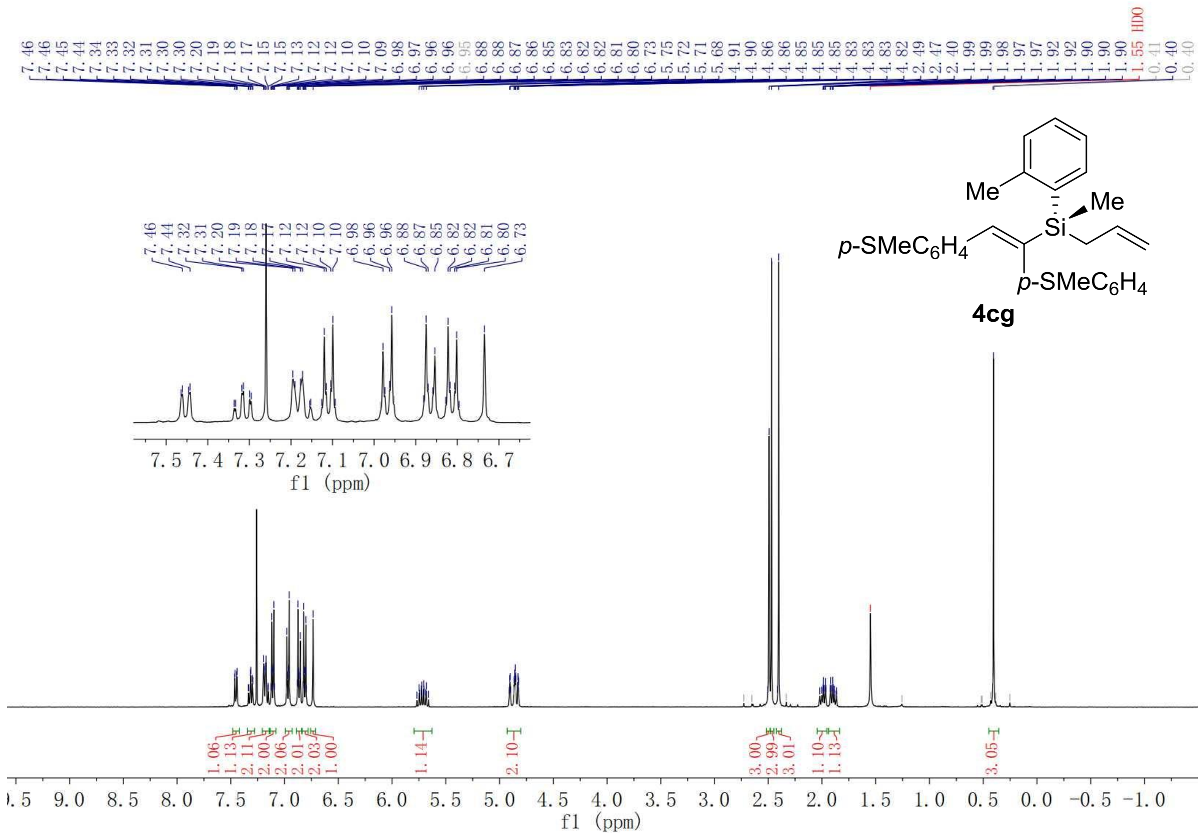

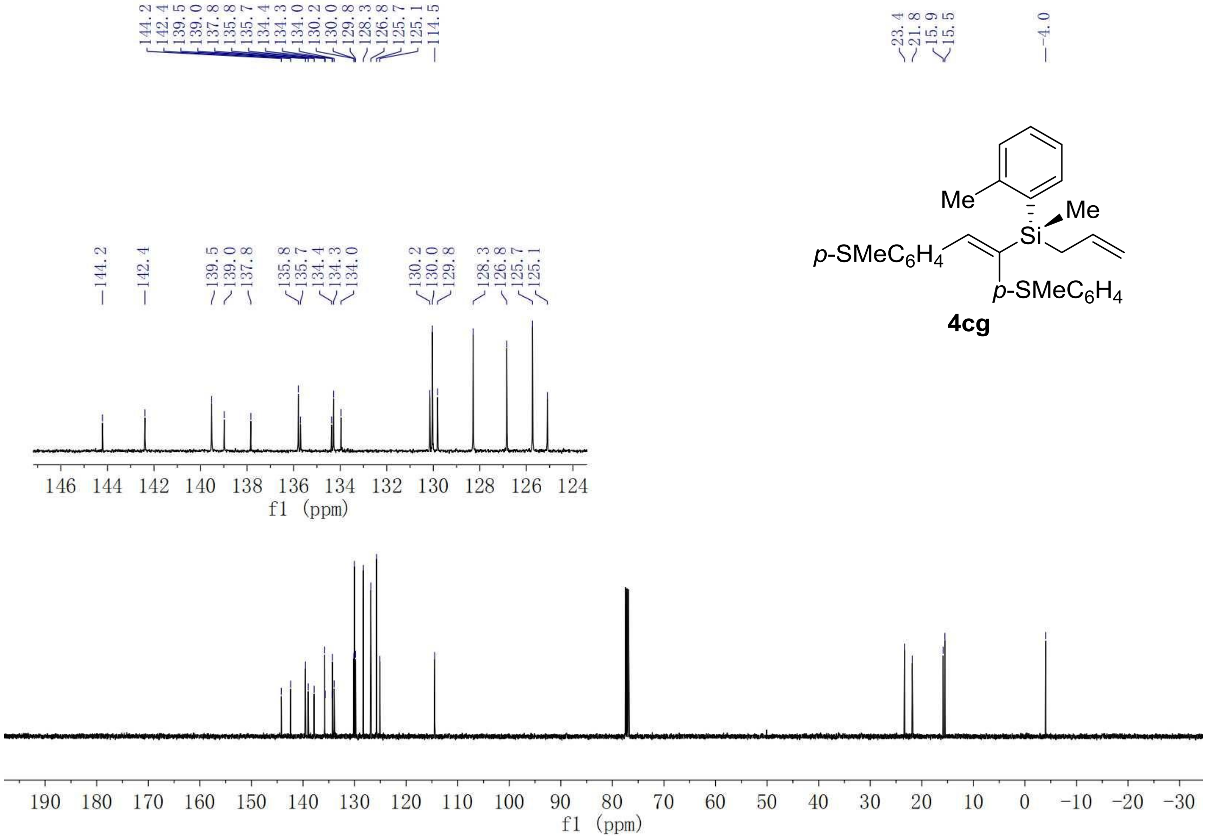

Supplementary Figure 54 ^{1}H and ^{13}C NMR Spectra for compound 4cg

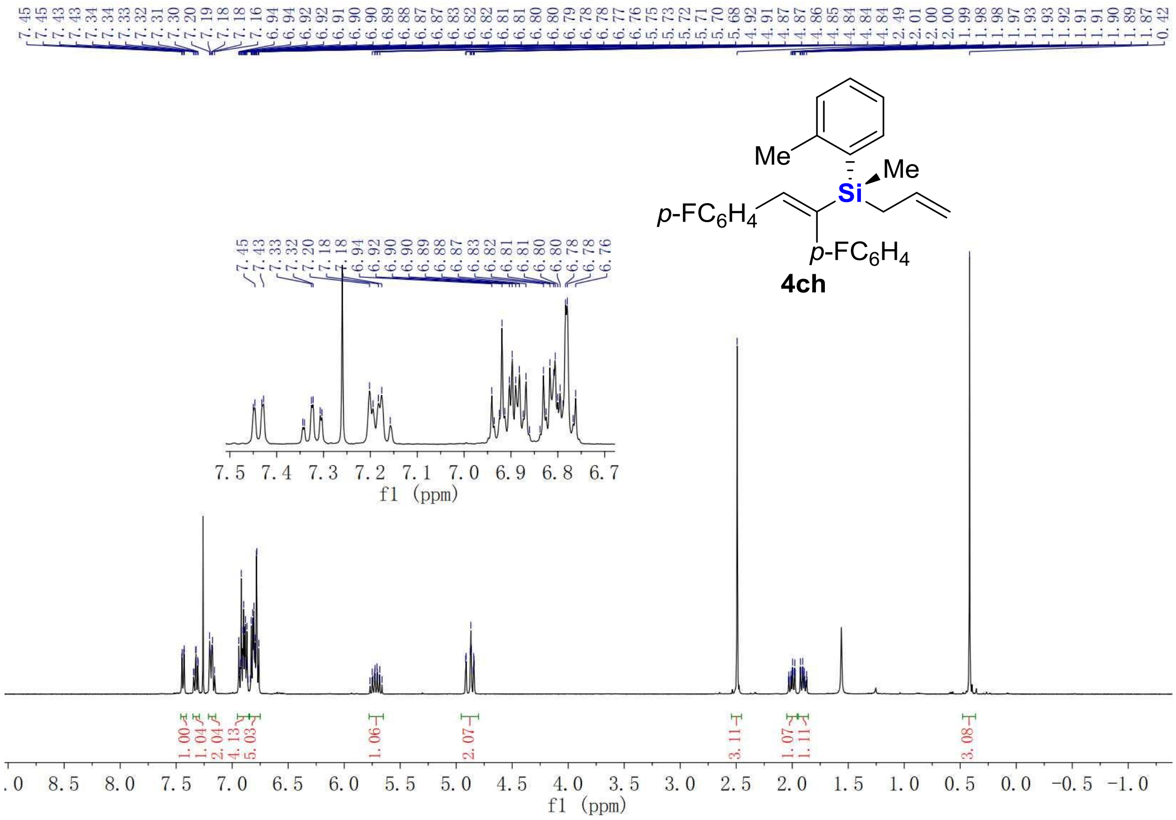
Me
Me
Si
p-FC6H4
p-FC6H4
4ch
f1 (ppm)

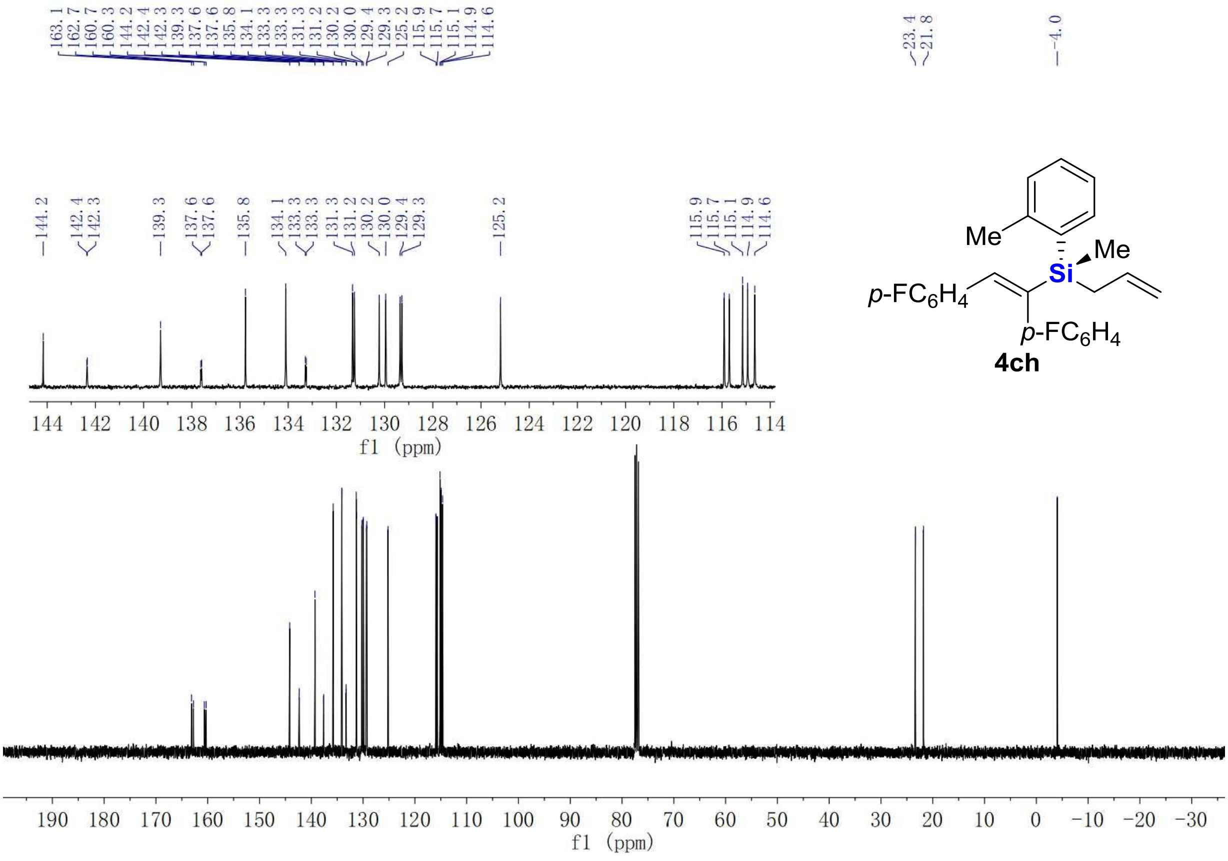
Me
Me
Si
p-FC6H4
p-FC6H4
4ch
f1 (ppm)

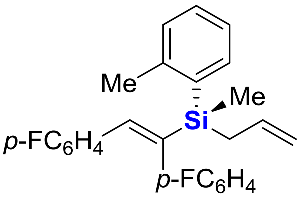

4ch

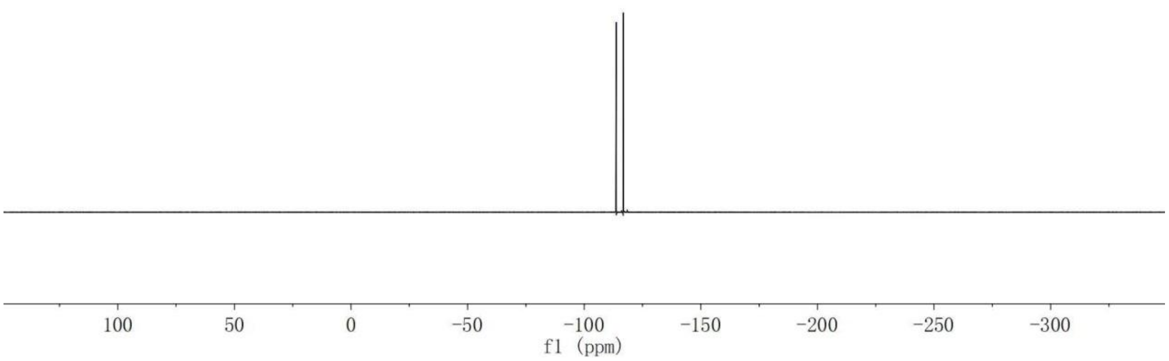

Supplementary Figure 55 ^{1}H, ^{13}C and ^{19}F NMR Spectra for compound 4ch

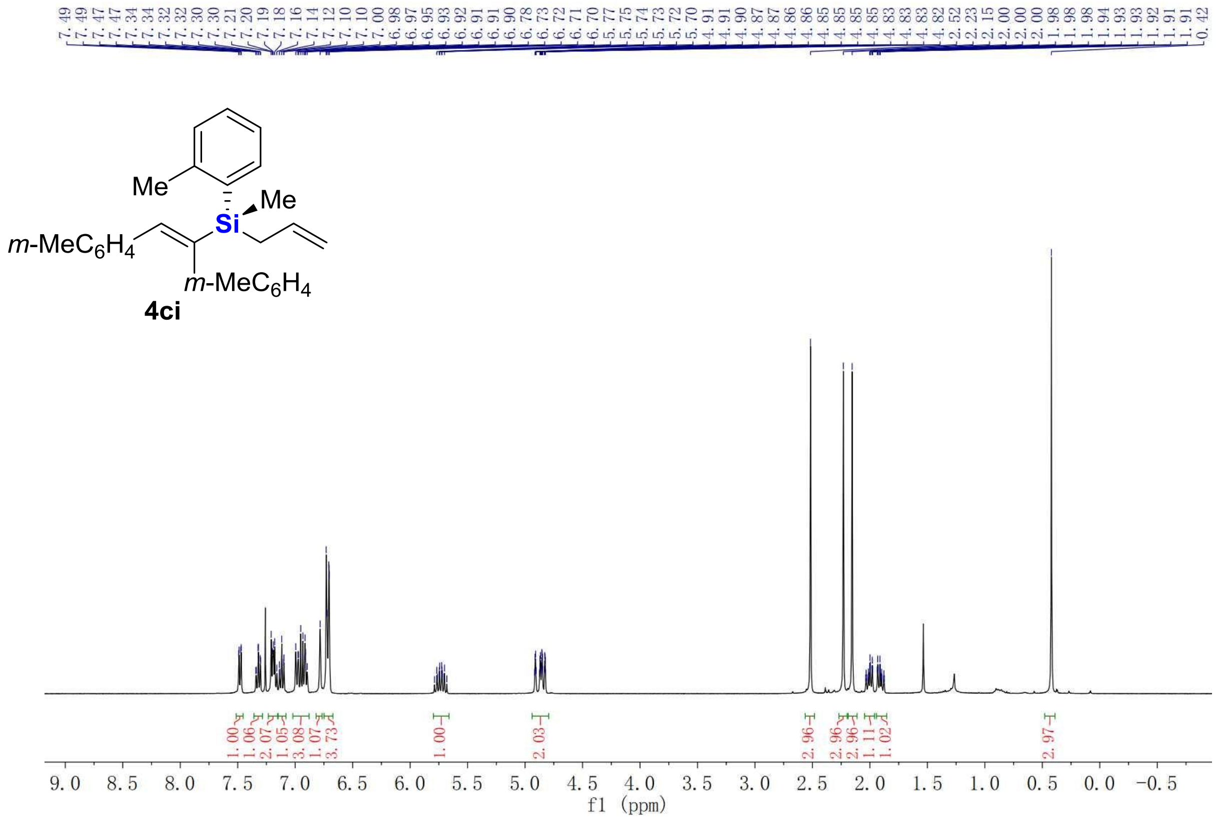

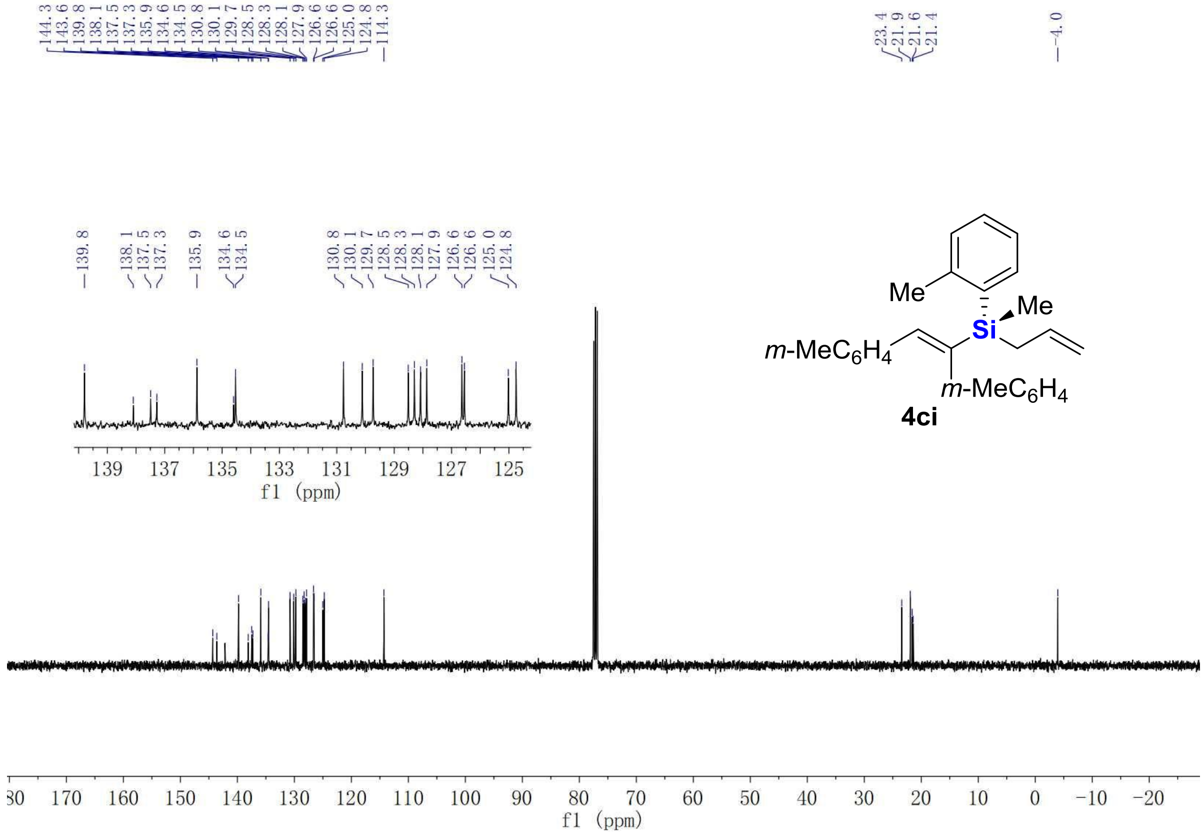

Supplementary Figure 56 ^{1}H and ^{13}C NMR Spectra for compound 4ci

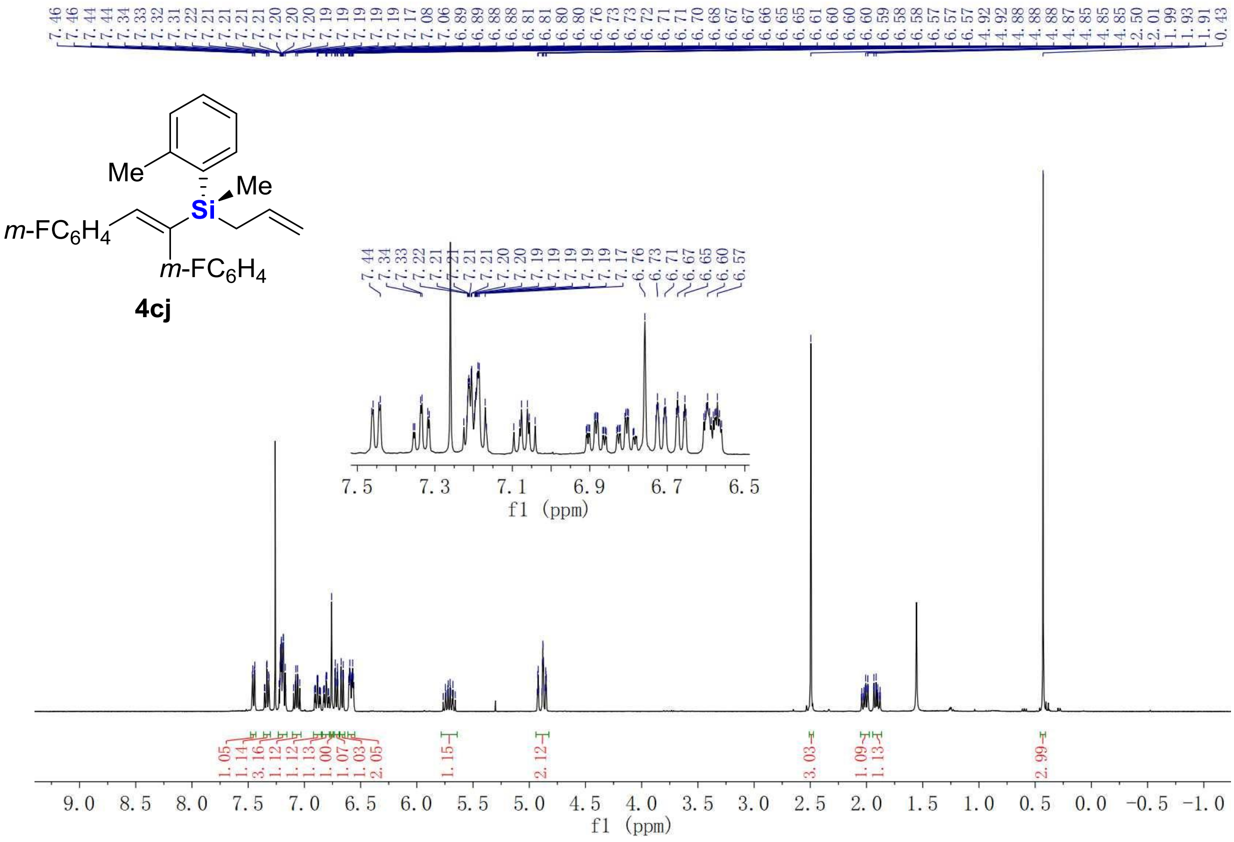
Me
Me
Si
m-FC6H4
m-FC6H4
4cj
1.05
1.14
3.16
1.12
1.12
1.13
1.00
1.07
1.03
2.05
1.15
2.12
3.03
1.09
1.13
2.99
f1 (ppm)

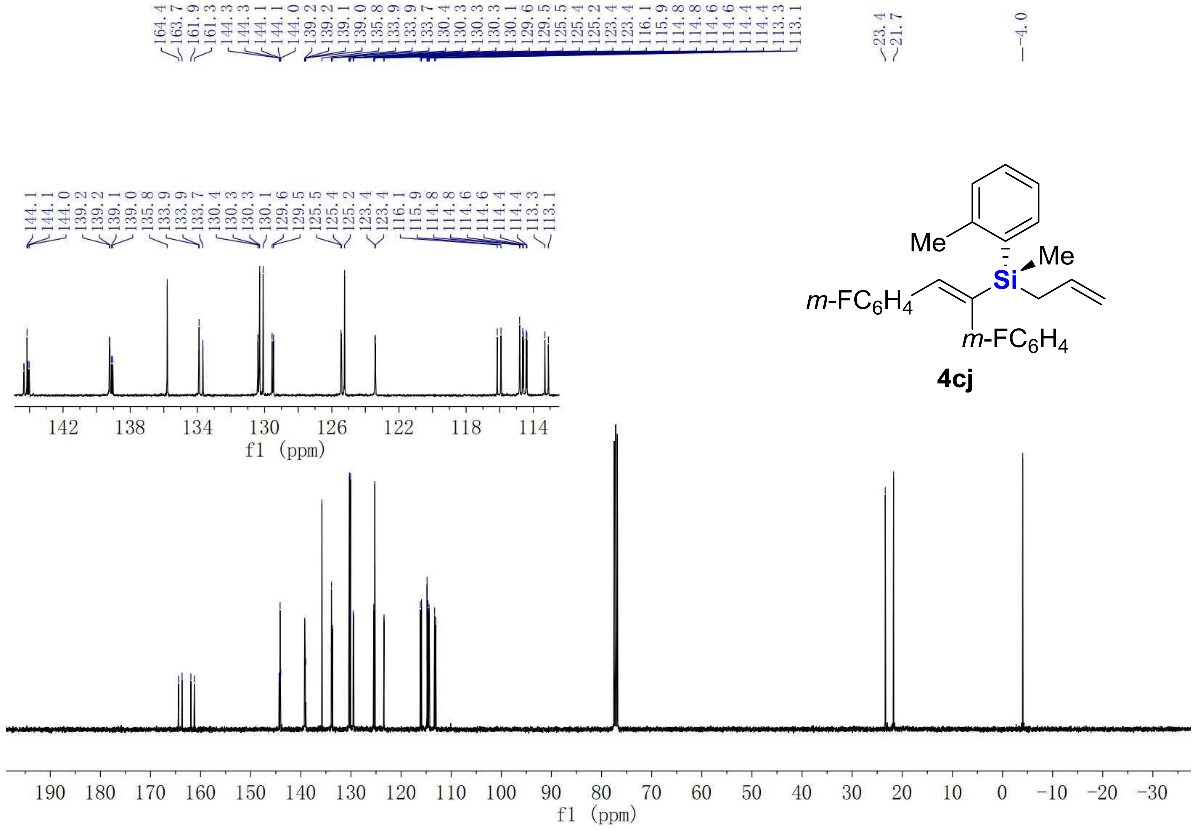
Me
Me
Si
m-FC6H4
m-FC6H4
4cj
f1 (ppm)

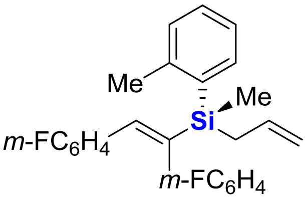

4cj

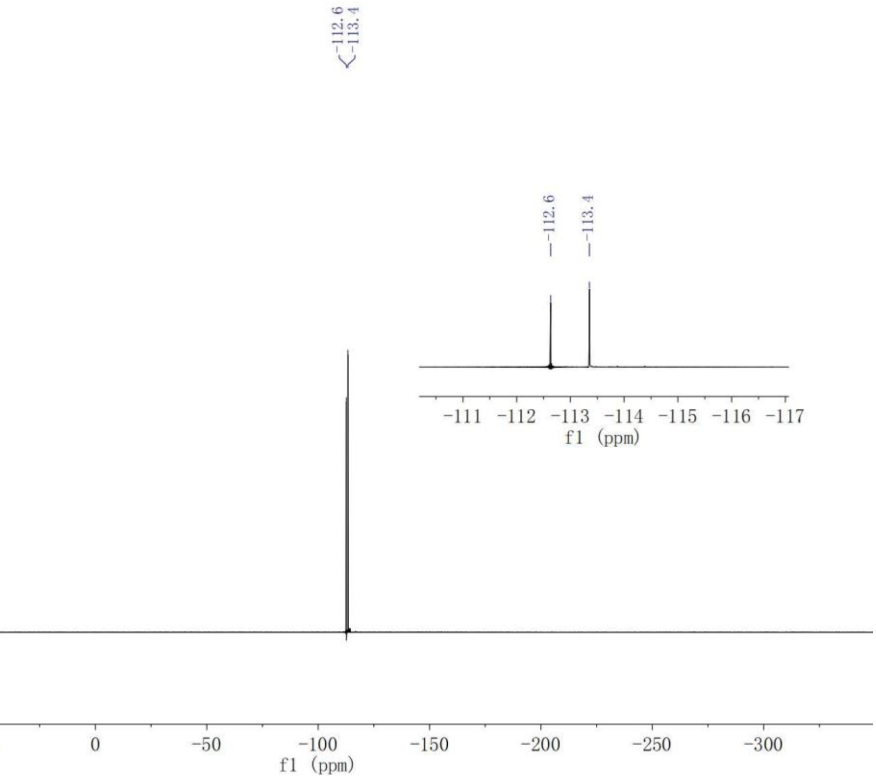

Supplementary Figure 57 ^{1}H, ^{13}C and ^{19}F NMR Spectra for compound 4cj

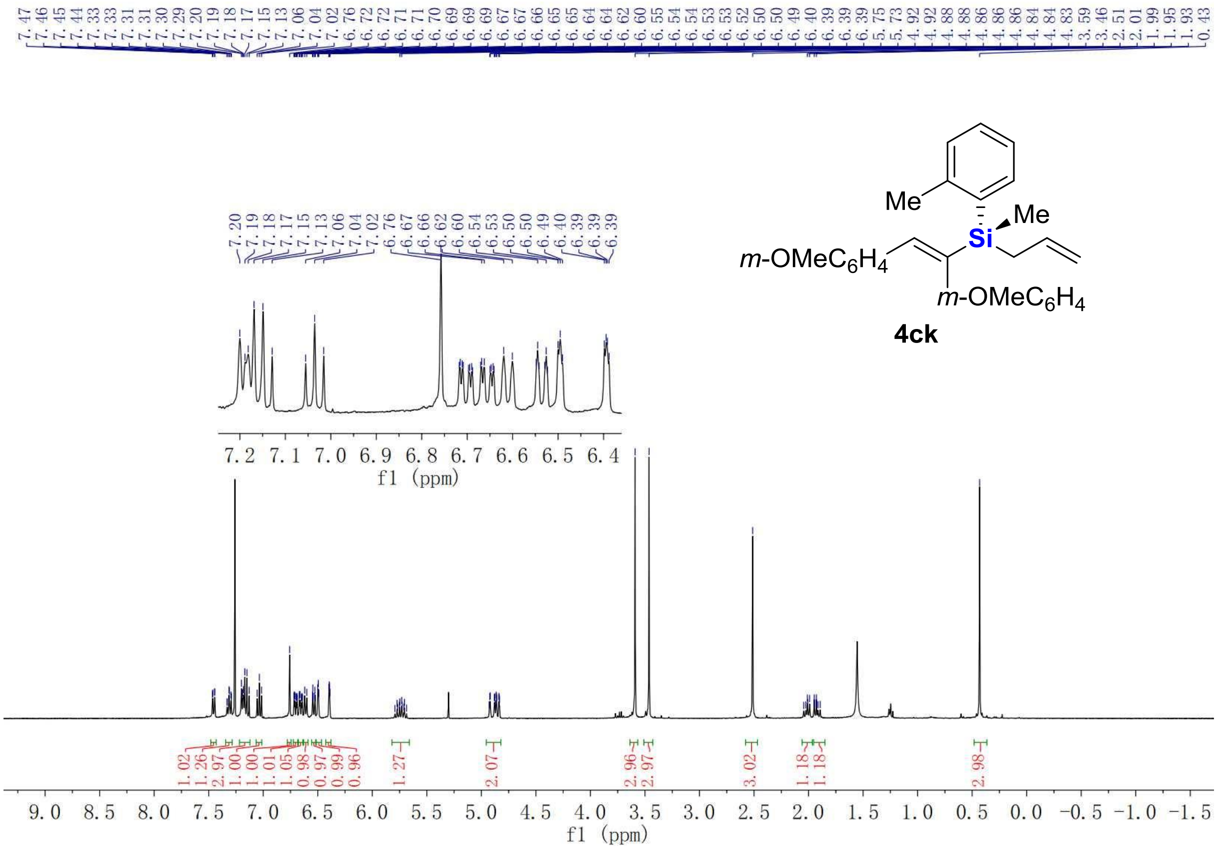

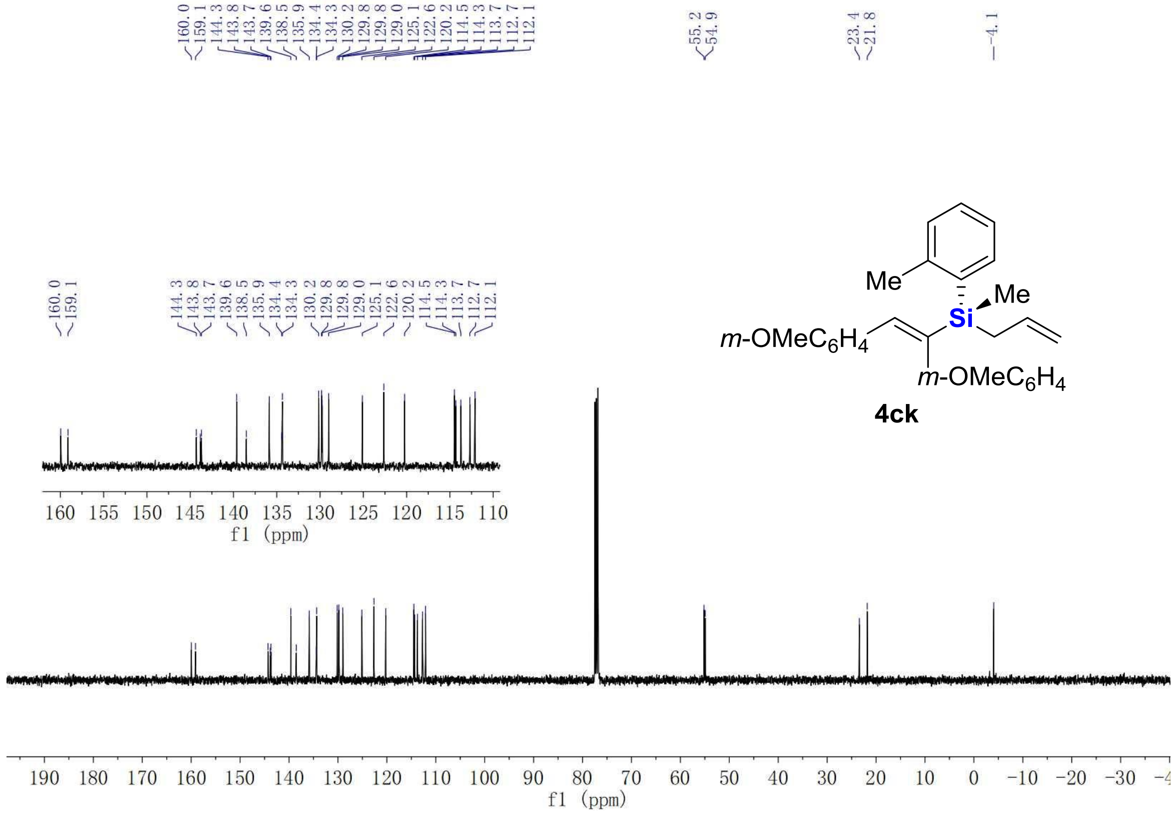

Supplementary Figure 58 ^{1}H and ^{13}C NMR Spectra for compound 4ck

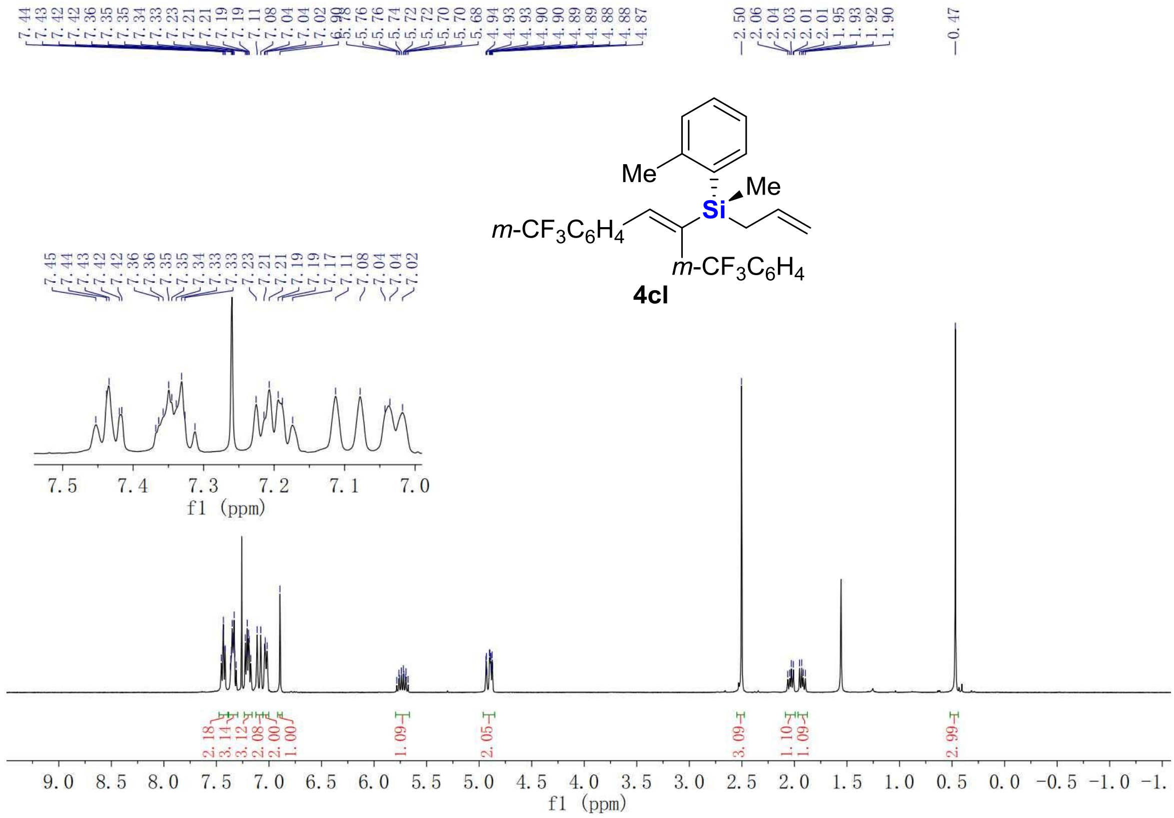

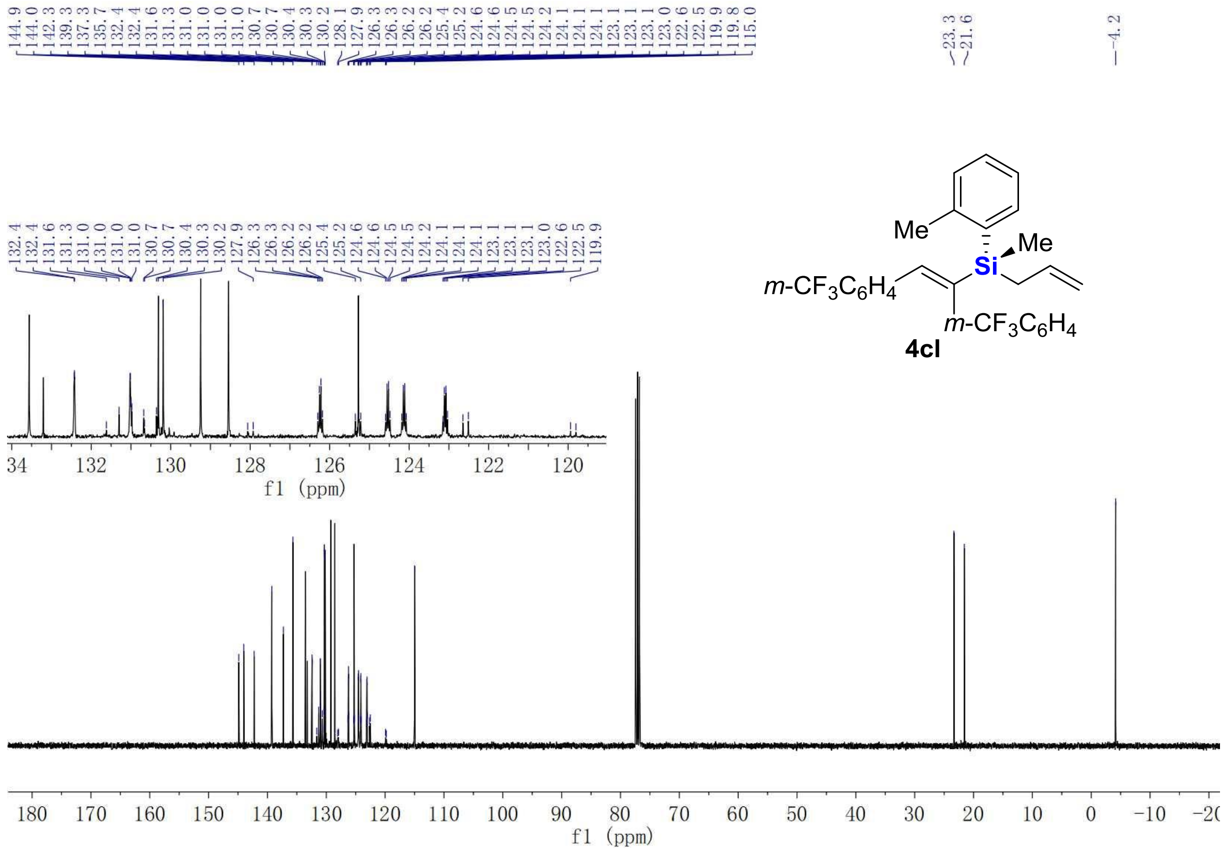

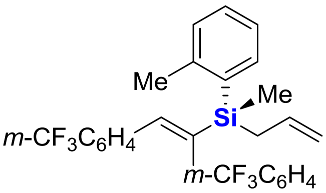

4cl

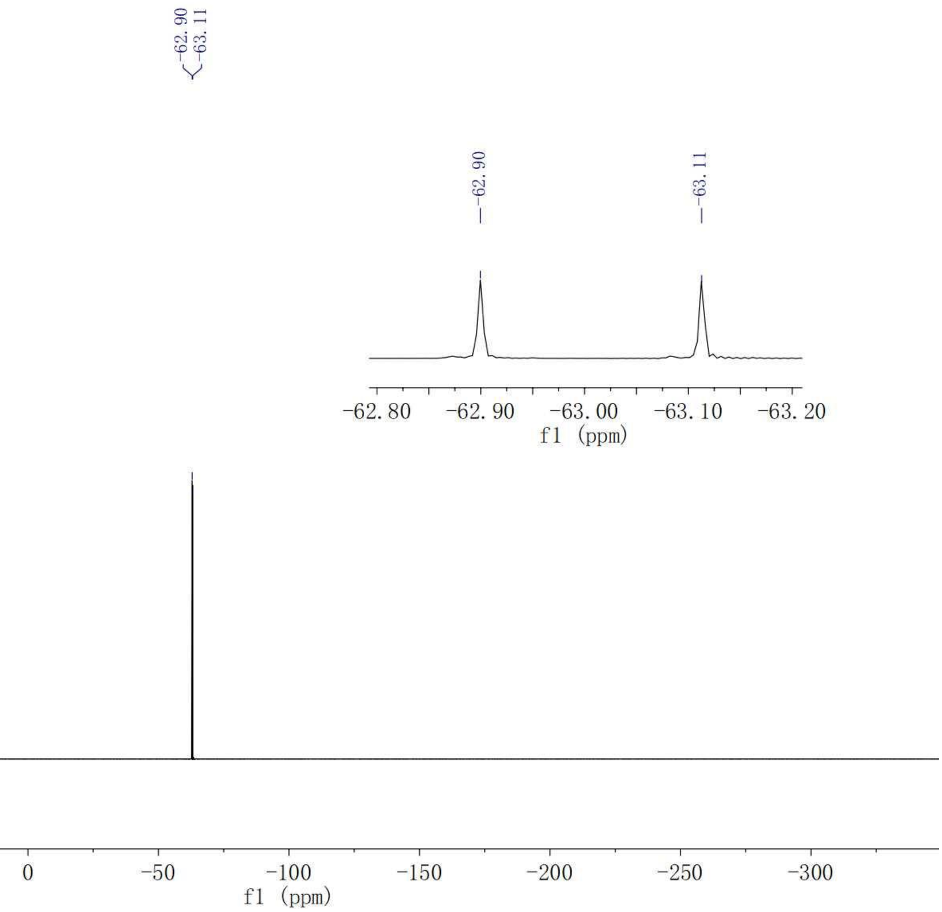

Supplementary Figure 59 ^{1}H, ^{13}C and ^{19}F NMR Spectra for compound 4cl

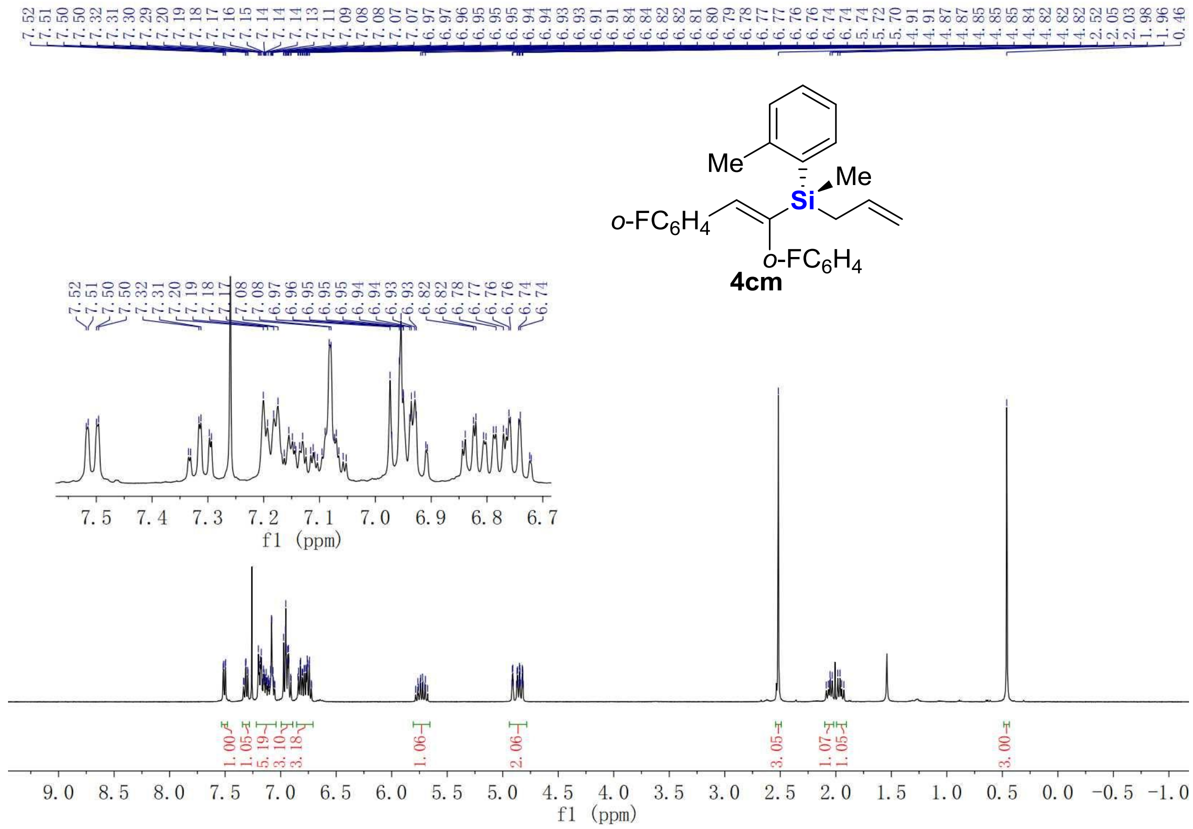
7.52 7.51 7.50 7.50 7.32 7.31 7.30 7.29 7.20 7.19 7.18 7.17 7.16 7.15 7.14 7.14 7.14 7.13 7.11 7.09 7.08 7.08 7.07 7.07 6.97 6.97 6.96 6.95 6.95 6.95 6.94 6.94 6.93 6.93 6.91 6.91 6.84 6.82 6.82 6.81 6.80 6.79 6.78 6.77 6.77 6.76 6.76 6.74 6.74 5.74 5.74 5.72 5.70 4.91 4.91 4.87 4.87 4.85 4.85 4.85 4.84 4.84 4.82 4.82 4.82 2.52 2.05 2.03 1.98 1.96 0.46
Me
Me
Si
o-FC6H4
o-FC6H4
4cm
1.00
1.05
5.19
3.10
3.18
1.06
2.06
3.05
1.07
1.05
3.00
f1 (ppm)

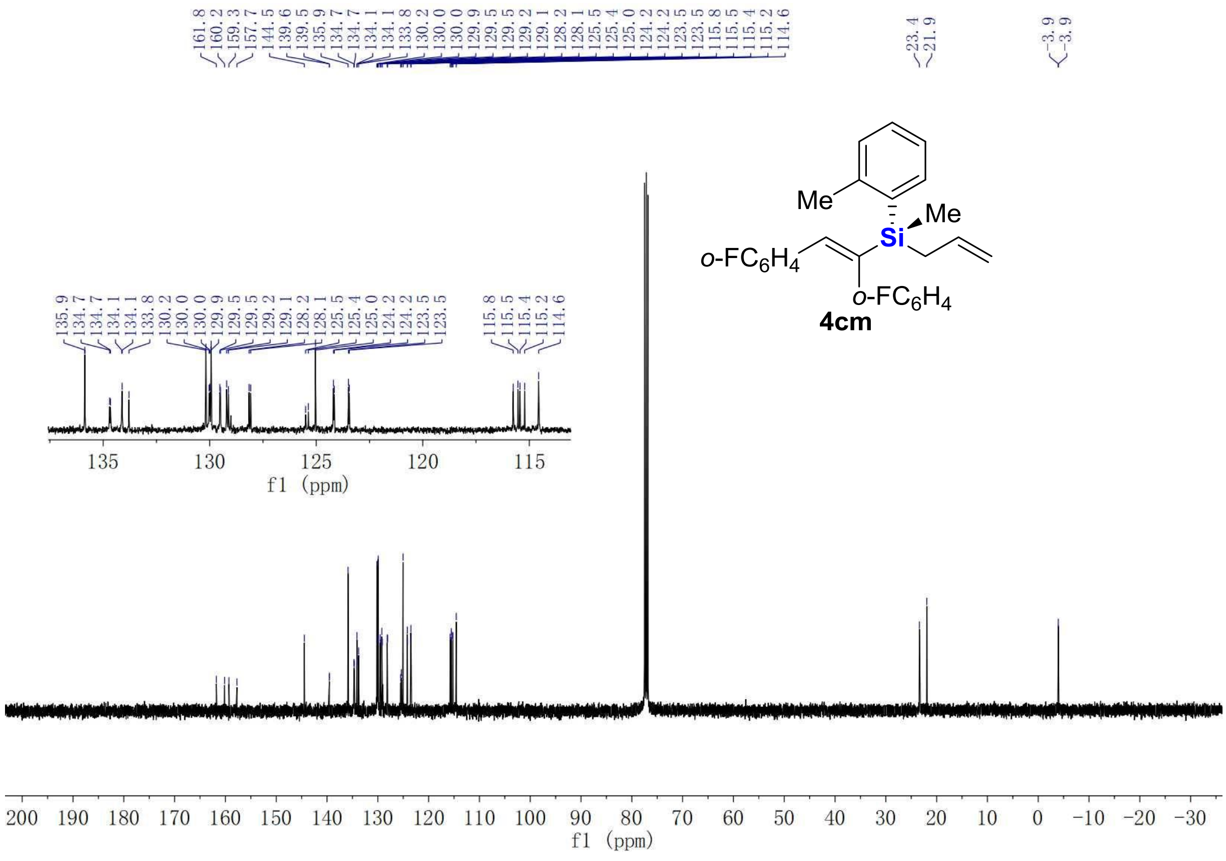
161.8 160.2 159.3 157.7 144.5 139.6 139.5 135.9 134.7 134.7 134.1 134.1 133.8 130.2 130.0 130.0 129.9 129.5 129.5 129.2 129.1 128.2 128.1 125.5 125.4 125.0 124.2 124.2 123.5 123.5 115.8 115.5 115.4 115.2 114.6
23.4 21.9
-3.9 -3.9
Me
Me
Si
o-FC6H4
o-FC6H4
4cm
f1 (ppm)

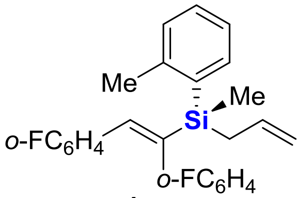

4cm

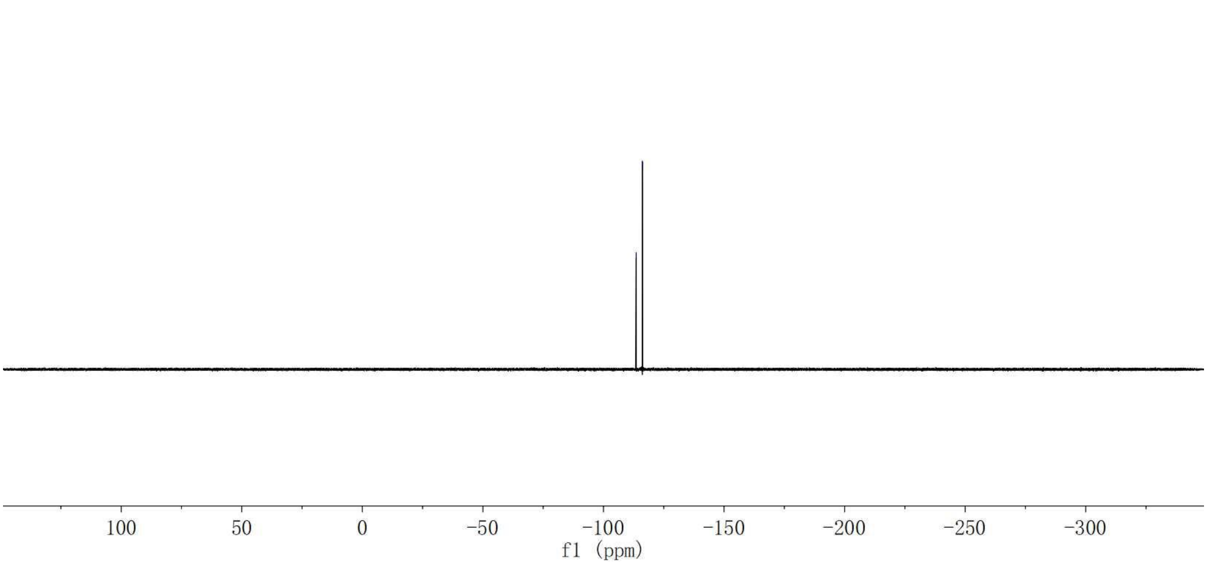

Supplementary Figure 60 ^{1}H, ^{13}C and ^{19}F NMR Spectra for compound 4cm

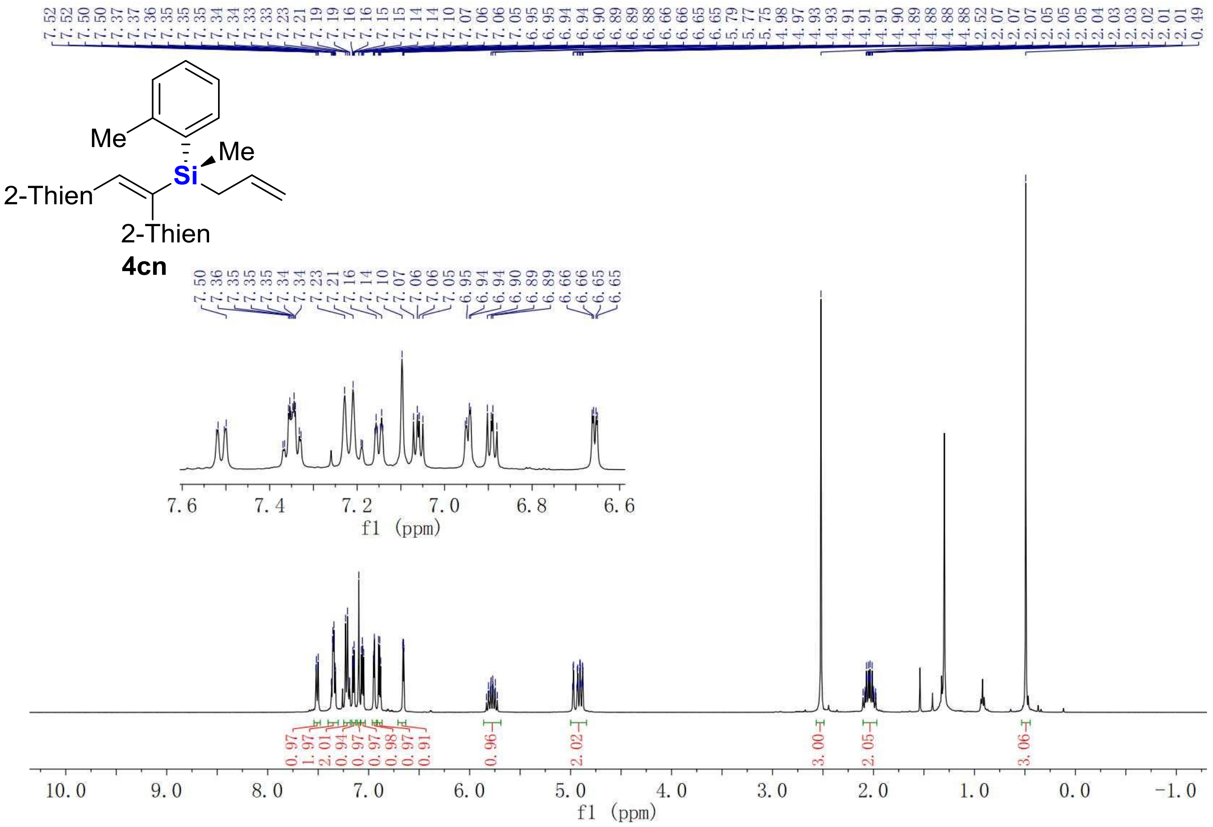

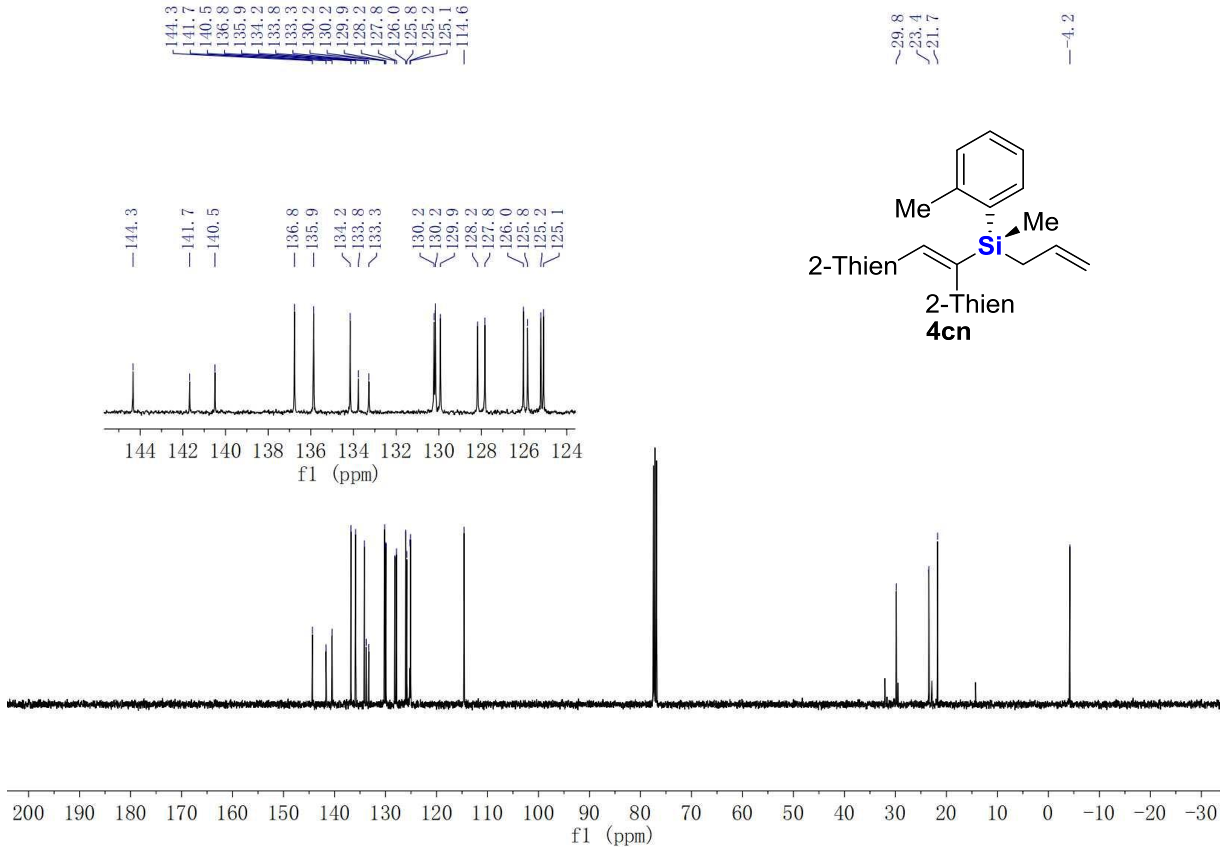

Supplementary Figure 61 ^{1}H and ^{13}C NMR Spectra for compound 4cn

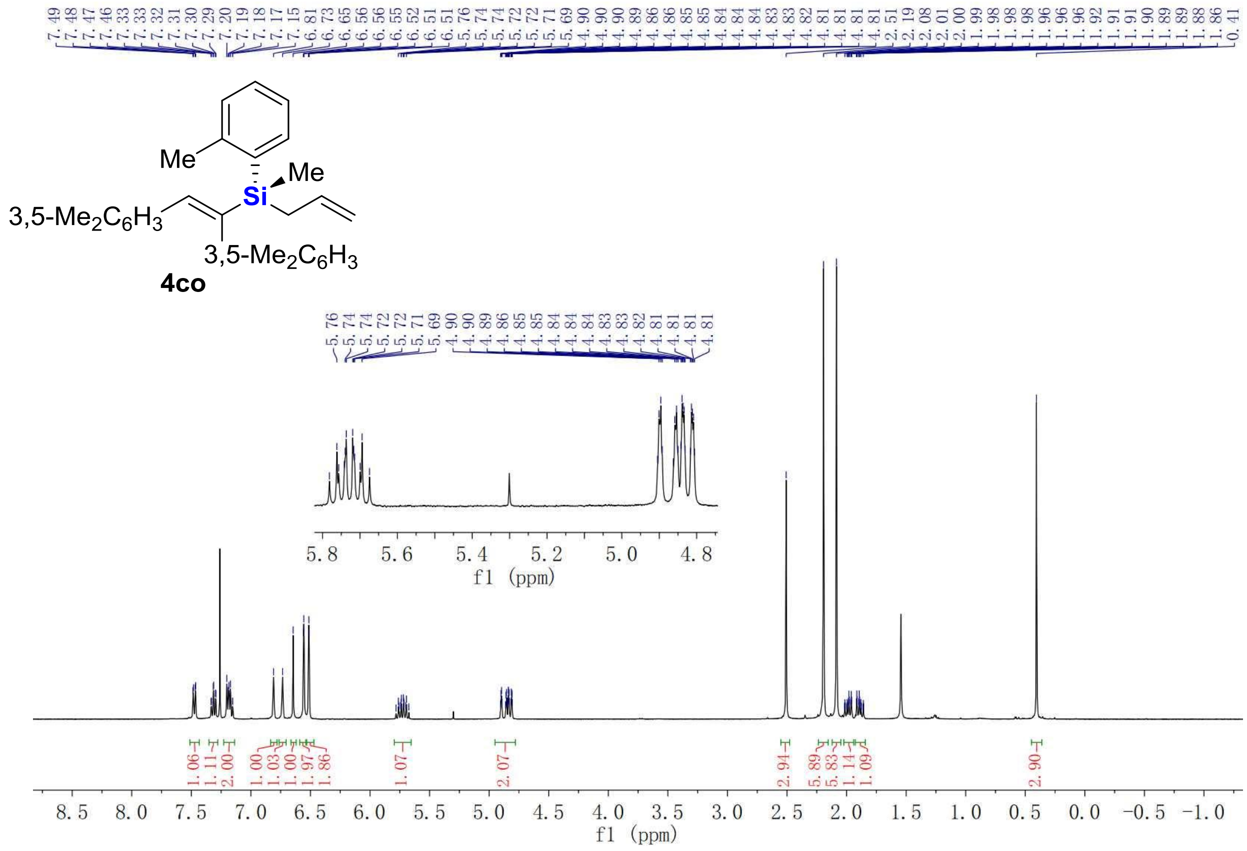

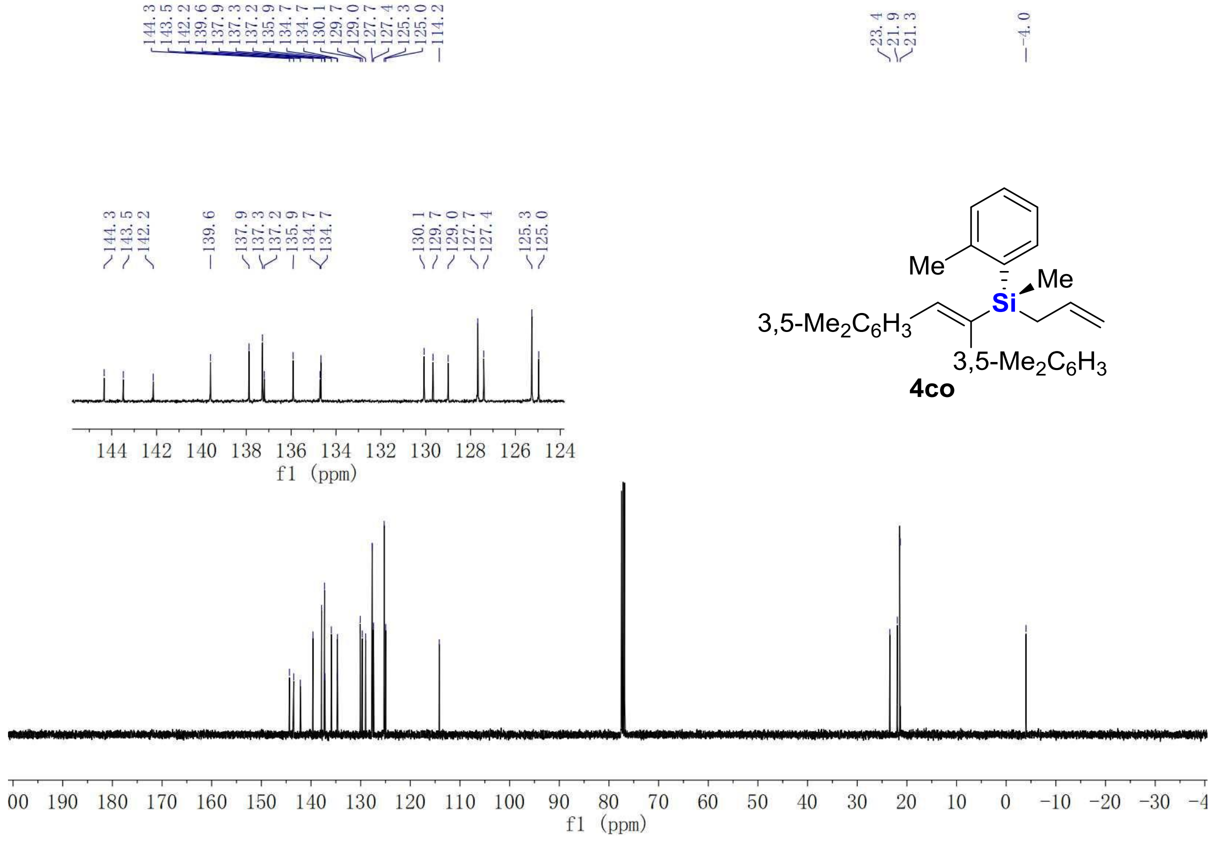

Supplementary Figure 62 ^{1}H and ^{13}C NMR Spectra for compound 4co

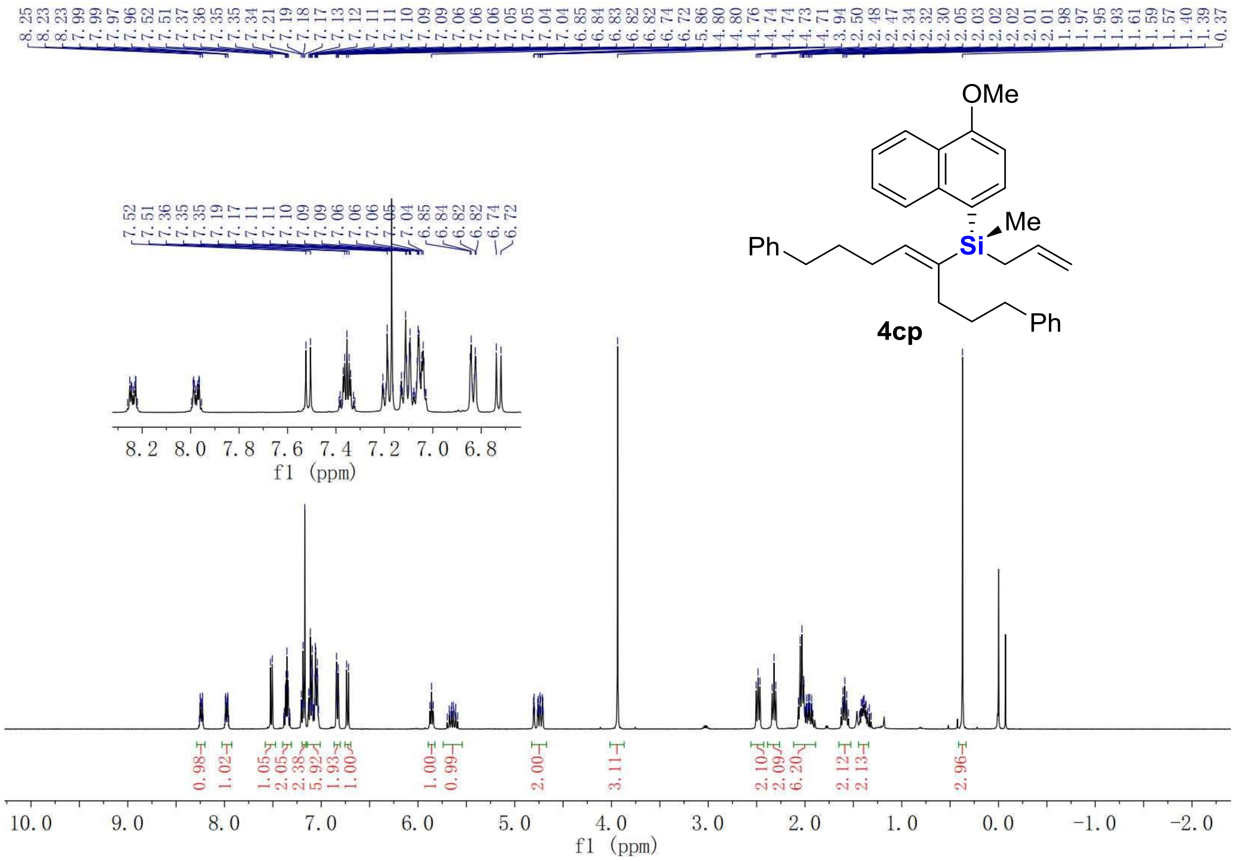

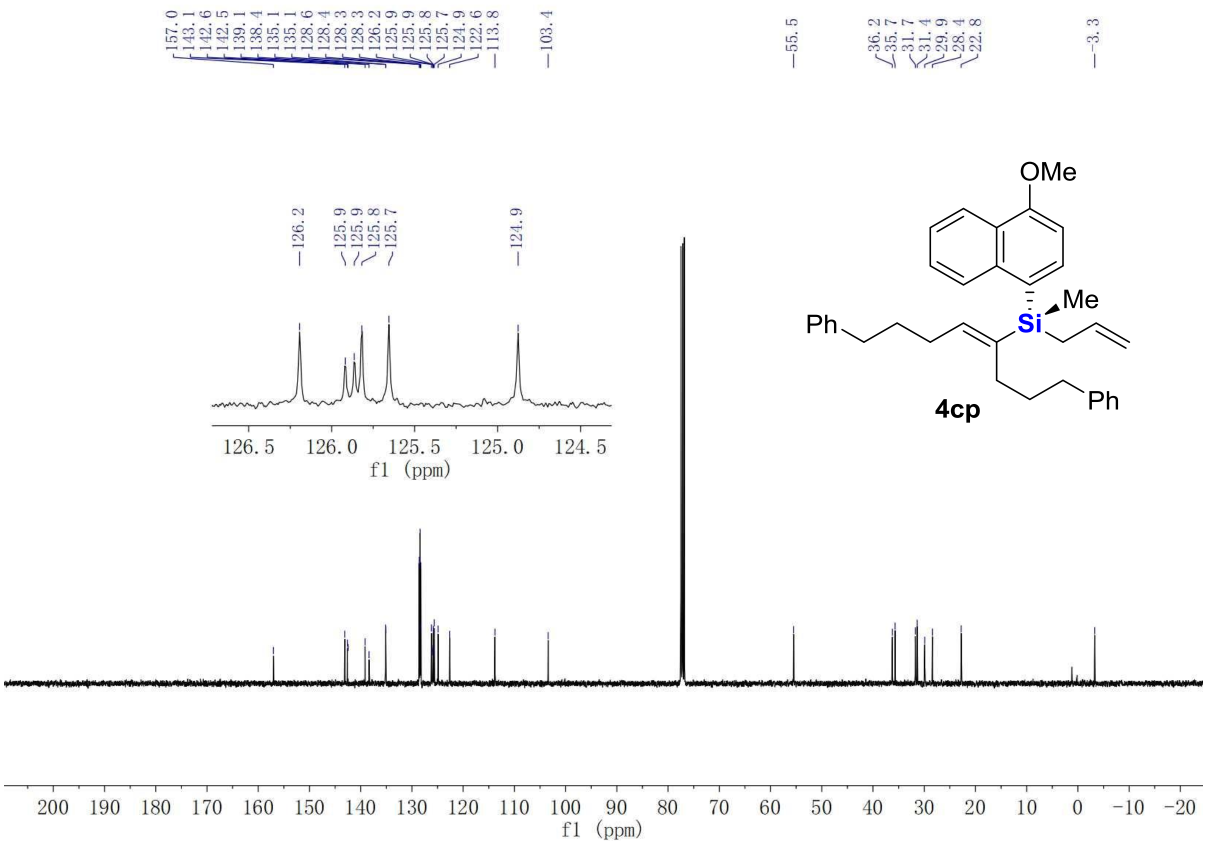

Supplementary Figure 63 ^{1}H and ^{13}C NMR Spectra for compound 4cp

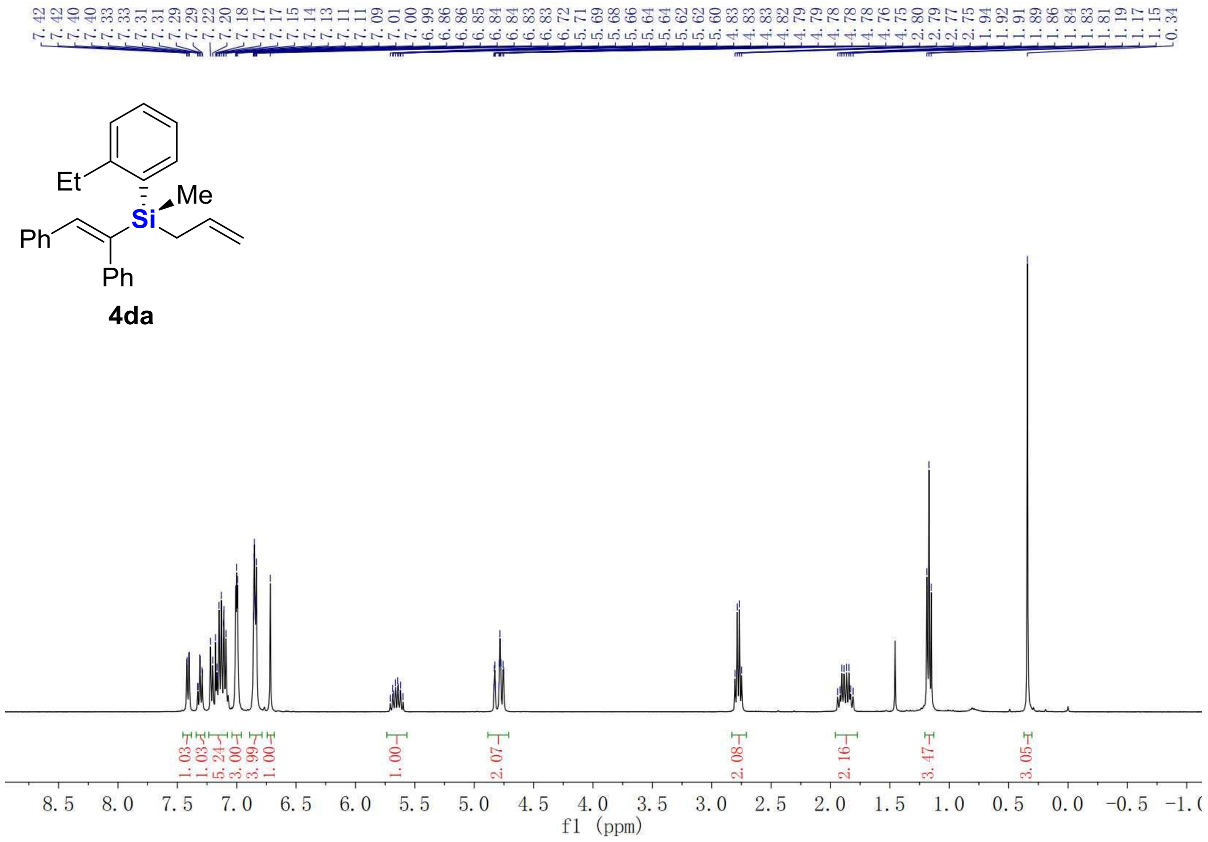

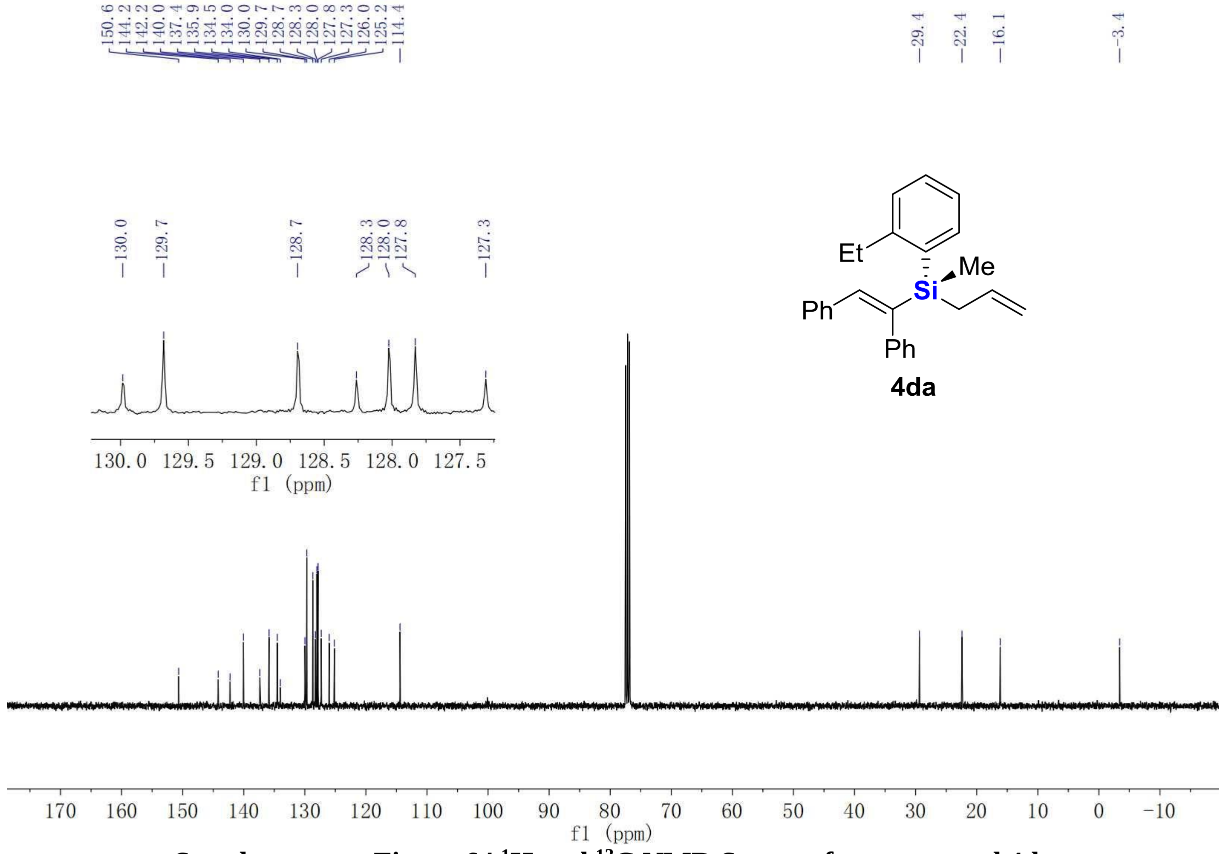

Supplementary Figure 64 ^{1}H and ^{13}C NMR Spectra for compound 4da

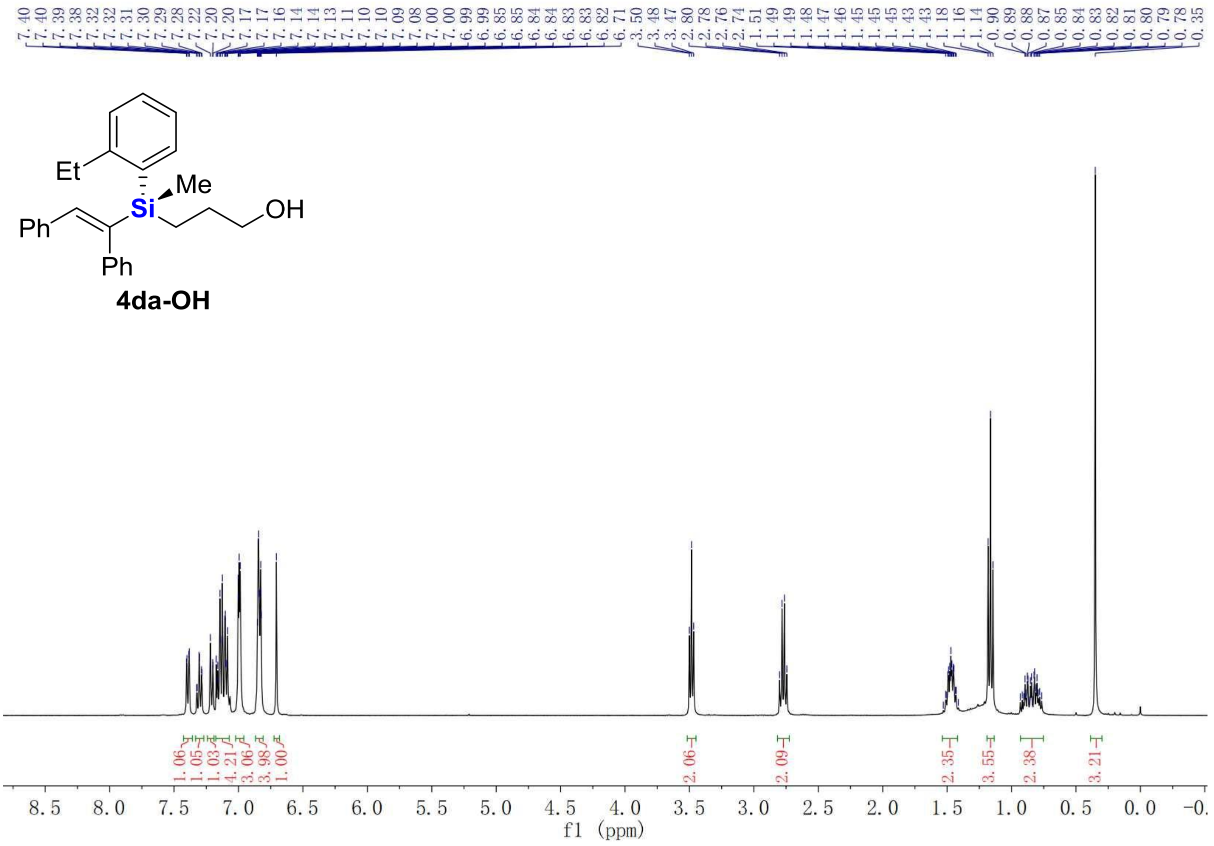

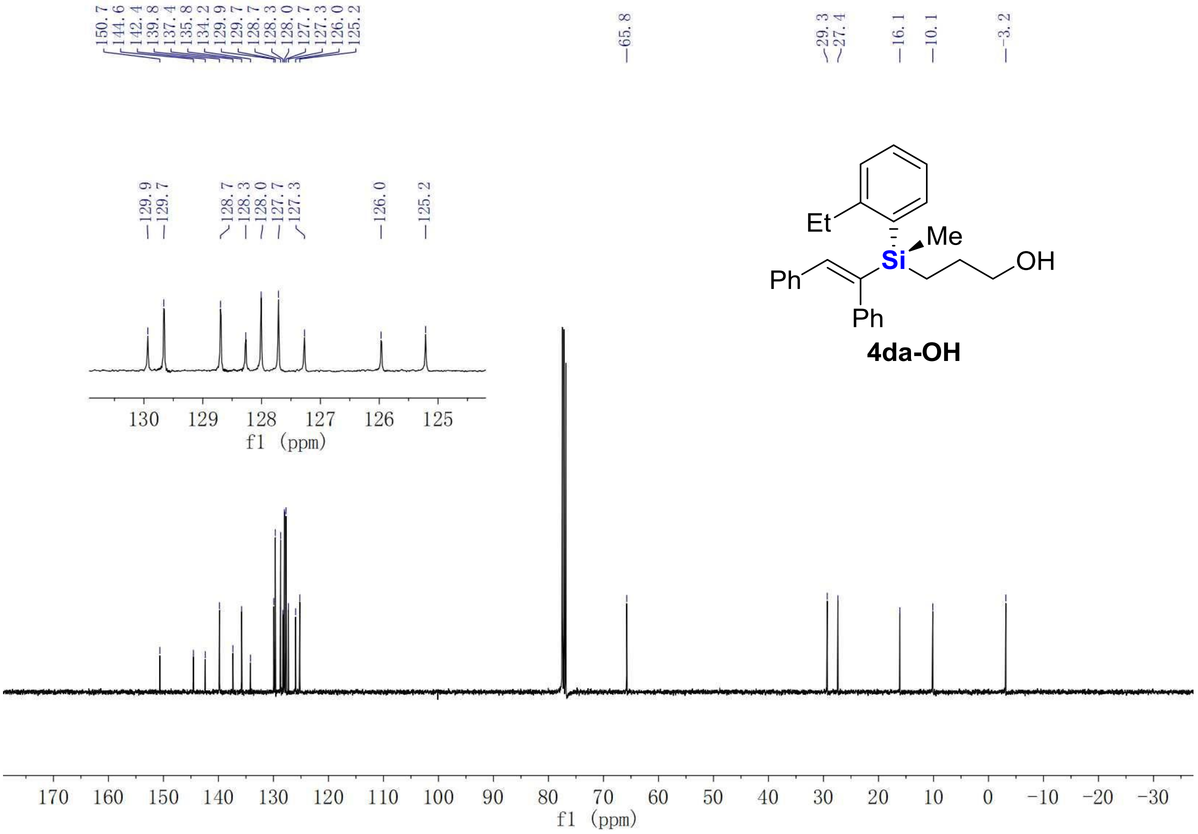

Supplementary Figure 65 ^{1}H and ^{13}C NMR Spectra for compound 4da-OH

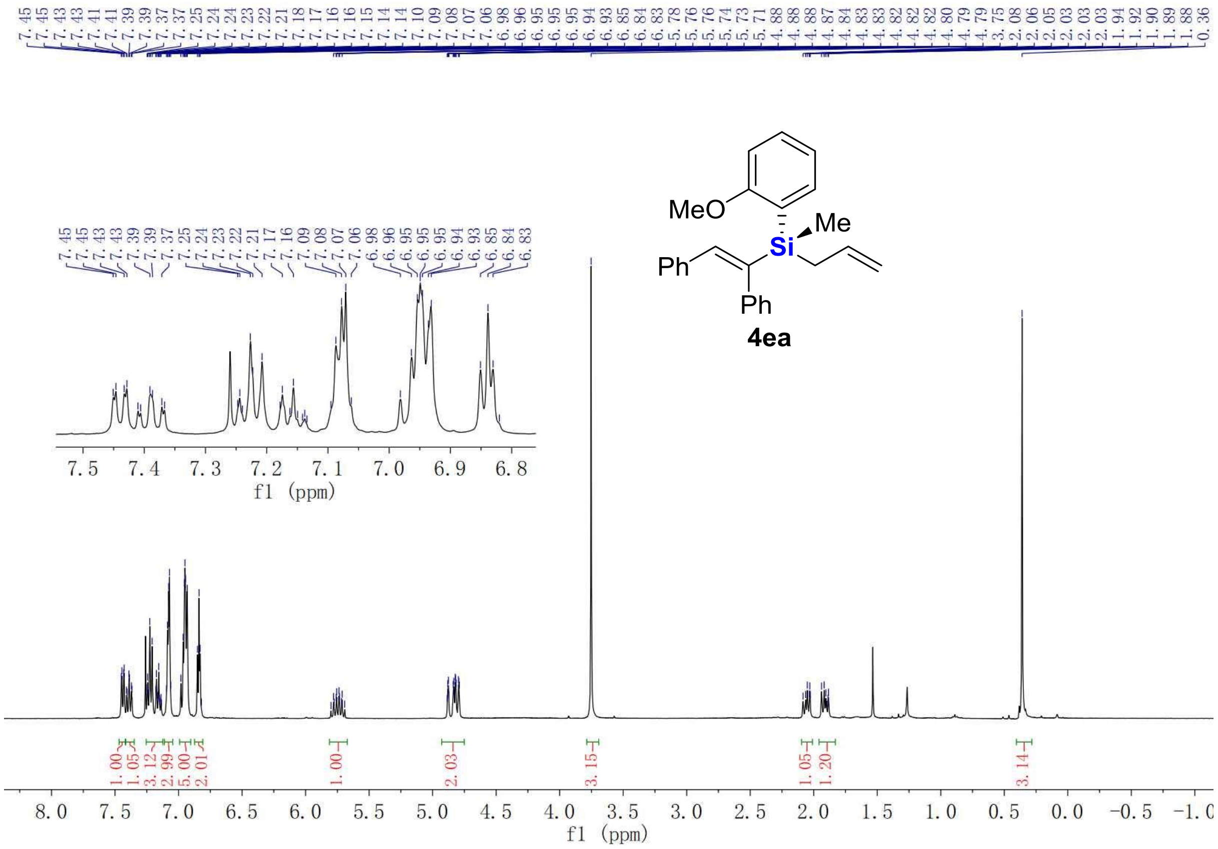

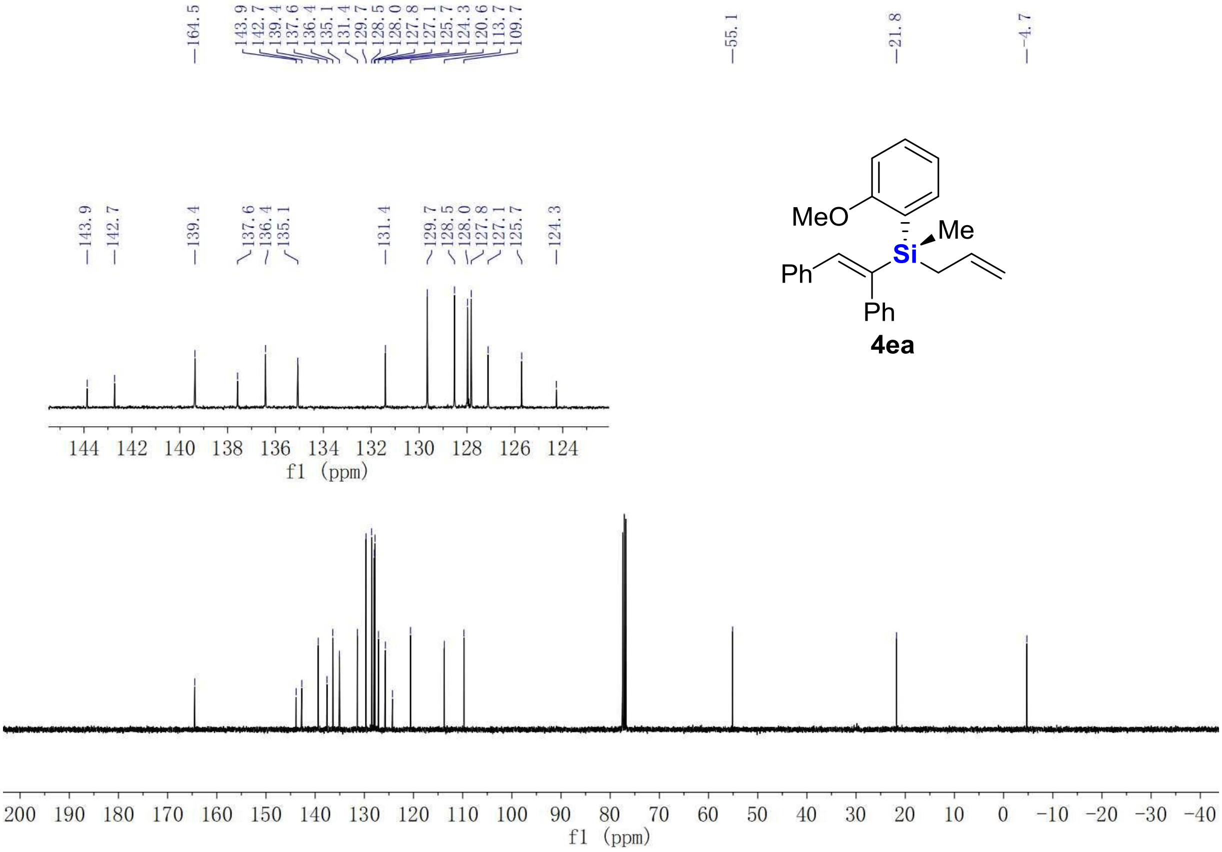

Supplementary Figure 66 ^{1}H and ^{13}C NMR Spectra for compound 4ea

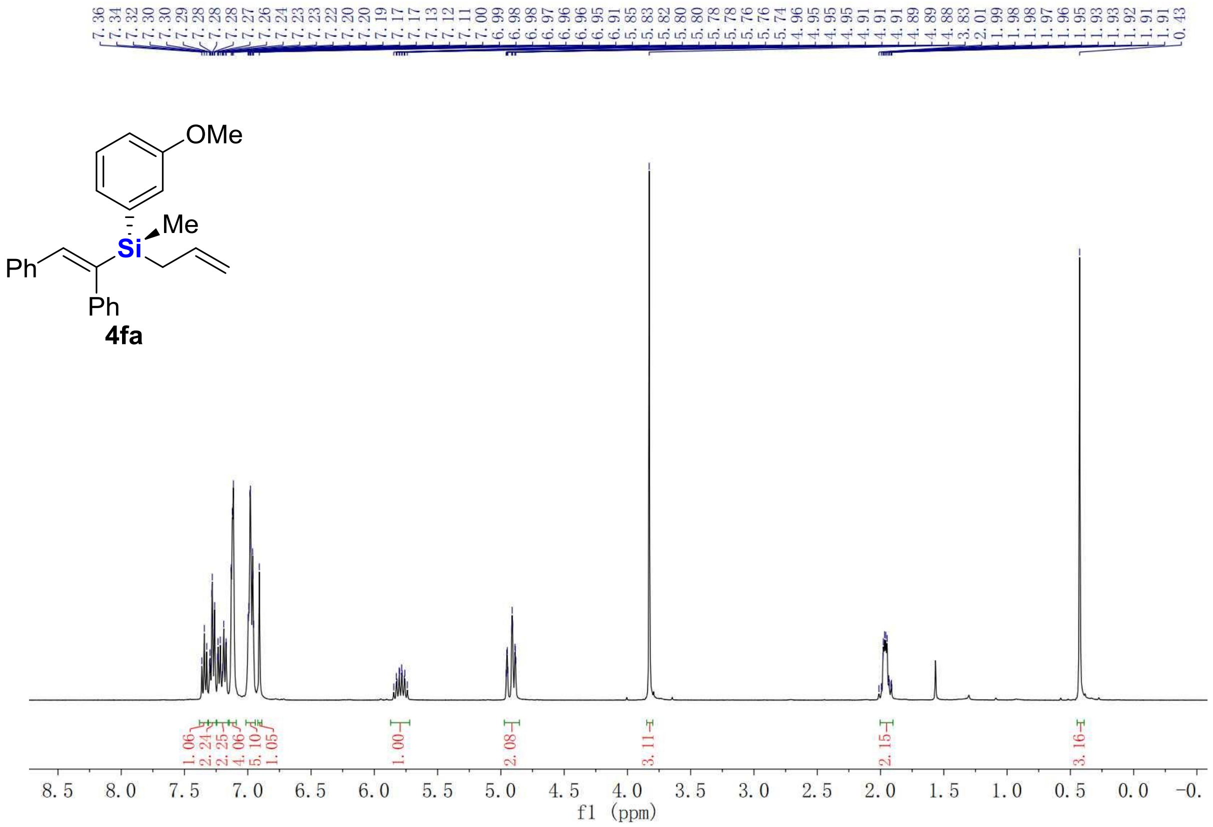

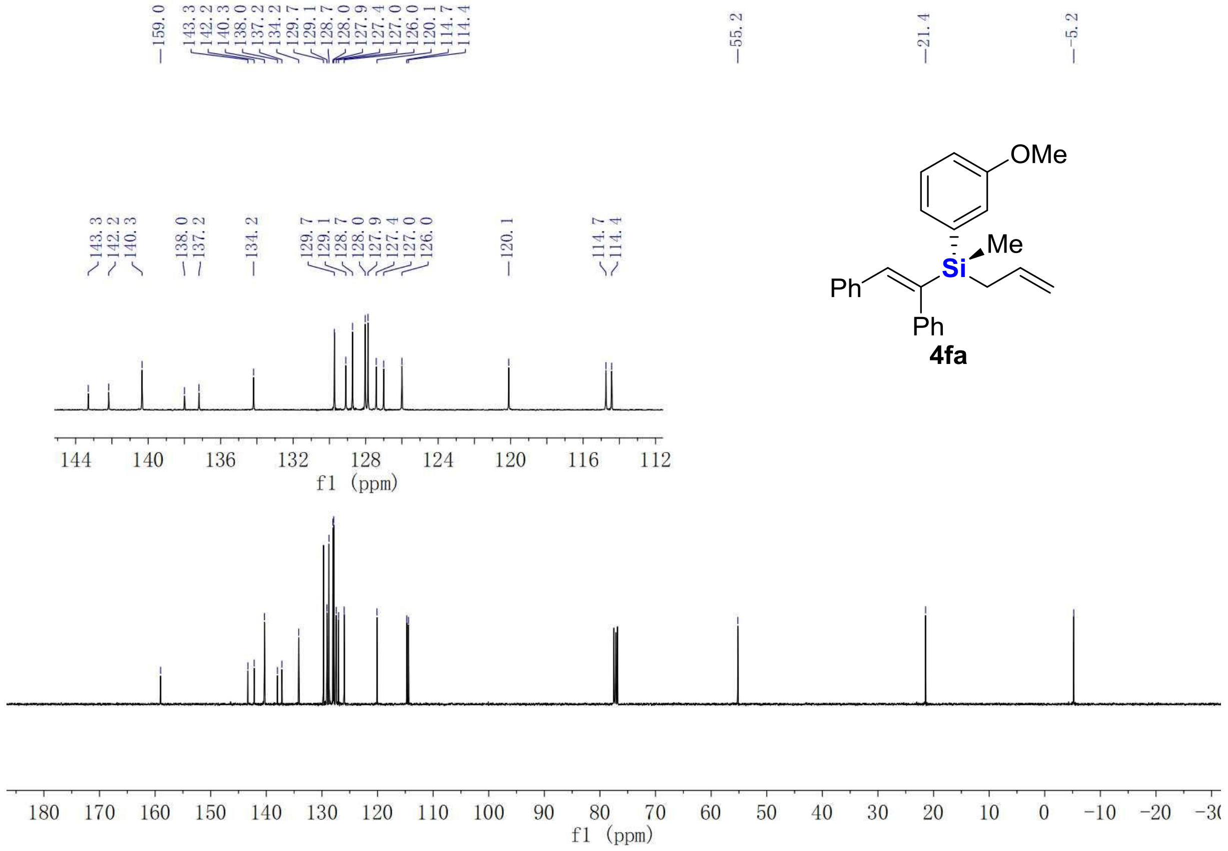

Supplementary Figure 67 ^{1}H and ^{13}C NMR Spectra for compound 4fa

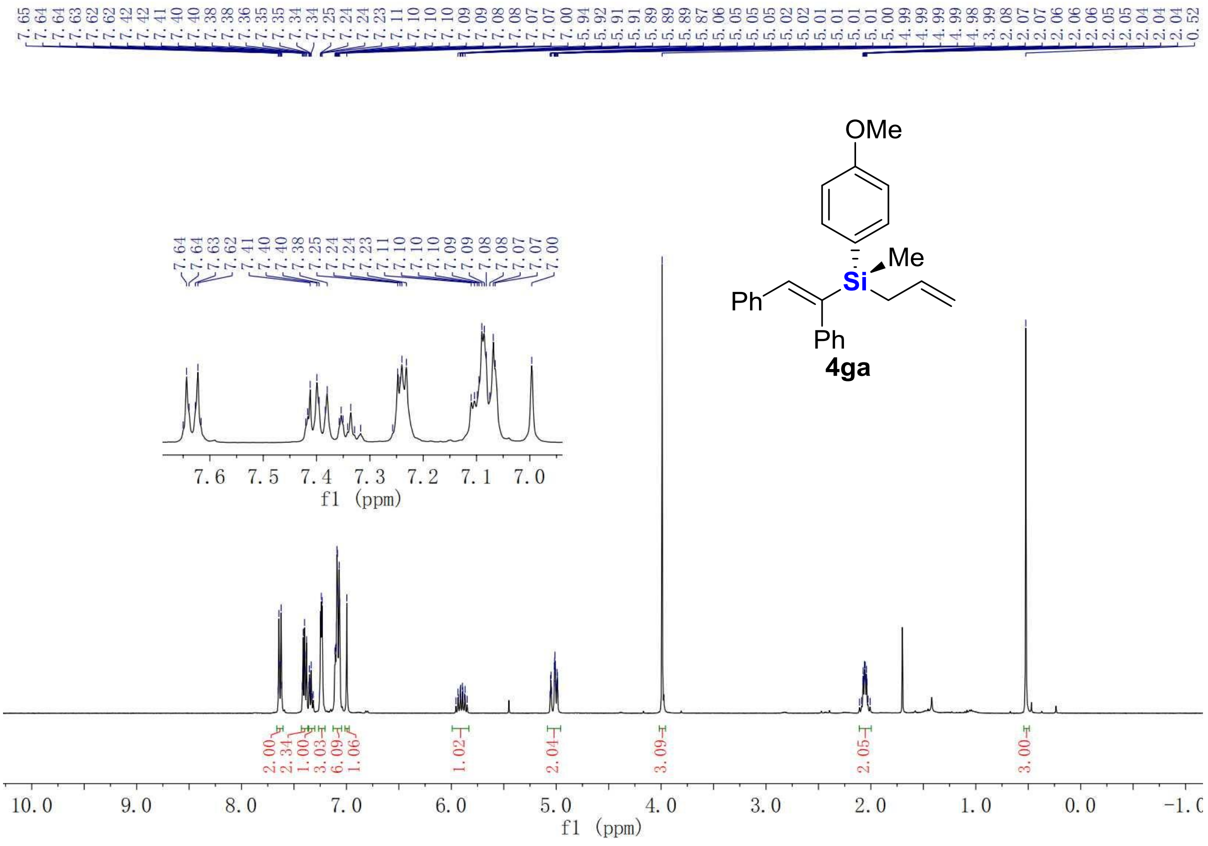

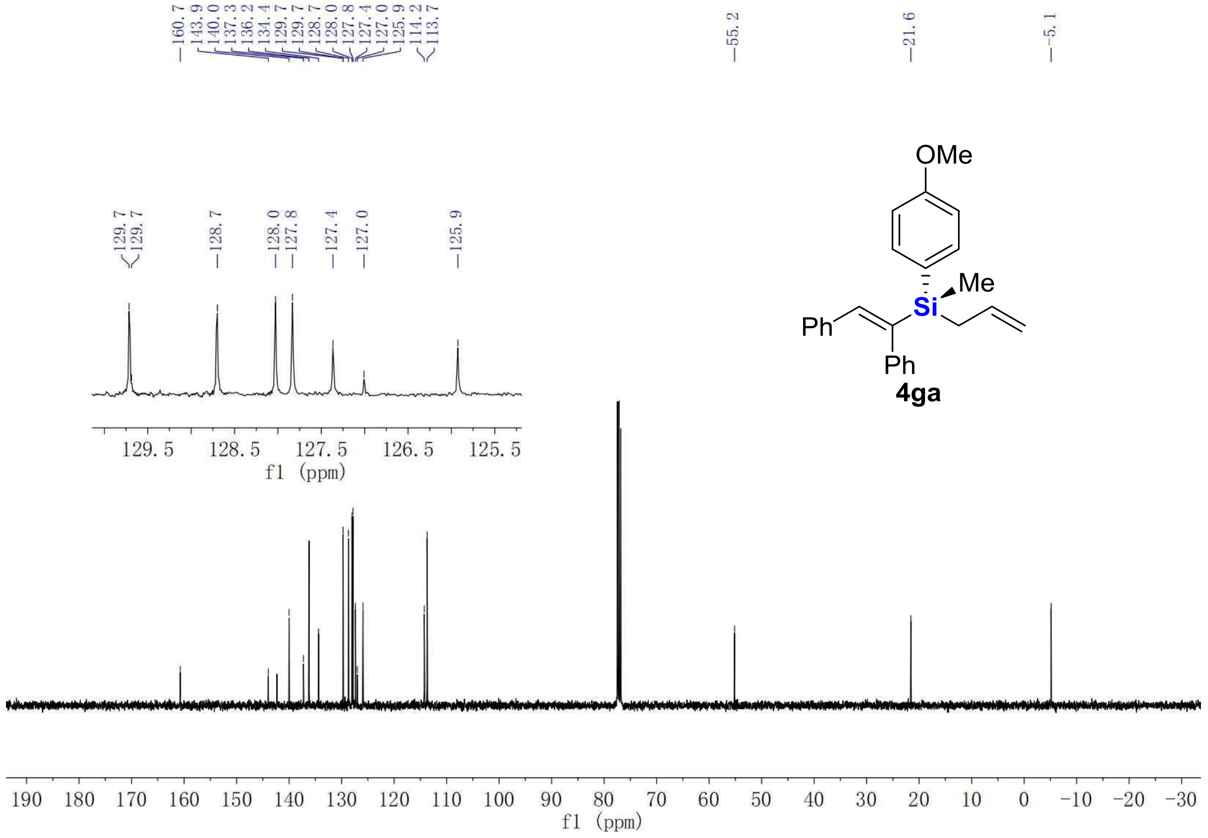

Supplementary Figure 68 ^{1}H and ^{13}C NMR Spectra for compound 4ga

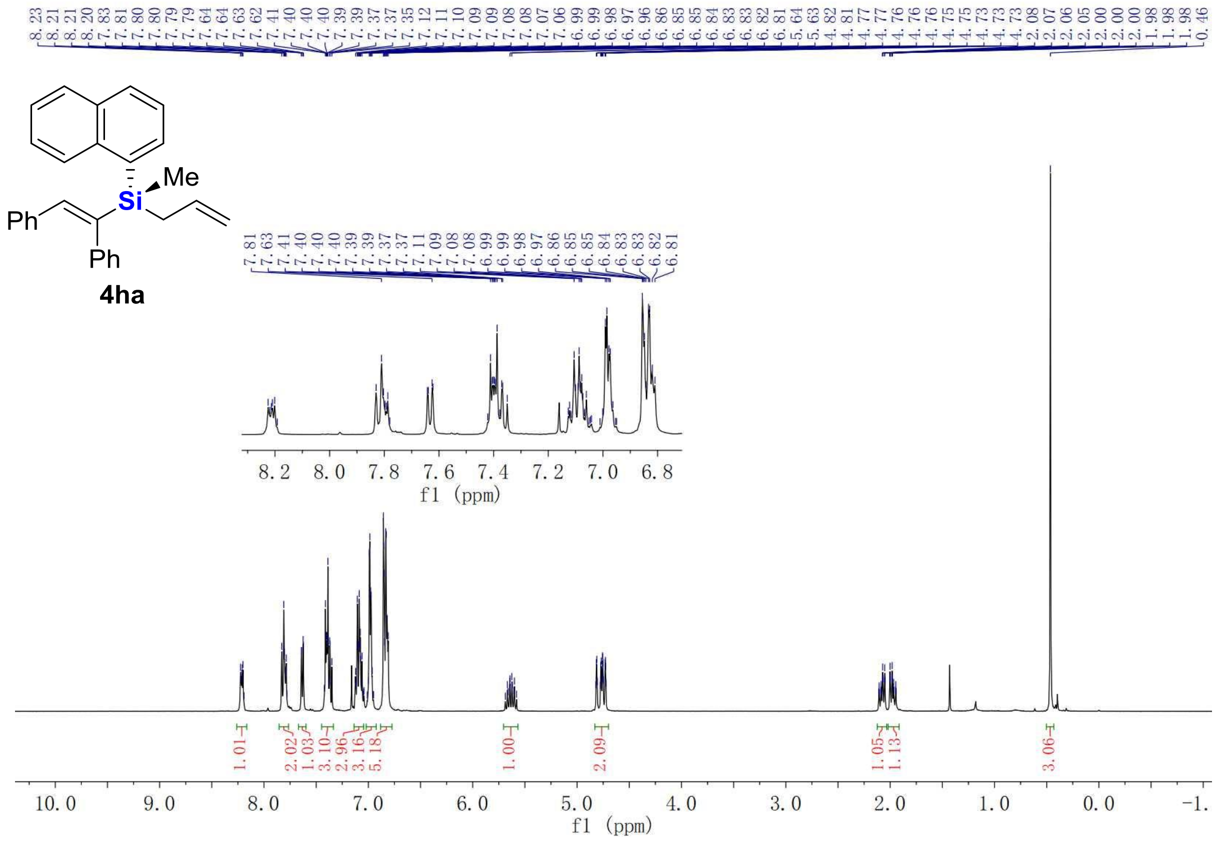

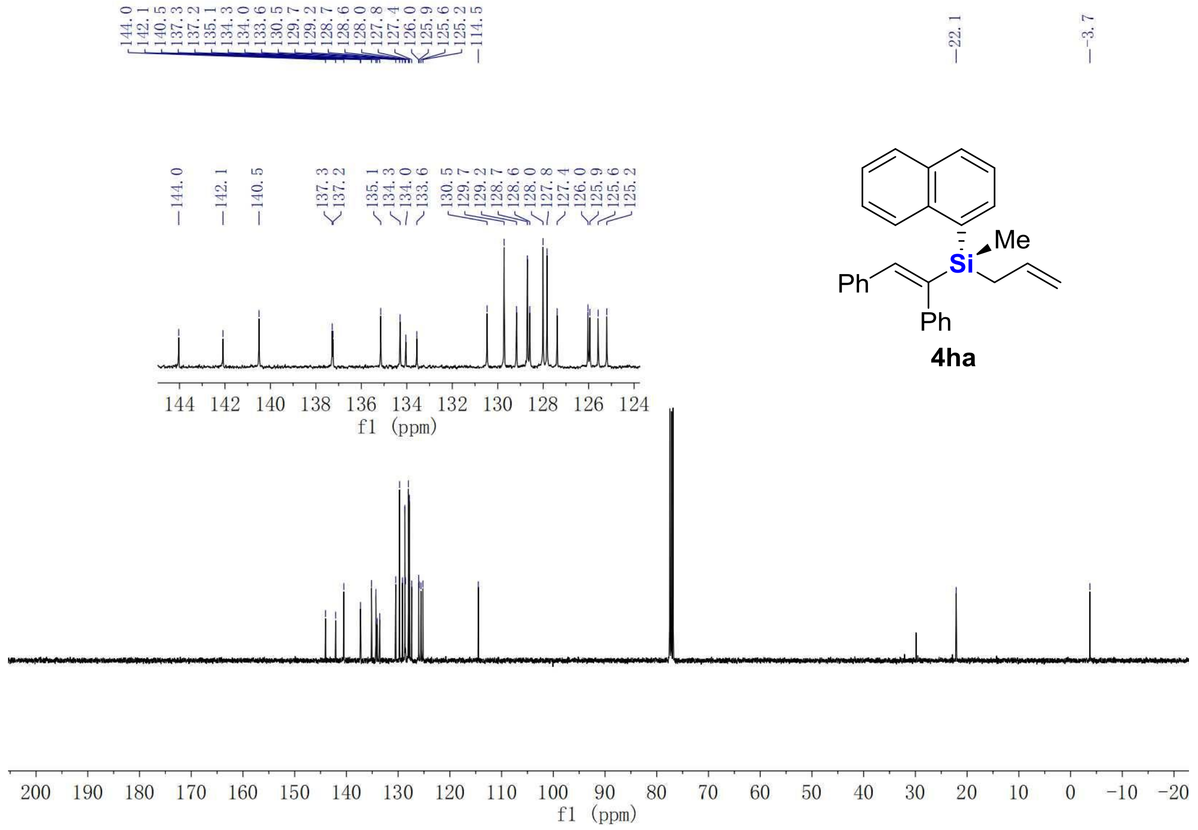

Supplementary Figure 69 ^{1}H and ^{13}C NMR Spectra for compound 4ha

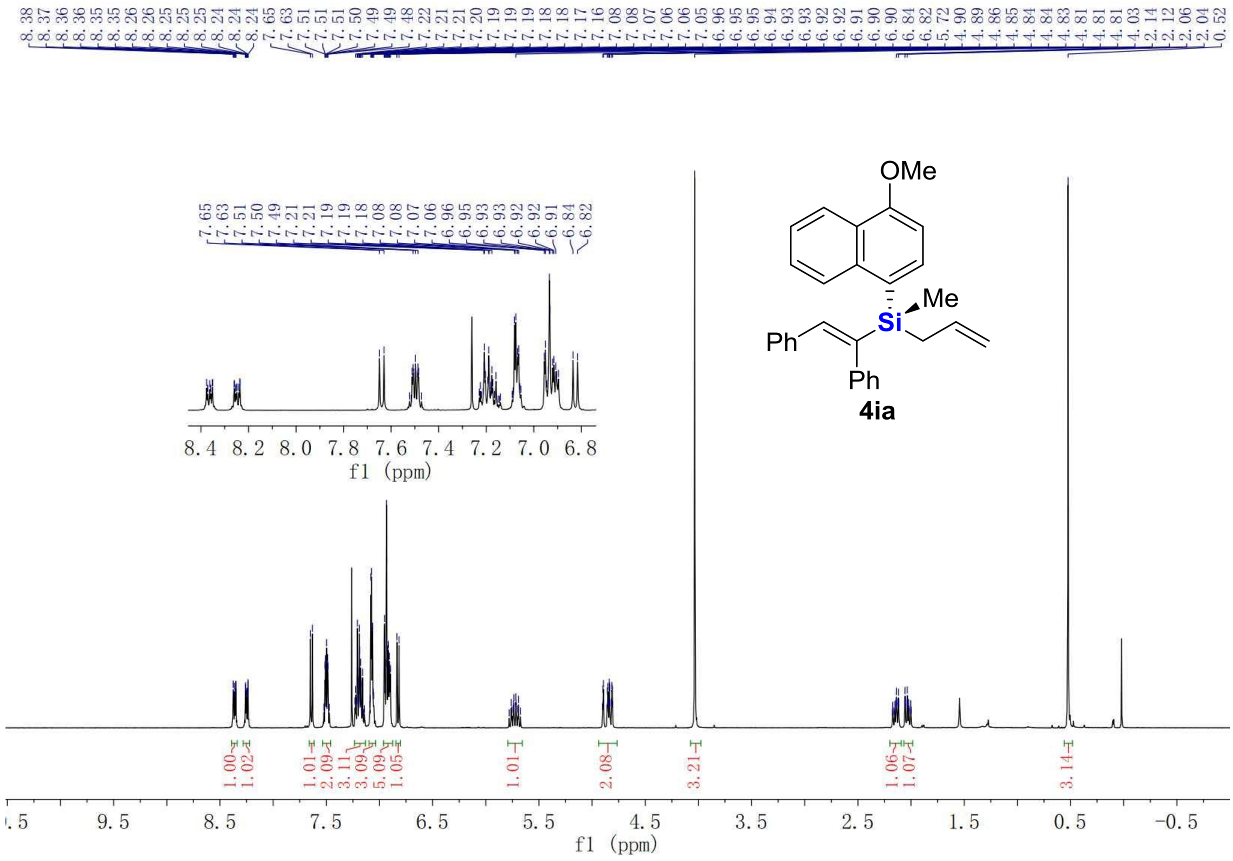

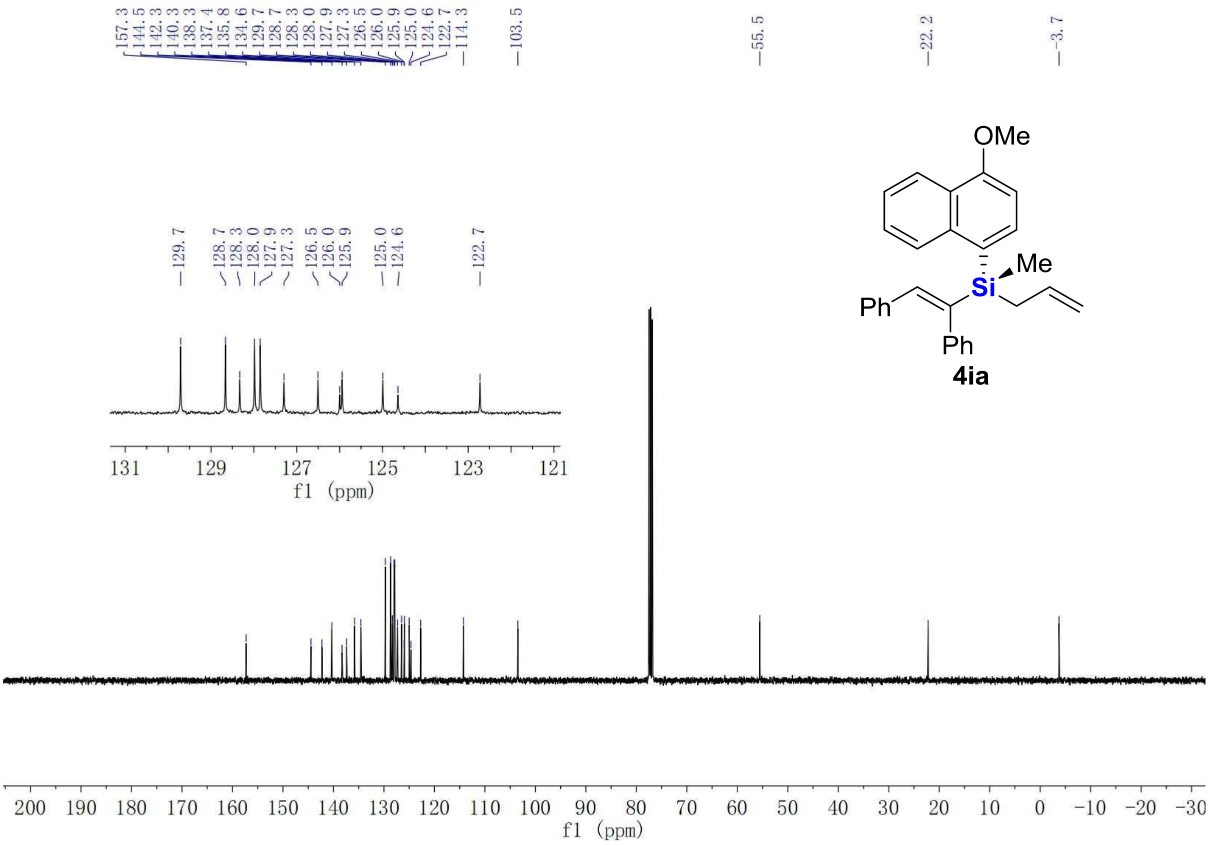

Supplementary Figure 70 ^{1}H and ^{13}C NMR Spectra for compound 4ia

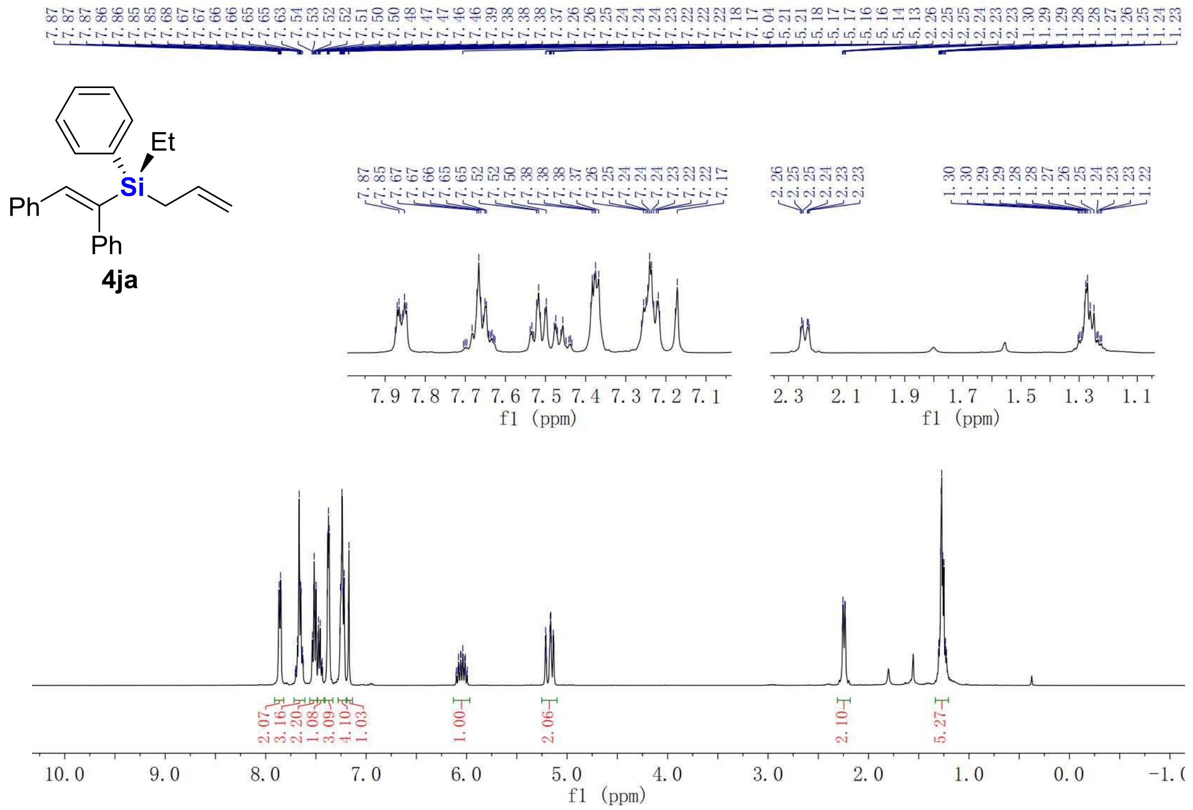

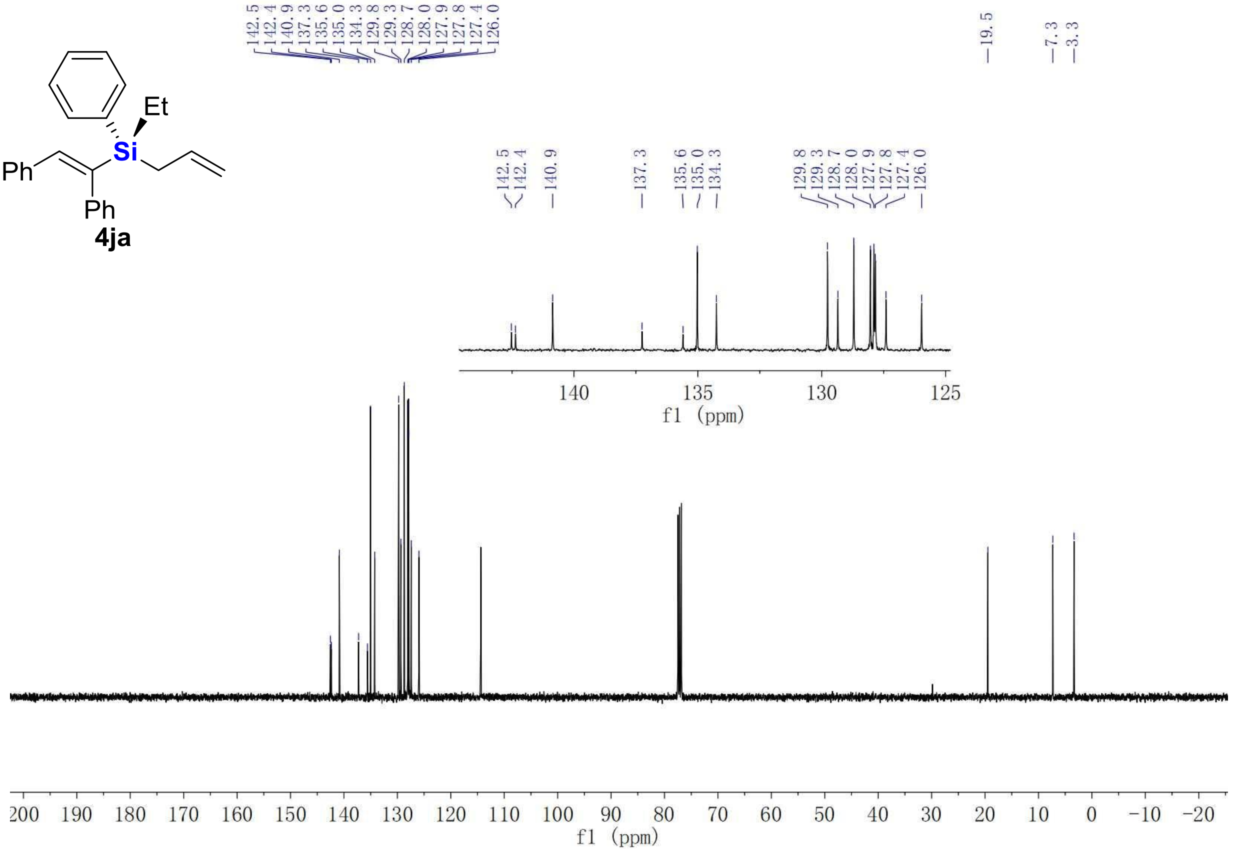

Supplementary Figure 71 ^{1}H and ^{13}C NMR Spectra for compound 4ja

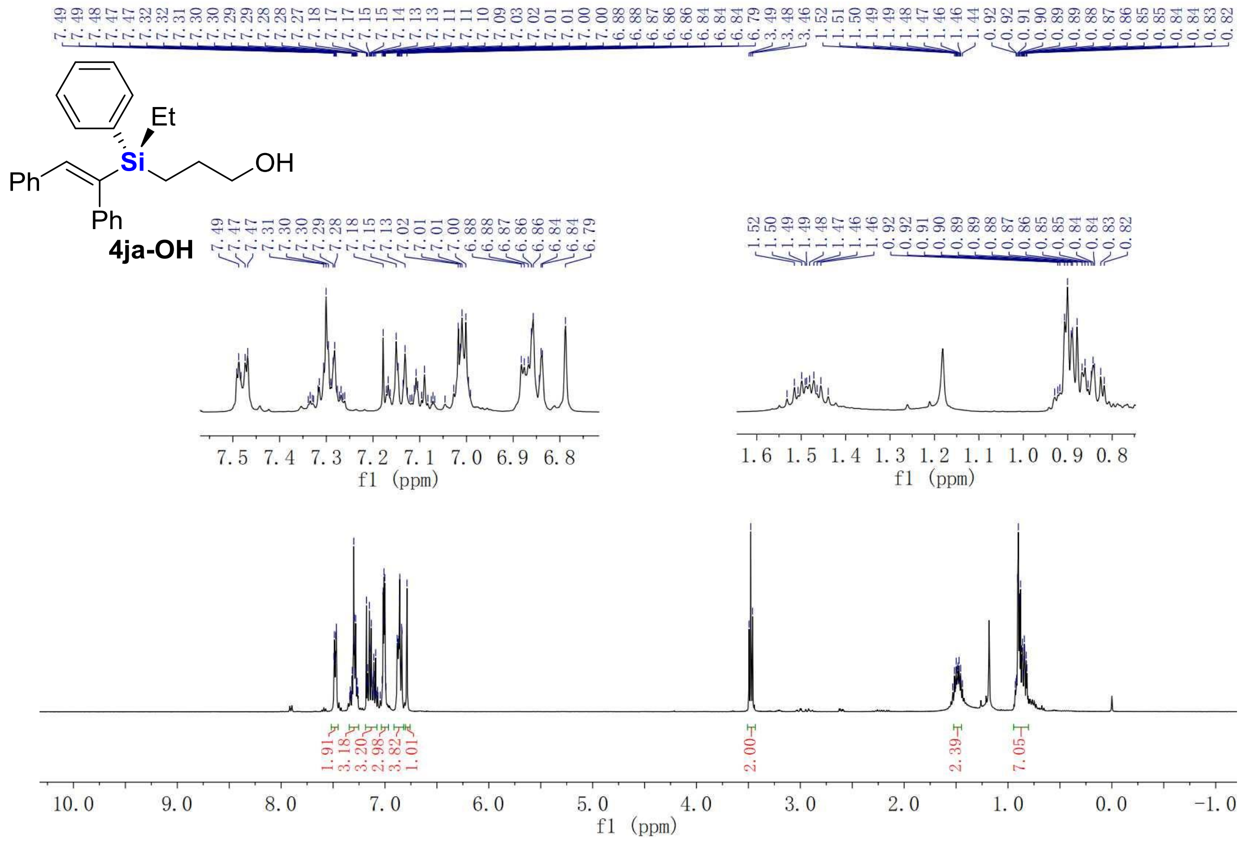

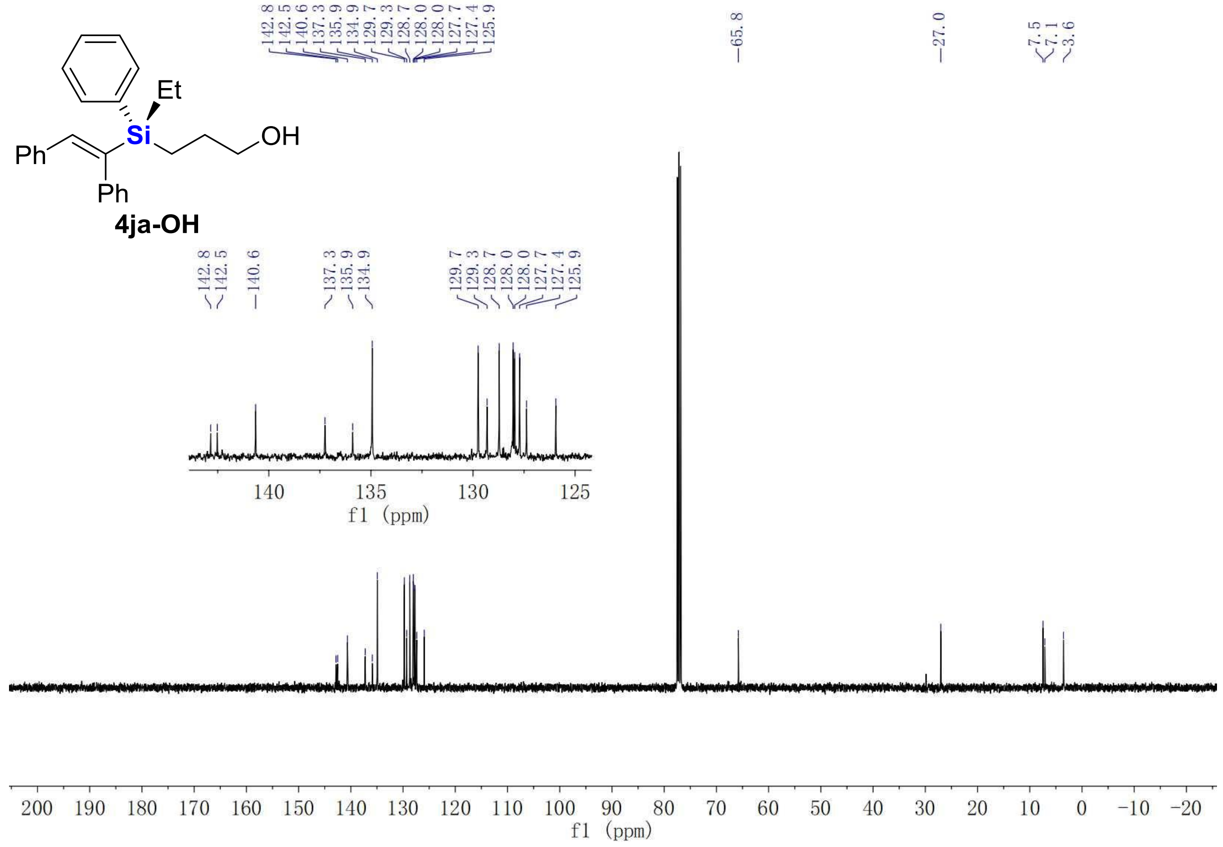

Supplementary Figure 72 ^{1}H and ^{13}C NMR Spectra for compound 4ja-OH

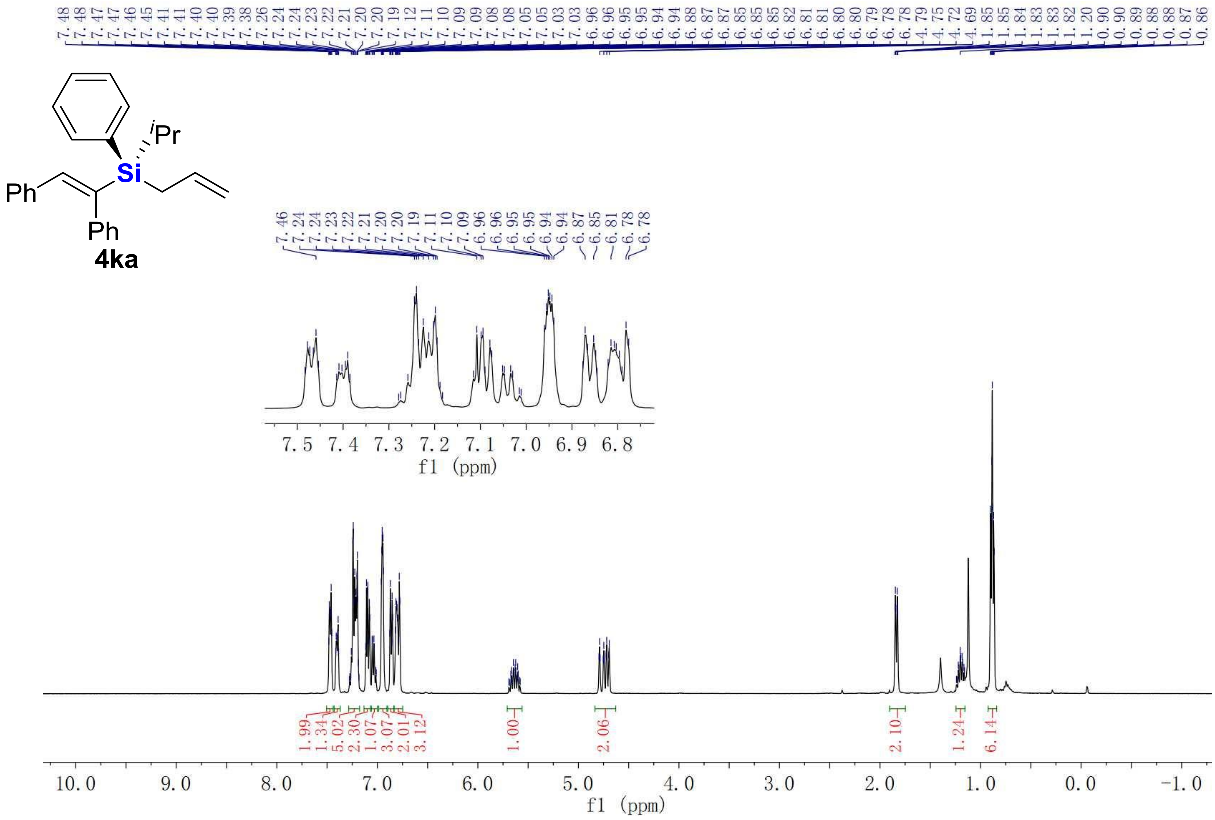

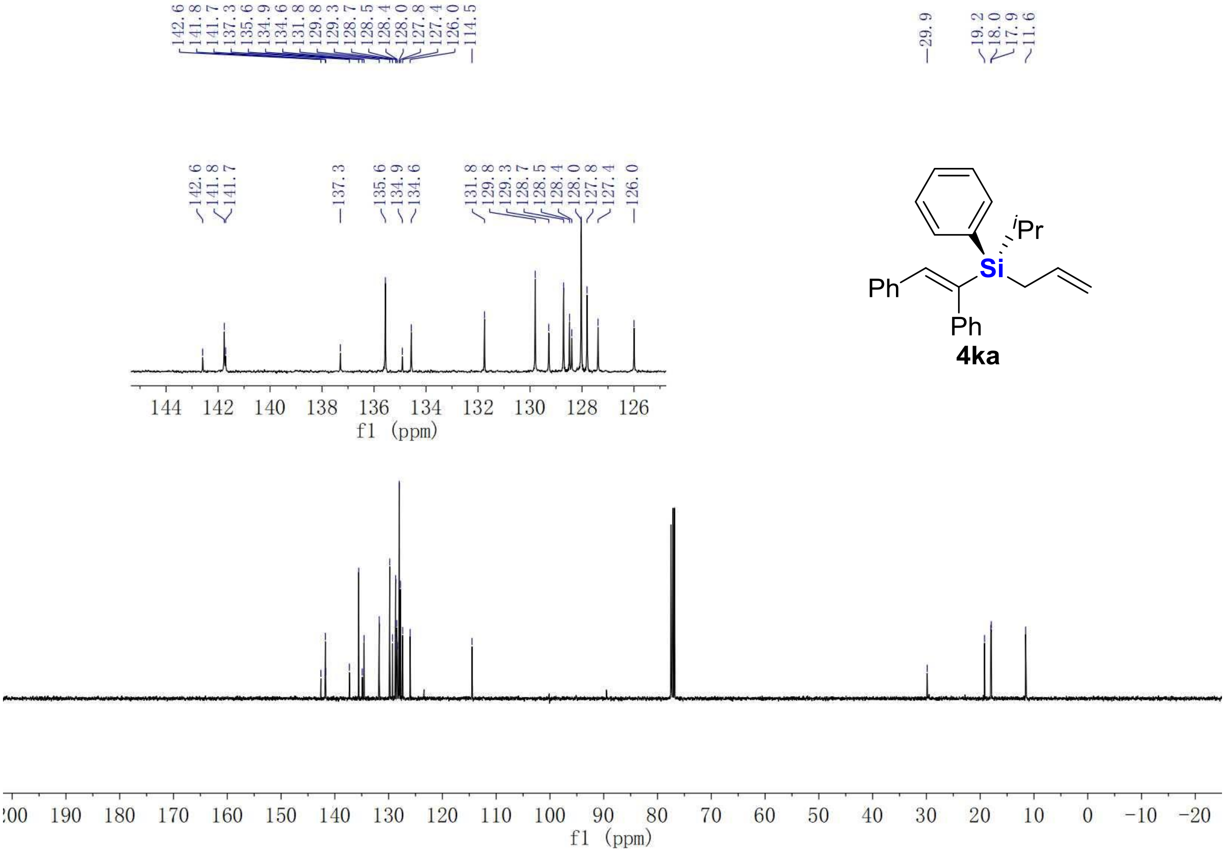

Supplementary Figure 73 ^{1}H and ^{13}C NMR Spectra for compound 4ka

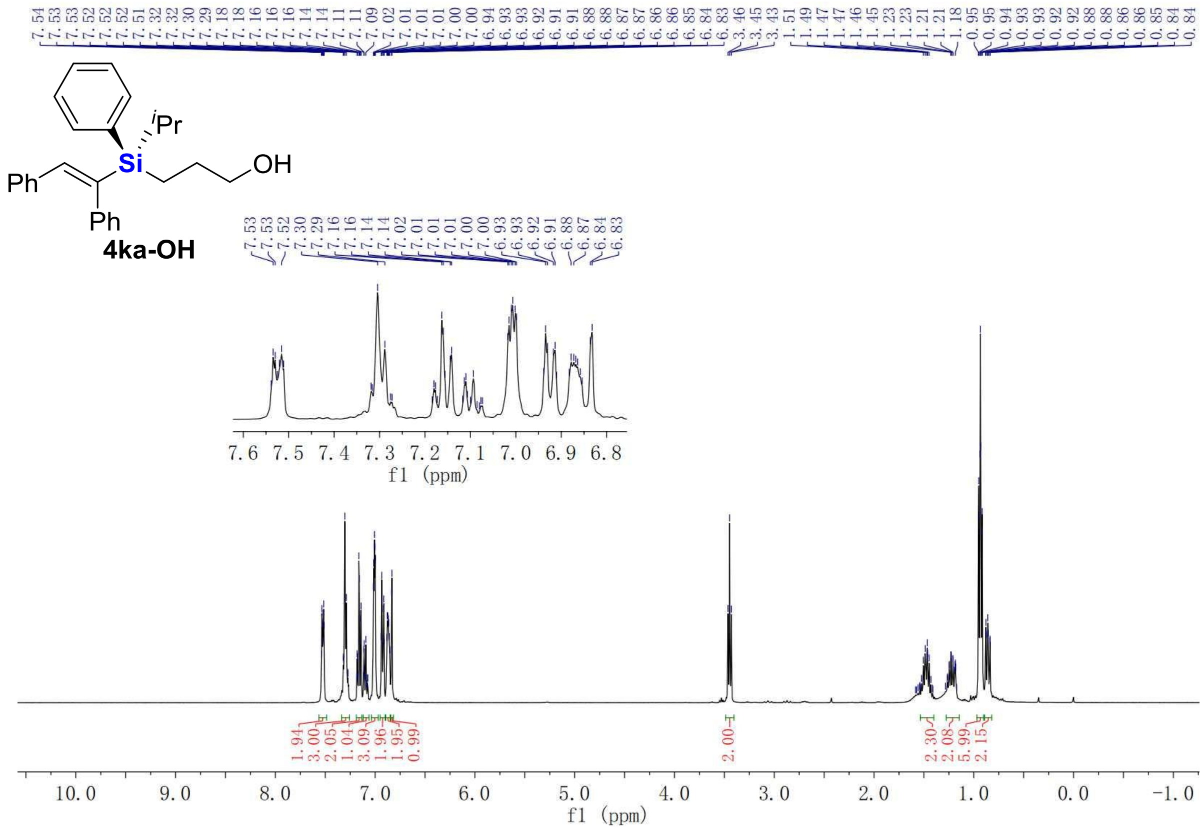

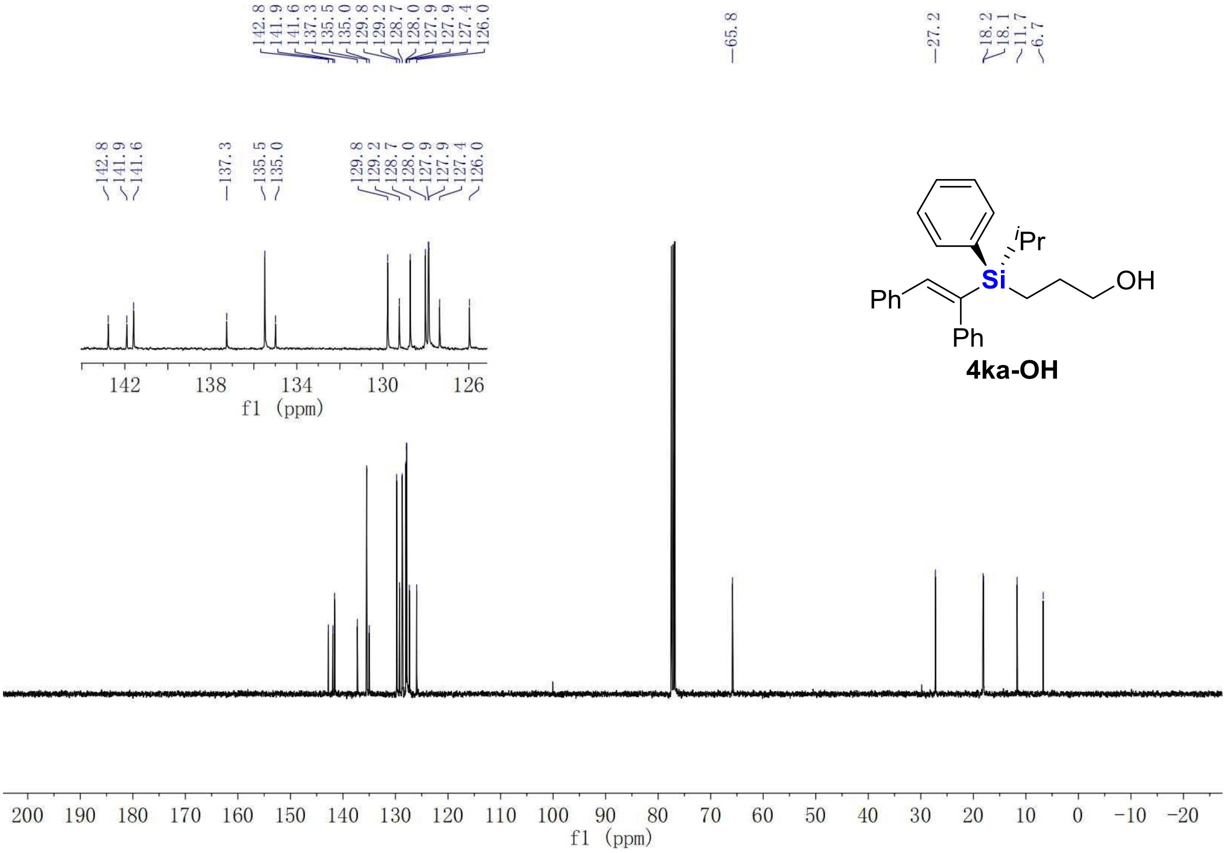

Supplementary Figure 74 ^{1}H and ^{13}C NMR Spectra for compound 4ka-OH

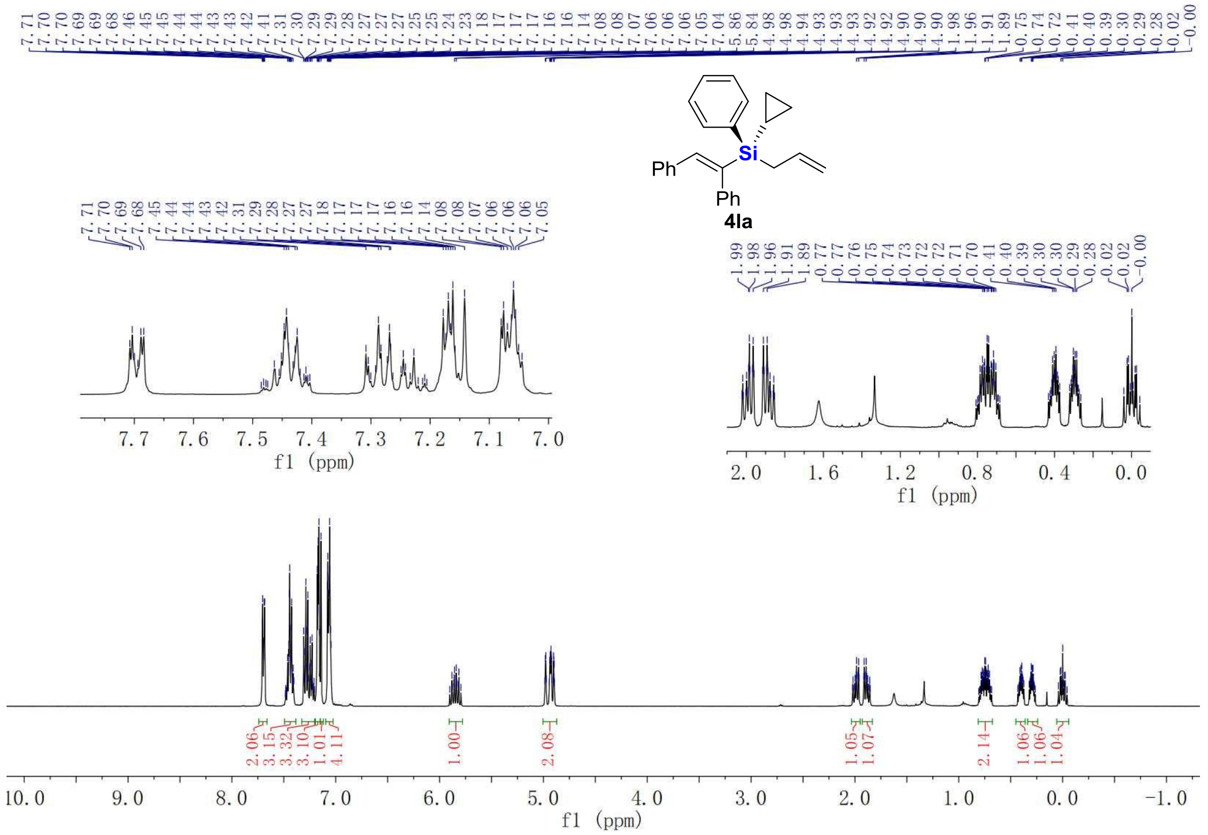

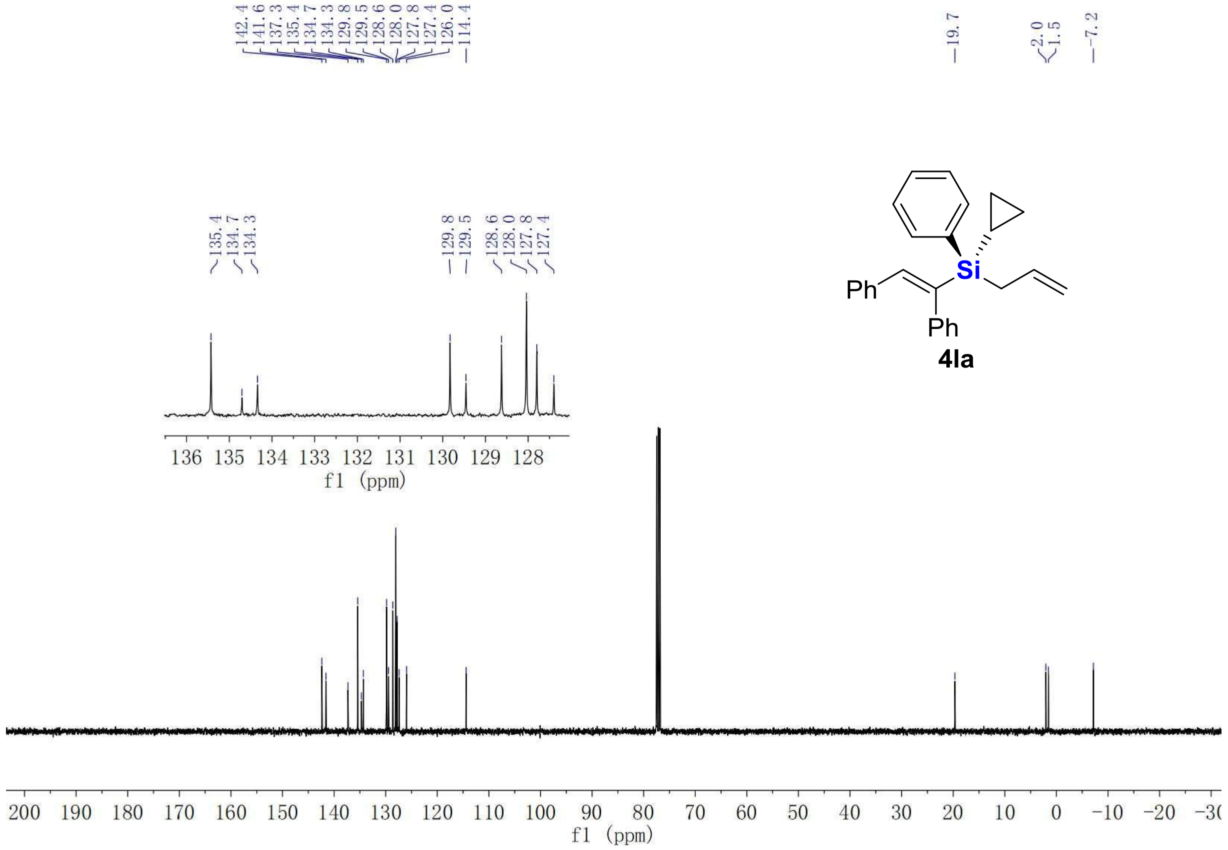

Supplementary Figure 75 ^{1}H and ^{13}C NMR Spectra for compound 4la

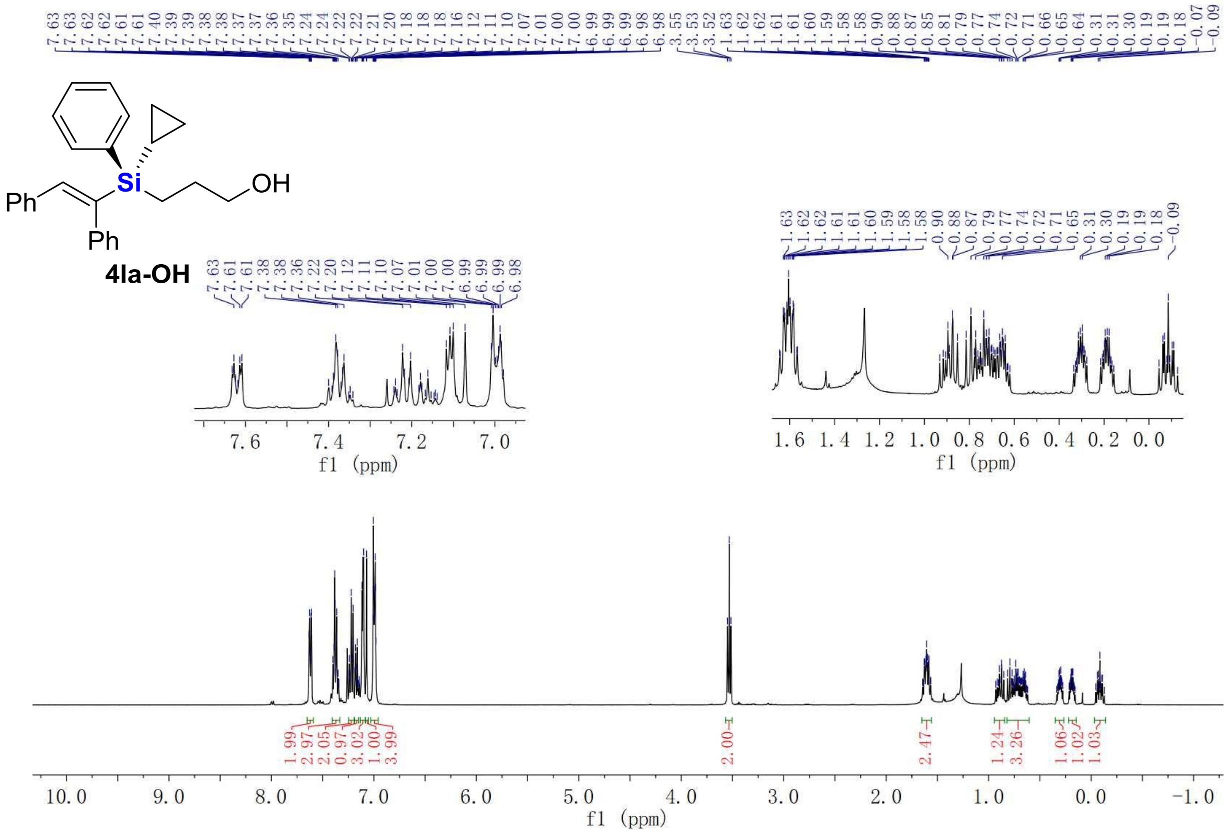

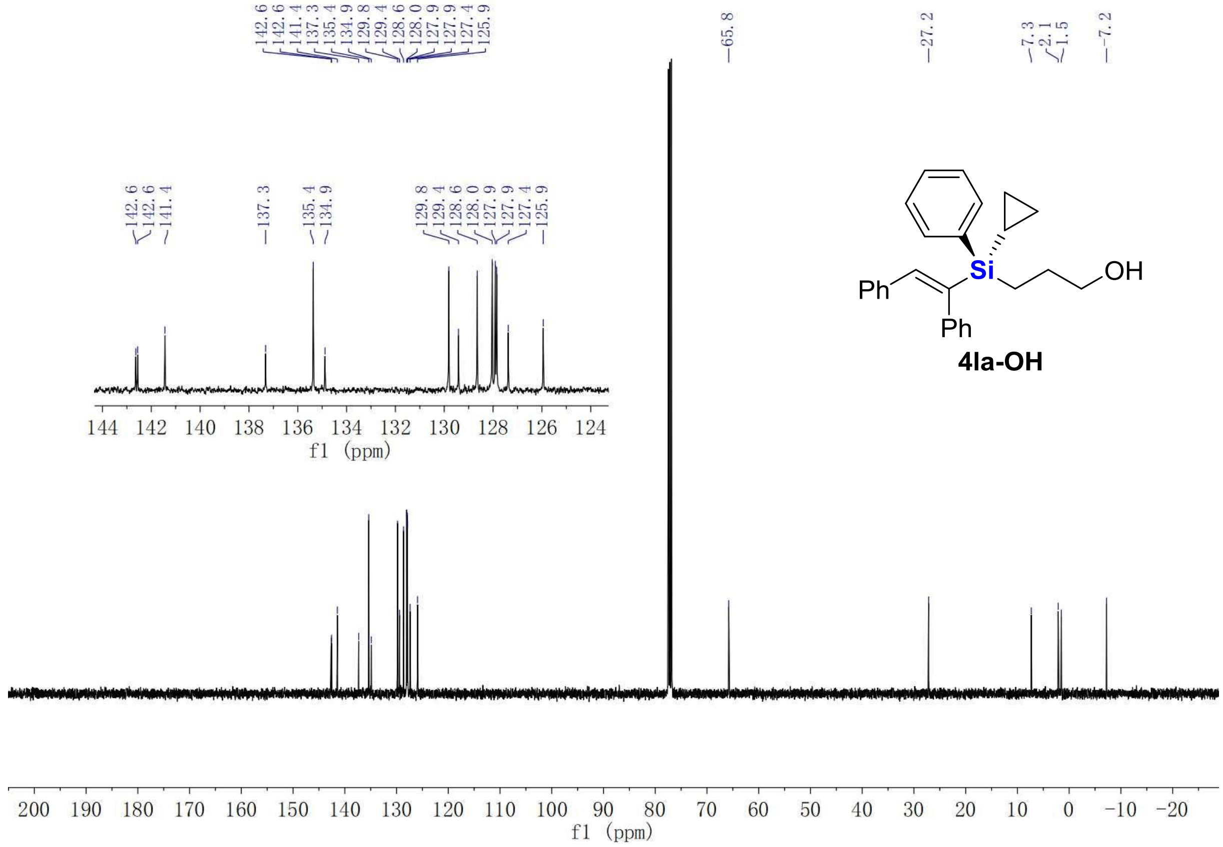

Supplementary Figure 76 ^{1}H and ^{13}C NMR Spectra for compound 4la-OH

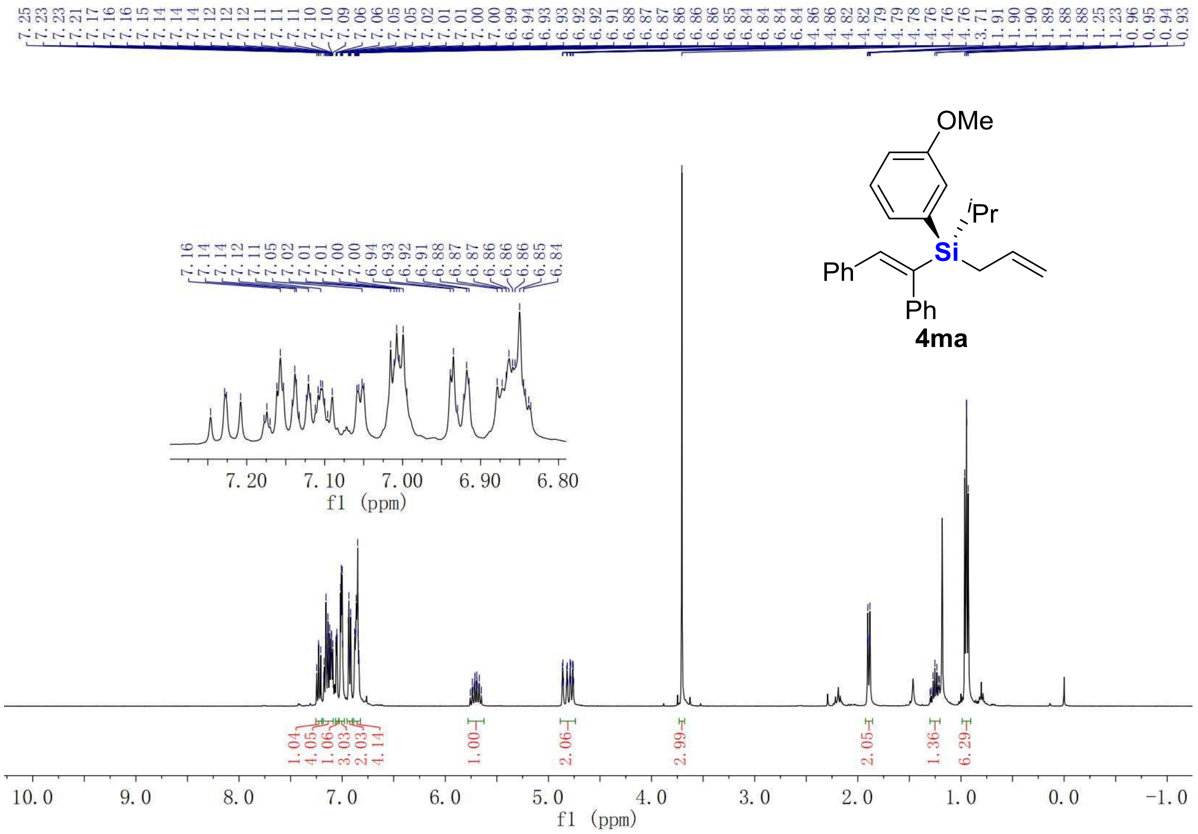

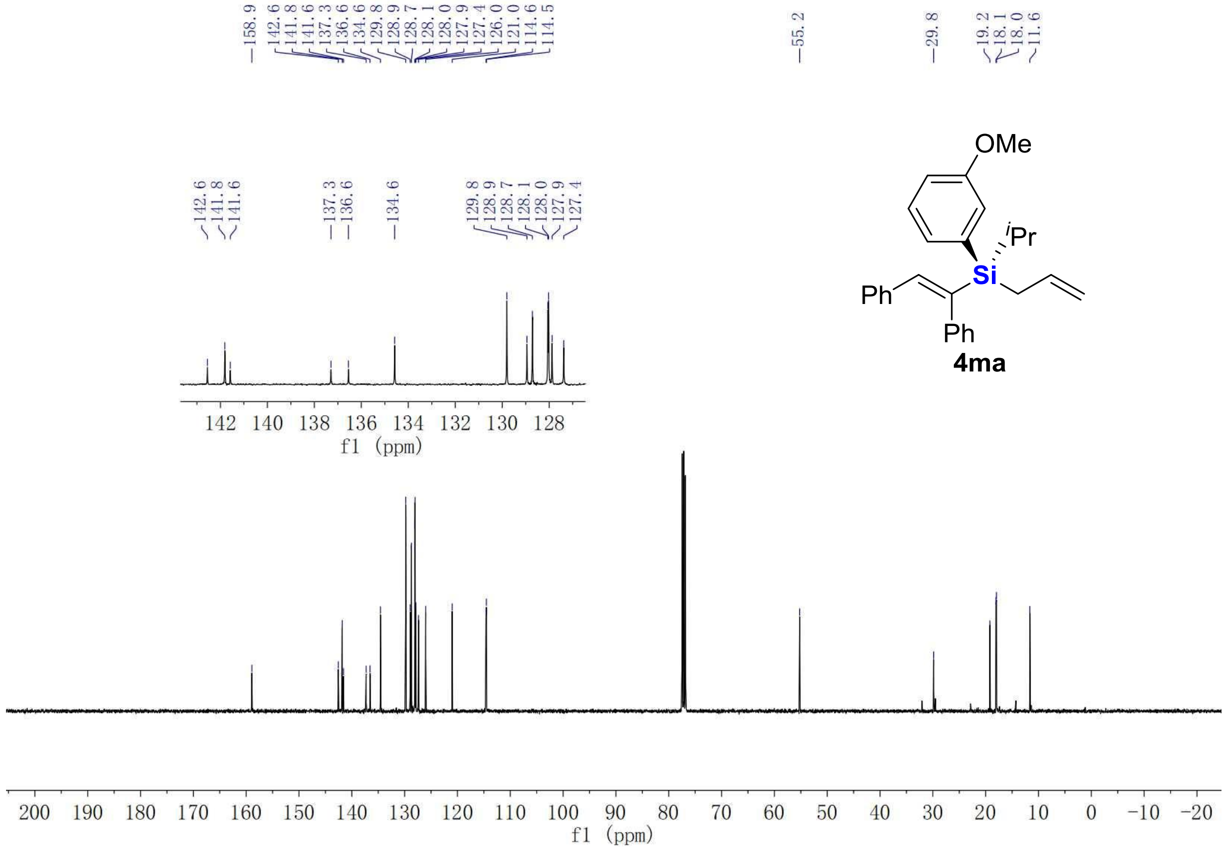

Supplementary Figure 77 ^{1}H and ^{13}C NMR Spectra for compound 4ma

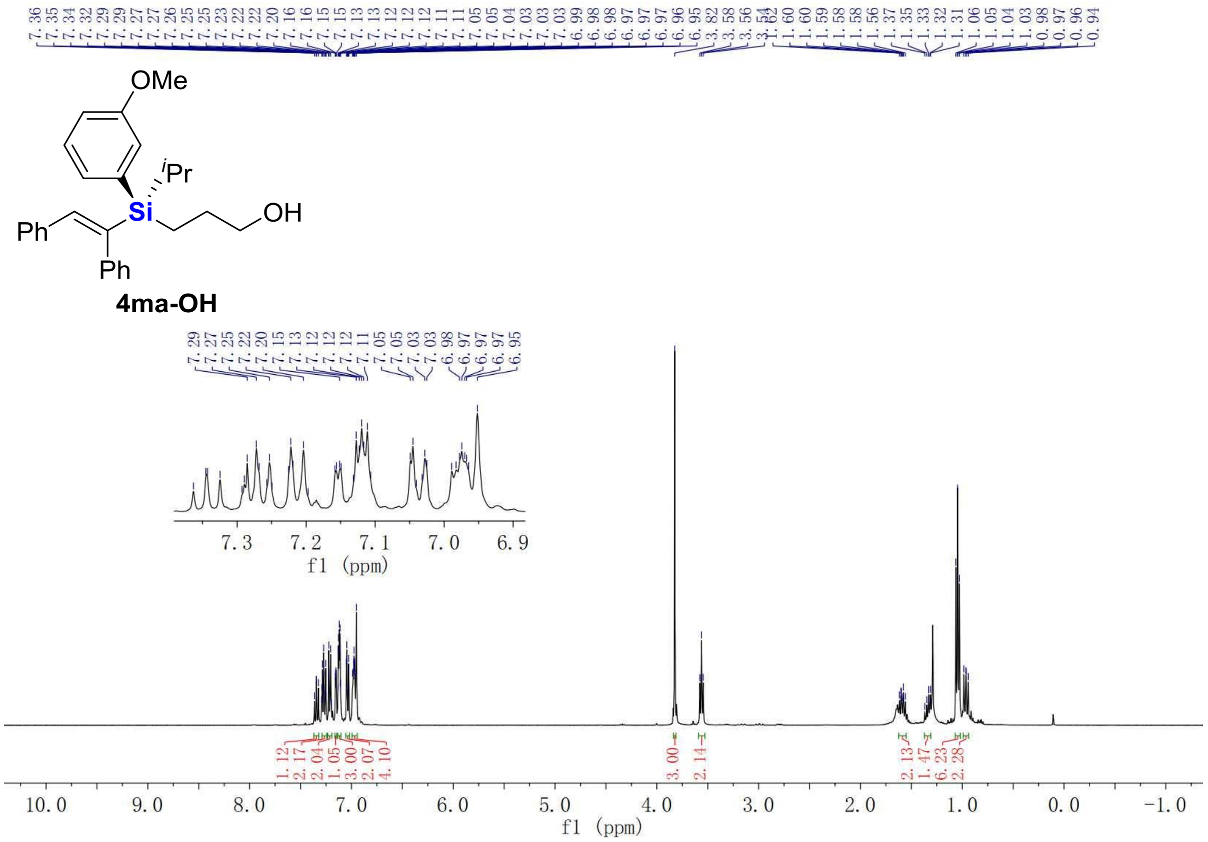

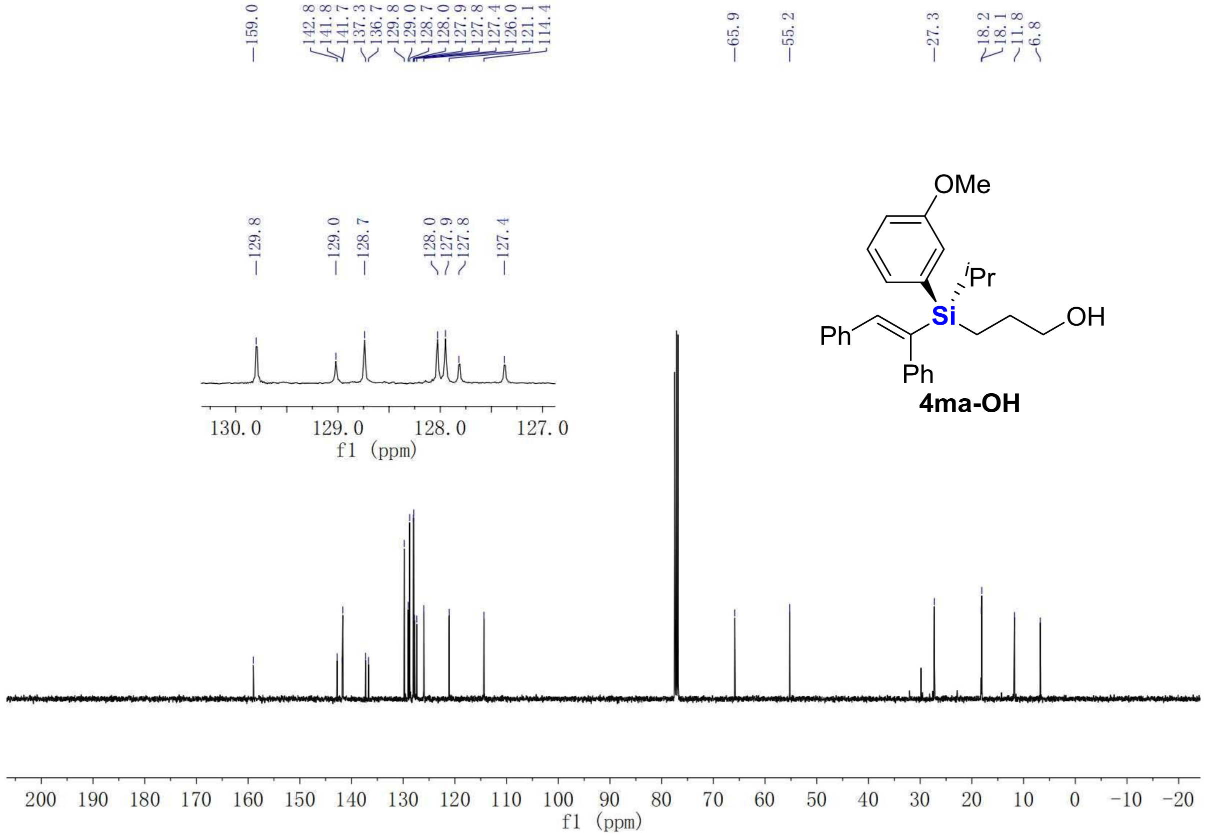

Supplementary Figure 78 ^{1}H and ^{13}C NMR Spectra for compound 4ma-OH

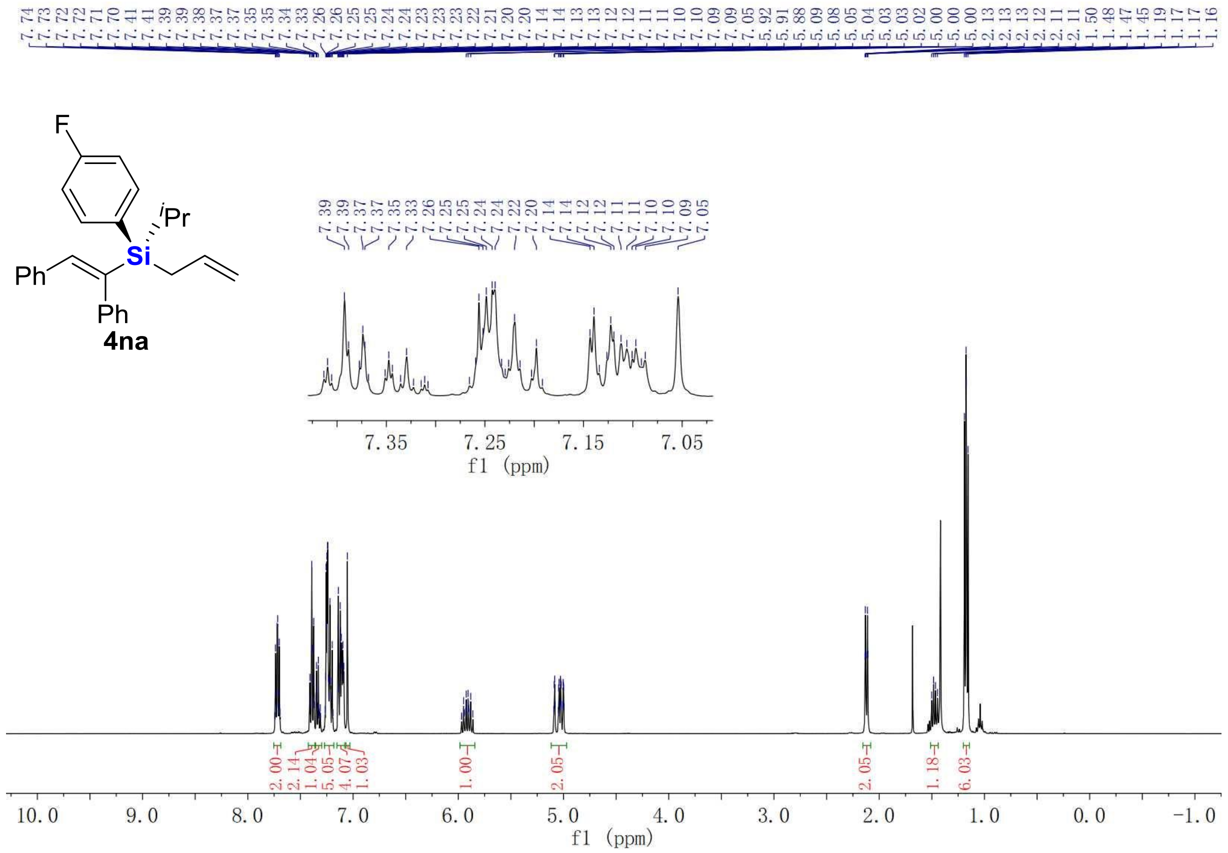
7.74 7.73 7.72 7.71 7.70 7.41 7.39 7.39 7.38 7.37 7.37 7.35 7.35 7.34 7.33 7.26 7.26 7.25 7.25 7.24 7.24 7.23 7.23 7.23 7.22 7.21 7.20 7.20 7.14 7.13 7.13 7.12 7.12 7.11 7.11 7.10 7.10 7.09 7.09 7.05 5.92 5.91 5.88 5.09 5.08 5.05 5.04 5.03 5.03 5.02 5.00 5.00 5.00 2.13 2.13 2.13 2.12 2.11 1.50 1.48 1.47 1.45 1.19 1.17 1.17 1.16
F
iPr
Si
Ph
Ph
4na
7.39 7.39 7.37 7.37 7.35 7.33 7.26 7.25 7.25 7.24 7.24 7.22 7.20 7.14 7.14 7.12 7.12 7.11 7.11 7.10 7.10 7.09 7.05
7.35 7.25 7.15 7.05
f1 (ppm)
2.00 2.14 1.04 5.05 4.07 1.03 1.00 2.05 2.05 1.18 6.03
10.0 9.0 8.0 7.0 6.0 5.0 4.0 3.0 2.0 1.0 0.0 -1.0
f1 (ppm)

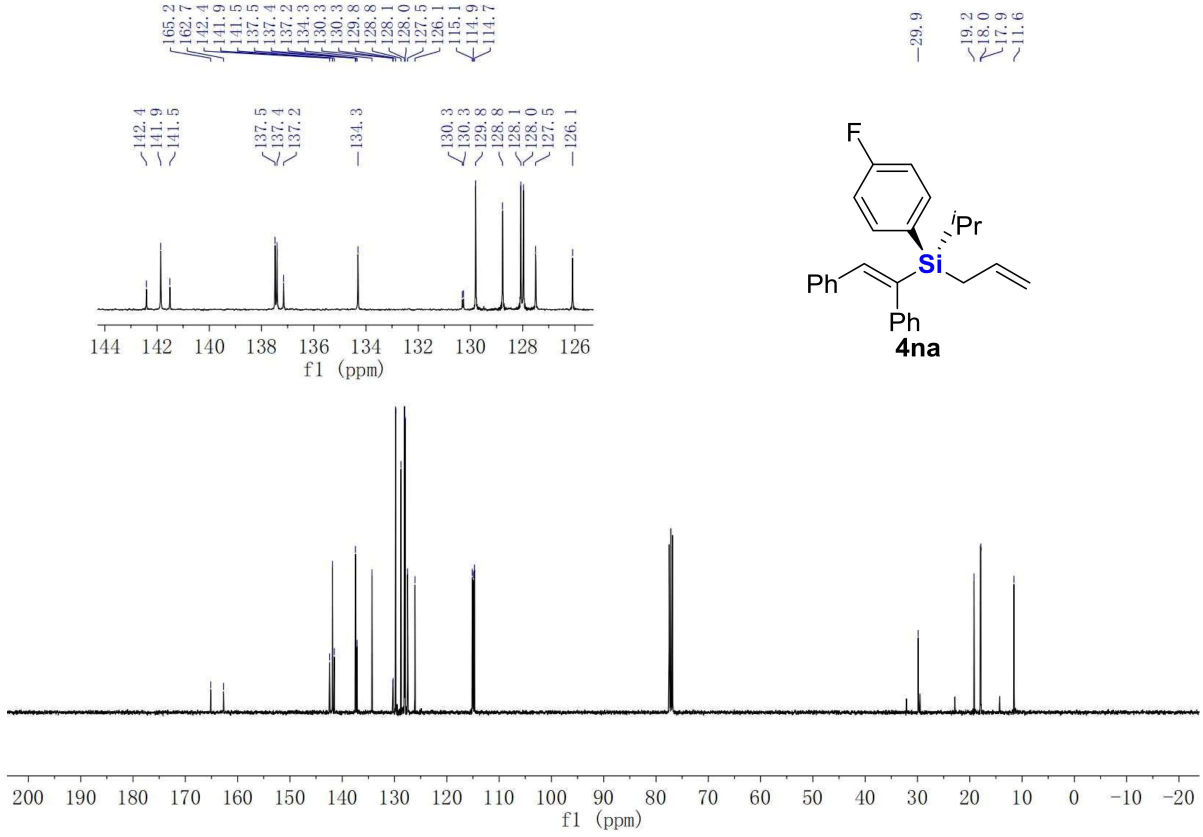
165.2 162.7 142.4 141.9 141.5 137.5 137.4 137.2 134.3 130.3 130.3 129.8 128.8 128.1 128.0 127.5 126.1 115.1 114.9 114.7
29.9
19.2 18.0 17.9 11.6
142.4 141.9 141.5 137.5 137.4 137.2 134.3 130.3 130.3 129.8 128.8 128.1 128.0 127.5 126.1
144 142 140 138 136 134 132 130 128 126
f1 (ppm)
F
iPr
Si
Ph
Ph
4na
200 190 180 170 160 150 140 130 120 110 100 90 80 70 60 50 40 30 20 10 0 -10 -20
f1 (ppm)

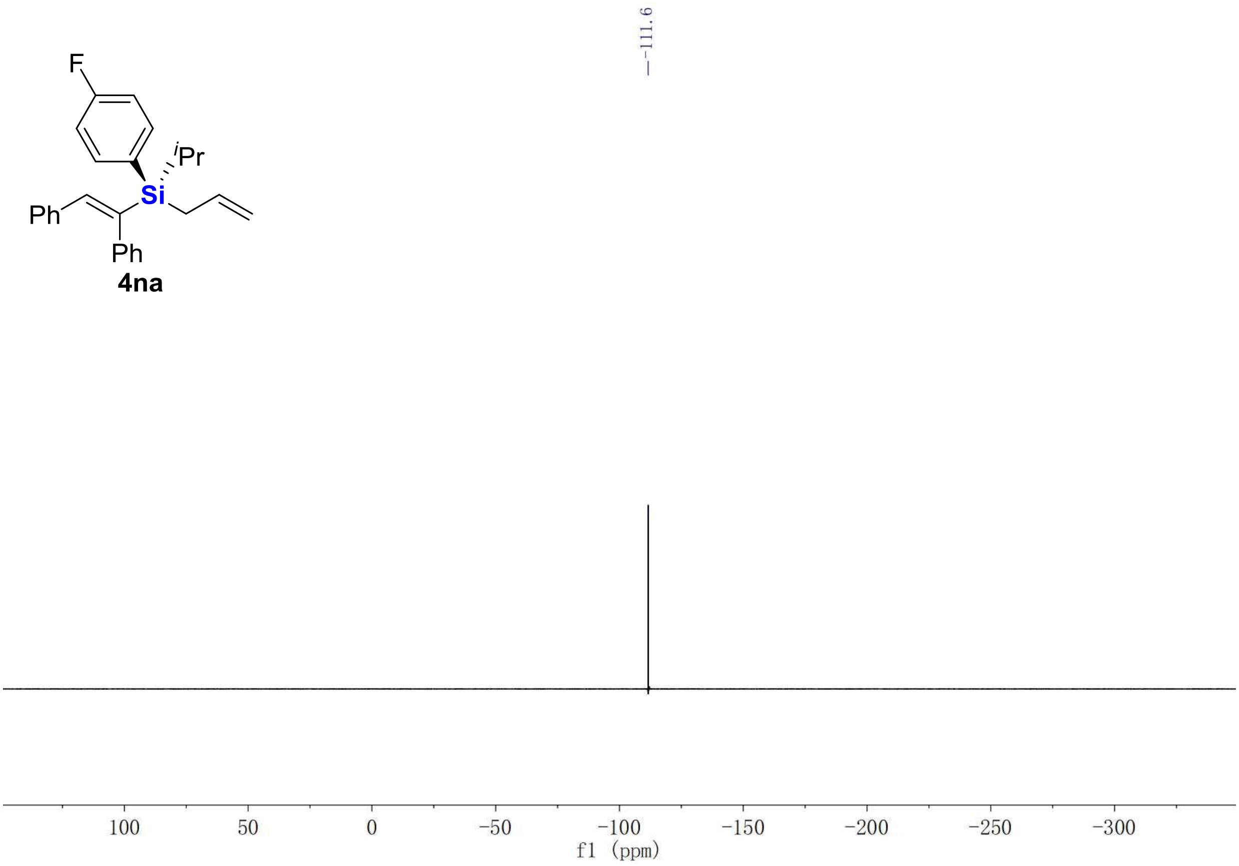

Supplementary Figure 79 ^{1}H, ^{13}C and ^{19}F NMR Spectra for compound 4na

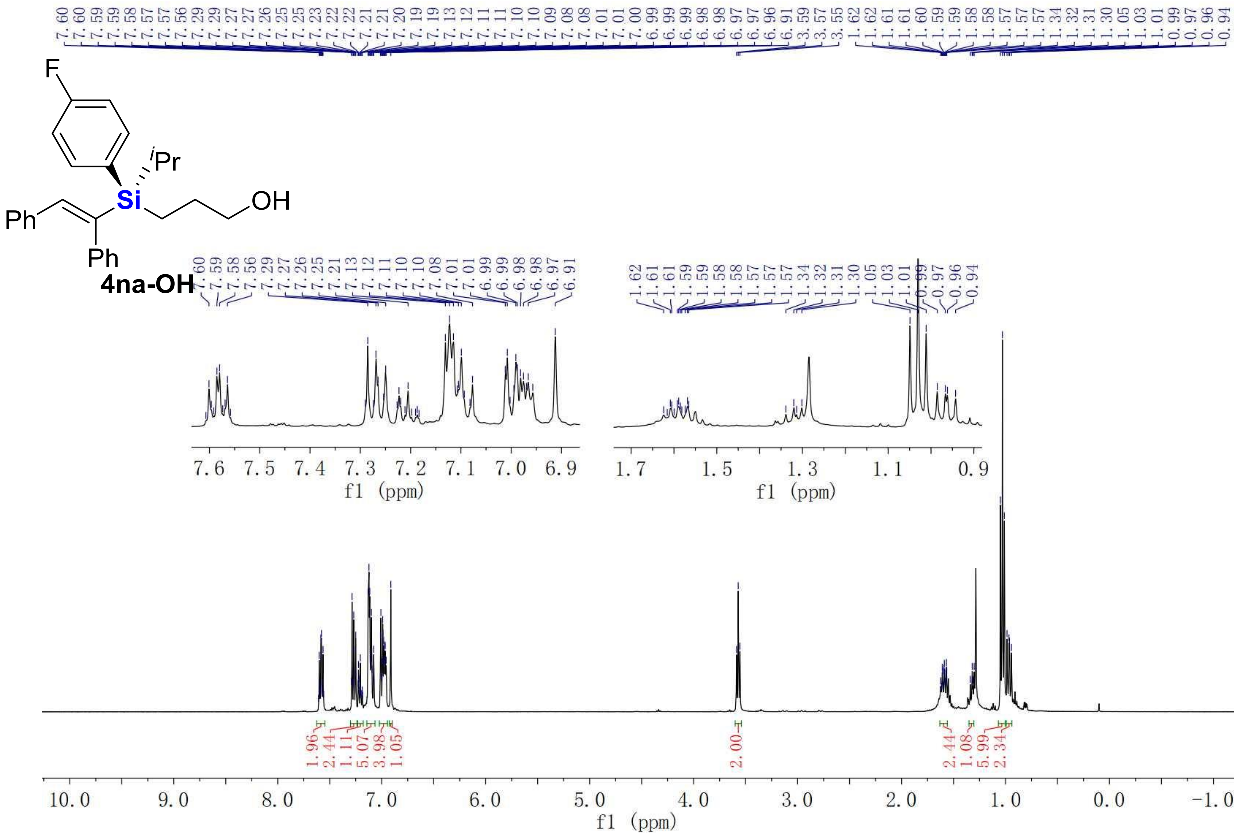

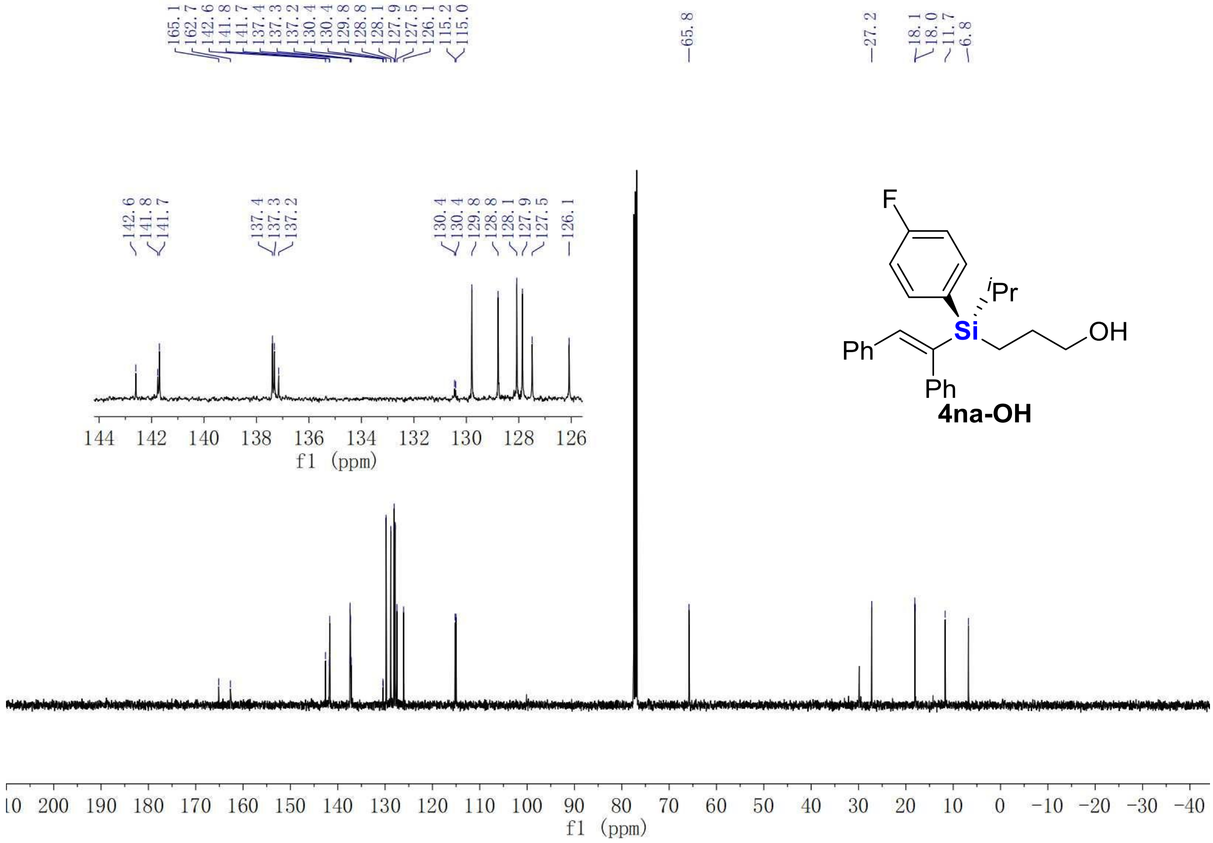

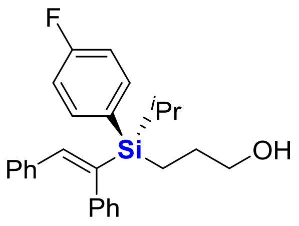
4na-OH

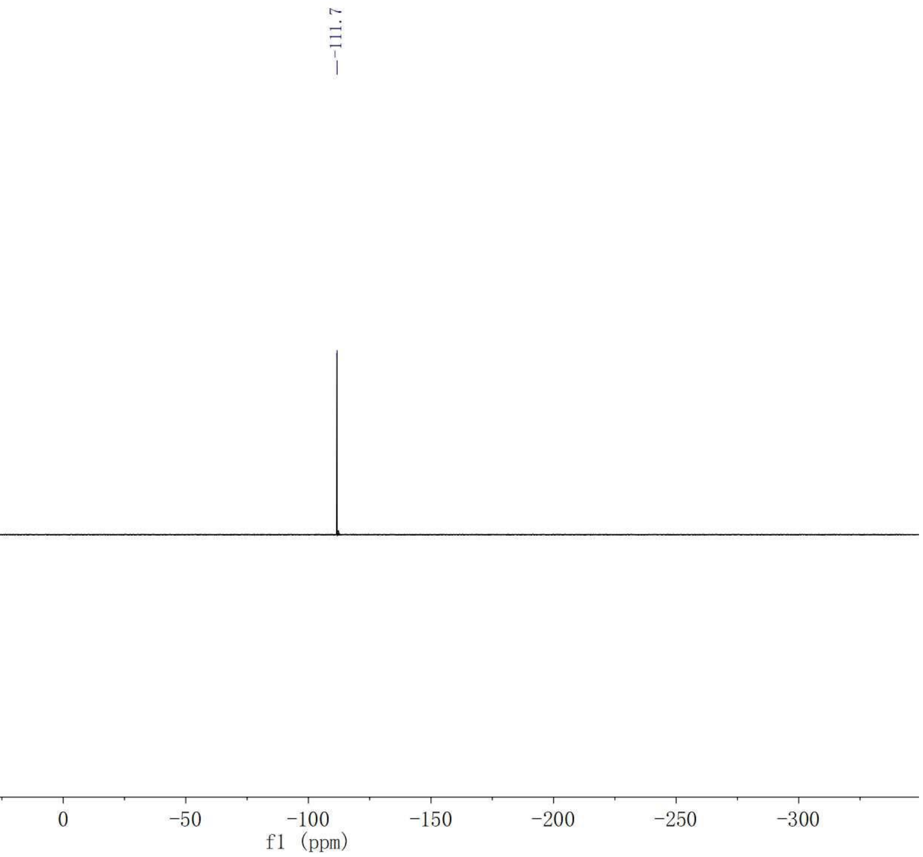

Supplementary Figure 80 ^{1}H, ^{13}C and ^{19}F NMR Spectra for compound 4na-OH

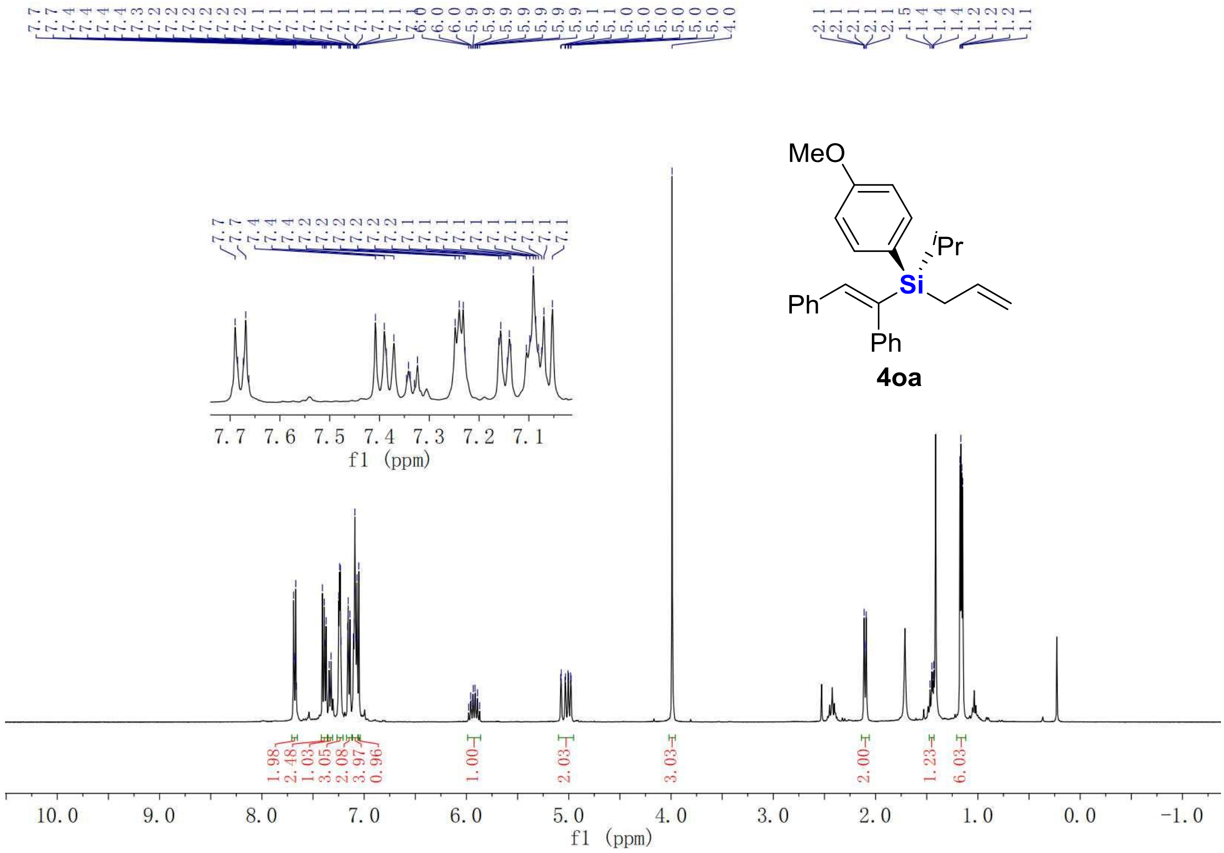

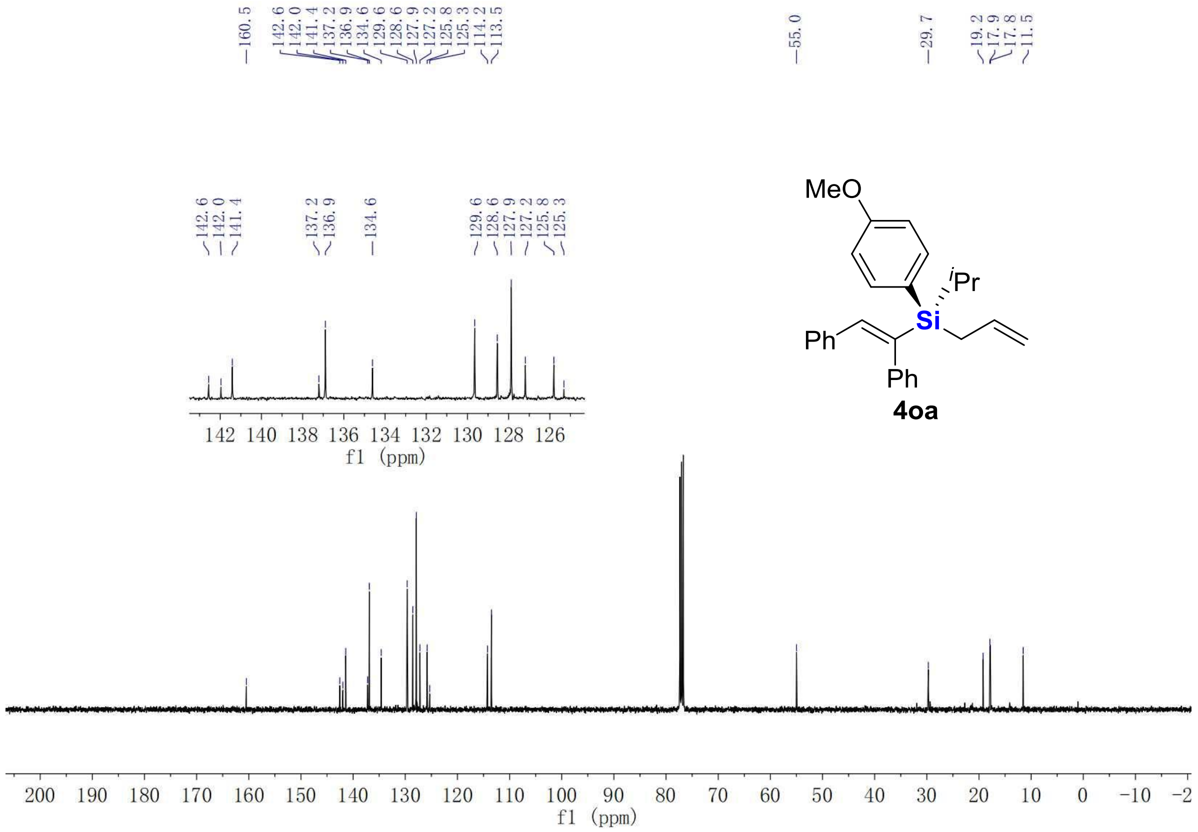

Supplementary Figure 81 ^{1}H and ^{13}C NMR Spectra for compound 4oa

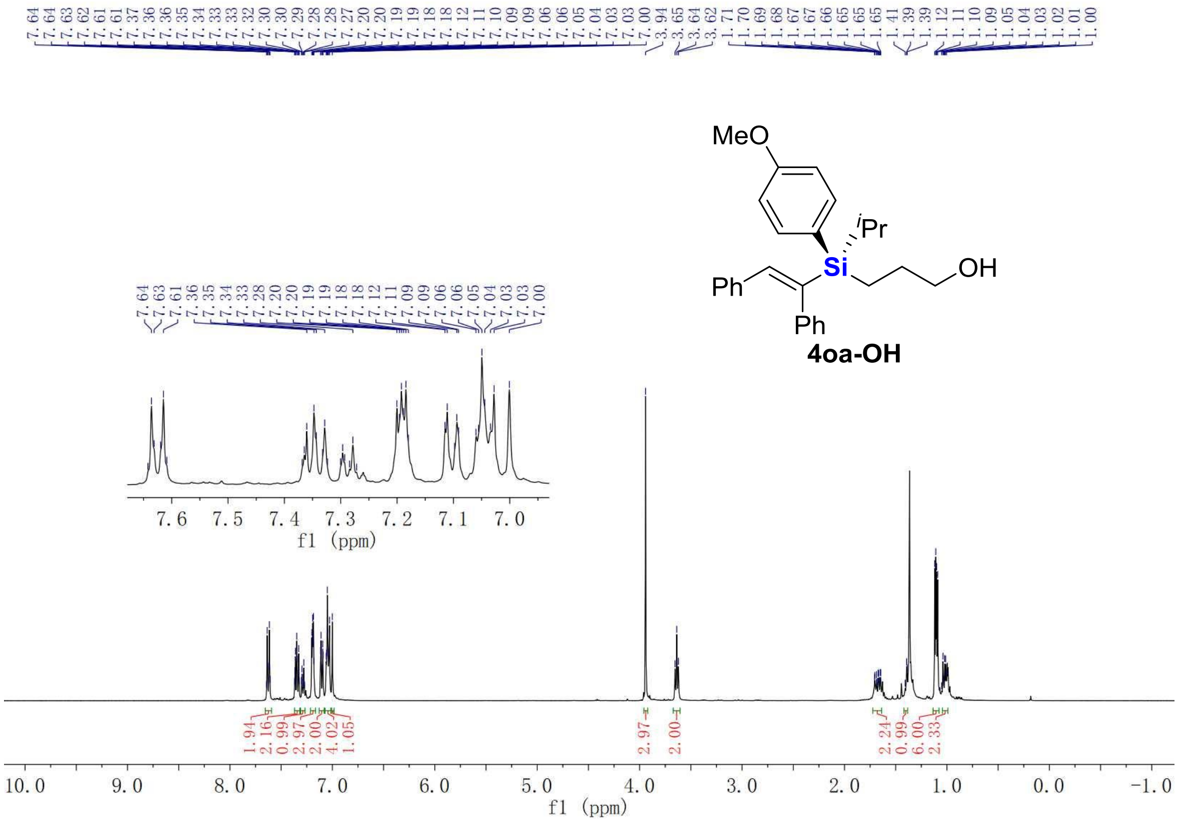

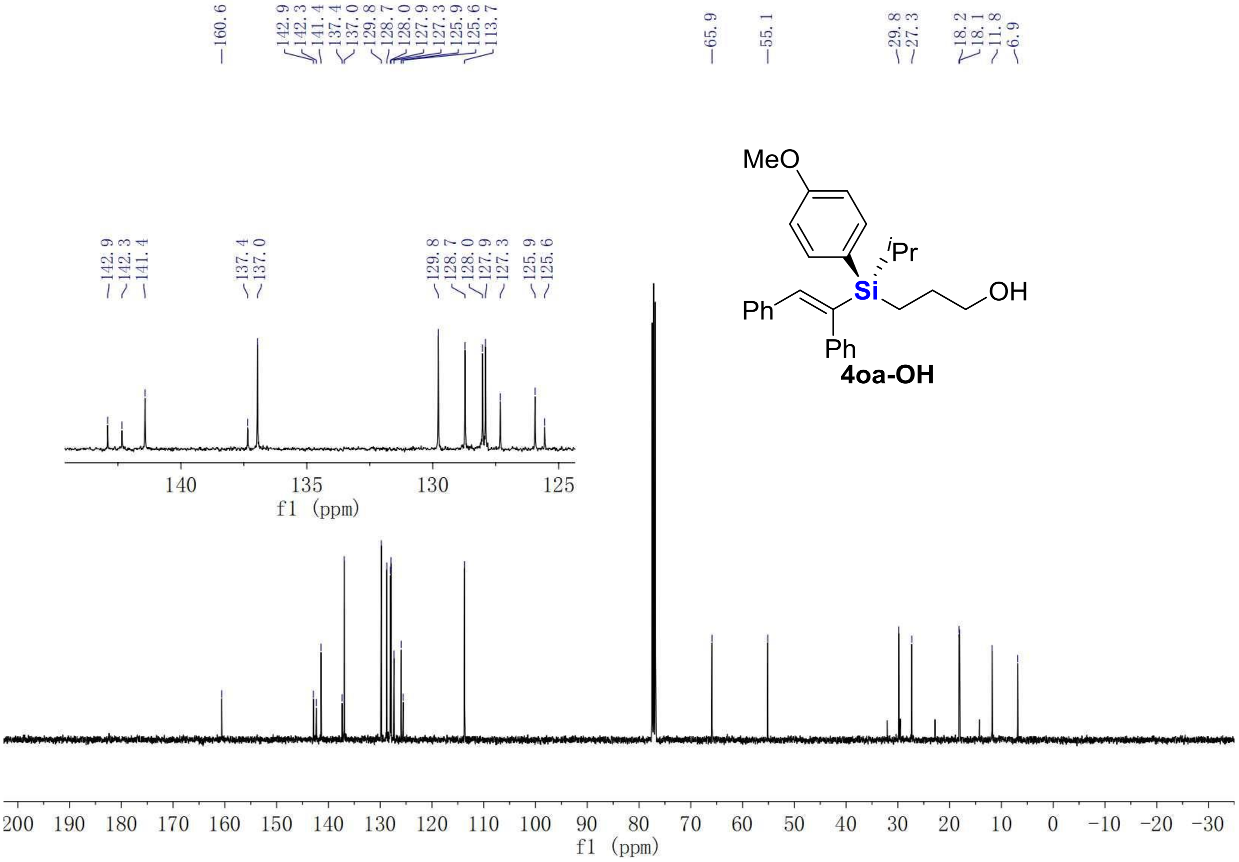

Supplementary Figure 82 ^{1}H and ^{13}C NMR Spectra for compound 4oa-OH

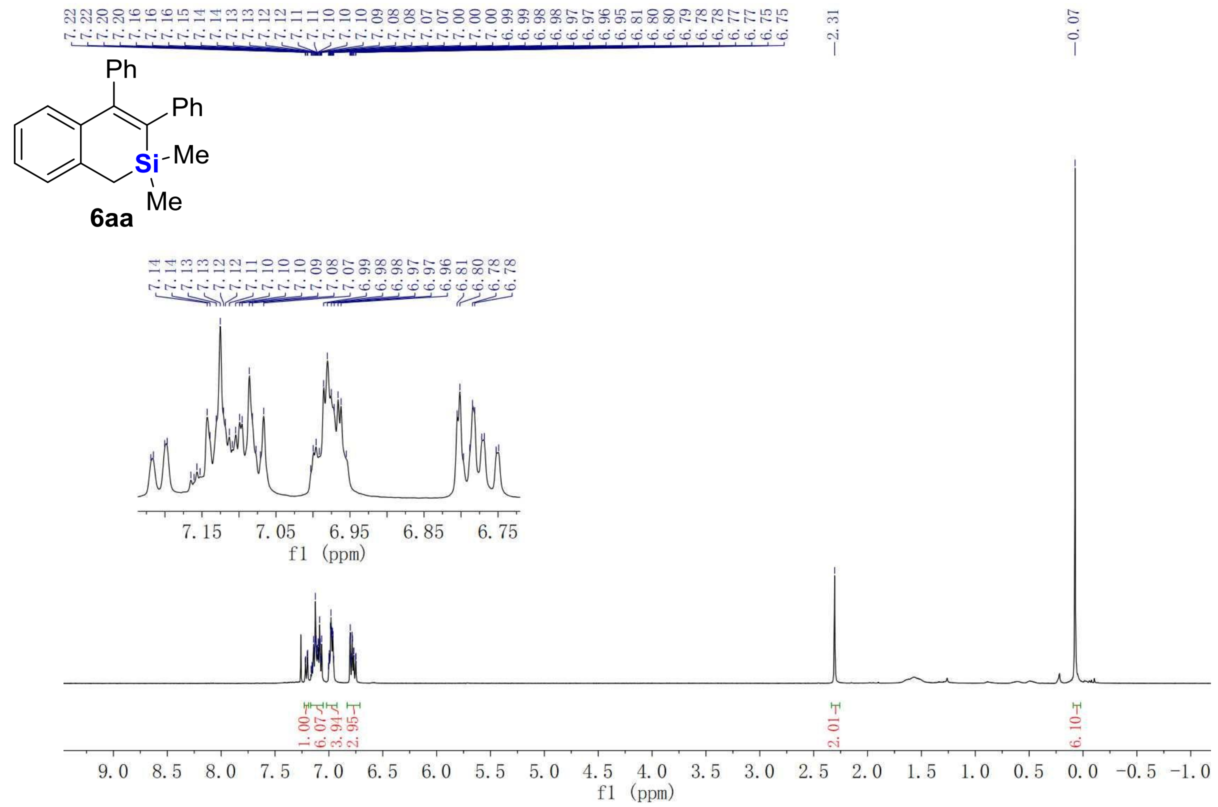

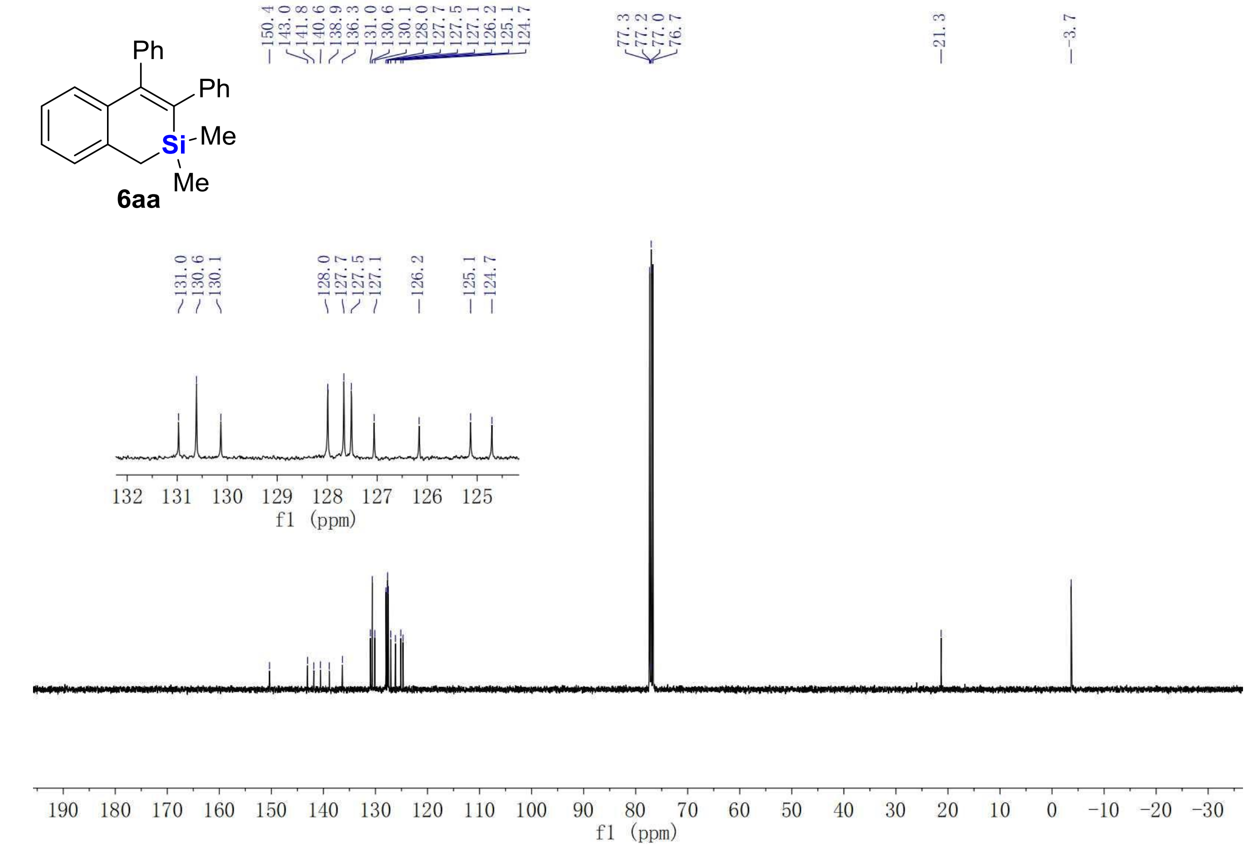

Supplementary Figure 83 ^{1}H and ^{13}C NMR Spectra for compound 6aa

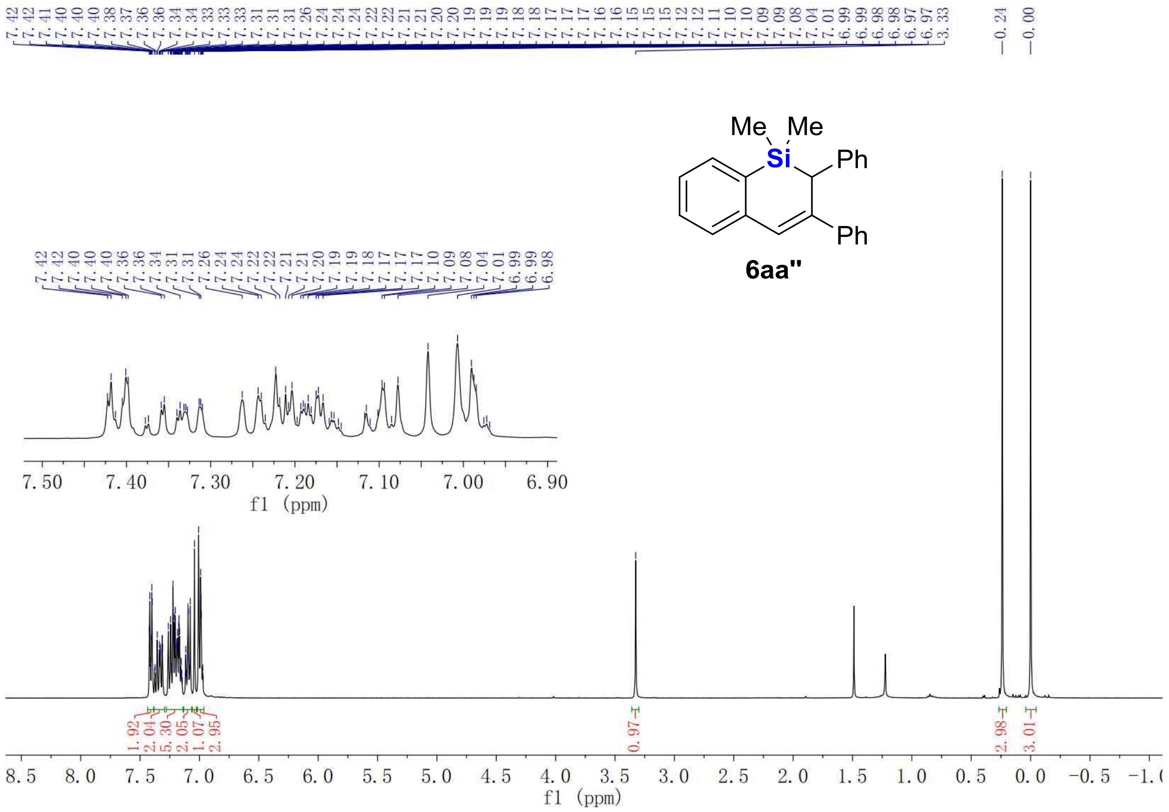

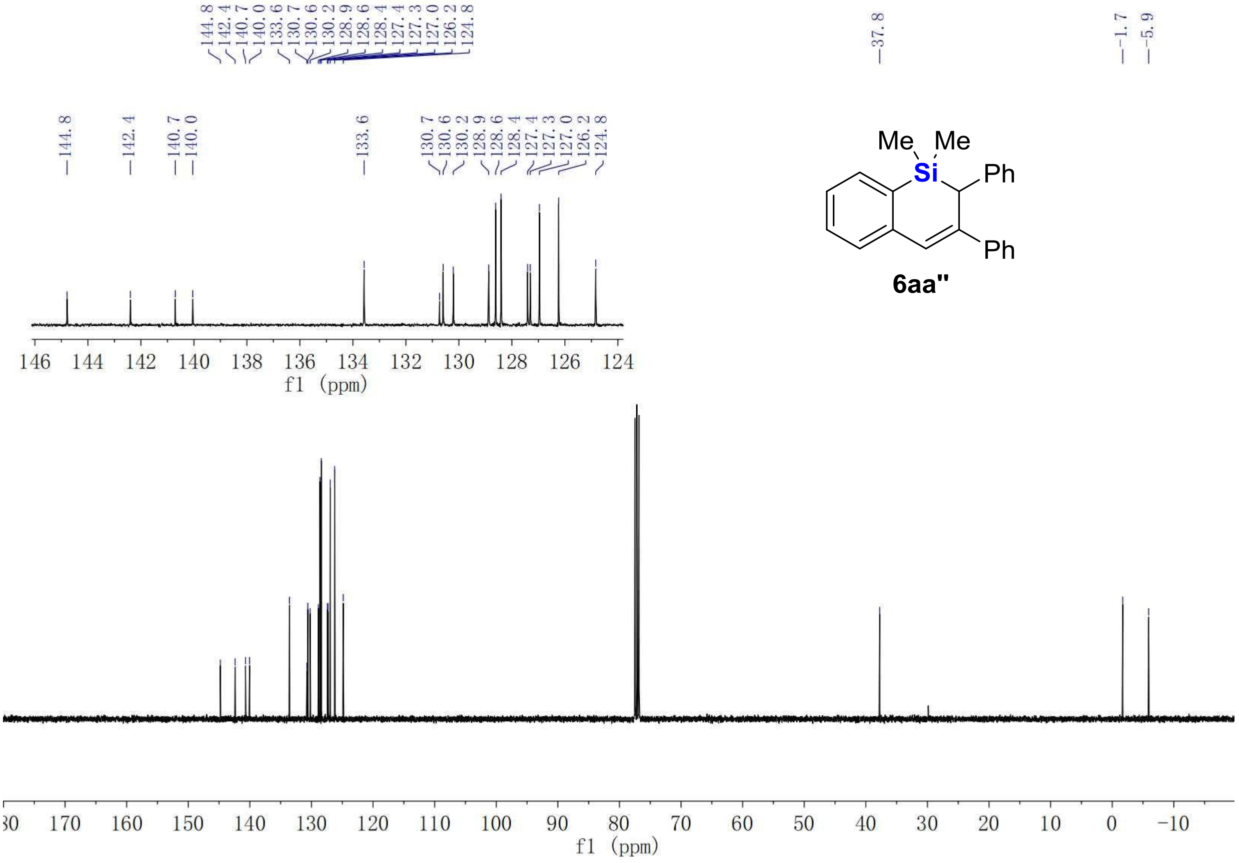

Supplementary Figure 84 ^{1}H and ^{13}C NMR Spectra for compound 6aa''

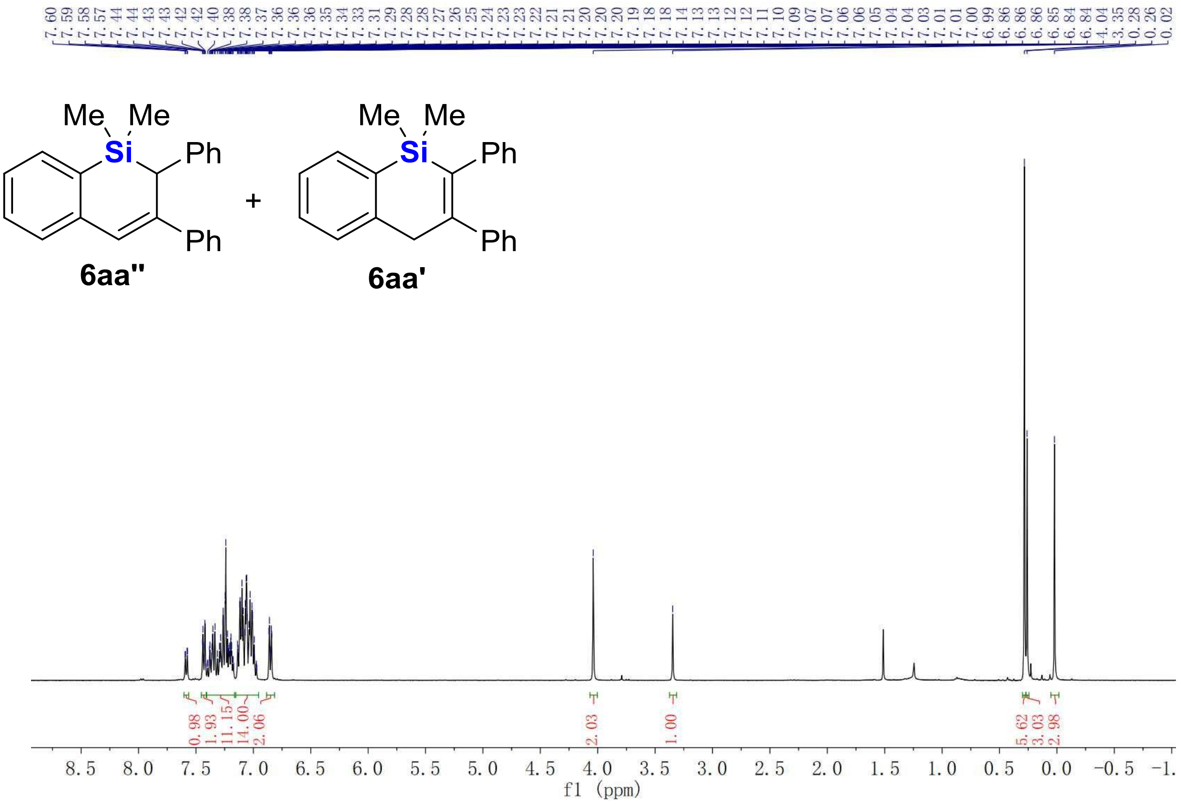

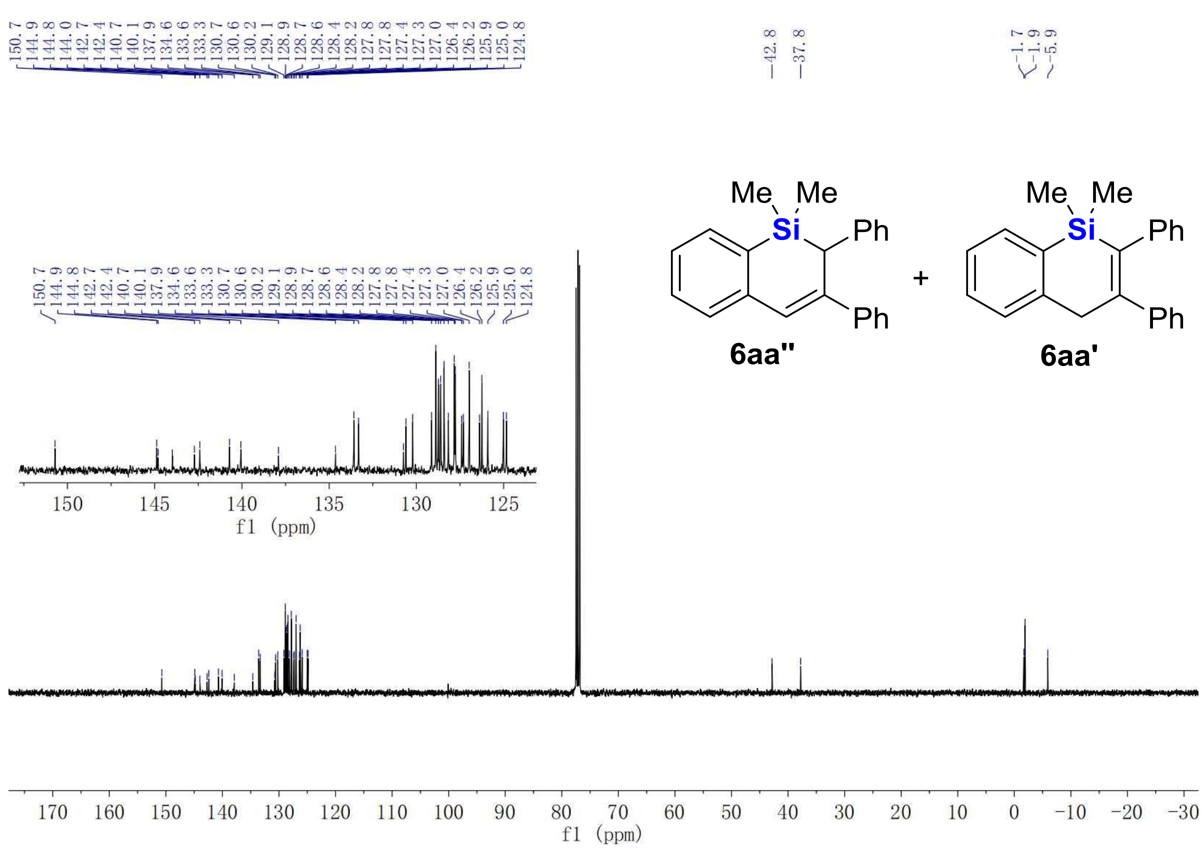

Supplementary Figure 85 ^{1}H and ^{13}C NMR Spectra for mixture of 6aa’ and 6aa’’

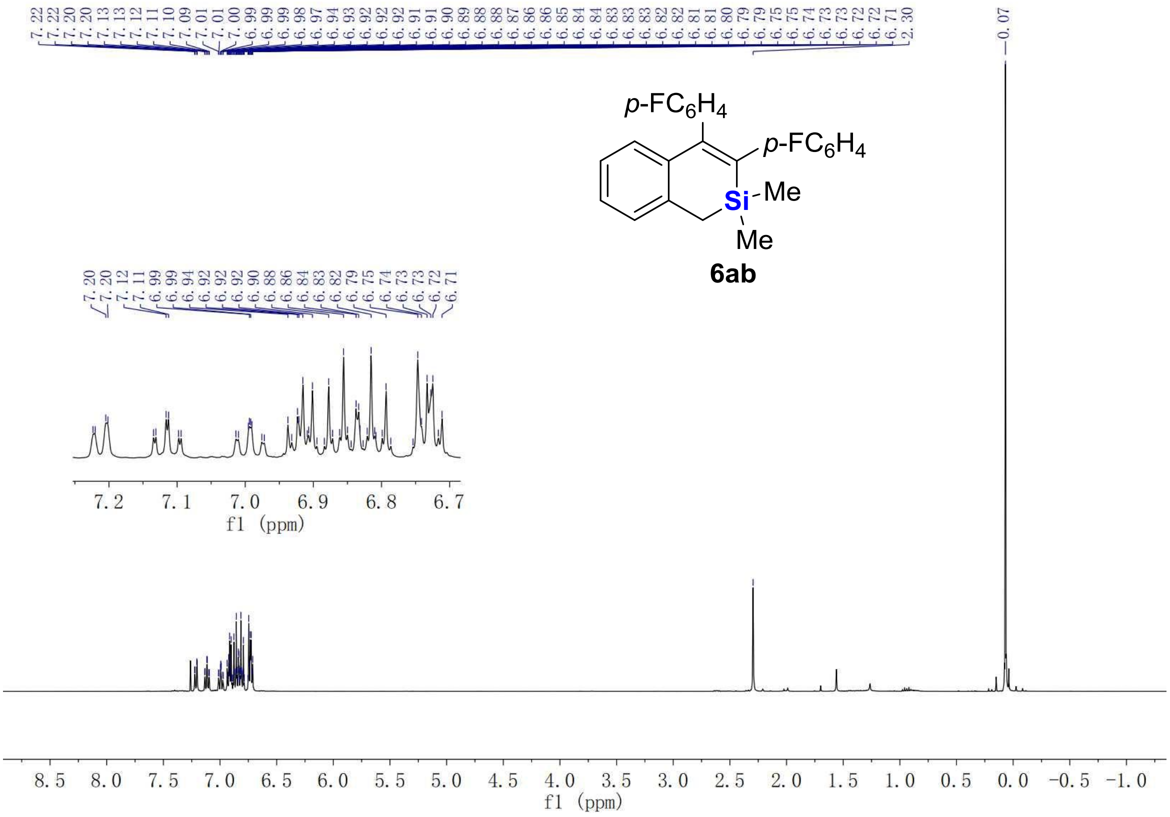
7.22 7.22 7.20 7.20 7.13 7.12 7.12 7.11 7.10 7.09 7.01 7.00 6.99 6.99 6.98 6.97 6.94 6.93 6.92 6.92 6.91 6.91 6.90 6.89 6.88 6.88 6.87 6.86 6.86 6.85 6.84 6.84 6.83 6.83 6.83 6.82 6.82 6.81 6.81 6.80 6.79 6.75 6.75 6.74 6.73 6.73 6.72 6.72 6.71 2.30
0.07
p-FC6H4
p-FC6H4
Si
Me
Me
6ab
7.20 7.20 7.12 7.11 6.99 6.99 6.94 6.92 6.92 6.92 6.90 6.88 6.86 6.84 6.83 6.82 6.79 6.75 6.74 6.73 6.73 6.72 6.71
7.2 7.1 7.0 6.9 6.8 6.7
f1 (ppm)
8.5 8.0 7.5 7.0 6.5 6.0 5.5 5.0 4.5 4.0 3.5 3.0 2.5 2.0 1.5 1.0 0.5 0.0 −0.5 −1.0
f1 (ppm)

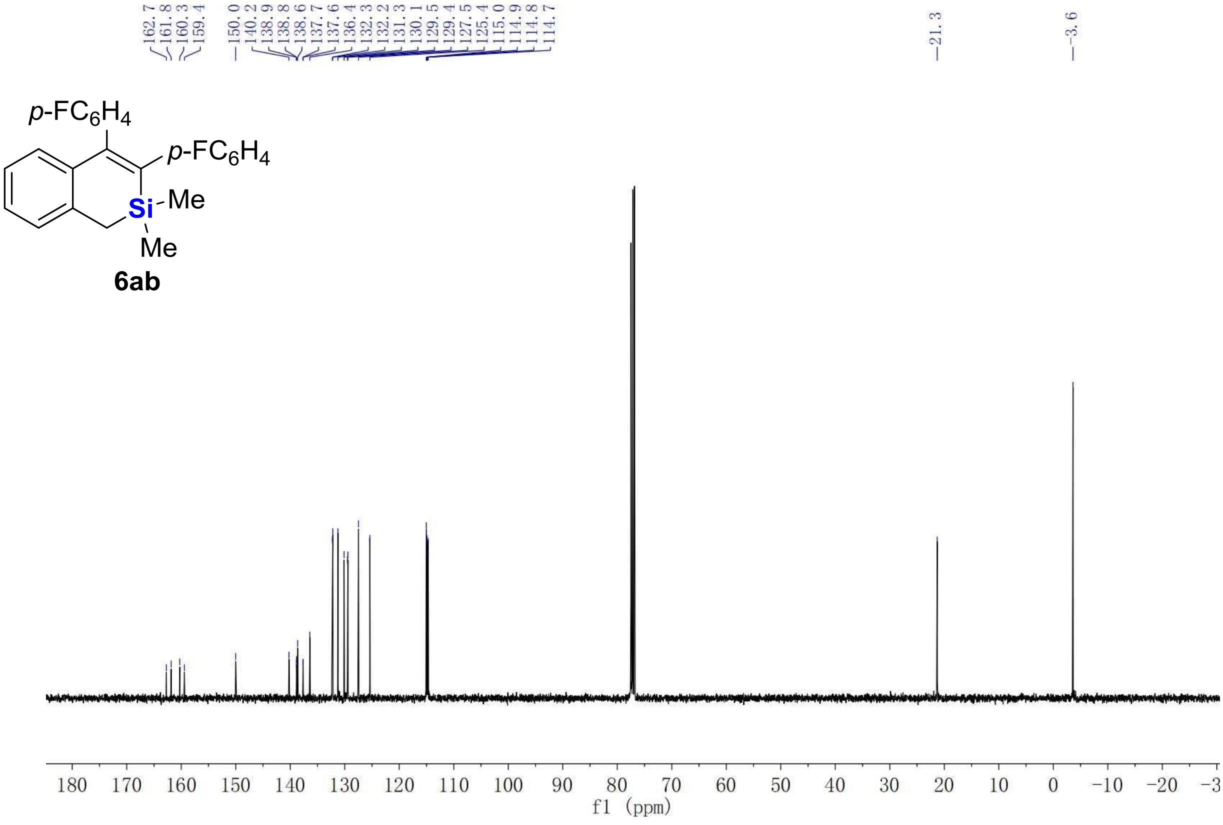
162.7 161.8 160.3 159.4 150.0 140.2 138.9 138.8 138.6 137.7 137.6 136.4 132.3 132.2 131.3 130.1 129.5 129.4 127.5 125.4 115.0 114.9 114.8 114.7
21.3
−3.6
p-FC6H4
p-FC6H4
Si
Me
Me
6ab
180 170 160 150 140 130 120 110 100 90 80 70 60 50 40 30 20 10 0 −10 −20 −3
f1 (ppm)

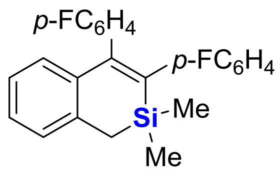

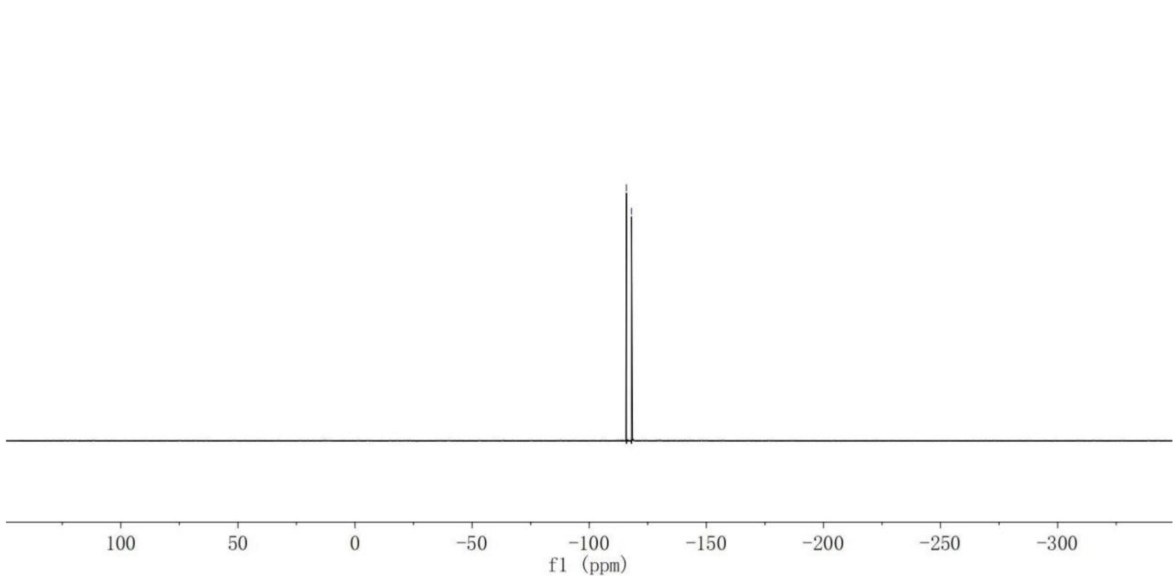

Supplementary Figure 86 ^{1}H, ^{13}C and ^{19}F NMR Spectra for compound 6ab

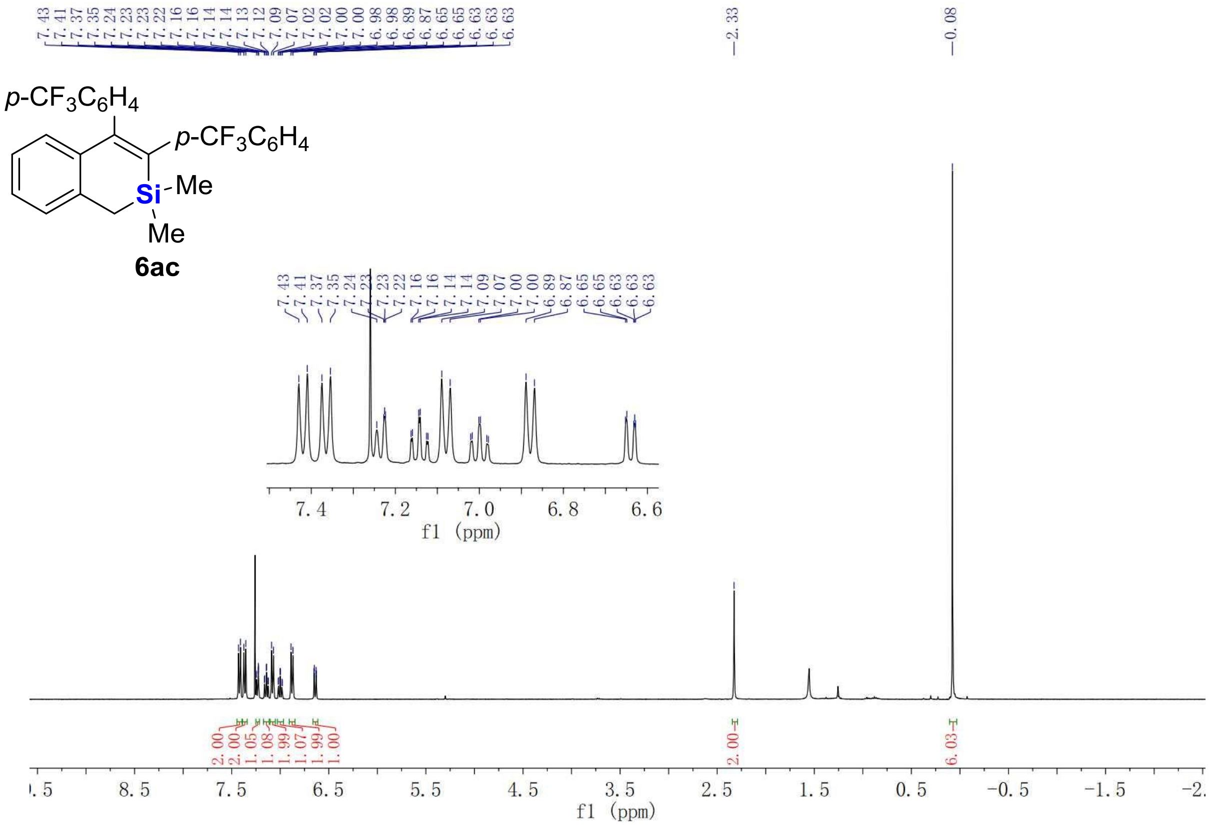
p-CF3C6H4
p-CF3C6H4
Si
Me
Me
6ac
f1 (ppm)

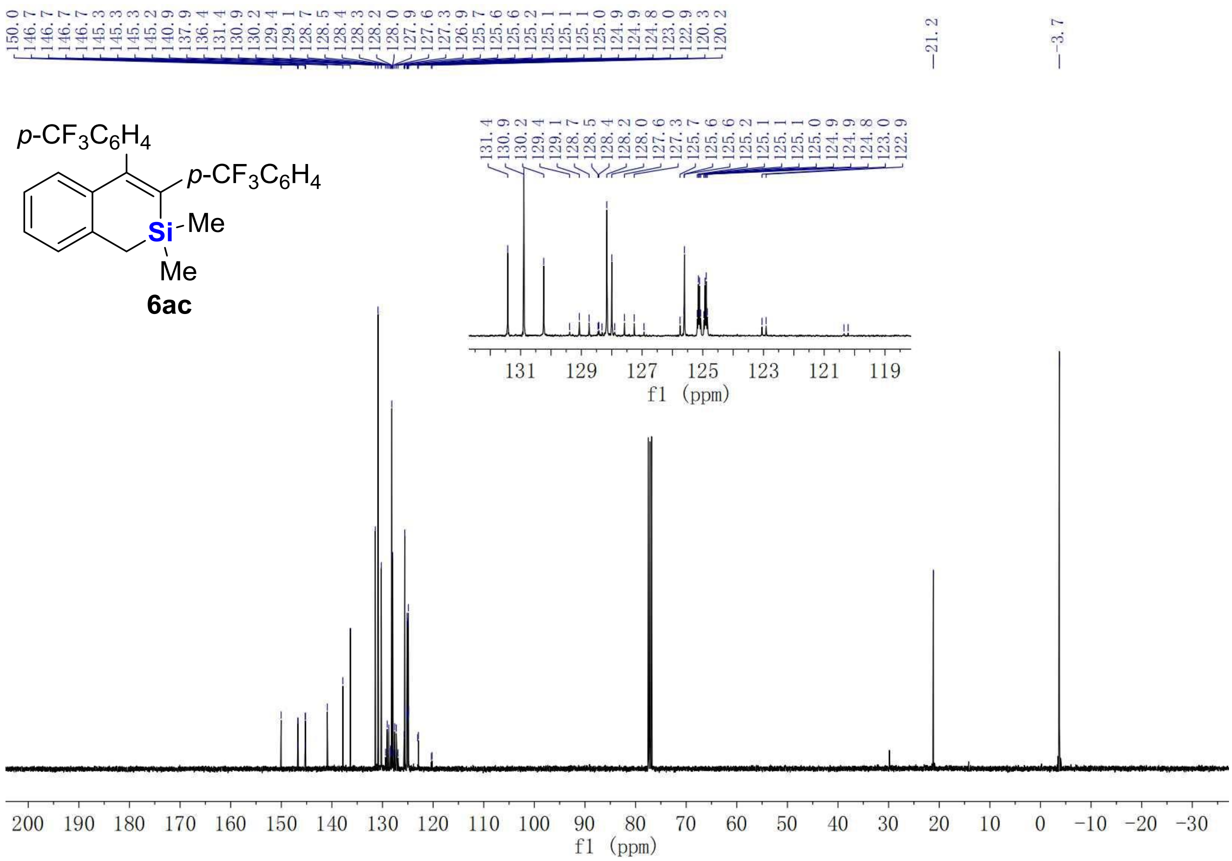
p-CF3C6H4
p-CF3C6H4
Si
Me
Me
6ac
f1 (ppm)

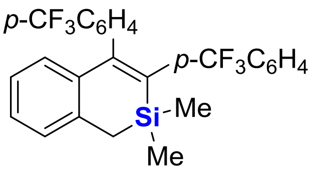

6ac

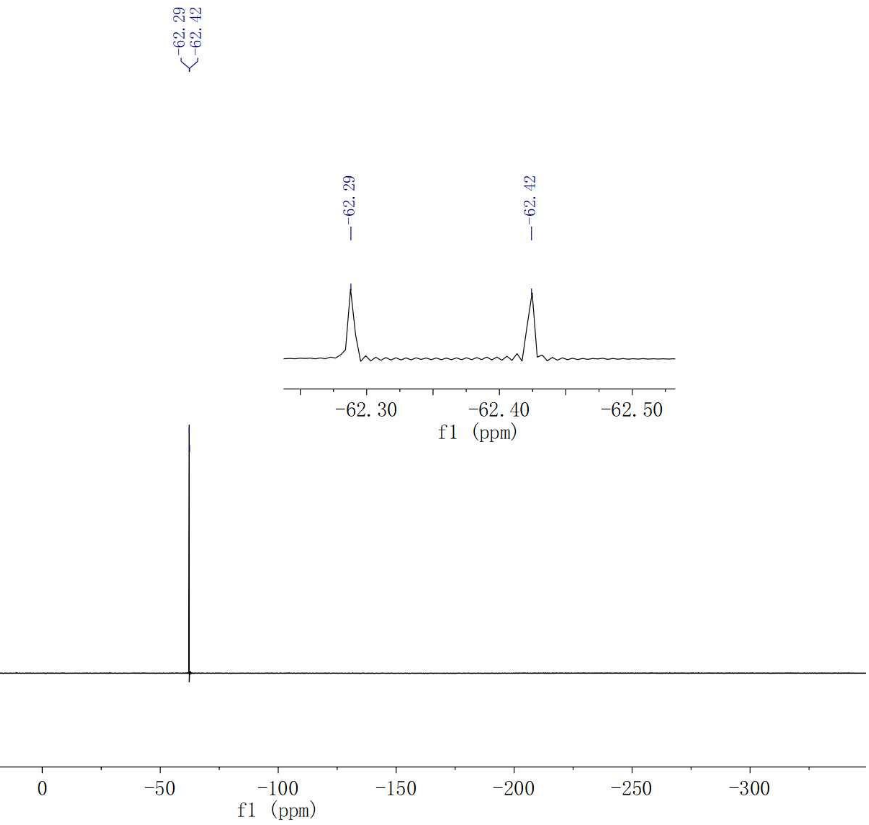

Supplementary Figure 87 ^{1}H, ^{13}C and ^{19}F NMR Spectra for compound 6ac

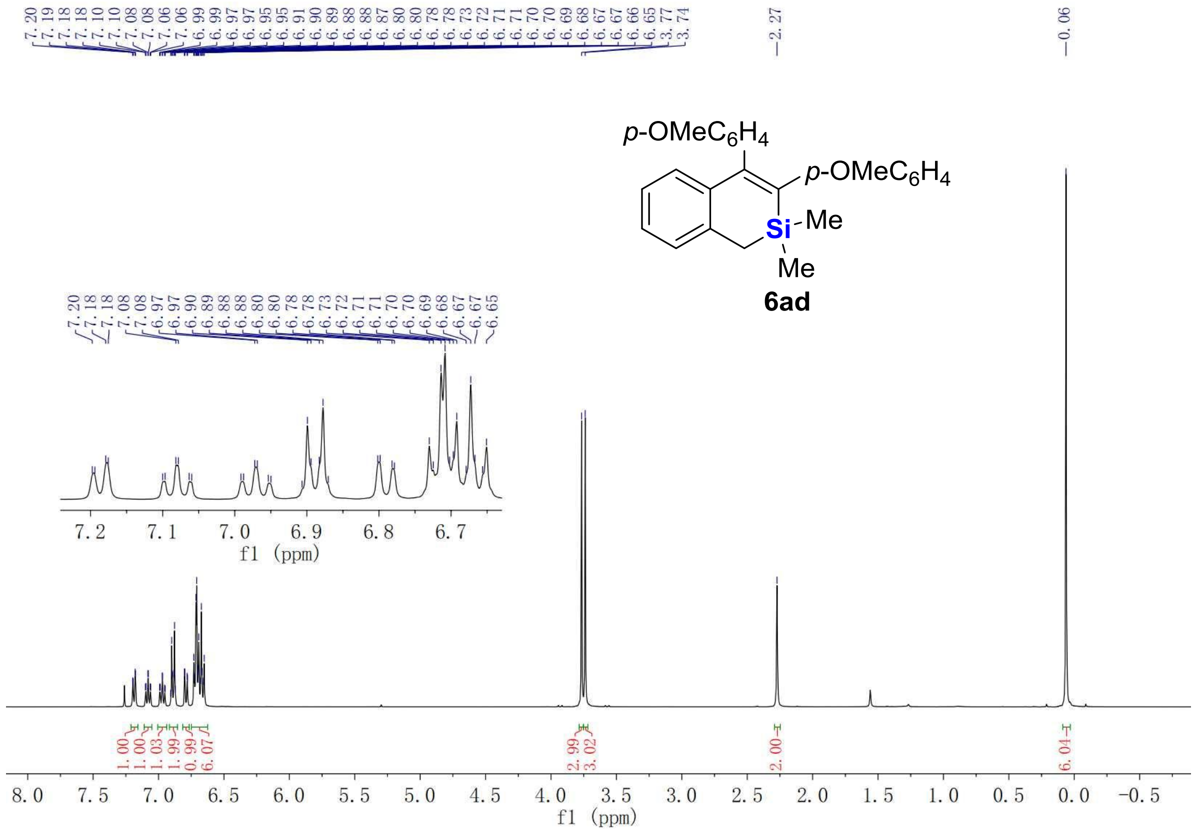

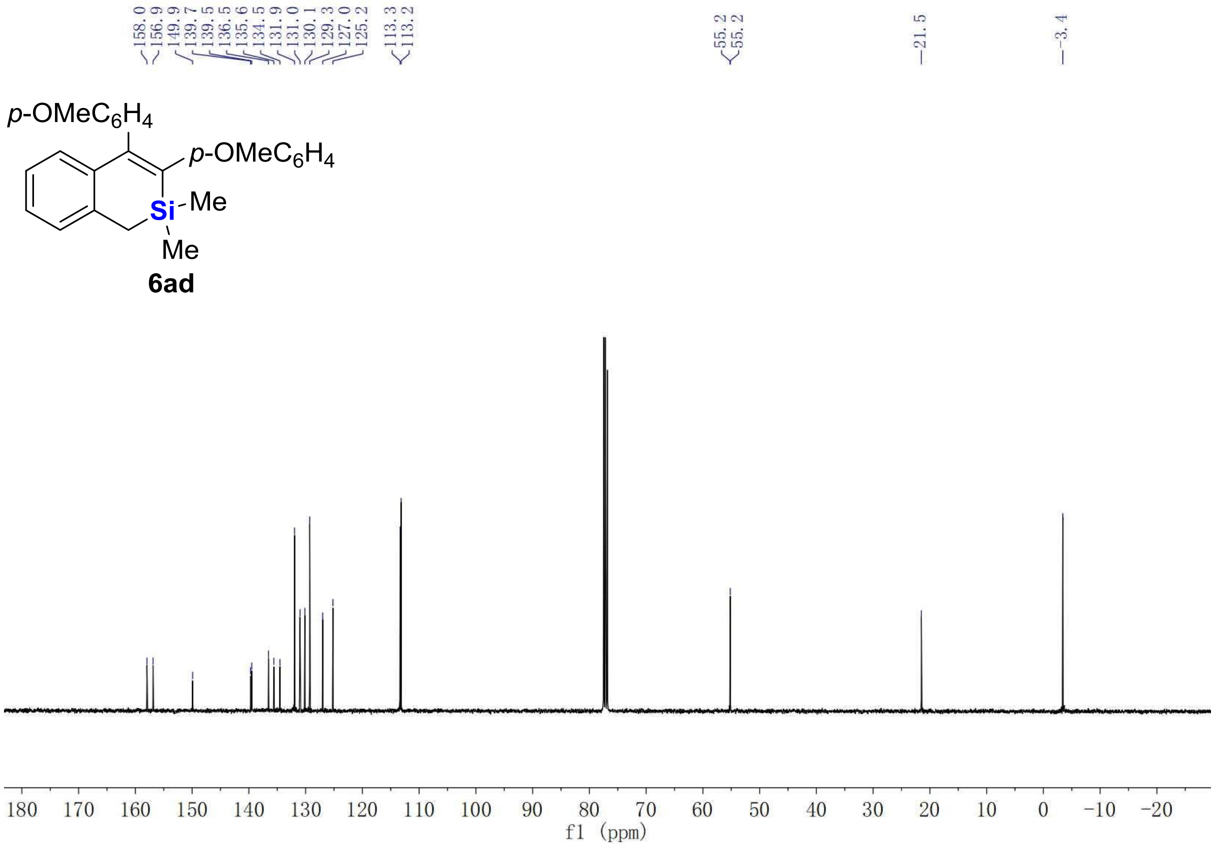

Supplementary Figure 88 ^{1}H and ^{13}C NMR Spectra for compound 6ad

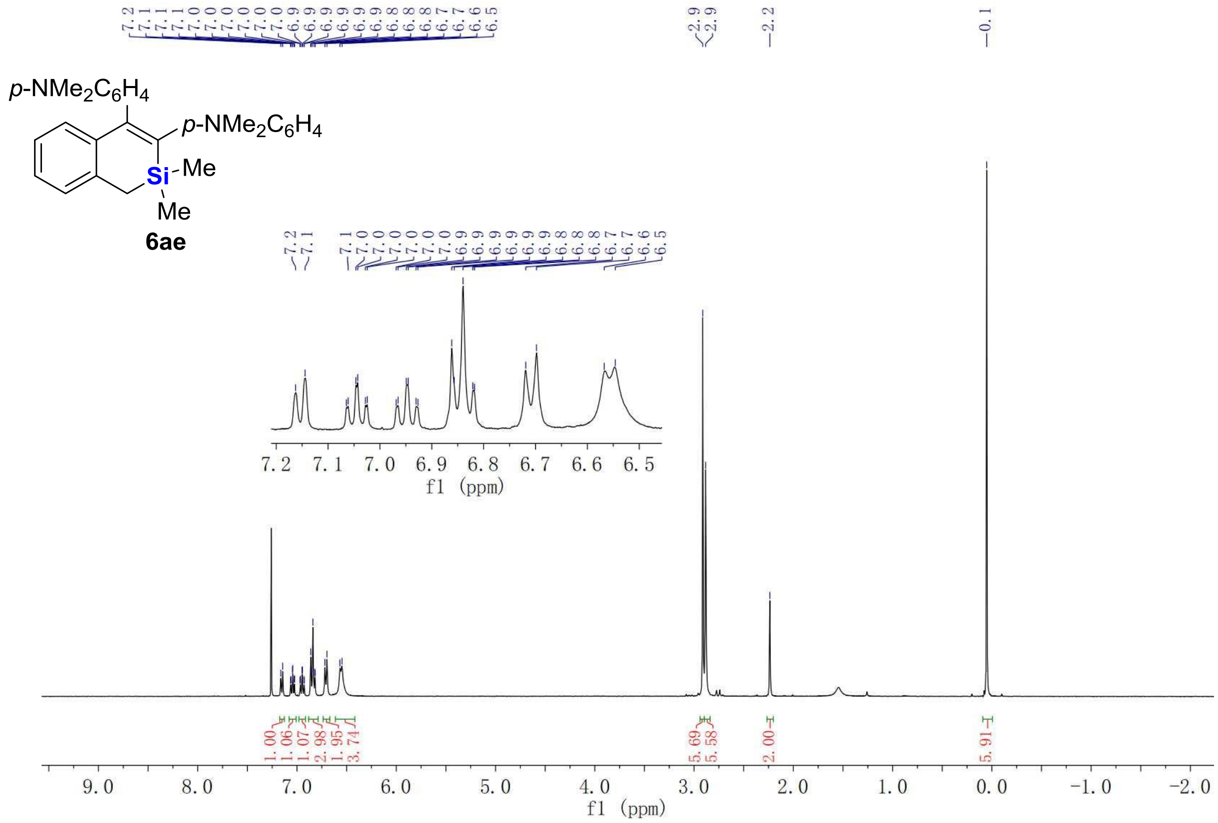

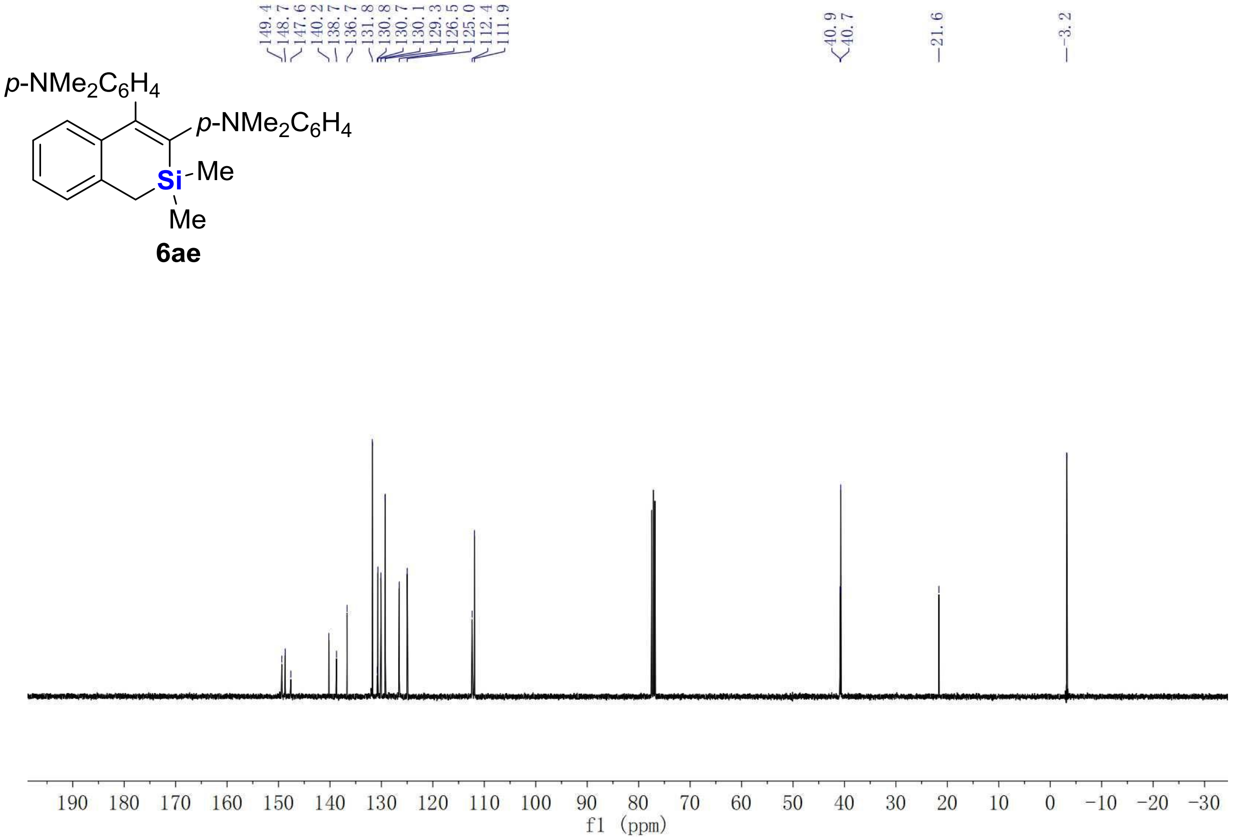

Supplementary Figure 89 ^{1}H and ^{13}C NMR Spectra for compound 6ae

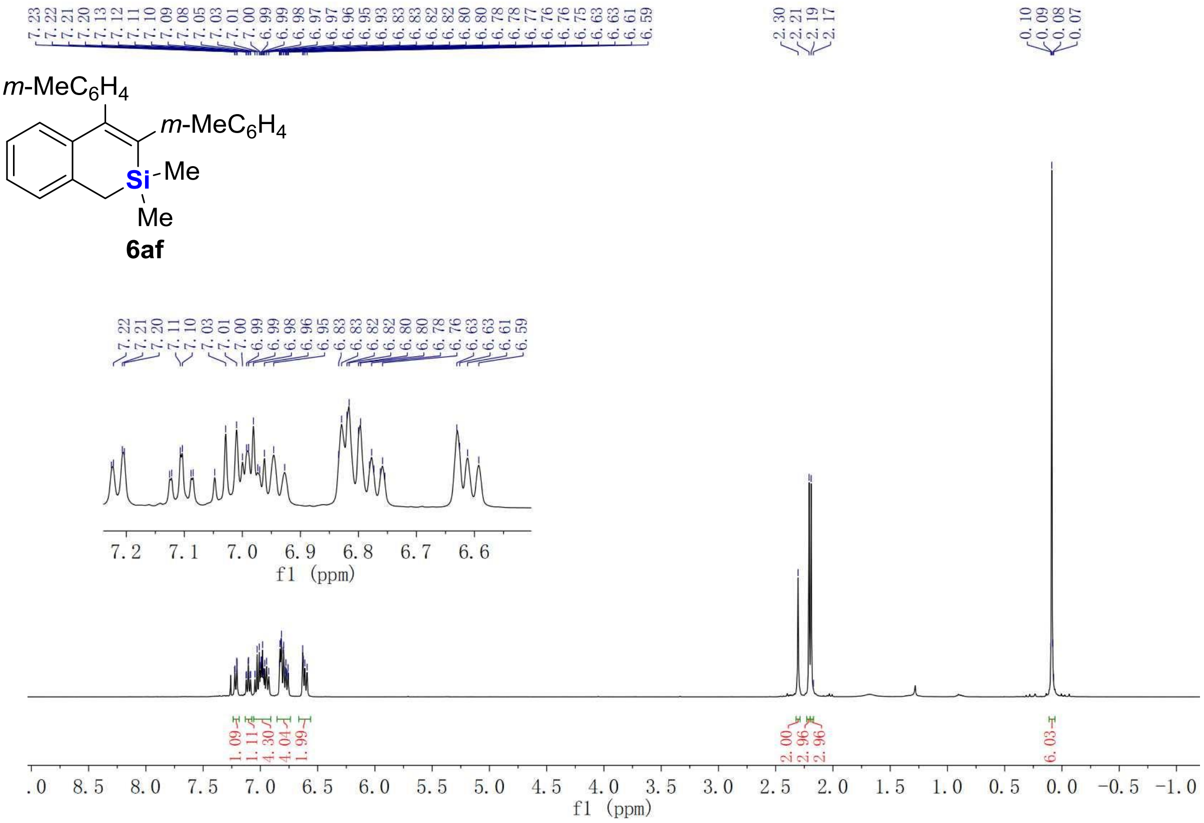

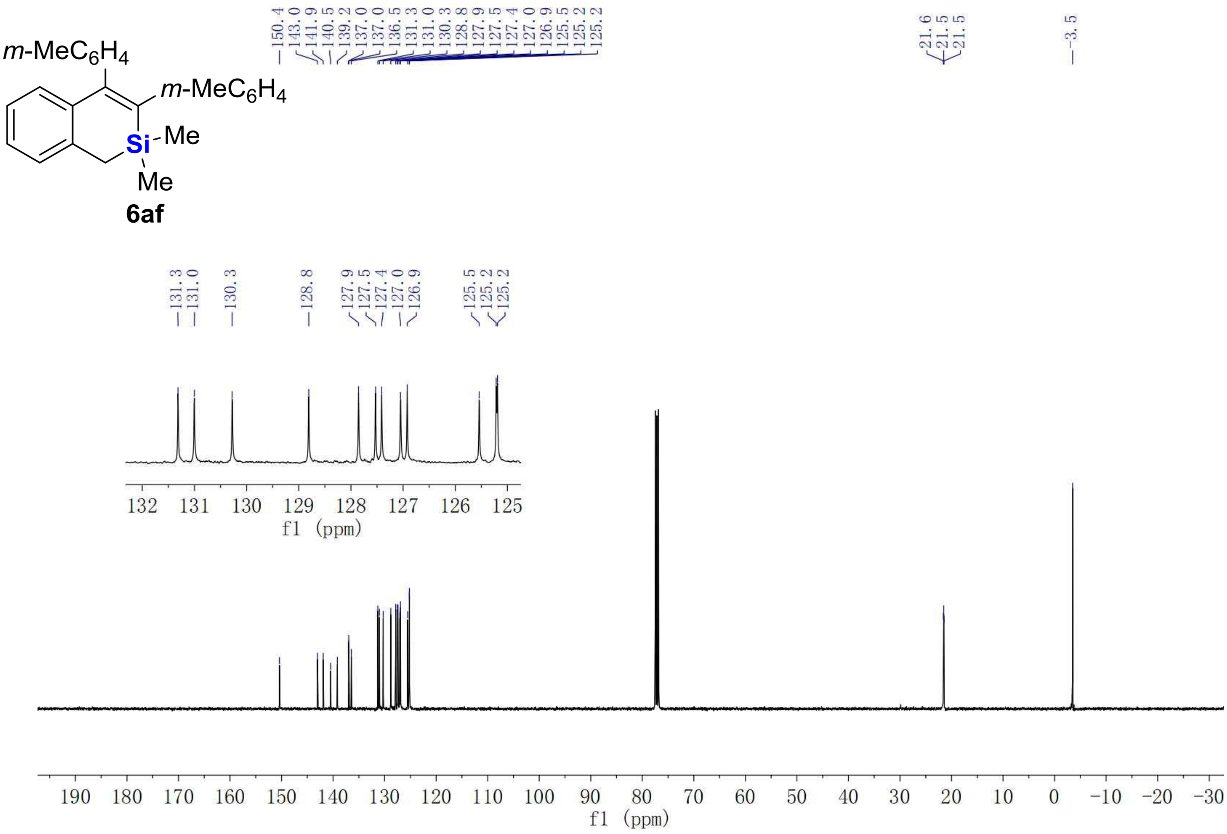

Supplementary Figure 90 ^{1}H and ^{13}C NMR Spectra for compound 6af

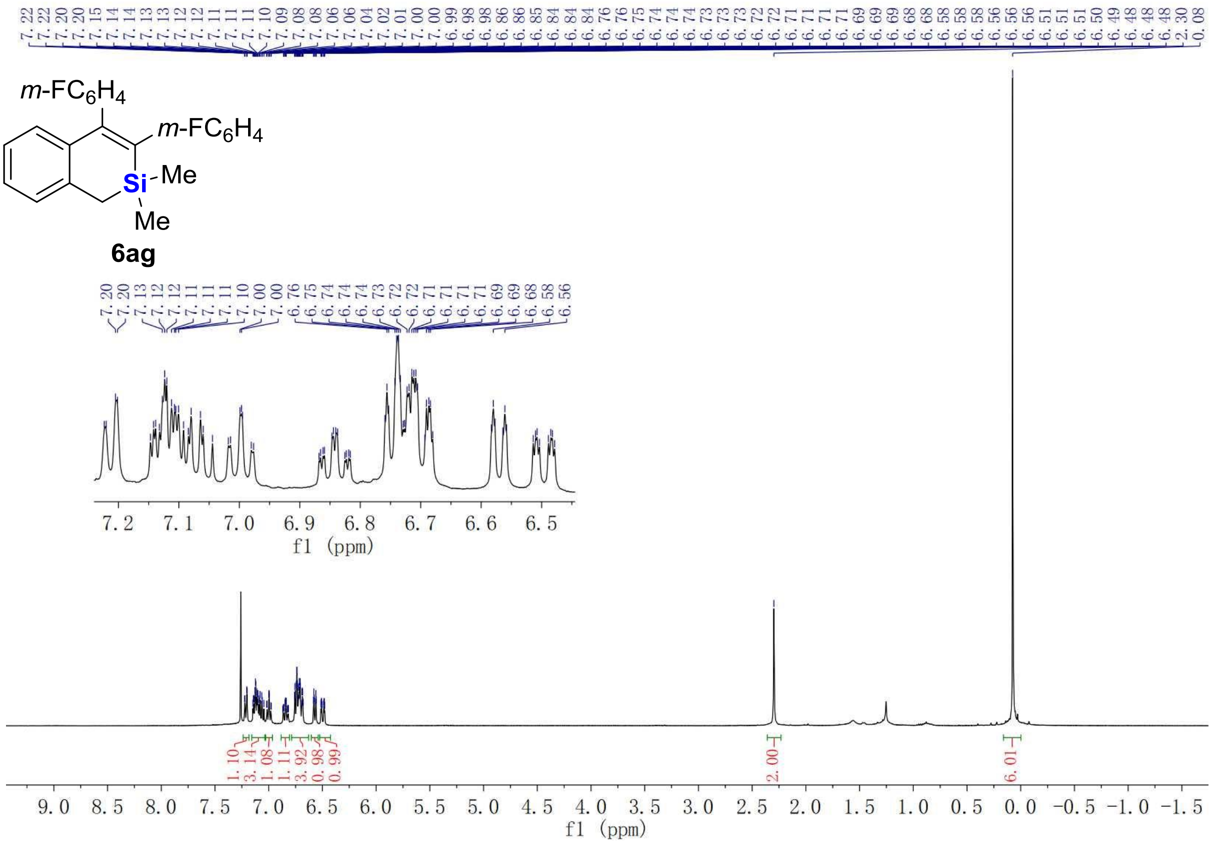
m-FC6H4
m-FC6H4
Si
Me
Me
6ag
f1 (ppm)
f1 (ppm)

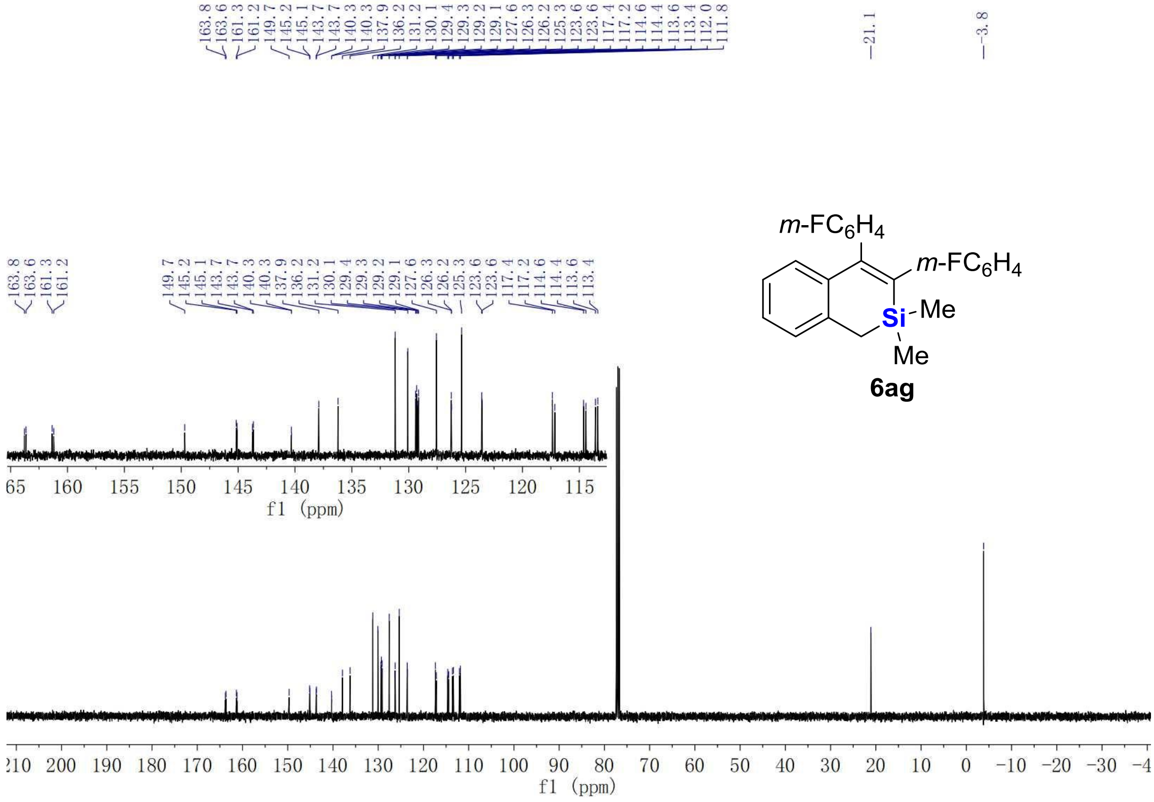
m-FC6H4
m-FC6H4
Si
Me
Me
6ag
f1 (ppm)
f1 (ppm)

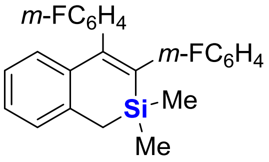

6ag

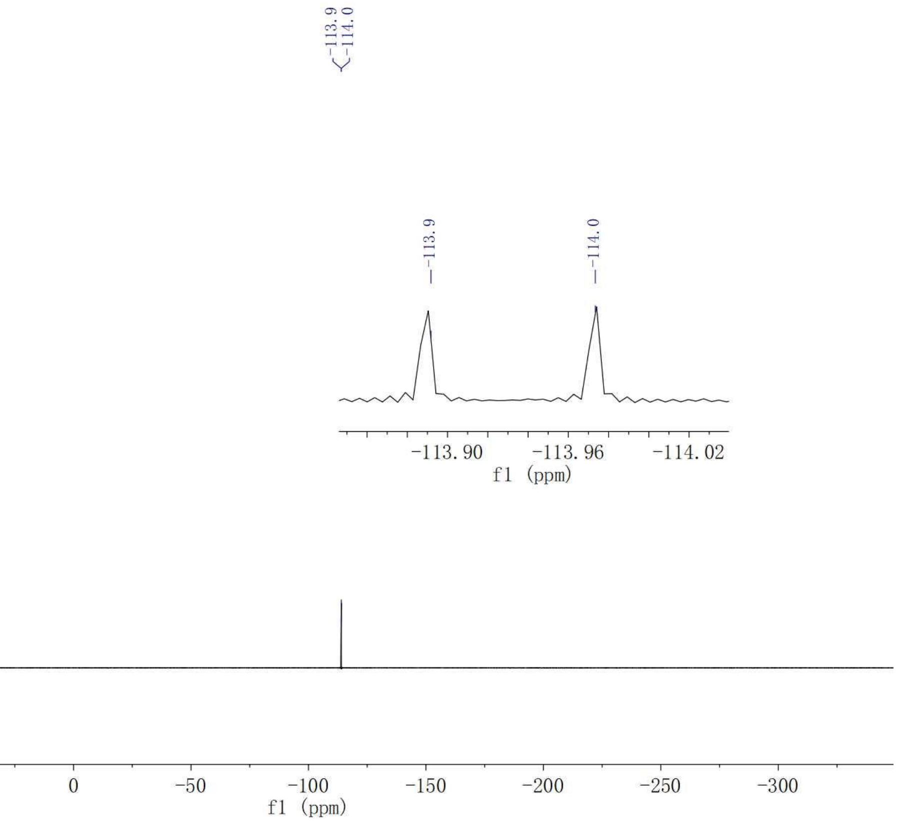

Supplementary Figure 91 ^{1}H, ^{13}C and ^{19}F NMR Spectra for compound 6ag

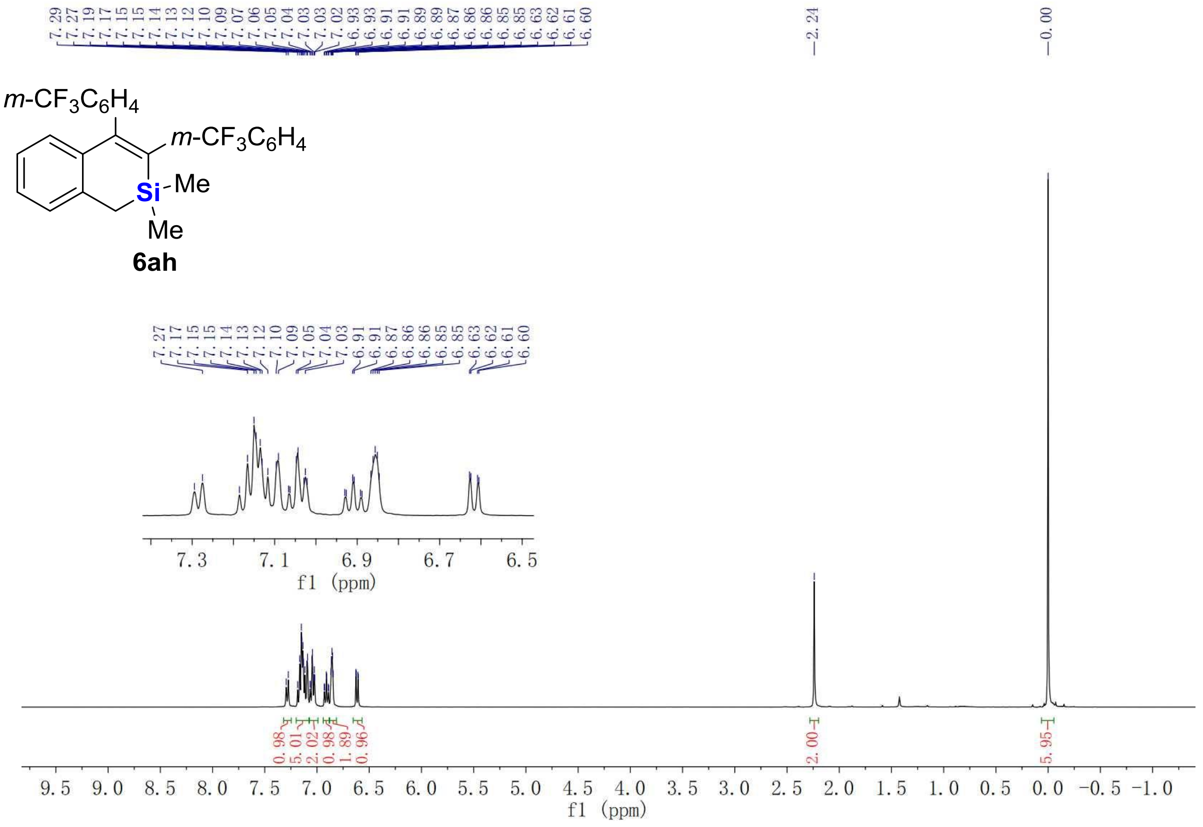
7.29 7.27 7.19 7.17 7.15 7.15 7.14 7.13 7.12 7.10 7.09 7.07 7.06 7.05 7.04 7.03 7.03 7.02 6.93 6.91 6.91 6.89 6.87 6.86 6.86 6.85 6.63 6.62 6.61 6.60
2.24
0.00
m-CF3C6H4
m-CF3C6H4
Si
Me
Me
6ah
7.27 7.17 7.15 7.15 7.14 7.13 7.12 7.10 7.09 7.05 7.04 7.03 6.91 6.91 6.87 6.86 6.86 6.85 6.63 6.62 6.61 6.60
7.3 7.1 6.9 6.7 6.5
f1 (ppm)
0.98 5.01 2.02 0.98 1.89 0.96
2.00
5.95
9.5 9.0 8.5 8.0 7.5 7.0 6.5 6.0 5.5 5.0 4.5 4.0 3.5 3.0 2.5 2.0 1.5 1.0 0.5 0.0 -0.5 -1.0
f1 (ppm)

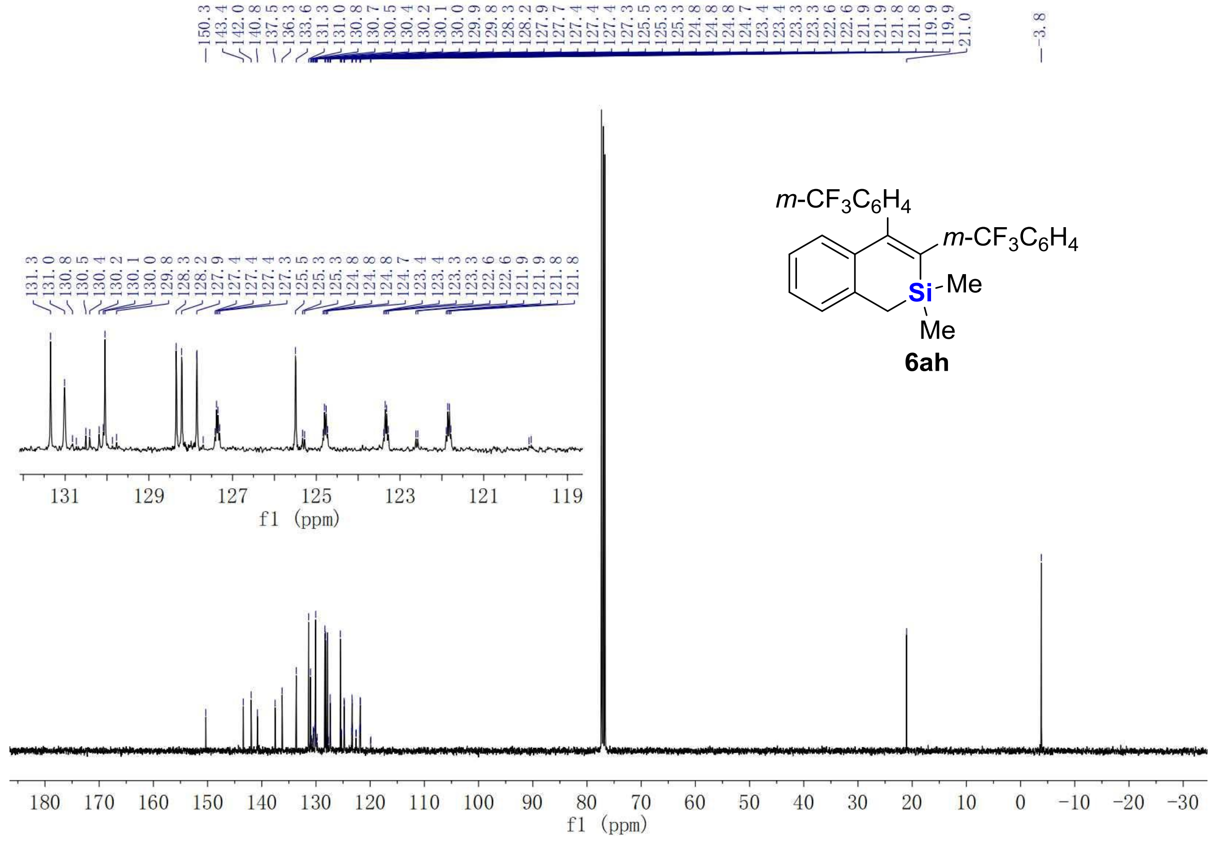
150.3 143.4 142.0 140.8 137.5 136.3 133.6 131.3 131.0 130.8 130.7 130.5 130.4 130.2 130.1 130.0 129.9 129.8 128.3 128.2 127.9 127.7 127.4 127.4 127.4 127.3 125.5 125.3 125.3 124.8 124.8 124.8 124.7 123.4 123.4 123.3 123.3 122.6 122.6 121.9 121.9 121.8 121.8 119.9 119.9 21.0
-3.8
131.3 131.0 130.8 130.5 130.4 130.2 130.1 130.0 129.8 128.3 128.2 127.9 127.4 127.4 127.4 127.3 125.5 125.3 125.3 124.8 124.8 124.7 123.4 123.4 123.3 123.3 122.6 122.6 121.9 121.8
131 129 127 125 123 121 119
f1 (ppm)
m-CF3C6H4
m-CF3C6H4
Si
Me
Me
6ah
180 170 160 150 140 130 120 110 100 90 80 70 60 50 40 30 20 10 0 -10 -20 -30
f1 (ppm)

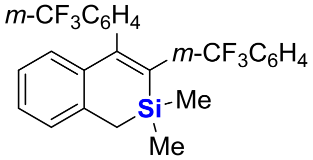

6ah

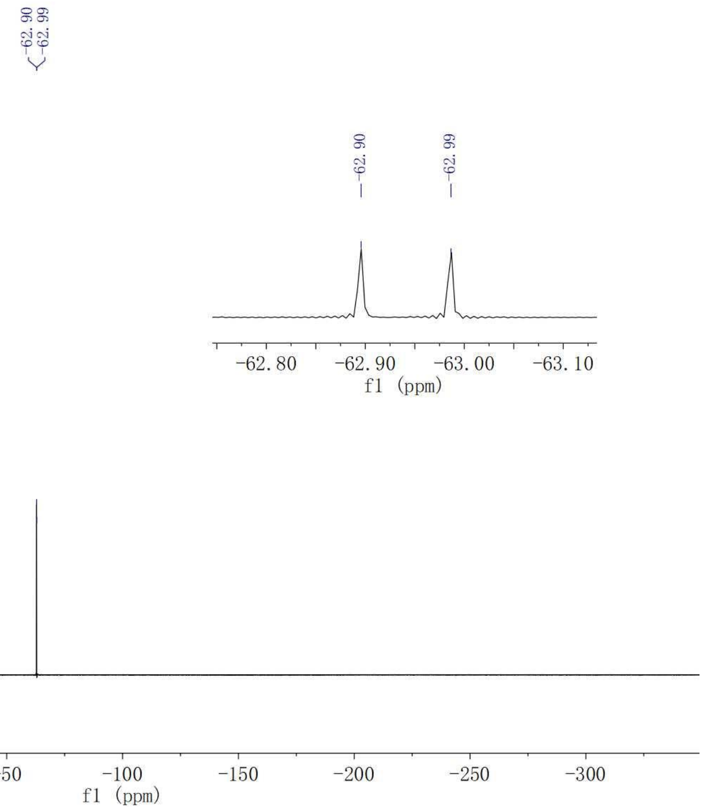

Supplementary Figure 92 ^{1}H, ^{13}C and ^{19}F NMR Spectra for compound 6ah

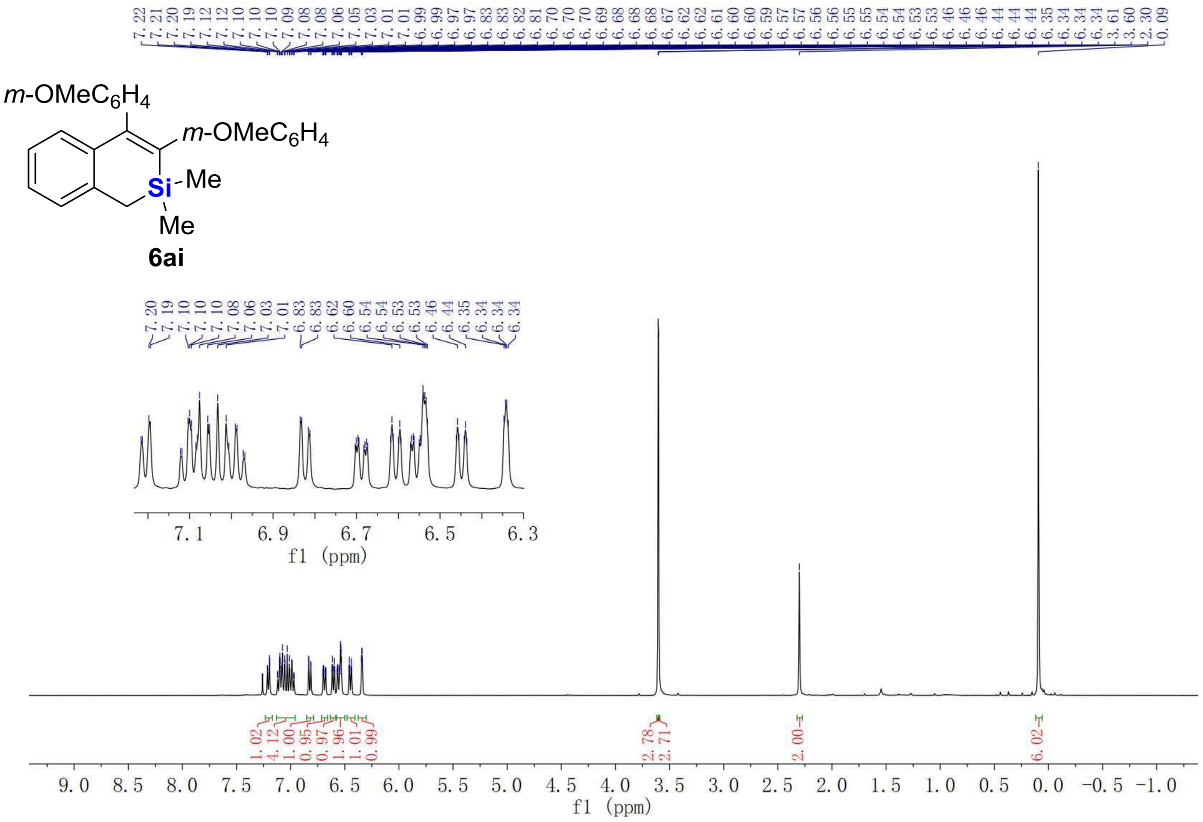

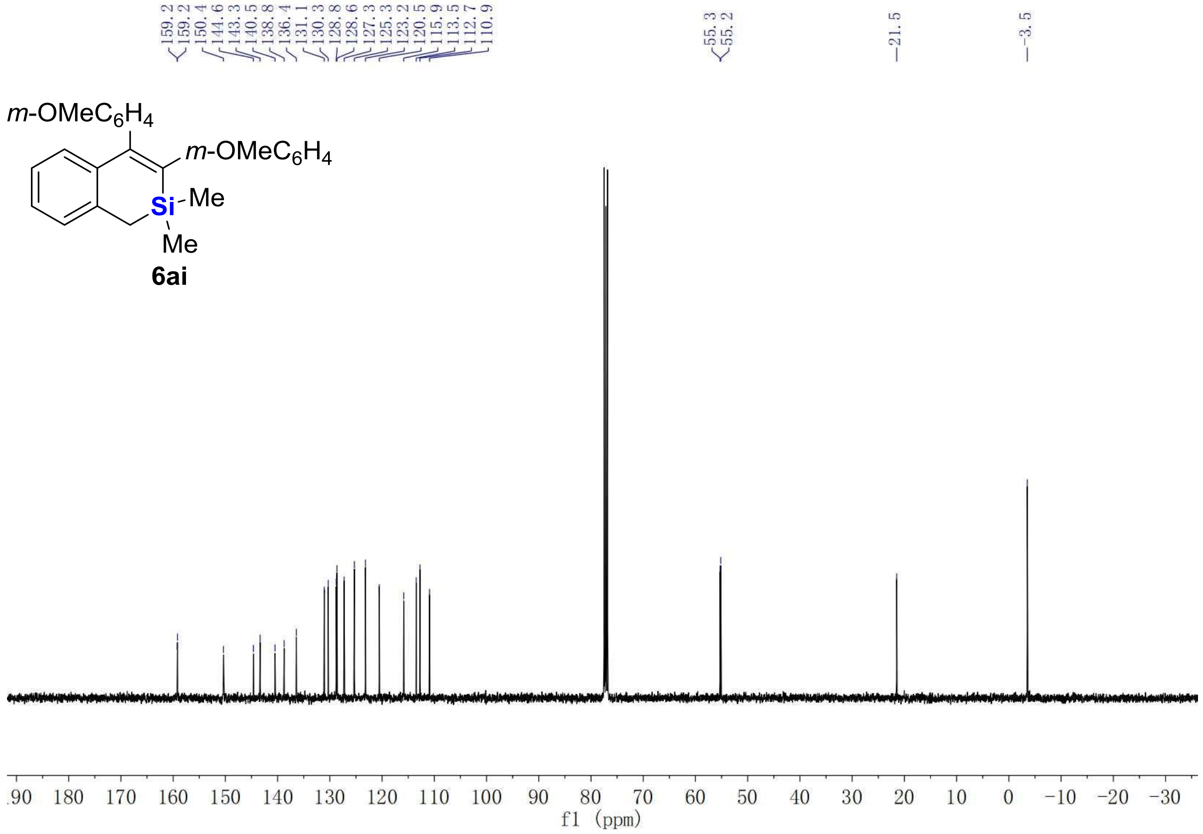

Supplementary Figure 93 ^{1}H and ^{13}C NMR Spectra for compound 6ai

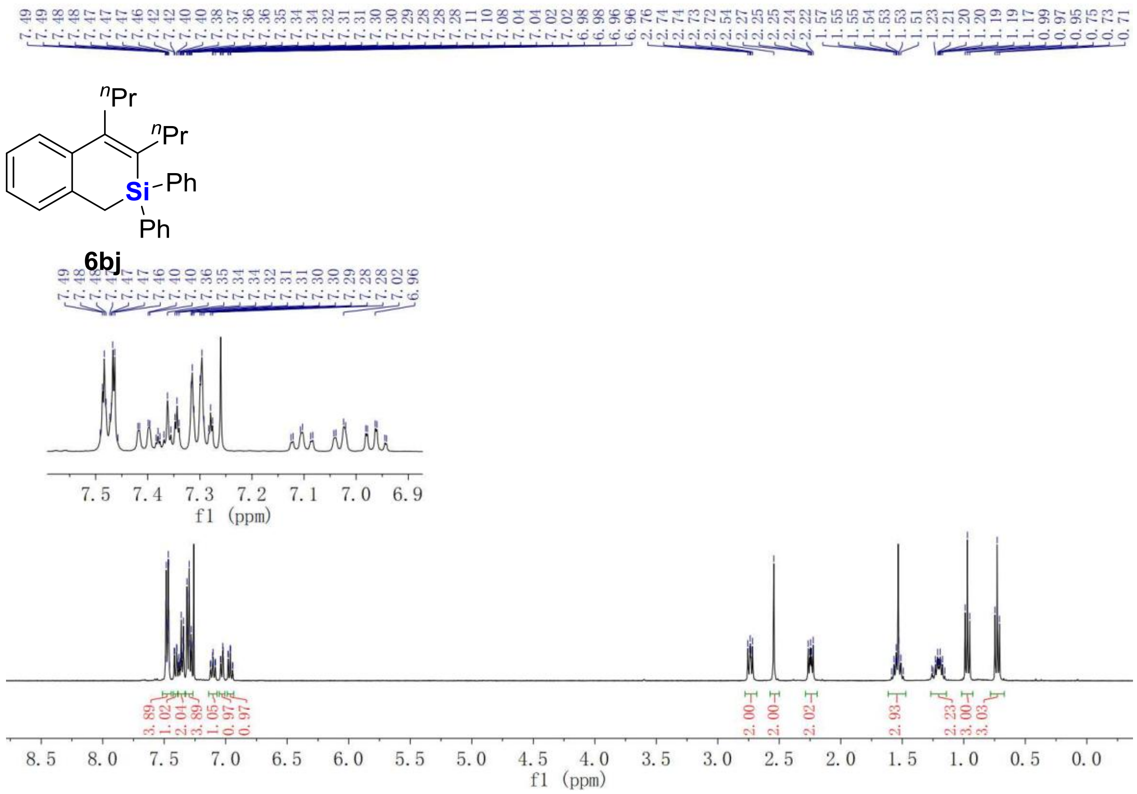

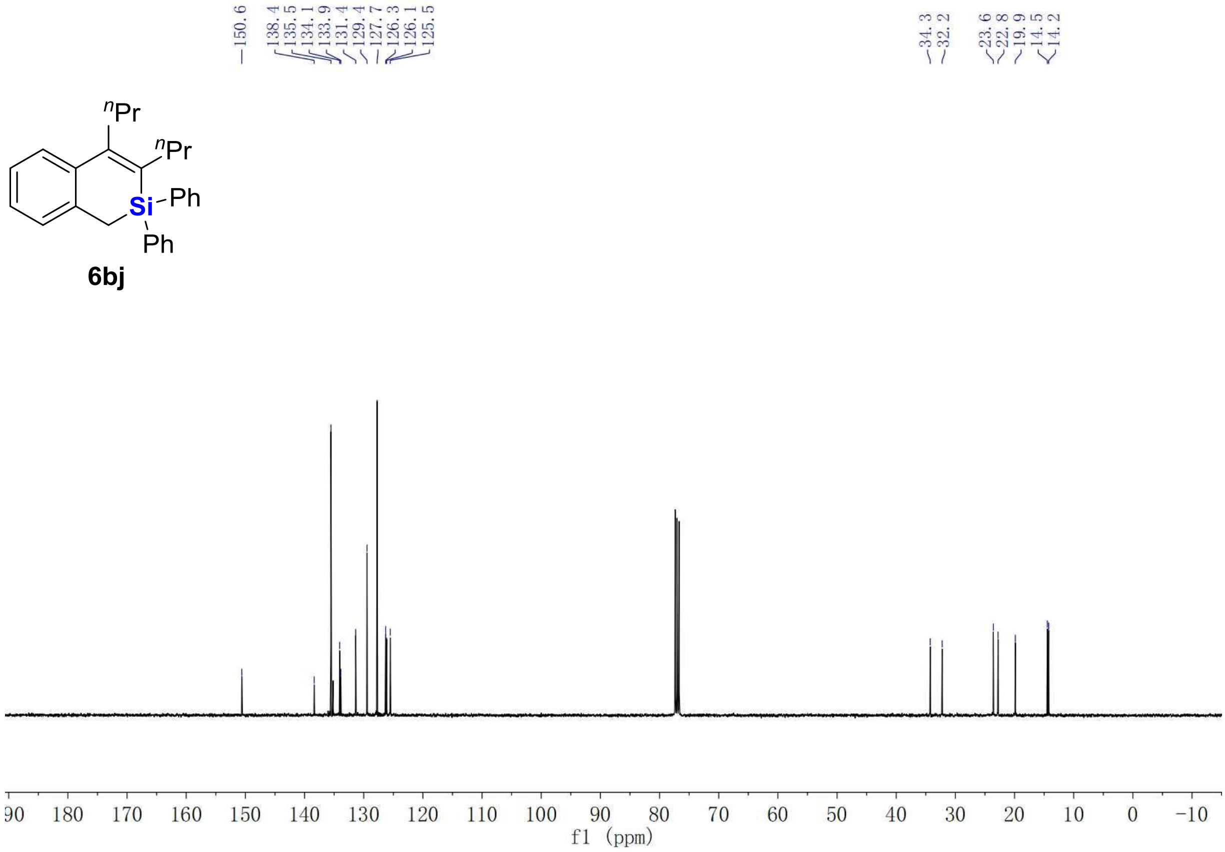

Supplementary Figure 94 ^{1}H and ^{13}C NMR Spectra for compound 6bj

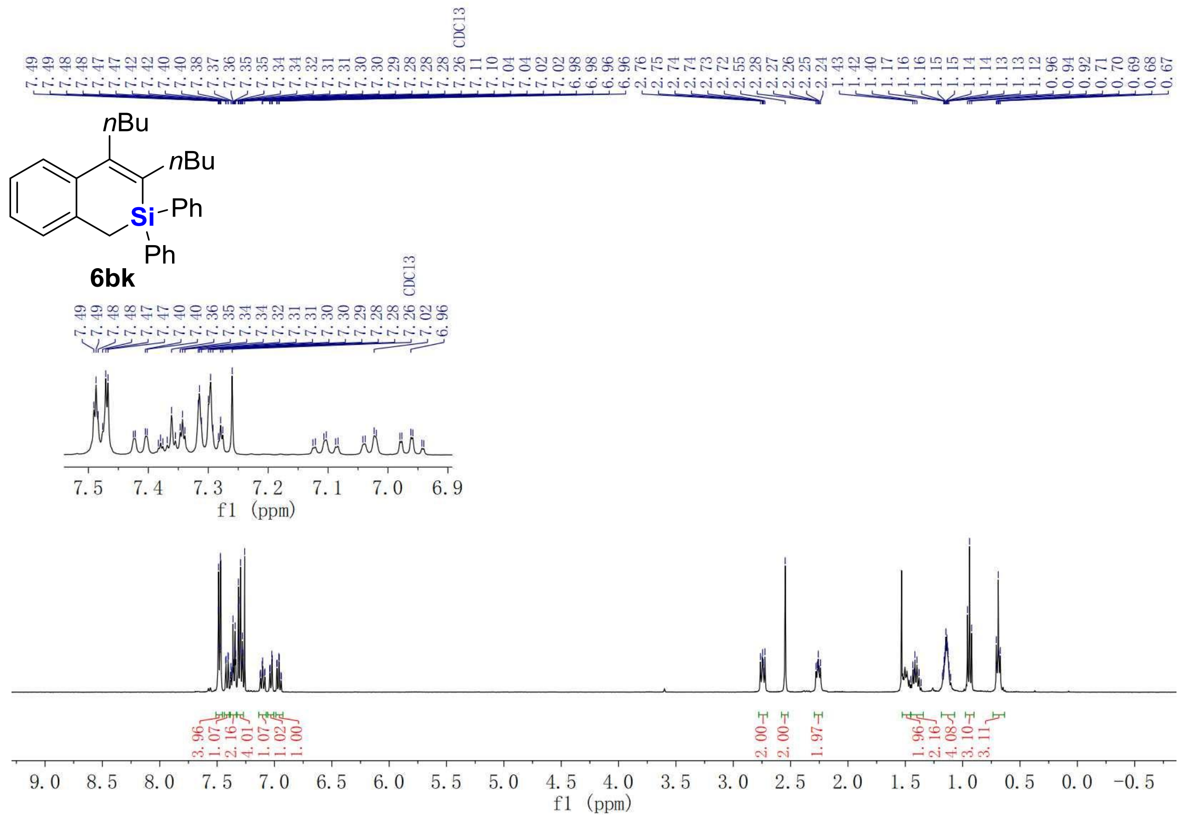

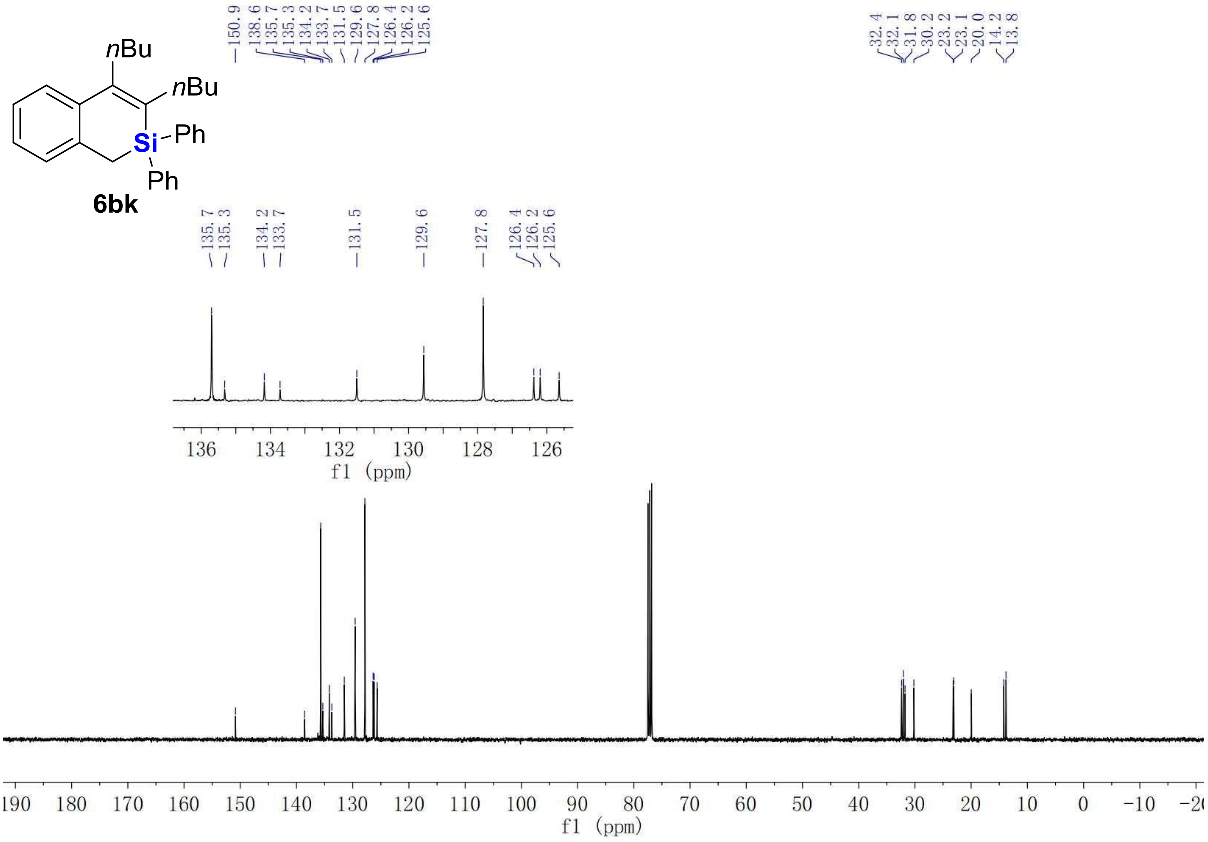

Supplementary Figure 95 ^{1}H and ^{13}C NMR Spectra for compound 6bk

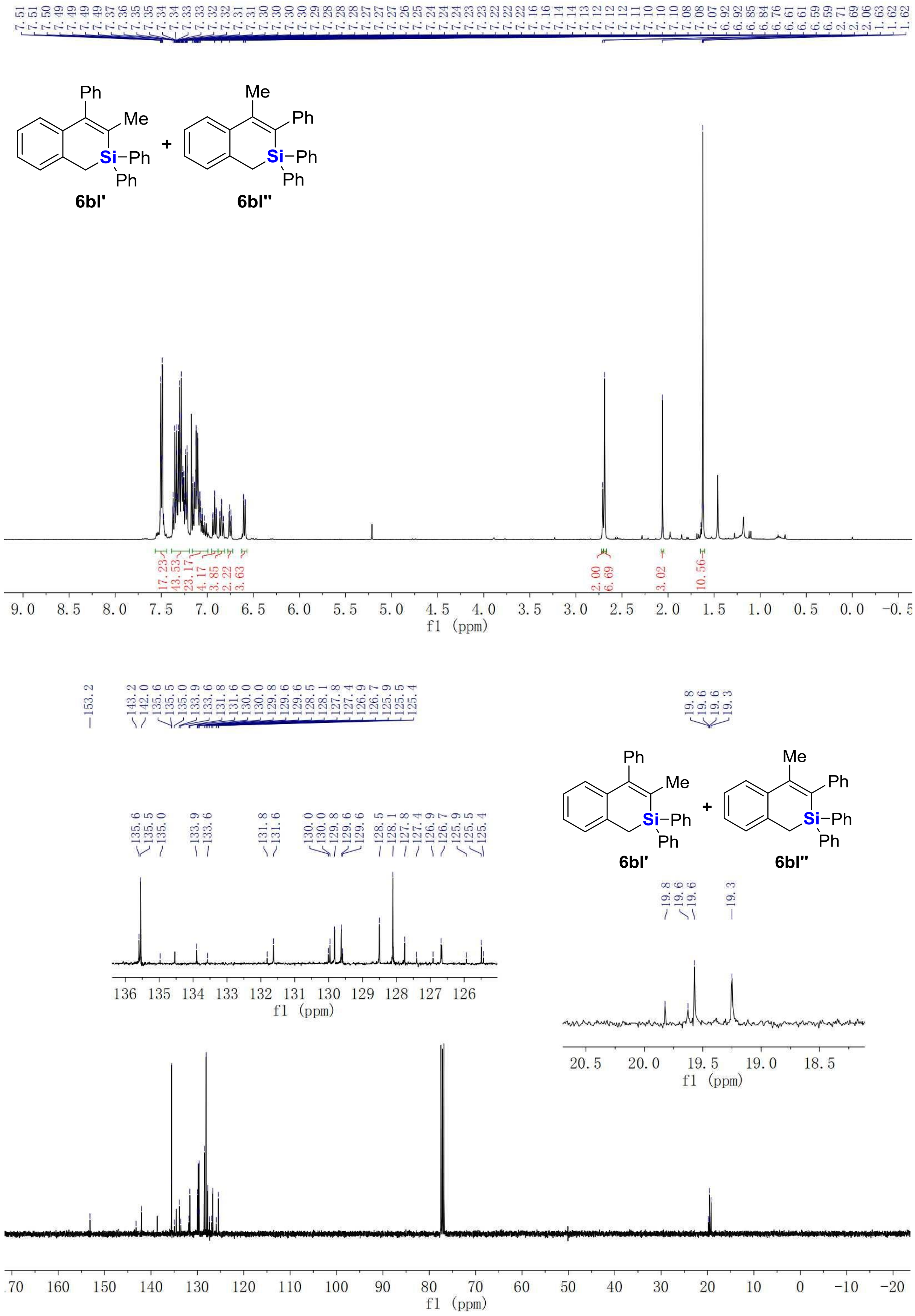

Supplementary Figure 96 ^{1}H and ^{13}C NMR Spectra for compound 6bl

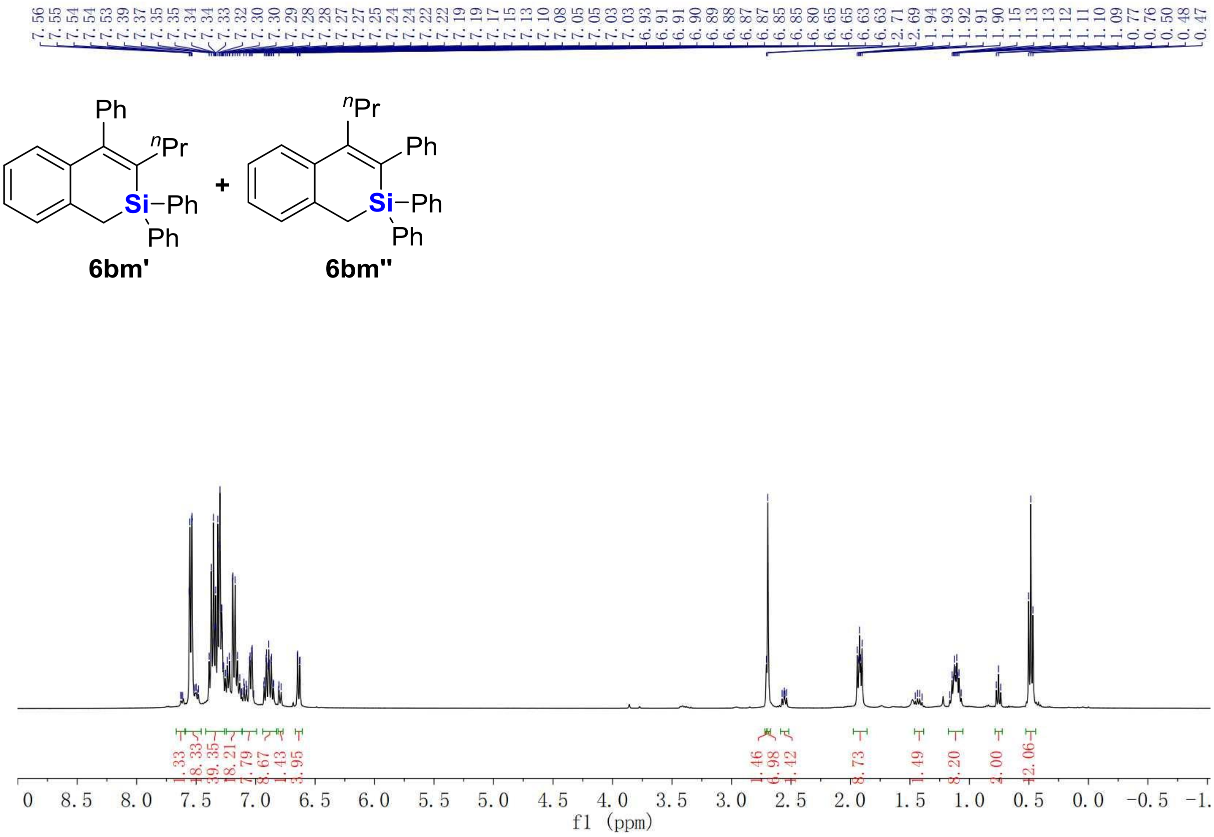

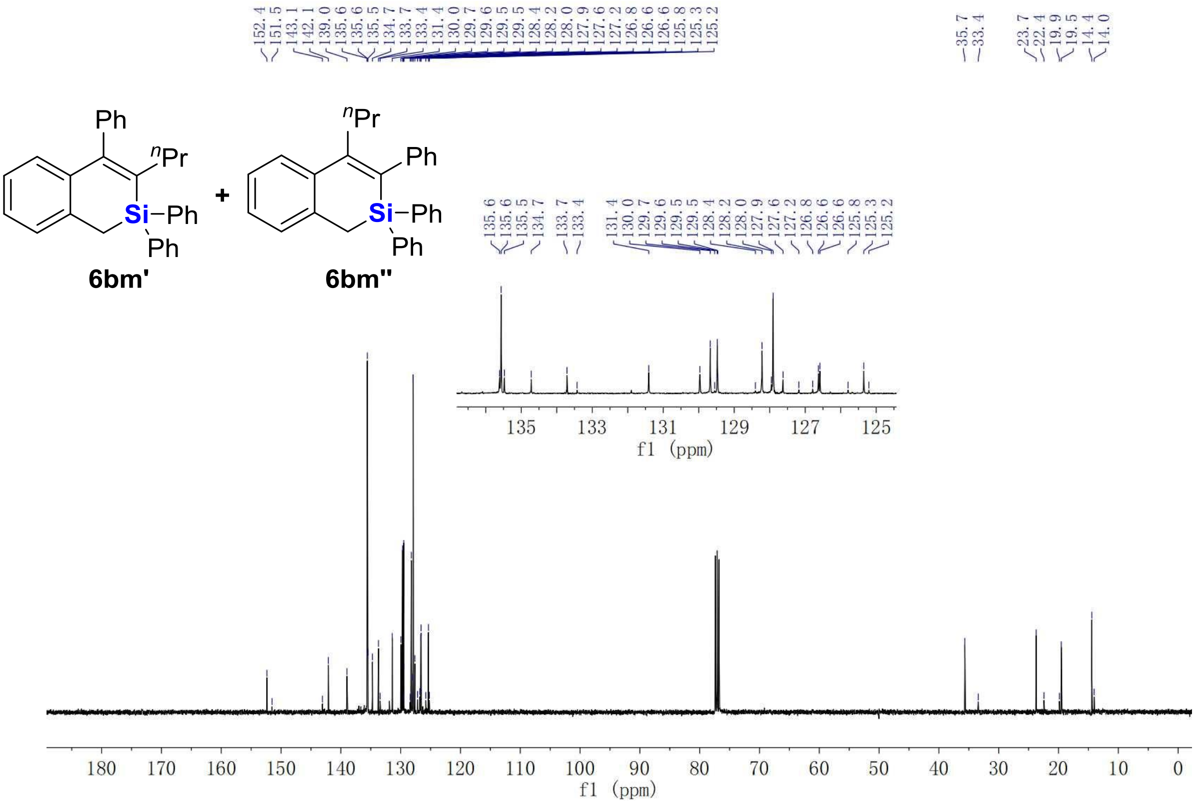

Supplementary Figure 97 ^{1}H and ^{13}C NMR Spectra for compound 6bm

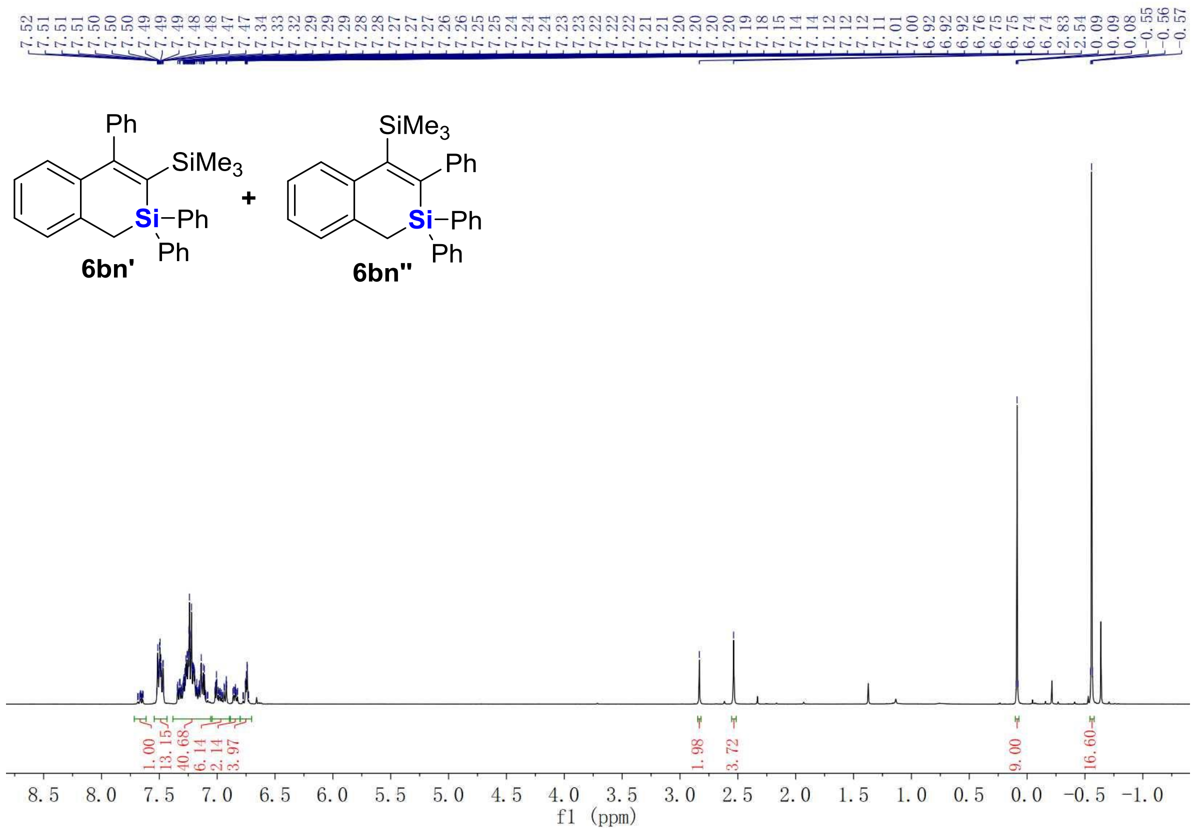

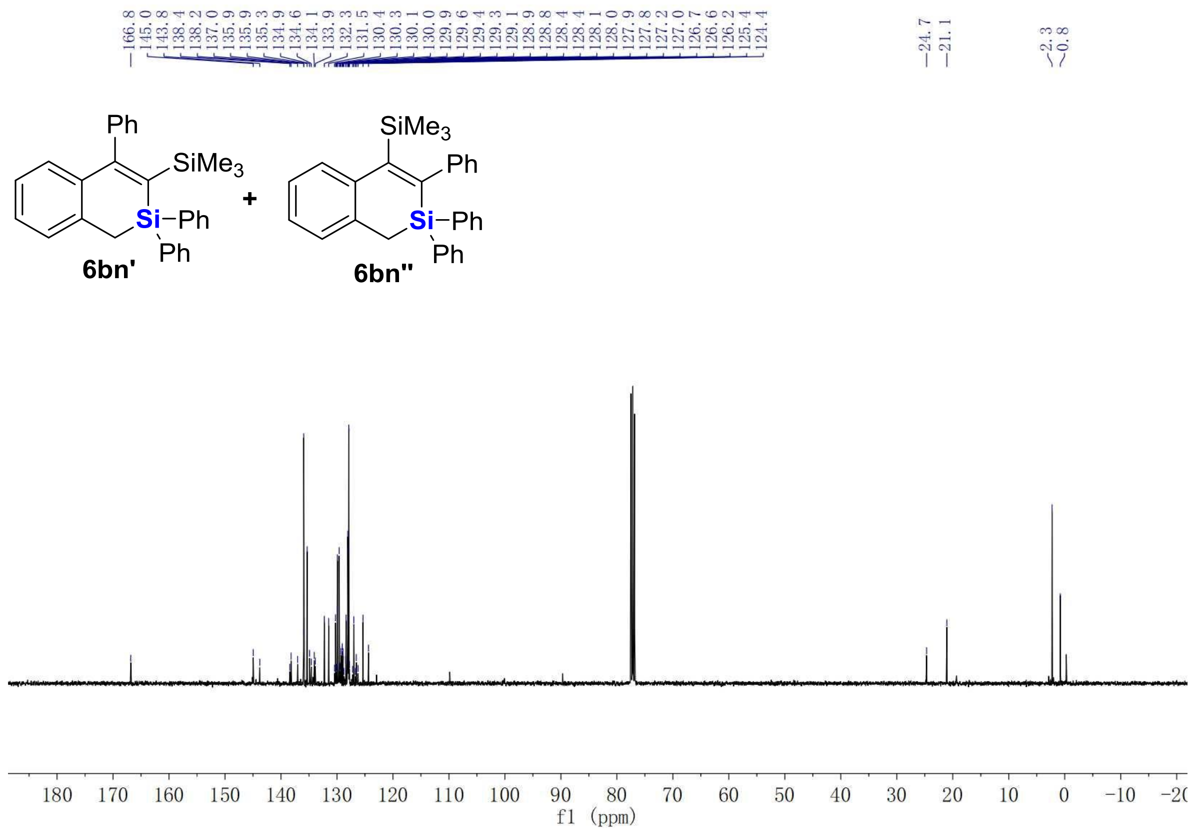

Supplementary Figure 98 ^{1}H and ^{13}C NMR Spectra for compound 6bn

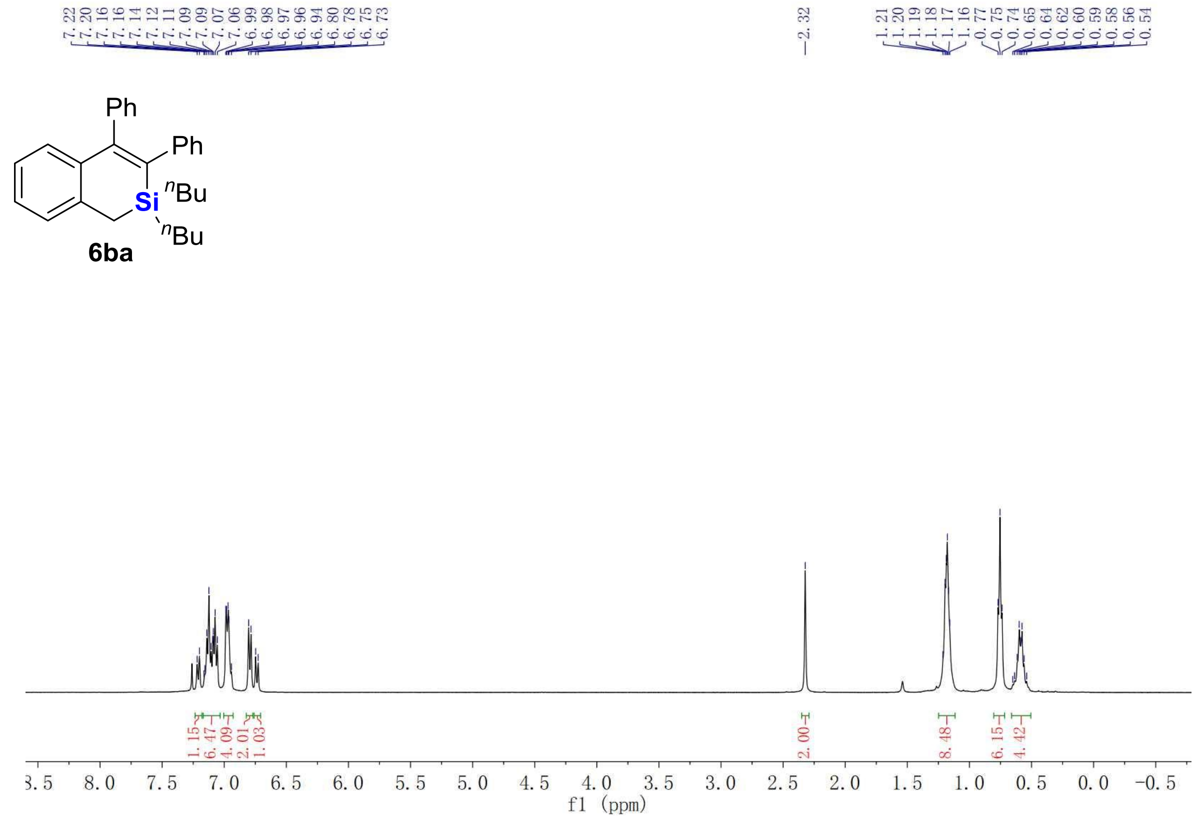

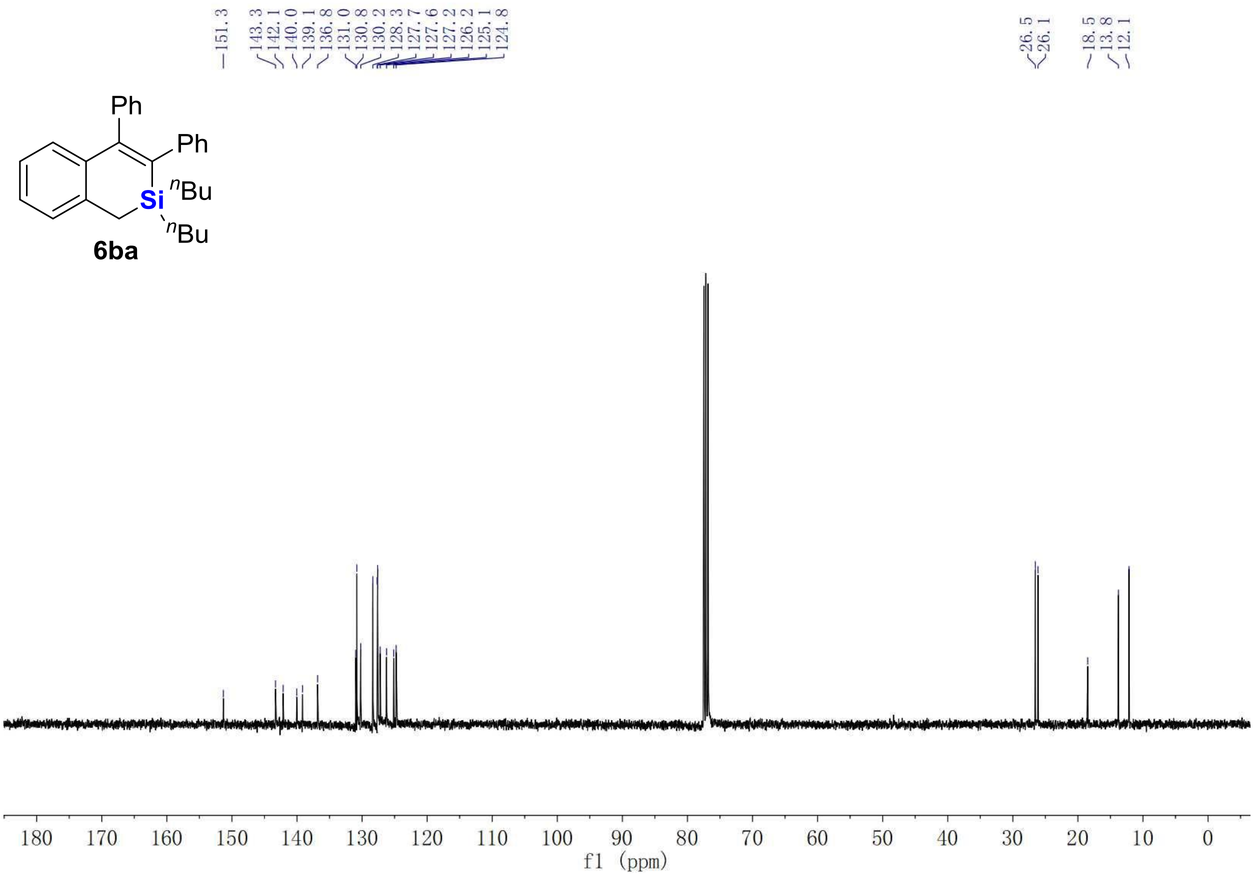

Supplementary Figure 99 ^{1}H and ^{13}C NMR Spectra for compound 6ba

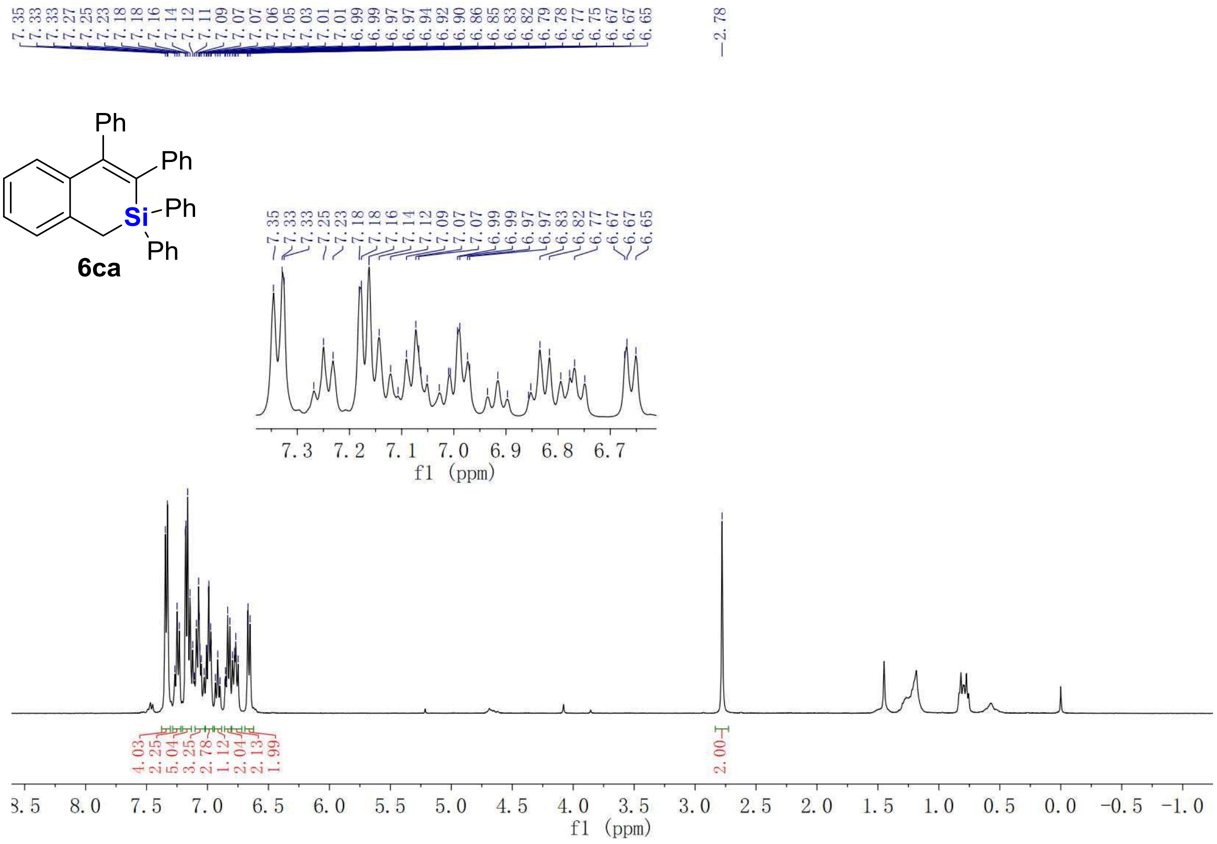

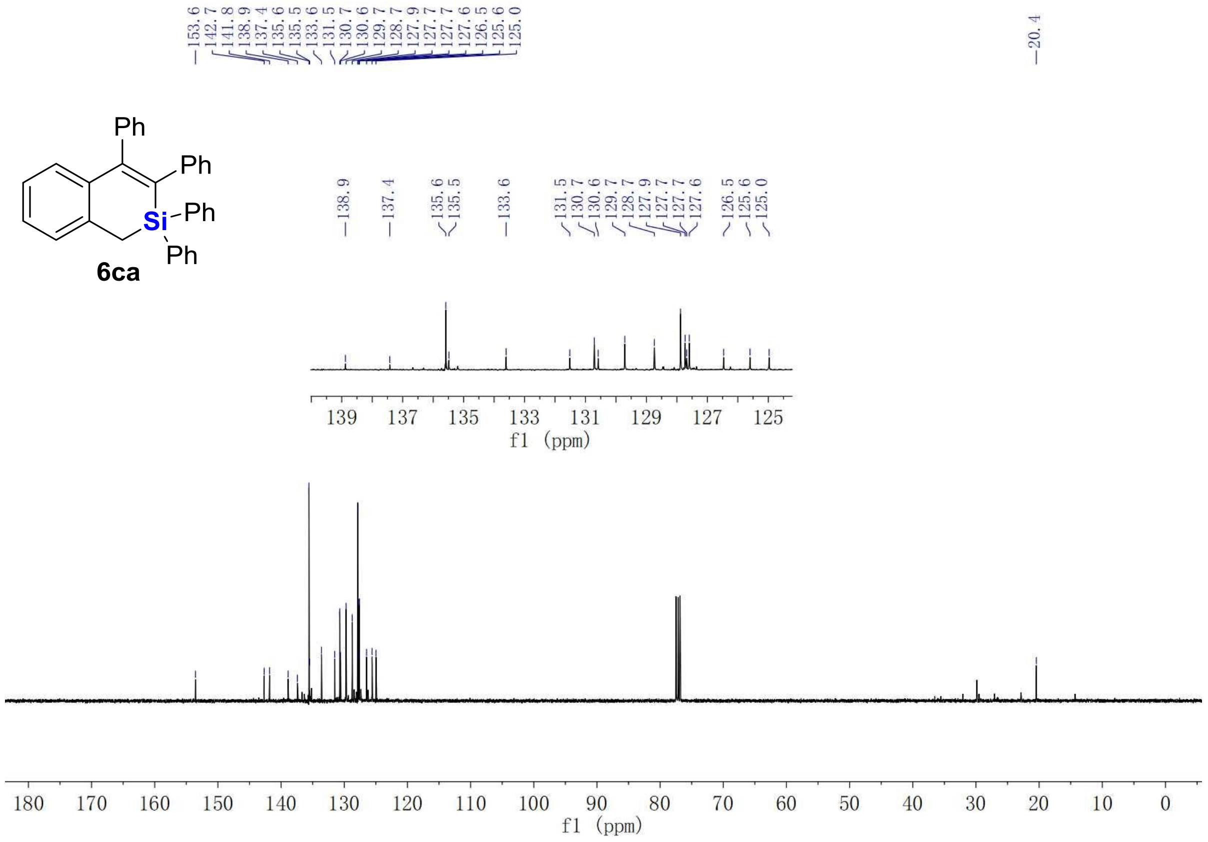

Supplementary Figure 100 ^{1}H and ^{13}C NMR Spectra for compound 6ca

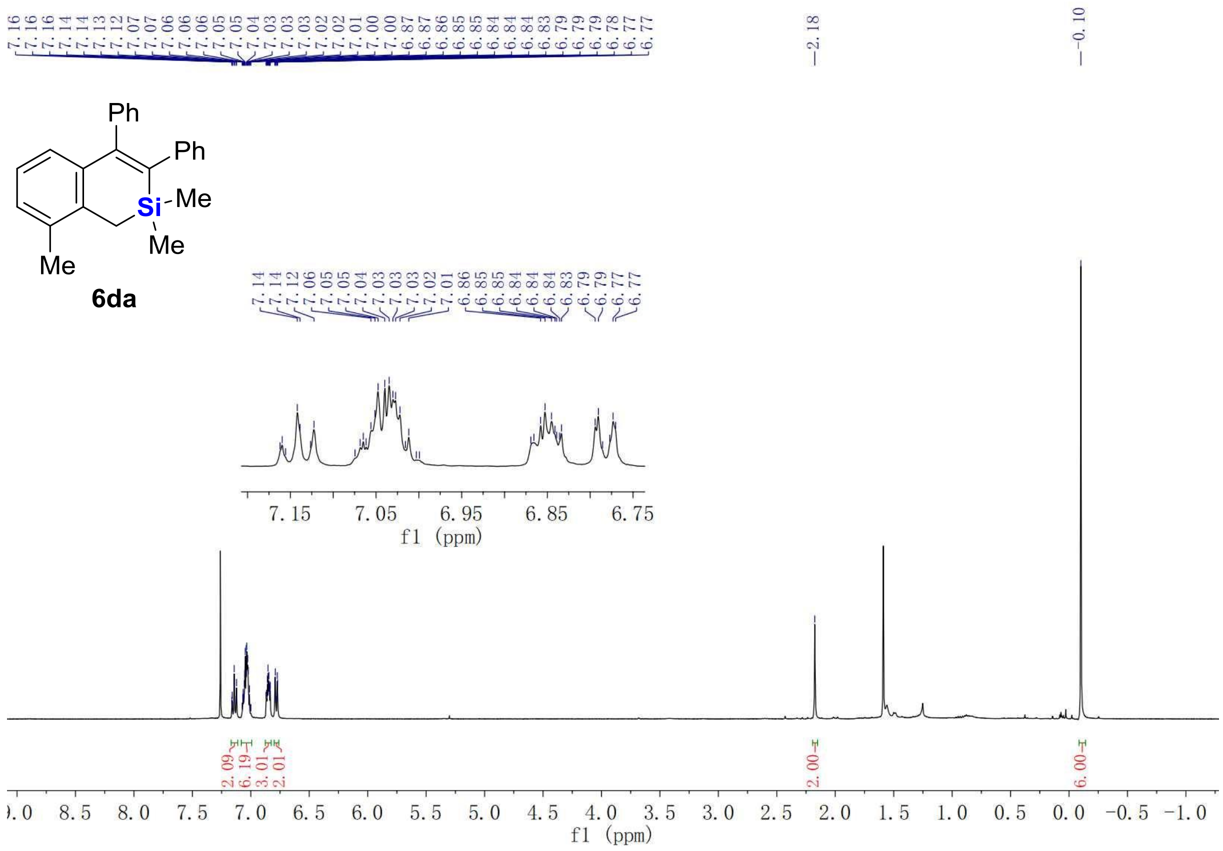

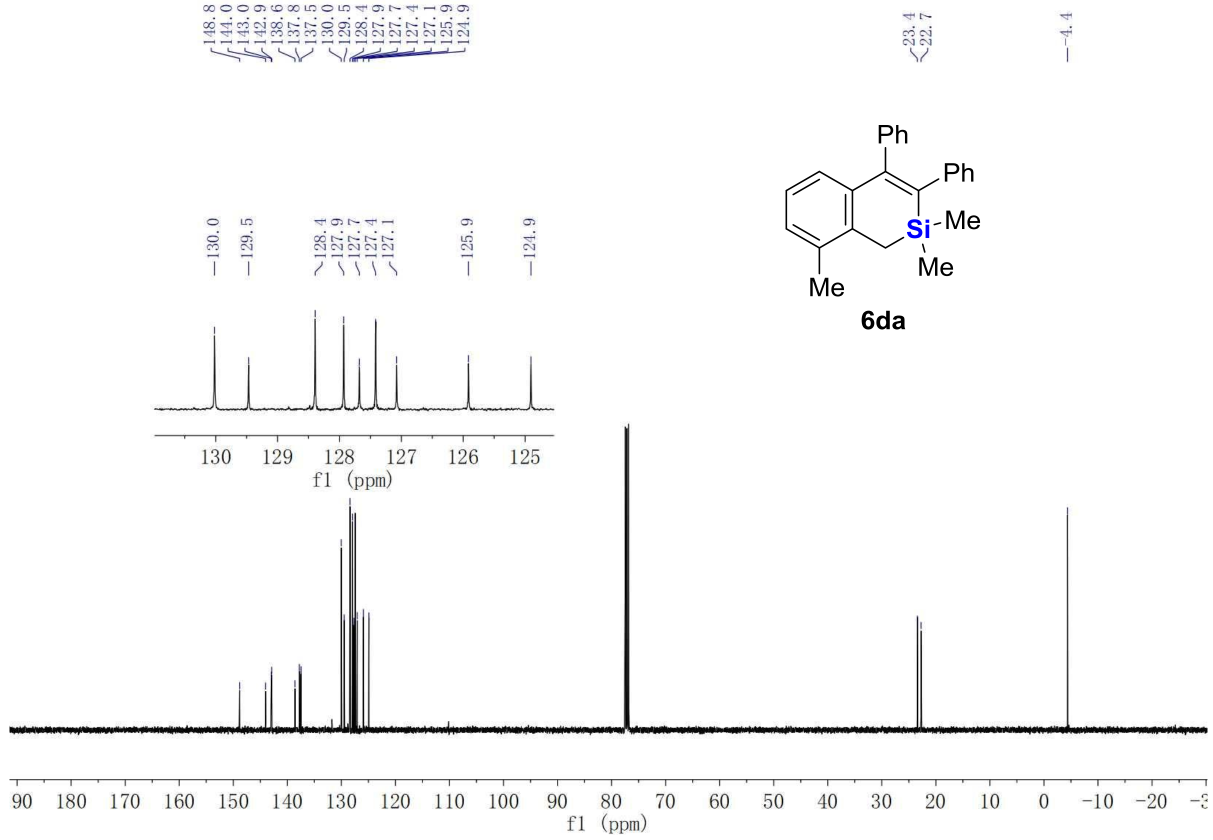

Supplementary Figure 101 ^{1}H and ^{13}C NMR Spectra for compound 6da

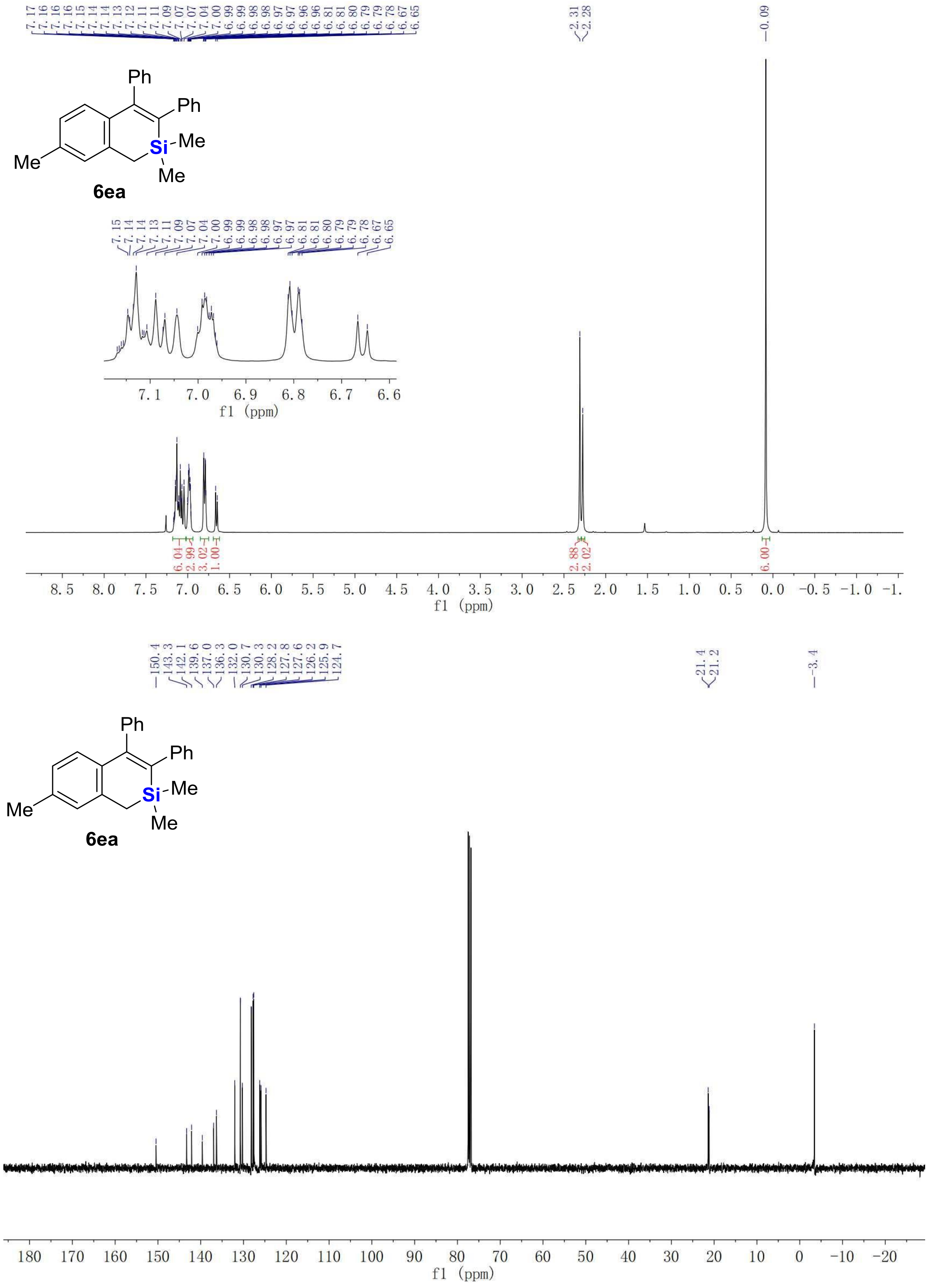

Supplementary Figure 102 ^{1}H and ^{13}C NMR Spectra for compound 6ea

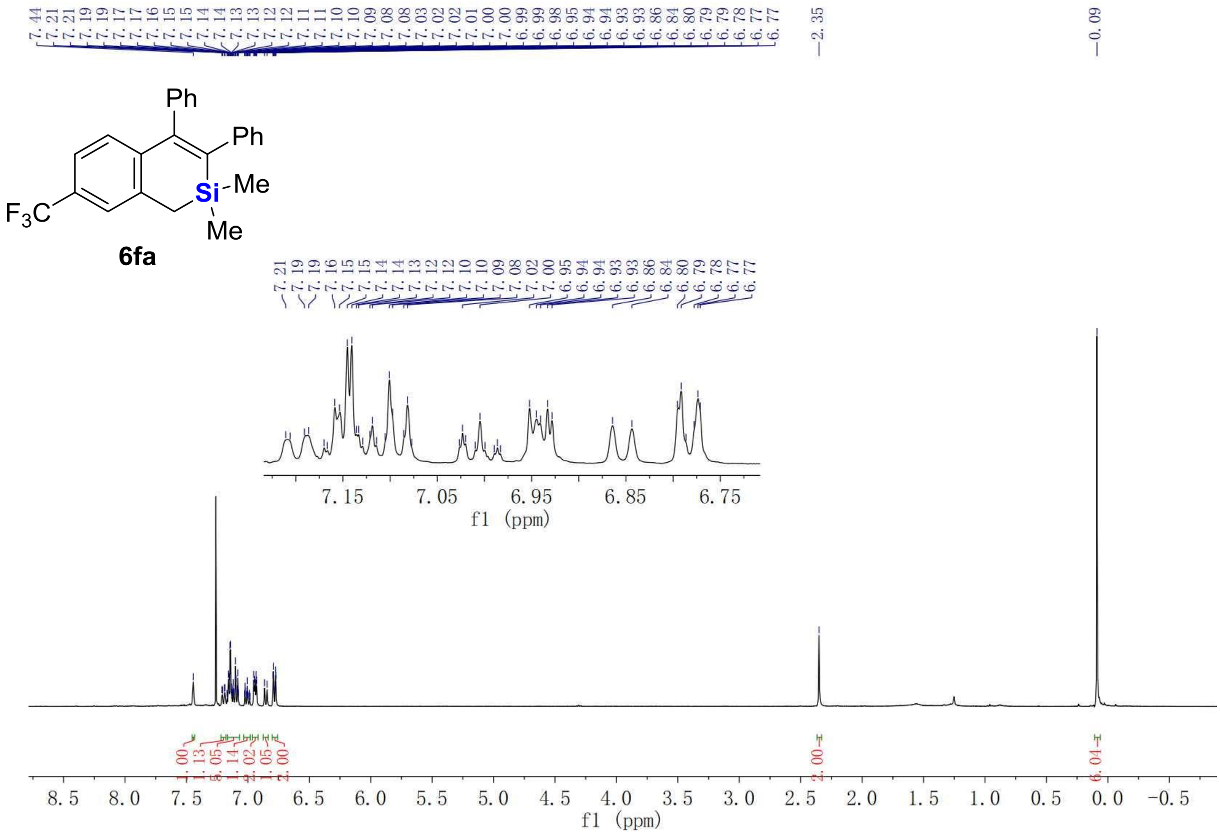
7.44
7.21
7.21
7.19
7.19
7.17
7.17
7.16
7.15
7.15
7.15
7.14
7.14
7.13
7.13
7.12
7.12
7.11
7.11
7.10
7.10
7.09
7.08
7.08
7.03
7.02
7.02
7.01
7.00
7.00
6.99
6.99
6.98
6.95
6.94
6.94
6.93
6.93
6.86
6.84
6.80
6.79
6.78
6.77
6.77
2.35
0.09
Ph
Ph
F3C
Si
Me
Me
6fa
f1 (ppm)

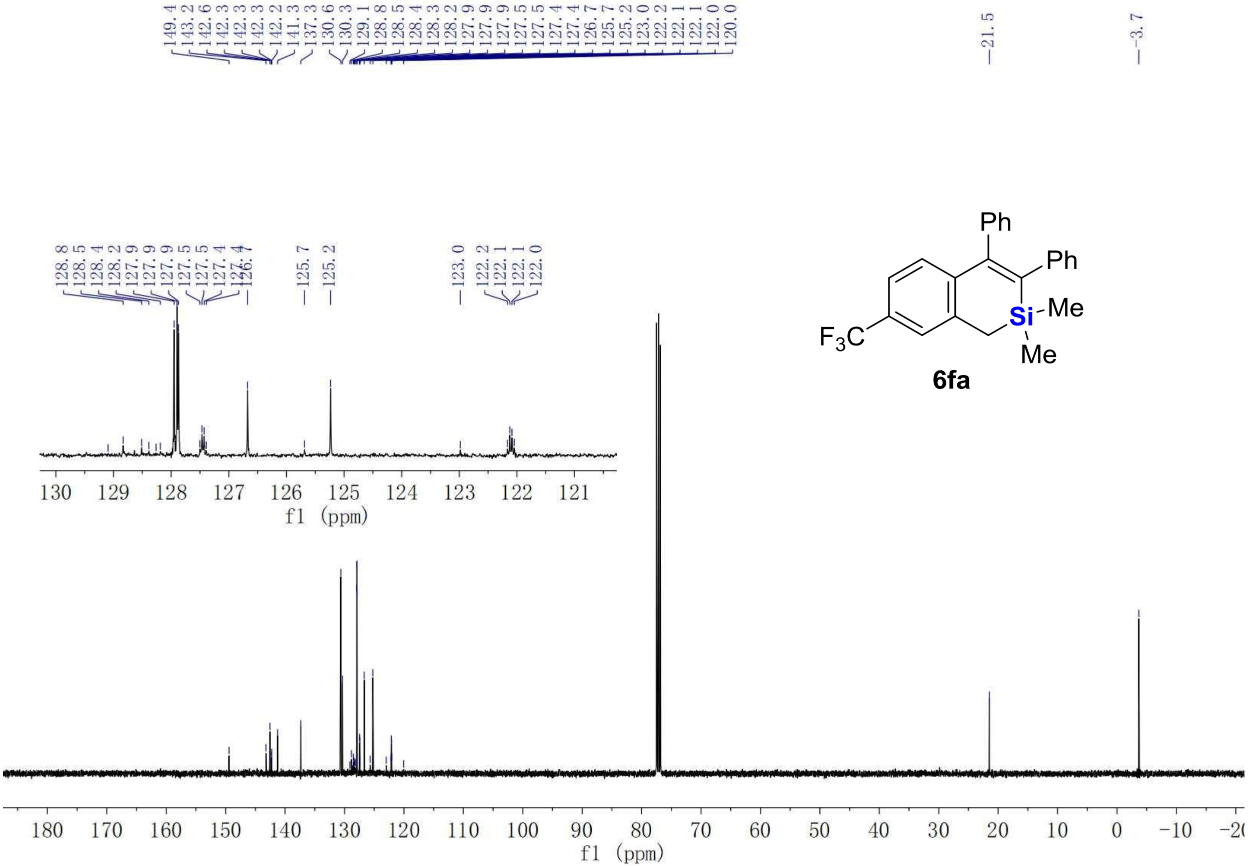
149.4
143.2
142.6
142.3
142.3
142.3
142.2
141.3
137.3
130.6
130.3
129.1
128.8
128.5
128.4
128.3
128.2
127.9
127.9
127.9
127.5
127.5
127.4
127.4
126.7
125.7
125.2
123.0
122.2
122.1
122.1
122.0
120.0
21.5
-3.7
Ph
Ph
F3C
Si
Me
Me
6fa
f1 (ppm)

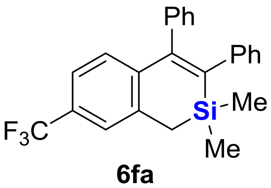

6fa

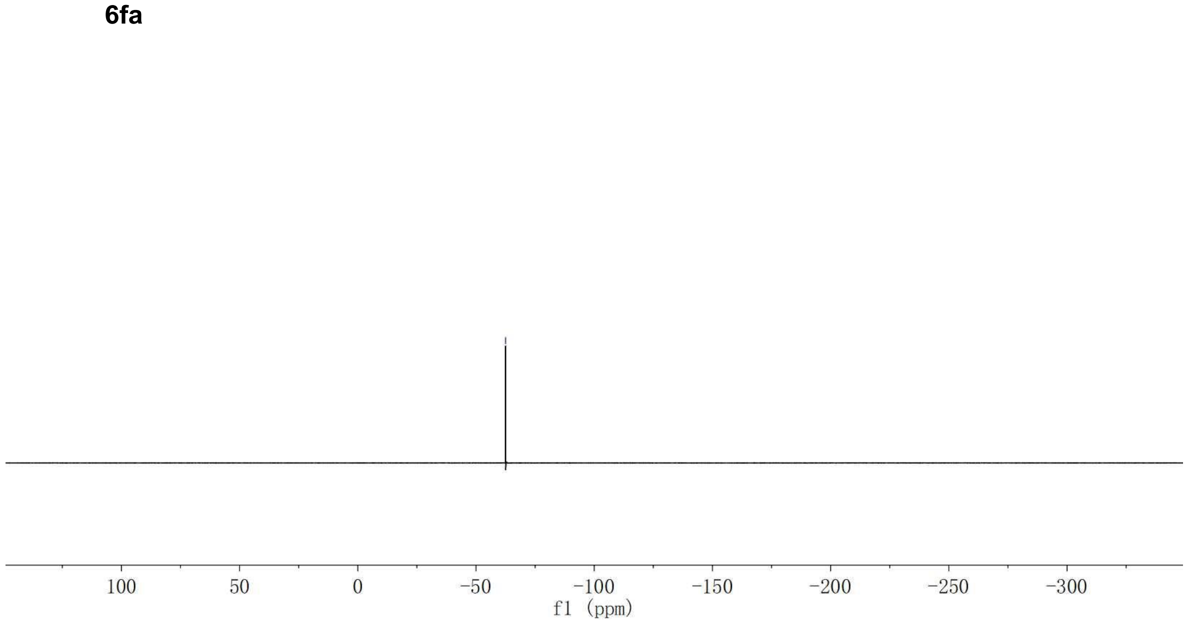

Supplementary Figure 103 ^{1}H, ^{13}C and ^{19}F NMR Spectra for compound 6fa

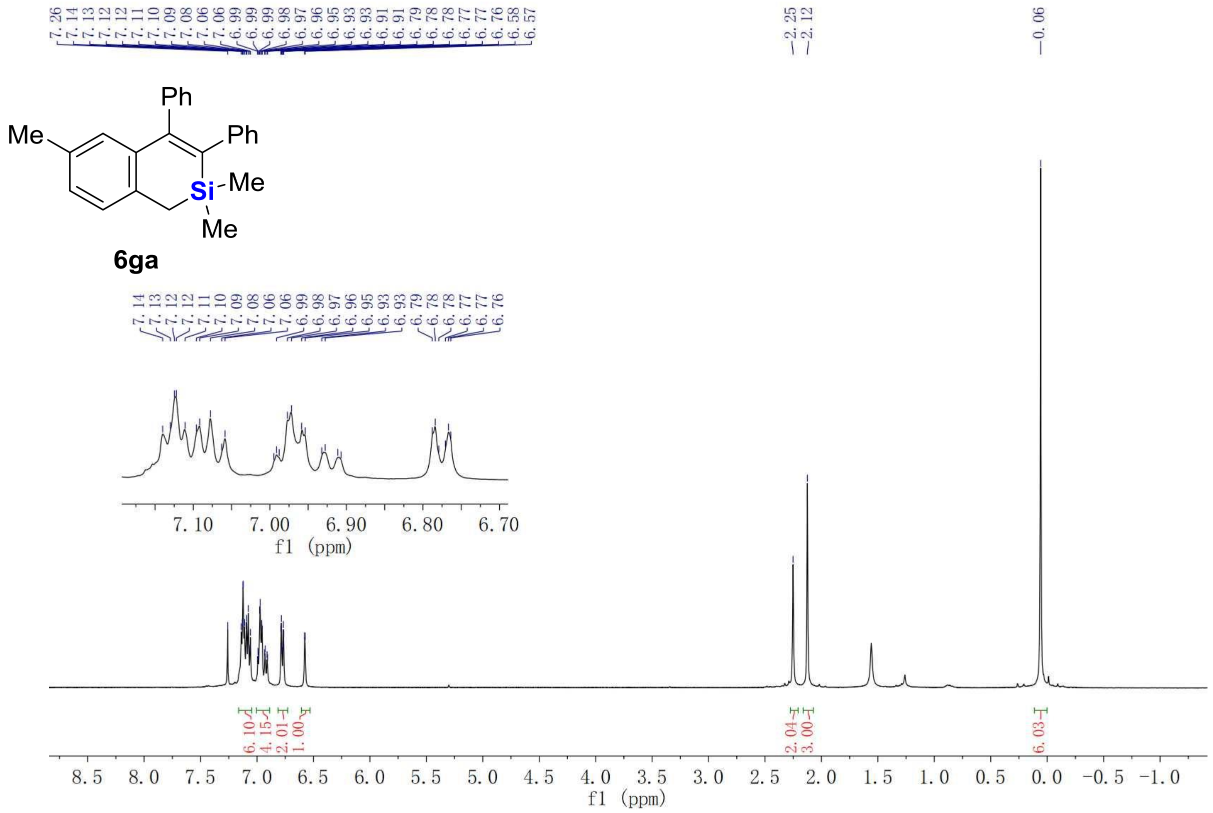

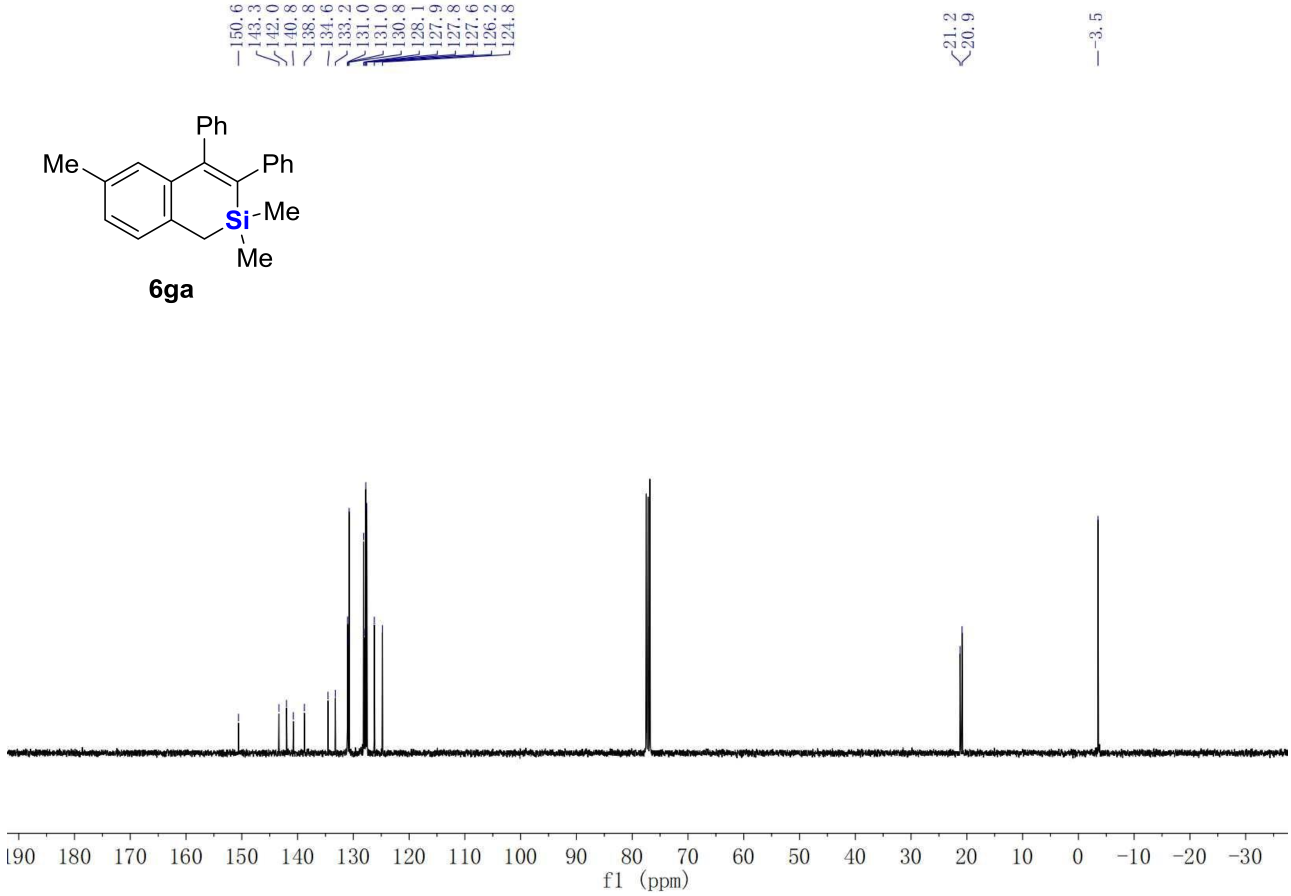

Supplementary Figure 104 ^{1}H and ^{13}C NMR Spectra for compound 6ga

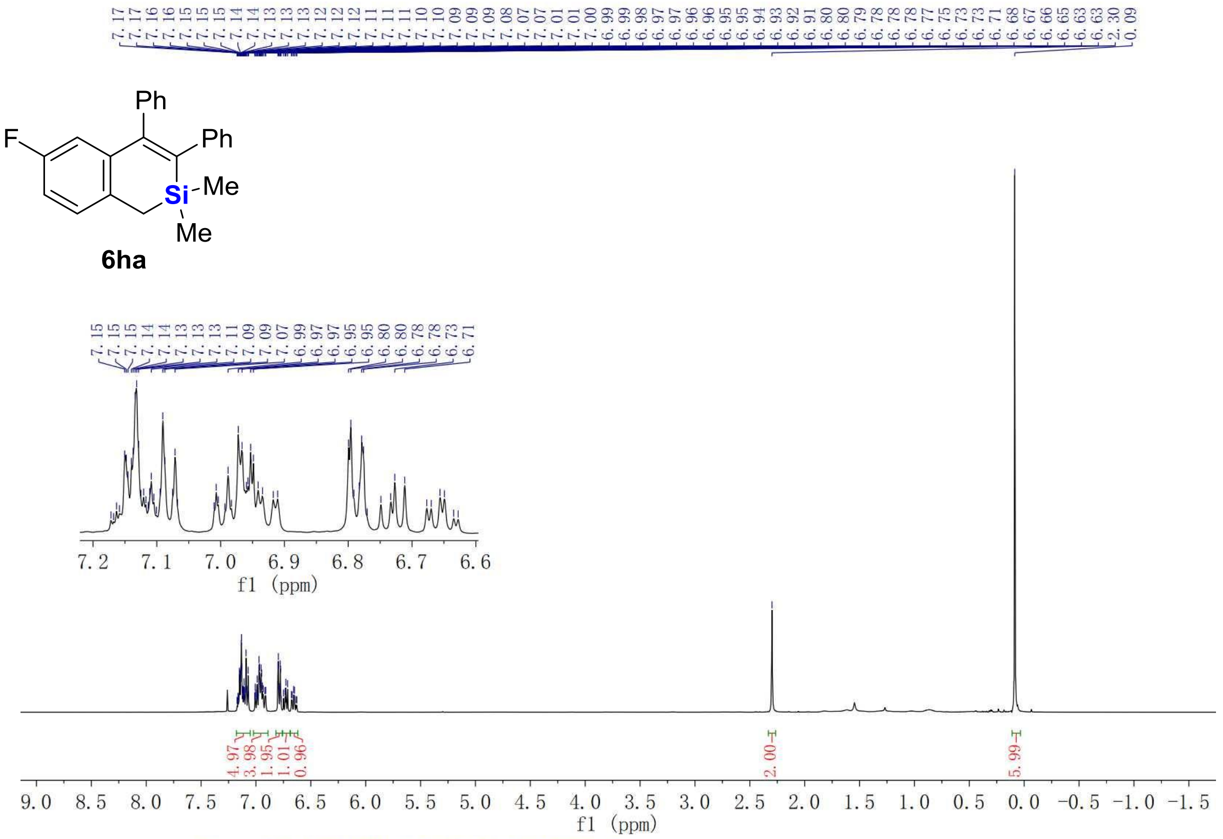

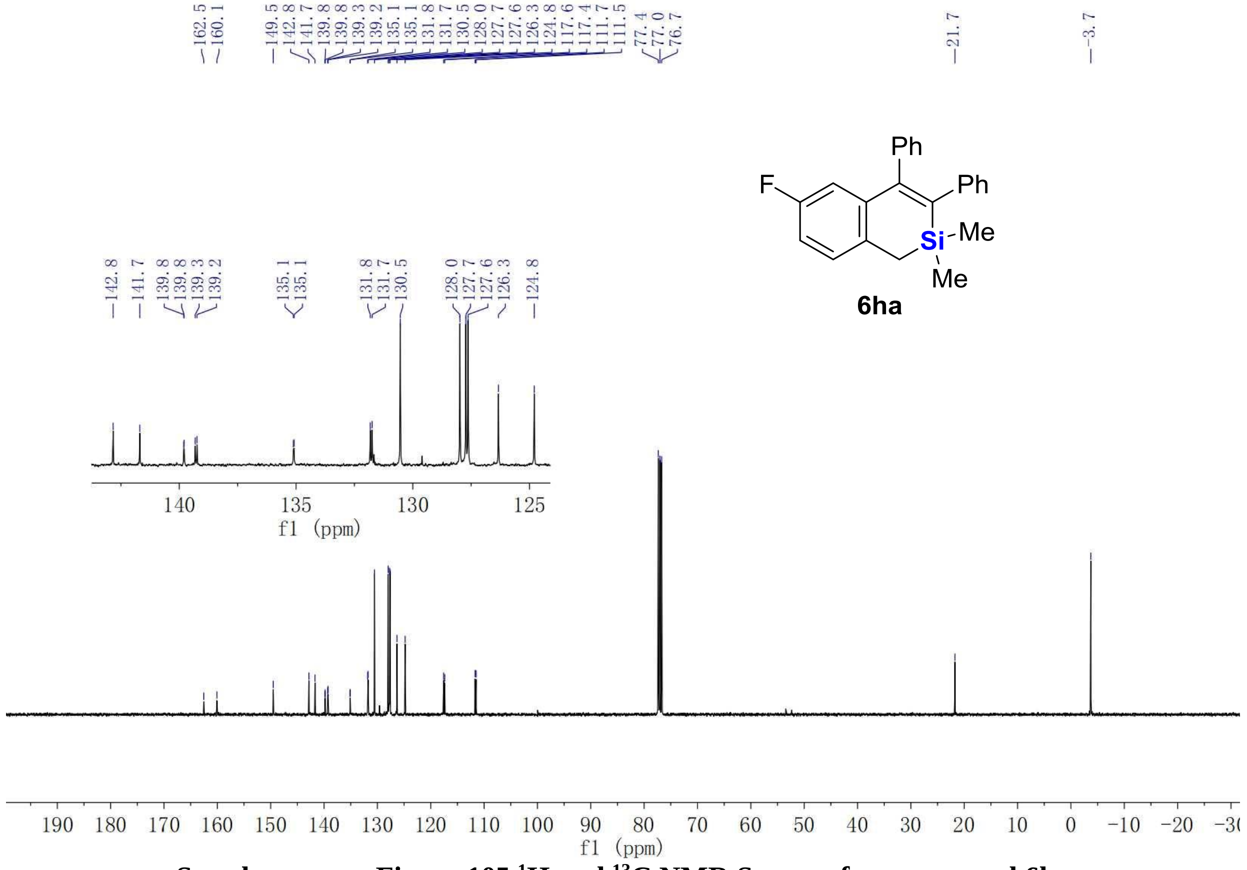

Supplementary Figure 105 ^{1}H and ^{13}C NMR Spectra for compound 6ha

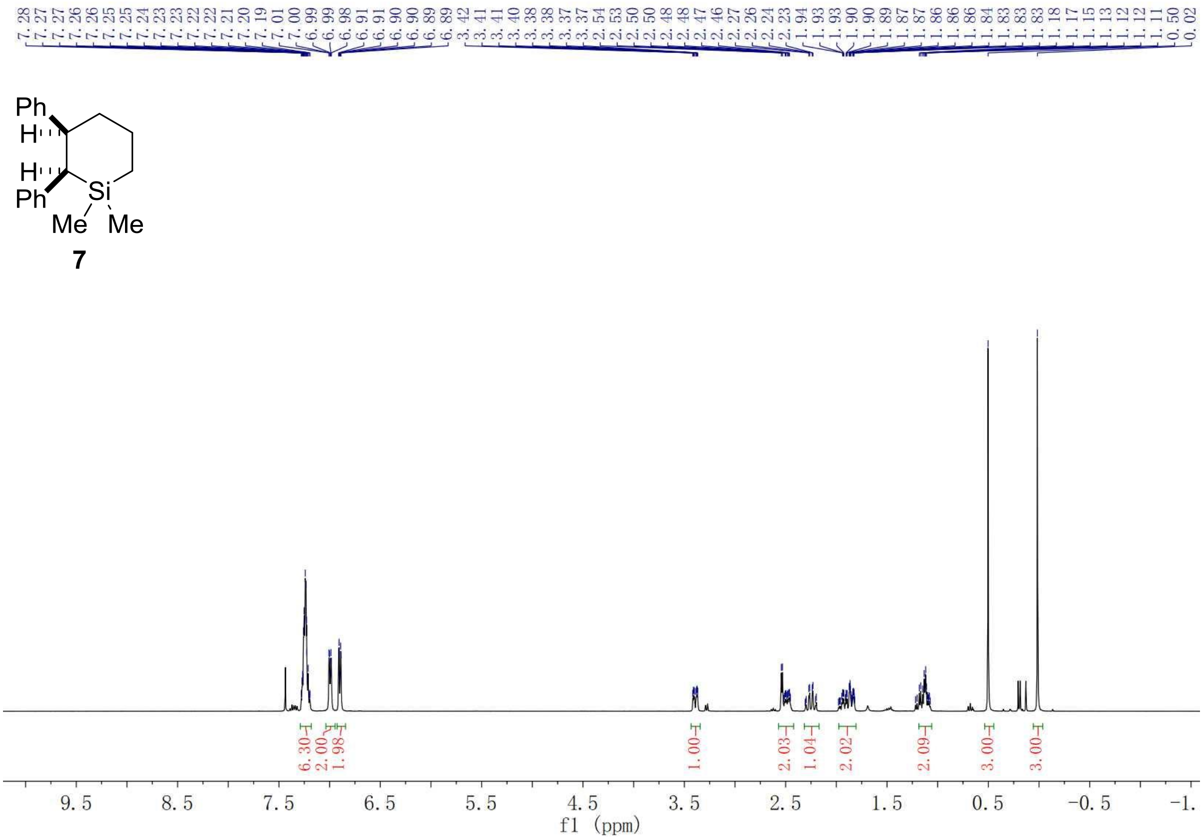

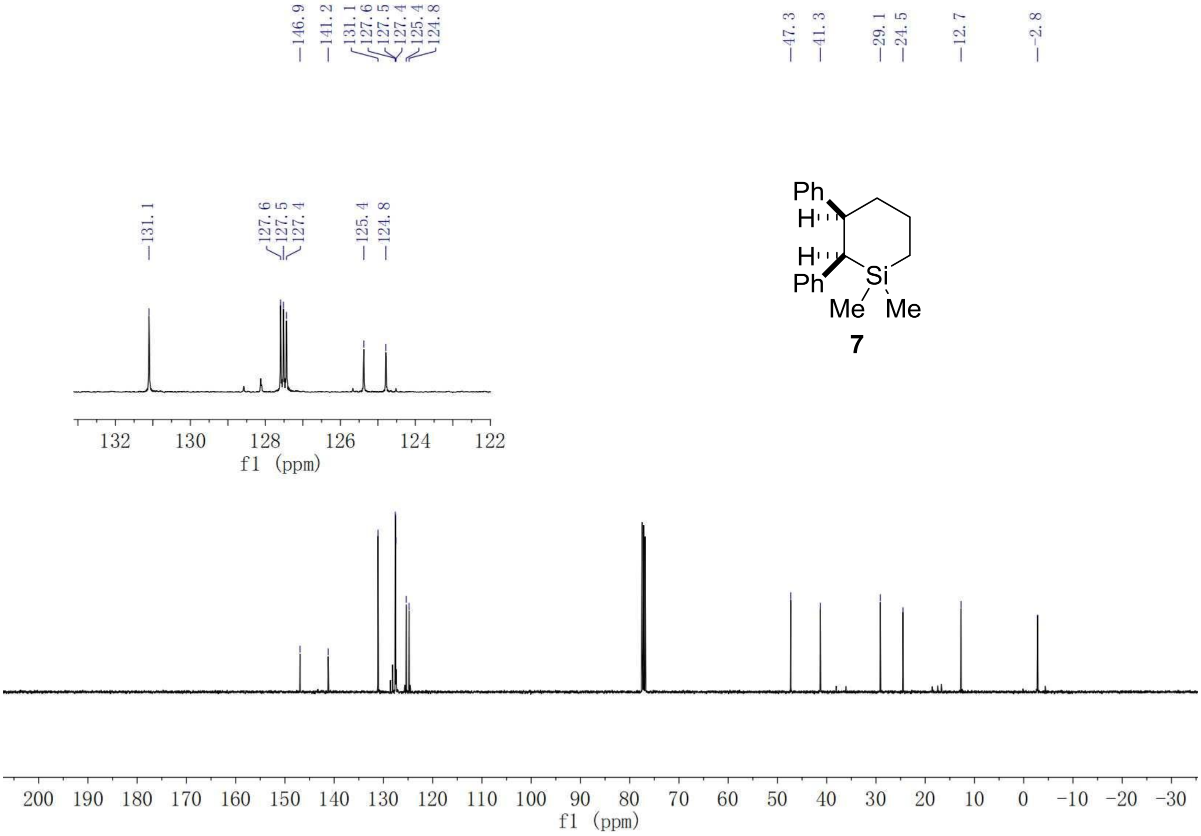

Supplementary Figure 106 ^{1}H and ^{13}C NMR Spectra for compound 7

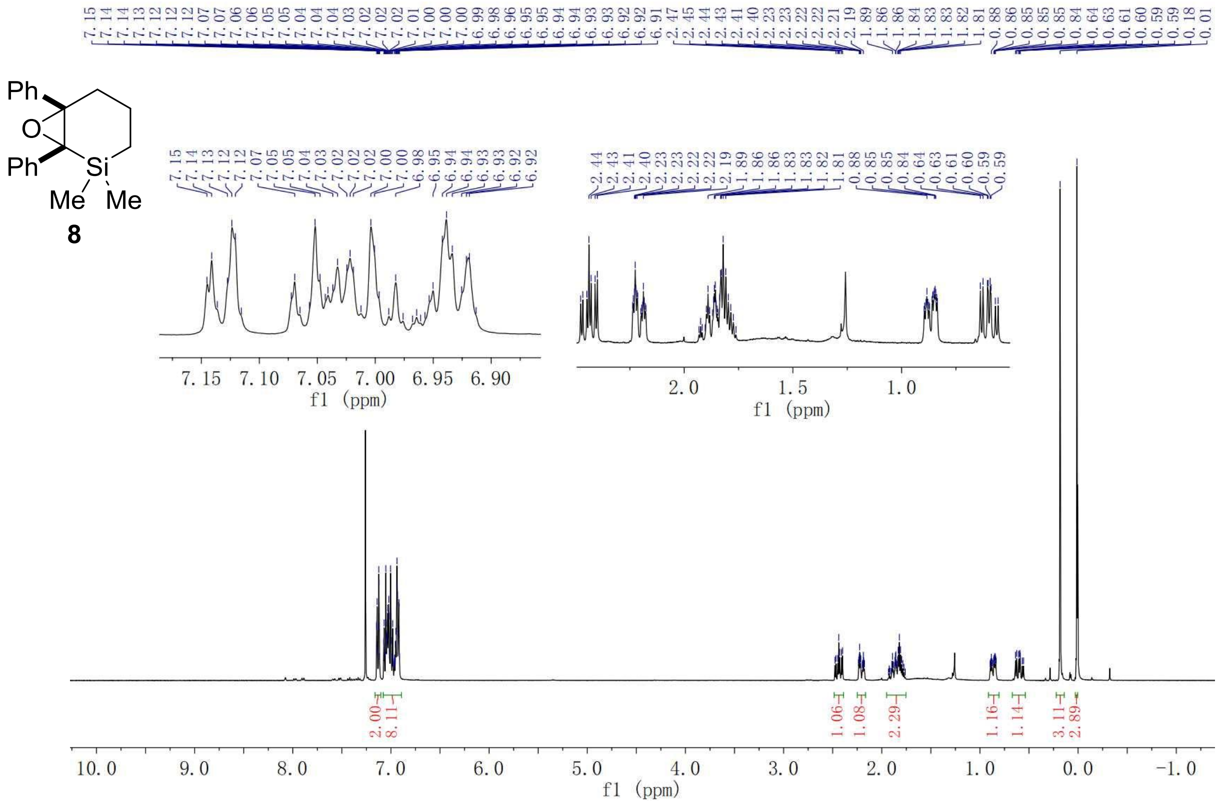

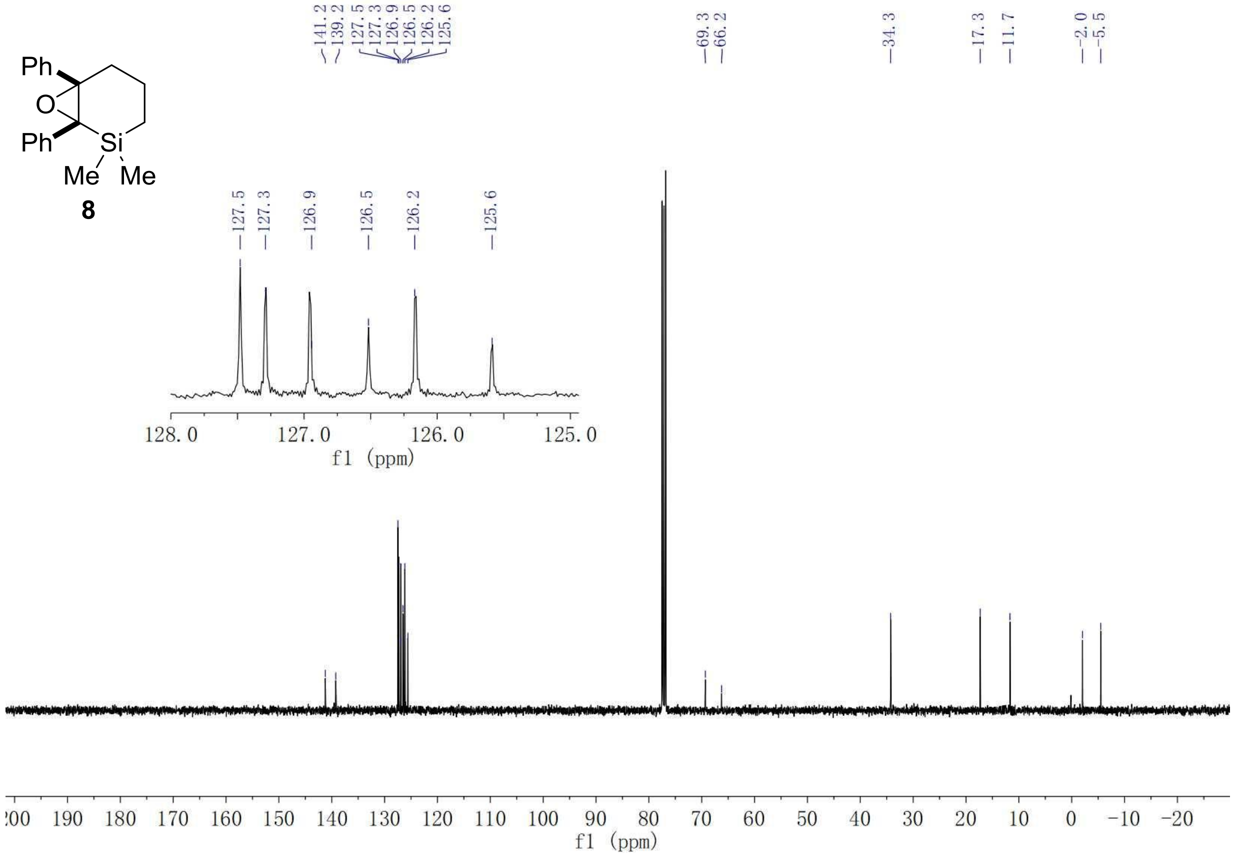

Supplementary Figure 107 ^{1}H and ^{13}C NMR Spectra for compound 8

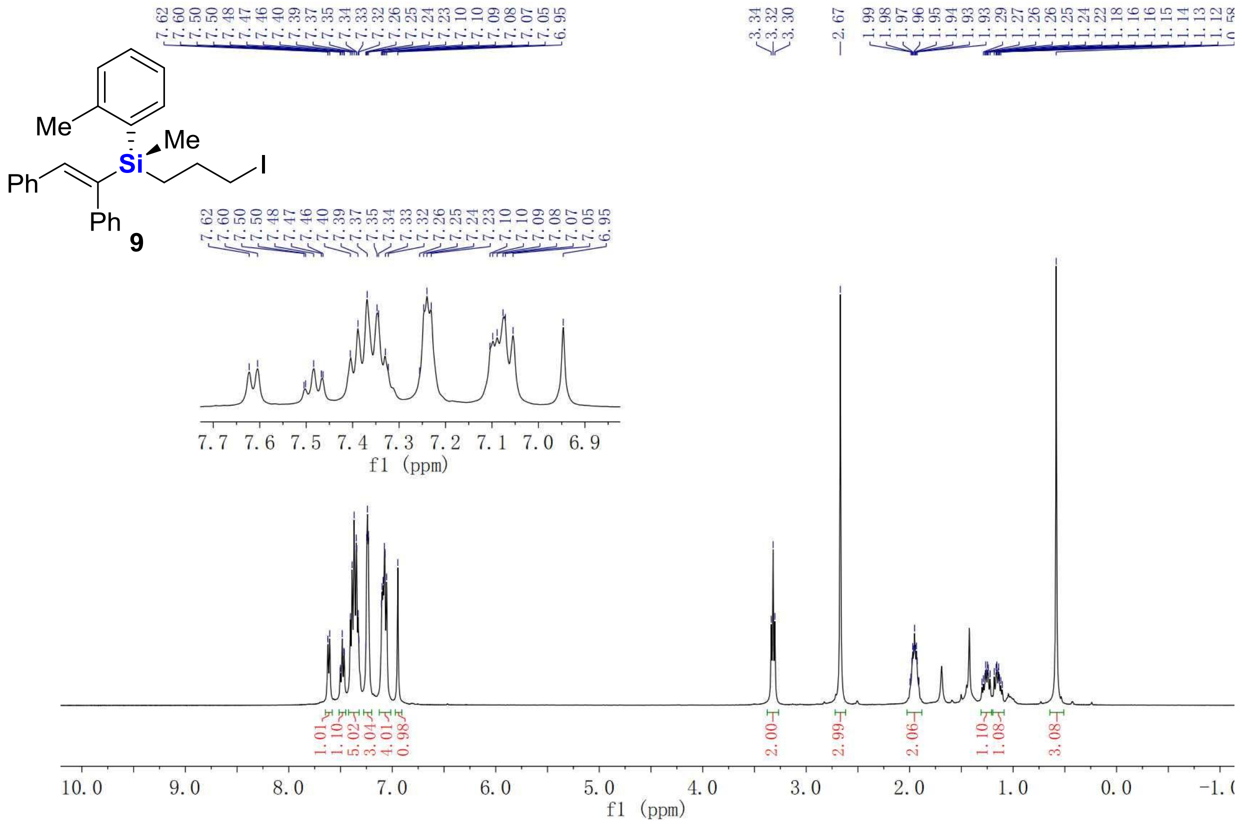

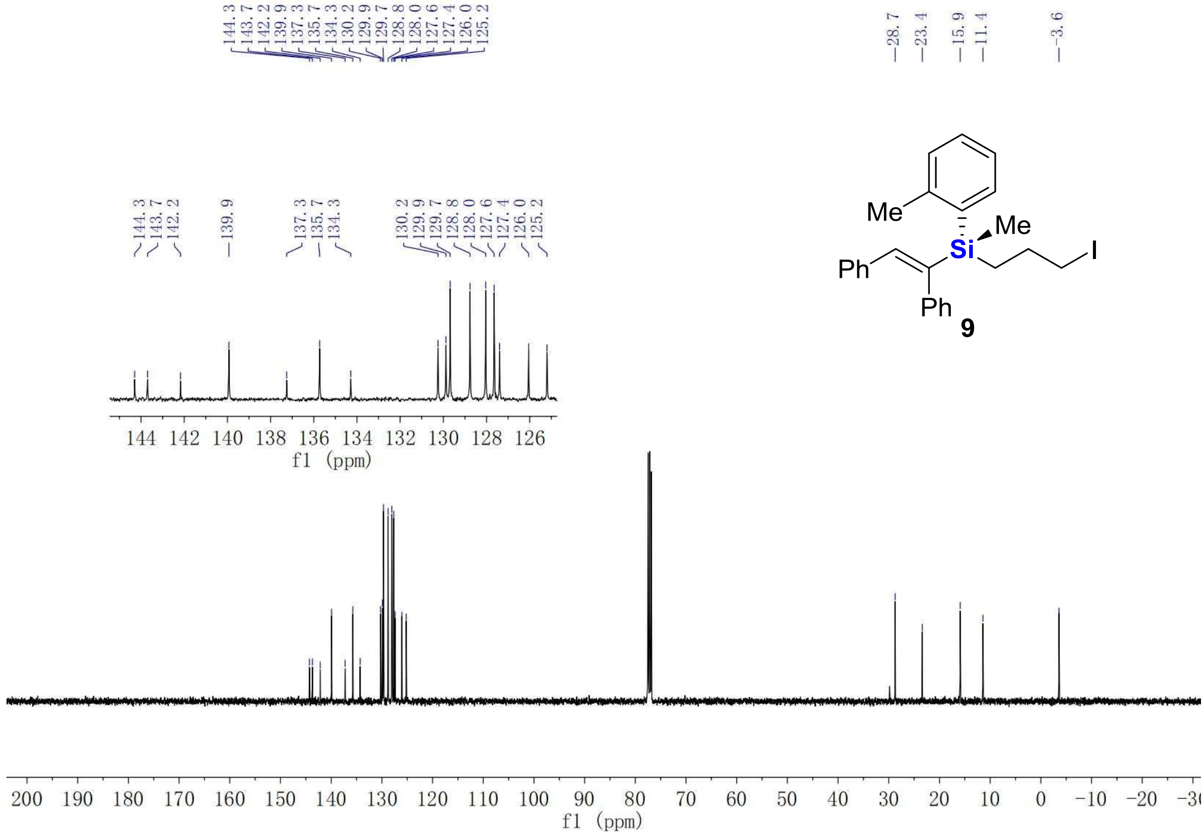

Supplementary Figure 108 ^{1}H and ^{13}C NMR Spectra for compound 9

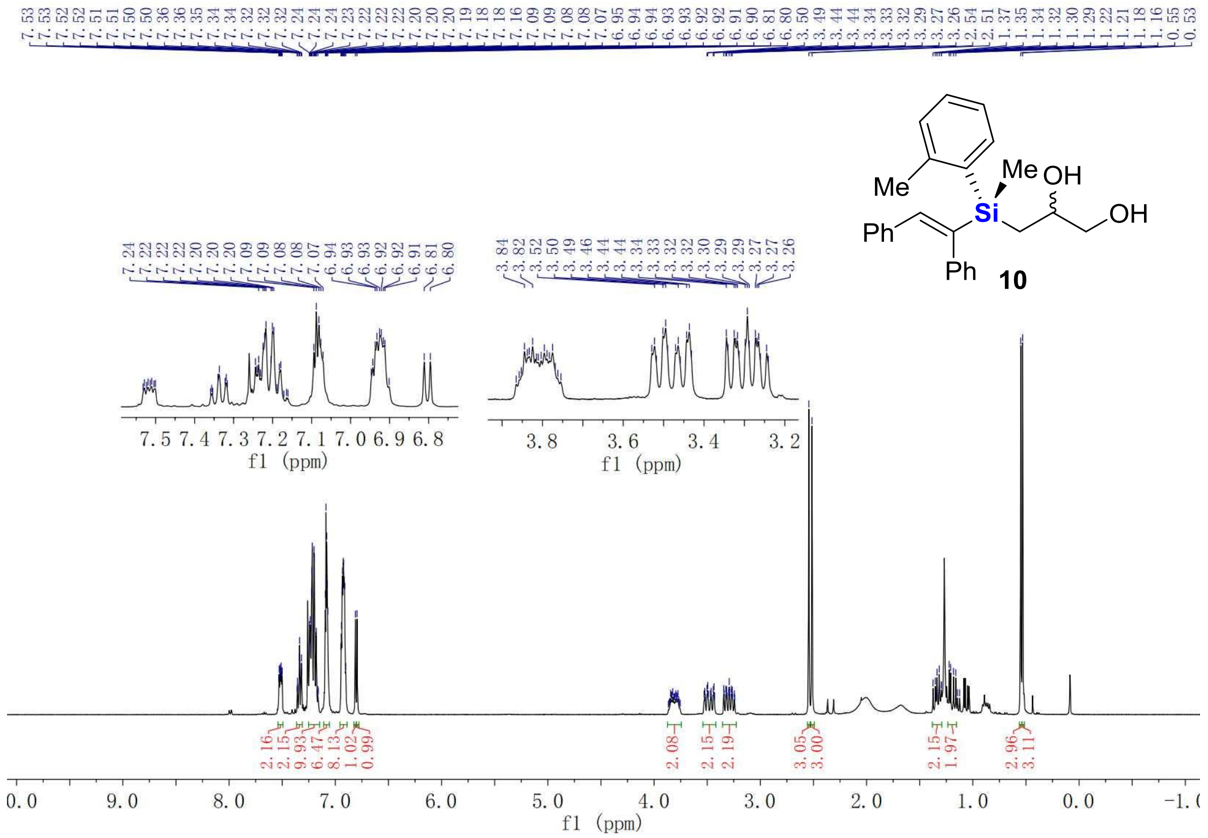

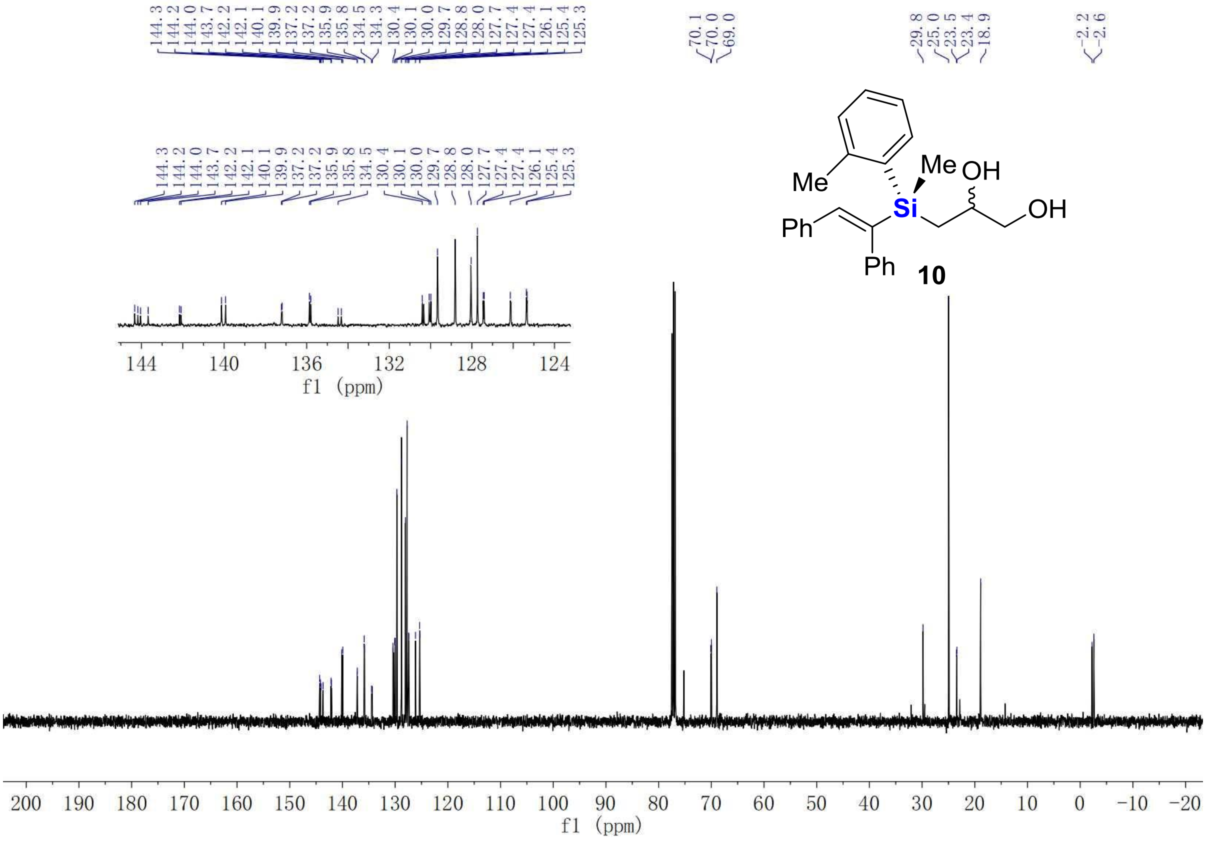

Supplementary Figure 109 ^{1}H and ^{13}C NMR Spectra for compound 10

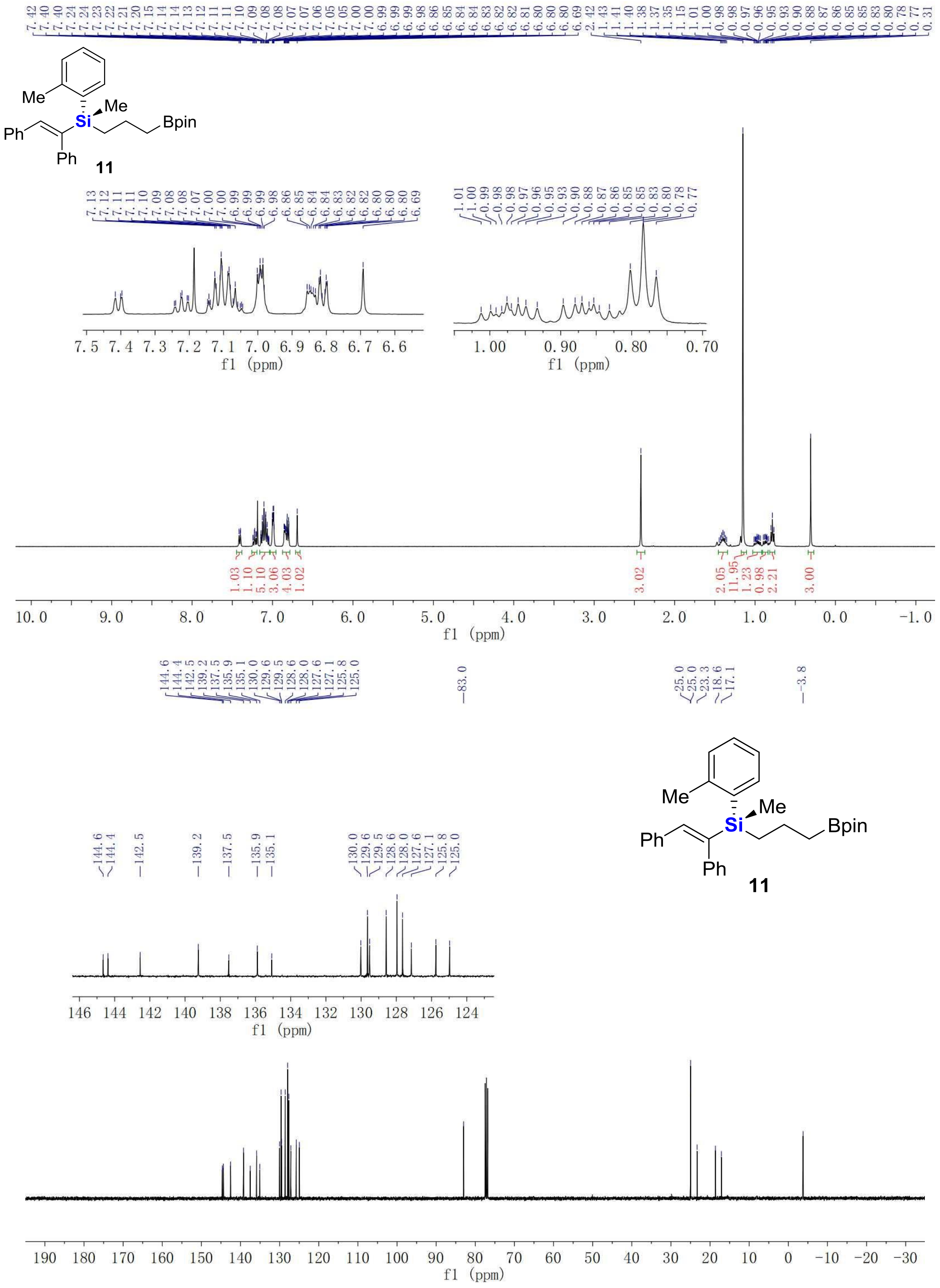

Supplementary Figure 110 ^{1}H and ^{13}C NMR Spectra for compound 11

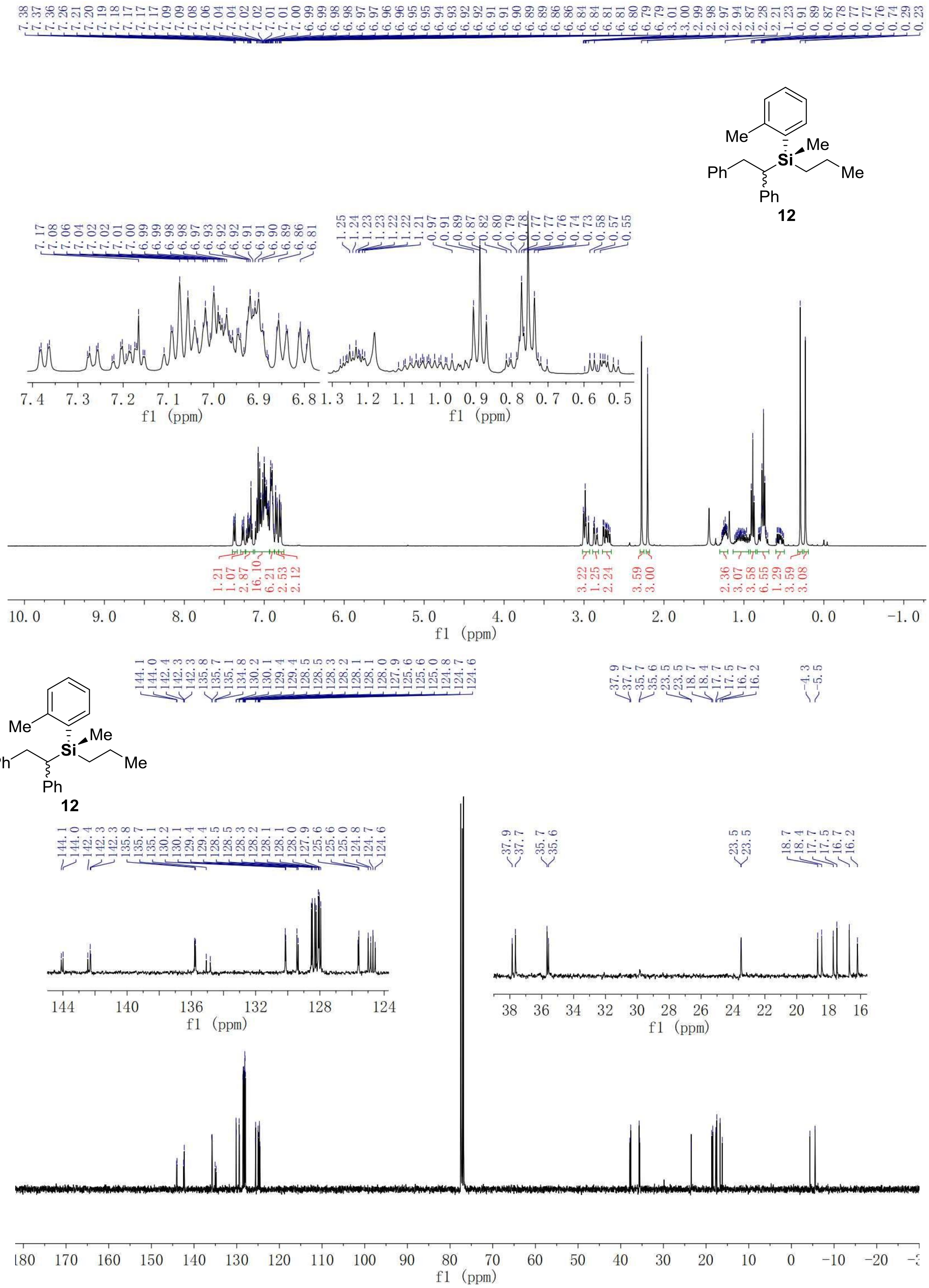

Supplementary Figure 111 ^{1}H and ^{13}C NMR Spectra for compound 12

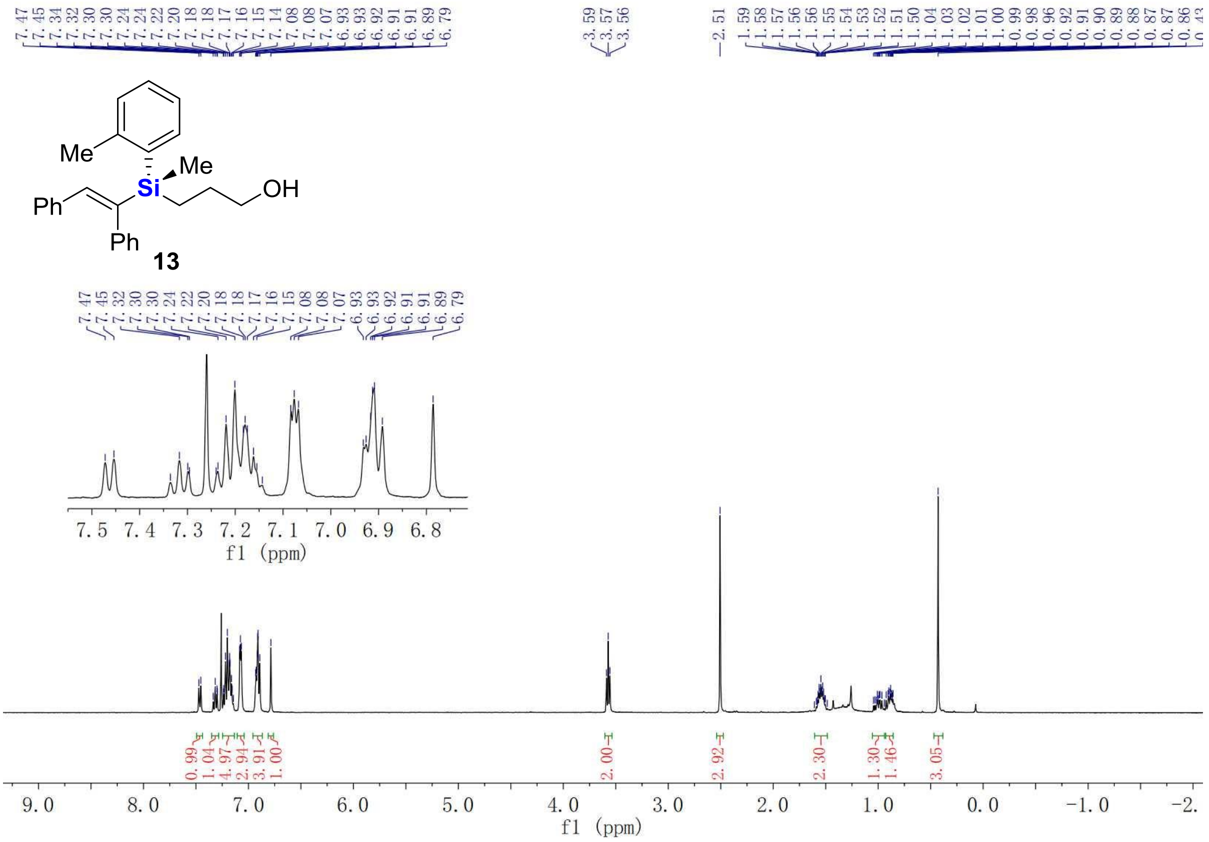

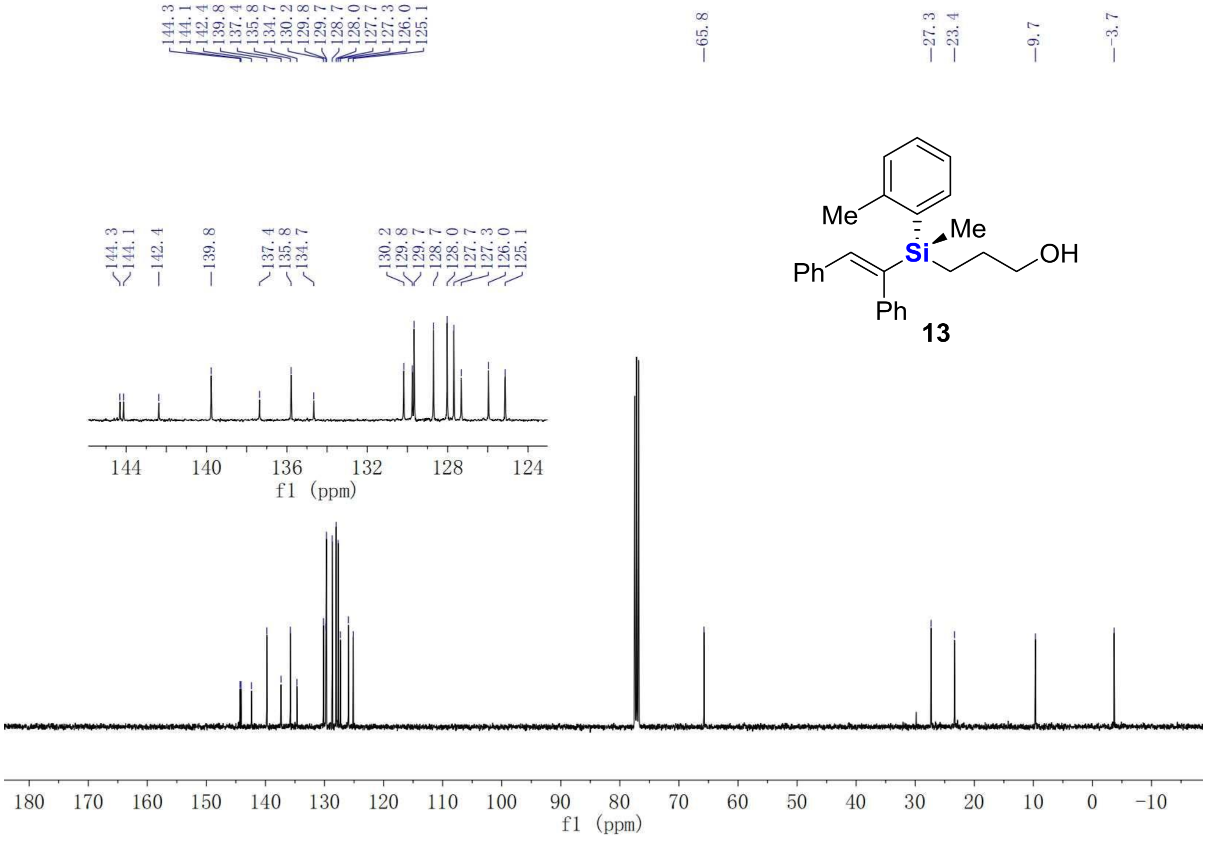

Supplementary Figure 112 ^{1}H and ^{13}C NMR Spectra for compound 13

HPLC charts of chiral products

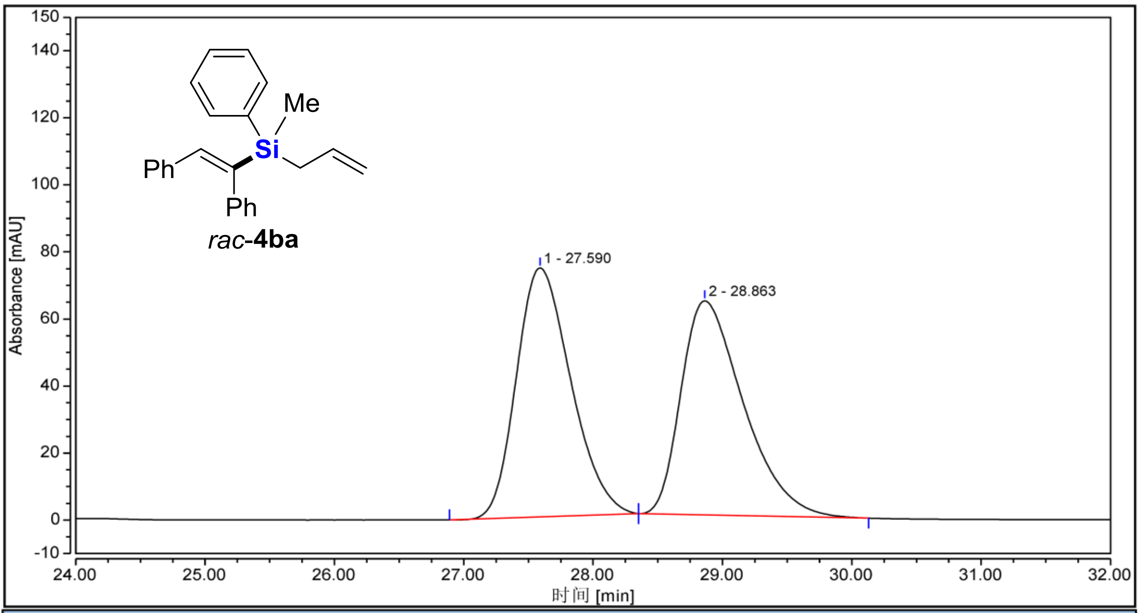

Result

Peak #	Run time [min][	Area [mAU*min]	Height [mAU]	Area %	Height %
1	27.590	36.203	74.274	50.35	53.77
2	28.863	35.698	63.850	49.65	46.23
**Totals:**		**71.901**	**138.124**	**100.00**	**100.00**

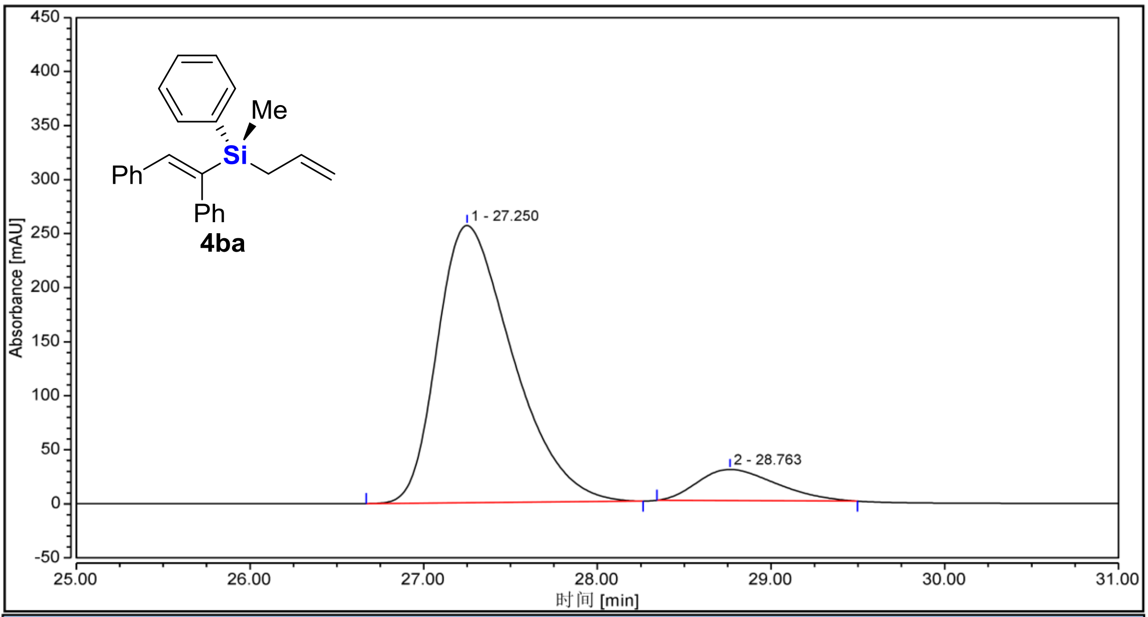

Result

Peak #	Run time [min][	Area [mAU*min]	Height [mAU]	Area %	Height %
1	27.250	129.025	256.437	89.50	89.89
2	28.763	15.137	28.847	10.50	10.11
**Totals:**		**144.162**	**285.285**	**100.00**	**100.00**

Supplementary Figure 113 HPLC of compounds *rac*-4ba and 4ba

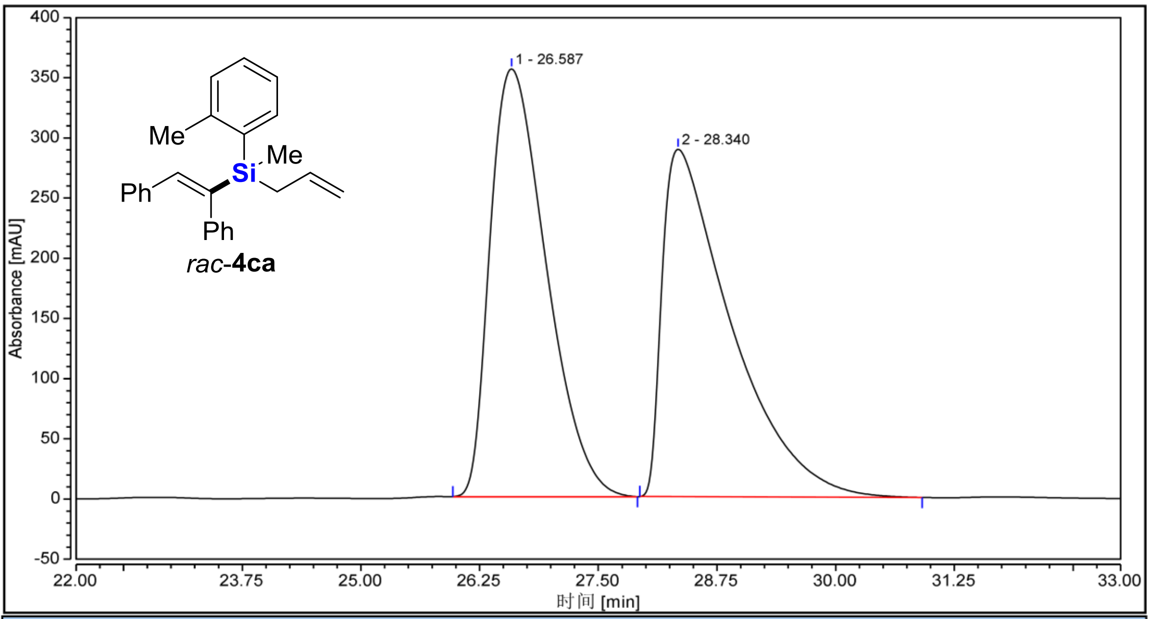

Result

Peak #	Run time [min][	Area [mAU*min]	Height [mAU]	Area %	Height %
1	26.587	243.742	355.301	50.09	55.18
2	28.340	242.859	288.626	49.91	44.82
**Totals:**		**486.600**	**643.927**	**100.00**	**100.00**

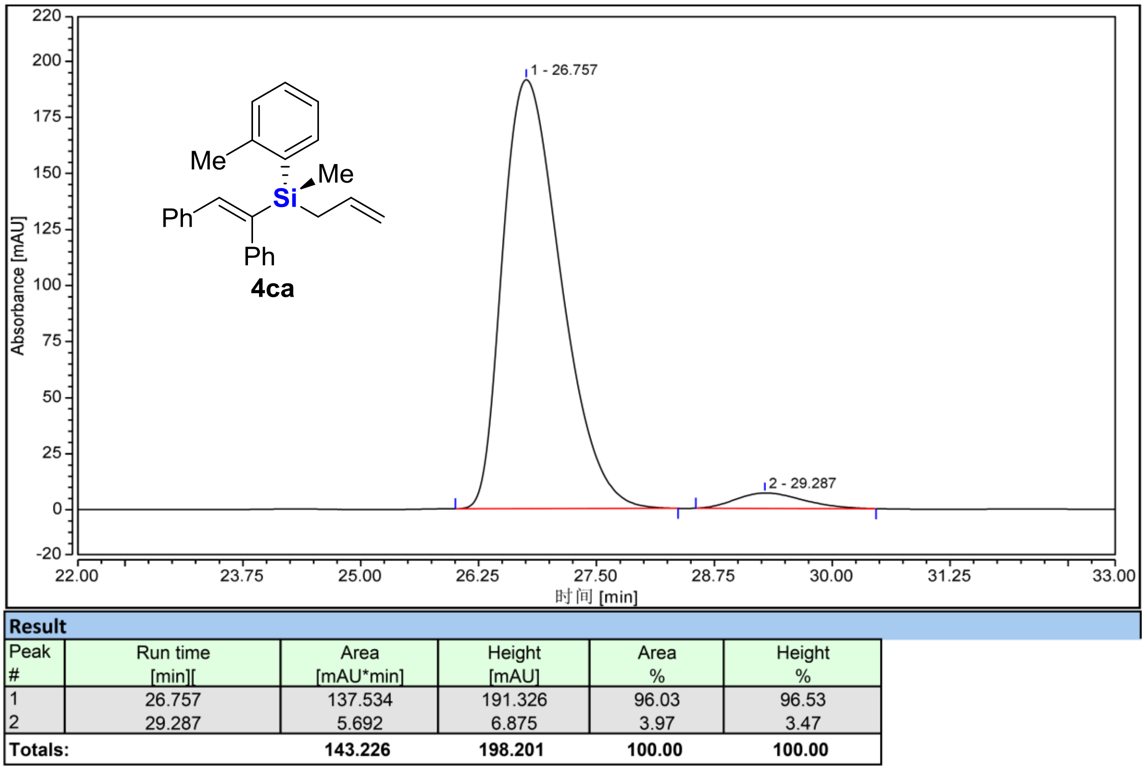

Result

Peak #	Run time [min][	Area [mAU*min]	Height [mAU]	Area %	Height %
1	26.757	137.534	191.326	96.03	96.53
2	29.287	5.692	6.875	3.97	3.47
**Totals:**		**143.226**	**198.201**	**100.00**	**100.00**

Supplementary Figure 114 HPLC of compounds *rac*-4ca and 4ca

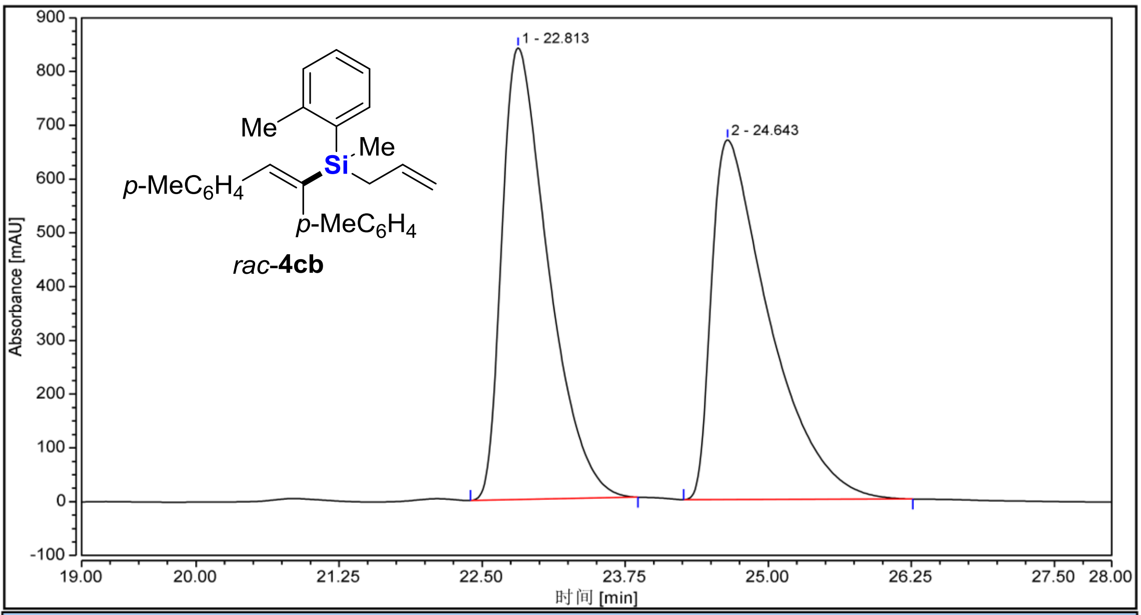

Result

Peak #	Run time [min][	Area [mAU*min]	Height [mAU]	Area %	Height %
1	22.813	394.436	840.116	50.09	55.68
2	24.643	392.947	668.791	49.91	44.32
**Totals:**		**787.383**	**1508.907**	**100.00**	**100.00**

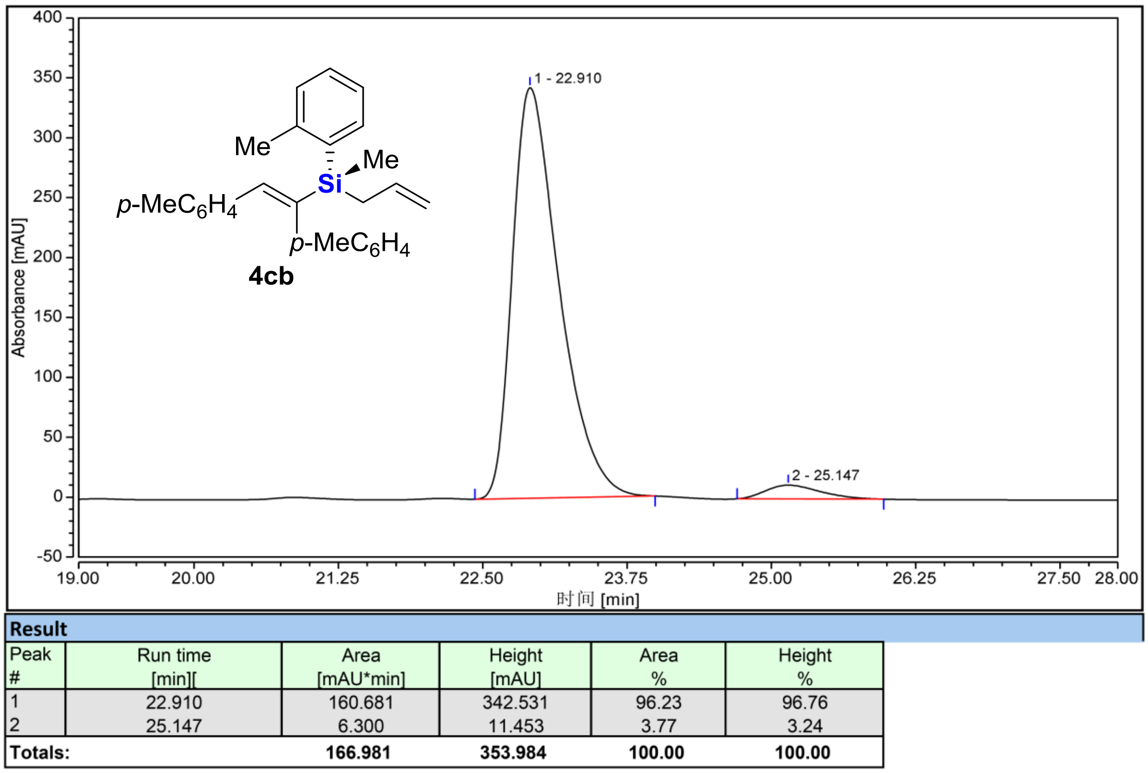

Result

Peak #	Run time [min][	Area [mAU*min]	Height [mAU]	Area %	Height %
1	22.910	160.681	342.531	96.23	96.76
2	25.147	6.300	11.453	3.77	3.24
**Totals:**		**166.981**	**353.984**	**100.00**	**100.00**

Supplementary Figure 115 HPLC of compounds *rac*-4cb and 4cb

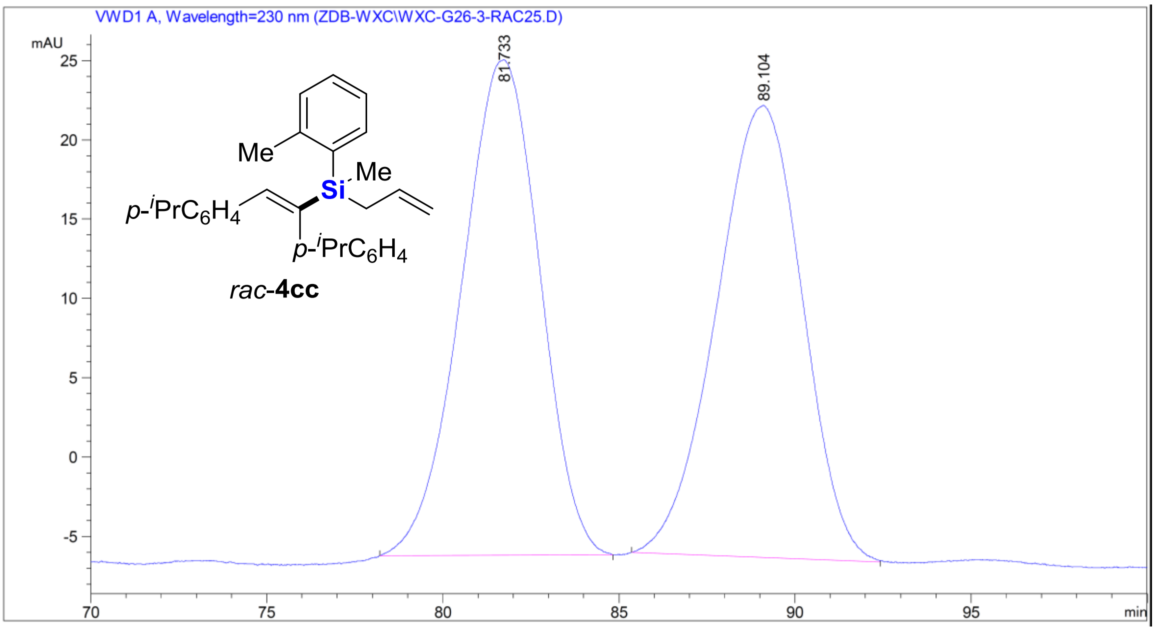

Peak #	RetTime [min]	Type	Width [min]	Area mAU *s	Height [mAU]	Area %
1	81.733	BB	1.8284	4817.24512	31.23560	49.9144
2	89.104	BV	1.9999	4833.75879	28.48883	50.0856

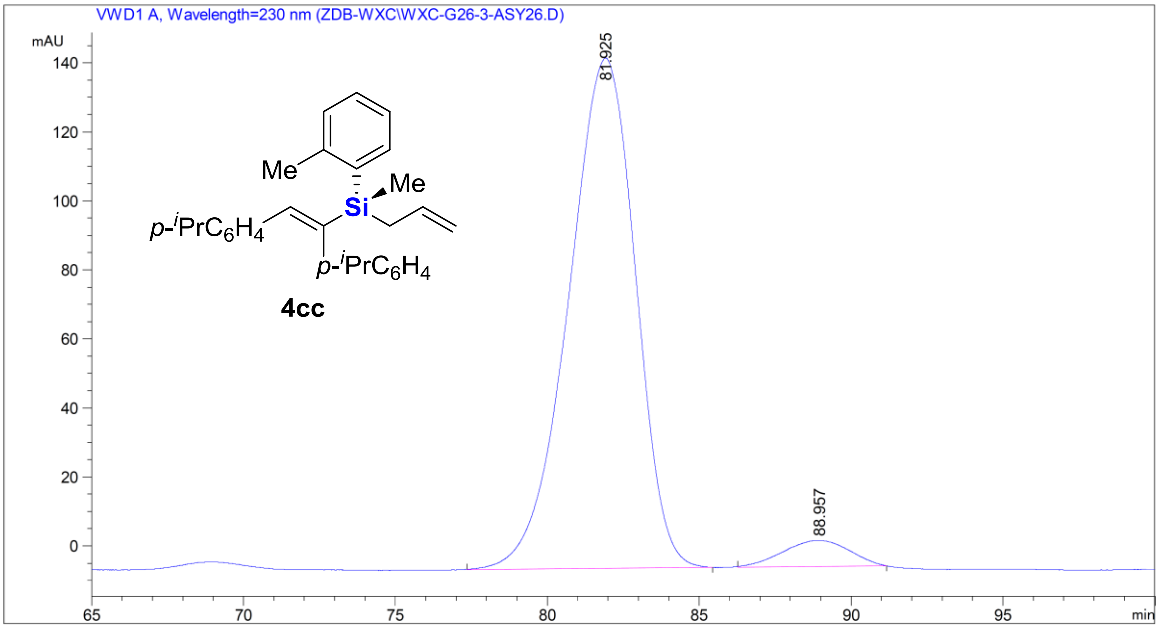

Peak #	RetTime [min]	Type	Width [min]	Area mAU *s	Height [mAU]	Area %
1	81.925	MM R	2.5495	2.26276e4	147.92081	95.1328
2	88.957	MM R	2.5460	1157.66919	7.57848	4.8672

Supplementary Figure 116 HPLC of compounds *rac*-4cc and 4cc

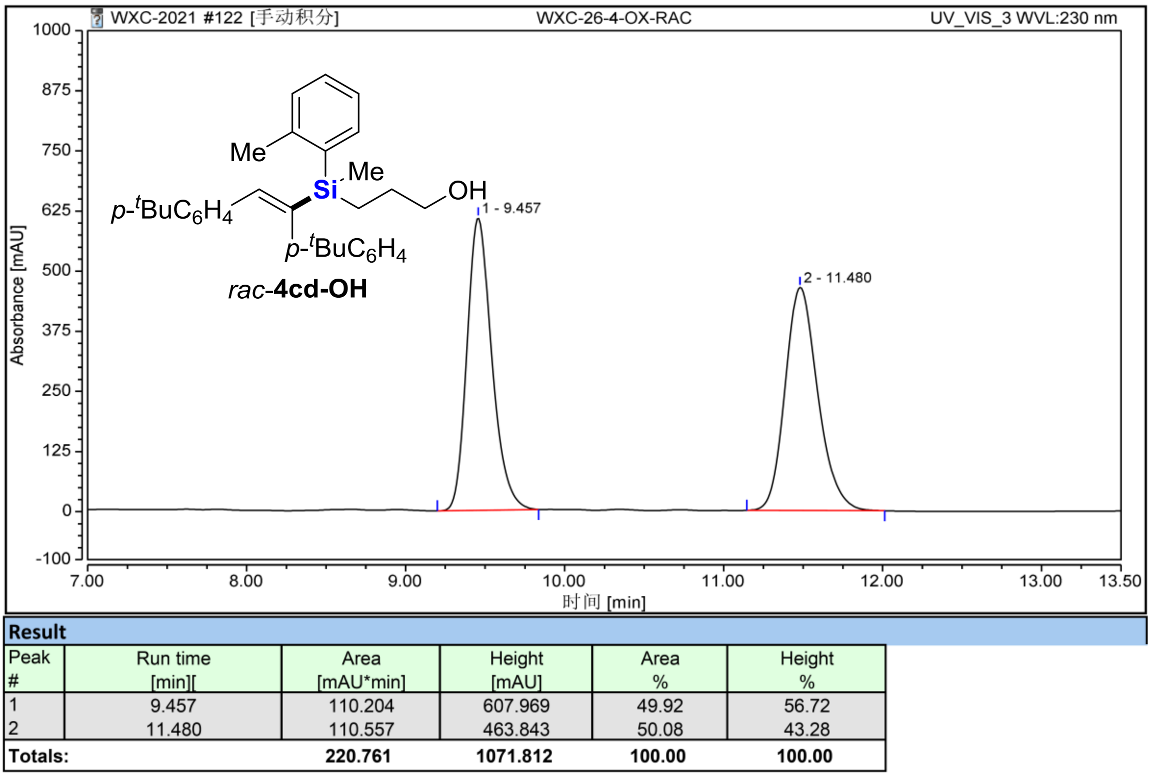

Result					
Peak #	Run time [min][	Area [mAU*min]	Height [mAU]	Area %	Height %
1	9.457	110.204	607.969	49.92	56.72
2	11.480	110.557	463.843	50.08	43.28
**Totals:**		**220.761**	**1071.812**	**100.00**	**100.00**

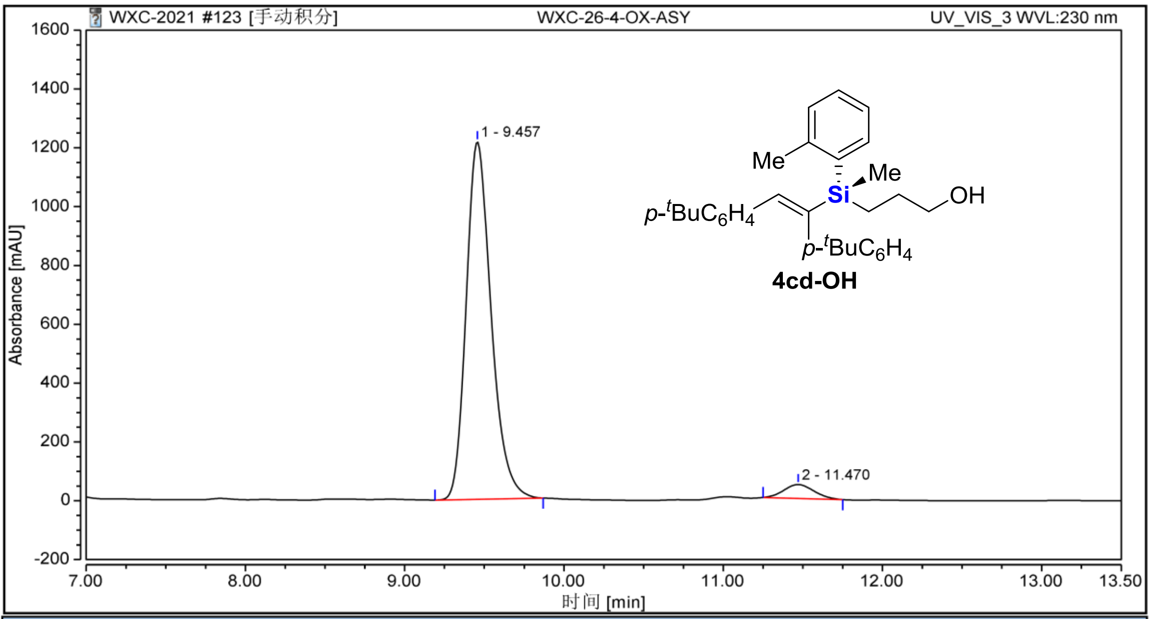

Result					
Peak #	Run time [min][	Area [mAU*min]	Height [mAU]	Area %	Height %
1	9.457	220.726	1215.670	95.54	96.30
2	11.470	10.301	46.756	4.46	3.70
**Totals:**		**231.027**	**1262.426**	**100.00**	**100.00**

Supplementary Figure 117 HPLC of compounds *rac*-4cd-OH and 4cd-OH

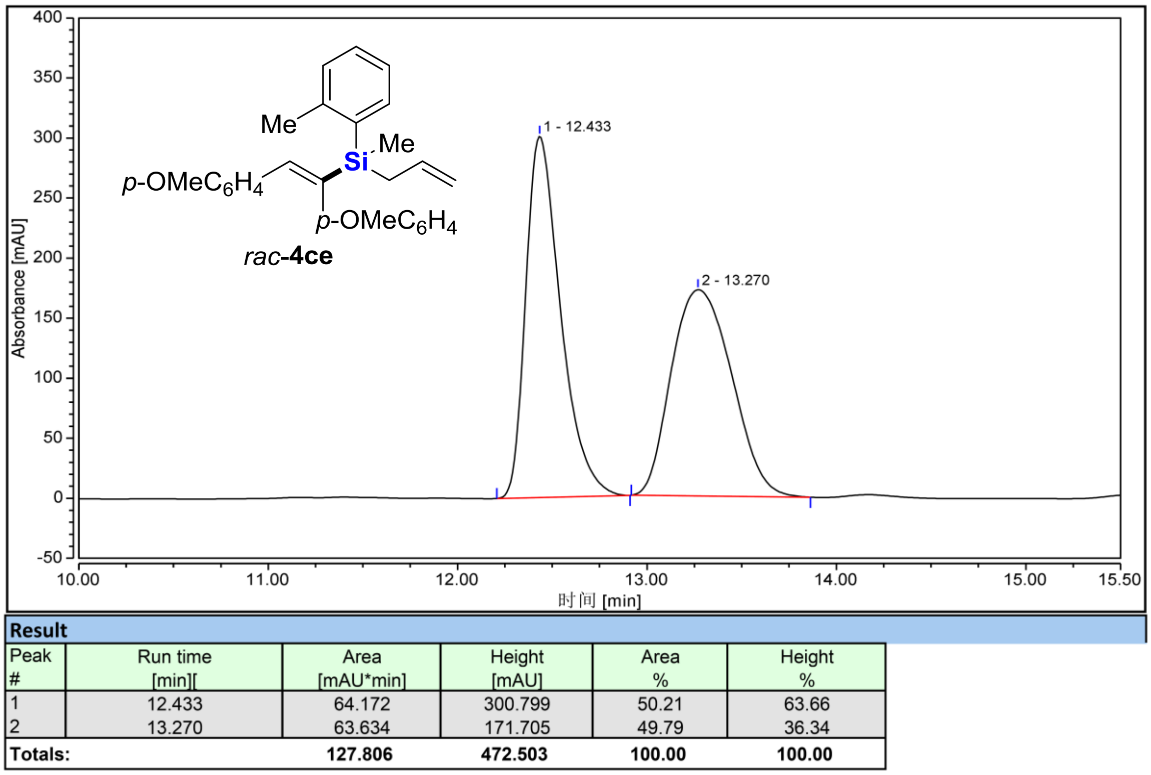

Result

Peak #	Run time [min][	Area [mAU*min]	Height [mAU]	Area %	Height %
1	12.433	64.172	300.799	50.21	63.66
2	13.270	63.634	171.705	49.79	36.34
**Totals:**		**127.806**	**472.503**	**100.00**	**100.00**

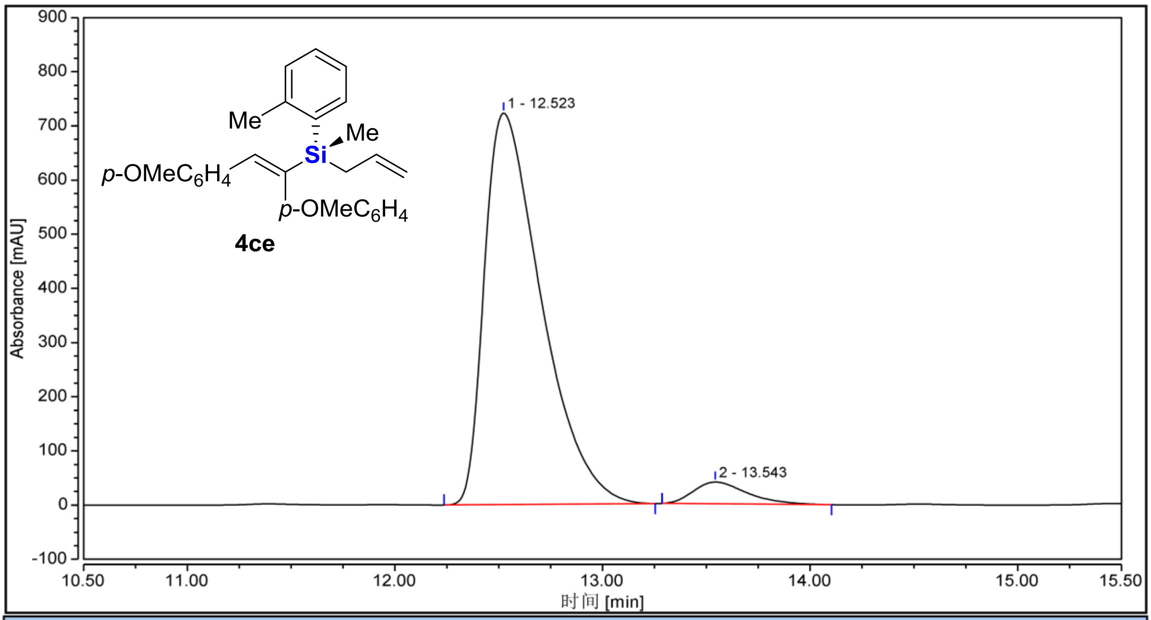

Result

Peak #	Run time [min][	Area [mAU*min]	Height [mAU]	Area %	Height %
1	12.523	230.055	722.895	95.01	94.73
2	13.543	12.091	40.244	4.99	5.27
**Totals:**		**242.146**	**763.139**	**100.00**	**100.00**

Supplementary Figure 118 HPLC of compounds *rac*-4ce and 4ce

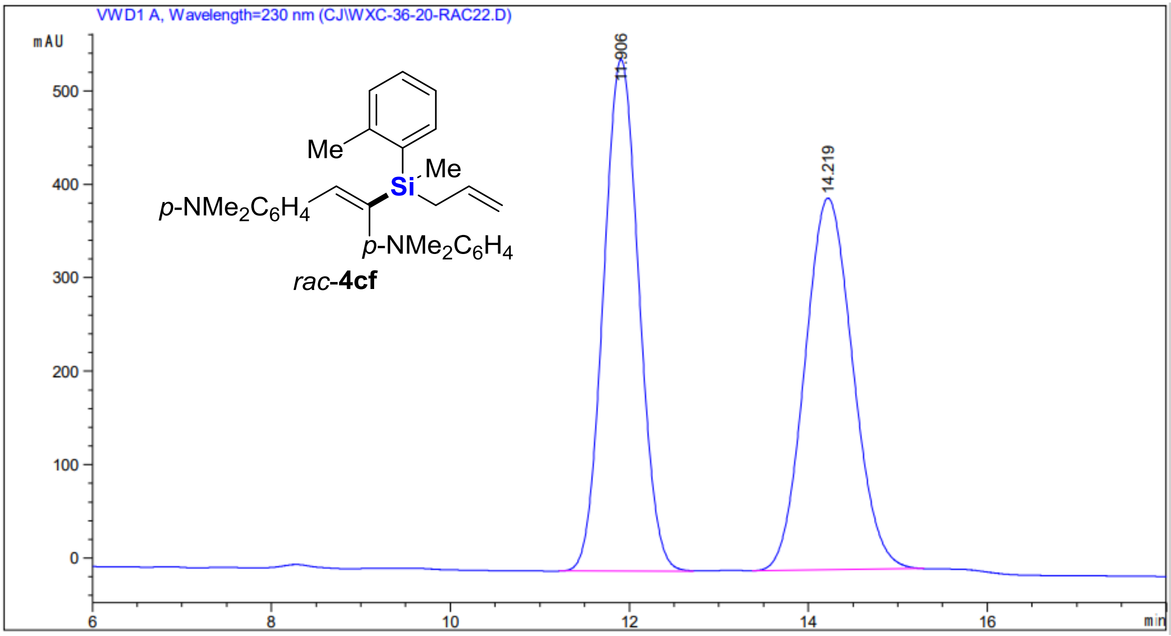

Peak #	RetTime [min]	Type	Width [min]	Area mAU *s	Height [mAU]	Area %
1	11.906	MM R	0.4573	1.50247e4	547.60248	50.4151
2	14.219	MM R	0.6192	1.47773e4	397.73529	49.5849

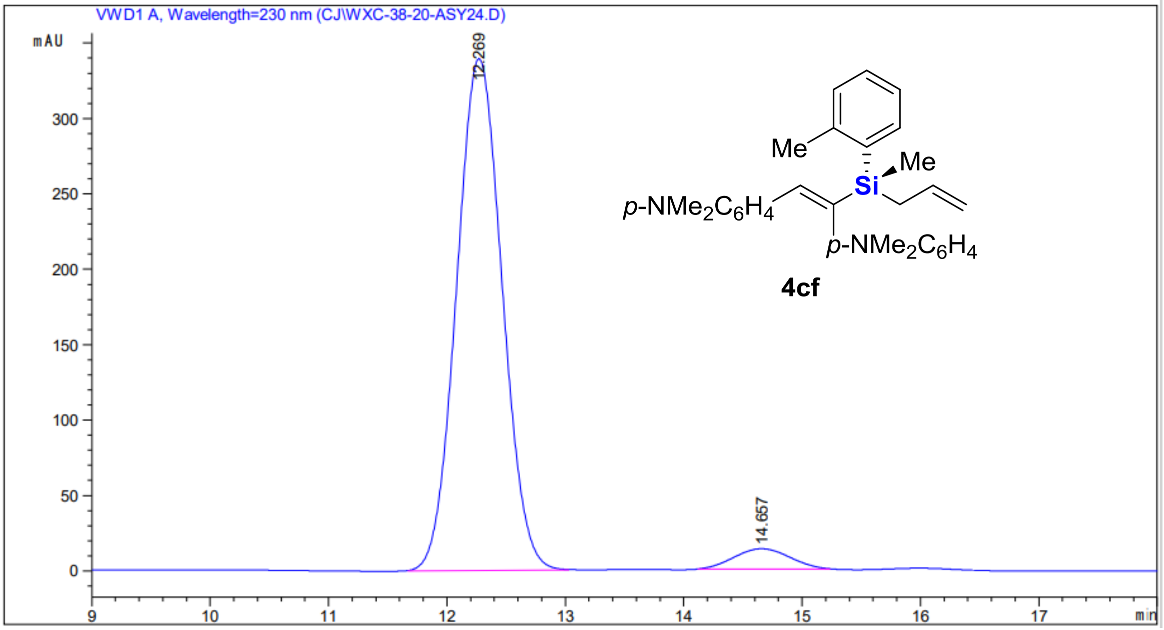

Peak #	RetTime [min]	Type	Width [min]	Area mAU *s	Height [mAU]	Area %
1	12.269	MM R	0.4454	9072.07031	339.47787	95.3700
2	14.657	MM R	0.5451	440.43112	13.46743	4.6300

Supplementary Figure 119 HPLC of compounds *rac*-4cf and 4cf

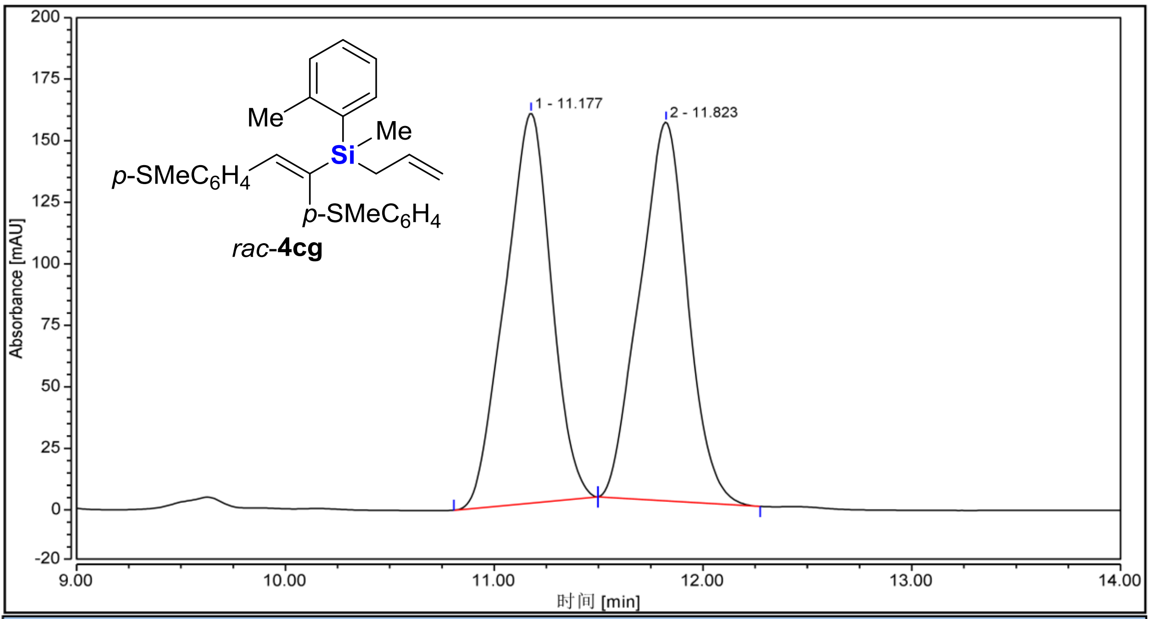

Result

Peak #	Run time [min][	Area [mAU*min]	Height [mAU]	Area %	Height %
1	11.177	40.772	158.337	49.85	50.72
2	11.823	41.025	153.830	50.15	49.28
**Totals:**		**81.798**	**312.167**	**100.00**	**100.00**

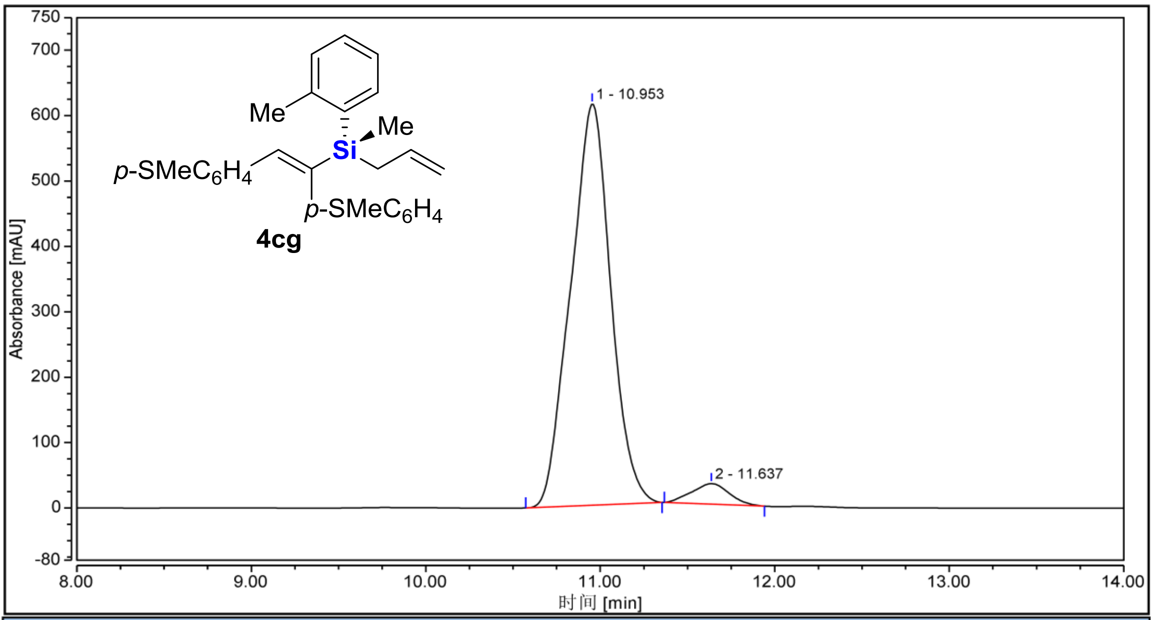

Result

Peak #	Run time [min][	Area [mAU*min]	Height [mAU]	Area %	Height %
1	10.953	163.376	613.831	95.41	95.12
2	11.637	7.868	31.478	4.59	4.88
**Totals:**		**171.245**	**645.310**	**100.00**	**100.00**

Supplementary Figure 120 HPLC of compounds *rac*-4cg and 4cg

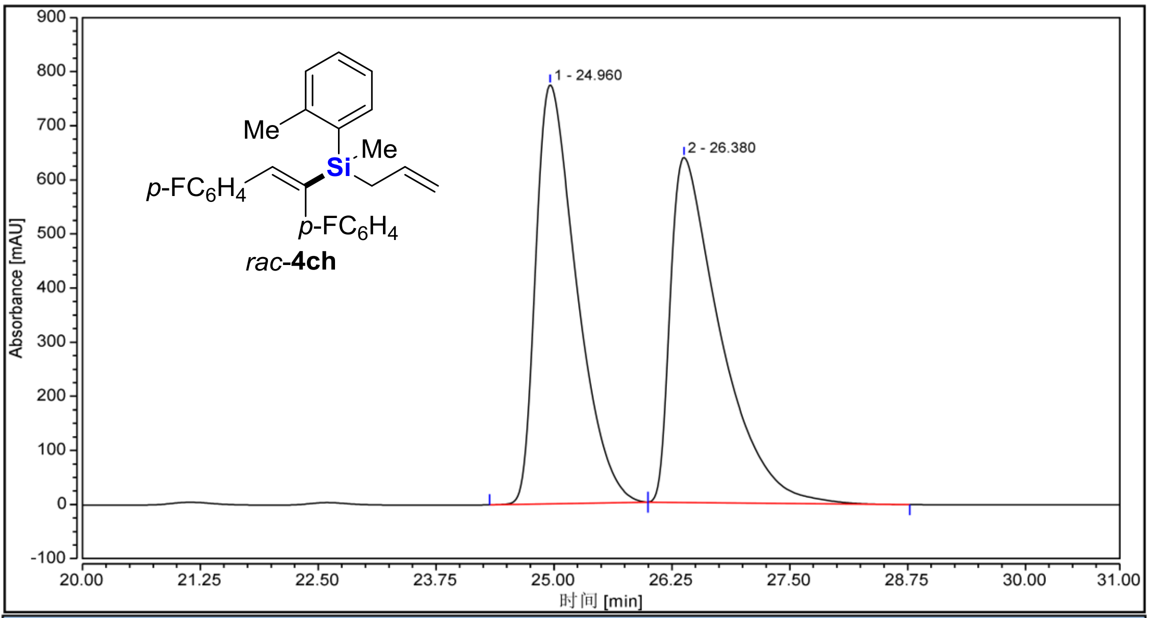

Result

Peak #	Run time [min][	Area [mAU*min]	Height [mAU]	Area %	Height %
1	24.960	388.124	774.190	49.79	54.84
2	26.380	391.411	637.510	50.21	45.16
**Totals:**		**779.535**	**1411.699**	**100.00**	**100.00**

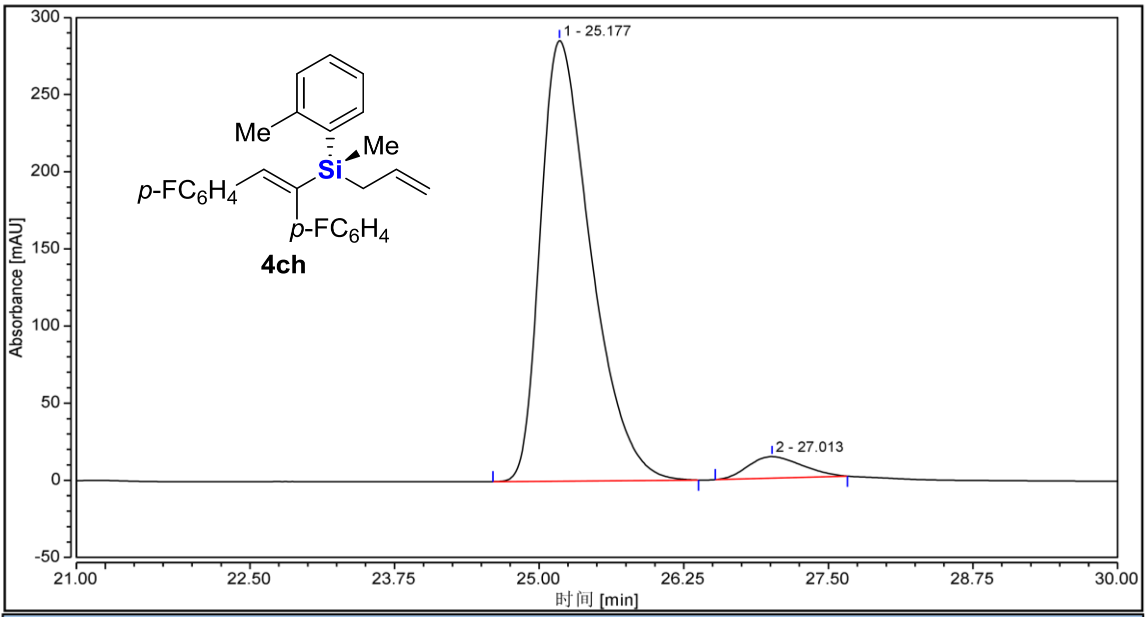

Result

Peak #	Run time [min][	Area [mAU*min]	Height [mAU]	Area %	Height %
1	25.177	144.454	285.584	95.06	95.34
2	27.013	7.507	13.963	4.94	4.66
**Totals:**		**151.961**	**299.547**	**100.00**	**100.00**

Supplementary Figure 121 HPLC of compounds *rac*-4ch and 4ch

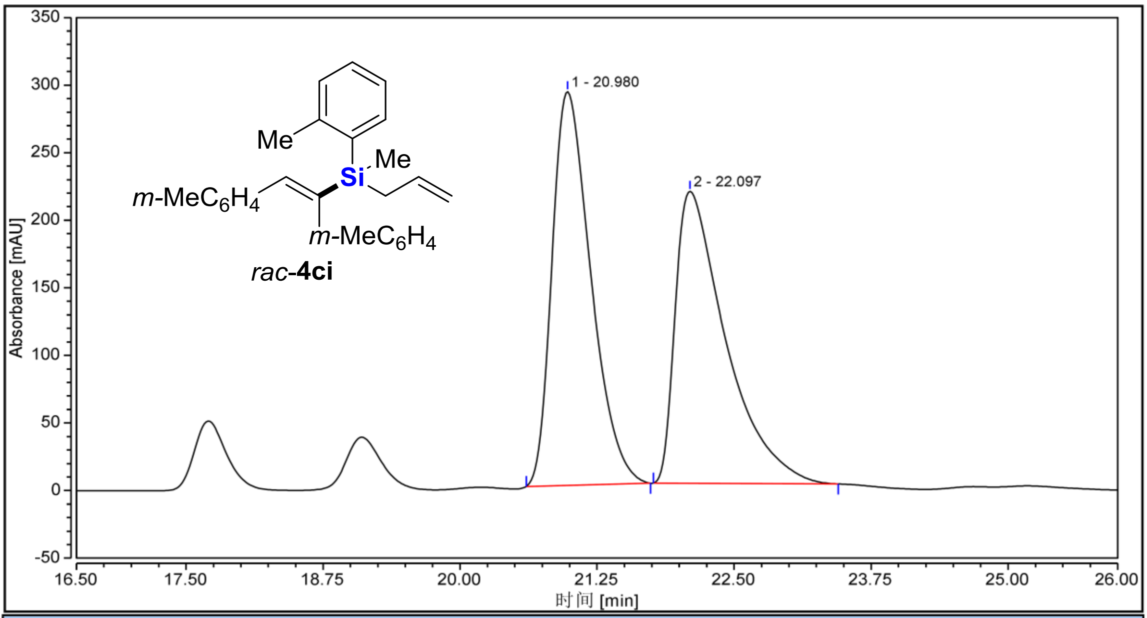

Result

Peak #	Run time [min][	Area [mAU*min]	Height [mAU]	Area %	Height %
1	20.980	119.515	291.270	50.97	57.42
2	22.097	114.970	215.972	49.03	42.58
**Totals:**		**234.485**	**507.242**	**100.00**	**100.00**

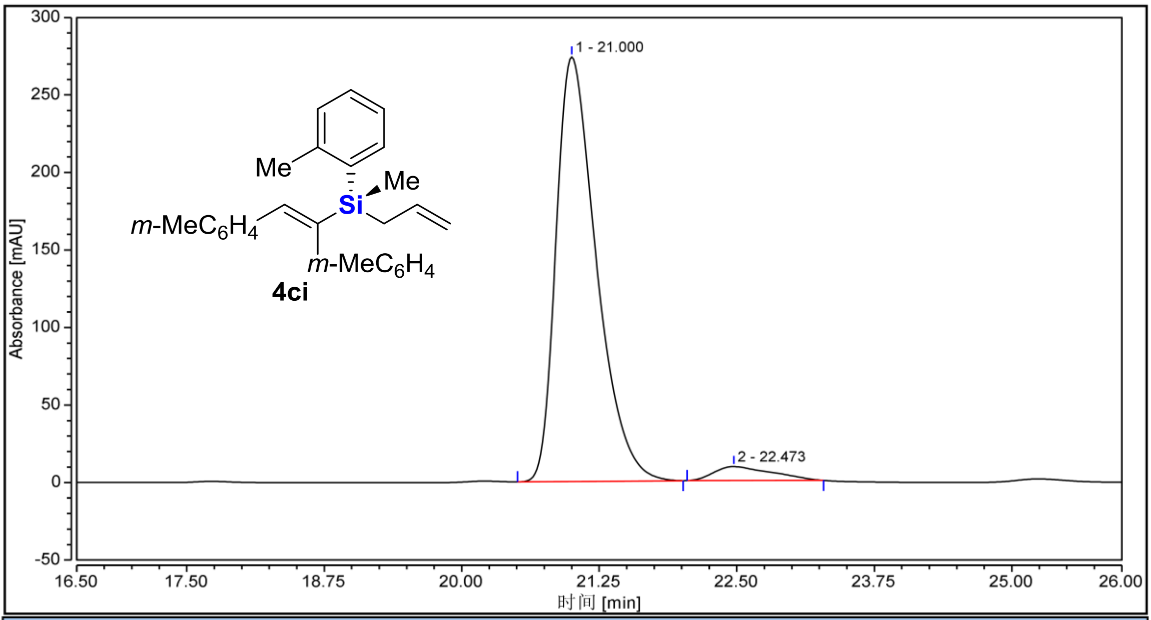

Result

Peak #	Run time [min][	Area [mAU*min]	Height [mAU]	Area %	Height %
1	21.000	116.213	273.888	95.50	96.82
2	22.473	5.473	8.993	4.50	3.18
**Totals:**		**121.687**	**282.882**	**100.00**	**100.00**

Supplementary Figure 122 HPLC of compounds *rac*-4ci and 4ci

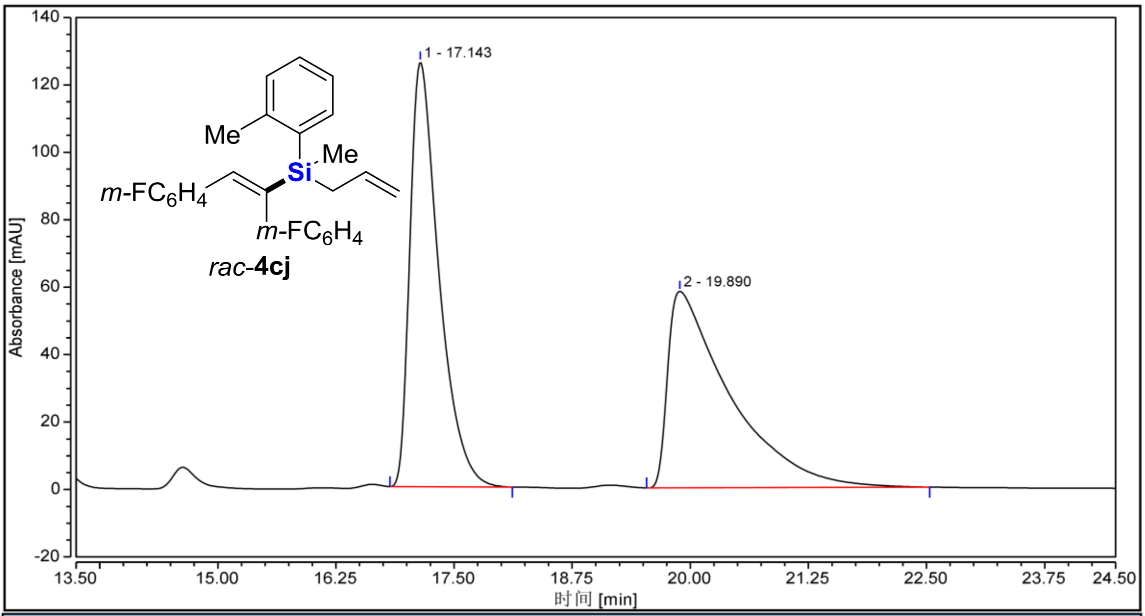

Result

Peak #	Run time [min][	Area [mAU*min]	Height [mAU]	Area %	Height %
1	17.143	44.709	125.887	50.19	68.36
2	19.890	44.366	58.268	49.81	31.64
**Totals:**		**89.075**	**184.154**	**100.00**	**100.00**

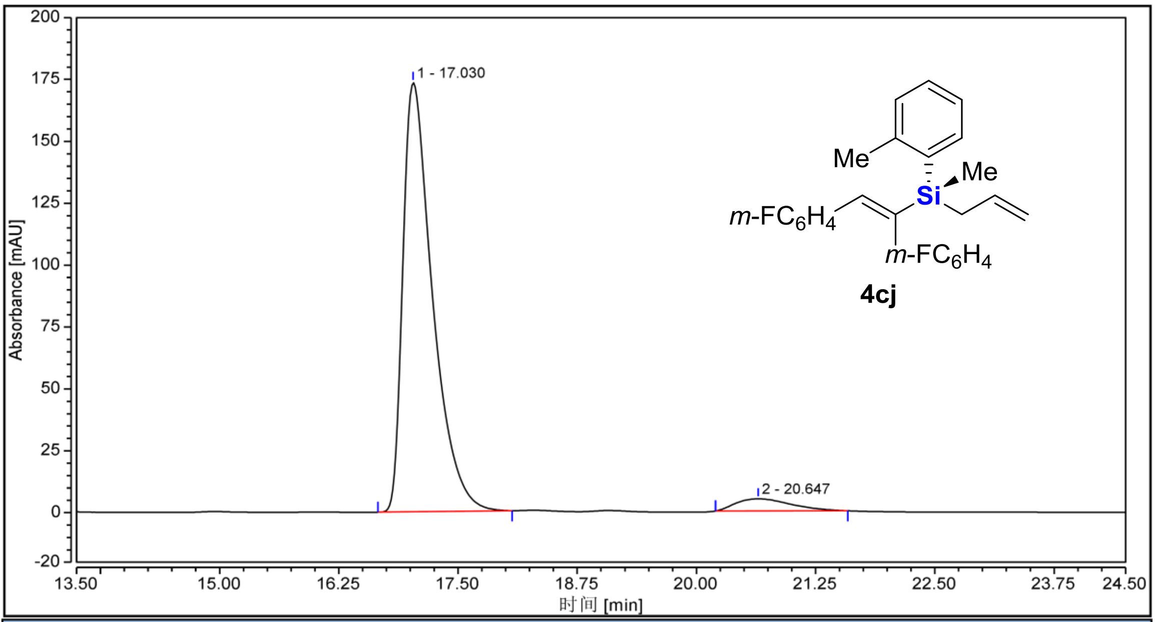

Result

Peak #	Run time [min][	Area [mAU*min]	Height [mAU]	Area %	Height %
1	17.030	62.070	173.352	95.01	97.26
2	20.647	3.262	4.878	4.99	2.74
**Totals:**		**65.332**	**178.230**	**100.00**	**100.00**

Supplementary Figure 123 HPLC of compounds *rac*-4cj and 4cj

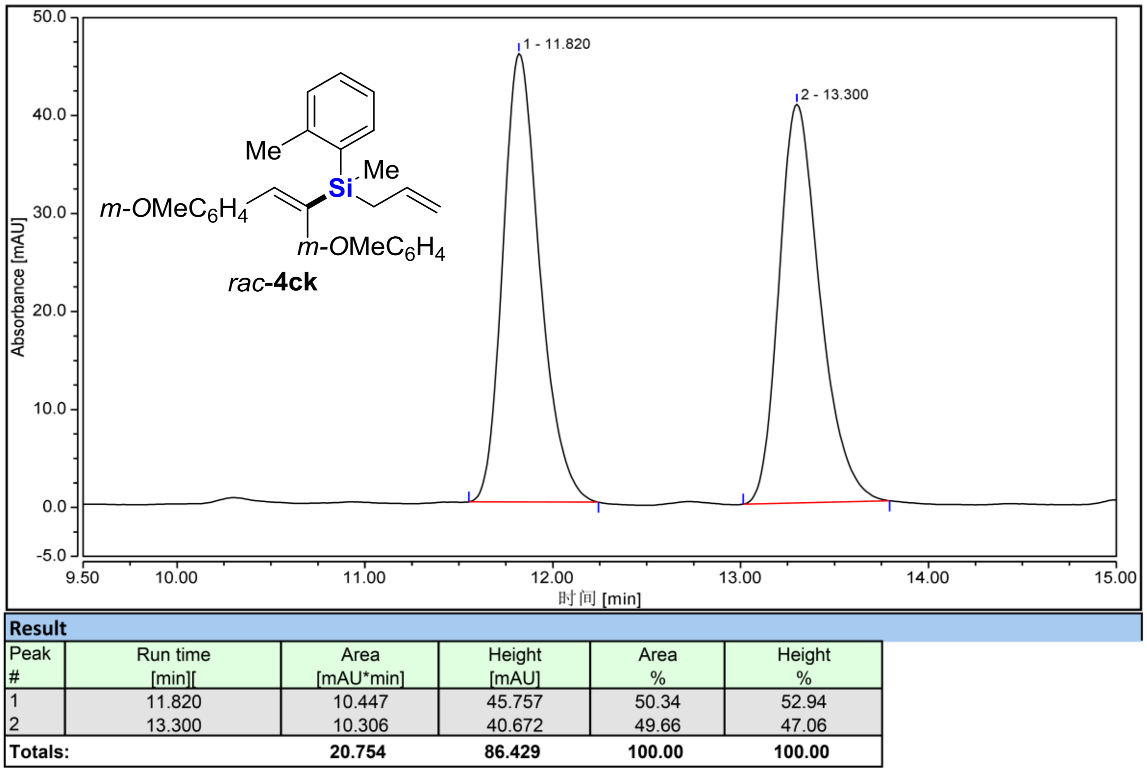

Result

Peak #	Run time [min][	Area [mAU*min]	Height [mAU]	Area %	Height %
1	11.820	10.447	45.757	50.34	52.94
2	13.300	10.306	40.672	49.66	47.06
**Totals:**		**20.754**	**86.429**	**100.00**	**100.00**

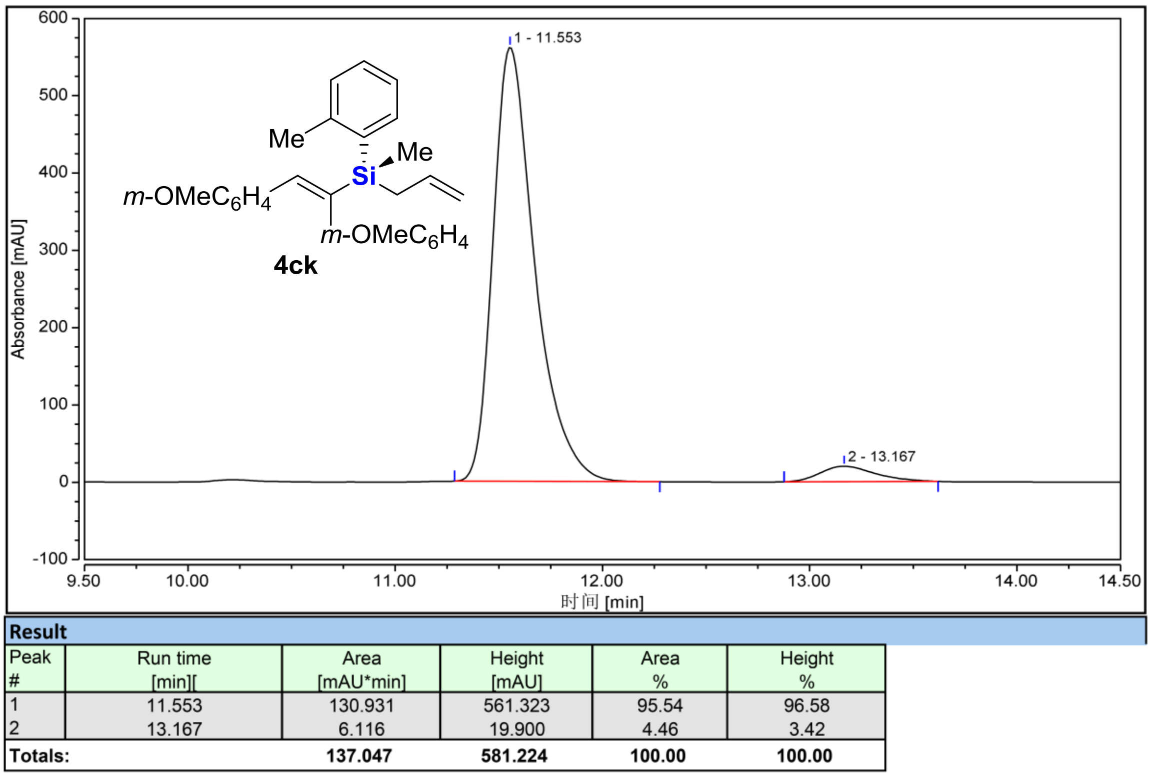

Result

Peak #	Run time [min][	Area [mAU*min]	Height [mAU]	Area %	Height %
1	11.553	130.931	561.323	95.54	96.58
2	13.167	6.116	19.900	4.46	3.42
**Totals:**		**137.047**	**581.224**	**100.00**	**100.00**

Supplementary Figure 124 HPLC of compounds *rac*-4ck and 4ck

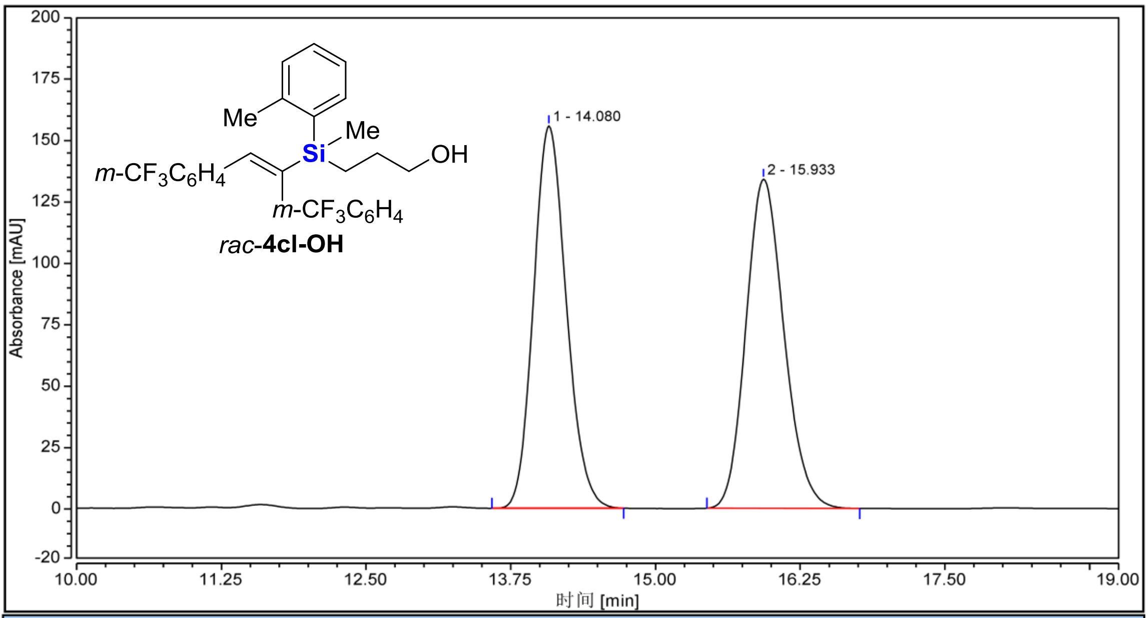

Result

Peak #	Run time [min][	Area [mAU*min]	Height [mAU]	Area %	Height %
1	14.080	51.107	155.615	50.03	53.75
2	15.933	51.036	133.896	49.97	46.25
**Totals:**		**102.143**	**289.511**	**100.00**	**100.00**

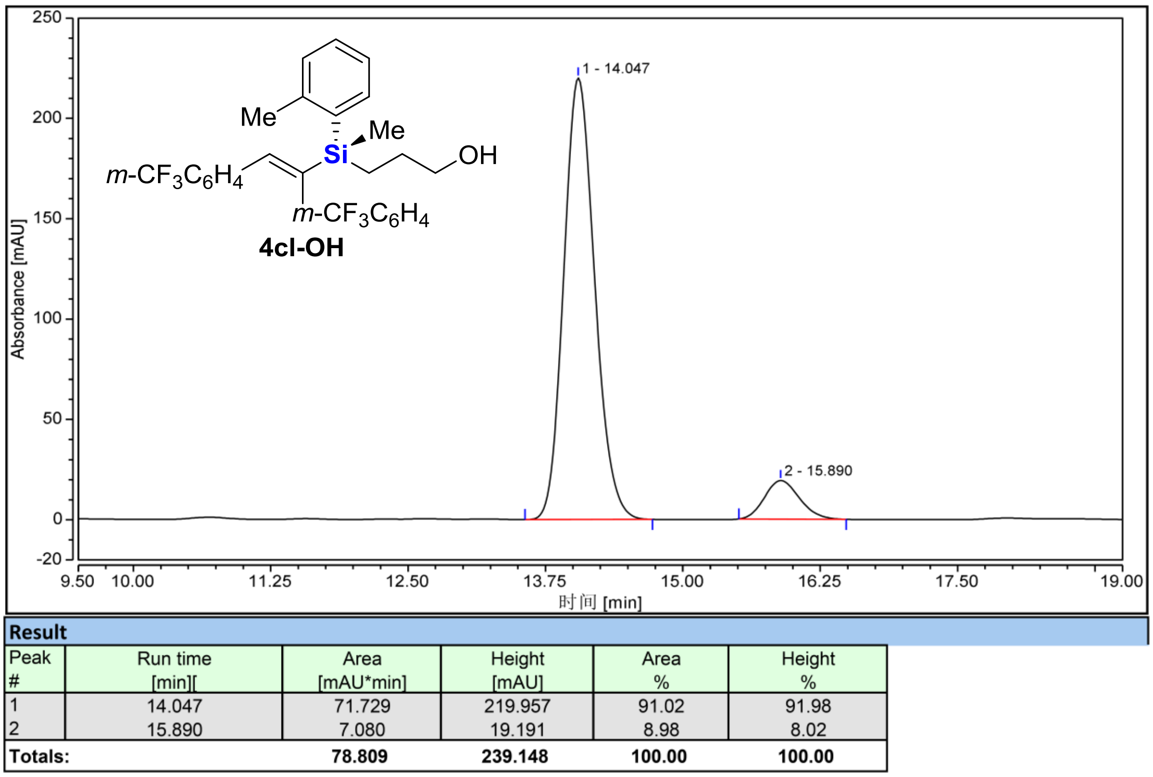

Result

Peak #	Run time [min][	Area [mAU*min]	Height [mAU]	Area %	Height %
1	14.047	71.729	219.957	91.02	91.98
2	15.890	7.080	19.191	8.98	8.02
**Totals:**		**78.809**	**239.148**	**100.00**	**100.00**

Supplementary Figure 125 HPLC of compounds *rac*-4cl-OH and 4cl-OH

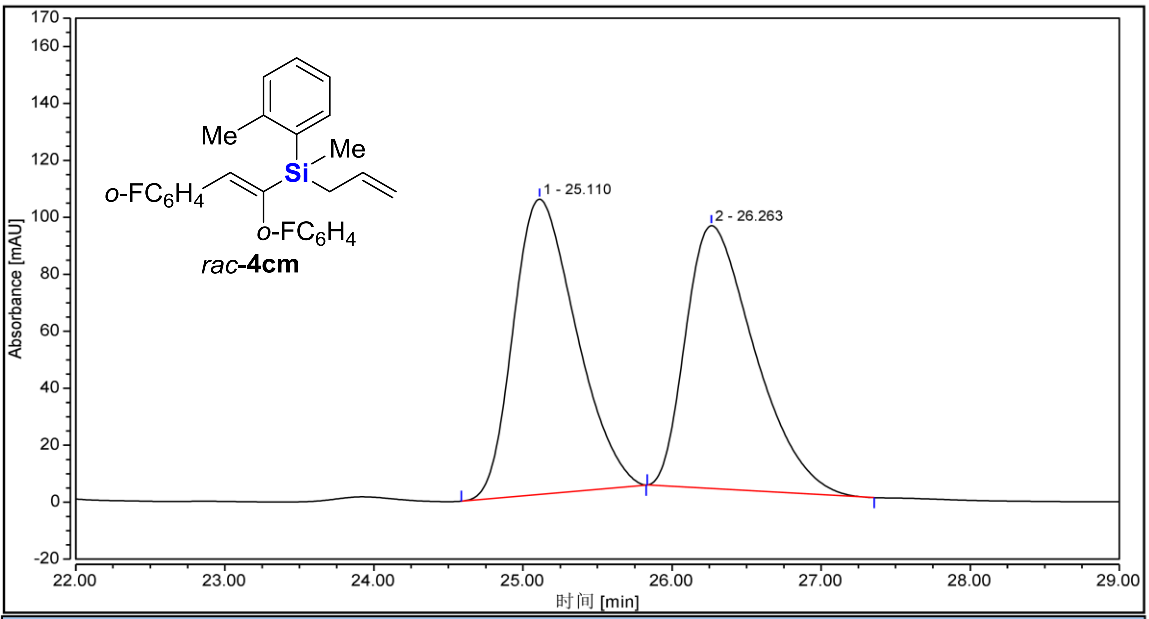

Result					
Peak #	Run time [min][	Area [mAU*min]	Height [mAU]	Area %	Height %
1	25.110	50.624	103.646	50.49	52.90
2	26.263	49.636	92.279	49.51	47.10
**Totals:**		**100.260**	**195.925**	**100.00**	**100.00**

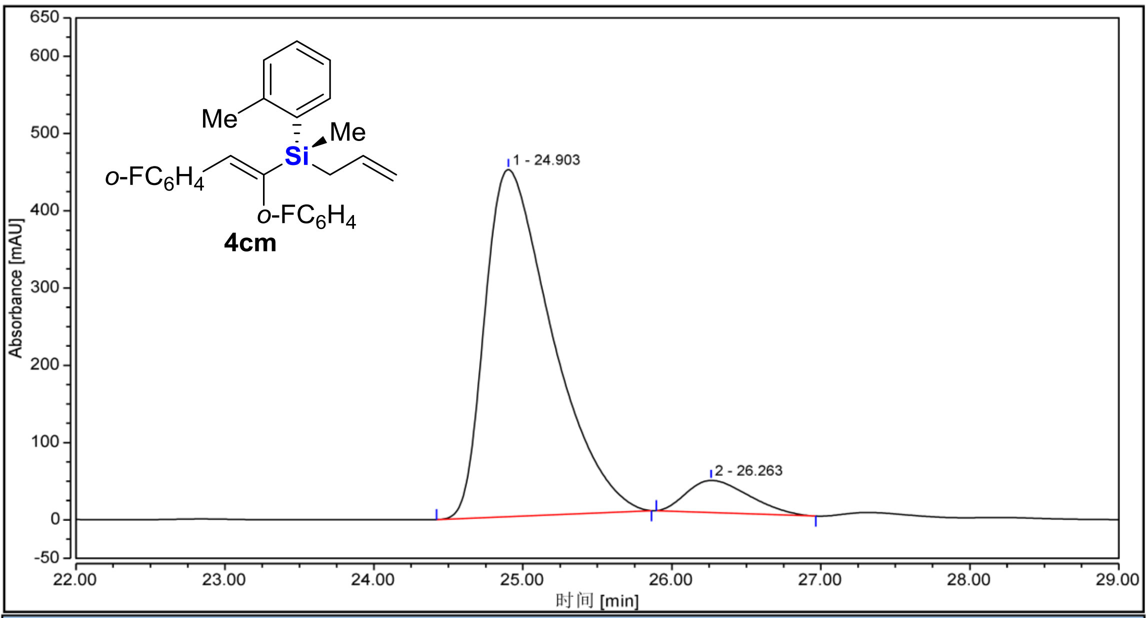

Result					
Peak #	Run time [min][	Area [mAU*min]	Height [mAU]	Area %	Height %
1	24.903	235.049	449.296	92.09	91.54
2	26.263	20.180	41.539	7.91	8.46
**Totals:**		**255.229**	**490.835**	**100.00**	**100.00**

Supplementary Figure 126 HPLC of compounds *rac*-4cm and 4cm

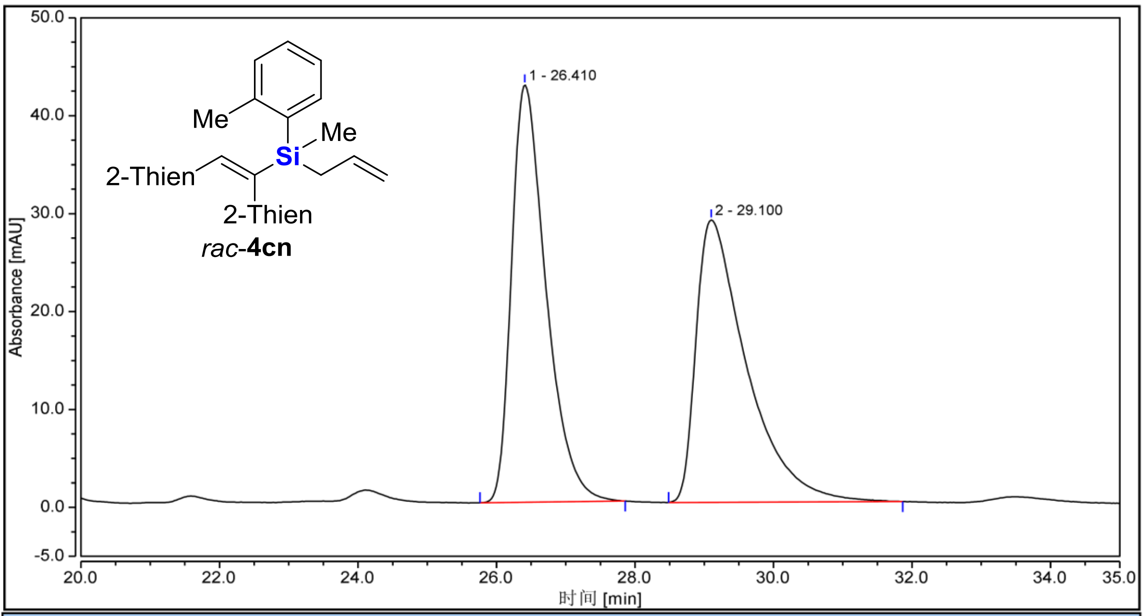

Result

Peak #	Run time [min][	Area [mAU*min]	Height [mAU]	Area %	Height %
1	26.410	24.597	42.602	50.30	59.64
2	29.100	24.305	28.829	49.70	40.36
**Totals:**		**48.902**	**71.431**	**100.00**	**100.00**

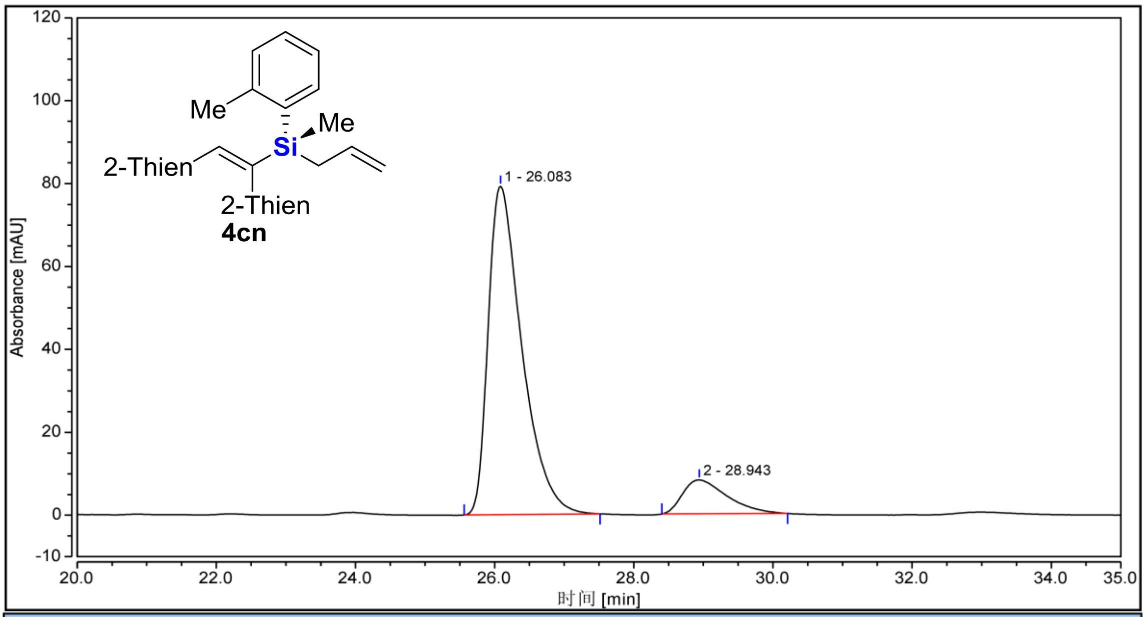

Result

Peak #	Run time [min][	Area [mAU*min]	Height [mAU]	Area %	Height %
1	26.083	44.371	79.239	88.00	90.64
2	28.943	6.053	8.184	12.00	9.36
**Totals:**		**50.424**	**87.423**	**100.00**	**100.00**

Supplementary Figure 127 HPLC of compounds *rac*-4cn and 4cn

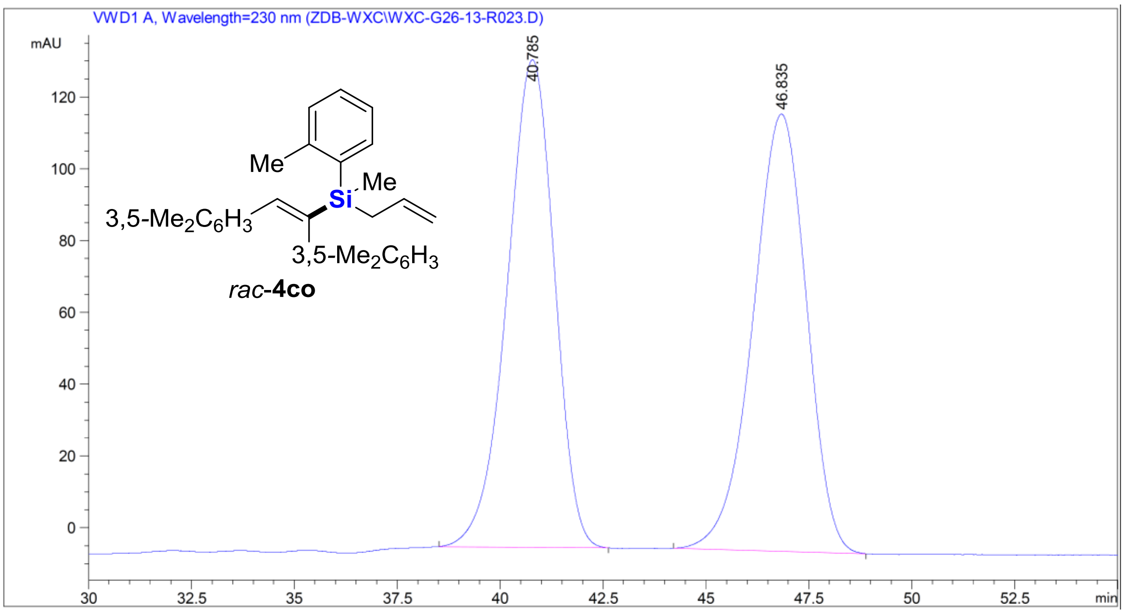

i

Peak #	RetTime [min]	Type	Width [min]	Area mAU *s	Height [mAU]	Area %
1	40.785	MM R	1.3319	1.08576e4	135.86742	49.5876
2	46.835	MM R	1.5094	1.10381e4	121.87929	50.4124

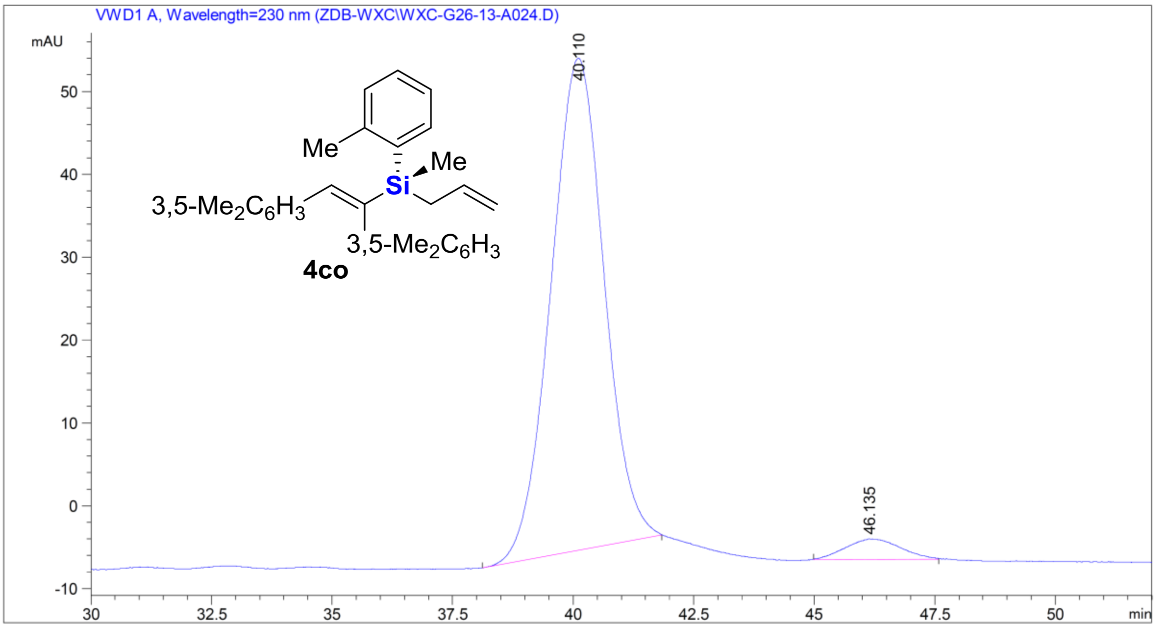

Peak #	RetTime [min]	Type	Width [min]	Area mAU *s	Height [mAU]	Area %
1	40.110	MM R	1.2784	4552.52100	59.35115	95.9010
2	46.135	MM R	1.3112	194.58606	2.47337	4.0990

Supplementary Figure 128 HPLC of compounds *rac*-4co and 4co

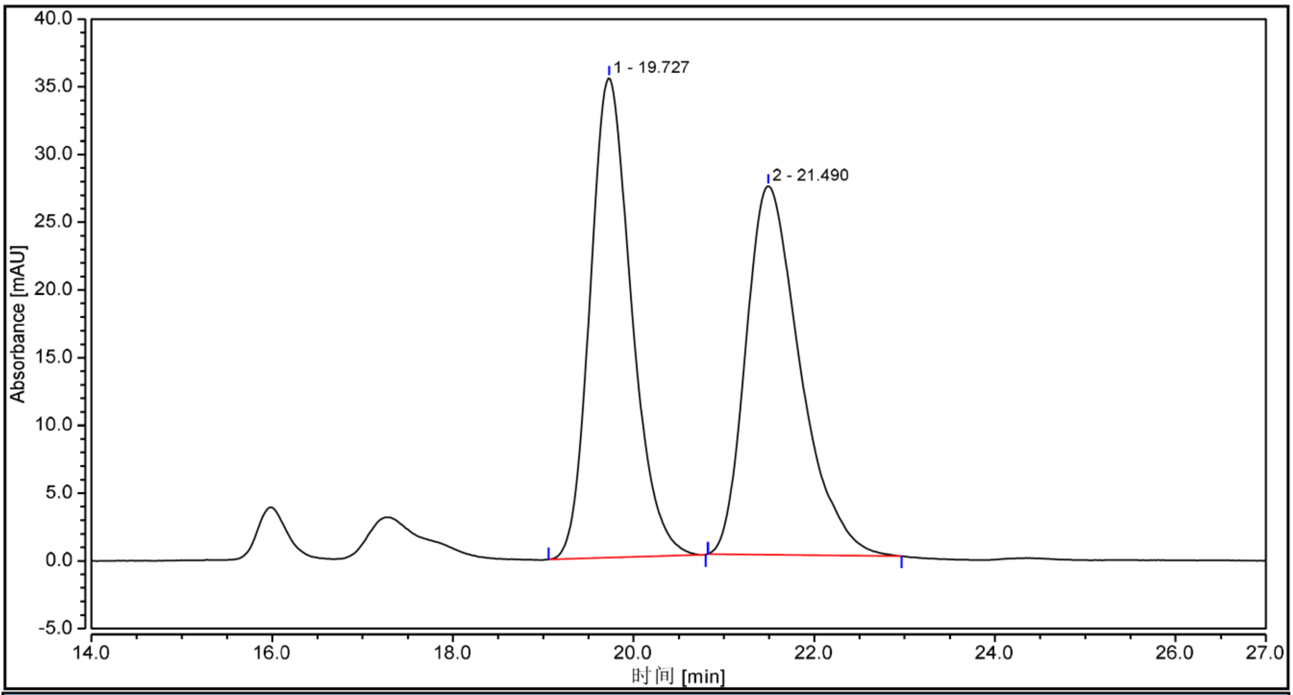

Result

Peak #	Run time [min][	Area [mAU*min]	Height [mAU]	Area %	Height %
1	19.727	18.785	35.416	49.79	56.54
2	21.490	18.940	27.223	50.21	43.46
**Totals:**		**37.726**	**62.639**	**100.00**	**100.00**

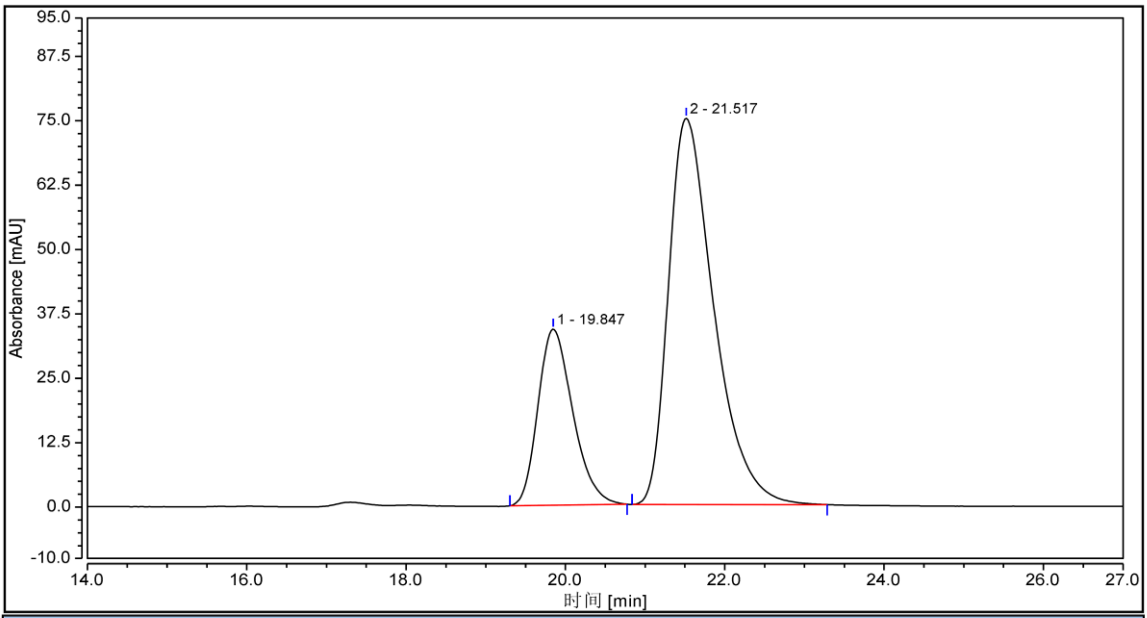

Result

Peak #	Run time [min][	Area [mAU*min]	Height [mAU]	Area %	Height %
1	19.847	17.316	34.175	25.91	31.30
2	21.517	49.521	75.013	74.09	68.70
**Totals:**		**66.837**	**109.188**	**100.00**	**100.00**

Supplementary Figure 129 HPLC of compounds *rac*-4cp and 4cp

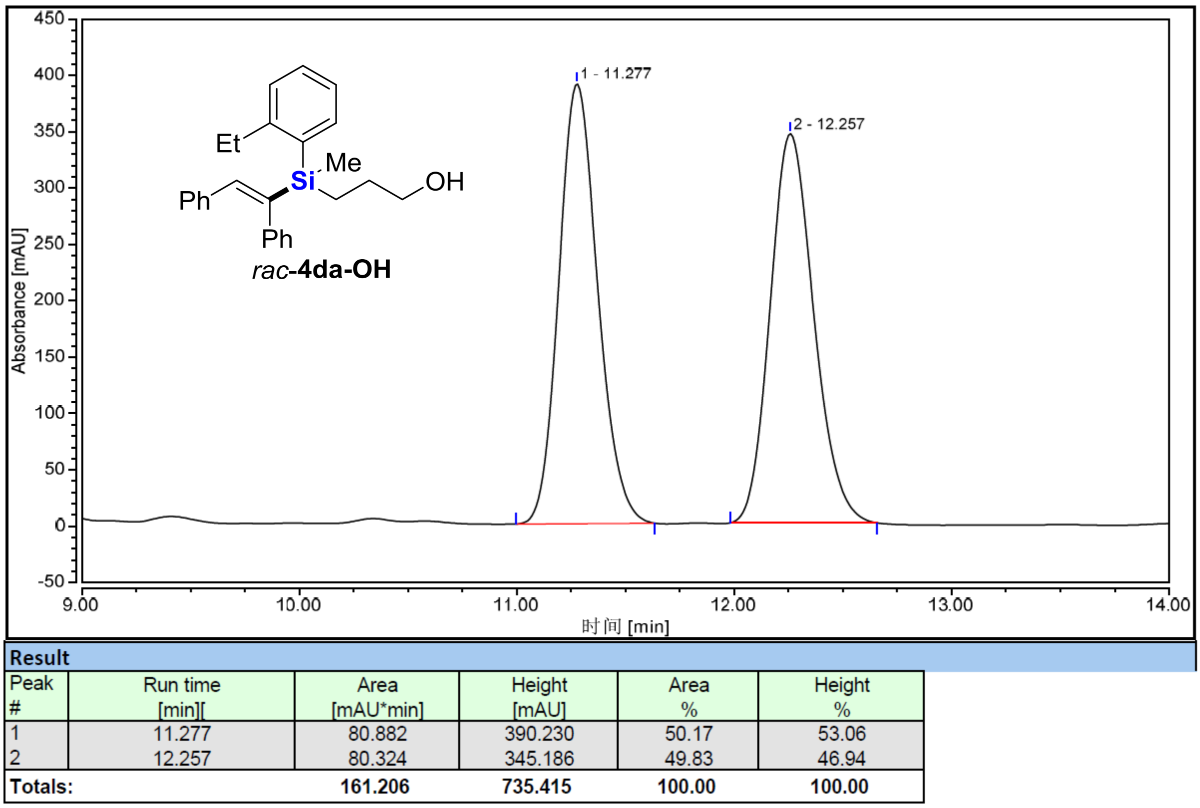

Result

Peak #	Run time [min][	Area [mAU*min]	Height [mAU]	Area %	Height %
1	11.277	80.882	390.230	50.17	53.06
2	12.257	80.324	345.186	49.83	46.94
**Totals:**		**161.206**	**735.415**	**100.00**	**100.00**

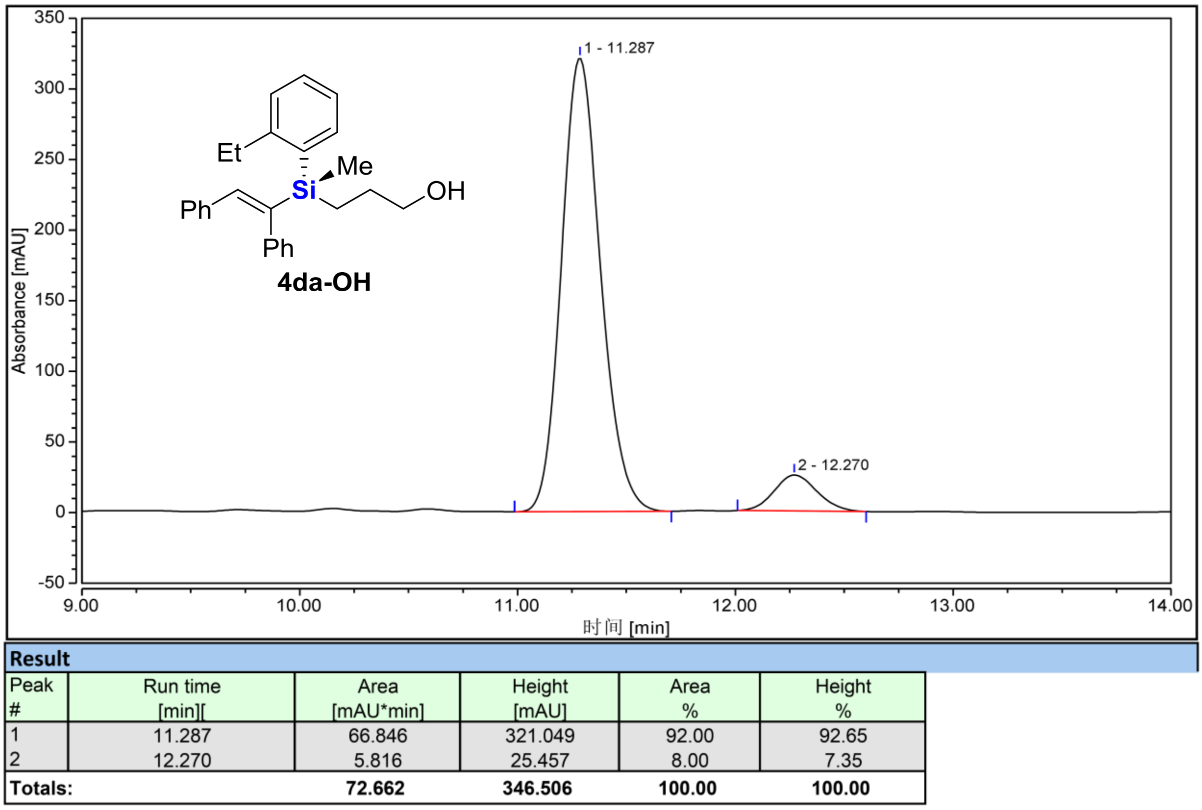

Result

Peak #	Run time [min][	Area [mAU*min]	Height [mAU]	Area %	Height %
1	11.287	66.846	321.049	92.00	92.65
2	12.270	5.816	25.457	8.00	7.35
**Totals:**		**72.662**	**346.506**	**100.00**	**100.00**

Supplementary Figure 130 HPLC of compounds *rac*-4da-OH and 4da-OH

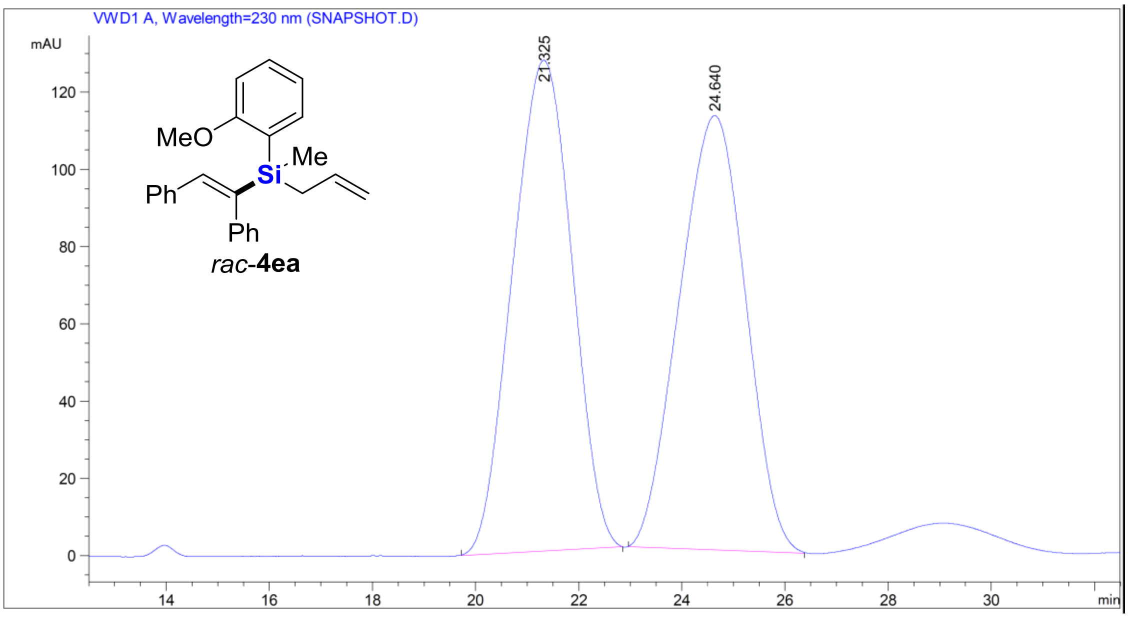

Peak #	RetTime [min]	Type	Width [min]	Area mAU *s	Height [mAU]	Area %
1	21.325	MM R	1.3371	1.01937e4	127.05792	50.4009
2	24.640	MM R	1.4873	1.00315e4	112.40982	49.5991

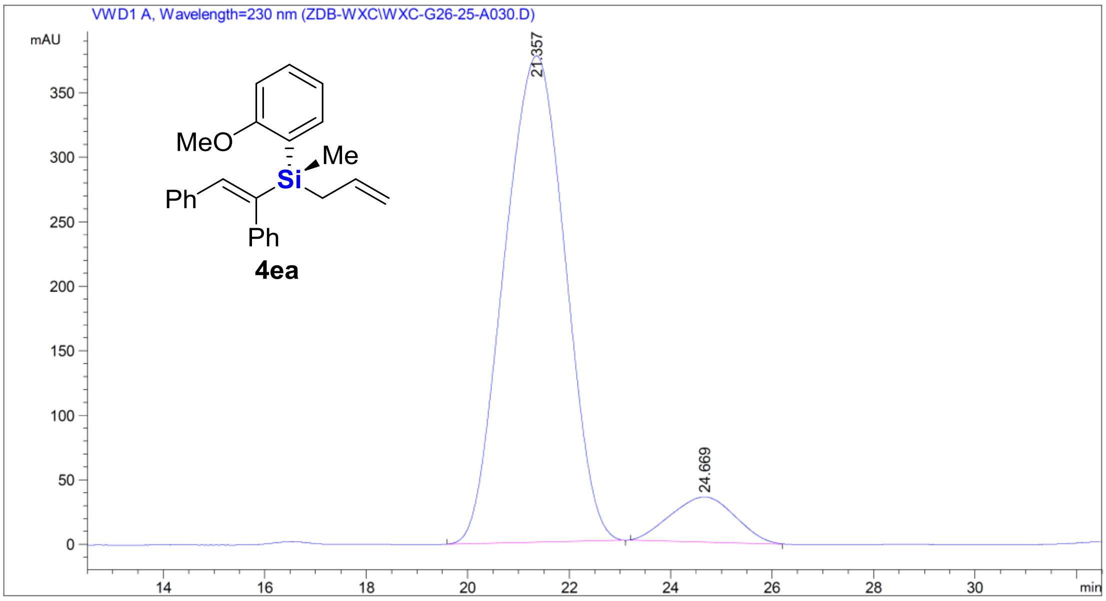

Peak #	RetTime [min]	Type	Width [min]	Area mAU *s	Height [mAU]	Area %
1	21.357	MM R	1.0686	3.14234e4	377.02765	91.0434
2	24.669	MM R	1.4673	3091.34155	35.11405	8.9566

Supplementary Figure 131 HPLC of compounds *rac*-4ea and 4ea

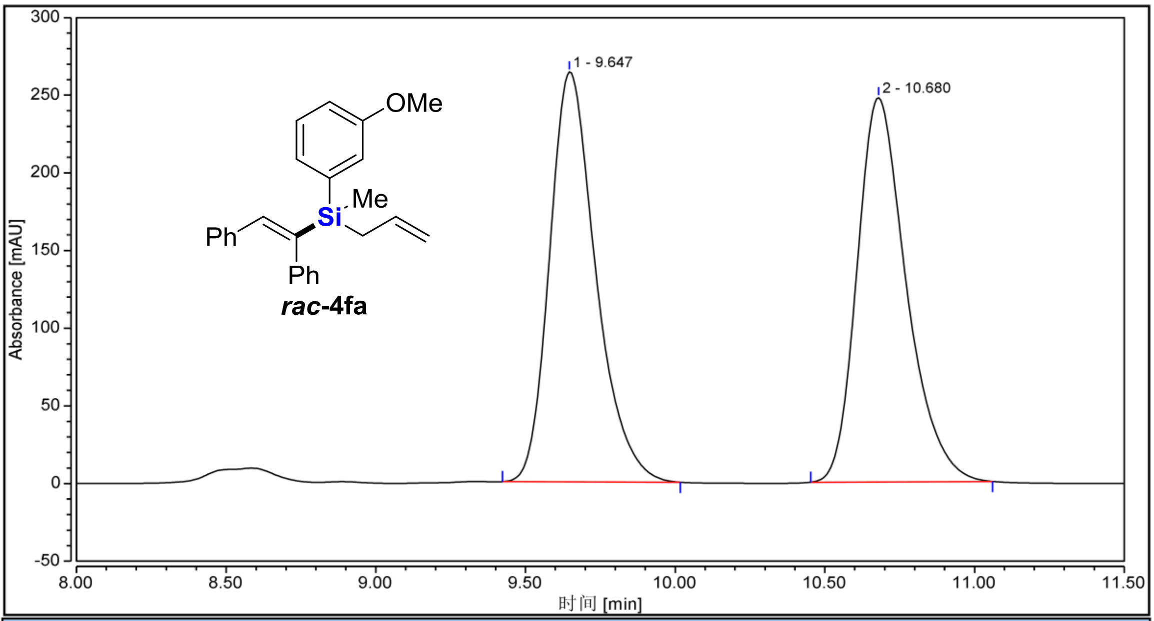

Result

Peak #	Run time [min][	Area [mAU*min]	Height [mAU]	Area %	Height %
1	9.647	47.237	263.931	50.05	51.62
2	10.680	47.143	247.344	49.95	48.38
**Totals:**		**94.379**	**511.275**	**100.00**	**100.00**

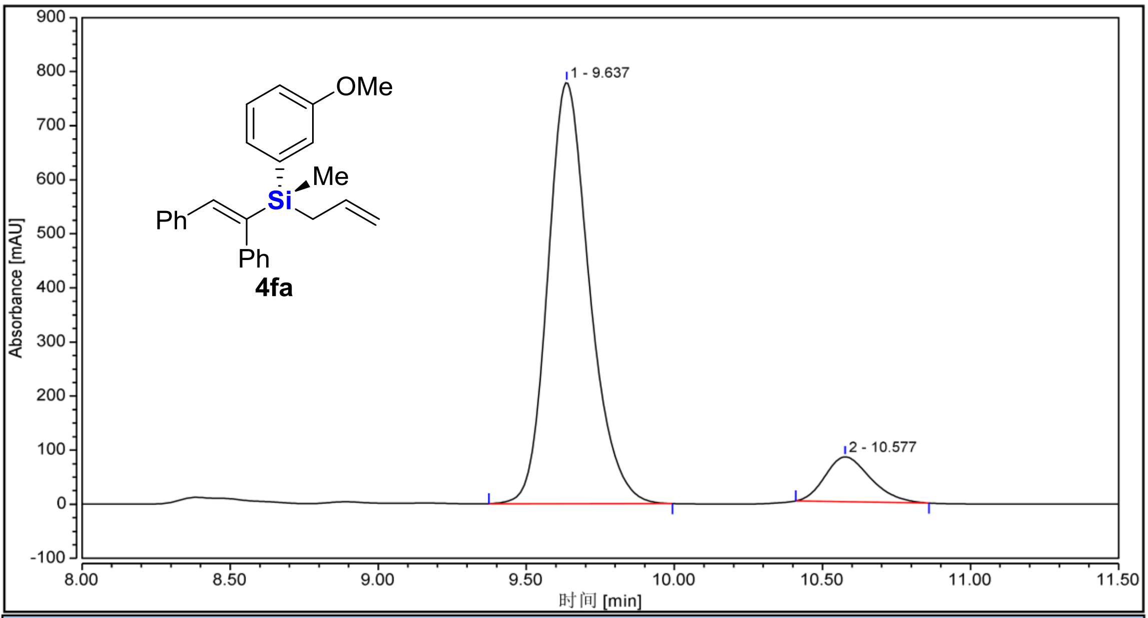

Result

Peak #	Run time [min][	Area [mAU*min]	Height [mAU]	Area %	Height %
1	9.637	127.035	778.846	89.58	90.37
2	10.577	14.771	82.965	10.42	9.63
**Totals:**		**141.806**	**861.810**	**100.00**	**100.00**

Supplementary Figure 132 HPLC of compounds *rac*-4fa and 4fa

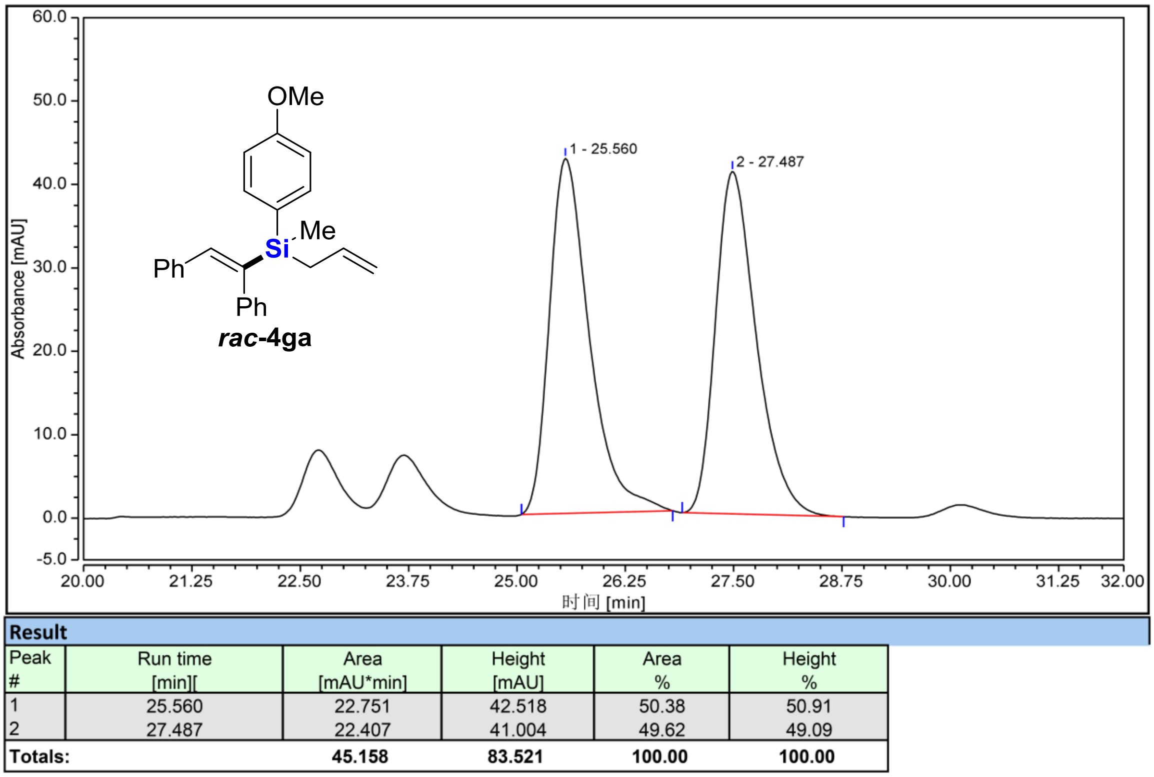

Result

Peak #	Run time [min][	Area [mAU*min]	Height [mAU]	Area %	Height %
1	25.560	22.751	42.518	50.38	50.91
2	27.487	22.407	41.004	49.62	49.09
**Totals:**		**45.158**	**83.521**	**100.00**	**100.00**

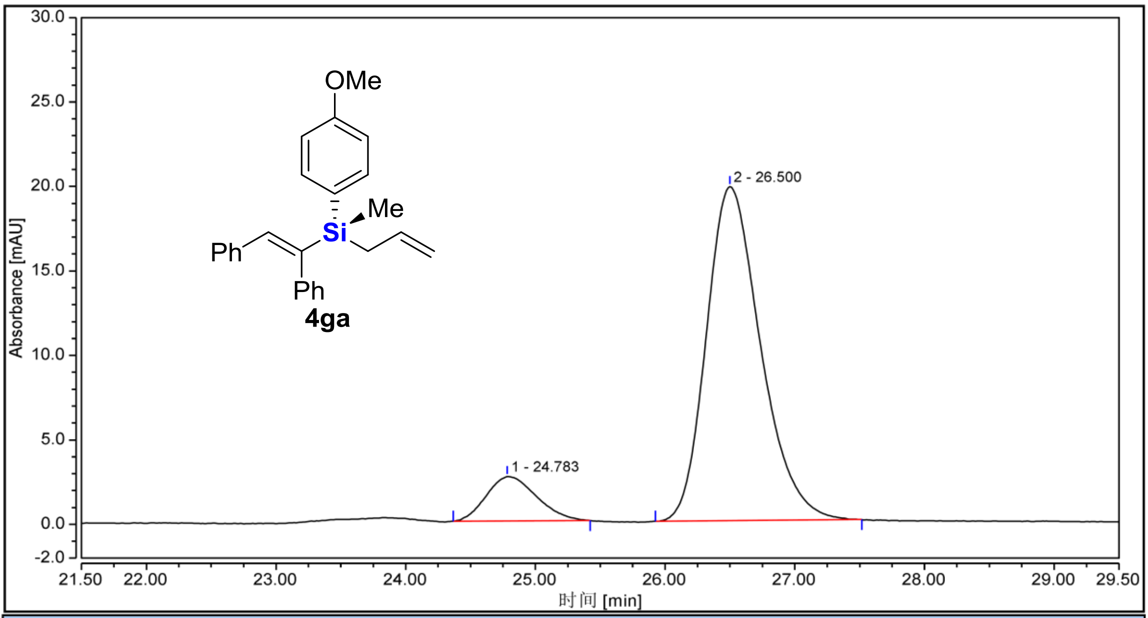

Result

Peak #	Run time [min][	Area [mAU*min]	Height [mAU]	Area %	Height %
1	24.783	1.180	2.635	10.99	11.76
2	26.500	9.564	19.766	89.01	88.24
**Totals:**		**10.744**	**22.400**	**100.00**	**100.00**

Supplementary Figure 133 HPLC of compounds *rac*-4ga and 4ga

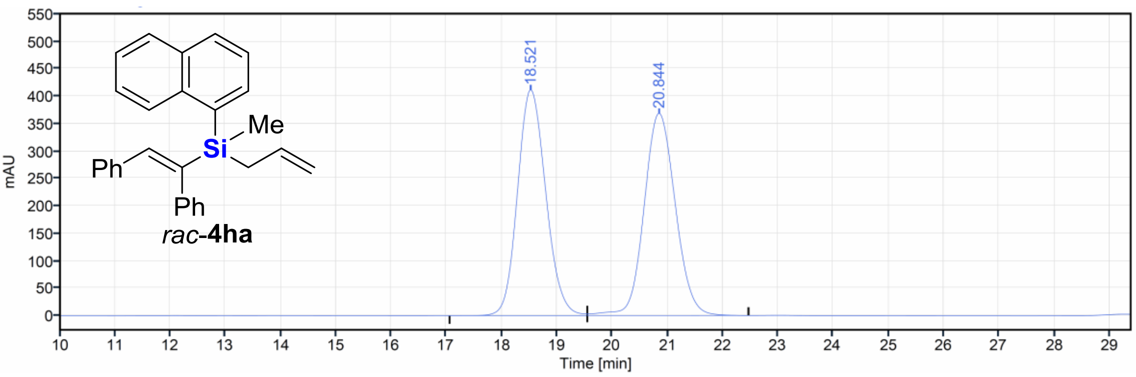

Signal: DAD1B,Sig=210,4 Ref=360,100

RT [min]	Type	Width [min]	Area	Height	Area%
18.521	BV	2.49	14640.89	411.50	50.24
20.844	VB	2.92	14500.64	368.60	49.76

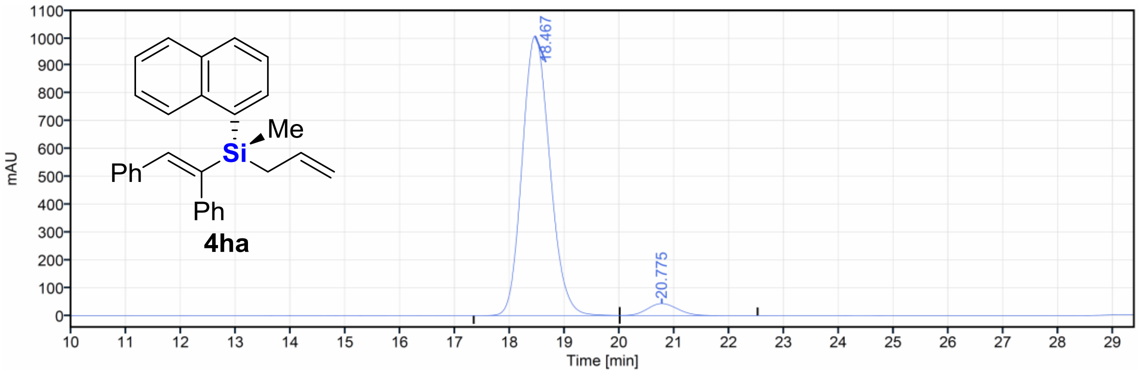

Signal: DAD1B,Sig=210,4 Ref=360,100

RT [min]	Type	Width [min]	Area	Height	Area%
18.467	BV	2.66	35365.39	1008.40	95.44
20.775	VB	2.51	1691.59	43.54	4.56

Supplementary Figure 134 HPLC of compounds *rac*-4ha and 4ha

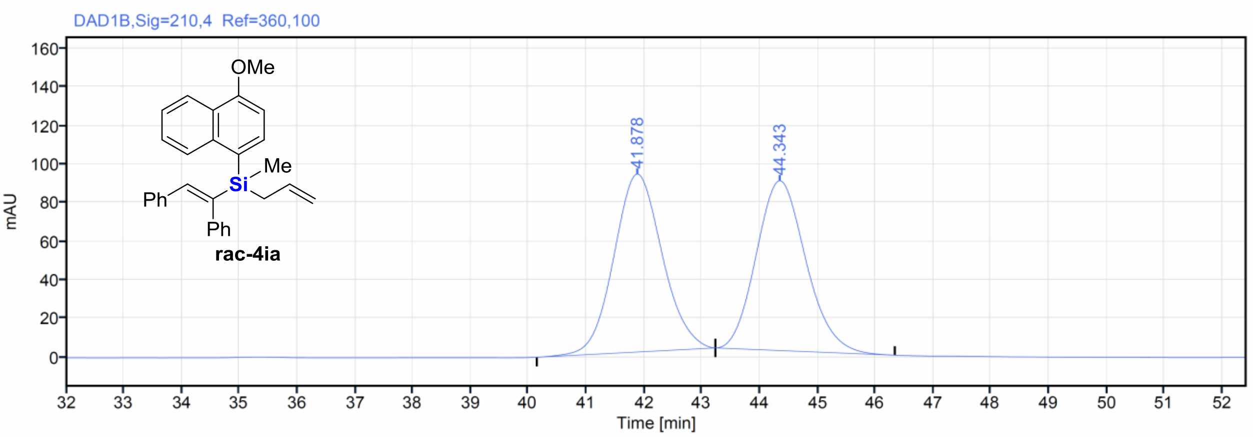

Signal: DAD1B,Sig=210,4 Ref=360,100

RT [min]	Type	Width [min]	Area	Height	Area%
41.878	BB	3.09	5349.57	92.29	50.46
44.343	BB	3.10	5252.85	88.06	49.54

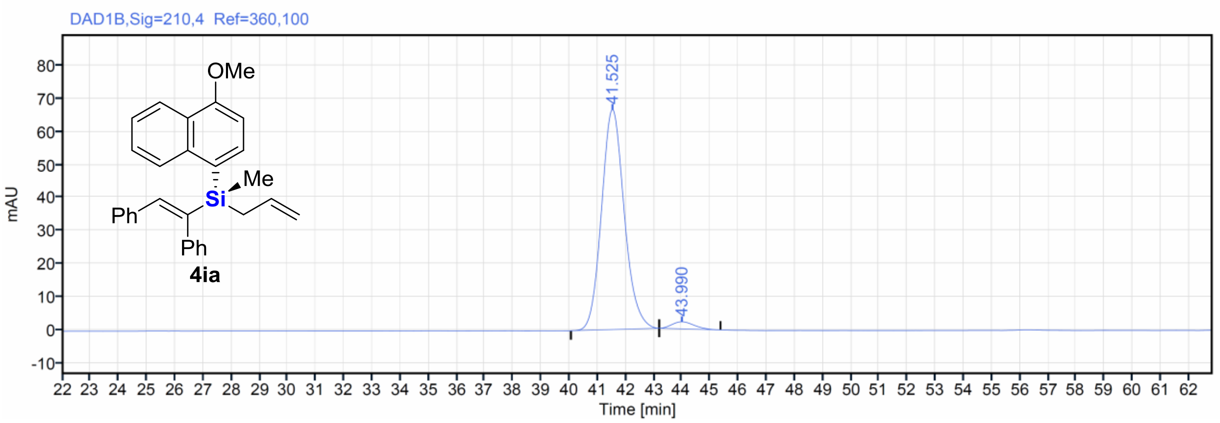

Signal: DAD1B,Sig=210,4 Ref=360,100

RT [min]	Type	Width [min]	Area	Height	Area%
41.525	BB	3.14	3712.50	66.17	96.97
43.990	BBA	2.17	115.92	2.12	3.03

Supplementary Figure 135 HPLC of compounds *rac*-4ia and 4ia

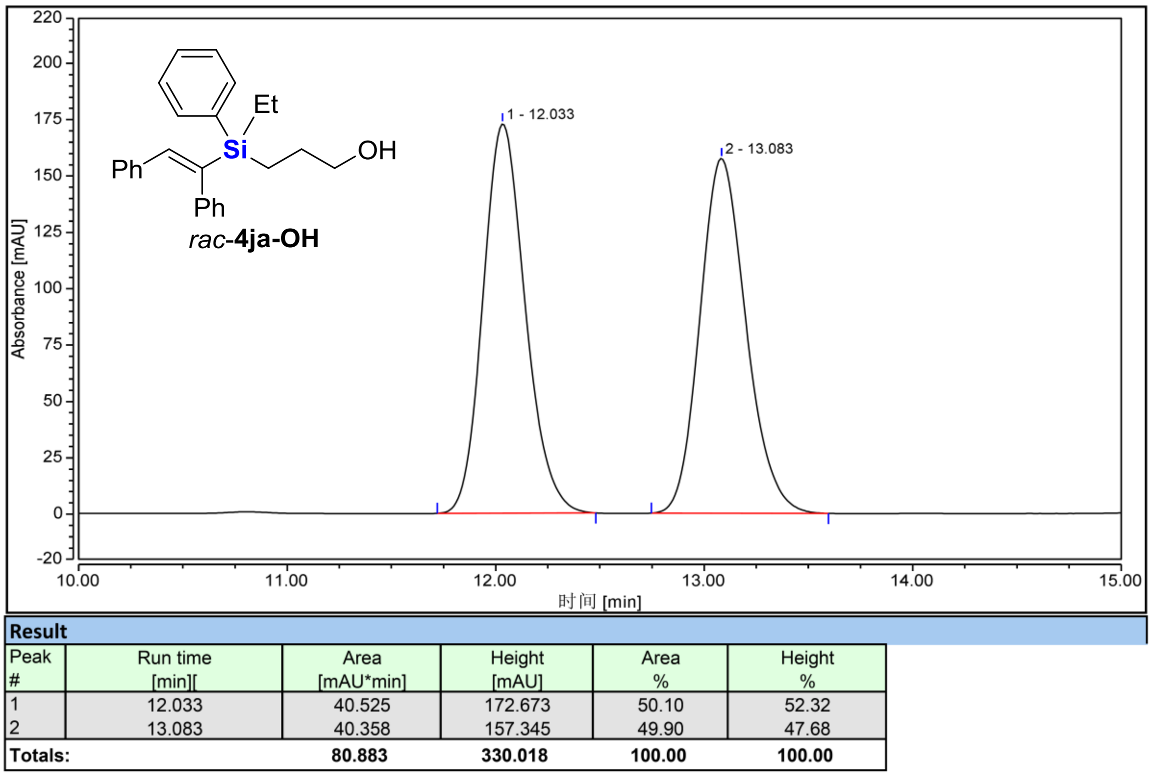

Result

Peak #	Run time [min][	Area [mAU*min]	Height [mAU]	Area %	Height %
1	12.033	40.525	172.673	50.10	52.32
2	13.083	40.358	157.345	49.90	47.68
**Totals:**		**80.883**	**330.018**	**100.00**	**100.00**

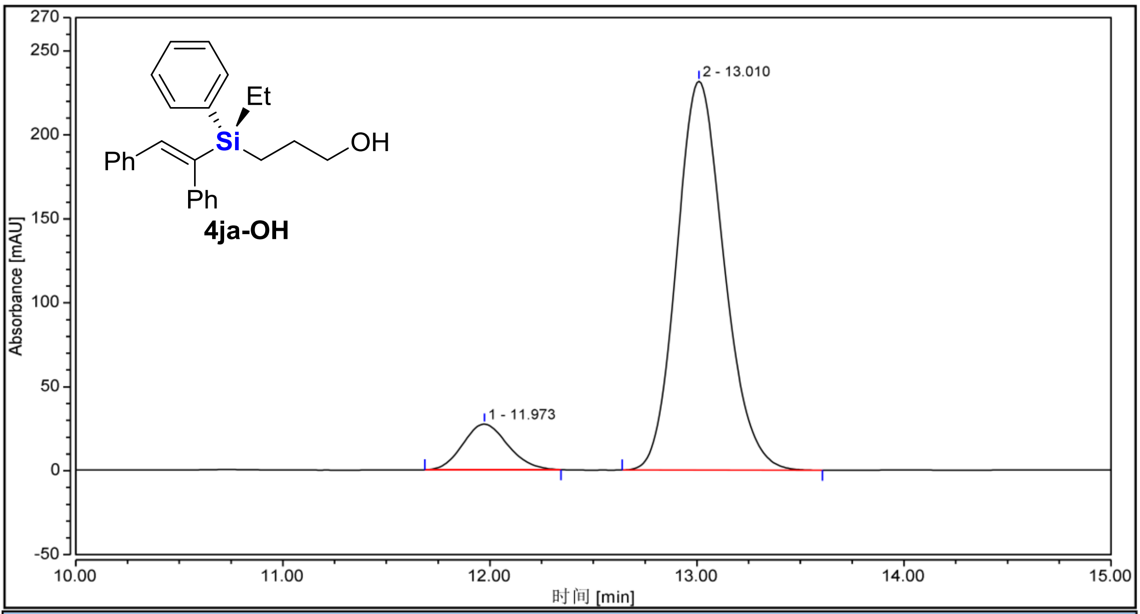

Result

Peak #	Run time [min][	Area [mAU*min]	Height [mAU]	Area %	Height %
1	11.973	6.836	27.179	10.00	10.50
2	13.010	61.515	231.617	90.00	89.50
**Totals:**		**68.351**	**258.796**	**100.00**	**100.00**

Supplementary Figure 136 HPLC of compounds *rac*-4ja-OH and 4ja-OH

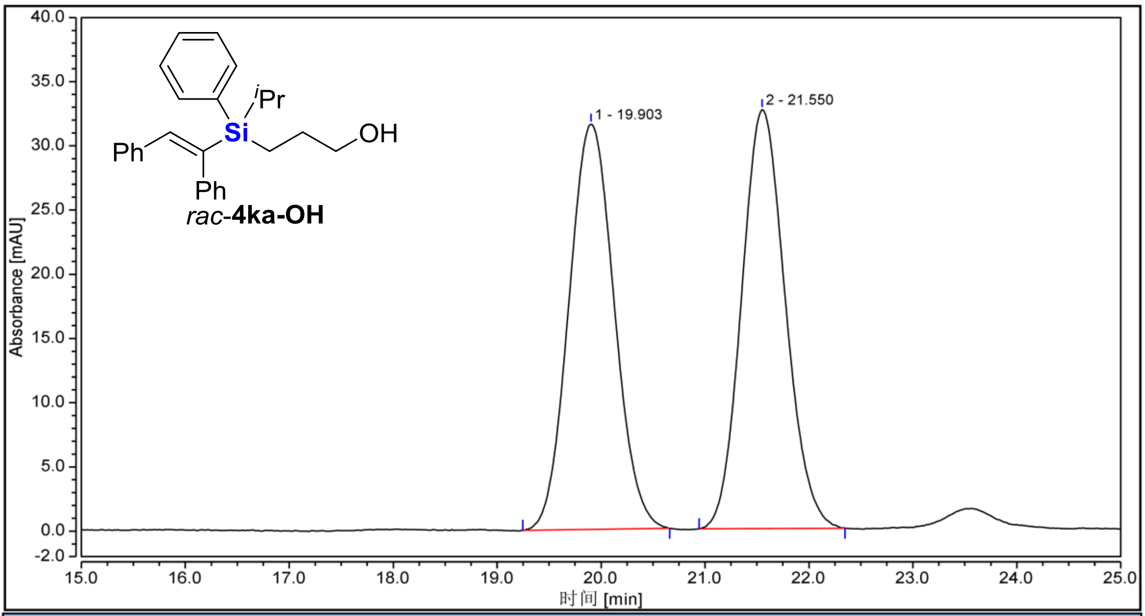

Result					
Peak #	Run time [min][	Area [mAU*min]	Height [mAU]	Area %	Height %
1	19.903	15.925	31.562	50.04	49.16
2	21.550	15.899	32.639	49.96	50.84
Totals:		**31.824**	**64.201**	**100.00**	**100.00**

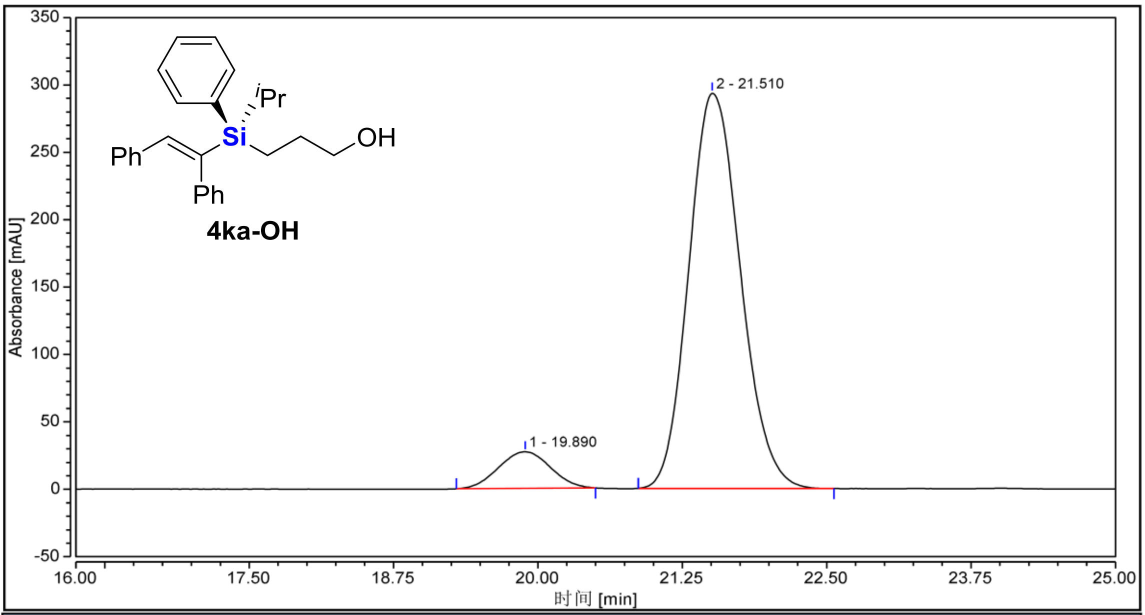

Result					
Peak #	Run time [min][	Area [mAU*min]	Height [mAU]	Area %	Height %
1	19.890	14.086	27.159	8.49	8.48
2	21.510	151.780	293.176	91.51	91.52
Totals:		**165.866**	**320.335**	**100.00**	**100.00**

Supplementary Figure 137 HPLC of compounds *rac*-4ka-OH and 4ka-OH

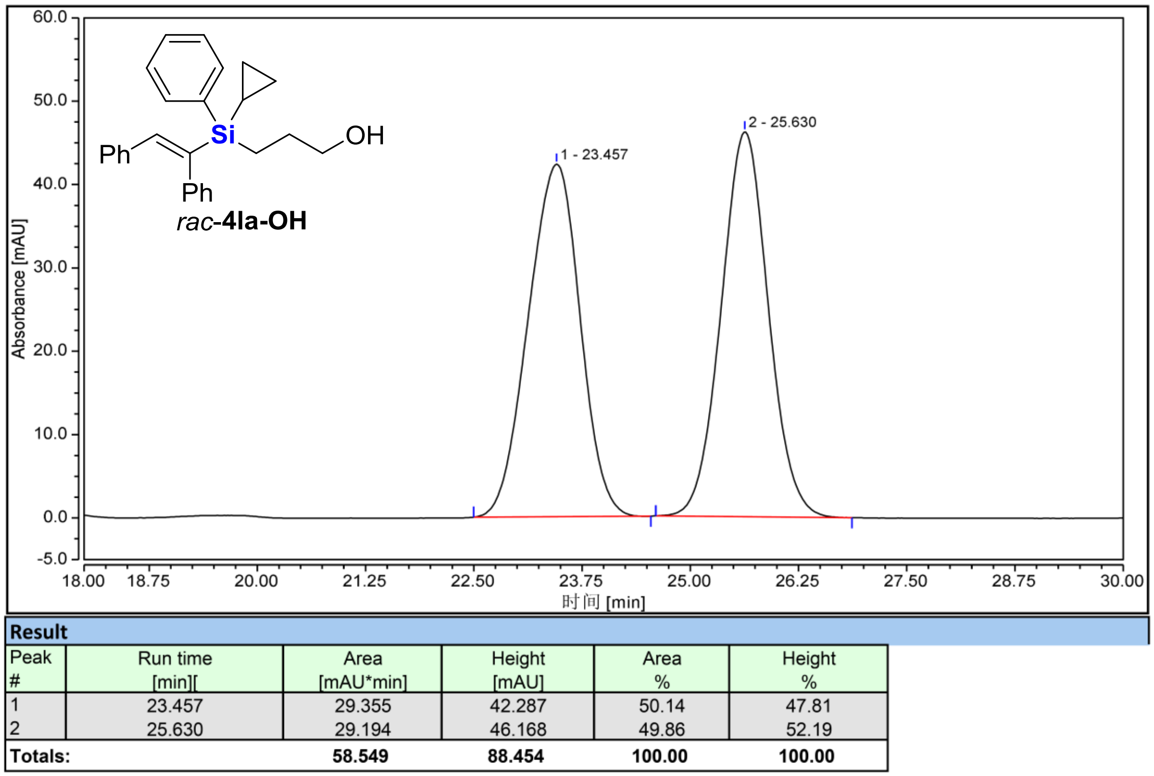

Peak #	Run time [min][	Area [mAU*min]	Height [mAU]	Area %	Height %
1	23.457	29.355	42.287	50.14	47.81
2	25.630	29.194	46.168	49.86	52.19
**Totals:**		**58.549**	**88.454**	**100.00**	**100.00**

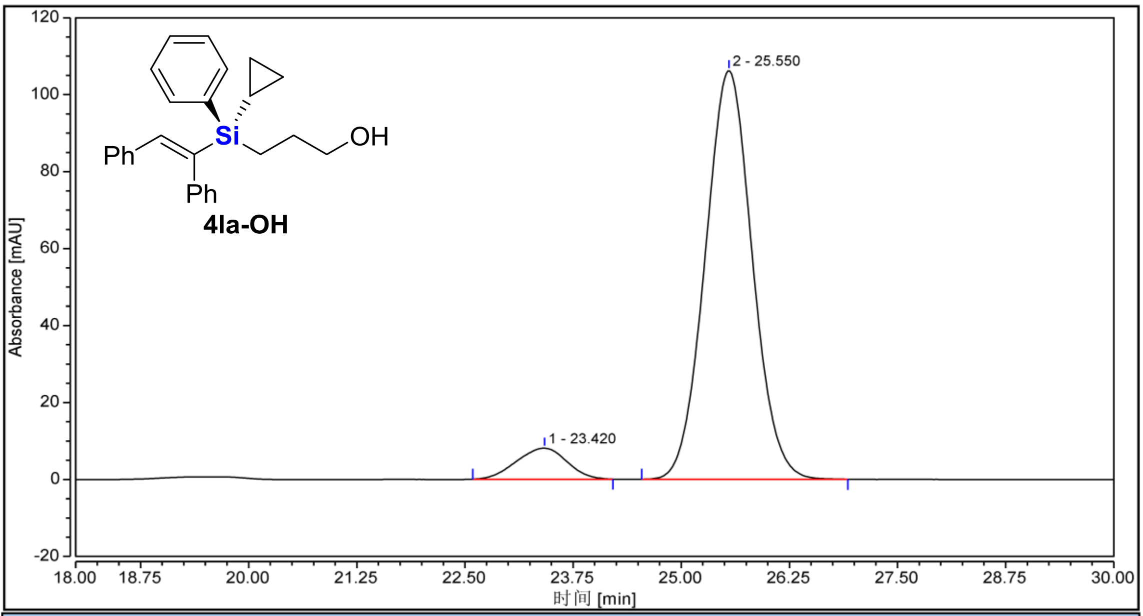

Peak #	Run time [min][	Area [mAU*min]	Height [mAU]	Area %	Height %
1	23.420	5.448	8.060	7.52	7.06
2	25.550	66.988	106.162	92.48	92.94
**Totals:**		**72.436**	**114.222**	**100.00**	**100.00**

Supplementary Figure 138 HPLC of compounds *rac*-4la-OH and 4la-OH

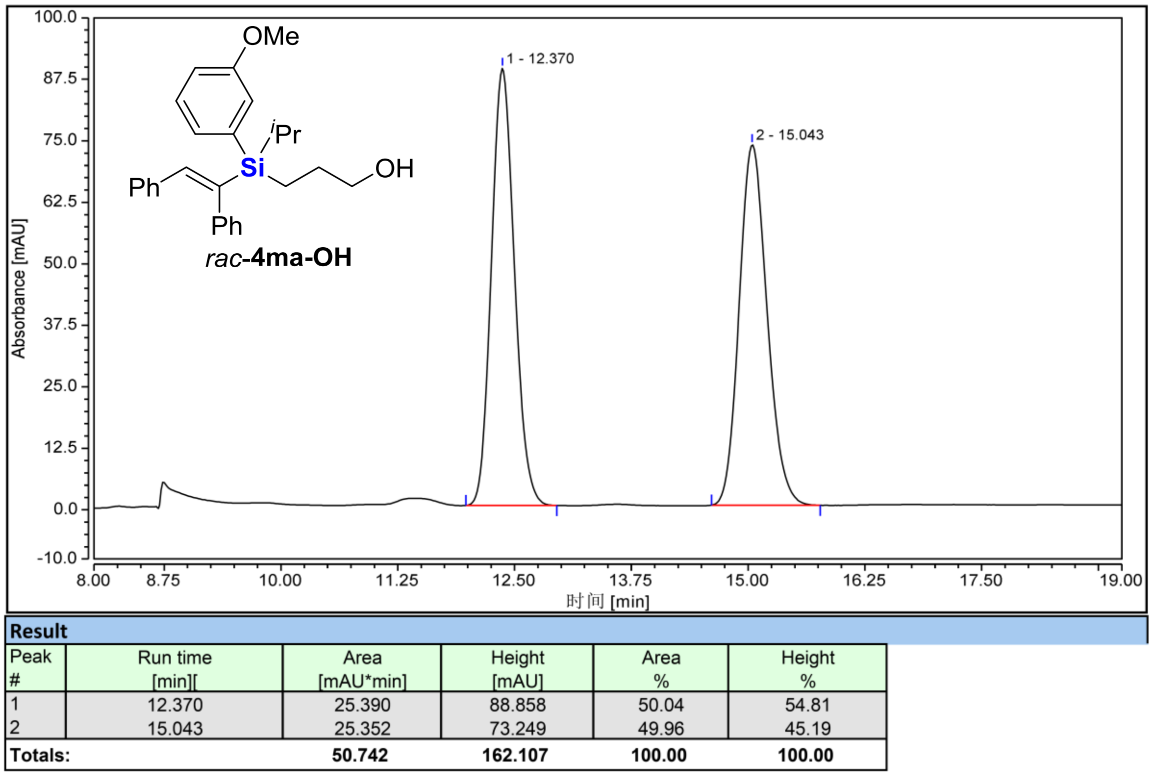

Result

Peak #	Run time [min][	Area [mAU*min]	Height [mAU]	Area %	Height %
1	12.370	25.390	88.858	50.04	54.81
2	15.043	25.352	73.249	49.96	45.19
**Totals:**		**50.742**	**162.107**	**100.00**	**100.00**

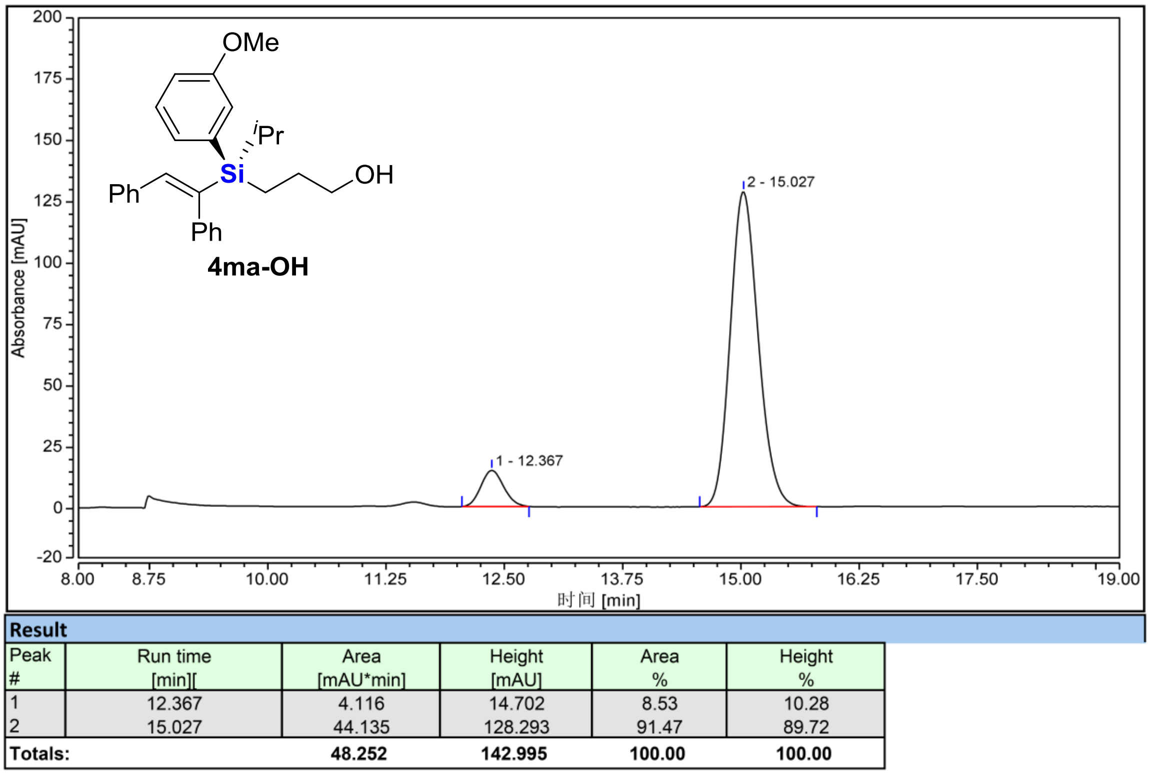

Result

Peak #	Run time [min][	Area [mAU*min]	Height [mAU]	Area %	Height %
1	12.367	4.116	14.702	8.53	10.28
2	15.027	44.135	128.293	91.47	89.72
**Totals:**		**48.252**	**142.995**	**100.00**	**100.00**

Supplementary Figure 139 HPLC of compounds *rac*-4ma-OH and 4ma-OH

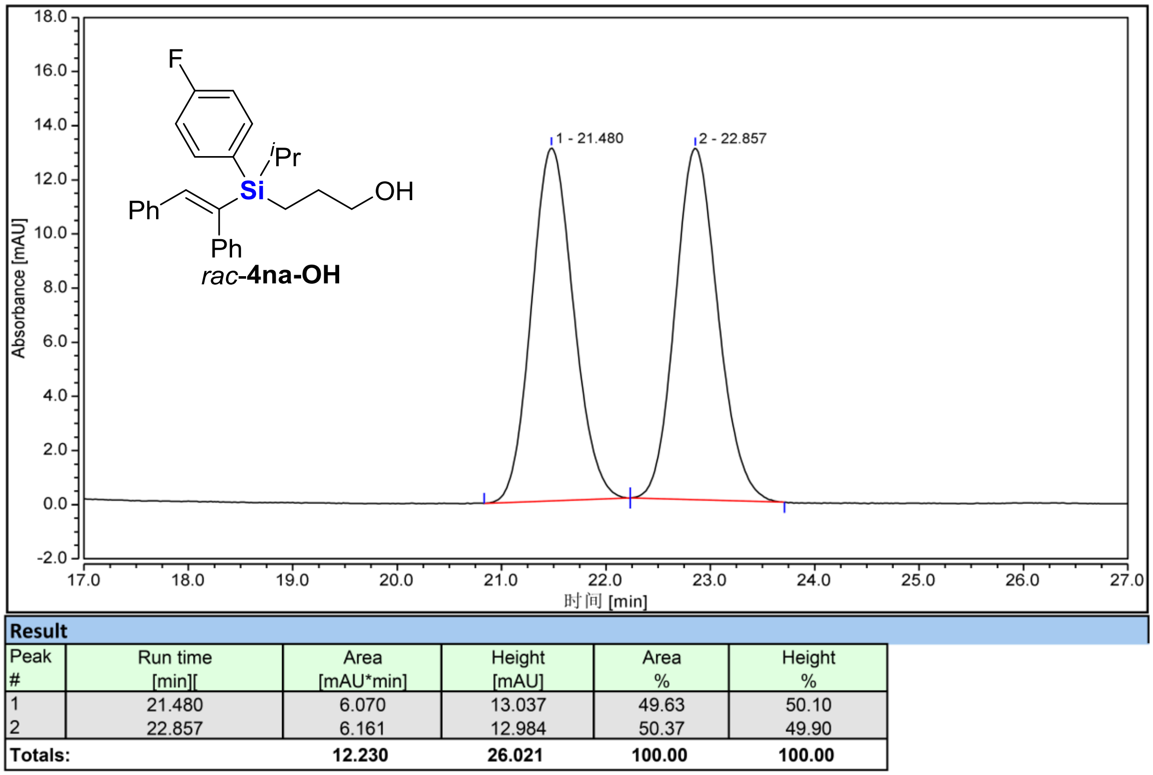

Result

Peak #	Run time [min][	Area [mAU*min]	Height [mAU]	Area %	Height %
1	21.480	6.070	13.037	49.63	50.10
2	22.857	6.161	12.984	50.37	49.90
**Totals:**		**12.230**	**26.021**	**100.00**	**100.00**

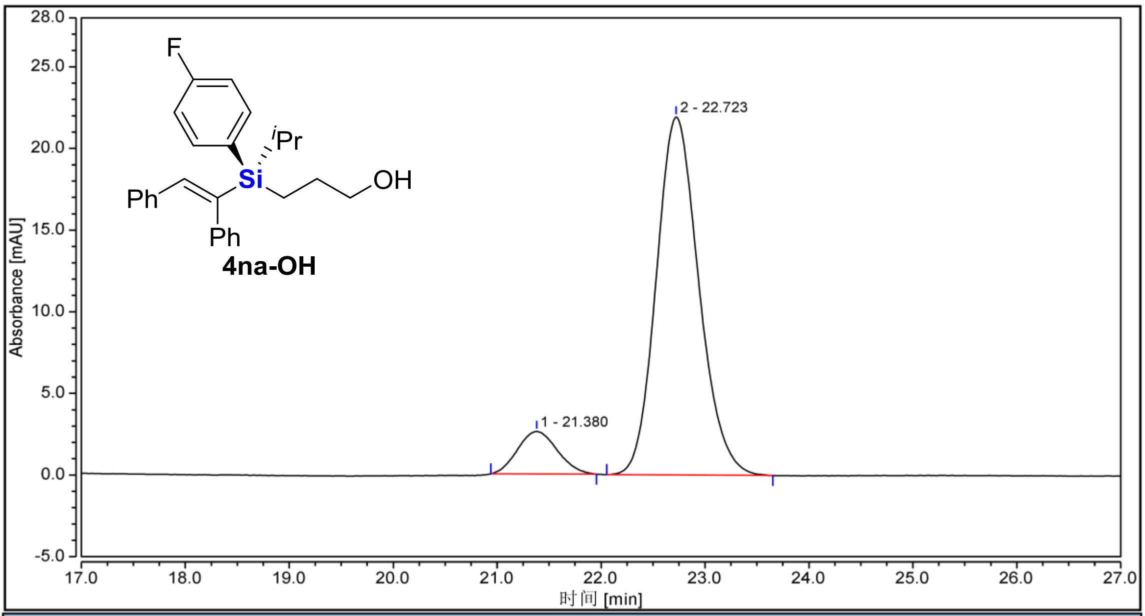

Result

Peak #	Run time [min][	Area [mAU*min]	Height [mAU]	Area %	Height %
1	21.380	1.155	2.606	10.00	10.63
2	22.723	10.387	21.912	90.00	89.37
**Totals:**		**11.542**	**24.518**	**100.00**	**100.00**

Supplementary Figure 140 HPLC of compounds *rac*-4na-OH and 4na-OH

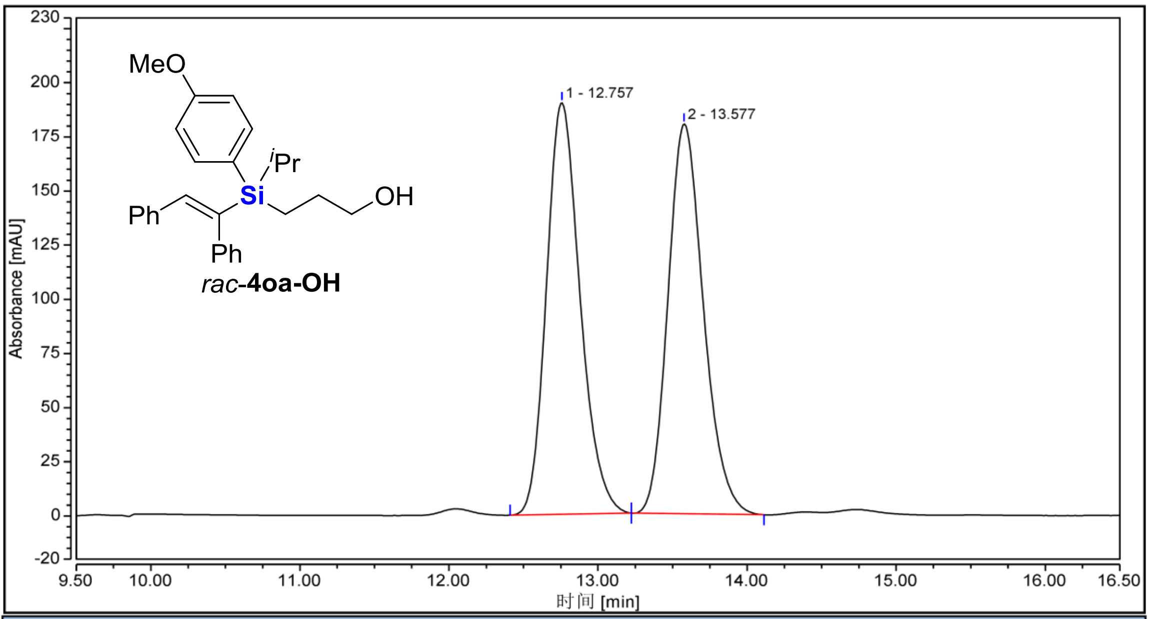

Result

Peak #	Run time [min][	Area [mAU*min]	Height [mAU]	Area %	Height %
1	12.757	49.736	190.116	50.03	51.37
2	13.577	49.681	179.985	49.97	48.63
**Totals:**		**99.416**	**370.101**	**100.00**	**100.00**

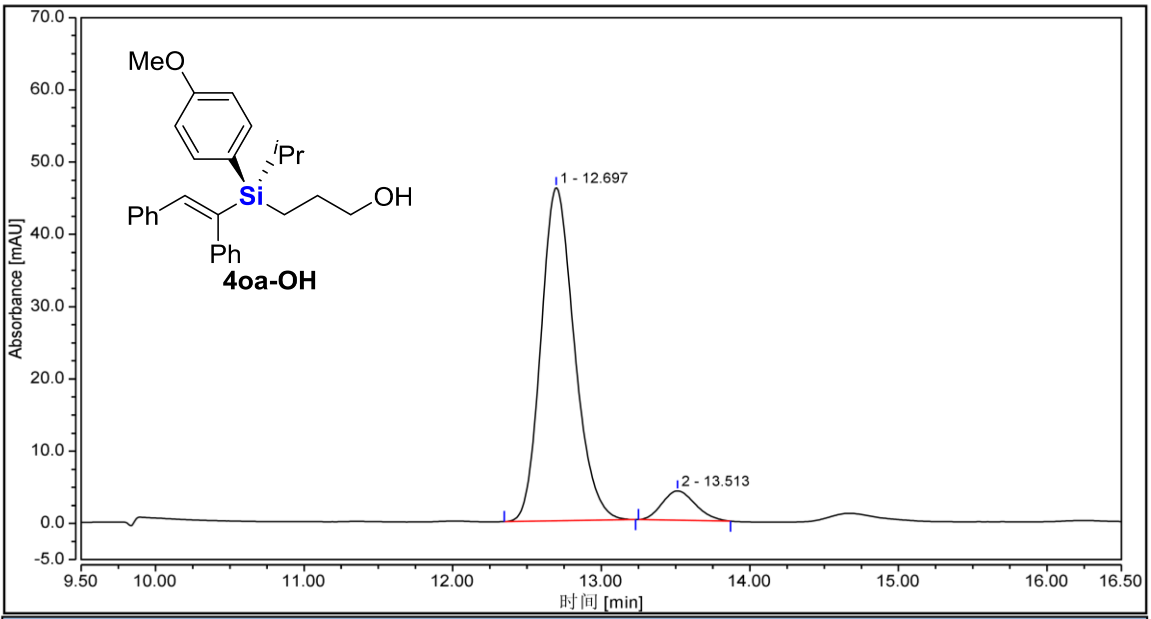

Result

Peak #	Run time [min][	Area [mAU*min]	Height [mAU]	Area %	Height %
1	12.697	11.954	46.086	91.91	91.92
2	13.513	1.052	4.049	8.09	8.08
**Totals:**		**13.006**	**50.135**	**100.00**	**100.00**

Supplementary Figure 141 HPLC of compounds *rac*-4oa-OH and 4oa-OH

Absolute energies and coordinates of optimized structures

Reductive Elimination vs Ligand-to-Ligand H Transfer (L = IPr, 393.15 K, Toluene):

Energetic information:

Chemical Structure	Electronic Energy (a.u.)	Enthalpy (a.u.)	Free Energy (a.u.)	Imaginary Frequency (cm^{-1})
**IPr-B**	-3693.383151	-3692.386712	-3692.589596	
**IPr-TS$_{RE}$**	-3693.372776	-3692.377258	-3692.577064	-268.90
**IPr-TS$_{LLHT}$**	-3693.362018	-3692.368029	-3692.565743	-937.45

Cartesian coordinates:

IPr-B

C	0.63035000	-0.83822500	2.13279200
C	0.08981700	-2.15451100	2.63580700
Si	-1.21029100	-3.13353800	0.19888400
C	0.11353300	-3.24863200	1.56900900
H	-0.01035900	-4.24207000	2.04655800
H	1.12357000	-3.27161700	1.10663000
C	-0.38907700	-3.00159800	-1.49816100
H	-1.13307800	-3.00951700	-2.31546000
H	0.30502200	-3.84233000	-1.67965000
H	0.18734600	-2.05713000	-1.57262700
C	-2.19314900	-4.74460700	0.24127600
H	-1.51546500	-5.61743700	0.18562000
H	-2.91731000	-4.83811400	-0.58745900
H	-2.76857800	-4.83681800	1.18134200
Ni	0.02790900	-0.10062800	0.46539500
C	-2.34629500	-1.66314800	0.52860300
C	-1.74555100	-0.48845500	0.88235800
C	-2.46488600	0.75322700	1.22070400

C	-3.38257000	1.33605600	0.32569400
C	-2.25639500	1.39831000	2.45505300
C	-4.07230600	2.49925800	0.65810100
H	-3.56314900	0.84909900	-0.64142600
C	-2.95612000	2.55319600	2.79229700
H	-1.54198600	0.96311900	3.16578300
C	-3.86808400	3.11371800	1.89398700
H	-4.78410100	2.92940200	-0.05798900
H	-2.78757000	3.02433400	3.76880700
H	-4.41870500	4.02417000	2.15902700
C	-3.81711700	-1.82719500	0.44023300
C	-4.40518800	-2.38641500	-0.70863700
C	-4.67336300	-1.43846700	1.48621500
C	-5.78663800	-2.53435100	-0.81845600
H	-3.75809900	-2.69437800	-1.54206000
C	-6.05333500	-1.59372800	1.38329800
H	-4.23852300	-1.00733800	2.39634500
C	-6.61930300	-2.13841400	0.22885700
H	-6.21751100	-2.96409500	-1.73112500
H	-6.69703800	-1.28530300	2.21615000
H	-7.70611400	-2.25784700	0.14779500
H	0.70772500	-2.48178600	3.50270600
H	-0.93608500	-2.03012400	3.03445000
H	1.71421100	-0.94969900	1.92989900
H	0.50342900	-0.02262500	2.87635800
N	1.34969300	1.52635700	-1.47932800
N	2.89619000	0.26366600	-0.69428500
C	1.56445200	0.55362400	-0.53870200
C	3.47285400	1.03058700	-1.69585000
H	4.53216300	0.95757500	-1.93740900

C	2.49341900	1.82718400	-2.19510400
H	2.50522600	2.58779100	-2.97482100
C	0.13733600	2.28972700	-1.54125500
C	0.02789400	3.41242600	-0.69402200
C	-1.13009200	4.18617400	-0.79829200
H	-1.26259800	5.05237400	-0.13929500
C	-2.12277100	3.86982800	-1.72189400
H	-3.02057000	4.49492900	-1.79114700
C	-1.99339300	2.75097000	-2.53554400
H	-2.79274600	2.50131000	-3.24434200
C	-0.86457700	1.92636500	-2.45580800
C	3.65144000	-0.64715200	0.11847400
C	3.99877700	-1.90292100	-0.40949000
C	4.79075800	-2.74237400	0.38447100
H	5.08225600	-3.72748300	0.00318200
C	5.20095200	-2.35176700	1.65315900
H	5.81752500	-3.02494800	2.25986500
C	4.81326000	-1.11633800	2.16553300
H	5.11973100	-0.83672000	3.17883100
C	4.02898200	-0.23621200	1.41476800
C	3.58026400	1.09797300	1.98212800
C	3.51197400	1.11133800	3.50318900
C	4.45168600	2.24762400	1.47579600
H	2.54911000	1.26947500	1.61530700
H	2.93833300	0.25290600	3.89519400
H	3.01412200	2.03233400	3.85451400
H	4.51464900	1.09419500	3.96892200
H	4.46509000	2.31343400	0.37348900
H	5.49785400	2.13175500	1.81639300
H	4.08255500	3.21665000	1.86020200

C	1.09122500	3.74853100	0.33308000
C	1.46805800	5.22572600	0.33343600
C	0.64389300	3.28408500	1.71782800
H	2.00688600	3.18181600	0.07845900
H	1.77609300	5.57304900	-0.66900200
H	2.30921800	5.40915700	1.02579500
H	0.63322500	5.86835600	0.66668200
H	0.40214300	2.20239600	1.70933400
H	-0.26617400	3.82108000	2.04418100
H	1.43507900	3.46136700	2.47051100
C	3.54404900	-2.35659600	-1.78322600
C	4.61501200	-2.09409000	-2.84224400
C	3.14780100	-3.82989200	-1.80424800
H	2.64631900	-1.76491900	-2.04965100
H	4.88393700	-1.02716100	-2.91921300
H	4.27022500	-2.42123600	-3.83970200
H	5.54118400	-2.65209900	-2.60995300
H	2.47538900	-4.08905400	-0.96881300
H	4.02893200	-4.49432900	-1.74342900
H	2.62732100	-4.07604000	-2.74638800
C	-0.76043400	0.69445000	-3.33074900
C	-1.92527000	-0.25512600	-3.06659800
C	-0.65811400	1.06915300	-4.80685500
H	0.16723300	0.15583900	-3.05740500
H	-1.94838500	-0.56863200	-2.00554800
H	-1.83848200	-1.16498200	-3.68736600
H	-2.90059500	0.21141900	-3.30018600
H	0.20494700	1.73066200	-5.00341000
H	-1.56453000	1.59861900	-5.15416200
H	-0.54140400	0.16828200	-5.43551400

IPr-TS$_{RE}$

C	0.11450800	-1.14595600	2.53642300
C	0.52743000	-2.60652400	2.46143200
Si	-0.66880600	-3.15039900	-0.02418300
C	0.89271300	-3.05669500	1.04735900
H	1.37257600	-4.05769600	1.06879600
H	1.64645000	-2.36647600	0.61834700
C	-0.22511600	-2.90362000	-1.84248600
H	-1.10464800	-2.98851400	-2.50464900
H	0.50432000	-3.66090900	-2.18269200
H	0.22049000	-1.90265300	-2.00169600
C	-1.39028700	-4.88048800	0.18981500
H	-0.67799700	-5.65482700	-0.15191700
H	-2.33063700	-5.02686700	-0.37083000
H	-1.61823000	-5.08750000	1.25205200
Ni	0.37121600	-0.10308700	0.90113100
C	-1.87997200	-1.86113000	0.61887900
C	-1.35532500	-0.71227100	1.16750300
C	-2.13877700	0.48476600	1.54673600
C	-3.06145700	1.00757500	0.61585000
C	-2.01073500	1.16694300	2.77271700
C	-3.83744000	2.12241600	0.90922500
H	-3.17393300	0.50987100	-0.35458800
C	-2.79488800	2.28006400	3.06864500
H	-1.28258400	0.82907600	3.51703000
C	-3.71847500	2.76496100	2.14319200
H	-4.54898200	2.49271200	0.16139700
H	-2.67562400	2.77786900	4.03898300
H	-4.33723300	3.63880100	2.37924900
C	-3.33122700	-2.10127000	0.49461300

C	-3.87717000	-2.67899900	-0.66888200
C	-4.22762100	-1.80285700	1.54140900
C	-5.24359400	-2.92116000	-0.79416200
H	-3.21030800	-2.92942700	-1.50491800
C	-5.59198000	-2.04611300	1.42032300
H	-3.83434200	-1.37657800	2.47230800
C	-6.11273400	-2.60220800	0.24930000
H	-5.63338500	-3.36427100	-1.71887900
H	-6.25996200	-1.80376500	2.25599900
H	-7.18804700	-2.79352400	0.15514500
H	1.37609100	-2.77711300	3.15615900
H	-0.29679000	-3.23843000	2.84832700
H	0.98433400	-0.47140000	2.78536400
H	-0.61491700	-0.98828900	3.34679900
N	0.91588700	1.57133700	-1.55663700
N	2.61790400	0.48518700	-0.81254200
C	1.27222800	0.68516600	-0.57398700
C	3.05990400	1.21416600	-1.90492500
H	4.09733200	1.19430100	-2.23456700
C	1.98654100	1.89883800	-2.37372900
H	1.88275700	2.59921300	-3.20177300
C	-0.32378100	2.29050500	-1.58985900
C	-0.43922500	3.43504500	-0.77543600
C	-1.62520400	4.16943600	-0.85749400
H	-1.75628100	5.05431900	-0.22312700
C	-2.64269400	3.79481800	-1.73029600
H	-3.56295700	4.38863100	-1.78302100
C	-2.50087700	2.66238700	-2.52325100
H	-3.31257300	2.37032300	-3.20133400
C	-1.33962000	1.88197800	-2.46778500

C	3.44829100	-0.34389400	0.01158300
C	3.95324300	-1.54713400	-0.51621100
C	4.75372700	-2.34064100	0.31527000
H	5.16218700	-3.28135700	-0.07113700
C	5.01231200	-1.97192600	1.62818300
H	5.62529200	-2.61629200	2.26875600
C	4.49157600	-0.78301800	2.13364300
H	4.70661200	-0.50560900	3.17049800
C	3.71500600	0.06595800	1.33987500
C	3.23108700	1.40273100	1.87413200
C	3.20613800	1.48782400	3.39282100
C	4.04819900	2.55403100	1.28716400
H	2.18326900	1.52870200	1.53483600
H	2.65282100	0.64625600	3.84788900
H	2.70872200	2.42054200	3.71200200
H	4.22093500	1.50041500	3.83126800
H	4.02018100	2.57100900	0.18333800
H	5.10883500	2.48233800	1.59379000
H	3.66400200	3.52777100	1.64304500
C	0.66209700	3.86442500	0.17448800
C	0.99481100	5.34779900	0.05133000
C	0.30434100	3.48630800	1.60899700
H	1.58038400	3.30594300	-0.08922200
H	1.23495900	5.63057200	-0.98932400
H	1.86824000	5.60171600	0.67859200
H	0.16172700	5.99151600	0.38699400
H	0.15742200	2.39084600	1.69459300
H	-0.63525700	3.97102000	1.93407500
H	1.10509600	3.78776900	2.31066400
C	3.64922400	-2.01419200	-1.92634000

C	4.81031200	-1.71360000	-2.87443000
C	3.31161300	-3.50222400	-1.97909700
H	2.76053700	-1.45852900	-2.28255200
H	5.06650900	-0.64098600	-2.90911300
H	4.57144900	-2.03319900	-3.90479300
H	5.72209600	-2.25522900	-2.56094000
H	2.56658400	-3.78205800	-1.21487000
H	4.20534800	-4.13324000	-1.82291700
H	2.90127900	-3.77035900	-2.96867300
C	-1.21775700	0.64967800	-3.33841600
C	-2.27579300	-0.38295800	-2.96281200
C	-1.27913000	1.00697600	-4.82128400
H	-0.22961600	0.19122400	-3.14763600
H	-2.14167300	-0.72877200	-1.92116900
H	-2.21530000	-1.26708900	-3.62302200
H	-3.30041900	0.02491100	-3.05197900
H	-0.50158500	1.74180800	-5.09850700
H	-2.25695700	1.44303200	-5.09708600
H	-1.13241800	0.10933200	-5.44834400

IPr-TS$_{LLHT}$

C	1.79270300	-1.75362800	1.67608200
C	0.54330700	-1.87955800	2.39554200
Si	-1.31400800	-3.08703400	0.55022300
C	-0.35335100	-3.08926300	2.20348100
H	-1.11057900	-3.13816700	3.00976800
H	0.24390500	-4.01902300	2.28285500
C	-0.04979600	-3.39871000	-0.81842100
H	-0.51023500	-3.53061400	-1.81374900
H	0.50853000	-4.32698700	-0.58765400
H	0.68779800	-2.57809800	-0.88940800

C	-2.49112600	-4.55525300	0.59438800
H	-1.94224000	-5.46975800	0.88911500
H	-2.96244000	-4.76315500	-0.38268700
H	-3.30866200	-4.40787700	1.32312500
Ni	0.49794000	-0.51864100	0.83645300
C	-2.23539100	-1.42952800	0.58139300
C	-1.49457600	-0.30962900	0.89555000
C	-2.09411100	0.96095000	1.32954100
C	-3.00914900	1.67202500	0.53553600
C	-1.77845400	1.49277700	2.59576200
C	-3.59032000	2.85366300	0.98835600
H	-3.27341900	1.27361200	-0.45128400
C	-2.38455700	2.65568000	3.06351500
H	-1.05338800	0.96660200	3.23272900
C	-3.29400000	3.34835000	2.26031000
H	-4.29834000	3.38762300	0.34139500
H	-2.13589400	3.03295100	4.06315000
H	-3.76461300	4.26995200	2.62248200
C	-3.72205900	-1.48178800	0.60592900
C	-4.43254500	-2.17003900	-0.39468800
C	-4.47762200	-0.91480600	1.65112600
C	-5.82365400	-2.25339500	-0.38250000
H	-3.87372100	-2.66006100	-1.20151900
C	-5.86710100	-1.00292000	1.67190800
H	-3.96143500	-0.40414000	2.47157000
C	-6.55170000	-1.66345900	0.65022400
H	-6.34248700	-2.79135100	-1.18527900
H	-6.42336300	-0.55292800	2.50323300
H	-7.64589200	-1.72933500	0.66680700
H	0.57383700	-1.48186600	3.42732900

H	-0.43978000	-0.90018400	2.00146800
H	2.07811000	-2.59352200	1.02068200
H	2.64662100	-1.33496100	2.22966400
N	0.92673000	1.29649500	-1.54258400
N	2.75018800	0.50085900	-0.74268800
C	1.38457900	0.44117700	-0.56307100
C	3.11257400	1.34689400	-1.77363900
H	4.15543400	1.51846100	-2.03543500
C	1.96152400	1.84113600	-2.28411600
H	1.77007300	2.51843900	-3.11524100
C	-0.41843400	1.69418300	-1.86015200
C	-0.76905100	3.03771800	-1.60770600
C	-1.99471900	3.48885900	-2.10875300
H	-2.30584800	4.52324300	-1.93268800
C	-2.83034300	2.64059800	-2.82582500
H	-3.78344200	3.01469200	-3.21780800
C	-2.47233500	1.31245100	-3.03234100
H	-3.15691700	0.66017500	-3.58200600
C	-1.25641600	0.80334500	-2.55783900
C	3.75486700	-0.11921400	0.06948500
C	4.23984000	-1.38529700	-0.30276500
C	5.26602300	-1.93403400	0.47185400
H	5.66461200	-2.92326000	0.22001300
C	5.78082400	-1.24785600	1.56917400
H	6.58450700	-1.69647000	2.16455300
C	5.27080800	-0.00175800	1.92086300
H	5.67240300	0.51683900	2.79924500
C	4.24519100	0.59310700	1.17724500
C	3.62417300	1.90393900	1.62182300
C	2.55776400	1.63687600	2.68536700

C	4.64829600	2.92065900	2.11118100
H	3.10792200	2.35249200	0.75081000
H	1.75937400	0.97970700	2.28441500
H	2.08545600	2.57664800	3.02497700
H	2.99518300	1.13482400	3.56970500
H	5.45293000	3.08644500	1.37264400
H	5.12385900	2.61182700	3.05963500
H	4.16156200	3.89350600	2.30284400
C	0.13688800	3.94973200	-0.79725100
C	-0.22522600	5.42446000	-0.90033600
C	0.17498000	3.52343800	0.66996800
H	1.16536300	3.84527800	-1.19527500
H	-0.27998800	5.77495200	-1.94651100
H	0.53229100	6.03824400	-0.38230100
H	-1.19569200	5.64201200	-0.41760100
H	0.41196400	2.45106700	0.79973300
H	-0.80538800	3.69699300	1.14825900
H	0.93058000	4.11172500	1.22448500
C	3.65064400	-2.12993000	-1.48495900
C	4.35393000	-1.75974200	-2.79083800
C	3.64679300	-3.64270300	-1.30305200
H	2.59410300	-1.80753100	-1.57116400
H	4.29811500	-0.67891900	-3.00687400
H	3.89752000	-2.29033000	-3.64623300
H	5.42365000	-2.03903500	-2.75555500
H	3.21826000	-3.94402000	-0.33069800
H	4.66286900	-4.07312600	-1.37078900
H	3.04318500	-4.12034100	-2.09526500
C	-0.87070400	-0.65035500	-2.76588800
C	-2.04181500	-1.49873300	-3.23723100

C	0.31421900	-0.85219400	-3.71261900
H	-0.57319500	-1.01744800	-1.76275800
H	-2.92717100	-1.37066000	-2.59245600
H	-1.77466200	-2.56990800	-3.23332700
H	-2.33868100	-1.25132500	-4.27381400
H	1.24232200	-0.36374100	-3.37192800
H	0.08510100	-0.46937700	-4.72520900
H	0.53716200	-1.93122300	-3.81076500

Reductive Elimination vs Ligand-to-Ligand H Transfer (L = PMe_3, 373.15 K, Toluene):

Energetic information (without concentration correction):

Chemical Structure	Electronic Energy (a.u.)	Enthalpy (a.u.)	Free Energy (a.u.)	Imaginary Frequency (cm^{-1})
PMe_3	-460.881147	-460.758468	-460.804039	
**P2-B**	-3456.033635	-3455.406307	-3455.547326	
**P-TS$_{RE}$**	-2995.088274	-2994.586683	-2994.703830	-276.68
**P2-TS$_{RE}$**	-3456.009622	-3455.383074	-3455.521212	-337.70
**P-TS$_{LLHT}$**	-2995.097012	-2994.598576	-2994.715999	-845.81

Cartesian coordinates:

PMe_3

P	0.00000500	-0.00002200	-0.60818900
C	1.42336400	-0.77382700	0.27946900
H	1.51746600	-1.83609200	-0.00805400
H	2.36606100	-0.27547800	-0.00842600
H	1.32323500	-0.71907000	1.38067700
C	-1.38189000	-0.84566800	0.27946400
H	-2.34886900	-0.39630400	-0.00852400
H	-1.42142300	-1.91138500	-0.00796300
H	-1.28475500	-0.78574900	1.38067300

C	-0.04148300	1.61953100	0.27943100
H	0.83131000	2.23224500	-0.00819600
H	-0.94454500	2.18671700	-0.00813500
H	-0.03851400	1.50523200	1.38060400

P2-B

C	0.95279500	-1.40380000	1.80502300
C	0.92344300	-2.83396200	1.27692000
Si	-1.73966700	-2.44955200	0.14100800
C	0.04357800	-3.06997100	0.04517800
H	0.01422700	-4.15494600	-0.18637900
H	0.51386900	-2.59395400	-0.84193300
C	-2.71130200	-3.22372600	-1.28574800
H	-3.79349900	-3.01506700	-1.20471600
H	-2.59528400	-4.32424400	-1.27448700
H	-2.38314800	-2.87182500	-2.28142800
C	-2.54568200	-3.04641900	1.73938500
H	-2.58359700	-4.15046000	1.79767600
H	-3.58504800	-2.67641800	1.81774000
H	-2.00346200	-2.68342900	2.63142500
Ni	1.26795400	-0.26696300	0.19600500
C	-1.76602100	-0.57028200	0.12864500
C	-0.60330300	0.12030800	0.22851700
C	-0.47659600	1.58581200	0.33539500
C	-1.03110500	2.46484700	-0.61750600
C	0.31552600	2.15262300	1.35837800
C	-0.77318200	3.83389300	-0.57373600
H	-1.68950100	2.05995700	-1.39685600
C	0.56969200	3.52166000	1.40226600
H	0.70954000	1.48986600	2.13992700
C	0.03914600	4.37009500	0.42707100

H	-1.21992400	4.49386900	-1.32781300
H	1.18241200	3.93502500	2.21374100
H	0.24146000	5.44697900	0.45915900
C	-3.08520000	0.11050200	0.20000100
C	-4.03441200	-0.03915900	-0.82670600
C	-3.42955700	0.92665400	1.29198100
C	-5.26816100	0.60889800	-0.77209400
H	-3.78283500	-0.65612200	-1.69955300
C	-4.66299200	1.57117300	1.35145300
H	-2.70475700	1.05131600	2.10659900
C	-5.58984800	1.41685800	0.31883900
H	-5.98490600	0.48406000	-1.59319600
H	-4.90527600	2.20181700	2.21565200
H	-6.56062600	1.92427700	0.36517900
H	1.95580700	-3.16792300	1.03826700
H	0.58516200	-3.52413100	2.08347700
H	1.72658300	-1.32778100	2.59907800
H	-0.00544600	-1.15729500	2.30125700
P	3.38038400	-0.04675600	0.77584400
C	4.55314900	0.94271900	-0.23892000
H	4.14076000	1.95727500	-0.38193300
H	4.70646600	0.49583000	-1.23605700
H	5.54006600	1.03688400	0.25051600
C	3.54905800	0.86236400	2.36113100
H	2.93993800	0.38594200	3.14701900
H	3.18643000	1.89670600	2.23184900
H	4.60138100	0.89881200	2.69797900
C	4.37287100	-1.55193300	1.11001500
H	4.44764300	-2.16938900	0.19769100
H	3.87462900	-2.16095200	1.88328400

H	5.39292100	-1.30518200	1.45715200
P	1.39929800	0.04484800	-1.99528800
C	1.91745400	1.63157800	-2.76015400
H	2.94563700	1.89045100	-2.45692000
H	1.25141700	2.43793200	-2.40456000
H	1.87594200	1.59448400	-3.86463100
C	2.55415900	-1.15087100	-2.78103300
H	2.26477300	-2.17988300	-2.50309400
H	3.58797600	-0.99283400	-2.42938700
H	2.54544900	-1.07231400	-3.88392400
C	-0.12931100	-0.31543900	-2.93674100
H	-0.91480400	0.41482400	-2.68052000
H	-0.51298700	-1.31157800	-2.65544100
H	0.04680300	-0.28979000	-4.02781000

P-TS$_{RE}$

C	0.65247300	-1.11235400	2.23541800
C	0.35442800	-2.59880500	2.26409100
Si	-1.50214000	-2.33415200	0.10757700
C	-1.05944700	-2.96794800	1.83104900
H	-1.79477000	-2.54615500	2.54643800
H	-1.19103200	-4.06702600	1.88157700
C	-0.31333600	-3.12059900	-1.12902300
H	-0.56317900	-2.86110800	-2.17455600
H	-0.29987000	-4.22434500	-1.05288100
H	0.71272700	-2.75113000	-0.92716600
C	-3.27690600	-2.82386300	-0.27549500
H	-3.42814600	-3.90448100	-0.09508700
H	-3.56930700	-2.62534800	-1.32150500
H	-3.99178800	-2.27823000	0.36718400
Ni	1.62354400	-0.58472600	0.64026500

C	-1.29105300	-0.46774600	0.20184600
C	-0.11633600	0.00942000	0.71875300
C	0.30981800	1.42179500	0.82151800
C	0.12874900	2.29196600	-0.27950300
C	0.99646600	1.94033600	1.94135000
C	0.62295000	3.59164200	-0.26759500
H	-0.41879700	1.93078200	-1.15769900
C	1.48897400	3.24392500	1.94949400
H	1.13972600	1.31981100	2.83167100
C	1.31273200	4.07915400	0.84540600
H	0.46367700	4.23584300	-1.14104200
H	2.01793500	3.61197000	2.83683400
H	1.70289700	5.10324700	0.85444300
C	-2.37765900	0.43242900	-0.23024500
C	-2.98394000	0.27308200	-1.49063600
C	-2.84986100	1.47440300	0.59050100
C	-4.00288700	1.12152400	-1.91863900
H	-2.62318200	-0.52242200	-2.15769200
C	-3.87049800	2.32058400	0.16644900
H	-2.40001400	1.61415000	1.58132200
C	-4.45265500	2.15183900	-1.09180700
H	-4.44855100	0.97877200	-2.91063600
H	-4.21981500	3.12217500	0.82858900
H	-5.25661500	2.81848400	-1.42462500
H	1.08794200	-3.12690000	1.62099200
H	0.54931600	-2.97898200	3.29167600
H	1.72728300	-0.91491100	2.54453900
H	0.03049900	-0.54648000	2.95021300
P	2.71701200	-0.32611500	-1.17893400
C	1.63348500	-0.23209600	-2.65667000

H	0.87331100	0.55363300	-2.50725500
H	1.10096600	-1.18813700	-2.79344600
H	2.20709300	-0.00621900	-3.57464300
C	3.67979600	1.22804700	-1.34274000
H	4.48934200	1.25287800	-0.59321900
H	3.01758800	2.08900800	-1.14291200
H	4.12360800	1.34100200	-2.34942400
C	3.94366800	-1.57870200	-1.72660400
H	3.45876400	-2.56692700	-1.80943500
H	4.75755000	-1.66763800	-0.98684400
H	4.38662800	-1.32133900	-2.70641700

P2-TS$_{RE}$

C	0.46259400	-0.59728200	2.01227600
C	0.67150400	-2.10307900	2.13852200
Si	-1.10358100	-2.54630000	-0.03389400
C	-0.49806900	-2.98070900	1.70364900
H	-1.35219500	-2.83449800	2.39670500
H	-0.21431100	-4.04794600	1.79317500
C	0.34170900	-2.91836500	-1.20519300
H	0.07802100	-2.82952500	-2.27569900
H	0.70490900	-3.95263000	-1.04537200
H	1.19340200	-2.23354600	-1.01265700
C	-2.54548800	-3.67029600	-0.47834300
H	-2.27584000	-4.72676700	-0.29091700
H	-2.84151800	-3.59616000	-1.54001600
H	-3.44279200	-3.44289700	0.12500000
Ni	1.27440400	0.02860200	0.17994700
C	-1.57383900	-0.74671200	0.14712700
C	-0.57104600	0.10185300	0.61299900
C	-0.70310800	1.57805300	0.76644900

C	-1.52687400	2.32550200	-0.10700800
C	0.08336000	2.32618700	1.67240700
C	-1.54557000	3.71652000	-0.09100800
H	-2.18091200	1.80196700	-0.81005500
C	0.06165000	3.72041700	1.68915400
H	0.71858300	1.81775700	2.40266500
C	-0.74658700	4.43105000	0.80350000
H	-2.20381000	4.25023300	-0.78750500
H	0.68735000	4.25632500	2.41330100
H	-0.76309900	5.52684800	0.81690400
C	-2.99051200	-0.36322200	0.09777000
C	-3.81348000	-0.71249100	-0.99415300
C	-3.60133300	0.35385600	1.15051100
C	-5.15955100	-0.35731700	-1.03963600
H	-3.36885100	-1.25916100	-1.83673700
C	-4.94463400	0.71242900	1.10534700
H	-2.99527000	0.62737700	2.02423200
C	-5.73672400	0.36184200	0.00863700
H	-5.76543100	-0.64011100	-1.90963400
H	-5.38471700	1.26762000	1.94323100
H	-6.79583000	0.64243900	-0.02551500
H	1.56969700	-2.39957200	1.55691300
H	0.93023800	-2.32078800	3.19916400
H	1.34472600	-0.07651600	2.43256200
H	-0.35349100	-0.26842100	2.68244500
P	1.21755500	0.63529400	-1.92184900
C	-0.34360300	0.35211800	-2.83627100
H	-1.17932500	0.85738400	-2.32669900
H	-0.58131300	-0.72521000	-2.84546900
H	-0.28066800	0.71779600	-3.87739600

C	1.50769300	2.42189700	-2.23082800
H	2.50318800	2.71511700	-1.85408700
H	0.75782600	3.01264800	-1.67547400
H	1.44395400	2.67981700	-3.30454100
C	2.40875800	-0.12776700	-3.10018500
H	2.28366000	-1.22529100	-3.10078800
H	3.45000900	0.09314300	-2.80709400
H	2.25935300	0.23857200	-4.13292500
P	3.43697700	-0.28453800	0.53552600
C	3.96958200	-0.49064700	2.28573200
H	3.43087500	-1.33372000	2.75317800
H	3.72848100	0.41918700	2.86354300
H	5.05554400	-0.67843100	2.37159100
C	4.21464700	-1.77186100	-0.21809700
H	4.08332600	-1.76147100	-1.31354600
H	3.71878200	-2.68263500	0.16127500
H	5.29520400	-1.83965400	0.00797100
C	4.60581100	1.03257600	0.00532800
H	4.32324100	1.98924000	0.47889000
H	4.55371900	1.17990300	-1.08713200
H	5.65280000	0.80027700	0.27526600

P-TS$_{LLHT}$

C	3.00761600	-1.71278700	1.15851000
C	1.85591800	-1.81890200	2.00896700
Si	-0.34928000	-2.49347900	0.17805900
C	0.73043900	-2.79674400	1.72996500
H	0.02871900	-2.80771400	2.58688300
H	1.13541600	-3.82780400	1.66195500
C	0.66351100	-2.89233800	-1.36800600
H	0.06433800	-2.77650600	-2.29065300

H	1.03315000	-3.93533300	-1.34417300
H	1.54567300	-2.23243100	-1.45773500
C	-1.77126200	-3.72244100	0.28507600
H	-1.39490700	-4.74813900	0.45753600
H	-2.38344900	-3.74880200	-0.63377300
H	-2.45509100	-3.47541900	1.11786200
Ni	1.69863900	-0.31915300	0.68558300
C	-0.99914400	-0.71566100	0.19112400
C	-0.12221800	0.27650900	0.53981300
C	-0.40751200	1.71629500	0.60562600
C	-1.14868700	2.38873800	-0.38751200
C	0.16328300	2.49893100	1.62832800
C	-1.30691800	3.77252200	-0.35588200
H	-1.60673900	1.81073200	-1.19939600
C	-0.01876700	3.87839600	1.67668400
H	0.76552100	1.99722400	2.39777900
C	-0.74997100	4.52633100	0.67893900
H	-1.88080500	4.26874400	-1.14795000
H	0.42695600	4.45728900	2.49467700
H	-0.88179100	5.61421300	0.70530300
C	-2.43129800	-0.47613700	-0.10790900
C	-3.01455900	-0.98176300	-1.28310100
C	-3.25675500	0.24999300	0.76931000
C	-4.35707100	-0.75540900	-1.58231100
H	-2.38945700	-1.54865500	-1.98759100
C	-4.59946300	0.47346500	0.47582400
H	-2.82594400	0.64435500	1.69793500
C	-5.15719300	-0.02457600	-0.70347900
H	-4.78254500	-1.15282100	-2.51184000
H	-5.22088000	1.04205000	1.17834300

H	-6.21428600	0.15186600	-0.93392200
H	2.03049200	-1.60626200	3.07703900
H	0.91953700	-0.45049400	1.95630900
H	3.12353600	-2.43836700	0.33613000
H	3.96303500	-1.41607800	1.61895600
P	2.47773700	0.61131600	-1.10592900
C	1.35170300	0.66165700	-2.54748300
H	0.41621500	1.17158800	-2.25973400
H	1.08965400	-0.36193600	-2.86510100
H	1.80376800	1.19639400	-3.40250400
C	2.88091400	2.38282500	-0.88616000
H	3.62052000	2.50980800	-0.07737200
H	1.96979600	2.93523200	-0.59697400
H	3.28919500	2.82452500	-1.81364600
C	4.01341900	-0.05863500	-1.84320400
H	3.85770300	-1.11258000	-2.13283000
H	4.82434600	-0.04083300	-1.09525000
H	4.33439300	0.50977700	-2.73517700

Ni-Catalyzed Cycloaddition of 5a with 2a (373.15 K, 1,4-Dioxane):

Energetic information:

Chemical Structure	Electronic Energy (a.u.)	Enthalpy (a.u.)	Free Energy (a.u.)	Imaginary Frequency (cm^{-1})
**2a**	-539.026574	-538.819456	-538.884104	
**5a**	-639.299391	-639.103012	-639.164050	
**6aa**	-1178.424874	-1178.018624	-1178.114651	
**6aa'**	-1178.418638	-1178.011768	-1178.107981	
cod	-311.776237	-311.584110	-311.634271	
PMe_3	-460.878814	-460.756135	-460.801706	

**IM1**	-2969.010263	-2968.553245	-2968.668360	
**IM1b**	-2741.736954	-2741.295601	-2741.398591	
**IM1c**	-3147.399496	-3146.869525	-3146.996845	
**IM1d**	-3069.241207	-3068.796642	-3068.910102	
**IM1e**	-2608.329955	-2608.008030	-2608.097787	
**TS1**	-3147.397882	-3146.868832	-3146.993771	-88.18
**TS1'**	-3069.232506	-3068.788858	-3068.901275	-94.36
**IM2**	-3069.266376	-3068.819898	-3068.932763	
**IM2'**	-3069.256721	-3068.810068	-3068.922793	
**TS2**	-3147.406562	-3146.877120	-3147.001476	-85.91
**TS2'**	-3147.402081	-3146.872723	-3146.997333	-59.43
**IM3**	-3608.354793	-3607.699406	-3607.845120	
**IM3'**	-3608.349030	-3607.694000	-3607.840870	
**TS3**	-3608.334603	-3607.680851	-3607.825460	-216.07
**TS3'**	-3608.319044	-3607.664709	-3607.809215	-297.99

Cartesian coordinates:

2a

C	-0.61113400	-0.00066400	-0.00012800
C	0.61101100	-0.00071100	0.00000500
C	-2.03101500	-0.00052400	-0.00022800
C	-2.75191900	-1.21223400	-0.00615300
C	-2.75095300	1.21180100	0.00609500
C	-4.14257600	-1.20688600	-0.00604700
H	-2.20077100	-2.15881500	-0.01089600
C	-4.14160900	1.20763700	0.00615800
H	-2.19896800	2.15790800	0.01089100
C	-4.84344200	0.00065600	0.00007600
H	-4.68779300	-2.15775800	-0.01074800
H	-4.68605400	2.15894800	0.01100000

H	-5.93937200	0.00109100	0.00016100
C	2.03098900	-0.00055900	0.00011100
C	2.75196300	-1.21222400	0.00609500
C	2.75094800	1.21177100	-0.00608400
C	4.14262400	-1.20685000	0.00608300
H	2.20088900	-2.15884400	0.01092300
C	4.14160100	1.20763700	-0.00606200
H	2.19896100	2.15787900	-0.01101700
C	4.84348800	0.00068400	0.00003000
H	4.68783600	-2.15772800	0.01098600
H	4.68599900	2.15898300	-0.01104500
H	5.93941600	0.00113600	0.00002800

5a

C	-1.29331700	-1.51034800	0.00033300
C	-0.32680300	-0.50258800	-0.00075100
C	-0.71274500	0.85555700	-0.00158400
C	-2.06312900	1.21765200	-0.00086600
C	-3.01952900	0.20106500	0.00043100
C	-2.64157300	-1.14829500	0.00080700
H	-1.01490000	-2.57252500	0.00087200
H	-2.37097100	2.27111700	-0.00134300
H	-4.08576200	0.45947400	0.00100200
H	-3.41721400	-1.92386200	0.00159400
Si	1.47658600	0.00207300	-0.00011300
C	0.55930400	1.68039900	-0.00298200
H	0.67565200	2.32607200	-0.89429000
H	0.67562600	2.32973600	0.88566300
C	2.47283700	-0.39451500	1.54052500
H	2.73517400	-1.46712100	1.58864600
H	3.42131900	0.17295900	1.56471200

H	1.91377900	-0.15027200	2.46060800
C	2.47671700	-0.40032800	-1.53683300
H	3.42545500	0.16669800	-1.56104100
H	2.73885700	-1.47320900	-1.57990700
H	1.92020300	-0.15969000	-2.45940600

6aa

Si	0.67654100	-2.37303500	-0.07657500
C	2.13018500	-1.79977500	-1.12430200
H	1.70678600	-1.55790800	-2.12233900
H	2.90634500	-2.56705800	-1.29469800
C	1.25245500	-2.95541400	1.61528900
H	0.40839000	-3.21399500	2.27967300
H	1.89067600	-3.85496500	1.53490000
H	1.84487600	-2.17295000	2.12295600
C	-0.33404400	-3.69187000	-0.94487600
H	0.26388600	-4.60313200	-1.12902900
H	-1.21662100	-3.99200300	-0.35190400
H	-0.70790900	-3.33421100	-1.92117400
C	-0.27994800	-0.74516800	0.00567700
C	0.42629500	0.42848300	-0.00956000
C	-0.26984100	1.74468300	0.03249900
C	-1.21782600	2.03640000	1.02366000
C	0.00196700	2.72238200	-0.93726300
C	-1.88694400	3.25831900	1.03522100
H	-1.43333500	1.28633900	1.79390900
C	-0.67641300	3.93922900	-0.93613700
H	0.75315700	2.51538600	-1.71019100
C	-1.62541700	4.21148000	0.05045800
H	-2.62204900	3.46751300	1.82112900
H	-0.46024700	4.68360300	-1.71158300

H	-2.15629200	5.17054200	0.05557600
C	-1.75562300	-0.81172500	-0.01527300
C	-2.43459600	-1.64835000	0.88898900
C	-2.52741100	-0.10962400	-0.95907200
C	-3.82414300	-1.75142600	0.87889100
H	-1.85572800	-2.21515300	1.63136600
C	-3.91475800	-0.22208000	-0.97997900
H	-2.02294900	0.53386800	-1.68969500
C	-4.57247300	-1.03702900	-0.05667500
H	-4.32662500	-2.39882800	1.60733600
H	-4.49106300	0.33430800	-1.72871700
H	-5.66521800	-1.12085600	-0.07184400
C	3.93497800	1.69042300	0.66945100
C	4.72941600	0.66154100	0.17026300
C	4.12094500	-0.44975900	-0.41032800
C	2.73021400	-0.56964300	-0.50776700
C	1.91342800	0.47702300	0.00196400
C	2.54988900	1.59330400	0.57785900
H	4.39060200	2.57128800	1.13589100
H	5.82241100	0.72035800	0.23072700
H	4.74360900	-1.26040200	-0.80971600
H	1.93371900	2.40233600	0.98555100

6aa'

C	1.11761300	1.61087100	0.75671700
C	2.43142300	1.19505700	0.14102100
Si	1.49011000	-1.44716300	0.46771000
C	2.76463300	-0.16389800	-0.02689800
C	1.58763300	-2.96862900	-0.62472600
H	0.81718300	-3.71385100	-0.35743500
H	2.56918300	-3.46927000	-0.53625200

H	1.43868800	-2.71125100	-1.68880700
C	1.74323500	-1.93615300	2.27124600
H	2.74623200	-2.37753600	2.41928300
H	1.00265600	-2.68166900	2.61352000
H	1.66784400	-1.06277100	2.94363200
C	-0.13529400	0.83405000	0.39495000
C	-0.13442100	-0.51837900	0.23479600
C	-1.37231200	1.65321400	0.32161700
C	-1.42408900	2.81548300	-0.46553600
C	-2.52362400	1.28999000	1.03929400
C	-2.59177800	3.57062800	-0.55541500
H	-0.54135700	3.11696800	-1.04397700
C	-3.68608500	2.05221600	0.96371700
H	-2.49846000	0.39177800	1.66732200
C	-3.72787000	3.19283900	0.16058100
H	-2.61410200	4.46336800	-1.19144300
H	-4.56996700	1.75213900	1.53858100
H	-4.64469400	3.79014200	0.09623700
C	-1.32826800	-1.29057200	-0.17470700
C	-2.10596200	-0.91893100	-1.28578900
C	-1.68987000	-2.46262900	0.51239000
C	-3.20963000	-1.67250600	-1.67562200
H	-1.83184700	-0.01999900	-1.85096600
C	-2.80035300	-3.21323000	0.13035400
H	-1.09617800	-2.78073800	1.38027100
C	-3.56750700	-2.82044700	-0.96625900
H	-3.79770200	-1.36057900	-2.54692800
H	-3.06715800	-4.11503600	0.69413500
H	-4.43807500	-3.41171800	-1.27270400
H	1.21732800	1.57071100	1.86431000

H	0.95107300	2.68335800	0.55589500
C	4.01091000	-0.49011600	-0.58280100
H	4.28086400	-1.54515200	-0.73052700
C	3.34879100	2.18156900	-0.24263500
H	3.08729900	3.24056500	-0.11589600
C	4.58535900	1.83898600	-0.78635000
H	5.28795700	2.62654000	-1.08373300
C	4.92108100	0.49639300	-0.95861600
H	5.89002300	0.21945700	-1.39043100

cod

H	-2.74983900	-0.31646200	0.63756600
C	-1.91644900	-0.01172500	-0.03034200
C	-1.09833000	1.07964700	0.66224200
H	-2.42573500	0.43265500	-0.90972200
C	-1.19628200	-1.23888900	-0.49244700
C	-0.03561100	1.68866200	-0.20961500
H	-1.79955400	1.87744400	0.96724500
H	-0.68187300	0.69633300	1.60805400
C	0.03544000	-1.68849900	-0.20974000
H	-1.79290200	-1.86639800	-1.17210300
C	1.19612400	1.23915500	-0.49238700
H	-0.32933500	2.62485000	-0.70826600
C	1.09864600	-1.08000800	0.66195100
H	0.32885700	-2.62488000	-0.70820700
C	1.91629400	0.01192200	-0.03037200
H	1.79280200	1.86645200	-1.17218100
H	1.80020500	-1.87797100	0.96569500
H	0.68305800	-0.69785700	1.60865500
H	2.42549000	-0.43223600	-0.90990700
H	2.74983700	0.31648500	0.63743400

IM1

Ni	0.81584800	-0.08853000	-0.00780100
P	1.94408900	-1.91918300	0.24419100
C	2.67509400	-2.79095200	-1.20160200
H	1.87790200	-3.06392800	-1.91424200
H	3.37738000	-2.12531400	-1.73274900
H	3.21397600	-3.71009400	-0.90540200
C	1.05078200	-3.29407400	1.06559800
H	0.64816400	-2.94334300	2.03150800
H	0.18785000	-3.61120200	0.45638600
H	1.70499700	-4.16703200	1.24596800
C	3.41333300	-1.81621900	1.35127300
H	4.17135900	-1.12719700	0.94061300
H	3.10525000	-1.42151100	2.33532800
H	3.88880800	-2.80288400	1.50490900
P	2.30051900	1.46966100	-0.24431100
C	3.79066900	1.05009900	-1.24355200
H	4.38026300	0.25255100	-0.75938900
H	3.47684300	0.67587500	-2.23379200
H	4.45046300	1.92475200	-1.39477300
C	3.09427300	2.24735800	1.22194300
H	2.32279000	2.69628000	1.87062100
H	3.62144600	1.48306300	1.81896300
H	3.81711500	3.03390200	0.93600800
C	1.73388800	2.95108300	-1.16512100
H	1.34290500	2.64188200	-2.14975100
H	0.90330300	3.44158900	-0.63012900
H	2.54775800	3.68382800	-1.31779200
C	-0.99568800	-0.55033300	-0.06498500
C	-0.85734500	0.74504100	0.04547600

C	-1.97307100	-1.61430100	-0.07588800
C	-3.10138100	-1.56461300	0.76821700
C	-1.84260200	-2.72352900	-0.93363100
C	-4.04750900	-2.58536300	0.75939900
H	-3.21912000	-0.70550400	1.43964200
C	-2.79776300	-3.73660000	-0.95162200
H	-0.97453000	-2.76793500	-1.60457800
C	-3.90273300	-3.67669900	-0.10026100
H	-4.91383700	-2.52761200	1.42964000
H	-2.67939100	-4.58460800	-1.63722000
H	-4.65138700	-4.47736100	-0.10892700
C	-1.59060900	1.98973300	0.06416500
C	-1.19082700	3.06355600	0.88267600
C	-2.74474000	2.16100500	-0.72769500
C	-1.91155200	4.25449900	0.91329200
H	-0.30143900	2.93733200	1.51402800
C	-3.45628600	3.35698200	-0.70649800
H	-3.07133800	1.33289500	-1.36823700
C	-3.04546400	4.41039000	0.11378500
H	-1.58577000	5.07208200	1.56792400
H	-4.34749300	3.46887500	-1.33601500
H	-3.60993800	5.34990400	0.13187100

IM1b

Ni	-0.34604700	0.01412100	-0.19167000
P	-1.65169600	-1.68837000	-0.04179900
C	-1.04964100	-3.30058700	-0.69308200
H	-0.78060100	-3.19571500	-1.75864400
H	-0.13454900	-3.59766900	-0.15307900
H	-1.79887800	-4.10840700	-0.59320800
C	-3.30778900	-1.63668400	-0.85268200

H	-3.91713200	-0.81646200	-0.43414800
H	-3.19125700	-1.44484300	-1.93388500
H	-3.86627200	-2.58309900	-0.72543300
C	-2.14469400	-2.20315100	1.65805900
H	-1.24367100	-2.43943700	2.25080200
H	-2.66713100	-1.37654600	2.17025400
H	-2.80429300	-3.09125400	1.65377400
P	-1.56794900	1.75625000	0.07743900
C	-0.80911400	3.40318400	0.39470500
H	-0.28485600	3.39954800	1.36601100
H	-0.05904900	3.62271400	-0.38511100
H	-1.55873000	4.21683600	0.40444400
C	-2.88669300	1.78650900	1.36687000
H	-3.58836900	0.94753600	1.21578000
H	-2.43622600	1.66248800	2.36751800
H	-3.46446900	2.72994800	1.35940700
C	-2.56441300	2.16577000	-1.41807000
H	-1.88937500	2.34650700	-2.27259400
H	-3.21531500	1.31672200	-1.68830200
H	-3.19425700	3.06315300	-1.27023300
C	4.48215900	0.47444100	-0.50005900
H	5.22526900	0.72387100	-1.27261600
C	3.71271100	1.64714600	0.04025900
H	3.71616400	2.43198000	-0.74183800
H	4.26975800	2.10482800	0.88673400
C	2.25033500	1.41623000	0.46064800
H	2.19189000	0.90668500	1.43883500
H	1.80480100	2.41386300	0.63975300
C	1.45914500	0.66056600	-0.57682300
H	1.45802700	1.17223300	-1.55911400

C	1.39819700	-0.76585600	-0.63819700
H	1.36292000	-1.18789100	-1.65790800
C	2.02369700	-1.71418600	0.37476100
H	2.05210000	-2.72084700	-0.08511000
H	1.39004200	-1.83340400	1.27830300
C	3.44855800	-1.37679200	0.87520000
H	3.38525700	-0.67567300	1.72530000
H	3.88623400	-2.29887400	1.30236900
C	4.37478300	-0.81749400	-0.15852300
H	5.01250500	-1.53696000	-0.69428900

IM1c

C	-1.98705000	0.06257100	-1.54394100
C	-2.26952200	-0.01553300	-0.15150300
C	-3.08170000	-1.09102600	0.33715500
C	-3.64368400	-2.02394300	-0.51644900
C	-3.32743000	-1.95530200	-1.88918500
C	-2.51601400	-0.94697700	-2.38765600
H	-1.57862100	0.96594400	-2.01338300
H	-4.30218700	-2.81635400	-0.13775100
H	-3.74334800	-2.70159300	-2.57795700
H	-2.31890300	-0.89628600	-3.46571300
Si	-2.36466000	0.80681700	1.54252300
C	-3.20587600	-0.88786200	1.83003300
H	-4.23872500	-0.93101300	2.22227900
H	-2.60379400	-1.61442500	2.41250400
C	-0.80958600	1.15064100	2.52994600
H	-0.11277100	0.29337600	2.51012000
H	-0.26099700	2.02677500	2.13772500
H	-1.06384600	1.36266100	3.58620000
C	-3.55008300	2.25803400	1.67908000

H	-4.46743000	2.09218000	1.08712800
H	-3.85529000	2.41696900	2.73006600
H	-3.09040400	3.20094200	1.33134100
Ni	-0.22812600	-0.47372600	-0.45474700
P	0.09054000	-2.64258400	-0.09570200
C	-1.17362400	-3.96327300	0.10462800
H	-1.87807500	-3.71398300	0.91626400
H	-1.76231300	-4.07999800	-0.82033500
H	-0.68954700	-4.92932600	0.34084900
C	1.05769100	-2.91204400	1.44239800
H	1.98838900	-2.31876600	1.41397000
H	0.47251100	-2.57478300	2.31667600
H	1.31874300	-3.97699100	1.58544000
C	1.17483200	-3.44248800	-1.34084900
H	0.68265800	-3.42934000	-2.32896900
H	2.12031300	-2.88106200	-1.42577100
H	1.40799200	-4.48971800	-1.07357200
C	0.67696700	1.17858800	-0.48195900
C	1.52848700	0.21482500	-0.29564600
C	0.55225400	2.61021900	-0.62058400
C	-0.70653600	3.24128400	-0.64259400
C	1.70090100	3.42607200	-0.70539600
C	-0.81623000	4.62698700	-0.73597000
H	-1.61300500	2.62655300	-0.58209400
C	1.58923000	4.80832900	-0.80446800
H	2.68861100	2.94975700	-0.69116100
C	0.33058900	5.41712100	-0.81854200
H	-1.80829900	5.09476700	-0.74672000
H	2.49505800	5.42306100	-0.86988300
H	0.24593000	6.50731200	-0.89512900

C	2.88671600	-0.15785400	0.02361500
C	3.72466600	-0.79606000	-0.91057600
C	3.40459200	0.10123400	1.30911700
C	5.02465900	-1.16398400	-0.57075700
H	3.34509000	-0.98268300	-1.92229600
C	4.70190400	-0.27486500	1.64578600
H	2.76395400	0.60205700	2.04588700
C	5.51879800	-0.91337400	0.71022300
H	5.66165700	-1.65211300	-1.31819700
H	5.08209500	-0.06453300	2.65275400
H	6.53954200	-1.20964800	0.97742600

IM1d

C	-0.60478400	-0.91003700	1.72180000
C	-1.56648600	-0.55354100	0.71964000
C	-2.34347200	0.64686200	0.81592800
C	-2.09385400	1.59637900	1.79791800
C	-0.97104600	1.37155300	2.62082300
C	-0.24318300	0.17640200	2.58345600
H	-0.34056700	-1.94727900	1.97168800
H	-2.68623400	2.51520600	1.88751600
H	-0.66829700	2.14149700	3.34347800
H	0.55464000	0.02796000	3.32447000
Si	-2.56444600	-1.09004200	-0.75994700
C	-3.35693900	0.58975900	-0.31016500
H	-4.40344600	0.53327900	0.04858700
H	-3.30974600	1.43560900	-1.02219100
C	-1.63299000	-1.19194300	-2.39371000
H	-1.08331300	-2.14787700	-2.47997200
H	-2.30949200	-1.12770800	-3.26657200
H	-0.88630300	-0.38048200	-2.47607500

C	-3.69397500	-2.58591700	-0.60633200
H	-4.40413100	-2.64831000	-1.45173800
H	-3.11831600	-3.52978700	-0.59842500
H	-4.28632000	-2.55469200	0.32502300
Ni	0.47763800	-0.03581600	0.35979800
P	2.19371000	-1.29190500	0.11545700
C	2.37413100	-2.80594300	1.15382600
H	2.35245800	-2.52931400	2.22332300
H	1.52304300	-3.48690400	0.97610500
H	3.31193900	-3.35790100	0.95354200
C	3.85313400	-0.53771500	0.41344500
H	4.01337400	0.31094500	-0.27487200
H	3.90209700	-0.13798700	1.44186700
H	4.68209900	-1.25860900	0.27740100
C	2.49302600	-2.03204200	-1.54840700
H	1.65924900	-2.70483600	-1.81424600
H	2.52983300	-1.23915600	-2.31580500
H	3.43598000	-2.61018800	-1.59146200
P	0.74586100	1.81142900	-0.74495300
C	-0.68656800	2.81272500	-1.32044800
H	-1.24935300	2.25437800	-2.08945400
H	-1.37190500	2.99914400	-0.47457500
H	-0.37570600	3.78337700	-1.75054700
C	1.73918800	1.79742500	-2.29824400
H	2.74406900	1.38288200	-2.10407000
H	1.25465000	1.14923400	-3.05012600
H	1.85686300	2.80742000	-2.73449300
C	1.66035300	3.08550000	0.21940200
H	1.06913500	3.35404600	1.11217100
H	2.62369700	2.67802300	0.57247100

H	1.85560800	4.00178800	-0.36926200

IM1e

C	0.80732100	1.37045000	-1.51523100
C	1.47027500	0.63108000	-0.50501300
C	1.24946200	0.94133700	0.87963500
C	0.37541300	1.98660300	1.26399200
C	-0.26082400	2.73635500	0.23973400
C	-0.03929300	2.44173300	-1.13082500
H	0.93475600	1.13164500	-2.57839100
H	0.18601500	2.21381800	2.32016800
H	-0.95118100	3.54508000	0.50807500
H	-0.56346000	3.02620000	-1.89615500
Si	2.46903600	-0.86793700	-0.00222100
C	2.03939300	-0.07024200	1.68308900
H	2.89020500	0.37630600	2.23387700
H	1.43506100	-0.64283400	2.41123700
C	1.66897800	-2.53009900	-0.34210300
H	1.70242300	-2.78833100	-1.41672100
H	2.16845400	-3.34921000	0.20741800
H	0.60735300	-2.51186100	-0.03934200
C	4.28957500	-0.91696200	-0.45813200
H	4.82688500	-1.69698800	0.11221800
H	4.43337800	-1.14385700	-1.53032000
H	4.78882600	0.04672500	-0.25578700
Ni	-0.58738300	0.68772300	-0.09312300
P	-2.16425500	-0.67431400	0.00674500
C	-2.22615900	-1.82419100	1.45301900
H	-1.31440800	-2.44663200	1.48429200
H	-2.25345600	-1.23989500	2.38943700
H	-3.10692500	-2.49404400	1.42861900

C	-2.38755500	-1.90200000	-1.35579600
H	-2.54402000	-1.37052500	-2.31062100
H	-1.47550800	-2.51367800	-1.46943000
H	-3.24686700	-2.57771900	-1.18293200
C	-3.89070800	-0.02269700	0.08868300
H	-3.99902900	0.63674100	0.96724000
H	-4.10187300	0.58773900	-0.80644300
H	-4.64786200	-0.82730100	0.15462000

IM2

C	-2.19363200	0.88059000	-1.28366400
C	-1.64503100	0.06710100	-0.27515700
C	-2.54370300	-0.67515000	0.52815100
C	-3.92639000	-0.57281000	0.33285200
C	-4.44420800	0.24797400	-0.67067500
C	-3.57299800	0.96553600	-1.49207100
H	-1.52880800	1.45894600	-1.94564200
H	-4.60816500	-1.15789700	0.96519600
H	-5.52868300	0.31788400	-0.82054500
H	-3.96988900	1.59509600	-2.29922100
Si	-0.33956900	-2.06179300	0.43175500
C	-1.88971400	-1.63670200	1.47139400
H	-2.53685800	-2.49229400	1.74964400
H	-1.58390700	-1.14717100	2.41950700
C	0.83647800	-3.08738000	1.53385400
H	1.73993900	-3.47357400	1.02582300
H	0.29102000	-3.97283800	1.91690800
H	1.17162200	-2.51389700	2.41859300
C	-0.90221000	-3.20388500	-0.97005200
H	-1.31493100	-4.15020900	-0.56915900
H	-0.08140700	-3.47352100	-1.65995400

H	-1.69208200	-2.72085000	-1.57304800
Ni	0.28043200	0.03751600	-0.09308700
P	0.38554700	2.20399800	0.43597900
C	-1.05430600	2.73586900	1.43654900
H	-1.17613000	2.05574600	2.29754000
H	-1.97446500	2.65561700	0.83261500
H	-0.94487500	3.77222000	1.80630800
C	1.75961200	2.68619500	1.56116400
H	2.73861600	2.58649300	1.06113700
H	1.76686400	2.02156400	2.44294600
H	1.65750700	3.72919200	1.91408300
C	0.46189500	3.55995800	-0.80415300
H	-0.42336700	3.50924300	-1.46080600
H	1.35767500	3.45660600	-1.44057200
H	0.48166800	4.55605200	-0.32429200
P	2.35639700	-0.45375000	-0.47935600
C	3.56671800	-0.72617900	0.87420000
H	3.24858300	-1.57972400	1.49573000
H	3.61475300	0.16205100	1.52714600
H	4.57949400	-0.93131200	0.48125200
C	2.66062800	-1.92730400	-1.53202400
H	2.07641900	-1.83834600	-2.46443400
H	2.32216000	-2.84372500	-1.02067400
H	3.72961200	-2.03842200	-1.79210700
C	3.23918100	0.81197400	-1.48679600
H	3.33869500	1.75691100	-0.92557900
H	2.65790700	1.02831500	-2.40045500
H	4.24982900	0.47805700	-1.78686000

IM2'

C	-3.44821500	-1.12977400	0.90192200

C	-2.34952600	-0.86603400	0.08373900
C	-2.35017000	0.28287600	-0.74040600
C	-3.43987900	1.16060300	-0.69495200
C	-4.52147200	0.90018900	0.15418100
C	-4.53439400	-0.24541600	0.94798700
H	-3.45754600	-2.02063400	1.54694200
H	-3.44919100	2.05610700	-1.33279100
H	-5.36482300	1.60147600	0.19259600
H	-5.38112000	-0.44275600	1.61687200
Si	-0.67490600	-1.74748500	-0.06933200
C	-1.13351500	0.46335100	-1.58276400
H	-1.15145300	-0.26307600	-2.42483700
H	-1.10169200	1.46147500	-2.06366500
C	-0.32927000	-2.50786000	1.64666600
H	0.60875200	-3.08929200	1.71918000
H	-0.30297800	-1.72860500	2.43195700
H	-1.14889000	-3.20299700	1.91674900
C	-0.83499200	-3.20194600	-1.27976000
H	0.09641400	-3.78986900	-1.37650800
H	-1.62889700	-3.89701200	-0.94381500
H	-1.11315400	-2.85647700	-2.29184300
Ni	0.55037700	0.08093000	-0.57784800
P	0.80430300	2.17998100	0.07677200
C	2.33440700	3.09056100	0.55446300
H	2.98560600	2.45665000	1.18128400
H	2.90948100	3.37674800	-0.34378300
H	2.10452300	4.01093200	1.12319300
C	-0.02973400	2.02317600	1.70914200
H	-1.05178500	1.62924300	1.56094100
H	0.51130500	1.30445400	2.34946200

H	-0.09414700	2.99261600	2.23938000
C	-0.16605600	3.55123200	-0.66249200
H	0.19299900	3.78073300	-1.68074100
H	-1.22507000	3.25212500	-0.73864300
H	-0.10515700	4.46967600	-0.05075500
P	2.42326300	-0.80199600	0.01343000
C	2.75965300	-2.54620000	-0.45248200
H	2.07576700	-3.22918200	0.07804300
H	2.58218900	-2.67788000	-1.53387200
H	3.80106900	-2.84084300	-0.22564000
C	2.95627700	-0.80540300	1.77186100
H	2.99852900	0.22641800	2.16143900
H	2.22722500	-1.36293700	2.38410400
H	3.95211400	-1.26872400	1.89843300
C	3.86475000	0.00414700	-0.79880800
H	3.73827400	-0.03108800	-1.89478800
H	3.93155700	1.06568800	-0.50895100
H	4.81753700	-0.49500200	-0.54237800

IM3

Si	-1.15896900	-1.83862600	1.56294000
C	0.58995800	-2.45519900	1.12985300
H	0.73479900	-3.43430400	1.63014400
H	1.33501600	-1.75860400	1.56836800
C	-1.14152000	-1.42789500	3.41194600
H	-2.08208600	-0.97033800	3.76899400
H	-0.99109400	-2.35282100	4.00091400
H	-0.31762400	-0.74162200	3.68072100
C	-2.36618400	-3.25260600	1.27610000
H	-2.09271000	-4.15001000	1.86201400
H	-3.40054100	-2.97723800	1.55131300

H	-2.38318800	-3.54066400	0.20872700
Ni	1.23457900	0.20057800	0.01809500
C	-1.63593400	-0.39208100	0.45231900
C	-0.67521900	0.36749000	-0.14547100
C	-0.91839000	1.57410000	-0.96667200
C	-1.95363100	2.49355900	-0.69134800
C	-0.00501900	1.93187600	-1.98199400
C	-2.06801500	3.69191000	-1.39235300
H	-2.67700900	2.26619500	0.09997200
C	-0.12707400	3.12192800	-2.69550400
H	0.82054200	1.24528400	-2.20763700
C	-1.15996600	4.01353700	-2.40306500
H	-2.88175100	4.38483000	-1.14575700
H	0.59650600	3.35817900	-3.48523900
H	-1.25583000	4.95482500	-2.95683100
C	-3.08209600	-0.24656900	0.15556900
C	-4.03569300	-0.12410500	1.18120000
C	-3.54803900	-0.26844500	-1.17142500
C	-5.39455500	0.00287400	0.89504800
H	-3.69790900	-0.10860000	2.22691600
C	-4.90454400	-0.14574900	-1.45970600
H	-2.81809200	-0.38876500	-1.98248100
C	-5.83589700	-0.00378600	-0.42849900
H	-6.11624400	0.10890300	1.71432800
H	-5.24181700	-0.16609600	-2.50322300
H	-6.90403700	0.09227700	-0.65602200
P	3.41756900	-0.13132900	-0.22966500
C	4.06268400	-1.51551600	0.78368600
H	3.92563900	-1.31002200	1.85946100
H	3.49246400	-2.42905500	0.53884000

H	5.13521900	-1.70074600	0.58992400
C	4.64768400	1.19487300	0.11702200
H	4.48305800	2.04113200	-0.57301400
H	4.56014600	1.58092100	1.14631400
H	5.68193200	0.83216900	-0.02645000
C	3.98836400	-0.63139600	-1.89640200
H	3.55466600	-1.60835700	-2.16481400
H	3.65279700	0.09794100	-2.65405600
H	5.09083900	-0.69859200	-1.93245900
C	0.74513100	-2.68672300	-3.15339900
C	0.58258000	-3.85416800	-2.41051000
C	0.57961300	-3.77946800	-1.01697700
C	0.74687900	-2.55380700	-0.35823200
C	0.95015300	-1.36584800	-1.10088700
C	0.92519500	-1.46369700	-2.49858800
H	0.73366500	-2.72306800	-4.25028000
H	0.44759800	-4.82015900	-2.91176300
H	0.42889500	-4.69172800	-0.42152500
H	1.06457200	-0.56849500	-3.12138600
P	1.26980000	1.73104400	1.62511900
C	1.88443700	3.40122500	1.17813600
H	2.90656600	3.35357500	0.76784800
H	1.22882800	3.81600400	0.39145900
H	1.87922400	4.09014000	2.04282800
C	2.28159400	1.30088500	3.09827400
H	1.89957000	0.36599200	3.54517100
H	3.33667400	1.12802100	2.82800300
H	2.24400500	2.09328500	3.86794100
C	-0.31141100	2.17926800	2.42926600
H	-0.97280800	2.66522900	1.69275300

H	-0.83392100	1.27957500	2.79392200
H	-0.14625200	2.87486800	3.27210900

IM3'

C	0.91299800	-0.59349300	-2.02589000
C	1.45762100	-1.73866100	-1.29583200
Si	-1.23789600	-2.45294400	-0.46070000
C	0.64546600	-2.58554300	-0.47183500
C	-1.94610100	-3.00100200	-2.12287100
H	-3.04485500	-2.87154100	-2.13133200
H	-1.73472500	-4.06856300	-2.31749900
H	-1.54075100	-2.42243700	-2.97130400
C	-1.94416200	-3.65975100	0.80944100
H	-1.60444000	-4.69150300	0.60033100
H	-3.04746700	-3.67428500	0.74589000
H	-1.67794500	-3.42655800	1.85627200
Ni	1.09641200	0.23449700	-0.23091800
P	2.95150400	1.43547200	-0.66782700
C	4.69427800	0.95440900	-0.31204000
H	4.97572100	0.07290000	-0.91224400
H	4.81627200	0.67644500	0.74789800
H	5.39862400	1.77551100	-0.54217000
C	3.14378000	1.98090800	-2.41039200
H	2.20133100	2.42033500	-2.77893200
H	3.38319100	1.11918400	-3.05695300
H	3.95105300	2.72840700	-2.51599000
C	2.88796100	3.05309300	0.20230700
H	3.12855700	2.90762900	1.26968800
H	1.87331200	3.48582500	0.14062700
H	3.61437200	3.77553900	-0.21355900
C	-0.82711800	0.31912800	-0.19339100

C	-1.76057400	-0.66416000	-0.18123600
C	-1.12872800	1.75971400	-0.14735700
C	-0.65180200	2.62410100	-1.15364900
C	-1.86731400	2.33208200	0.90674800
C	-0.89652200	3.99365200	-1.10629800
H	-0.09731800	2.18533200	-1.99398900
C	-2.09253800	3.70631900	0.96626000
H	-2.27662000	1.67494700	1.68576100
C	-1.60619400	4.54661600	-0.03641500
H	-0.52850400	4.64062600	-1.91262400
H	-2.66680800	4.12605000	1.80140700
H	-1.78912400	5.62644400	0.00872100
C	-3.21944700	-0.37549900	-0.17915900
C	-3.80761200	0.42462400	-1.17456000
C	-4.06017000	-0.89910800	0.81847700
C	-5.17331700	0.69807000	-1.16707400
H	-3.17001600	0.83654200	-1.96710200
C	-5.42724000	-0.62297900	0.83109700
H	-3.62328400	-1.51554100	1.61543700
C	-5.99127700	0.17670100	-0.16300300
H	-5.60586800	1.32546800	-1.95595000
H	-6.05797500	-1.03472800	1.62853900
H	-7.06614900	0.39239100	-0.15655800
H	1.57195100	-0.20510600	-2.82021900
H	-0.12010600	-0.68880200	-2.38827500
C	1.28231300	-3.55998300	0.31162300
H	0.67310400	-4.21011300	0.95551400
C	2.85862200	-1.96129300	-1.30287800
H	3.47533200	-1.34295900	-1.97045200
C	3.45586100	-2.94279300	-0.52197000

H	4.54320100	-3.08740900	-0.56233700
C	2.66765200	-3.74636500	0.30566900
H	3.12492900	-4.52671200	0.92540000
P	1.19399000	0.13878400	1.98438600
C	0.92170000	1.62724300	3.02133200
H	-0.06364900	2.06802300	2.79320800
H	1.68013000	2.39881900	2.81091000
H	0.96228400	1.37950600	4.09799100
C	0.02328200	-1.03467500	2.75730100
H	0.20691200	-2.04655400	2.35682300
H	-1.01468700	-0.75675100	2.50541300
H	0.14215200	-1.05715400	3.85611400
C	2.79129500	-0.47880100	2.63983000
H	3.57595700	0.28600500	2.50886700
H	3.09657900	-1.38319300	2.08285400
H	2.72764300	-0.72586700	3.71551900

TS1

C	-2.03986200	0.47365400	-1.55116700
C	-2.28528100	0.16848800	-0.19737000
C	-3.36271800	-0.68726500	0.15612400
C	-4.22440500	-1.18966800	-0.81250700
C	-3.95058400	-0.90885000	-2.16098200
C	-2.87064600	-0.10875500	-2.52670900
H	-1.30538600	1.22658000	-1.86030300
H	-5.09001700	-1.80614600	-0.53802100
H	-4.60834200	-1.31270900	-2.94096200
H	-2.69514900	0.11073200	-3.58685100
Si	-1.97030200	0.50590700	1.66521500
C	-3.33967000	-0.83154100	1.65808600
H	-4.30092200	-0.60380300	2.15601800

H	-3.03612100	-1.84496400	1.98951300
C	-0.47368600	0.14116800	2.74996900
H	-0.00672700	-0.83744400	2.54922400
H	0.30848700	0.91262200	2.63358700
H	-0.80529100	0.15161500	3.80883600
C	-2.60642300	2.19452000	2.19218100
H	-3.48489900	2.51526800	1.60510100
H	-2.90139900	2.17487300	3.25833900
H	-1.82754600	2.97144400	2.08692000
Ni	-0.22631500	-0.33907900	-0.21076500
P	-0.20014100	-2.53349700	-0.23676000
C	-1.63039200	-3.48377800	-0.88635000
H	-2.50632000	-3.35959200	-0.22716900
H	-1.91582700	-3.10628200	-1.88330800
H	-1.39729100	-4.56198800	-0.96462800
C	0.09018800	-3.43218500	1.34188400
H	1.03970900	-3.10208400	1.79690200
H	-0.72140700	-3.21820500	2.05947300
H	0.14082500	-4.52589400	1.18581400
C	1.15422300	-3.22026100	-1.26763800
H	1.05976300	-2.85058600	-2.30332900
H	2.13263200	-2.88257400	-0.88417400
H	1.13934200	-4.32567900	-1.28245400
C	0.83610900	1.23237000	-0.23818400
C	1.58167400	0.17992900	-0.09125900
C	0.82842800	2.67173100	-0.32887800
C	-0.37734500	3.39962400	-0.36060300
C	2.03908600	3.39672000	-0.38561500
C	-0.37595000	4.79047900	-0.44111000
H	-1.32870900	2.85509900	-0.31471600

C	2.03756200	4.78419300	-0.46639900
H	2.98651700	2.84476200	-0.35967600
C	0.83018300	5.48947900	-0.49511700
H	-1.32773800	5.33481200	-0.46192100
H	2.98955100	5.32726400	-0.50647700
H	0.83210100	6.58377600	-0.55945500
C	2.88631100	-0.42405500	0.02002700
C	3.82135300	-0.31724500	-1.02871100
C	3.24727400	-1.17587400	1.15511200
C	5.05789600	-0.95224000	-0.94874700
H	3.55276800	0.26307800	-1.91967800
C	4.48985400	-1.79827000	1.23644900
H	2.53596200	-1.25145300	1.98703400
C	5.39882300	-1.69865900	0.18109100
H	5.76688900	-0.86299000	-1.78067700
H	4.75283900	-2.37038600	2.13434200
H	6.37287200	-2.19744200	0.24103800

TS1'

C	-3.63233800	-0.84437000	0.96968700
C	-2.35205500	-0.94409100	0.42364400
C	-2.15392700	-0.74371700	-0.95755000
C	-3.23442700	-0.47806900	-1.80267400
C	-4.50958600	-0.36033700	-1.24237800
C	-4.70825800	-0.53369300	0.13170800
H	-3.80745000	-0.99834200	2.04346400
H	-3.08744900	-0.33413300	-2.88141800
H	-5.36602800	-0.12262100	-1.88562200
H	-5.71773800	-0.43084700	0.54870500
Si	-0.54684700	-1.34658400	0.76417700
C	-0.68492300	-0.85152300	-1.27871400

H	-0.42444200	-1.75518800	-1.86682700
H	-0.28096800	0.02211300	-1.84200800
C	0.24697800	-0.67305800	2.37311300
H	0.74669300	-1.49396300	2.92188500
H	1.01247400	0.11274800	2.19955400
H	-0.52242400	-0.23288000	3.03470500
C	-0.29803400	-3.21747100	0.78123600
H	0.77648800	-3.47940600	0.81660200
H	-0.77090400	-3.67330000	1.67155800
H	-0.73367000	-3.70840900	-0.10705100
Ni	0.77648000	0.17507000	-0.12592800
P	0.05590000	2.20815200	0.17714200
C	1.16480500	3.64264800	-0.16390200
H	2.06114600	3.58024500	0.47781200
H	1.51059400	3.60725400	-1.21209200
H	0.67111400	4.61813600	0.00945100
C	-0.53051700	2.64897600	1.86513200
H	-1.36495000	1.98330900	2.14837000
H	0.27426200	2.51159100	2.60785800
H	-0.88422700	3.69564500	1.91739600
C	-1.42674800	2.72424300	-0.79019400
H	-1.24913000	2.58625900	-1.87171600
H	-2.28612800	2.08672000	-0.51567000
H	-1.69779800	3.78205400	-0.61192100
P	2.76425900	-0.57142800	-0.38221000
C	2.96373500	-1.94523300	-1.59873700
H	2.31600000	-2.79244500	-1.31186700
H	2.63417600	-1.60869800	-2.59771700
H	4.00728900	-2.30502300	-1.67651900
C	3.59339300	-1.32543300	1.08075000

H	3.70532000	-0.57457600	1.88226900
H	2.97656200	-2.14718600	1.48458300
H	4.59379600	-1.72666700	0.83204200
C	4.11826500	0.53922800	-0.96508800
H	3.83936800	0.99355800	-1.93208800
H	4.25903100	1.36383700	-0.24451200
H	5.08284300	0.01097700	-1.09189400

TS2

C	-2.89569500	-0.84872500	-1.55490900
C	-2.29565900	-0.28498100	-0.41305100
C	-3.12799700	0.47491800	0.44302100
C	-4.49348700	0.62575600	0.15733100
C	-5.06078200	0.04699800	-0.97587300
C	-4.25116900	-0.68568000	-1.84543300
H	-2.28360000	-1.43095300	-2.26201600
H	-5.12232800	1.21865100	0.83635600
H	-6.12967800	0.17466800	-1.18587200
H	-4.67828300	-1.13374100	-2.75206700
Si	-0.67261700	0.69686800	1.74062000
C	-2.50258600	1.16529400	1.62262500
H	-3.00524300	0.90703000	2.57882600
H	-2.60686000	2.26741000	1.54109500
C	0.27581200	2.10843100	2.56258000
H	1.36429600	1.91970400	2.59920400
H	-0.07773400	2.19450700	3.60882600
H	0.12216600	3.08828100	2.07889100
C	-0.36822600	-0.75803300	2.91783400
H	-0.59034200	-0.44142200	3.95668100
H	0.69280900	-1.06833700	2.89291600
H	-0.99156500	-1.64196600	2.69921200

Ni	-0.38364800	-0.55368800	-0.15029900
P	-0.45357700	-2.76116100	-0.11236200
C	0.84311100	-3.58828900	0.87963200
H	0.74579100	-3.30113900	1.93996300
H	1.84268200	-3.26614000	0.53844900
H	0.77565800	-4.68909500	0.80607200
C	-1.98636200	-3.52945400	0.52492000
H	-2.84061100	-3.20906900	-0.09594800
H	-2.18717400	-3.18531800	1.55364600
H	-1.92539300	-4.63298300	0.52353700
C	-0.27958200	-3.61735000	-1.72523500
H	0.66741100	-3.33522500	-2.21459100
H	-1.10349200	-3.32902400	-2.39913100
H	-0.29946600	-4.71532100	-1.60026100
C	1.44756300	-0.05010800	-0.22842500
C	0.65862900	1.01499900	-0.14514800
C	2.82786900	-0.44702500	-0.29039000
C	3.25421200	-1.50147800	-1.12381500
C	3.79659600	0.18257900	0.52154800
C	4.58891400	-1.89031800	-1.16520400
H	2.51263700	-2.00069600	-1.75954000
C	5.12620600	-0.22686700	0.49818200
H	3.48085700	0.99254100	1.19101600
C	5.53173400	-1.25962200	-0.34980500
H	4.89945700	-2.69985400	-1.83633600
H	5.85841200	0.27058600	1.14521400
H	6.58119900	-1.57432100	-0.37408700
C	0.63984300	2.42777700	-0.49249000
C	-0.58269100	3.07480200	-0.74707100
C	1.82803400	3.17497200	-0.58296900

C	-0.61713700	4.42898100	-1.06744900
H	-1.50881700	2.48522600	-0.70861200
C	1.79007900	4.52857700	-0.90468200
H	2.78884900	2.67952300	-0.39874900
C	0.56826800	5.16319400	-1.14109500
H	-1.57862700	4.91650800	-1.26679400
H	2.72497100	5.09781000	-0.96940900
H	0.54147500	6.23059400	-1.38915500

TS2'

C	-1.90665000	-0.53384200	-1.38406400
C	-3.10174700	-0.31919500	-0.52096500
Si	-1.09760400	0.36724900	1.24107300
C	-2.90340600	0.27643200	0.74297300
C	-0.72691000	1.94919600	2.20268300
H	0.34457700	2.03159200	2.46190300
H	-1.29072200	1.90886300	3.15537200
H	-1.01749100	2.87799500	1.68460300
C	-0.64928700	-0.98997000	2.49047300
H	-1.12683500	-0.76584500	3.46521700
H	0.44427300	-1.02246500	2.65642600
H	-0.98005900	-1.99787100	2.18815100
Ni	-0.09614200	-0.60975400	-0.53617200
P	0.01147300	-2.80564400	-0.57206100
C	1.19701400	-3.64147700	0.54344500
H	0.93521400	-3.42660500	1.59343900
H	2.21582700	-3.25144400	0.37487700
H	1.20179400	-4.73723600	0.39800900
C	-1.54553600	-3.70142400	-0.21409100
H	-2.32563500	-3.39776300	-0.93248100
H	-1.91727400	-3.44541300	0.79256100

H	-1.41152600	-4.79686500	-0.27383200
C	0.45255100	-3.50995900	-2.20578800
H	1.45575900	-3.16931100	-2.51275800
H	-0.26503900	-3.15766300	-2.96665800
H	0.44161200	-4.61509900	-2.19468800
C	1.63034900	0.09257100	-0.17597300
C	0.73354400	1.05596600	-0.30312500
C	3.01294800	-0.16283300	0.12937400
C	3.75072300	-1.14737700	-0.55840000
C	3.66403000	0.54927600	1.15974400
C	5.08388800	-1.39430500	-0.24666400
H	3.25784700	-1.70700400	-1.36348600
C	4.99000800	0.28192400	1.48644200
H	3.10067600	1.31232100	1.71228700
C	5.70928500	-0.68621900	0.78210400
H	5.64280300	-2.15253000	-0.80798800
H	5.47216400	0.84098400	2.29723900
H	6.75606500	-0.88925100	1.03523900
C	0.61686600	2.47184100	-0.59499300
C	-0.62403900	3.04435100	-0.92882800
C	1.75374400	3.30257200	-0.58396000
C	-0.72761700	4.39738600	-1.23658300
H	-1.51936000	2.40723900	-0.93267800
C	1.64860100	4.65488200	-0.89481400
H	2.72971100	2.86763400	-0.33597700
C	0.40833500	5.20922000	-1.22007000
H	-1.70452400	4.82403800	-1.49224600
H	2.54504500	5.28616200	-0.88224200
H	0.32766900	6.27531700	-1.46189900
H	-2.07995700	-1.36890800	-2.09678900

H	-1.74676000	0.36344900	-2.02126100
C	-4.00516000	0.61683000	1.54082300
H	-3.85169400	1.09713300	2.51781200
C	-4.41174000	-0.64000000	-0.91603100
H	-4.58039600	-1.12151000	-1.88903600
C	-5.49759200	-0.32971000	-0.09989800
H	-6.51418900	-0.58287200	-0.42600300
C	-5.30119100	0.31498900	1.12519300
H	-6.15930300	0.56246500	1.76154100

TS3

Si	-0.37059800	-0.81066500	-2.35799000
C	0.91367600	0.56325900	-2.73058700
H	0.81988500	0.88212300	-3.78742000
H	1.90176200	0.05454500	-2.66498600
C	0.61532300	-2.41868800	-2.18656800
H	-0.02134800	-3.30684300	-2.01661700
H	1.20378300	-2.61092200	-3.10539500
H	1.33365900	-2.34157200	-1.34635800
C	-1.52179800	-0.99023000	-3.83721700
H	-0.94622100	-1.12562500	-4.77205500
H	-2.20605600	-1.85223400	-3.74444200
H	-2.15327600	-0.09162400	-3.96286800
Ni	1.18018500	-0.19718800	0.36019500
C	-1.32653000	-0.25190700	-0.84943000
C	-0.64474700	0.40866900	0.15658100
C	-1.37380500	1.06254500	1.27740400
C	-1.76687800	0.33660100	2.41093700
C	-1.75059800	2.41680100	1.21782400
C	-2.49236300	0.92767900	3.44437600
H	-1.52324200	-0.72815000	2.47427700

C	-2.46543000	3.01587900	2.25291000
H	-1.49866000	2.99892800	0.32413700
C	-2.83783200	2.27686000	3.37598300
H	-2.79524700	0.32379800	4.30841900
H	-2.74659200	4.07298000	2.17269500
H	-3.40467800	2.74700000	4.18797000
C	-2.78890400	-0.48551300	-0.83918900
C	-3.27433600	-1.78925000	-1.06716200
C	-3.74766600	0.52639100	-0.63934800
C	-4.63734600	-2.07664100	-1.06829400
H	-2.55181800	-2.60072700	-1.23696800
C	-5.11136300	0.24246400	-0.64102600
H	-3.41600600	1.56005800	-0.50206900
C	-5.56738800	-1.06020000	-0.84895500
H	-4.97599100	-3.10558000	-1.24117100
H	-5.83023800	1.05642500	-0.48643700
H	-6.64133900	-1.28002100	-0.84885400
P	3.36654000	-0.17309300	0.02932400
C	4.23728900	-1.51219500	-0.88550300
H	4.03975300	-2.49878100	-0.43450000
H	3.86958300	-1.54635500	-1.92612200
H	5.33077600	-1.34863300	-0.90796800
C	4.32187500	-0.12084500	1.59983300
H	3.97780400	0.73749300	2.20378700
H	4.14949300	-1.03431000	2.19488800
H	5.40882600	-0.01468000	1.42672700
C	4.05592300	1.29718500	-0.81939600
H	3.70184900	1.33938000	-1.86359400
H	3.70174300	2.21851900	-0.32488200
H	5.16119300	1.28436200	-0.81921100

C	1.31430000	3.90703800	-0.05487700
C	1.31722400	4.12424400	-1.43296000
C	1.12968900	3.04171200	-2.29938600
C	0.93758000	1.74578800	-1.81613800
C	0.85454000	1.52772500	-0.41386000
C	1.06691400	2.62819800	0.44361700
H	1.48152900	4.74139400	0.63802300
H	1.47788500	5.13051500	-1.83752000
H	1.17095000	3.20316500	-3.38559700
H	1.01371900	2.47938000	1.53133700
P	0.91605100	-2.03187600	1.58378500
C	0.96326000	-1.82142300	3.41099200
H	1.97202000	-1.48353900	3.70683700
H	0.25272300	-1.04700400	3.74313200
H	0.74162700	-2.76568300	3.94177900
C	2.12614900	-3.41399500	1.42682700
H	2.16215600	-3.77113700	0.38255600
H	3.14130300	-3.08707300	1.71143300
H	1.84958800	-4.26859500	2.07206000
C	-0.61892100	-2.99960500	1.29552800
H	-1.51561400	-2.35862700	1.27811400
H	-0.54834400	-3.47105800	0.29980300
H	-0.75164400	-3.79747700	2.04932000

TS3'

C	-0.52029300	0.51862600	-1.77735100
C	-1.24060000	1.76574900	-1.36263800
Si	0.80905200	2.33010100	0.63676700
C	-0.84669600	2.55011400	-0.24923800
C	1.81426400	3.89105800	0.30687200
H	2.77980100	3.88807300	0.84418200

H	1.26295500	4.79669100	0.62111500
H	2.04358900	3.99740600	-0.76882700
C	0.45406200	2.29193600	2.49663900
H	-0.00281800	3.24572400	2.82274400
H	1.36857800	2.14847300	3.10133400
H	-0.25423400	1.48706000	2.76302300
Ni	-1.02358000	-0.49186500	-0.01298300
P	-3.19704400	-0.91029200	-0.26567900
C	-4.33897600	0.33422400	0.44898400
H	-4.12880900	1.32129600	0.00076600
H	-4.16127800	0.42379400	1.53494400
H	-5.40194700	0.07904400	0.28150600
C	-3.83689800	-1.06060000	-1.98248900
H	-3.31093400	-1.87969900	-2.50457800
H	-3.66025700	-0.13072200	-2.54593300
H	-4.92117400	-1.27471300	-1.99797700
C	-3.89733100	-2.47041600	0.41476400
H	-3.75949500	-2.53106700	1.50747800
H	-3.38642700	-3.33860300	-0.03741100
H	-4.97898100	-2.55461800	0.20119000
C	0.75652200	-0.15363000	-0.57930400
C	1.61946700	0.82280300	-0.08414300
C	1.17733800	-1.52741500	-0.95410500
C	0.55737100	-2.28562000	-1.96976900
C	2.15857300	-2.18701100	-0.17971400
C	0.89343300	-3.62084300	-2.19276800
H	-0.19122400	-1.82418200	-2.62066200
C	2.48850800	-3.51959600	-0.39936300
H	2.67331200	-1.63209400	0.61084400
C	1.85601100	-4.25229500	-1.40680400

H	0.39363200	-4.17281400	-2.99805500
H	3.25730300	-3.99271300	0.22382000
H	2.11812200	-5.30197900	-1.58201400
C	3.07850500	0.76905000	-0.21990100
C	3.70766000	0.27465600	-1.38422800
C	3.92613500	1.24745100	0.80310400
C	5.09267000	0.24566300	-1.50799800
H	3.08431500	-0.08343000	-2.21355700
C	5.31307400	1.22241700	0.67947700
H	3.47656300	1.63145100	1.73034200
C	5.90966800	0.71682400	-0.47673100
H	5.54404200	-0.14180600	-2.42993800
H	5.93706700	1.59771600	1.50013800
H	7.00097300	0.69581700	-0.57682900
H	-1.19829100	-0.11043600	-2.38782600
H	0.33116900	0.70886100	-2.45411100
C	-1.65499300	3.64030800	0.11352900
H	-1.36892600	4.25147300	0.98216100
C	-2.36399500	2.16959200	-2.10925200
H	-2.62263000	1.61600100	-3.02149800
C	-3.13749000	3.26686700	-1.73898700
H	-4.00552100	3.55343200	-2.34559100
C	-2.79751200	3.99873100	-0.60167100
H	-3.40071300	4.86033900	-0.29179300
P	-0.63003400	-1.36083000	1.95690400
C	-0.64297800	-3.19795600	1.91908400
H	0.11101800	-3.54890900	1.19266800
H	-1.62486200	-3.57478200	1.58857700
H	-0.41263900	-3.63214100	2.90964400
C	0.99009900	-1.02720300	2.77306400

H	0.84767900	-0.59259800	3.77720100
H	1.57229500	-0.30905500	2.16535800
H	1.58117800	-1.95299700	2.87675500
C	-1.79837000	-1.03145900	3.34146100
H	-2.82644300	-1.32057200	3.06417200
H	-1.81244000	0.04693300	3.57725900
H	-1.51724600	-1.58419100	4.25666300

5. Supplementary References

1. Chen, H. et al. Rhodium-Catalyzed R reaction of silacyclobutanes with unactivated alkynes to afford silacyclohexenes. *Angew. Chem. Int. Ed.* **58**, 4695-4699 (2019).
2. Ishida, N., Okumura, S. & M. Murakami, Site- and Regio-selective Incorporation of Carbon Dioxide into the C(sp2)–Si Bond of Benzosilacyclobutenes . *Chem. Lett.* **47**, 570 (2018).
3. Park, K., Bae, G., Moon, J., Choe, J., Song, K. & Lee, S. Synthesis of Symmetrical and Unsymmetrical Diarylalkynes from Propiolic Acid Using Palladium-Catalyzed Decarboxylative Coupling. *J. Org. Chem.* **75**, 6244–6251 (2010).
4. Dailler, D., Rocaboy, R. & Baudoin, O. Synthesis of β-lactams by palladium(0)-Catalyzed C(sp3)−H carbamoylation. *Angew. Chem. Int. Ed.* **56**,7218 –7222 (2017).
5. Sieber, J. D. & Morken, J. P. Asymmetric Ni-Catalyzed conjugate allylation of activated enones. *J. Am. Chem. Soc.* **130**, 4978–4983 (2008).
6. Hong, K. & Morken, J. P. Catalytic enantioselective diboration of cyclic dienes. a modified ligand with general utility. *J. Org. Chem.* **76**, 9102–9108 (2011).
7. Frisch, M. J., Trucks, G. W., Schlegel, H. B., Scuseria, G. E., Robb, M. A., Cheeseman, J. R., Scalmani, G., Barone, V., Petersson, G. A., Nakatsuji, H., Li, X., Caricato, M., Marenich, A. V., Bloino, J., Janesko, B. G., Gomperts, R., Mennucci, B., Hratchian, H. P., Ortiz, J. V., Izmaylov, A. F., Sonnenberg, J. L., Williams - Young, D., Ding, F., Lipparini, F., Egidi, F., Goings, J., Peng, B., Petrone, A., Henderson, T., Ranasinghe, D., Zakrzewski, V. G., Gao, J., Rega, N., Zheng, G., Liang, W., Hada, M., Ehara, M., Toyota, K., Fukuda, R., Hasegawa, J., Ishida, M., Nakajima, T., Honda, Y., Kitao, O., Nakai, H., Vreven, T., Jr. Throssell, K., Montgomery, J. A., Peralta, J. E., Ogliaro, F., Bearpark, M. J., Heyd, J. J., Brothers, E. N., Kudin, K. N., Staroverov, V. N., Keith, T. A., Kobayashi, R., Normand, J., Raghavachari, K., Rendell, A. P., Burant, J. C., Iyengar, S. S., Tomasi, J., Cossi, M., Millam, J. M., Klene, M., Adamo, C., Cammi, R., Ochterski, J. W., Martin, R. L., Morokuma, K., Farkas, O., Foresman, J. B. & Fox, D. J. Gaussian 16, Revision A.03; Gaussian, Inc.: Wallingford, CT, **2016**.
8. Grimme, S. Exploration of chemical compound, conformer, and reaction space with meta-dynamics simulations based on tight-binding quantum chemical calculations. *J. Chem. Theory Comput.* **15**, 2847-2862 (2019).
9. Pracht, P., Bohle, F. & Grimme, S. Automated exploration of the low-energy chemical space with fast quantum chemical methods. *Phys. Chem. Chem. Phys.* **22**, 7169-7192 (2020). Grimme, S. et al. Fully automated quantum-chemistry-based computation of spin–spin-coupled nuclear magnetic resonance spectra. *Angew. Chem. Int. Ed.* **56**, 14763-14769 (2017).
10. Bannwarth, C., Ehlert, S. & Grimme, S. GFN2-xTB—an accurate and broadly parametrized self-consistent tight-binding quantum chemical method with multipole electrostatics and density-dependent dispersion contributions. *J. Chem. Theory Comput.* **15**, 1652–1671 (2019).
11. Becke, A. D., Density - functional thermochemistry. III. The role of exact exchange. *J. Chem. Phys.* **98**, 5648 (1993). Lee, C., Yang, W. & Parr, R. G. Development of the Colle-Salvetti correlation-energy formula into a functional of the electron density. *Phys. Rev. B*, **37**, 785 (1988). Vosko, S. H., Wilk, L. & Nusair, M. Accurate spin-dependent electron liquid correlation energies for local spin density calculations: a critical analysis. *Can. J. Phys*. **58**, 1200 (1980). Stephens, F., Devlin, J., Chabalowski, C. F. & Frisch, M. J. Ab Initio calculation of vibrational absorption and circular dichroism spectra using density functional force fields. *J. Phys. Chem.* 98, 11623–11627 (1994).
12. Zhao, Y. & Truhlar, D. G. The M06 suite of density functionals for main group thermochemistry, thermochemical kinetics, noncovalent interactions, excited states, and transition elements: two new functionals and systematic testing of four M06-class functionals and 12 other functionals. *Theor. Chem. Acc.* **120**, 215–241 (2008).
13. Lu, T. & Chen, F. Multiwfn: A multifunctional wavefunction analyser. *J. Comp. Chem.* **33**, 580-592 (2012).

14. Zhao, Y. & Truhlar, D. G. A new local density functional for main-group thermochemistry, transition metal bonding, thermochemical kinetics, and noncovalent interactions. *J. Chem. Phys.* **125**, 194101 (2006).
15. Weigend, F. & Ahlrichs, R. Balanced basis sets of split valence, triple zeta valence and quadruple zeta valence quality for H to Rn: Design and assessment of accuracy. *Phys. Chem. Chem. Phys. 7*, 3297-3305 (2005).
16. Adamo, C. & Barone, V. Toward reliable density functional methods without adjustable parameters: The PBE0 model. *J. Chem. Phys.* **110**, 6158 (1999). Grimme, S., Ehrlich, S. & Georigk, L. Effect of the damping function in dispersion corrected density functional theory. *J. Comp. Chem.* **32**, 1456-1465 (2011).
17. Marenich, A. V., Cramer, C. J. & D. G. Truhlar, D. G. Universal Solvation Model Based on Solute Electron Density and on a Continuum Model of the Solvent Defined by the Bulk Dielectric Constant and Atomic Surface Tensions. *J. Phys. Chem. B* **113**, 6378–6396 (2009).
18. Luchini, G., Alegre-Requena, J. V., Funes-Ardoiz, I. & Paton, R. S. GoodVibes: automated thermochemistry for heterogeneous computational chemistry data [version 1; peer review: 2 approved with reservations]. *F1000Research* **2020**, *9*, 291.
19. Grimme, S. Supramolecular Binding Thermodynamics by Dispersion-Corrected Density Functional Theory. *Chem. Eur. J.* **18**, 9955-9964 (2012). Li, Y., Gomes, J., Sharada, S. M., Bell, A. T. & Head-Gordon, M. Improved Force-Field parameters for QM/MM simulations of the energies of adsorption for molecules in zeolites and a free rotor correction to the rigid rotor harmonic oscillator model for adsorption enthalpies. *J. Phys. Chem. C* **119**, 1840–1850 (2015).
20. Delano, W. L. The PyMOL Molecular Graphics System Delano Scientific (2002).
21. Lefebvre, C., Rubez, G., Khartabil, H., Boisson, J.-C., Contreras-García, J. & Hénon, E. Accurately extracting the signature of intermolecular interactions present in the NCI plot of the reduced density gradient versus electron density. *Phys. Chem. Chem. Phys.* **19**, 17928-17936 (2017).
22. Lv, X., Zhang, X., Sa, R., Huang, F. & Lu, G. Computational exploration of substrate and ligand effects in nickel-catalyzed C–Si bond carboxylation with CO_2. *Org. Chem. Front.* **6**, 3629-3635 (2019).
